# Hydrogen-Bonding Ability of Noyori–Ikariya Catalysts Enables Stereoselective Access to CF_3_-Substituted *syn*-1,2-Diols via Dynamic Kinetic Resolution

**DOI:** 10.1021/acscatal.3c00980

**Published:** 2023-04-21

**Authors:** Maša Sterle, Matej Huš, Matic Lozinšek, Anamarija Zega, Andrej Emanuel Cotman

**Affiliations:** † Faculty of Pharmacy, University of Ljubljana, Aškerčeva cesta 7, SI-1000 Ljubljana, Slovenia; ‡ Jožef Stefan Institute, Jamova cesta 39, SI-1000 Ljubljana, Slovenia; § National Institute of Chemistry, Department of Catalysis and Chemical Reaction Engineering, Hajdrihova ulica 19, SI-1000 Ljubljana, Slovenia; # Association for Technical Culture of Slovenia, Zaloška cesta 65, SI-1000 Ljubljana, Slovenia; δ Institute for the Protection of Cultural Heritage of Slovenia, Poljanska 40, SI-1000 Ljubljana, Slovenia

**Keywords:** asymmetric catalysis, DFT, drug design, fluorine, hydrogenation, kinetic resolution, ruthenium

## Abstract

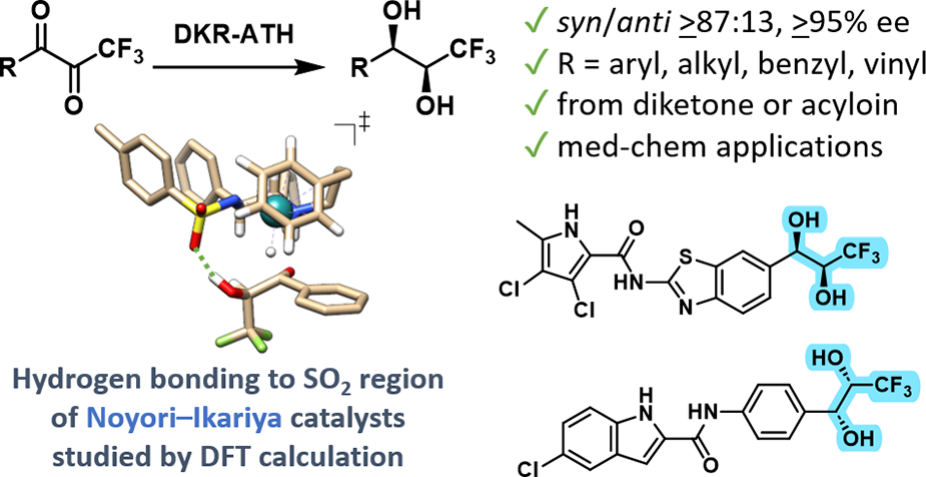

Stereopure CF_3_-substituted *syn*-1,2-diols were prepared via the reductive dynamic kinetic resolution of the corresponding racemic α-hydroxyketones in HCO_2_H/Et_3_N. (Het)aryl, benzyl, vinyl, and alkyl ketones are tolerated, delivering products with ≥95% ee and ≥87:13 *syn*/*anti*. This methodology offers rapid access to stereopure bioactive molecules. Furthermore, DFT calculations for three types of Noyori–Ikariya ruthenium catalysts were performed to show their general ability of directing stereoselectivity via the hydrogen bond acceptor SO_2_ region and CH/π interactions.

Dynamic kinetic resolution (DKR) is a powerful synthetic method for the conversion of stereoisomeric mixtures to valuable enantiomerically pure products. Efficient DKR relies on a fast dynamic interconversion of the starting material’s enantiomers and on a highly enantio-discriminating asymmetric transformation.^1,2^ Noyori–Ikariya type ruthenium catalysts for asymmetric transfer hydrogenation (ATH), for example **C1**–**C8** (Figure 1a),^3−8^ were used in efficient DKR of several classes of complex ketones.^9−11^ Traditionally, the origin of enantioselectivity for the reduction of ketones with this type of catalyst has been associated with the CH/π interaction between an electron-deficient η^6^-arene ligand and π electrons of the ketone substrate.^12^ Configuration at the α-stereocenter during DKR-ATH was deemed to be controlled by the steric repulsion (Figure 1b),^13−20^ although this assertion was rarely supported by computational analysis.^21,22^

**Figure 1:**
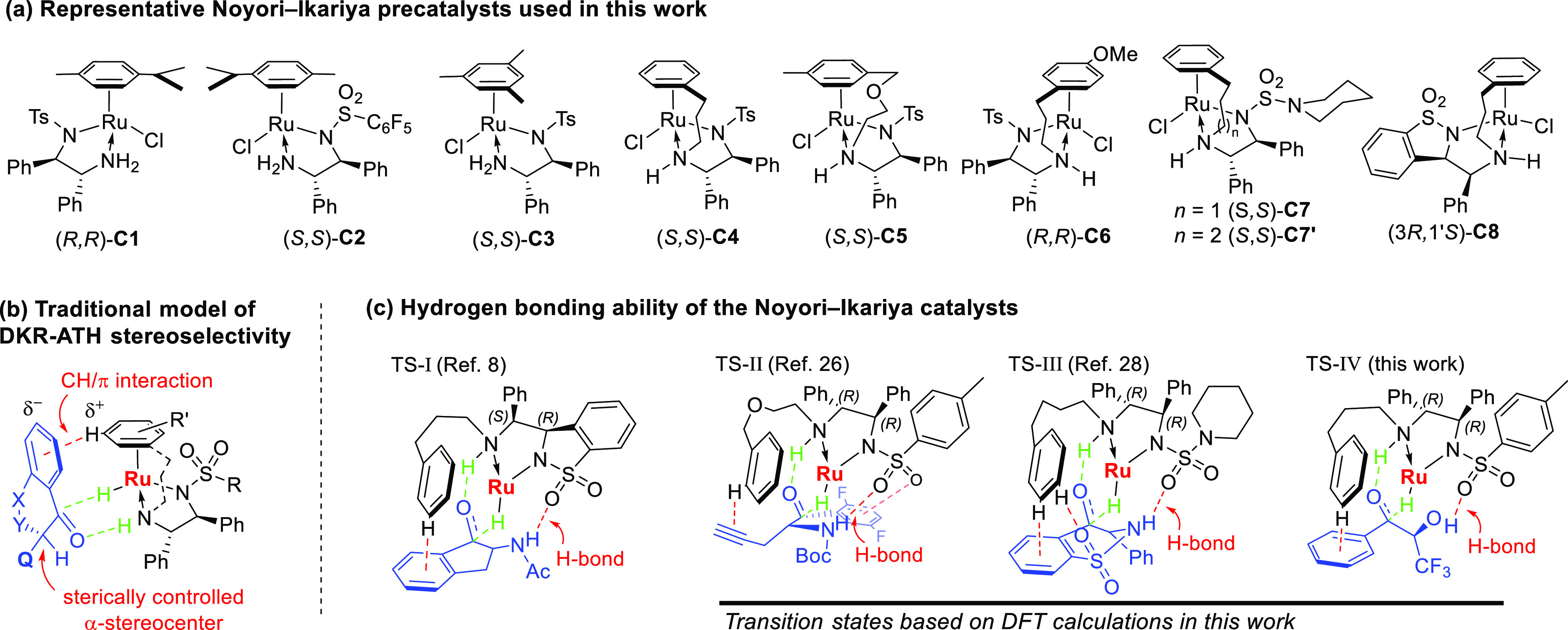
(a) Representative Noyori–Ikariya precatalysts used in this work; (b) Traditional model of DKR-ATH stereoselectivity; and (c) Hydrogen bonding ability of the Noyori–Ikariya catalysts.

In 2019, we highlighted another region of the Noyori–Ikaria type ruthenium catalysts that can be involved in stereochemical control at the α-stereocenter during DKR-ATH, i.e., the SO_2_ moiety.^8^ Indeed, the two-point catalyst–substrate attraction between catalyst **C8** and 2-acetamido-1-indanone was involved in the stabilization of the most favorable transition state: the commonly encountered CH/π interaction controlled the configuration at carbon-1 and configuration at carbon-2 was controlled by additional SO_2_/NHAc hydrogen bonding, delivering the unexpected *trans* product (Figure 1c, TS-I).^8^ This goes hand in hand with computational evidence that the SO_2_ region of the Noyori–Ikariya complexes contributes to enantioselectivity of acetophenone reduction by destabilization of the transition state leading to the minor stereomer via SO_2_/π repulsion.^23,24^

The rather niche ruthenium catalyst **C8** based on the *syn*-ULTAM ligand^25^ outperformed the more common *anti*-1,2-diphenylethylenediamine-based catalysts for the reduction of α-acetamido benzo-fused ketones. Therefore, we wondered whether the hydrogen bonding ability during DKR-ATH is limited to **C8** or is it a general feature of the Noyori–Ikariya family of ruthenium catalysts. Retrospectively, the hydrogen-bond-driven catalyst–substrate recognition might have been involved in DKR-ATH of other ketones with a hydrogen-bond donor α-substituent. In particular, DKR-ATH of linear α-(BocNH) arylketones afforded the unexpected enantiomer of omarigliptin intermediate,^26,27^ where the aromatic ring of the substrate was not involved in stabilization of the most favorable transition state (Figure 1c, TS-II). Furthermore, DKR-ATH of cyclic α-sulfonylamino ketones to the corresponding stereopure benzosultams employing **C7′** exhibited exceptional diastereo- and enantioselectivity and turnover number, which points to the energetically favorable catalyst–substrate interaction (Figure 1c, TS-III).^28^

To verify the general ability of the Noyori–Ikaria-type ruthenium catalysts to control the stereochemical outcome during DKR-ATH via hydrogen bonding to the SO_2_ moiety, the transition state geometries TS-II and TS-III were calculated for the aforementioned literature reductions involving structurally distinct **C5** and **C7′** (Figure 1c). DFT calculations in Gaussian 16^29^ were performed at the M06-2X/6-31+G(d,p)+LANL2DZ level.^30−32^ In both cases, a strong hydrogen bond was confirmed (2.36 and 2.01 Å, respectively) in the most favorable configuration. Interestingly, for the reaction in Figure 1c, TS-II, three attractive interactions were identified: SO/NH, CH/π, and SO/(difluorophenyl). Also, in TS-III, a third attractive interaction was identified between η^6^-arene ligand of the catalyst **C7′** and SO_2_ of the substrate. The coordinates and visualizations of TS-II and TS-III are reported in the Supporting Information (SI).

Hereafter we report an efficient asymmetric reduction of CF_3_-substituted 1,2-diketones **1** via DKR-ATH of intermediate α-hydroxy-α-CF_3_-ketones **2**. The latter are another example of ketones with a hydrogen-bond donor α substituent that might interact with the SO_2_ region of the catalyst. The resulting diols *syn*-**3** are valuable motifs in medicinal chemistry,^33−36^ but the literature reports on their asymmetric synthesis are limited to a few sporadic examples. Stereoselective synthesis of *syn-***3a** (98% ee) was achieved via Sharpless dihydroxylation.^37^*anti*-1-(2-Naphthyl)-2-CF_3_-1,2-ethandiol (89% ee) was prepared in three steps from the asymmetric diazo-aldol reaction product.^38^ Asymmetric aldol reaction was employed for the synthesis of trifluoromethylated sugar analogues.^39^ The pegylated 1-vinyl-2-CF_3_-glycol (96% ee) was prepared via alkoxyallylboration of fluoral with “ate” complex.^40^

The model 1-phenyl-2-trifluoromethylethandione **1a** in the form of a monohydrate was prepared from benzaldehyde via umpolung, acylation with trifluoroacetic anhydride, and hydrolysis.^41^ It was subjected to DKR-ATH using a range of Noyori–Ikariya-type ruthenium catalysts **C1**–**C8**, HCO_2_H/Et_3_N 3:2 as a source of hydrogen, and DMF as a cosolvent at 60 °C (Table 1). At a substrate/catalyst (S/C) ratio 1000, the reaction reached full conversion with tethered catalysts **C4**, **C5**, **C7**, and **C8**. The best stereoselectivity was achieved using HCO_2_H/Et_3_N in a 3:2 molar ratio, compared to 5:2 (azeotrope) and 3.4:2 (optimal for the reduction of benzils);^42^ see Table S1. The results with the most stereoselective catalysts **C4** and **C5** were back-to-back (Table S1), both achieving full conversion after 2 h (S/C = 1000) or 4 h (S/C = 2000). **C5** displayed marginally better stereoselectivity, but both catalysts were considered in further studies. Apart from excellent diastereo- and enantioselectivity, the catalysts displayed an outstanding turnover frequency for the double reduction of **1a**. As a comparison, the **C5**-catalyzed reduction of acetophenone (S/C = 1000, 60 °C) reached full conversion only after 3 h.^5^ And the **C7**-catalyzed stepwise double reduction of the homologous 1-phenyl-3-CF_3_-propan-1,3-dione (S/C = 1000, 60 °C) was finished in 20 h.^13^

**Table 1:**
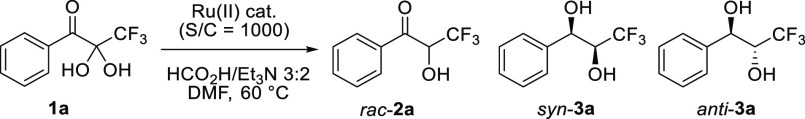
Catalyst Screening for Ru(II)-Catalyzed DKR-ATH of **1a**^a^

	Ru-cat.	Time	**1a**:**2a**:**3a**	*syn*-**3a**/*anti*-**3a**	ee (*syn*)
1	(*R*,*R*)-**C1**	2	4:60:36	98:2	
		18	0:41:59	95:5	–91.3
2	(*S*,*S*)-**C2**	2	0:44:56	87:13	
		18	0:13:87	87:13	97.7
3	(*S*,*S*)-**C3**	2	0:1:99	96:4	
		18	0:1:99	96:4	99.1
4	(*S*,*S*)-**C4**	1	0:25:75	96:4	
		2	0:0:100	96:4	99.5
5	(*S*,*S*)-**C5**	1	0:17:83	97:3	
		2	0:0:100	97:3	99.8
6	(*R*,*R*)-**C6**	2	0:18:82	98:2	
		18	0:7:93	98:2	–97.3
7	(*S*,*S*)-**C7**	1	0:19:81	96:4	
		2	0:0:100	96:4	98.5
8	(3*R*,1′*S*)-**C8**	1	0:0:100	64:36	–74.9

a DKR-ATH of **1a** (110 mg, 0.5 mmol) was carried out using the active hydride Ru(II) cat. (S/C = 1000, 0.5 μmol) prepared in situ from the corresponding monomeric (**C1**–**C6**) or μ-Cl dimeric (**C7**, **C8**) chloride precatalysts in HCO_2_H/Et_3_N (0.5 mL); with DMF (1 mL) as a cosolvent. **1a**:**2a**:**3a** and *syn*-**3a**/*anti*-**3a** ratios were determined by ^1^H and ^19^F NMR analysis of reaction mixture aliquots, and ee of *syn*-**3a** by GC analysis using chiral stationary phase after extraction. For additional results, see Table S1.

We hypothesized that the efficient double reduction of **1a** is driven by a favorable substrate–catalyst interaction involving hydrogen bonding between the OH group of the intermediate *rac*-**2a** and the SO_2_ group of the catalyst. Indeed, a kinetic study (Table S2) revealed that during the double reduction of diketone **1a**, catalyzed by **C4** at 40 °C, the first reduction is rate-determining; i.e., the intermediate monoalcohol **2a** is reduced preferentially in the presence of **1a**. To prove that dynamic kinetic resolution (DKR) of the intermediate stereochemically labile **2a** is involved in the reduction process, the racemic **2a** was subjected to transfer hydrogenation, and its reduction gave similar stereoselectivity to that of the reduction of **1a** (SI, page S6). The enantiomeric ratio of **2a** was essentially 1:1 during the course of the reaction starting from either **1a** or **2a** which points to its fast epimerization in the reaction medium. Rather surprisingly, the monoreduction of **1a** to **2a** is a noncatalyzed and reversible process: stirring **1a** or **2a** in the reaction medium without the catalyst at 60 °C resulted in a mixture of **1a** and **2a** in 5:95 and 1:99 ratio, respectively (see SI page S5). We computed a barrier of 18.0 kcal mol^–1^ for the HCO_2_H catalyzed dehydration of hydrate (geminal diol) **1a** to diketone **1a′**. Its further reduction to **2a** by HCO_2_H without a catalyst was shown by DFT calculation to be strongly exothermic and entropically favored because of the formation of CO_2_ (Δ*G* = −29.8 kcal mol^–1^). This makes the overall dehydration of **1a** and its reduction to **2a** favorable (Δ*G* = −19.5 kcal mol^–1^). The **1a**–**2a** redox equilibrium and keto–enol equilibrium^21^ of **2a** contribute to DKR-enabling fast epimerization of **2a** in the reaction medium. The enol form of **2a** is only 4.8 kcal mol^–1^ less stable than the keto form when hydrogen bonding to HCO_2_H and Et_3_N is considered. The keto–enol tautomerization, where the hydrogen atom is transferred between the sp^3^-C atom and the adjacent carbonyl group, is kinetically inaccessible due to a high activation barrier of 57.6 kcal mol^–1^. However, the barrier is lowered to 18.2 kcal mol^–1^ when the reaction is catalyzed by HCO_2_H/Et_3_N. This guarantees a quick conversion between (*R*)-**2a** and (*S*)-**2a**.

Next, we wanted to prove the origin of enantioselectivity and reaction kinetics during DKR-ATH of *rac*-**2a**, catalyzed by the catalyst (*S*,*S*)-**C4**, by elucidating the structures of plausible transition state (TS) geometries and ascertaining the energetics of the reactions. In the reaction between *rac***-2a** and the active form of Ru(II) catalyst (*S*,*S*)-**C4**, **Cat–H**_**2**_, four diastereomeric TS can exist, depending on the substrate enantiomer and the side of the attack. Moreover, a different prereaction complex (PrC) corresponds to each TS. Selectivity is thus governed by the stability of both TS *and* PrC. (Figure 2)

**Figure 2:**
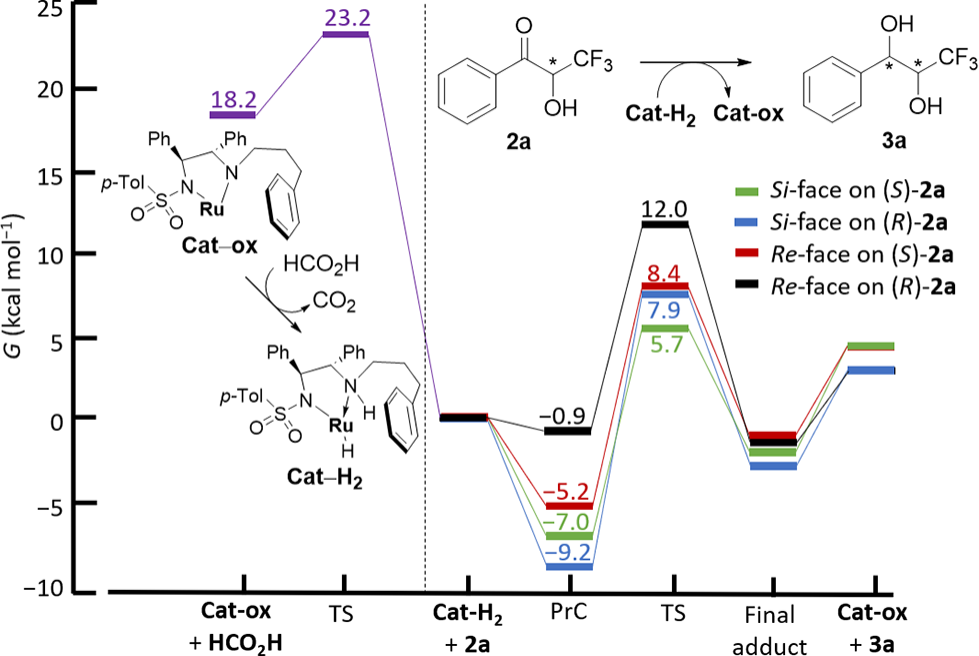
Gibbs free energies for the reaction between *rac***-2a** and the Ru(II) catalyst **C4** active form and for catalyst regeneration with HCO_2_H, at 298.15 K and 1 atm, showing that the *Si*-face attack on the (*S*) enantiomer is most favorable. PrC = prereaction complex; TS = transition state.

First, we analyzed the relative stabilities of individual PrC. Leading up to the (1*R,*2*S*)-**3a**, (1*R,*2*R*)-**3a**, (1*S,*2*S*)-**3a**, and (1*S,*2*R*)-**3a**, their stabilities (calculated as Gibbs free energies relative to infinitely separated catalyst and substrate) are −7.0, −9.2, −5.2, and −0.9 kcal mol^–1^, respectively, at 298.15 K and 1 atm. Hence, the lower stability of their PrC alone can explain why (1*S,*2*R*)-**3a** is detected in traces (below 0.2%) and (1*S,*2*S*)-**3a** forms only in minute quantities (below 1.0%). Only (1*R*,2*S*)-**3a** and (1*R,*2*R*)-**3a** are expected to form in noticeable amounts.

The energies of the TS are 12.7, 17.1, 13.6, and 12.9 kcal mol^–1^ (relative to *each* corresponding PrC) for the formation of (1*R,*2*S*)-**3a**, (1*R,*2*R*)-**3a**, (1*S,*2*S*)-**3a**, and (1*S,*2*R*)-**3a**, respectively, as shown in Figure 2. A large difference in the energy of the TS en route to (*1R,*2*S*)-**3a** and (1*R,*1*R*)-**3a** explains why, despite having a slightly less stable PrC, the former will form much faster, which is consistent with experimentally observed *syn*-selectivity. The product ratio is estimated on the basis of the absolute energies of the TS only, and the following ratio is expected: 96.5:2.3:1.1:0.003 at 298.15 K (Figure 3), which is in reasonable agreement with the experimental data. The overall catalytic cycle for the reduction of both enantiomers of **2a** to respective stereoisomers of **3a** is energetically favored, as shown in Figure 2, due to the hydrogen transfer from HCO_2_H to the oxidized form of the catalyst. This hydrogen transfer is a quick step with a low barrier of 5 kcal mol^–1^ and a downhill Gibbs free energy (−18.2 kcal mol^–1^), because both contributions are favorable: the energy change is negative and the entropy is increased due to the formation of CO_2_. The overall catalytic cycle of (*S*)-**2a** + HCO_2_H → (1*R,*2*S*)-**3a** + CO_2_ is thus energetically downhill (−13.3 kcal mol^–1^).

**Figure 3:**
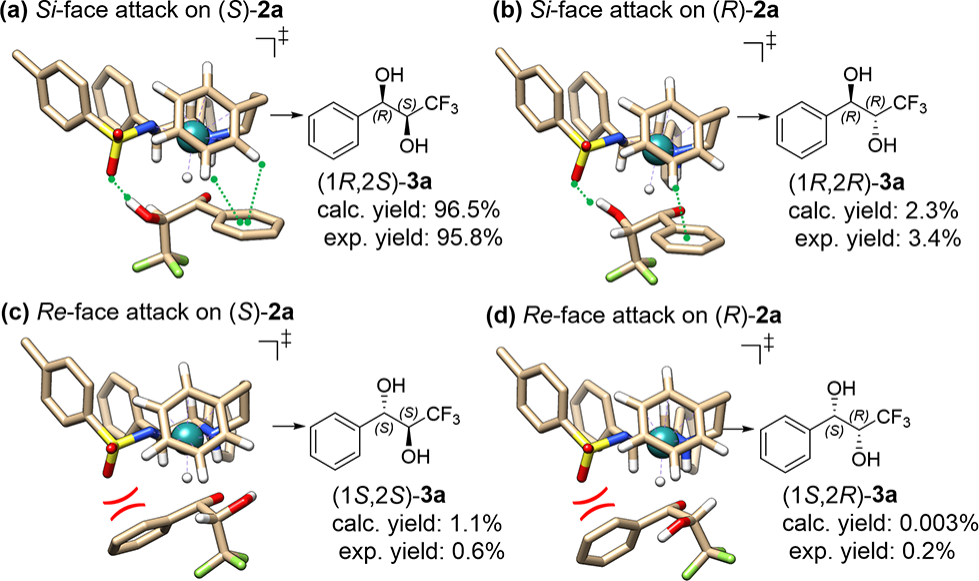
Optimized transition state (TS) geometries leading to the four stereomeric products of **3a** via (a) *Si*-face attack of (*S*,*S*)-**C4** active form on (*S*)-**2a**, (b) *Si*-face attack on (*R*)-**2a**, (c) *Re*-face attack on (*S*)-**2a**, and (d) *Re*-face attack on (*R*)-**2a**. The enantioselectivity determining attractive and repulsive interactions are highlighted by green and red symbols, respectively. Nonessential H atoms are omitted for clarity. Calculated yields are estimated from the relative Gibbs free energies of the TS at 298.15 K and 1 atm.

The stabilization of the particular TS leading to formation of the major stereoisomer (1*R,*2*S*)-**3a**, is brought about by an attractive hydrogen bonding (1.92 Å) interaction between the catalyst’s SO_2_ region and ketone’s α-substituent (O=S=O···H–O–CH), *and* CH/π interaction between the aromatic rings of the catalyst η^6^-arene ligand and substrate (Figure 3a). When (*R*)-**2a** is attacked from the *Si-*face (Figure 3b), the hydrogen bond was confirmed as well (1.75 Å), but the CH/π interaction appears to be weaker due to orientation of the aromatic rings. During the *Re*-face attack of the catalyst (*S*,*S*)-**C4** to either enantiomer of **2a** (Figure 3c and 3d), no hydrogen bond can form, and there is even an SO/π repulsive interaction, making both products disfavored. Rather unexpectedly, the CF_3_ group does not stabilize the *Re*-face attack via an attractive interaction with η^6^-arene. Note that ketones with electron-donating α-substituents studied herein engage in hydrogen bonding with the catalyst via heteroatom-bound β-hydrogen of the ketone, whereas the ketones with electron-withdrawing α-substituents were shown computationally to form an attractive interaction with the SO_2_ group of the Noyori–Ikariya-type ruthenium^21^ and the related rhodium^43,44^ catalysts via the acidic ketone’s α-hydrogen.

With the optimal conditions in hand, we explored the substrate scope.^45^ Because the double reduction of **1a** to **3a** involved DKR of the intermediate racemic **2a**, diketones, as well as acyloins, would be appropriate starting materials. The required CF_3_-substituted 1,2-diketones **1b**, **1e**, **1f**, **1h**–**1l**, and **1n** were prepared from the corresponding (het)aryl aldehydes, similarly to **1a**.^41^ The racemic monoalcohols **2c**, **2d**, **2g**, **2m**, and **2o**–**2q** were prepared via acylation-rearrangement of the corresponding α-hydroxy- or α-amino acids with trifluoroacetic anhydride,^46^ and **2r** was prepared via *N*-heterocyclic carbene catalyzed crossed acyloin condensation.^47^ The two best performing catalysts, **C4** and **C5** were used for DKR-ATH of the substrates. For comprehensive results, see Table S3 in the SI, and Table 2 contains the optimal conditions for each substrate in terms of diastereo- and enantioselectivity. The reaction gives good stereoselectivities for the reduction of aryl ketones with electron acceptor or donor substituents. For the latter, higher catalyst loading was required to achieve full conversion. During the reduction of the formyl-substituted **1l**, the aldehyde group was reduced as well, and the corresponding triol **3l** was isolated. Heterocyclic examples **2m** and **1n** were reduced to the corresponding *syn*-diols with excellent stereoselectivity. DKR-ATH of the α,β-unsaturated ketone **2r** under our standard reaction conditions afforded the major product **3r** with a preserved double bond, along with 16% of the saturated side products. The absolute configuration of the (het)aryl-substituted diols **3a**–**3n** was assigned on the basis of the single-crystal X-ray diffraction (SCXRD) analysis of **3e**. Remarkably, the dialkylketones **2o**–**2q** were reduced to the corresponding *syn*-diols with >95% ee using **C4** or **C5**; the first one was more diastereoselective for the synthesis of the benzyl substituted **3o**, and **C5** was optimal for the synthesis of **3p** and **3q**. In the case of dialkylketone reduction, the stereoselectivity could (in the absence of appropriately positioned aromatic ring on the substrate) rely either on hydrogen bonding of CF_3_CH(OH) moiety to SO_2_ of the catalyst or on CF_3_–η^6^-arene attractive interaction.^13,48^ The two hydrogenation modes would deliver the opposite enantioselectivity, which is often the case for the Noyori–Ikariya reduction of dialkyl vs alkyl aryl ketones.^49−51^ Based on the SCXRD analysis of **3o**, the order of elution in chiral chromatographic analysis, and computational analysis of the transition states during the reduction of the model substrate, where no CF_3_–η^6^-arene attractive interaction has been identified during *Re*-face attack (Figure 3c and 3d), we assigned to the alkyl-substituted diols **3o**, **3p**, and **3q** the same absolute configuration as to the (het)aryl-substituted **3a**–**3n**.

**Table 2:**
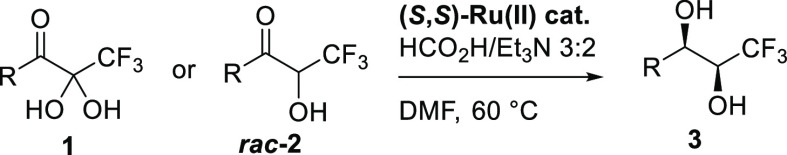

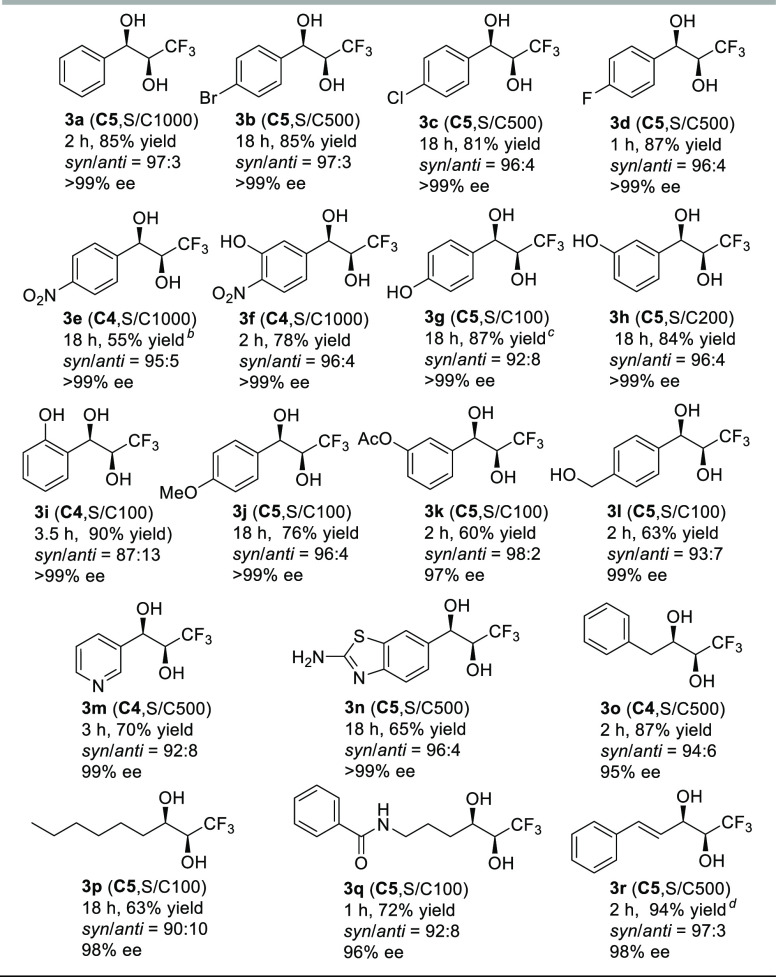
Scope of the CF_3_-Subsituted 1,2-Diols *syn*-**3** Available via Ru(II)-Catalyzed DKR-ATH^a^

a The reactions were carried out using **1** or **2** (0.5 mmol) and (*S*,*S*)-diphenylethylenediamine-based ruthenium catalyst in HCO_2_H/Et_3_N 3:2 (0.5 mL) and DMF (1 mL) at 60 °C.

b Isolated yield of stereopure *syn*-**3e** on a gram-scale.

c 88% conversion.

d 16% of double bond reduction products.

Racemic diols **3** to be used as standards for the determination of stereoisomeric ratios were prepared by NaBH_4_ reduction in ethanol. It is noteworthy that using this approach, the alternative diastereomers *anti*-**3** were the major products (*anti*/*syn* ratios between 65:35 and 97:3); for details, see Table S3.

To demonstrate synthetic usefulness of the developed method, the *p*-nitro derivative **1e** was reduced on a gram scale using a S/C = 1000. The diol **3e** was acetonide-protected, and then the nitro group was reduced to the corresponding amine building block **4** using iron in acetic acid. After straightforward coupling to 5-chloroindole-2-carboxylic acid and deprotection, compound **5** was prepared. Compound **5** is a glycogen phosphorylase inhibitor, previously reported as racemic *syn* diastereomer (Scheme 1a).^33^ Furthermore, as a support to the hit-to-lead optimization campaign of our in-house antibacterial topoisomerase inhibitor ULD1,^52,53^ its analogues with carboxylic acid-to-CF_3_-diol replacement were prepared.^35^ The stereochemical array of compounds **6** was accessed from the diketone **1n** by employing respective enantiomers of **C5** to access homochiral *syn*-**6**, or NaBH_4_ reduction to target the racemic *anti*-**6** (Scheme 1b).

**Scheme 1:**
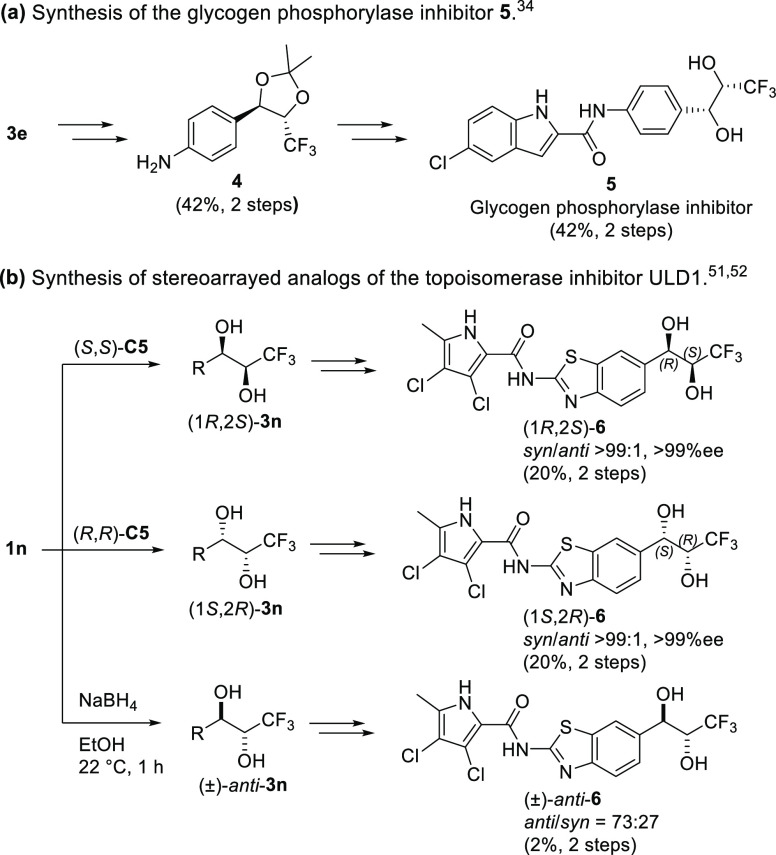
Further Synthetic Transformations of Stereopure DKR-ATH Products **3**

In conclusion, we have successfully developed *syn*-selective reductive access to homochiral CF_3_-substituted-1,2-diols by employing dynamic kinetic resolution during the Noyori–Ikariya asymmetric transfer hydrogenation of the corresponding α-hydroxyketones. The origin of stereoselectivity was investigated by using DFT calculations and was attributed to the hydrogen bonding between the ketone’s H-bond donor α-substituent and the catalyst’s SO_2_ moiety. High stereoselectivity (*syn*/*anti* ≥87:13, ≥95% ee) was achieved for the reduction of (het)aryl, vinyl, benzyl, and alkyl ketones. Furthermore, elaboration of the stereopure diols to molecules relevant for medicinal chemistry was demonstrated.

## Supporting Information Available

X-ray data for **3e** (CIF)
X-ray data for **3o** (CIF)
Additional results, experimental procedures, chiral HPLC and GC chromatograms, NMR spectra of the prepared compounds, SCXRD details, theoretical calculation details and geometries (PDF)

## Supplementary Material

cs3c00980_si_001.cif

cs3c00980_si_002.cif
