# Supplementary material for: Hydrogen-Bonding Ability of Noyori–Ikariya Catalysts Enables Stereoselective Access to CF3-Substituted syn-1,2-Diols via Dynamic Kinetic Resolution
Source: ACS Catal. 2023 Apr 21;13(9):6242–8. doi: 10.1021/acscatal.3c00980 (PMC10167654; doi:10.1021/acscatal.3c00980)
Supplement: Supplementary file 3 — cs3c00980_si_003.pdf [file cs3c00980_si_003.pdf]

Hydrogen-bonding ability of Noyori–Ikariya catalysts enables stereoselective access to CF_3-substituted *syn*-1,2-diols via dynamic kinetic resolution

Maša Sterle,† Matej Huš,§,#,δ Matic Lozinšek,‡ Anamarija Zega,† Andrej Emanuel Cotman*†

† Faculty of Pharmacy, University of Ljubljana, Aškerčeva cesta 7, SI-1000 Ljubljana, Slovenia

§ National Institute of Chemistry, Department of Catalysis and Chemical Reaction Engineering, Hajdrihova ulica 19, SI-1000 Ljubljana, Slovenia

Association for Technical Culture of Slovenia, Zaloška cesta 65, SI-1000 Ljubljana, Slovenia

δ Institute for the Protection of Cultural Heritage of Slovenia, Poljanska 40, SI-1000 Ljubljana, Slovenia

‡ Jožef Stefan Institute, Jamova cesta 39, SI-1000 Ljubljana, Slovenia

Supporting Information

Contents

1 Materials and instrumentation 2

2 Additional experimental results 3

2.1 Table S1. Additional results on catalyst screening for Ru(II)-catalyzed DKR-ATH of **1a**. 3

2.2 Reaction kinetics 4

2.3 Control experiments 5

2.4 Table S3. Reduction of diketones **1** or α-hydroxyketones **2** using $NaBH_4$ or DKR-ATH employing catalysts **C4** and **C5**. 9

3 Synthesis of DKR-ATH substrates **1a**, **1b**, **1e**, **1f**, **1h**–**1l** and **2c**, **2d**, **2g**, **2m**, **2o**–**2r** 11

4 Synthesis of *syn*- and *anti*-CF_3-substituted-1,2-diols **3a**–**3r** 18

5 Further synthetic transformations of the diols **3e** and **3n** 26

6 Determination of stereomeric ratios 29

6.1 Determination of diastereomeric ratios by ^{19}F NMR 29

6.2 Determination of enantiomeric ratios by chiral GC and HPLC chromatography 47

7 NMR spectra 88

8 Single-crystal X-ray difraction 191

9 Theoretical calculations 1966

10 References 2255

1 Materials and instrumentation

General. Reactions were conducted under an inert atmosphere using anhydrous solvents when required. For reactions that require heating, an oil bath was used as the heat source. Analytical thin layer chromatography (TLC) was performed on Silica Gel $60F_{254}$ plates. Flash column chromatography was performed using Silica Gel 60 (40–63 μm). The enantiomeric excess (ee) of reduced products was determined by normal phase HPLC analysis on Agilent Technologies 1100 instrument with G1365B UV–vis detector, G1316A thermostat, and G1313A autosampler or Waters 2695 separations module, equipped with Waters 2996 Photodiode Array Detector, using Chiralpak IA column (25 cm) or Chiralpak IB-3 (25 cm) column as specified; or GC analysis on Shimadzu GC-2010 gas chromatograph, equipped with AOC-20i auto injector, helium as a carrier gas, and flame ionisation detector, using CP-ChiraSil-DEX CB column (25 m x 0.25 cm). ^{1}H NMR (400 MHz; internal Me_4Si = 0 ppm), ^{13}C NMR (100 MHz; internal Chloroform-*d* = 77.16 ppm, DMSO-d_6 = 39.52 ppm, Methanol-d_4 = 49.00 ppm, Acetone-d_6 = 29.84 ppm), and ^{19}F NMR (380 MHz, external CCl_3F = 0 ppm) spectra were recorded on a Bruker AVANCE III 400 spectrometer (Bruker Corporation, Billerica, MA, USA). HRMS were obtained using Exactive Plus Orbitrap mass spectrometer (Thermo Fisher Scientific, Waltham, MA, USA). The catalysts were weighted on Metler Toledo XPR2 balance (± 0.5 µg) and the other reagents on Metler Toledo XSR205 balance (± 10 µg).

Catalysts. (*R*,*R*)-**C1** and (*S*,*S*)-**C5** were purchased from TCI (Tokyo, Japan); (*S*,*S*)-**C2** was purchased from abcr GmbH (Karlsruhe, Germany); (*S*,*S*)-**C3** and (*S*,S)-**C4** were purchased from Sigma-Aldrich (St. Louis, MO, USA) as ruthenium chloride monomers; (*R*,*R*)-**C6** was kindly donated by Prof Martin Wills from the University of Warwick, UK; (*S*,*S*)-**C7** and (3*R*, 1'*S*)-**C8** were prepared according to the literature procedures as *μ*-(ruthenium dichloride) dimers.[1,2] All catalysts were activated by stirring in HCO_2H/Et_3N at 22 °C for 30 min under a light stream of argon.

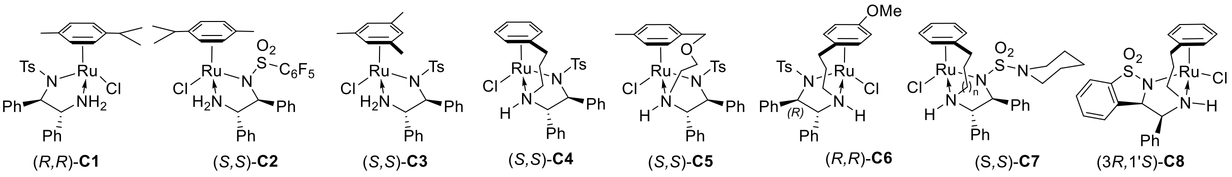

Reagents. HCO_2H/Et_3N 5:2 was prepared by adding Et_3N (280 mL, 2 mol) to HCO_2H (189 mL, 5 mol) at 0 °C under nitrogen atmosphere and used as such. It was stored at room temperature without any precautions regarding air and moisture. Analogously, HCO_2H/Et_3N 3:2 was prepared by adding Et_3N (280 mL, 2 mol) to HCO_2H (113 mL, 3 mol). The molar ratios were confirmed by ^{1}H NMR analysis (relaxation delay = 25 s).

2 Additional experimental results

2.1 *Table S1. Additional results on catalyst screening for Ru(II)-catalyzed DKR-ATH of* ***1a***.[a]

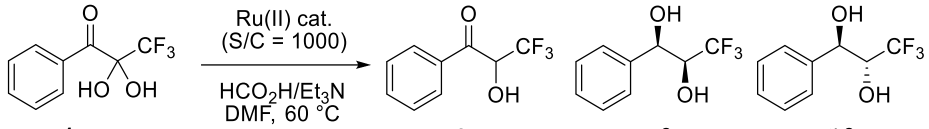

1a *rac*-**2a** *syn*-**3a** *anti*-**3a**

	Ru(II) cat.	HCO_2H/Et_3N	Time [h]	1a:2a:3a	*syn*-3a/ *anti*-3a	ee (*syn*)
1	(*R*,*R*)-**C1**	3:2	1	2:65:33	99:1	-
			2	4:60:36	98:2	-
			18	0:41:59	95:5	−91.3
2	(*S*,*S*)-**C2**	3:2	1	0:65:35	88:12	-
			2	0:44:56	87:13	-
			18	0:13:87	87:13	97.7
3	(*S*,*S*)-**C3**	3:2	1	0:5:95	96:4	-
			2	0:1:99	96:4	-
			18	0:1:99/	96:4	99.1
4	(*S*,*S*)-**C4**	3:2	1	0:25:75	96:4	-
			2	0:0:100	95:5	-
			18	0:0:100	96:4	99.5
5	(*S*,*S*)-**C5**	3:2	1	0:17:83	98:2	-
			2	0:0:100	97:3	-
			18	0:0:100	97:3	99.8
6	(*R*,*R*)-**C6**	3:2	1	0:25:75	99:1	-
			2	0:18:82	98:2	-
			18	0:7:93	98:2	−97.3
7	(*S*,*S*)-**C7**	3:2	1	0:19:81	96:4	-
			2	0:0:100	96:4	
			18	0:0:100	96:4	98.5
8	(3*R*,1'*S*)-**C8**	3:2	1	0:0:100	64:36	-
			2	0:0:100	64:36	-
			18	0:0:100	64:36	−74.9
9	(*S*,*S*)-**C4**	3.4:2	2	0:0:100	95:5	n.d.
10	(*S*,*S*)-**C4**	5:2	2	0:0:100	93:7	>99
11	(*S*,*S*)-**C4**[b]	3:2	4	0:0:100	96:4	>99
12	(*S*,*S*)-**C5**[b]	3:2	4	0:0:100	98:2	>99
13	(*S*,*S*)-**C5**[c]	3:2	1	49:24:27	-	-
			2	24:18:58	-	-
			18	1:2:97	97:3	>99.9

*[a]DKR-ATH of 1a (110 mg, 0.5 mmol) was carried out at 60 °C using the active hydride Ru(II) cat. (S/C = 1000, 0.5 μmol) prepared in situ from the corresponding monomeric (**C1–C6**) or μ-Cl dimeric (**C7, C8**) chloride precatalysts in HCO_2H/Et_3N (0.5 mL); with DMF (1 mL) as a cosolvent. **1a**:**2a**:**3a** and syn-**3a**/anti-**3a** ratio were determined by ^1H and ^{19}F NMR, and ee of syn-**3a** by GC analysis using chiral stationary phase. [b]S/C = 2000. [c]40 °C.*

2.2 Reaction kinetics

The ruthenium catalyst (*S*,*S*)-**C4** (310 µg, 0.5 µmol) was treated with HCO_2H/Et_3N 3:2 (0.5 mL) and stirred at r.t. for 30 min under a light stream of argon. A solution of diketone **1a** (110 mg, 0.5 mmol) in DMF (1 mL) was then added and the resulting homogenous mixture was stirred at 40 °C with continued argon sweeping. Reaction mixture aliquots (20 µL) were sampled at regular intervals (**Table S2**) and partitioned between ethyl acetate (0.5 mL) and water (0.5 mL) in order to quench the reaction. The organic layer was concentrated and analysed by ^{19}F NMR to determine the ratio between **1a**, **2a**, *syn*-**3a** and *anti*-**3a**. ^{19}F NMR (376 MHz, Chloroform-*d*) δ −73.82 (d, *J* = 6.3 Hz, **2a**), −74.82 (d, *J* = 6.7 Hz, *anti*-**3a**), −76.81 (d, *J* = 6.9 Hz, *syn*-**3a**), −81.14 (s, **1a**).

Table S2. Kinetics of (*S*,*S*)-**C4** catalyzed reduction of **1a**.

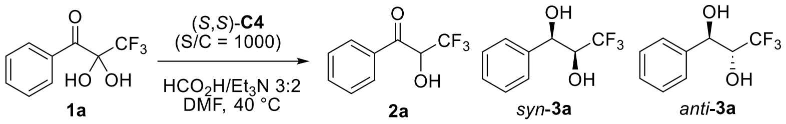

time [min]	**2a** [%]	*syn*-**3a** [%]	**1a** [%]	*anti*-**3a** [%]
5	4.91	0.65	94.43	0
10	5.37	2.61	92.01	0
15	8.05	5.04	86.01	0
20	10.58	8.7	80.72	0
25	1	16.89	81.47	0.64
30	3.01	28.26	68.08	0.65
35	3.04	36.96	58.59	1.05
40	1.74	42.21	54.63	1.42
45	9.27	29.31	60.62	0.8
50	9.72	32.04	57.34	0.9
55	14.05	31.14	54.1	0.71
60	5.62	42.9	50.13	1.36
70	3.47	57.02	37.28	2.23
80	3.24	62.45	31.73	2.5
90	2.47	64.55	29.83	3.15
100	2.61	63.78	30.31	2.7
110	10.4	64.5	30.91	2.65
120	9	66	27.28	2.7

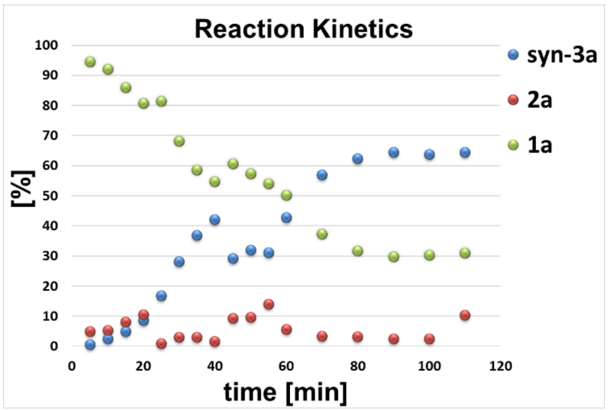

2.3 Control experiments

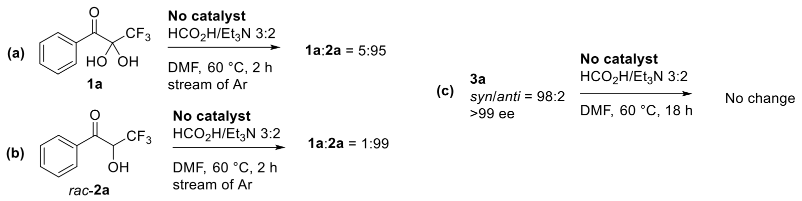

* The reactions without catalyst were performed using glassware and magnetic stirring bars washed with aqua regia.

Control experiment (a). A solution of **1a** (55 mg, 0.25 mmol) in DMF (0.5 mL) and HCO_2H/Et_3N 3:2 (0.25 mL) was stirred at 60 °C under a light stream of argon. An aliquot of the reaction mixture was analyzed by ^{19}F NMR after 1 h to reveal noncatalyzed reduction of **1a** to **2a**, **1a**:**2a** = 15:85. And after 2 h, NMR analysis of a reaction mixture aliquot revealed **1a**:**2a** = 5:95.

Control experiment (b). A solution of **2a** (51 mg, 0.25 mmol) in DMF (0.5 mL) and HCO_2H/Et_3N 3:2 (0.25 mL) was stirred at 60 °C under a light stream of argon. An aliquot of the reaction mixture was analyzed by ^{19}F NMR after 1 h to reveal reversible conversion of **2a** to **1a**, **1a**:**2a** = 0.5:99.5. And after 2 h, NMR analysis of a reaction mixture aliquot revealed **1a**:**2a** = 1:99.

Control experiment (c). A solution of **3a**, *syn/anti* = 97:3, (52 mg, 0.25 mmol) in DMF (0.5 mL) and HCO_2H/Et_3N 3:2 (0.25 mL) was stirred at 60 °C under a light stream of argon. An aliquot of the reaction mixture was analyzed by ^1H and ^{19}F NMR after 1 h, 2 h and 18 h. No decomposition or change in diastereomeric ratio of **3a** was observed.

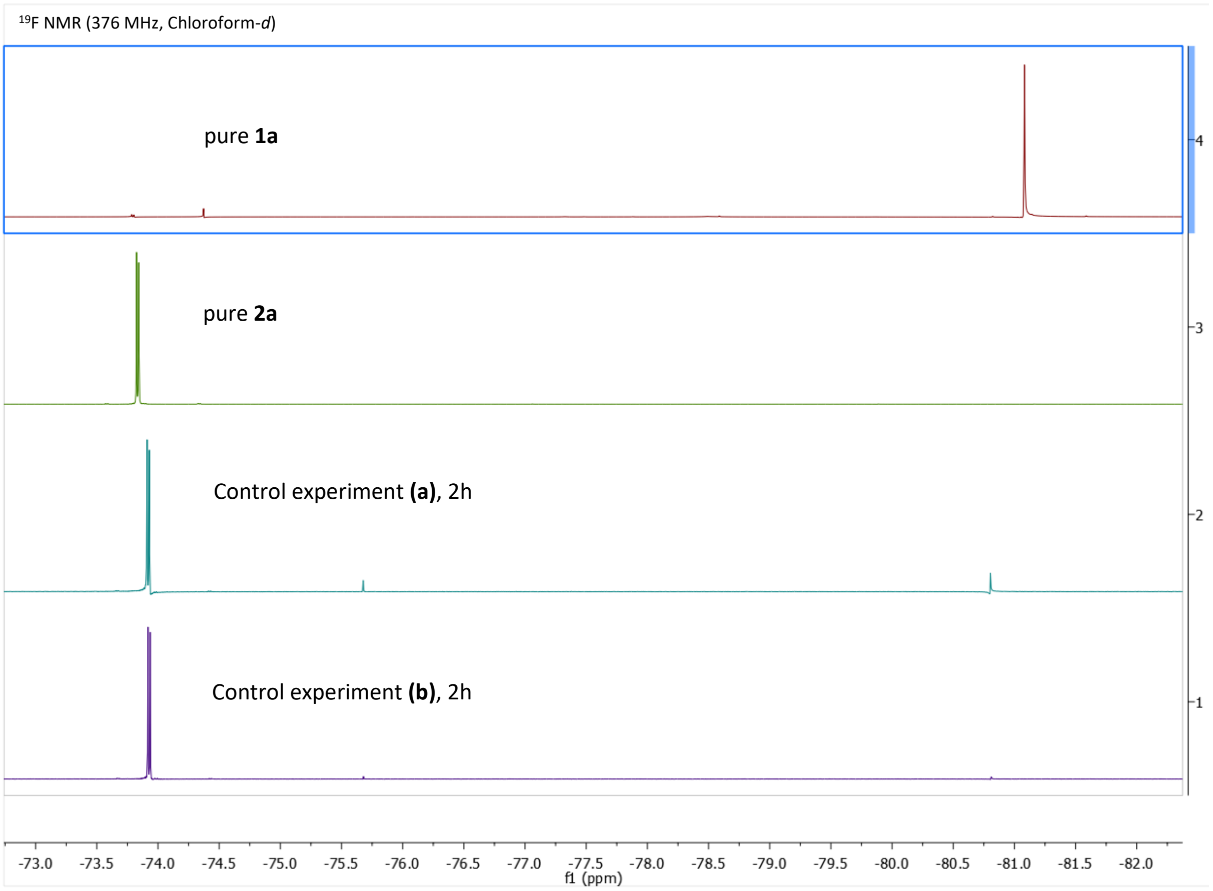

(d) 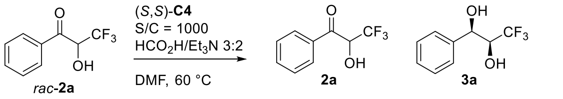

	2a	3a
30 min	81% **2a**<br>1% ee	19% **3a**<br>*syn*/*anti* = 97:3
1 h	63% **2a**<br>2% ee	37% **3a**<br>*syn*/*anti* = 97:3
2 h	5% **2a**<br>5% ee	95% **3a** (>99% ee)<br>*syn*/*anti* = 96:4

(e) 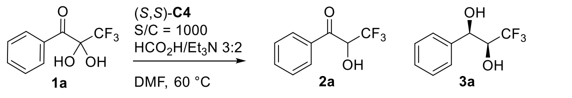

	2a	3a
30 min	43% **2a**<br>2% ee	57% **3a**<br>*syn*/*anti* = 96:4
1 h	23% **2a**<br>3% ee	77% **3a**<br>*syn*/*anti* = 96:4
2 h	<1% **2a**<br>11% ee	>99% **3a** (>99% ee)<br>*syn*/*anti* = 96:4

Control experiment (d). A mixture of (*S*,*S*)-**C4** (0.47 mg, S/C = 1000) and HCO_2H/Et_3N 3:2 (0.75 mL) was stirred at 22 °C for 30 min under a light stream of argon. A solution of *rac*-**2a** (153 mg, 0.75 mmol) in DMF (1.5 mL) was added and the reaction mixture was stirred at 60 °C. An aliquot (0.2 mL) of the reaction mixture was sampled after 30 min, 1 h and 2 h and partitioned between EtOAc and water. The organic layer was washed with brine, dried over Na_2SO_4 and concentrated. The **1a**/**2a**/**3a** and *syn*/*anti* ratios were determined by ^{19}F NMR, and ee by HPLC on columns with chiral stationary phase.

Control experiment (e). A mixture of (*S*,*S*)-**C4** (0.47 mg, S/C = 1000) and HCO_2H/Et_3N 3:2 (0.75 mL) was stirred at 22 °C for 30 min under a light stream of argon. A solution of **1a** (165 mg, 0.75 mmol) in DMF (1.5 mL) was added and the reaction mixture was stirred at 60 °C. An aliquot (0.2 mL) of the reaction mixture was sampled after 30 min, 1 h and 2 h and partitioned between EtOAc and water. The organic layer was washed with brine, dried over Na_2SO_4 and concentrated. The **1a**/**2a**/**3a** and *syn*/*anti* ratios were determined by ^{19}F NMR, and ee by HPLC on columns with chiral stationary phase.

^{19}F NMR (376 MHz, Chloroform-*d*) δ −73.83 (d, *J* = 6.4 Hz, **2a**), −74.84 (d, *J* = 6.8 Hz, *anti*-**2a**), −76.84 (d, *J* = 6.8 Hz, *syn*-**2a**).

Enantiomeric excess of **2a** and *syn*-**3a** was determined by HPLC analysis on Chiralpak IA column (25 cm), eluent hexane/2-PrOH 95:5, flow rate 1 mL/min, λ = 254 nm. t_R = 10.8 min (**2a**); 14.9 min (**3a**); 16.0 min (*ent*-**2a**); 16.3 min (*ent*-**3a**). Note that UV absorbance of **3a** relative to **2a** is significantly lower.

rac-**2a**:

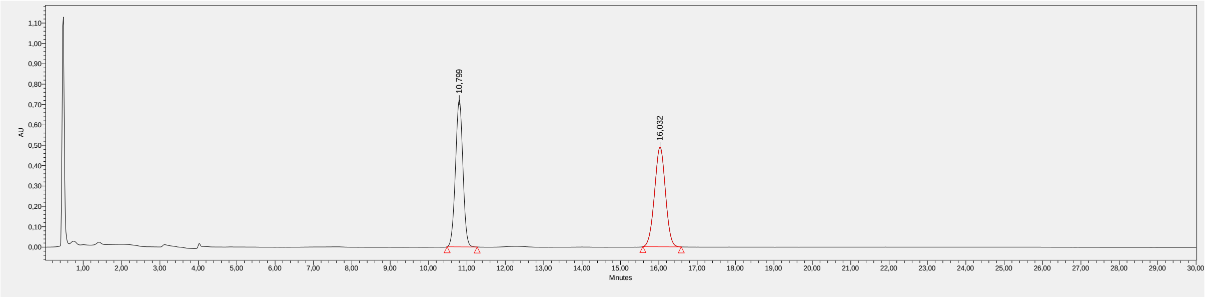

	Name	Retention Time	Area	% Area	Height
1	**2a**	10,799	9671825	50,06	721335
2	*ent*-**2a**	16,032	9648361	49,94	490274

A reference mixture of **3a** and *ent*-**3a** derived from runs with (*S*,*S*)-**C5** and (*R*,*R*)-**C6**, Table 1, entries 5 and 6:

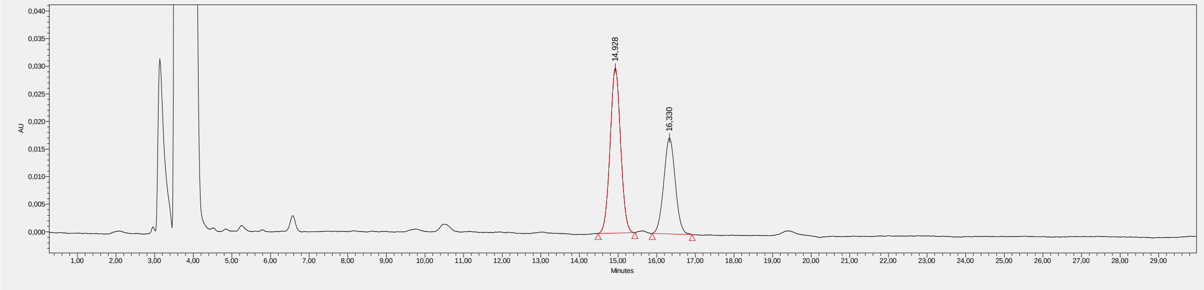

	Name	Retention Time	Area	% Area	Height
1	**3a**	14,928	564390	61,08	29960
2	*ent*-**3a**	16,330	359632	38,92	17382

Control experiment (d), 30 min:

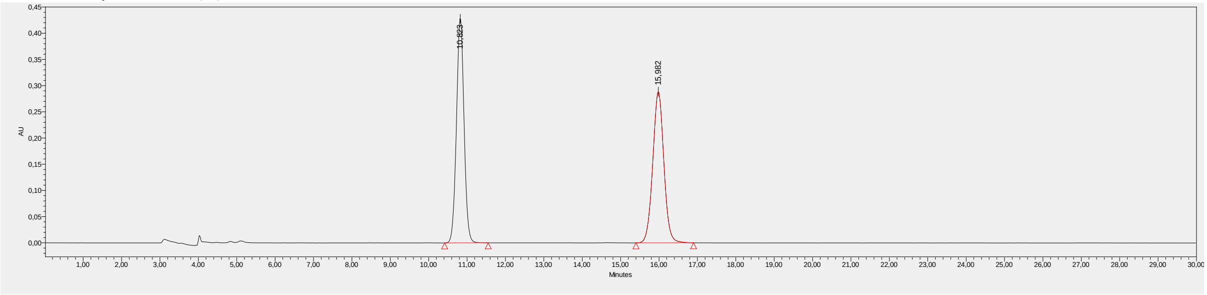

	Name	Retention Time	Area	% Area	Height
1	**2a**	10,823	5886363	50,47	429249
2	*ent*-**2a**	15,982	5776678	49,53	288595

Control experiment (d), 60 min:

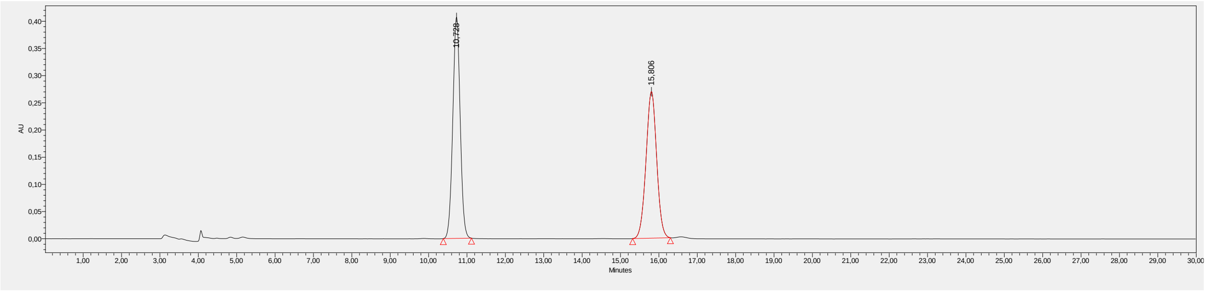

	Name	Retention Time	Area	% Area	Height
1	**2a**	10,728	5444144	51,21	407731
2	*ent*-**2a**	15,806	5186751	48,79	269782

Control experiment (d), 120 min:

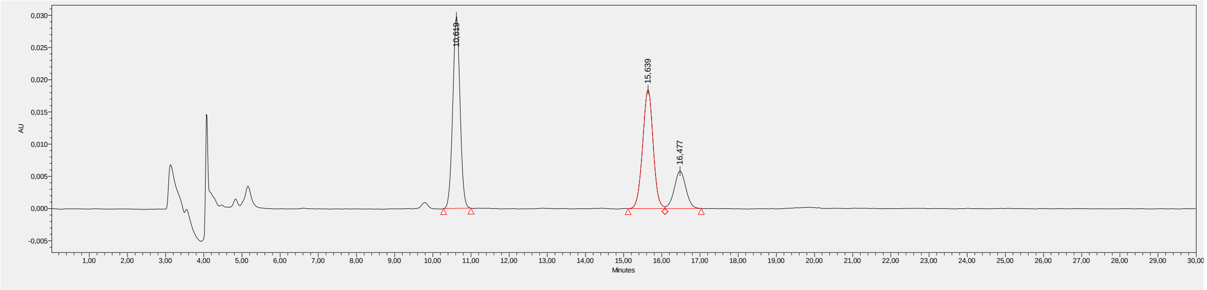

	Name	Retention Time	Area	% Area	Height
1	**2a**	10,619	394208	45,30	29789
2	*ent*-**2a**	15,639	353162	40,59	18473
3	*ent*-**3a**	16,477	122765	14,11	5823

Control experiment (e), 30 min:

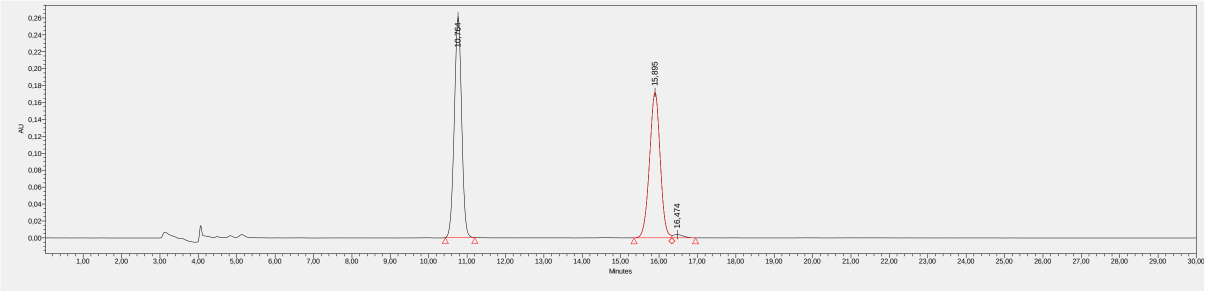

	Name	Retention Time	Area	% Area	Height
1	**2a**	10,764	3452806	50,54	261141
2	*ent*-**2a**	15,895	3307918	48,42	172037
3	*ent*-**3a**	16,474	71115	1,04	3768

Control experiment (e), 60 min:

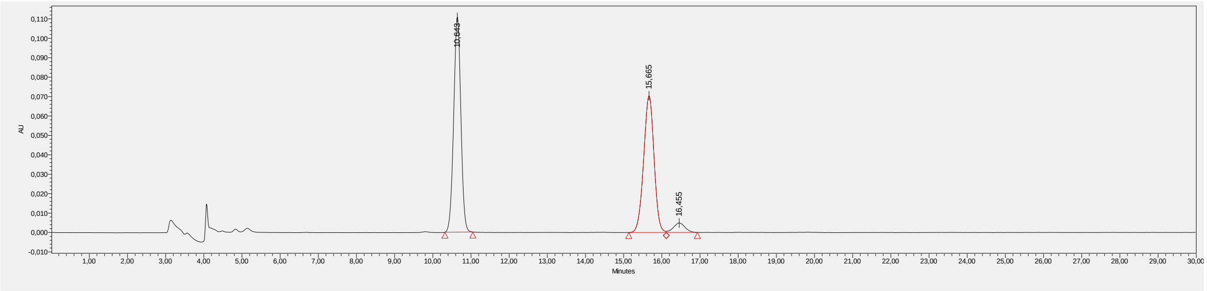

	Name	Retention Time	Area	% Area	Height
1	**2a**	10,643	1446139	50,24	110980
2	*ent*-**2a**	15,665	1329939	46,21	70456
3	*ent*-**3a**	16,455	102099	3,55	4884

Control experiment (e), 120 min:

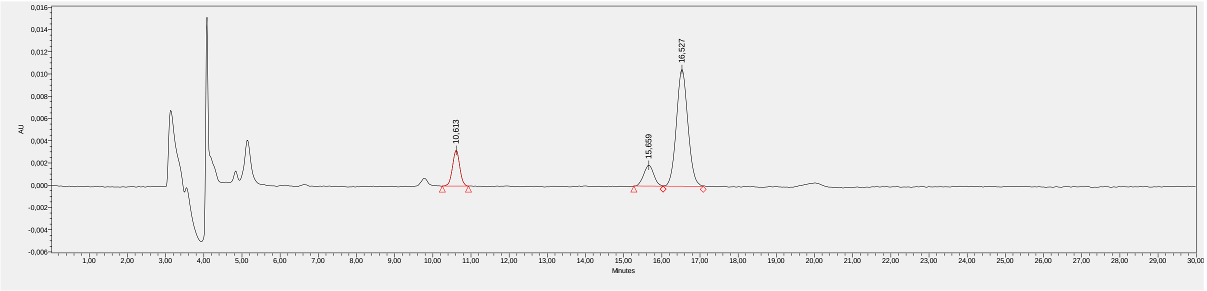

	Name	Retention Time	Area	% Area	Height
1	**2a**	10,613	42648	14,35	3225
2	*ent*-**2a**	15,659	34597	11,64	1880
3	*ent*-**3a**	16,527	219864	74,00	10510

2.4 *Table S3. Reduction of diketones **1** or α-hydroxyketones **2** using $NaBH_4$ or DKR-ATH employing catalysts **C4** and **C5**.*

		**$NaBH_4$** reduction	*(S,S)*-**C4**	*(S,S)*-**C5**
**1a**	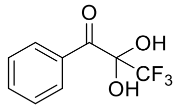	(±)-*anti*-**3a**<br>*anti/syn* = 97:3	(1*R*,2*S*)-*syn*-**3a**<br>S/C = 1000<br>*syn/anti* = 96:4<br>99% ee	(1*R*,2*S*)-*syn*-**3a**<br>S/C = 1000<br>*syn/anti* = 97:3<br>>99% ee
**1b**	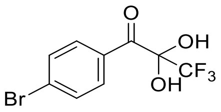	(±)-*anti*-**3b**<br>*anti/syn* = 86:14	(1*R*,2*S*)-*syn*-**3b**<br>S/C = 1000<br>*syn/anti* = 96:4<br>>99% ee	(1*R*,2*S*)-*syn*-**3b**<br>S/C = 500<br>*syn/anti* = 97:3<br>>99% ee
**2c**	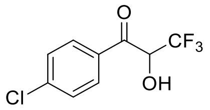	(±)-*anti*-**3c**<br>dr = 89:11	(1*R*,2*S*)-*syn*-**3c**<br>S/C = 500<br>*syn/anti* = 95:5<br>97% ee	(1*R*,2*S*)-*syn*-**3c**<br>S/C = 500<br>*syn/anti* = 96:4<br>>99% ee
**2d**	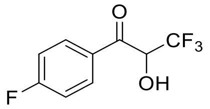	(±)-*anti*-**3d**<br>*anti/syn* = 91:9	(1*R*,2*S*)-*syn*-**3d**<br>S/C = 500<br>*syn/anti* = 95:5<br>98% ee	**3d** (1*R*,2*S*)-*syn*<br>S/C = 500<br>*syn/anti* = 96:4<br>>99% ee
**1e**	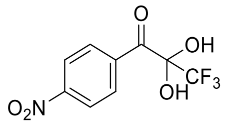	(±)-*anti*-**3e**<br>*anti/syn* = 67:33	(1*R*,2*S*)-*syn*-**3e**<br>S/C = 1000<br>*syn/anti* = 95:5<br>>99% ee	(1*R*,2*S*)-*syn*-**3e**<br>S/C = 500<br>*syn/anti* = 94:6<br>>99% ee
**1f**	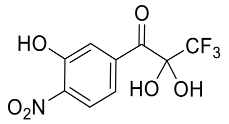	(±)-*anti*-**3f**<br>*anti/syn* = 96:4	(1*R*,2*S*)-*syn*-**3f**<br>S/C = 1000<br>*syn/anti* = 96:4<br>>99% ee	(1*R*,2*S*)-*syn*-**3f**<br>S/C = 100<br>*syn/anti* = 96:4<br>>99% ee
**2g**	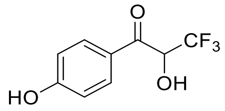	(±)-*anti*-**3g**<br>*anti/syn* = 89:11	(1*R*,2*S*)-*syn*-**3g**<br>S/C = 500<br>*syn/anti* = 84:16<br>≥99% ee	(1*R*,2*S*)-*syn*-**3g**<br>S/C = 100<br>*syn/anti* = 92:8<br>>99% ee<br>88% conversion
**1h**	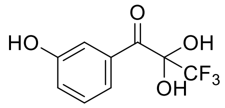	(±)-*anti*-**3h**<br>*anti/syn* = 93:7	(1*R*,2*S*)-*syn*-**3h**<br>S/C = 500<br>*syn/anti* = 93:7<br>>99% ee	(1*R*,2*S*)-*syn*-**3h**<br>S/C = 200<br>*syn/anti* = 96:4<br>>99% ee
**1i**	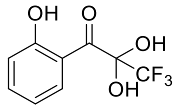	(±)-*anti*-**3i**<br>*anti/syn* = 91:9	(1*R*,2*S*)-*syn*-**3i**<br>S/C = 100<br>*syn/anti* = 87:13<br>>99% ee	(1*R*,2*S*)-*syn*-**3i**<br>S/C = 100<br>*syn/anti* = 69:31<br>>99% ee
**1j**	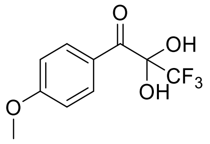	(±)-*anti*-**3j**<br>*anti/syn* = 97:3	(1*R*,2*S*)-*syn*-**3j**<br>S/C = 1000<br>*syn/anti* = 95:5<br>98% ee	(1*R*,2*S*)-*syn*-**3j**<br>S/C = 100<br>*syn/anti* = 96:4<br>>99% ee
**1k**	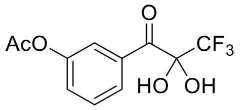	/	(1*R*,2*S*)-*syn*-**3k**<br>S/C = 1000<br>*syn/anti* = 96:4<br>96% ee	(1*R*,2*S*)-*syn*-**3k**<br>S/C = 100<br>*syn/anti* = 98:2<br>97% ee
**1l**	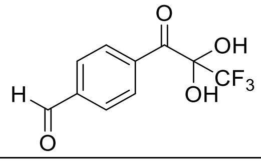	(±)-*anti*-**3l**<br>*anti/syn* = 96:4	(1*R*,2*S*)-*syn*-**3l**<br>S/C = 100<br>*syn/anti* = 75:25<br>>99% ee	(1*R*,2*S*)-*syn*-**3l**<br>S/C = 100<br>*syn/anti* = 93:7<br>>99% ee

		**$NaBH_4$** reduction	*(S,S)*-**C4**	*(S,S)*-**C5**
**2m**	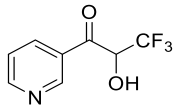	/	(1*R*,2*S*)-*syn*-**3m**<br>S/C = 500<br>*syn/anti* = 92:8<br>99% ee	/
**1n**	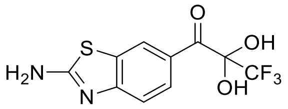	(±)-*anti*-**3n**<br>*anti/syn* = 96:4	(1*R*,2*S*)-*syn*-**3n**<br>S/C = 500<br>*syn/anti* = 91:9<br>>99% ee	(1*R*,2*S*)-*syn*-**3n**<br>S/C = 500<br>*syn/anti* = 96:4<br>>99% ee
**2o**	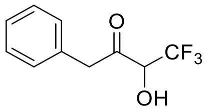	(±)-*anti*-**3o**<br>*anti/syn* = 65:35	(2*S*,3*R*)-*syn*-**3o**<br>S/C = 500<br>*syn/anti* = 94:6<br>95% ee	(2*S*,3*R*)-*syn*-**3o**<br>S/C = 500<br>*syn/anti* = 87:13<br>96% ee
**2p**	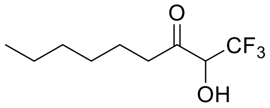	(±)-*anti*-**3p**<br>*anti/syn* = 69:31	(2*S*,3*R*)-*syn*-**3p**<br>S/C = 500<br>*syn/anti* = 77:23<br>96% ee	(2*S*,3*R*)-*syn*-**3p**<br>S/C = 100<br>*syn/anti* = 90:10<br>98% ee
**2q**	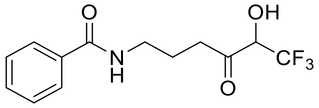	(±)-*anti*-**3q**<br>*anti/syn* = 65:35	(4*R*,5*S*)-*syn*-**3q**<br>S/C = 100<br>*syn/anti* = 79:21<br>72% ee	(4*R*,5*S*)-*syn*-**3q**<br>S/C = 100<br>*syn/anti* = 92:8<br>96% ee
**2r**	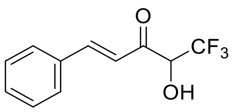	/	(*R*,*R*)-**C4** was used<br>(2*R*,3*S*)-*syn*-**3r**<br>S/C = 500<br>*syn/anti* = 96:4<br>97% ee	(2*S*,3*R*)-*syn*-**3r**<br>S/C = 500<br>*syn/anti* = 97:3<br>98% ee

3 Synthesis of DKR-ATH substrates 1a, 1b, 1e, 1f, 1h–1l and 2c, 2d, 2g, 2m, 2o–2r

2-aminobenzo[*d*]thiazole-6-carbaldehyde.[3] CAS: 106429-08-7. To a solution of the commercially available (2-aminobenzo[*d*]thiazol-6-yl)methanol (10 g, 55.46 mmol) in dry tetrahydrofuran, activated MnO_2 (48.2g, 555 mmol) was added. The resulting mixture was stirred at 22 °C for 24 h, then it was filtered through Celite and concentrated to obtain the title compound as yellow solid (7.1 g, 72% yield). **^{1}H NMR** (400 MHz, DMSO-*d$_6$*) δ 9.87 (s, 1H), 8.23 (d, *J* = 1.5 Hz, 1H), 8.05 (s, 2H), 7.76 (dd, *J* = 8.3, 1.7 Hz, 1H), 7.45 (d, *J* = 8.3 Hz, 1H).

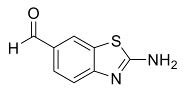

Chemical Formula: $C_8H_6N_2OS$
Molecular Weight: 178,21

2-hydroxy-2-(pyridin-3-yl)acetonitrile.[4] CAS: 17604-74-9. To a solution of nicotinaldehyde (6.3 g, 58.5 mmol) in 2 M HCl (31.5 mL) at −20 °C, 20% aqueous solution of KCN (22.2 mL) was added dropwise. The reaction mixture was stirred at 22 °C for 2 h. Upon completion of the reaction, oily orange product precipitated which was separated from water phase and used directly in the next step (1.1 g, 14% yield). **^{1}H NMR** (400 MHz, DMSO-*d$_6$*) δ 8.69 (d, *J* = 1.8 Hz, 1H), 8.67–8.57 (m, 1H), 7.91 (d, *J* = 7.9 Hz, 1H), 7.50 (dd, *J* = 7.8, 4.8 Hz, 1H), 7.24 (s, 1H), 5.88 (s, 1H).

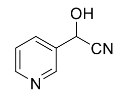

Chemical Formula: $C_7H_6N_2O$
Molecular Weight: 134,14

2-hydroxy-2-(pyridin-3-yl)acetic acid.[4] CAS: 138625-36-2. The solution of the above 2-hydroxy-2-(pyridin-3-yl)acetonitrile (1.083 g, 8.07 mmol) in 37% hydrochloric acid was refluxed for 2 h. Reaction mixture was cooled to 22 °C, the precipitate was filtered off, and the mother liquid was concentraded to get the title compound as oily product (1.6 g, 132%* yield; (*contains residual NH_4Cl). **^{1}H NMR** (400 MHz, DMSO-*d$_6$*) δ 8.91 (d, *J* = 1.3 Hz, 1H), 8.88 (d, *J* = 4.8 Hz, 1H), 8.6 (d, *J* = 8.12, 1H), 8.06 (dd, *J* = 8.1, 5.6 Hz, 1H), 5.45 (s, 1H).

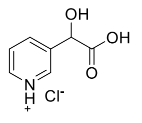

Chemical Formula: $C_7H_8ClNO_3$
Molecular Weight: 189,60

General procedure for synthesis of diketones 1 starting from the corresponding aldehydes.[5]

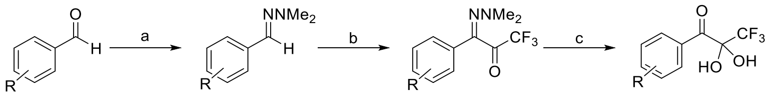

Reagents and conditions: (a) 1,1-dimethylhydrazine, MeOH, 60 °C, o.n.; (b) 2,2,2-trifluoroacetic anhydride, 2,6-lutidine, $CHCl_3$, 40 °C, 4 h; (c) 5 N H_2SO_4, 60 °C, 4 h.

Step a: A mixture of aldehyde and 1,1-dimethylhydrazine (1.3 eq) in toluene (5 mL/mmol) was stirred overnight at 22 °C then concentrated to obtain crude product.

Step b: A mixture of the above hydrazone (**Step a**), 2,6-lutidine (3 eq) and $CHCl_3$ (4 mL/mmol) at 0 °C was treated dropwise with trifluoroacetic anhydride (10 eq) and stirred overnight at 22 °C. The reaction mixture was partitioned between dichloromethane and water. The organic layer was washed with saturated aqueous $NaHCO_3$, 1 M HCl, brine, dried over Na_2SO_4, filtered and concentrated to get the crude product.

Step c: The suspension of starting material from **Step b** in 5 N H_2SO_4 (8 mL/mmol) was stirred overnight at 60–80 °C. The reaction mixture was partitioned betweeen diethyl ether and brine, dried over Na_2SO_4, filtered and concentrated.

2-(4-bromobenzylidene)-1,1-dimethylhydrazine.[6] CAS: 1848238-94-7. Prepared from 4-bromobenzaldehyde (5.0 g, 27 mmol) according to *General procedure for synthesis of diketones from starting aldehyde, Step a.* The crude product was purified by flash column chromatography, eluent ethyl acetate/hexane 1:6 to get the title compound as orange solid (5.0 g, 82% yield). **^{1}H NMR** (400 MHz, Chloroform-*d*) δ 7.42 (s, 4H), 7.13 (s, 1H), 2.97 (s, 6H).

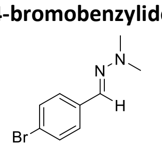

Chemical Formula: $C_9H_{11}BrN_2$
Molecular Weight: 227,11

1,1-dimethyl-2-(4-nitrobenzylidene)hydrazine.[7] CAS: 10424-92-7. Prepared from 4-nitrobenzaldehyde (10.0 g, 66.21 mmol) according to *General procedure for synthesis of diketones from starting aldehyde, Step a.* The crude product as an orange solid, was used directly in the next step (12.7 g, 99% yield). **^{1}H NMR** (400 MHz, Chloroform-*d*) δ 8.16 (d, *J* = 8.9 Hz, 2H), 7.63 (d, *J* = 8.9 Hz, 2H), 7.10 (s, 1H), 3.10 (s, 6H).

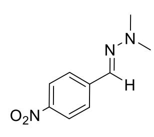

Chemical Formula: $C_9H_{11}N_3O_2$
Molecular Weight: 193,21

5-((2,2-dimethylhydrazineylidene)methyl)-2-nitrophenol. Prepared from 3-hydroxy-4-nitrobenzaldehyde (5.0 g, 30.0 mmol) according to *General procedure for synthesis of diketones from starting aldehyde, Step a.* The crude product as a red solid was used directly in the next step (6.37 g, quant. yield). **^{1}H NMR** (400 MHz, DMSO-*d$_6$*) δ 10.84 (s, 1H), 7.89 (d, *J* = 8.7 Hz, 1H), 7.18 (d, *J* = 1.7 Hz, 1H), 7.16 (s, 1H), 7.12 (dd, *J* = 8.8, 1.7 Hz, 1H), 3.03 (s, 6H).

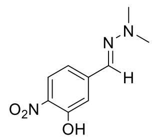

Chemical Formula: $C_9H_{11}N_3O_3$
Molecular Weight: 209,21

3-((2,2-dimethylhydrazineylidene)methyl)phenol. CAS:59670-31-4. Prepared from 3-hydroxybenzaldehyde (7.0 g, 57.3 mmol, 1 eq) according to *General procedure for synthesis of diketones from starting aldehyde, Step a.* The crude product was triturated with toluene to get the title compound as an off-white solid (8.80 g, 93.5% yield). **^{1}H NMR** (400 MHz, Chloroform-*d*) δ 7.38–7.11 (m, 3H), 7.04 (d, *J* = 7.7 Hz, 1H), 6.72 (ddd, *J* = 8.0, 2.5, 0.7 Hz, 1H), 5.68 (s, 1H), 2.96 (s, 6H).

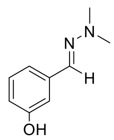

Chemical Formula: $C_9H_{12}N_2O$
Molecular Weight: 164,21

2-((2,2-dimethylhydrazineylidene)methyl)phenol. CAS: 59244-19-8. Prepared from 2-hydroxybenzaldehyde (6.0 g, 49.13 mmol) according to *General procedure for synthesis of diketones from starting aldehyde* (**Step a**) to get the title compound as an off-white oil (8.1 g, 100% yield). **^{1}H NMR** (400 MHz, Chloroform-*d*) δ 11.57 (s, 1H), 7.39 (s, 1H), 7.19–7.09 (m, 2H), 6.92 (dd, *J* = 8.1, 1.0 Hz, 1H), 6.85 (td, *J* = 7.5, 1.2 Hz, 1H), 2.93 (s, 6H).

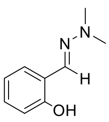

Chemical Formula: $C_9H_{12}N_2O$
Molecular Weight: 164,21

2-(4-methoxybenzylidene)-1,1-dimethylhydrazine.[8] CAS: 341979-31-5. Prepared from *p*-anisaldehyde (5.0 g, 36.72 mmol) according to *General procedure for synthesis of diketones from starting aldehyde, Step a.* The crude product was purified by flash column chromatography, eluent hexane/tetrahydrofurane 4:11 to get the title compound as red solid (4.2 g, 64% yield). **^{1}H NMR** (400 MHz, Chloroform-*d*) δ 7.51 (d, *J* = 8.7 Hz, 2H), 7.26 (s, 1H), 6.86 (d, *J* = 8.7 Hz, 2H), 3.80 (s, 3H), 2.92 (s, 6H).

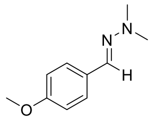

Chemical Formula: $C_{10}H_{14}N_2O$
Molecular Weight: 178,24

2-(4-(diethoxymethyl)benzylidene)-1,1-dimethylhydrazine. Prepared from 4-(diethoxymethyl)benzaldehyde (5.0 g, 24.00 mmol) according to *General procedure for synthesis of diketones from starting aldehyde, Step a.* The crude product as a red solid was used directly in the next step (6.23 g, 100% yield). **^{1}H NMR** (400 MHz, Chloroform-*d*) δ 7.55 (d, *J* = 8.3 Hz, 2H), 7.42 (d, *J* = 8.2 Hz, 2H), 7.24 (s, 1H), 5.50 (s, 1H), 3.72–3.45 (m, 4H), 2.97 (s, 6H), 1.23 (t, *J* = 7.1 Hz, 6H).

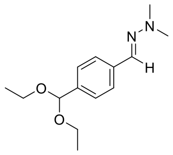

Chemical Formula: $C_{14}H_{22}N_2O_2$
Molecular Weight: 250,34

6-((2,2-dimethylhydrazineylidene)methyl)benzo[*d*]thiazol-2-amine. Prepared from 4-bromobenzaldehyde (5.0 g, 28.06 mmol) according to *General procedure for synthesis of diketones from starting aldehyde, Step a* in methanol at 60 °C. The crude product was purified by flash column chromatography, eluent dichloromethane/methanol 20:1 to get the title compound as orange solid (3.67 g, 59% yield). **^{1}H NMR** (400 MHz, DMSO-*d$_6$*) δ 7.79 (d, *J* = 1.6 Hz, 1H), 7.50 (s, 2H), 7.40 (dd, *J* = 8.4, 1.7 Hz, 1H), 7.32 (s, 1H), 7.27 (d, *J* = 8.3 Hz, 1H), 2.86 (s, 6H).

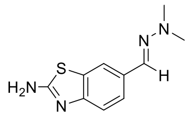

Chemical Formula: $C_{10}H_{12}N_4S$
Molecular Weight: 220,29

3-(4-bromophenyl)-3-(2,2-dimethylhydrazineylidene)-1,1,1-trifluoropropan-2-one. Prepared from (*E*)-2-(4-bromobenzylidene)-1,1-dimethylhydrazin (5.0 g, 22.12 mmol, 1 eq) according to *General procedure for synthesis of diketones from starting aldehyde, Step b.* The crude product was purified by recrystallization from ethyl acetate/hexane 1:9 to get the title compound as light orange solid (3.23 g, 45% yield). **^{1}H NMR** (400 MHz, Chloroform-*d*) δ 7.51 (d, *J* = 8.5 Hz, 2H), 7.10 (d, *J* = 8.5 Hz, 2H), 3.08 (s, 6H). **^{19}F NMR** (376 MHz, Chloroform-*d*) δ −68.99 (s).

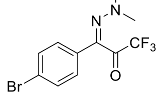

Chemical Formula: $C_{11}H_{10}BrF_3N_2O$
Molecular Weight: 323,11

3-(2,2-dimethylhydrazineylidene)-1,1,1-trifluoro-3-(4-nitrophenyl)propan-2-one.[5] CAS: 111269-45-5. Prepared from 1,1-dimethyl-2-(4-nitrobenzylidene)hydrazine (4.1 g, 21.22 mmol) according to *General procedure for synthesis of diketones from starting aldehyde, Step b.* The crude product was purified by recrystallization from chloroform followed by trituration with diethyl ether to get the title compound as orange oil (2.78 g, 45.30% yield). **^{1}H NMR** (400 MHz, Chloroform-*d*) δ 8.25 (d, *J* = 8.8 Hz, 2H), 7.43 (d, *J* = 8.8 Hz, 2H), 3.12 (s, 6H). **^{19}F NMR** (376 MHz, Chloroform-*d*) δ −69.15 (s).

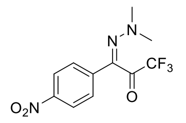

Chemical Formula: $C_{11}H_{10}F_3N_3O_3$
Molecular Weight: 289,21

3-(2,2-dimethylhydrazineylidene)-1,1,1-trifluoro-3-(3-hydroxy-4-nitrophenyl)propan-2-one. Prepared from 5-((2,2-dimethylhydrazineylidene)methyl)-2-nitrophenol (6.40 g, 30.4 mmol) according to *General procedure for synthesis of diketones from starting aldehyde, Step b,* to obtain crude product, which was used as such in the next step (11.15 g). **^{1}H NMR** (400 MHz, DMSO-d_6) δ 11.14 (s, 1H), 7.88 (d, *J* = 8.4 Hz, 1H), 7.00 (d, *J* = 1.6 Hz, 1H), 6.88 (dd, *J* = 8.4, 1.7 Hz, 1H), 3.13 (s, 6H). **^{19}F NMR** (376 MHz, DMSO-d_6) δ −67.53 (s).

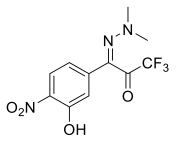

Chemical Formula: $C_{11}H_{10}F_3N_3O_4$
Molecular Weight: 305,21

3-(2,2-dimethylhydrazineylidene)-1,1,1-trifluoro-3-(3-hydroxyphenyl)propan-2-one. Prepared from 3-((2,2-dimethylhydrazineylidene)methyl)phenol (8.80 g, 53.6 mmol) according to *General procedure for synthesis of diketones from starting aldehyde, Step b.* The crude product was purified by flash column chromatography, eluent ethyl acetate/hexane 1:3 to get the title compound as brown oil (4.94 g, 35% yield). **^{1}H NMR** (400 MHz, Chloroform-*d*) δ 7.22–7.11 (m, 1H), 6.81–6.67 (m, 2H), 6.61 (dd, *J* = 2.4, 1.5 Hz, 1H), 5.72 (s, 1H), 3.09 (s, 6H). **^{19}F NMR** (376 MHz, Chloroform-*d*) δ −68.57 (s).

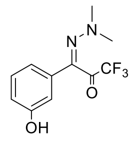

Chemical Formula: $C_{11}H_{11}F_3N_2O_2$
Molecular Weight: 260,22

3-(2,2-dimethylhydrazineylidene)-1,1,1-trifluoro-3-(2-hydroxyphenyl)propan-2-one. Prepared from 2-((2,2-dimethylhydrazineylidene)methyl)phenol (8.3 g, 50.54 mmol) according to *General procedure for synthesis of diketones from starting aldehyde, Step b.* The crude product was triturated successively with ethyl acetate, hexane, cyclohexane and diethyl ether to obtain a mixture of stereoisomers as yellow solid, which was used as such in the next step (4.8 g, 36% yield).

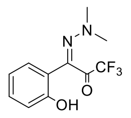

Chemical Formula: $C_{11}H_{11}F_3N_2O_2$
Molecular Weight: 260,22

3-(2,2-dimethylhydrazineylidene)-1,1,1-trifluoro-3-(4-methoxyphenyl)propan-2-one.[5] CAS: 111269-46-6. Prepared from (*E*)-2-(4-methoxybenzylidene)-1,1-dimethylhydrazine (4.2 g, 23.58 mmol) according to *General procedure for synthesis of diketones from starting aldehyde* (**Step b**). The crude product was purified by flash column chromatography, eluent ethyl acetate/hexane 1/9 to get the title compound (0.67g, 10.4% yield). **^{1}H NMR** (400 MHz, Chloroform-*d*) δ 7.13 (d, *J* = 8.8 Hz, 2H), 6.89 (d, *J* = 8.8 Hz, 2H), 3.83 (s, 3H), 3.06 (s, 6H). **^{19}F NMR** (376 MHz, Chloroform-*d*) δ −68.81 (s).

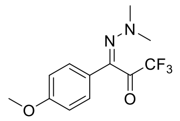

Chemical Formula: $C_{12}H_{13}F_3N_2O_2$
Molecular Weight: 274,24

3-(4-(diethoxymethyl)phenyl)-3-(2,2-dimethylhydrazineylidene)-1,1,1-trifluoropropan-2-one. Prepared from 2-(4-(diethoxymethyl)benzylidene)-1,1-dimethylhydrazine (6.23 g, 24.9 mmol) *according to General procedure for synthesis of diketones from starting aldehyde, Step b*, to obtain crude product, which was used directly in the next step without any further purification (10.1 g).

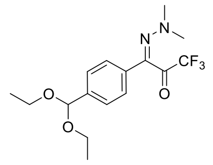

Chemical Formula: $C_{16}H_{21}F_3N_2O_3$
Molecular Weight: 346,35

***N*-(6-(1-(2,2-dimethylhydrazineylidene)-3,3,3-trifluoro-2-oxopropyl)benzo[*d*]thiazol-2-yl)-2,2,2-trifluoroacetamide.**

Prepared from 6-((2,2-dimethylhydrazineylidene)methyl)benzo[*d*]thiazol-2-amine (1.0 g, 4.54 mmol) according to *General procedure for synthesis of diketones from starting aldehyde, Step b.* During extraction, precipitate was formed. The precipitate was collected and washed with water and dichloromethane to get the title compound as white solid (1.06 g, 57%). **^{1}H NMR** (400 MHz, DMSO-d_6) δ 7.94 (d, *J* = 1.3 Hz, 1H), 7.59 (d, *J* = 8.3 Hz, 1H), 7.44 (dd, *J* = 8.3, 1.6 Hz, 1H), 3.07 (s, 6H). **^{19}F NMR** (376 MHz, DMSO-d_6) δ −67.42 (s), −74.23 (s).

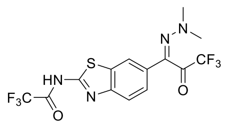

Chemical Formula: $C_{14}H_{10}F_6N_4O_2S$
Molecular Weight: 412,31

3,3,3-trifluoro-2,2-dihydroxy-1-phenylpropan-1-one (1a). CAS: 36750-88-6. Prepared according to the literature procedure.[5]

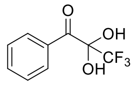

Chemical Formula: $C_9H_7F_3O_3$
Molecular Weight: 220,1472

White crystals. **^{1}H NMR** (400 MHz, Chloroform-*d*) δ 8.32 (d, *J* = 7.9 Hz, 2H), 7.65 (dd, *J* = 10.6, 4.3 Hz, 1H), 7.48 (dd, *J* = 11.1, 4.8 Hz, 2H), 5.01 (s, 2H). **^{19}F NMR** (376 MHz, Chloroform-*d*) δ −81.08 (s). **^{13}C NMR** (101 MHz, Chloroform-*d*) δ 192.20, 135.37, 131.98, 131.67, 128.68, 121.65 (q, *J* = 287.9 Hz), 94.10 (q, *J* = 34.4 Hz). **HRMS** (ESI) m/z: $[M - OH]^+$ Calcd for $C_9H_6O_2F_3$ 203.0314; Found 203.0314 (− 1.48 ppm).

1-(4-bromophenyl)-3,3,3-trifluoro-2,2-dihydroxypropan-1-one (1b).[9] CAS: 1007884-96-9. Prepared from 3-(4-bromophenyl)-3-(2,2-dimethylhydrazineylidene)-1,1,1-trifluoropropan-2-one (3.23 g, 10.00 mmol) according to *General procedure for synthesis of diketones from starting aldehyde, Step c.* The crude product was recrystallized from ethyl acetate/hexane 1:3 to get the title compound as white solid (0.57 g, 20% yield). **^{1}H NMR** (400 MHz, Chloroform-*d*) δ 8.19 (d, *J* = 8.6 Hz, 2H), 7.65 (d, *J* = 8.7 Hz, 2H), 4.92 (s, 2H). **^{19}F NMR** (376 MHz, Chloroform-*d*) δ −81.21 (s). **^{13}C NMR** (101 MHz, Chloroform-*d*) δ 191.25, 133.32, 132.17, 131.26, 130.44, 121.56 (q, *J* = 287.8 Hz), 93.96 (q, *J* = 34.4 Hz). **HRMS** (ESI) m/z: $[M - H]^-$ Calcd for $C_9H_5O_3BrF_3$ 296.9379; Found 296.9377 (− 0.86 ppm).

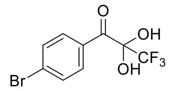

Chemical Formula: $C_9H_6BrF_3O_3$
Exact Mass: 297,9452

3,3,3-trifluoro-2,2-dihydroxy-1-(4-nitrophenyl)propan-1-one (1e).[5] CAS: 111269-65-9. Prepared from 3-(2,2-dimethylhydrazineylidene)-1,1,1-trifluoro-3-(4-nitrophenyl)propan-2-one (2.78 g, 9.61 mmol) according to *General procedure for synthesis of diketones from starting aldehyde, Step c,* to get the crude product as an orange solid which was used directly in the next step without any further purification (2.22 g, 93% yield). **^{1}H NMR** (400 MHz, Chloroform-*d*) δ 8.49 (d, *J* = 8.8 Hz, 2H), 8.34 (d, *J* = 9.1 Hz, 2H), 4.80 (s, 2H). **^{19}F NMR** (376 MHz, Chloroform-*d*) δ −81.08 (s). **^{13}C NMR** (101 MHz, Chloroform-*d*) δ 191.71, 151.18, 136.67, 132.84, 123.64, 121.53 (q, *J* = 288.2 Hz), 94.10 (q, *J* = 34.3 Hz). **HRMS** (ESI) m/z: $[M - H]^-$ Calcd for $C_9H_5O_5NF_3$ 264.0125; Found 264.0128 (− 0.99 ppm).

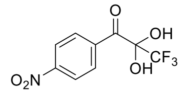

Chemical Formula: $C_9H_6F_3NO_5$
Exact Mass: 265,0198

3,3,3-trifluoro-2,2-dihydroxy-1-(3-hydroxy-4-nitrophenyl)propan-1-one (1f). Prepared from 3-(2,2-dimethylhydrazineylidene)-1,1,1-trifluoro-3-(3-hydroxy-4-nitrophenyl)propan-2-one (11.15 g, 16.38 mmol) according to *General procedure for synthesis of diketones from starting aldehyde, Step c.* The crude product was purified by flash column chromatography, eluent ethyl acetate/hexane 1:2 to get the title compound as yellow oil (1.30 g, 30% yield). **^{1}H NMR** (400 MHz, DMSO-*d$_6$*) δ 11.45 (s, 1H), 8.50 (s, 2H), 7.96 (d, *J* = 8.6 Hz, 1H), 7.93 (d, *J* = 1.7 Hz, 1H), 7.68 (dd, *J* = 8.6, 1.7 Hz, 1H). **^{19}F NMR** (376 MHz, Methanol-*d$_4$*) δ −80.08 (s). **^{13}C NMR** (101 MHz, Methanol-d$_4$) δ 193.08, 154.04, 141.03, 139.28, 126.20, 123.14 (q, *J* = 289.1 Hz), 122.97, 121.92, 98.49 (q, *J* = 31.9 Hz). **HRMS** (ESI) m/z: $[M - H_3O]^-$ Calcd for $C_9H_3O_5NF_3$ 261.9969; Found 261.9965 (− 1.60 ppm).

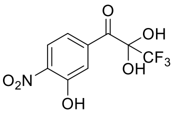

Chemical Formula: $C_9H_6F_3NO_6$
Molecular Weight: 281,1432

3,3,3-trifluoro-2,2-dihydroxy-1-(3-hydroxyphenyl)propan-1-one (1h). Prepared from 3-(2,2-dimethylhydrazineylidene)-1,1,1-trifluoro-3-(3-hydroxyphenyl)propan-2-one (4.78 g, 18.37 mmol) according to *General procedure for synthesis of diketones from starting aldehyde, Step c.* The crude product was triturated with dichloromethane to get the title compound as off-white solid (2.76 g, 69%). **^{1}H NMR** (400 MHz, DMSO-*d$_6$*) δ 9.79 (s, 1H), 8.22 (s, 2H), 7.64 (ddd, *J* = 11.1, 5.3, 1.4 Hz, 2H), 7.32 (t, *J* = 7.9 Hz, 1H), 7.04 (ddd, *J* = 8.1, 2.5, 0.9 Hz, 1H). **^{19}F NMR** (376 MHz, DMSO-*d$_6$*) δ −80.35 (s). **^{13}C NMR** (101 MHz, DMSO-*d$_6$*) δ 192.55, 157.04, 134.42, 129.28, 122.66 (q, *J* = 290.4 Hz), 121.29, 120.71, 116.89, 93.67 (q, *J* = 30.0 Hz). **HRMS** (ESI) m/z: $[M - H_3O]^-$ Calcd for $C_9H_4O_3F_3$ 217.0118; Found 217.0111 (− 3.42 ppm).

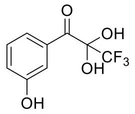

Chemical Formula: $C_9H_7F_3O_4$
Molecular Weight: 236,1462

3,3,3-trifluoro-1-(2-hydroxyphenyl)propane-1,2-dione (1i). Prepared from 3-(2,2-dimethylhydrazineylidene)-1,1,1-trifluoro-3-(2-hydroxyphenyl)propan-2-one (4.8 g, 18.45 mmol) according to *General procedure for synthesis of diketones from starting aldehyde, Step c.* The crude product was filtered through short pad of silica, eluent ethyl acetate, to get the title compound as yellow solid (2.20 g, 55% yield). **^{1}H NMR** (400 MHz, DMSO-*d$_6$*) δ 9.70 (s, 1H), 7.88 (ddd, *J* = 8.5, 7.3, 1.5 Hz, 1H), 7.78 (ddd, *J* = 7.7, 1.4, 0.6 Hz, 1H), 7.39 (d, *J* = 8.4 Hz, 1H), 7.34–7.18 (m, 1H). **^{19}F NMR** (376 MHz, DMSO-*d$_6$*) δ −80.99 (s). **^{13}C NMR** (101 MHz, DMSO-*d$_6$*) δ 192.21, 169.88, 140.52, 125.09, 123.87, 120.90 (q, *J* = 285.9 Hz), 118.33, 113.56, 97.70 (q, *J* = 33.3 Hz). **HRMS** (ESI) m/z: $[M - OH]^-$ Calcd for $C_9H_6O_3F_3$ 219.0264; Found 219.0261 (− 1.21 ppm).

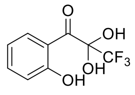

Chemical Formula: $C_9H_7F_3O_4$
Molecular Weight: 236,1462

3,3,3-trifluoro-2,2-dihydroxy-1-(4-methoxyphenyl)propan-1-one (1j).[5] CAS: 111269-66-0. Prepared from 3-(2,2-dimethylhydrazineylidene)-1,1,1-trifluoro-3-(4-methoxyphenyl)propan-2-one (0.67 g, 2.44 mmol) according to *General procedure for synthesis of diketones from starting aldehyde, Step c.* The crude product was purified by flash column chromatography, eluent ethyl acetate/hexane 1:4 to get the title compound as light yellow crystals (0.31 g, 55% yield). **^{1}H NMR** (400 MHz, Chloroform-*d*) δ 8.34 (d, *J* = 8.9 Hz, 2H), 6.96 (d, *J* = 9.1 Hz, 2H), 4.90 (s, 2H), 3.91 (s, 3H). **^{19}F NMR** (376 MHz, Chloroform-*d*) δ −81.36. **^{13}C NMR** (101 MHz, Chloroform-*d*) δ 189.96, 165.40, 134.87, 124.37, 121.77 (q, *J* =

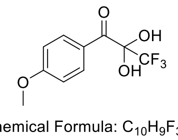

Chemical Formula: $C_{10}H_9F_3O_4$
Exact Mass: 250,0453

287.8 Hz), 114.04, 93.93 (q, J = 34.2 Hz), 55.78. **HRMS** (ESI) m/z: $[M - OH]^+$ Calcd for $C_{10}H_8O_3F_3$ 233.0420; Found 233.0418 (−0.97 ppm); m/z: $[M - H]^-$ Calcd for $C_{10}H_8O_4F_3$ 249.0380, found 249.0377 (− 1.23 ppm).

3-(3,3,3-trifluoro-2,2-dihydroxypropanoyl)phenyl acetate (1k). The solution of the diketone **1h** (200 mg, 0.917 mmol) in Ac_2O (3 eq) was stirred overnight at 60 °C. The reaction mixture was monitored by TLC analysis and after full conversion it was concentrated. The oily residue was partitioned between ethyl acetate and half-saturated aqueous solution of K_2CO_3. The organic layer was washed with brine, dried over Na_2SO_4, filtered and concentrated. The obtained crude product was further purified by preparative thin layer chromatography, eluent ethyl acetate/hexane 1:2 to get the title compound as white solid (0.043 g, 18% yield). **^{1}H NMR** (400 MHz, DMSO-d_6) δ 8.41 (s, 2H), 8.19–8.03 (m, 1H), 7.97–7.88 (m, 1H), 7.58 (t, J = 8.0 Hz, 1H), 7.45 (ddd, J = 8.1, 2.4, 1.0 Hz, 1H), 2.31 (s, 3H). **^{19}F NMR** (376 MHz, Methanol-d_4) δ −80.32 (s). **^{13}C NMR** (101 MHz, Methanol-d_4) δ 193.25, 170.88, 152.14, 136.31, 130.63, 129.08, 128.78, 124.93, 123.19 (q, J = 288.6 Hz), 98.67 (q, J = 32.2 Hz), 20.84. **HRMS** (ESI) m/z: $[M - OH]^+$ Calcd for $C_{11}H_8O_4F_3$ 261.0369; Found 261.0365 (−1.61 ppm); m/z: $[M - H]^-$ Calcd for $C_{11}H_8O_5F_3$ 277.0329; Found 277.0326 (− 1.16 ppm).

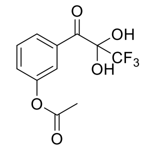

Chemical Formula: $C_{11}H_9F_3O_5$
Molecular Weight: 278,1832

4-(3,3,3-trifluoro-2,2-dihydroxypropanoyl)benzaldehyde (1l). Prepared from 3-(4-(diethoxymethyl)phenyl)-3-(2,2-dimethylhydrazineylidene)-1,1,1-trifluoropropan-2-one (10.1 g, 29.2 mmol) according to *General procedure for synthesis of diketones from starting aldehyde, Step c.* The crude product was purified by flash column chromatography, eluent ethyl acetate/hexane 1:4 to get the title compound as yellow oil (0.93 g, 13% yield). **^{1}H NMR** (400 MHz, DMSO-d_6) δ 10.12 (s, 1H), 8.50 (s, 2H), 8.35 (d, J = 8.3 Hz, 2H), 8.05 (d, J = 8.6 Hz, 2H). **^{19}F NMR** (376 MHz, DMSO-d_6) δ -80.48 (s). **^{13}C NMR** (101 MHz, DMSO-d_6) δ 193.07, 192.56, 138.90, 137.64, 130.80, 129.17, 122.56 (q, J = 290.5 Hz), 93.56 (q, J = 30.2 Hz). **HRMS** (ESI) m/z: $[M - H]^-$ Calcd for $C_{10}H_6O_4F_3$ 247.0224; Found 247.0220 (− 1.28 ppm).

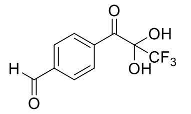

Chemical Formula: $C_{10}H_7F_3O_4$
Molecular Weight: 248,1572

1-(2-aminobenzo[*d*]thiazol-6-yl)-3,3,3-trifluoropropane-1,2-dione (1n). Prepared from *N*-(6-(1-(2,2-dimethylhydrazineylidene)-3,3,3-trifluoro-2-oxopropyl)benzo[*d*]thiazol-2-yl)-2,2,2-trifluoroacetamide (1.00 g, 2.42 mmol) according to *General procedure for synthesis of diketones from starting aldehyde, Step c.* After cooling the reaction mixture to 0 °C, the formed precipitate was collected and washed with water to get the title compound as off-white solid (0.60 g, 91% yield). **^{1}H NMR** (400 MHz, DMSO-d_6) δ 9.00 (s, 2H), 8.64 (d, J = 1.5 Hz, 1H), 8.23 (dd, J = 8.6, 1.7 Hz, 1H), 7.48 (d, J = 8.6 Hz, 1H). **^{19}F NMR** (376 MHz, DMSO-d_6) δ −80.33 (s). **^{13}C NMR** (101 MHz, DMSO-d_6) δ 190.93, 170.66, 150.38, 129.66, 127.72, 127.31, 124.97, 122.69 (q, J = 290.4 Hz), 115.28, 93.76 (q, J = 30.2 Hz). **HRMS** (ESI) m/z: $[M - OH]^-$ Calcd for $C_{10}H_6O_2N_2F_3S$ 275.0097; Found 275.0089 (− 2.51 ppm).

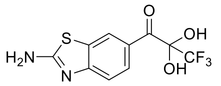

Chemical Formula: $C_{10}H_7F_3N_2O_3S$
Molecular Weight: 292,2322

General procedure for the synthesis of CF_3 substituted α-hydroxyketones 2.[10]

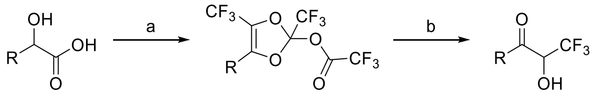

Reagents and conditions: (a) 2,2,2-trifluoroacetic anhydride, pyridine, benzene, 85 °C, 3 h; (b) 5% HCl, 70 °C, 15 min.

Step a: A mixture of α-hydroxycarboxylic acid, dry pyridine (6 eq) and benzene (3.5 mL/mmol) was cooled to 0 °C and treated dropwise with trifluoroacetic anhydride (5 eq) under a stream of argon. After stirring at 85 °C for 3 h the reaction mixture was cooled to 70 °C and used directly in the next step.

Step b: 5% HCl (aq) (2.5 mL/mmol) was added to the above mixture and stirred for 15 minutes. The reaction mixture was partitioned between ethyl acetate and water. The organic layer was washed with water, 1 M HCl, saturated aqueous $NaHCO_3$, brine, dried over Na_2SO_4, filtered and concentrated to get the crude product.

3,3,3-trifluoro-2-hydroxy-1-phenylpropan-1-one (2a).[11] CAS: 322-01-0. Prepared from D-mandelic acid (5.00 g, 32.9 mmol) according to *General procedure for synthesis of CF_3 substituted α-hydroxyketones.* The crude product was recrystalised from cyclohexane to get the title compound as white crystals (2.0 g, 30% yield). **^{1}H NMR** (400 MHz, Methanol-d_4) δ 8.08 (dd, J = 8.3, 1.1 Hz, 2H), 7.84 – 7.60 (m, 1H), 7.61 – 7.46 (m, 2H), 5.61 (q, J = 7.2 Hz, 1H). **^{19}F NMR** (376 MHz, Methanol-d_4) δ −75.84 (d, J = 6.9 Hz). **^{13}C NMR** (101 MHz, Methanol-d_4) δ 194.77, 136.17, 135.35, 130.32, 129.82, 124.81 (q, J = 283.1 Hz), 72.46 (q, J = 30.2 Hz). **HRMS** (ESI) m/z: $[M - H]^-$ Calcd for $C_9H_6O_2F_3$ 203.0325; Found 203.0319 (− 2.89 ppm).

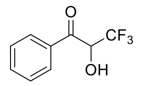

Chemical Formula: $C_9H_7F_3O_2$
Molecular Weight: 204,1482

Alternatively, **2a** was prepared by iron/acetic acid mediated chemoselective monoreduction of diketone **1a:** To a solution of **1a** (718 mg, 3.26 mmol) in AcOH (33 mL, 10 mL/mmol), iron powder (1.80 g, 32.6 mmol, 10 eq) was added and the resulting suspension vigorously stirred overnight. After completion of the reaction, the pH was adjusted to 7 by adding 2 M NaOH(aq).

Water (20 mL) and methanol (20 mL) were added, the resulting suspension filtered trough cotton and concentrated under reduced pressure. The oily residue was partitioned between water (50 mL) and ethyl acetate (3 x 50 mL). The combined organic layers were washed with saturated aqueous $NaHCO_3$, brine, dried over Na_2SO_4, filtered and concentrated. The crude product was purified by flash column chromatography, eluent ethyl acetate/hexane 1:6 to get the title compound as white crystals (285 mg, 43% yield). **^{1}H NMR** (400 MHz, Chloroform-*d*) δ 7.99 (d, *J* = 7.5 Hz, 2H), 7.81 – 7.65 (m, 1H), 7.62 – 7.46 (m, 2H), 5.43 (dq, *J* = 8.3, 6.7 Hz, 1H), 4.27 (d, *J* = 8.3 Hz, 1H). **^{19}F NMR** (376 MHz, Chloroform-*d*) δ −73.83 (d, *J* = 6.6 Hz).

1-(4-chlorophenyl)-3,3,3-trifluoro-2-hydroxypropan-1-one (2c).[12] CAS: 1364625-45-5. Prepared from 4-chloromandelic acid

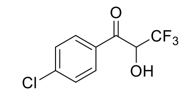

Chemical Formula: $C_9H_6ClF_3O_2$
Molecular Weight: 238,59

(2.00 g, 10.7 mmol) according to *General procedure for synthesis of CF_3 substituted α-hydroxyketones.* The crude product was triturated with cyclohexane to get the title compound as white solid (0.7 g, 27% yield). **^{1}H NMR** (400 MHz, Chloroform-*d*) δ 7.93 (d, *J* = 8.6 Hz, 2H), 7.53 (d, *J* = 8.7 Hz, 2H), 5.38 (q, *J* = 6.6 Hz, 1H), 4.16 (d, *J* = 24.9 Hz, 1H). **^{19}F NMR** (376 MHz, Chloroform-*d*) δ −73.82 (d, *J* = 6.2 Hz). **^{13}C NMR** (101 MHz, Chloroform-*d*) δ 192.11, 142.30, 131.76, 130.93, 129.61, 122.36 (q, *J* = 284.2 Hz), 71.18 (q, *J* = 31.5 Hz). **HRMS** (ESI) m/z: $[M - H]^-$ Calcd for $C_9H_5O_2ClF_3$ 236.9936; Found 236.9930 (− 2.26 ppm).

3,3,3-trifluoro-1-(4-fluorophenyl)-2-hydroxypropan-1-one (2d).[12] CAS: 1579305-47-7. Prepared from *p*-fluoromandelic acid

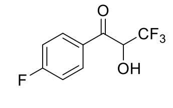

Chemical Formula: $C_9H_6F_4O_2$
Molecular Weight: 222,14

(1.00 g, 5.87 mmol) according to *General procedure for synthesis of CF_3 substituted α-hydroxyketones.* The crude product was purified by flash column chromatography, eluent ethyl acetate/hexane 1:6 to get the title compound as light brown solid (530 mg, 41% yield). **^{1}H NMR** (400 MHz, Chloroform-*d*) δ 8.04 (dd, *J* = 8.8, 5.3 Hz, 2H), 7.24 (dd, *J* = 14.8, 6.0 Hz, 2H), 5.38 (q, *J* = 6.6 Hz, 1H), 4.17 (s, 1H). **^{19}F NMR** (376 MHz, Chloroform-*d*) δ −73.88 (d, *J* = 6.7 Hz), (−100.12)–(−100.61) (m). **^{13}C NMR** (101 MHz, Chloroform-*d*) δ 191.56, 168.46, 165.88, 132.52 (d, *J* = 9.7 Hz), 122.41 (q, *J* = 284.3 Hz), 116.60 (d, *J* = 22.4 Hz), 71.10 (q, *J* = 31.5 Hz). **HRMS** (ESI) m/z: $[M - H]^-$ Calcd for $C_9H_5O_2F_4$ 221.0231; Found 221.0225 (− 2.79 ppm).

3,3,3-trifluoro-2-hydroxy-1-(4-hydroxyphenyl)propan-1-one (2g). Prepared from *p*-hydroxymandelic acid (2.00 g, 11.9

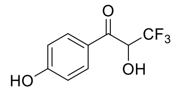

Chemical Formula: $C_9H_7F_3O_3$
Molecular Weight: 220,15

mmol) according to *General procedure for synthesis of CF_3 substituted α-hydroxyketones.* The crude product was purified by flash column chromatography, eluent ethyl acetate/hexane 1:1 to get the title compound as white solid (1.2 g, 45% yield). **^{1}H NMR** (400 MHz, DMSO-*d$_6$*) δ 10.63 (s, 1H), 7.95 (d, *J* = 8.8 Hz, 2H), 7.00 (s, 1H), 6.89 (d, *J* = 8.8 Hz, 2H), 5.63 (s, 1H). **^{19}F NMR** (376 MHz, DMSO-*d$_6$*) δ −73.15 (d, *J* = 7.0 Hz). **^{13}C NMR** (101 MHz, DMSO-*d$_6$*) δ 191.11, 163.06, 131.98, 126.06, 123.94 (q, *J* = 283.8 Hz), 115.40, 69.99 (q, *J* = 28.8 Hz). **HRMS** (ESI) m/z: $[M - H]^-$ Calcd for $C_9H_6O_3F_3$ 219.0274; Found 219.0267 (− 3.20 ppm).

3,3,3-trifluoro-2-hydroxy-1-(pyridin-3-yl)propan-1-one (2m).[12] CAS: 138380-45-7. Prepared from 2-hydroxy-2-(pyridin-3-

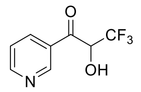

Chemical Formula: $C_8H_6F_3NO_2$
Molecular Weight: 205,14

yl)acetic acid (1.65 g, 10.7 mmol) according to *General procedure for synthesis of CF_3 substituted α-hydroxyketones.* The reaction mixture was partitioned between ethyl acetate and water. 2 M NaOH was added to the water layer to reach pH 7–8, then it was re-extracted with ethyl acetate. Organic layer was dried over Na_2SO_4, filtered and concentrated. The crude product was purified by flash column chromatography, eluent dichlorometane/methanol/amonium hydroxyde 20:1:0.1 followed by trituration with ethyl acetate to get the title compound as light brown solid (61 mg, 2.8% yield). **^{1}H NMR** (400 MHz, Chloroform-*d*) δ 9.20 (d, *J* = 2.0 Hz, 1H), 8.91 (dd, *J* = 4.8, 1.7 Hz, 1H), 8.28 (d, *J* = 8.0 Hz, 1H), 7.53 (ddd, *J* = 8.0, 4.9, 0.8 Hz, 1H), 5.41 (d, *J* = 5.5 Hz, 1H), 4.34 (s, 1H). **^{19}F NMR** (376 MHz, Chloroform-*d*) δ -73.69 (d, *J* = 6.7 Hz). **^{13}C NMR** (101 MHz, Chloroform-*d*) δ 192.41, 155.09, 150.34, 136.75, 129.31, 123.99, 122.22 (q, *J* = 284.4 Hz), 71.71 (q, *J* = 31.5 Hz). **HRMS** (ESI) m/z: $[M + H]^+$ Calcd for $C_8H_7O_2NF_3$ 206.0423; Found 206.0420 (− 1.45 ppm).

1-(2-aminobenzo[*d*]thiazol-6-yl)-3,3,3-trifluoro-2-hydroxypropan-1-one (2n) To a solution of **1n** (1.05 mmol, 1 eq) in AcOH

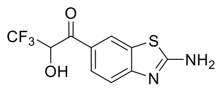

Chemical Formula: $C_{10}H_7F_3N_2O_2S$
Molecular Weight: 276,2332

(10 mL/mmol), Fe^0 (10.05 mmol, 10 eq) was added and the resulting suspension vigorously stirred for 2 h. Water (20 mL) and methanol (20 mL) were added, the resulting solution filtered and concentrated under reduced pressure. The oily residue was partitioned between water (200 mL) and ethyl acetate (3 x 200 mL). The combined organic layers were washed with saturated aqueous $NaHCO_3$ (100 mL), brine (100 mL), dried over Na_2SO_4, filtered and concentrated to get the title compound as light yellow solid (190 mg, 65.7%). The compound was used as a standard to determine full conversion of DKR-ATH. **^{1}H NMR** (400 MHz, DMSO-*d$_6$*) δ 8.42 (d, *J* = 1.8 Hz, 1H), 8.07 (s, 2H), 7.94 (dd, *J* = 8.5, 1.9 Hz, 1H), 7.40 (d, *J* = 8.5 Hz, 1H), 7.06 (d, *J* = 8.4 Hz, 1H), 5.70 (p, *J* = 7.5 Hz, 1H). **^{19}F NMR** (376 MHz, DMSO- *d$_6$*) δ -73.11 (d, *J* = 7.1 Hz). **^{13}C NMR** (101 MHz, Methanol-*d$_4$*) δ 192.95, 173.36, 158.58, 132.67, 129.46, 129.01, 124.90 (q, *J* = 283.1 Hz), 124.02, 118.33, 72.17 (q, *J* = 30.2 Hz).**HRMS** (ESI) m/z: $[M + H]^+$ Calcd for $C_{10}H_8O_2N_2F_3S$ 277.0253; Found 277.0253 (− 1.26 ppm).

3,3,3-trifluoro-1-(2-hydroxyphenyl)propane-1,2-dione (2o).[10] CAS: 121194-38-5. Prepared from 2-hydroxy-3-phenylpropanoic acid (2.50 g, 15.0 mmol) according to *General procedure for synthesis of CF_3 substituted α-hydroxyketones.* The crude product was distilled using Kugel-Rohr apparatus (80 °C, 0.01–0.02 mbar) then triturated with hexane and cyclohexane to get the title compound as light brown solid (1.10 g, 34% yield). **^{1}H NMR** (400 MHz, Chloroform-*d*) δ 7.45–7.29 (m, 3H), 7.24–7.11 (m, 2H), 4.61 (q, *J* = 7.5 Hz, 1H), 3.99 (dd, *J* = 42.3, 16.2 Hz, 3H). **^{19}F NMR** (376 MHz, Chloroform-*d*) δ −73.65 (d, *J* = 7.3 Hz). **^{13}C NMR** (101 MHz, Chloroform-*d*) δ 200.68, 131.57, 130.61, 129.65, 129.23, 128.89, 128.02, 122.59 (q, *J* = 283.5 Hz), 73.91 (q, *J* = 31.6 Hz), 46.54 (q, *J* = 2.5 Hz). **HRMS** (ESI) m/z: $[M - H]^-$ Calcd for $C_{10}H_8O_2F_3$ 217.0482; Found 217.0474 (− 3.49 ppm).

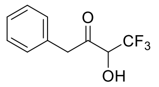
Chemical Formula: $C_{10}H_9F_3O_2$
Molecular Weight: 218,18

1,1,1-trifluoro-2-hydroxynonan-3-one (2p).[10] CAS: 156765-97-8. Prepared from 2-hydroxyoctanoic acid (2.50 g, 15.6 mmol) according to *General procedure for synthesis of CF_3 substituted α-hydroxyketones.* The crude product was purified by flash column chromatography, eluent ethyl acetate/hexane 1:6 then distilled using Kugel-Rohr apparatus (250 °C, 0.01–0.02 mbar) to get the title compound as colorless oil (1.56 g, 47% yield). **^{1}H NMR** (400 MHz, Chloroform-*d*) δ 4.60–4.36 (m, 1H), 4.04 (d, *J* = 6.3 Hz, 1H), 2.87–2.52 (m, 2H), 1.84–1.63 (m, 2H), 1.48–1.21 (m, 6H), 0.89 (t, *J* = 6.8 Hz, 3H). **^{19}F NMR** (376 MHz, Chloroform-*d*) δ −74.11 (d, *J* = 7.9 Hz). **^{13}C NMR** (101 MHz, Chloroform-*d*) δ 203.15, 122.61 (q, *J* = 283.2 Hz), 74.90 (q, *J* = 31.6 Hz), 39.82 (q, *J* = 2.2 Hz), 31.54, 28.70, 23.36, 22.54, 14.09. **HRMS** (ESI) m/z: $[M - H]^-$ Calcd for $C_9H_{14}O_2F_3$ 211.09514; Found 211.09443 (− 3.35 ppm).

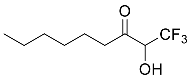
Chemical Formula: $C_9H_{15}F_3O_2$
Molecular Weight: 212,21

***N*-(6,6,6-Trifluoro-5-hydroxy-4-oxohexyl)benzamide (2q).**[13] CAS: 154918-77-1. Trifluoroacetic anhydride (4.1 mL, 29 mmol, 3 eq) was added dropwise to a refluxing solution of 1-benzoylpyrrolidine-3-carboxylic acid (2.11 g, 9.63 mmol) and pyridine (4.65 mL, 57.8 mmol, 6 eq) in dry benzene (50 mL, 5 mL/mmol) at 100 °C and the mixture was refluxed for 1 h. Then 5% HCl (2.5 mL/mmol) was added and the reaction mixture was stirred at 60 °C for 30 min. The reaction mixture was partitioned between ethyl acetate and water. The organic layer was washed with water, 1 M HCl, saturated aqueous $NaHCO_3$, brine, dried over Na_2SO_4, filtered and concentrated to get the crude product. The crude product was purified by flash column chromatography, eluent ethyl acetate/hexane 1:4 gradient to 1:1 to get the title compound as yellow oil (304 mg, 11% yield). **^{1}H NMR** (400 MHz, Chloroform-*d*) δ 7.84–7.66 (m, 2H), 7.58–7.37 (m, 3H), 6.35 (s, 1H), 4.65–4.46 (m, 1H), 4.22 (d, *J* = 6.0 Hz, 1H), 3.59–3.39 (m, 2H), 2.80 (qt, *J* = 18.6, 6.8 Hz, 2H), 2.18–1.90 (m, 2H). **^{19}F NMR** (376 MHz, Chloroform-*d*) δ −74.17 (d, *J* = 7.3 Hz). **^{13}C NMR** (101 MHz, Methanol-*d_4*) δ 205.77, 170.40, 135.64, 132.59, 129.51, 128.19, 124.65 (q, *J* = 282.9 Hz), 75.83 (q, *J* = 29.6 Hz), 40.07, 37.44 (q, *J* = 1.4 Hz), 23.96. **HRMS** (ESI) m/z: $[M + H]^+$ calcd. for $C_{13}H_{15}O_3NF_3$ 290.0998, found 290.0992 (− 2.32 ppm).

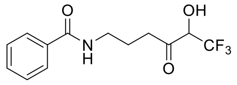
Chemical Formula: $C_{13}H_{14}F_3NO_3$
Molecular Weight: 289,2542

(*E*)-5,5,5-Trifluoro-4-hydroxy-1-phenylpent-1-en-3-one (2r). CAS: 1642331-44-9. To a suspension of cinammaldehyde (661 mg, 5.0 mmol), trifluoroacetaldehyde ethyl hemiacetal (1.2 mL, 10.0 mmol) and 2-phenyl-6,7-dihydro-5*H*-pyrrolo[2,1-*c*][1,2,4]triazol-2-ium tetrafluoroborate (137 mg, 0.5 mmol) in THF (35 mL) was added 1,8-diazabicyclo[5.4.0]undec-7-ene (0.220 mL, 1.5 mmol) and the reaction mixture was stirred at 22 °C, monitored by NMR. After 3 h, ^{1}H NMR analysis revealed 93% conversion of the aldehyde, and after 4 h the reaction mixture was concentrated under reduced pressure. The oily residue was partitioned between EtOAc (150 mL) and 1 M HCl (150 mL), the organic layer washed with brine (150 mL), dried over Na_2SO_4 and concentrated. The crude product was purified by flash chromatography on silica, eluent hexane/EtOAc 9:1 to get the title compound as a pale yellow solid (482 mg, 42% yield). **^{1}H NMR** (400 MHz, Chloroform-*d*) δ 7.91 (d, *J* = 15.8 Hz, 1H), 7.66–7.60 (m, 2H), 7.52–7.42 (m, 3H), 6.99 (dq, *J* = 15.9, 1.1 Hz, 1H), 4.86–4.71 (m, 1H), 4.24 (d, *J* = 6.7 Hz, 1H). **^{19}F NMR** (376 MHz, Chloroform-*d*) δ −74.07 (d, *J* = 7.3 Hz). NMR data is in accordance with the literature data.[14] Note: prolonged reaction times result in base-catalyzed detrifluoromethylation.

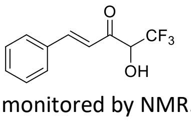

4 Synthesis of *syn*- and *anti*-CF_3-substituted-1,2-diols 3a–3r

General procedure for DKR-ATH. The ruthenium catalyst (*S,S*)-**C4** (3.10 mg for S/C = 100, 1.55 mg for S/C = 200, 0.62 mg for S/C = 500, 0.31mg for S/C = 1000) or (*S,S*)-**C5**. (3.25 mg for S/C = 100, 1.625 mg for S/C = 200, 0.65 mg for S/C = 500, 0.325 mg for S/C = 1000) was treated with HCO_2H/Et_3N 3:2 (0.5 mL) and stirred at r.t. for 30 min under a light stream of argon. A solution of diketone **3** (0.5 mmol) or α-hydroxyketone **2** (0.5 mmol) in DMF (1 mL) was then added and the resulting solution was stirred at 60 °C with continued argon sweeping. The reaction was monitored by NMR analysis and after full conversion, the reaction mixture was concentrated under reduced pressure and partitioned between ethyl acetate (10 mL) and water (10 mL). The organic layer was washed with brine, dried over Na_2SO_4, filtered through a short pad of silica, and concentrated to get the crude product **3a**–**3q**. This was optionally purified by flash chromatography on silica or crystallization/trituration to get single isomer.

General procedure for $NaBH_4$ reduction. To a solution of diketone **1** or α-hydroxyketone **2** in absolute ethanol (1 mL/mmol) at 0 °C was added portionwise $NaBH_4$ (10 eq). The resulting mixture was stirred at 22 °C for 2 h, then it was quenched with saturated NH_4Cl and partitioned between ethyl acetate (10 mL) and water (10 mL). The organic layer was washed with brine, dried over Na_2SO_4 and concentrated to get the crude racemic *anti*-**3a**–**3q**. This was used as a standard for determination of diastereomeric (*syn*/*anti*) ratio by non-decoupled ^{19}F NMR analysis, and enantiomeric ratio by chiral GC or HPLC analysis. Crude products were optionally purified by flash chromatography or crystallization/trituration to get diastereomerically pure (±)-*anti*-**3**

***syn*-3a (1*R*,2*S*)-3,3,3-trifluoro-1-phenylpropane-1,2-diol**.[15] Prepared from **1a** (110 mg, 0.5 mmol) according to the *General procedure for DKR-ATH* using (*S,S*)-**C4**, S/C = 1000. Full conversion after 2 h (87.6 mg, 85% isol. crude yield); *syn*/*anti* = 96:4; ee (*syn*) >99%. The crude product was purified by flash column chromatography, eluent dichlorometane/methanol 30:1 to get the stereomerically pure compound as colourless oil.

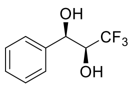

Chemical Formula: $C_9H_9F_3O_2$
Molecular Weight: 206,16

Prepared from **1a** (110 mg, 0.5 mmol) according to the *General procedure for DKR-ATH* using (*S,S*)-**C5**., S/C = 1000. Full conversion after 2 h (87.6 mg, 85% isol. crude yield); *syn*/*anti* = 97:3; ee (*syn*) >99%.

^1H NMR (400 MHz, Chloroform-*d*) δ 7.62–7.29 (m, 5H), 4.99 (t, *J* = 3.1 Hz, 1H), 4.02 (td, *J* = 7.2, 2.8 Hz, 1H), 3.35 (d, *J* = 7.7 Hz, 1H), 2.65 (d, *J* = 3.6 Hz, 1H). **^{19}F NMR** (376 MHz, Chloroform-*d*) δ −76.76 (d, *J* = 6.9 Hz). **^{13}C NMR** (101 MHz, Chloroform-*d*) δ 139.54, 128.91, 128.82, 126.39, 124.46 (q, *J* = 283.3 Hz), 73.66 (q, *J* = 29.5 Hz), 70.64 (q, *J* = 2 Hz). **HRMS** (ESI) m/z: $[M - H]^-$ Calcd for $C_9H_8O_2F_3$ 205.04819; Found 205.04735 (− 4.08 ppm).

(±)-*anti*-3a (±)-*anti* 3,3,3-trifluoro-1-phenylpropane-1,2-diol.[15] Prepared from **1a** (110 mg, 0.5 mmol) according to the *General procedure for $NaBH_4$ reduction* to get (±)-*anti*-**3a** as white solid (83% isol. crude yield); *anti*/*syn* = 97:3. **^1H NMR** (400 MHz, Chloroform-*d*) δ 7.47–7.36 (m, 5H), 4.96 (dd, *J* = 6.1, 4.0 Hz, 1H), 4.28–4.14 (m, 1H), 2.38 (d, *J* = 6.1 Hz, 1H), 2.35 (d, *J* = 4.0 Hz, 1H). **^{19}F NMR** (376 MHz, Methanol-*d₄*) δ −76.33 (d, *J* = 6.8 Hz). **^{13}C NMR** (101 MHz, Methanol-d_4) δ 142.59, 129.07, 128.86, 128.66, 126.70 (q, *J* = 283 Hz), 73.95 (q, *J* = 28.2 Hz), 73.88 (q, *J* = 1 Hz). **HRMS** calcd. for $C_9H_8O_2F_3$ $[M - H]^-$ 205.04819, found 205.04737 (− 3.99 ppm).

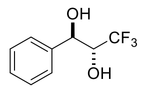

Chemical Formula: $C_9H_9F_3O_2$
Molecular Weight: 206,16

***syn*-3b (1*R*,2*S*)-1-(4-bromophenyl)-3,3,3-trifluoropropane-1,2-diol.** Prepared from **1b** (149 mg, 0.5 mmol) according to the *General procedure for DKR-ATH* using (*S,S*)-**C4**, S/C = 1000. Full conversion after 2 h (122 mg, 86% isol. crude yield); *syn*/*anti* = 96:4; ee (*syn*) >99%. The crude product was purified by flash column chromatography, eluent dichlorometane/methanol 30:1 to get the stereomerically pure compound as white solid.

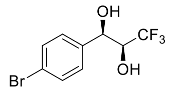

Chemical Formula: $C_9H_8BrF_3O_2$
Molecular Weight: 285,0602

Prepared from **1b** (149 mg, 0.5 mmol) according to the *General procedure for DKR-ATH* using (*S,S*)-**C5**, S/C = 500. Full conversion after 18 h (113 mg, 85% isol. crude yield). *syn*/*anti* = 97:3; ee (*syn*) >99%.

^1H NMR (400 MHz, Chloroform-*d*) δ 7.51 (d, *J* = 8.5 Hz, 2H), 7.25 (d, *J* = 8.3 Hz, 2H), 4.95 (s, 1H), 4.03–3.86 (m, 1H), 3.33 (d, *J* = 8.1 Hz, 1H), 2.75 (s, 1H). **^{19}F NMR** (376 MHz, Methanol-d_4) δ −76.92 (d, *J* = 7.0 Hz). **^{13}C NMR** (101 MHz, Methanol-d_4) δ 141.89, 132.26, 129.85, 126.28 (q, *J* = 283.1 Hz),122.46, 74.48 (q, *J* = 28.7 Hz), 71.84 (q, *J* = 2 Hz). **HRMS** (ESI) m/z: $[M - H]^-$ Calcd for $C_9H_7O_2BrF_3$ 282.9587; Found 282.95848 (− 0.78 ppm).

(±)-*anti*-3b (±)-*anti*-1-(4-bromophenyl)-3,3,3-trifluoropropane-1,2-diol. Prepared from **1b** (149 mg, 0.5 mmol) according to the *General procedure for $NaBH_4$ reduction* (99.8 mg, 70% isol. crude yield); *anti*/*syn* = 86:14. The crude product was purified by flash column chromatography, eluent dichlorometane/methanol 30/1 to get (±)-*anti*-**3b** as white solid.

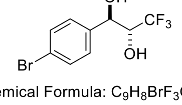

Chemical Formula: $C_9H_8BrF_3O_2$
Molecular Weight: 285,06

^{1}H NMR (400 MHz, Chloroform-*d*) δ 7.53 (d, *J* = 8.4 Hz, 2H), 7.31 (d, *J* = 8.4 Hz, 2H), 4.93 (dd, *J* = 5.7, 4.4 Hz, 1H), 4.28 – 4.11 (m, 1H), 2.35 (d, *J* = 6.0 Hz, 1H), 2.32 (d, *J* = 4.1 Hz, 1H). **^{19}F NMR** (376 MHz, Methanol-*d₄*) δ −76.44 (d, *J* = 6.9 Hz). **^{13}C NMR** (101 MHz, Methanol-*d₄*) δ 141.99, 132.08, 130.63, 126.59 (q, *J* = 282.9 Hz), 122.56, 73.89 (q, *J* = 28.2 Hz), 73.15 (q, *J* = 1 Hz). **HRMS** (ESI) m/z: [M − H]$^-$ Calcd for $C_9H_7O_2BrF_3$ 282.9587; found 282.9585 (− 0.85 ppm).

***syn*-3c (1*R*,2*S*)-1-(4-chlorophenyl)-3,3,3-trifluoropropane-1,2-diol.** Prepared from **2c** (119 mg, 0.5 mmol) according to the *General procedure for DKR-ATH* using (*S*,*S*)-**C4**, S/C = 500, to get *syn*-**3c**. Full conversion after 2 h (108 mg, 90% isol. yield); *syn*/*anti* = 95:5; ee (*syn*) = 97%. The crude product was purified by preparative thin layer chromatography, eluent dichloromethane/methanol 30:1 followed by crystallization to get syn-3c as white crystals, *syn*/*anti* = 97:3.

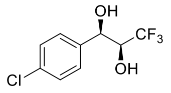

Chemical Formula: $C_9H_8ClF_3O_2$
Molecular Weight: 240,6062

Prepared from **2c** (119 mg, 0.5 mmol) according to the *General procedure for DKR-ATH* using (*S*,*S*)-**C5**, S/C = 500. Full conversion after 18 h (97.5 mg, 81% isol. crude yield); *syn*/*anti* = 96:4; ee (*syn*) >99%**.**

^{1}H NMR (400 MHz, Chloroform-*d*) δ 7.39–7.32 (m, 4H), 5.01 (t, *J* = 3.5 Hz, 1H), 4.01 (pd, *J* = 7.0, 3.0 Hz, 1H), 3.20 (d, *J* = 8.2 Hz, 1H), 2.58 (d, *J* = 4.0 Hz, 1H). **^{19}F NMR** (376 MHz, Methanol-*d₄*) δ −76.91 (d, *J* = 7.8 Hz). **^{13}C NMR** (101 MHz, Methanol-*d₄*) δ 141.38, 134.48, 129.52, 129.24, 126.28 (q, *J* = 283.0 Hz), 74.54 (q, *J* = 28.6 Hz), 71.80 (q, *J* = 2 Hz). **HRMS** (ESI) m/z: [M − H]$^-$ Calcd for $C_9H_7O_2ClF_3$ 239.0092; Found 239.0088 (− 1.90 ppm).

(±)-*anti*-3c (±)-*anti*-1-(4-chlorophenyl)-3,3,3-trifluoropropane-1,2-diol. Prepared from **2c** (119 mg, 0.5 mmol) according to the *General procedure for NaBH₄ reduction* (102 mg, 85% isol. crude yield); *anti*/*syn* = 89:11. The crude product was purified by preparative thin layer chromatography, eluent dichloromethane/methanol 30:1 followed by trituration with chloroform to get (±)-*anti*-**3c** as white solid.

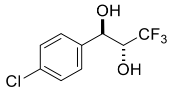

Chemical Formula: $C_9H_8ClF_3O_2$
Molecular Weight: 240,61

^{1}H NMR (400 MHz, Chloroform-*d*) δ 7.38 (s, 4H), 4.95 (dd, *J* = 5.9, 3.9 Hz, 1H), 4.19 (dd, *J* = 11.7, 6.1 Hz, 1H), 2.38 (d, *J* = 4.9 Hz, 1H), 2.33 (d, *J* = 4.0 Hz, 1H). **^{19}F NMR** (376 MHz, Methanol-*d₄*) δ −76.45 (d, *J* = 7.1 Hz). **^{13}C NMR** (101 MHz, Methanol-*d₄*) δ 141.52, 134.54, 130.30, 129.06, 126.60 (q, *J* = 282.8 Hz), 73.94 (q, *J* = 28.3 Hz), 73.10 (q, *J* = 2 Hz). **HRMS** (ESI) m/z: [M − H]$^-$ Calcd for $C_9H_7O_2ClF_3$ 239.0092; Found 239.0087 (− 2.11 ppm).

***syn*-3d (1*R*,2*S*)-3,3,3-trifluoro-1-(4-fluorophenyl)propane-1,2-diol.** Prepared from **2d** (111 mg, 0.5 mmol) according to the *General procedure for DKR-ATH* using (*S*,*S*)-**C4**, S/C = 500. Full conversion after 2 h (105 mg, 94% isol. crude yield); *syn*/*anti* = 95:5; ee (*syn*) = 98%. The crude product was purified by flash column chromatography, eluent dichloromethane/methanol 30:1 to get the diastereomerically pure compound as colorless oil.

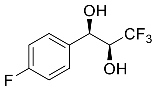

Chemical Formula: $C_9H_8F_4O_2$
Molecular Weight: 224,1546

Prepared from **2d** (111 mg, 0.5 mmol) according to the *General procedure for DKR-ATH* using (*S*,*S*)-**C5**, S/C = 500. Full conversion after 1 h (97.5 mg, 87% isol. crude yield); *syn*/*anti* = 96:4; ee (*syn*) >99%**.**

^{1}H NMR (400 MHz, Chloroform-*d*) δ 7.38–7.30 (m, 2H), 7.11–6.96 (m, 2H), 4.93 (s, 1H), 3.96 (s, 1H), 3.66 (s, 1H), 3.08 (s, 1H). **^{19}F NMR** (376 MHz, Chloroform-*d*) δ −76.49 (d, *J* = 7.3 Hz), (−113.08)–(−113.21) (m). **^{13}C NMR** (101 MHz, Chloroform-*d*) δ 162.76 (d, *J* = 247.3 Hz), 135.21 (d, *J* = 3.1 Hz), 128.28 (d, *J* = 8.2 Hz, 2C), 124.31 (q, *J* = 283.2 Hz), 115.76 (d, *J* = 21.6 Hz, 2C), 73.65 (q, *J* = 29.5 Hz), 70.19 (q, *J* = 2 Hz). **HRMS** (ESI) m/z: [M − H]$^-$ Calcd. for $C_9H_7O_2F_4$ 223.0388; Found 223.0381 (− 3.07 ppm).

(±)-*anti*-3d (±)-*anti* 3,3,3-trifluoro-1-(4-fluorophenyl)propane-1,2-diol Prepared from **2d** (111 mg, 0.5 mmol) according to the *General procedure for NaBH₄ reduction* (88.54 mg, 79% isol. crude yield); *anti*/*syn* = 91:9. The crude product was purified by flash column chromatography, eluent dichloromethane/methanol 30:1 to get (±)-*anti*-**3d** as colorless oil.

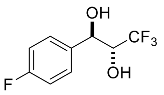

Chemical Formula: $C_9H_8F_4O_2$
Molecular Weight: 224,15

^{1}H NMR (400 MHz, Chloroform-*d*) δ 7.45–7.35 (m, 2H), 7.15–6.99 (m, 2H), 4.93 (dd, *J* = 5.7, 3.9 Hz, 1H), 4.24–4.08 (m, 1H), 2.58 (d, *J* = 4.9 Hz, 1H), 2.46 (d, *J* = 3.8 Hz, 1H). **^{19}F NMR** (376 MHz, Chloroform-*d*) δ −74.79 (d, *J* = 6.8 Hz), −112.86 (dd, *J* = 8.8, 5.0 Hz). **^{13}C NMR** (101 MHz, Chloroform-*d*) δ 162.91 (d, *J* = 247.4), 134.38 (d, *J* = 3.1 Hz), 129.09 (d, *J* = 8.3 Hz, 2C), 124.37 (q, *J* = 282.7 Hz), 115.69 (d, *J* = 21.6 Hz, 2C), 73.37 (q, *J* = 29.1 Hz), 72.17 (q, *J* = 1 Hz). **HRMS** (ESI) m/z: [M − H]$^-$ Calcd for $C_9H_7O_2F_4$ 223.0388; Found 223.0381 (− 2.85 ppm).

***syn*-3e (1*R*,2*S*)-3,3,3-trifluoro-1-(4-nitrophenyl)propane-1,2-diol.** Prepared from **1e** (1.00 g, 3.77 mmol) according to the *General procedure for DKR-ATH* using (*S*,*S*)-**C4**, S/C = 1000. For a gram-scale reaction it is important to bubble argon through the reaction mixture in order to expell CO_2. Full conversion after 18 h; *syn*/*anti* = 95:5; ee (*syn*) >99%. The crude product was purified by flash column chromatography, eluent dichlorometane/methanol gradient from 40:1 to 30:1 to get the stereomerically pure

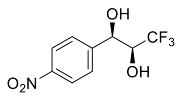

Chemical Formula: $C_9H_8F_3NO_4$
Molecular Weight: 251,1612

compound as light brown solid (0.52 g, 55% isol. yield). A crystal suitable for single-crystal X-ray analysis was grown by slow evaporation of hexane/dichloromethane solution.

Prepared from **1e** (124 mg, 0.5 mmol) according to the *General procedure for DKR-ATH*. using (*S,S*)-**C5**, S/C = 500. Full conversion after 18 h (89.2 mg, 71% isol. crude yield). *syn*/*anti* = 94:6; ee (*syn*) >99%

^{1}**H NMR** (400 MHz, Methanol-d_4) δ 8.23 (d, *J* = 8.9 Hz, 2H), 7.70 (d, *J* = 8.4 Hz, 2H), 5.06 (d, *J* = 3.0 Hz, 1H), 4.10 (qd, *J* = 7.2, 2.5 Hz, 1H). ^{19}**F NMR** (376 MHz, Methanol-d_4) δ −76.87 (d, *J* = 6.9 Hz). ^{13}**C NMR** (101 MHz, Methanol-d_4) δ 150.24, 148.87, 128.95 (2C), 126.24 (q, *J* = 283 Hz), 124.17 (2C), 74.29 (q, *J* = 29.0 Hz), 71.69 (q, *J* = 2 Hz). **HRMS** (ESI) m/z: [M − H]$^-$ Calcd for $C_9H_7O_4NF_3$ 250.0333; Found 250.0329 (− 1.38 ppm).

(±)-*anti*-3e (±)-*anti* 3,3,3-trifluoro-1-(4-nitrophenyl)propane-1,2-diol. Prepared from **1e** (247 mg, 1 mmol) according to the *General procedure for NaBH₄ reduction*. *anti*/*syn* = 67:33 (183.34 mg, 73% isol. crude yield). The crude product was purified by flash column chromatography, eluent dichlorometane/methanol gradiently from 40/1 to 30/1 to get (±)-*anti*-**3e** as brown solid.

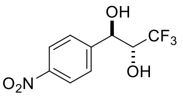

Chemical Formula: $C_9H_8F_3NO_4$
Molecular Weight: 251,16

^{1}**H NMR** (400 MHz, Methanol- d_4) δ 8.22 (d, *J* = 8.2 Hz, 2H), 7.67 (d, *J* = 8.3 Hz, 2H), 4.88 (s, 1H), 4.05 (p, *J* = 7.1 Hz, 1H) ^{19}**F NMR** (376 MHz, Methanol-d_4) δ −74.54 (d, *J* = 6.8 Hz). ^{13}**C NMR** (101 MHz, Methanol-d_4) δ 150.25, 148.96, 129.82, 126.49 (q, *J* = 282.8 Hz), 123.96, 73.94 (q, *J* = 28.5 Hz), 72.91 (q, *J* = 1 Hz). **HRMS** (ESI) m/z: [M − H]$^-$ Calcd for $C_9H_7O_4NF_3$ 250.0333; Found 250.0329 (− 1.38 ppm).

***syn*-3f (1*R*,2S)-3,3,3-trifluoro-1-(3-hydroxy-4-nitrophenyl)propane-1,2-diol.** Prepared from **1f** (141 mg, 0.5 mmol) according to the *General procedure for DKR-ATH* using (*S,S*)-**C4**, S/C = 1000. Full conversion after 2 h (104 mg, 78% isol. crude yield); *syn*/*anti* = 96:4; ee (*syn*) >99%. The crude product was purified by preparative thin layer chromatography, eluent dichloromethane/methanol 17:1 to get the stereomerically pure compound as beige solid.

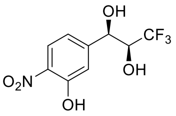

Chemical Formula: $C_9H_8F_3NO_5$
Molecular Weight: 267,1602

Prepared from **1f** (1401 mg, 0.5 mmol) according to the *General procedure for DKR-ATH* using (*S,S*)-**C5**-cat., S/C = 500. Full conversion after 18 h (101 mg, 76% isol. crude yield); *syn*/*anti* = 96:4; ee (*syn*) >99%

^{1}**H NMR** (400 MHz, DMSO-d_6) δ 10.90 (s, 1H), 7.86 (d, *J* = 8.6 Hz, 1H), 7.22 (d, *J* = 1.3 Hz, 1H), 7.03 (dd, *J* = 8.6, 1.5 Hz, 1H), 6.24 (d, *J* = 8.0 Hz, 1H), 5.87 (d, *J* = 5.9 Hz, 1H), 4.83 (dd, *J* = 5.9, 3.0 Hz, 1H), 4.14–4.05 (m, 1H). ^{19}**F NMR** (376 MHz, Methanol-d_4) δ −76.99 (d, *J* = 7.7 Hz). ^{13}**C NMR** (101 MHz, Methanol-d_4) δ 155.43, 152.96, 134.86, 126.21 (q, *J* = 283.3 Hz), 125.95, 119.45, 118.99, 74.09 (q, *J* = 29.1 Hz), 71.48 (q, *J* = 1 Hz). **HRMS** (ESI) m/z: [M − H]$^-$ Calcd for $C_9H_7O_5NF_3$ 266.0282; Found 266.0279 (− 1.02 ppm).

(±)-*anti*-3f (±)-*anti* 3,3,3-trifluoro-1-(3-hydroxy-4-nitrophenyl)propane-1,2-diol. Prepared from **1f** (140.6 mg, 0.5 mmol) according to the *General procedure for NaBH₄ reduction* (108.20 mg, 81% isol. crude yield). The crude product was purified by preparative thin layer chromatography, eluent dichloromethane/methanol 17/1 to get (±)-*anti*-**3f** as light brown solid. *anti*/*syn* = 96:4

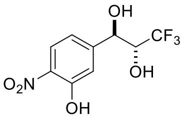

Chemical Formula: $C_9H_8F_3NO_5$
Molecular Weight: 267,16

^{1}**H NMR** (400 MHz, DMSO-d_6) δ 10.91 (s, 1H), 7.85 (d, *J* = 8.5 Hz, 1H), 7.16 (d, *J* = 1.5 Hz, 1H), 7.01 (dd, *J* = 8.6, 1.5 Hz, 1H), 6.38 (d, *J* = 7.1 Hz, 1H), 5.98 (d, *J* = 5.1 Hz, 1H), 4.60 (dd, *J* = 7.6, 5.2 Hz, 1H), 3.93 (h, *J* = 7.3 Hz, 1H). ^{19}**F NMR** (376 MHz, Methanol-d_4) δ −76.42 (d, *J* = 6.8 Hz). ^{13}**C NMR** (101 MHz, Methanol-d_4) δ 155.23, 152.84, 134.97, 126.44 (q, *J* = 283.0 Hz), 125.67, 120.44, 119.77, 73.78 (q, *J* = 28.5 Hz), 72.84 (q, *J* = 1 Hz). **HRMS** (ESI) m/z: [M − H]$^-$ Calcd for $C_9H_7O_5NF_3$ 266.0282; Found 266.0279 (− 0.94 ppm).

***syn*-3g (1*R*,2*S*)-3,3,3-trifluoro-1-(4-hydroxyphenyl)propane-1,2-diol.** Prepared from **2g** (110 mg, 0.5 mmol) according to the *General procedure for DKR-ATH* using (*S,S*)-**C4**, S/C = 500. Full conversion after 3 h (90.0 mg, 81% isol. crude yield); *syn*/*anti* = 84:16; ee (*syn*) ≥99%. The crude product was purified by flash column chromatography, eluent dichloromethane/methanol 20:1 to get the stereomerically pure compound as white solid.

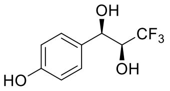

Chemical Formula: $C_9H_9F_3O_3$
Molecular Weight: 222,1632

Prepared from **2g** (111 mg, 0.5 mmol) according to the *General procedure for DKR-ATH* using (*S,S*)-**C5**, S/C = 100. Conversion after 18 h: 88% (96.6 mg, 87% isol. crude yield). *syn*/*anti* = 92:8; ee (*syn*) >99%

^{1}**H NMR** (400 MHz, Chloroform-*d*) δ 7.30 (d, *J* = 8.4 Hz, 2H), 6.85 (d, *J* = 8.7 Hz, 2H), 4.96 (t, *J* = 3.5 Hz, 1H), 4.89 (s, 1H), 4.07–3.97 (m, 1H), 3.21 (d, *J* = 8.0 Hz, 1H), 2.39 (d, *J* = 3.8 Hz, 1H). ^{19}**F NMR** (376 MHz, Methanol-d_4) δ −76.43 (d, *J* = 6.9 Hz). ^{13}**C NMR** (101 MHz, Methanol-d_4) δ 141.97, 132.07 (2C), 130.62 (2C), 126.57 (q, *J* = 283.0 Hz), 122.56, 73.88 (q, *J* = 28.4 Hz), 73.14 (q, *J* = 2 Hz). **HRMS** (ESI) m/z: [M − H]$^-$ Calcd for $C_9H_8O_3F_3$ 221.0431; Found 221.0425 (− 2.81 ppm).

(±)-*anti*-3g (±)-*anti* 3,3,3-trifluoro-1-(4-hydroxyphenyl)propane-1,2-diol. Prepared from **2g** (110 mg, 0.5 mmol) according to the *General procedure for NaBH4 reduction* (57.8 mg, 52% isol. crude yield); *anti*/*syn* = 89:11. The crude product was purified by flash column chromatography, eluent dichloromethane/methanol 20:1 to get (±)-*anti*-**3g** as white solid.

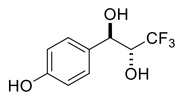

Chemical Formula: $C_9H_9F_3O_3$
Molecular Weight: 222,16

^{1}H NMR (400 MHz, Methanol-*d_4*) δ 7.23 (d, *J* = 8.6 Hz, 2H), 6.76 (d, *J* = 8.6 Hz, 2H), 4.62 (d, *J* = 7.8 Hz, 1H), 3.99 (p, *J* = 7.2 Hz, 1H). **^{19}F NMR** (376 MHz, Methanol-*d_4*) δ −76.38 (d, *J* = 6.9 Hz). **^{13}C NMR** (101 MHz, Methanol-*d_4*) δ 158.25, 133.42, 129.85 (2C), 126.75 (q, *J* = 283 Hz), 115.82 (2C), 73.95 (q, *J* = 28.1 Hz), 73.53. **HRMS** (ESI) m/z: [M − H]$^-$ Calcd for $C_9H_8O_3F_3$ 221.0431; Found 221.0424 (− 3.18 ppm).

***syn*-3h (1*R*,2*S*)-3,3,3-trifluoro-1-(3-hydroxyphenyl)propane-1,2-diol.** Prepared from **1h** (118 mg, 0.5 mmol) according to the *General procedure for DKR-ATH*. using (*S*,*S*)-**C4**, S/C = 500. Full conversion after 2 h (111 mg, 85% isol. crude yield); *syn*/*anti* = 93:7; ee (*syn*) >99%. The crude product was purified by flash column chromatography, eluent dichloromethane/methanol 20:1 to get the stereomerically pure compound as colorless oil.

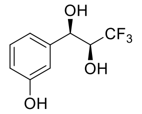

Chemical Formula: $C_9H_9F_3O_3$
Molecular Weight: 222,1632

Prepared from **1h** (118 mg, 0.5 mmol) according to the *General procedure for DKR-ATH* using (*S*,*S*)-**C5**, S/C = 200. Full conversion after 18 h (93.3 mg, 84% isol. crude yield); *syn*/*anti* = 96:4; ee (*syn*) >99%.

^{1}H NMR (400 MHz, Methanol-*d_4)* δ 7.05 (t, *J* = 7.8 Hz, 1H), 6.84–6.71 (m, *J* = 7.5, 5.1 Hz, 2H), 6.60 (ddd, *J* = 8.1, 2.4, 1.0 Hz, 1H), 4.69 (d, *J* = 3.7 Hz, 1H), 3.89 (qd, *J* = 7.4, 3.7 Hz, 1H). **^{19}F NMR** (376 MHz, Methanol-*d_4*) δ −77.13 (d, *J* = 6.9 Hz). **^{13}C NMR** (101 MHz, Methanol-*d_4*) δ 158.45, 144.05, 130.24, 126.33 (q, *J* = 283.1 Hz), 118.90, 115.66, 114.73, 74.72 (q, *J* = 28.5 Hz), 72.28 (q, *J* = 2 Hz). **HRMS** (ESI) m/z: [M − H]$^-$ Calcd for $C_9H_8O_3F_3$ 221.0431; Found 221.0424 (− 3.13 ppm).

(±)-*anti*-3h (±)-*anti* 3,3,3-trifluoro-1-(3-hydroxyphenyl)propane-1,2-diol. Prepared from 1h (46 mg, 0.21 mmol) according to the *General procedure for NaBH4 reduction* (36.85 mg, 79% isol. crude yield). The crude product was purified by flash column chromatography, eluent dichloromethane/methanol 20:1 to get (±)-*anti*-**3h** as colorless oil; *anti*/*syn* = 93:7.

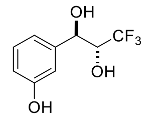

Chemical Formula: $C_9H_9F_3O_3$
Molecular Weight: 222,16

^{1}H NMR (400 MHz, $CDCl_3$) δ 7.29 (d, *J* = 7.9 Hz, 1H), 6.99 (d, *J* = 7.6 Hz, 1H), 6.95–6.91 (m, 1H), 6.84 (ddd, *J* = 8.1, 2.5, 0.8 Hz, 1H), 4.95 (s, 1H), 4.91 (dd, *J* = 6.1, 3.2 Hz, 1H), 4.18 (dd, *J* = 11.8, 6.0 Hz, 1H), 2.33 (s, 1H), 2.31 (d, *J* = 3.9 Hz, 1H). **^{19}F NMR** (376 MHz, Methanol-*d_4*) δ −76.33 (d, *J* = 6.8 Hz). **^{13}C NMR** (101 MHz, Methanol-*d_4*) δ 158.29, 144.16, 130.07, 126.71 (q, *J* = 282.9 Hz), 119.85, 115.76, 115.51, 73.90 (q, *J* = 28.2 Hz), 73.88 (q, *J* = 1 Hz). **HRMS** (ESI) m/z: [M − H]$^-$ Calcd for $C_9H_8O_3F_3$ 221.0431; Found 221.0425 (− 2.81 ppm).

***syn*-3i (1*R*,2*S*)-3,3,3-trifluoro-1-(2-hydroxyphenyl)propane-1,2-diol.** Prepared from **1i** (109 mg, 0.5 mmol) according to the *General procedure for DKR-ATH* using (*S*,*S*)-**C4**, S/C = 100) to get *syn*-**3i**. Full conversion after 3.5 h (100 mg, 90% isol. crude yield); *syn*/*anti* = 87:13; ee (*syn*) >99%. The crude product was purified by recrystallization from chloroform to get the stereomerically pure compound as white crystals.

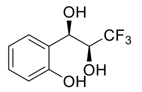

Chemical Formula: $C_9H_9F_3O_3$
Molecular Weight: 222,1632

Prepared from **1i** (109 mg, 0.5 mmol) according to the *General procedure for DKR-ATH* using (*S*,*S*)-**C5**, S/C = 100. Full conversion after 18 h (93.31 mg, 84% isol. crude yield); *syn*/*anti* = 69:31; ee (*syn*)>99 %

^{1}H NMR (400 MHz, Chloroform-*d*) δ 7.23 (dd, *J* = 7.7, 1.4 Hz, 1H), 7.20 (dd, *J* = 7.6, 1.4 Hz, 1H), 6.94 (td, *J* = 7.5, 1.0 Hz, 1H), 6.86 (dd, *J* = 8.1, 0.9 Hz, 1H), 6.64 (s, 1H), 5.16 (t, *J* = 4.3 Hz, 1H), 4.28 (dd, *J* = 10.9, 6.3 Hz, 1H), 3.22 (d, *J* = 6.0 Hz, 1H), 3.06 (d, *J* = 4.4 Hz, 1H). **^{19}F NMR** (376 MHz, Methanol-*d_4*) δ −77.41 (d, *J* = 8.1 Hz). **^{13}C NMR** (101 MHz, Methanol-*d_4*) δ 155.06, 129.40, 128.93, 128.46, 126.62 (q, *J* = 283.1 Hz), 120.25, 115.73, 72.54 (q, *J* = 28.6 Hz), 67.59 (q, *J* = 2 Hz). **HRMS** (ESI) m/z: [M − H]$^-$ Calcd for $C_9H_8O_3F_3$ 221.0431; Found 221.0423 (− 3.0 ppm).

(±)-*anti*-3i (±)-*anti* 3,3,3-trifluoro-1-(2-hydroxyphenyl)propane-1,2-diol. Prepared from **1i** (109 mg, 0.5 mmol) according to the *General procedure for NaBH4 reduction* (61.1 mg, 55% isol. crude yield); *anti*/*syn* = 91:9. The crude product was purified by flash column chromatography, eluent dichloromethane/methanol 20:1 to get (±)-*anti*-**3i** as white solid.

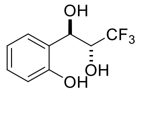

Chemical Formula: $C_9H_9F_3O_3$
Molecular Weight: 222,16

^{1}H NMR (400 MHz, Methanol-*d_4*) δ 7.30 (dd, *J* = 7.6, 1.5 Hz, 1H), 7.11 (td, *J* = 8.0, 1.7 Hz, 1H), 6.82 (td, *J* = 7.5, 1.0 Hz, 1H), 6.78 (dd, *J* = 8.1, 0.9 Hz, 1H), 5.05 (d, *J* = 7.1 Hz, 1H), 4.27 (p, *J* = 7.2 Hz, 1H). **^{19}F NMR** (376 MHz, Methanol-*d_4*) δ −76.01 (d, *J* = 7.5 Hz). **^{13}C NMR** (101 MHz, Methanol-*d_4*) δ 156.30, 129.82, 129.77, 127.85, 126.77 (q, *J* = 283 Hz), 120.41, 116.39, 72.92 (q, *J* = 28.2 Hz), 70.81. **HRMS** (ESI) m/z: [M − H]$^-$ Calcd for $C_9H_8O_3F_3$ 221.0431; Found 221.0424 (−3.18 ppm).

***syn*-3j (1*R*,2*S*)-3,3,3-trifluoro-1-(4-methoxyphenyl)propane-1,2-diol.** Prepared from **1j** (125 mg, 0.5 mmol) according to the *General procedure for DKR-ATH* ((*S*,*S*)-**C4**-cat., S/C = 1000) to get *syn*-**3j**. Full conversion after 18 h (102.74 mg, 87% isol. crude yield). The crude product was purified by flash column chromatography, eluent dichlorometane/methanol 30/1 to get the stereomeric pure compound as white solid. *syn*/*anti* = 95:5; ee *syn* = 98%

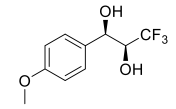

Chemical Formula: $C_{10}H_{11}F_3O_3$
Molecular Weight: 236,1902

Prepared from **1j** (41.5 mg, 0.18 mmol) according to the *General procedure for DKR-ATH* using (*S*,*S*)-**C5**, S/C = 100). Full conversion after 18 h (32.3 mg, 76% isol. crude yield); *syn*/*anti* = 96:4; ee (*syn*)>99%.

^{1}H NMR (400 MHz, Methanol-*d₄*) δ 7.33 (d, *J* = 8.5 Hz, 2H), 6.90 (d, *J* = 8.8 Hz, 2H), 4.80 (d, *J* = 4.2 Hz, 1H), 3.98 (qd, *J* = 7.4, 4.3 Hz, 1H), 3.78 (s, 3H). **^{19}F NMR** (376 MHz, Methanol-d_4) δ −76.88 (d, *J* = 6.9 Hz). **^{13}C NMR** (101 MHz, Methanol-d_4) δ 160.85Z, 134.37, 129.12, 126.33 (q, *J* = 283.1 Hz), 114.58, 74.80 (q, *J* = 28.1 Hz), 72.19 (q, *J* = 2 Hz), 55.66. **HRMS** (ESI) m/z: $[M-H]^-$ Calcd for $C_{10}H_{10}O_3F_3$ 235.0588; Found 235.0583 (−2.05 ppm).

(±)-*anti*-3j (±)-*anti* 3,3,3-trifluoro-1-(4-methoxyphenyl)propane-1,2-diol Prepared from **1j** (125 mg, 0.5 mmol) according to the *General procedure for $NaBH_4$ reduction* (83.9 mg, 71% isol. crude yield); *anti*/*syn* = 97:3. The crude product was purified by flash column chromatography, eluent dichlorometane/methanol 30:1 to get (±)-*anti*-**3j** as colorless oil.

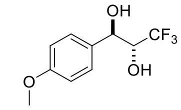

Chemical Formula: $C_{10}H_{11}F_3O_3$
Molecular Weight: 236,19

^{1}H NMR (400 MHz, Methanol-d_4) δ 7.32 (d, *J* = 8.6 Hz, 2H), 6.89 (d, *J* = 8.8 Hz, 2H), 4.86 (d, *J* = 7.7 Hz, 1H), 4.05 – 3.90 (m, 1H), 3.78 (s, 3H). **^{19}F NMR** (376 MHz, Methanol-d_4) δ −76.39 (d, *J* = 6.9 Hz). **^{13}C NMR** (101 MHz, Methanol-d_4) δ 160.88, 134.62, 130.09–128.82 (m), 126.73 (q, *J* = 282.8 Hz), 114.45, 73.98 (q, *J* = 28.0 Hz), 73.41 (q, *J* = 1 Hz), 55.66. **HRMS** (ESI) m/z: $[M-H]^-$ Calcd for $C_{10}H_{10}O_3F_3$ 235.0586; Found 235.0582 (− 2.22 ppm).

***syn*-3k 3-((1*R*,2*S*)-3,3,3-trifluoro-1,2-dihydroxypropyl)phenyl acetate.** Prepared from **1k** (140 mg, 0.5 mmol) according to the *General procedure for DKR-ATH* using (*S*,*S*)-**C4**, S/C = 1000. Full conversion after 1 h (118 mg, 89% isol. crude yield) *syn*/*anti* = 96:4; ee (*syn*) = 96%. The crude product was purified by preparative thin layer chromatography, eluent ethyl acetate/hexane 1:2 to get the diastereomerically pure compound as light yellow oil.

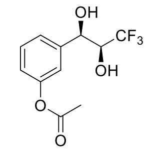

Chemical Formula: $C_{11}H_{11}F_3O_4$
Molecular Weight: 264,2002

Prepared from **1k** (20 mg, 0.072 mmol) according to the *General procedure for DKR-ATH* using (*S*,*S*)-**C5**, S/C = 100. Full conversion after 2 h (11 mg, 60% isol. crude yield). *syn*/*anti* = 98:2; ee *syn* = 97%

^{1}H NMR (400 MHz, Chloroform-*d*) δ 7.40 (t, *J* = 7.9 Hz, 1H), 7.24 (s, 1H), 7.17 (t, *J* = 1.9 Hz, 1H), 7.07 (ddd, *J* = 8.1, 2.3, 0.9 Hz, 1H), 5.03 (dd, *J* = 4.4, 2.5 Hz, 1H), 4.08–3.96 (m, 1H), 3.44 (d, *J* = 8.3 Hz, 1H), 2.78 (d, *J* = 4.6 Hz, 1H), 2.31 (s, 3H). **^{19}F NMR** (376 MHz, Methanol-d_4) δ −77.06 (d, *J* = 8.0 Hz). **^{13}C NMR** (101 MHz, Methanol-d_4) δ 171.29, 152.23, 144.58, 130.15, 126.25 (*J* = 283.0 Hz), 125.21, 122.06, 121.22, 74.50 (q, *J* = 28.7 Hz), 71.83, 20.92. **HRMS** (ESI) m/z: $[M-H]^-$ Calcd for $C_{11}H_{10}O_4F_3$ 263.0537; Found 263.0535 (− 0.52 ppm).

***ent*-*syn*-3k 3-((1*S*,2*R*)-3,3,3-trifluoro-1,2-dihydroxypropyl)phenyl acetate.** Prepared from **1k** (26.2 mg, 0.10 mmol) according to the *General procedure for DKR-ATH* using (*R*,*R*)-**C4** (0.062 mg, S/C = 1000). Reaction was stopped after 3.5 h because of catalyst deactivation and onset of deacetylation. Note that catalyst was deactivated due to low scale of the reaction. The crude product obtained after extraction was used for determination of ee of compound *syn*-**3k**. *syn*/*anti* = 98:2

***syn*-3l (1*R*,2*S*)-3,3,3-trifluoro-1-(4-(hydroxymethyl)phenyl)propane-1,2-diol.** Prepared from **1l** (115 mg, 0.5 mmol) according to the *General procedure for DKR-ATH* using (*S*,*S*)-**C5**, S/C = 100 to get *syn*-**3l**. 14% of residual aldehyde after 2 h (73.8 mg, 63% isol. crude yield); *syn*/*anti* = 93:7; ee (*syn*) >99%**.** The crude oily brown product was suspended in acetone and diethyl ether, filtered, concentrated and further purified by flash column chromatography, eluent dichloromethane/methanol 15/1 to get the stereomerically pure compound as light brown oil.

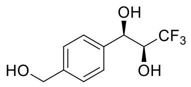

Chemical Formula: $C_{10}H_{11}F_3O_3$
Molecular Weight: 236,1902

Prepared from **1l** (115 mg, 0.5 mmol) according to the *General procedure for DKR-ATH* using (*S*,*S*)-**C4**, S/C = 100 to get *syn*-**3l**. 20% of residual aldehyde after 3 h (63 mg, 53% isol. crude yield); *syn*/*anti* = 75:25; ee (*syn*) >99%**.**

^{1}H NMR (400 MHz, Methanol-d_4) δ 7.43 (d, *J* = 8.1 Hz, 2H), 7.36 (d, *J* = 8.3 Hz, 2H), 4.89 (s, 1H), 4.62 (s, 2H), 4.04 (qd, *J* = 7.4, 3.9 Hz, 1H). **^{19}F NMR** (376 MHz, Methanol-d_4) δ -76.89 (d, *J* = 7.9 Hz). **^{13}C NMR** (101 MHz, Methanol-d_4) δ 142.30, 141.44, 127.89, 127.85, 126.32 (q, *J* = 283.0 Hz), 74.73 (q, *J* = 28.5 Hz), 72.32 (q, *J* = 2 Hz), 64.93. **HRMS** (ESI) m/z: $[M-H]^-$ Calcd for $C_{10}H_{10}O_3F_3$ 235.0588; Found 235.0583 (− 2.14 ppm).

(±)-*anti*-3l (±)-*anti* 3,3,3-trifluoro-1-(4-(hydroxymethyl)phenyl)propane-1,2-diol. Prepared from **1l** (115 mg, 0.5 mmol) according to the *General procedure for NaBH₄ reduction* to get (±)-*anti*-**3l** as light yellow oil (34 mg, 29% isol. crude yield). *anti*/*syn* = 96:4. **¹H NMR** (400 MHz, DMSO-d_6) δ 7.33 (d, *J* = 8.1 Hz, 2H), 7.26 (d, *J* = 8.2 Hz, 2H), 6.16 (d, *J* = 7.2 Hz, 1H), 5.64 (d, *J* = 5.2 Hz, 1H), 5.15 (t, *J* = 5.7 Hz, 1H), 4.55 (dd, *J* = 7.8, 5.2 Hz, 1H), 4.47 (d, *J* = 5.7 Hz, 2H), 4.02–3.84 (m, *J* = 7.5 Hz, 1H). **¹⁹F NMR** (376 MHz, DMSO-d_6) δ −73.02 (d, *J* = 7.6 Hz). **¹³C NMR** (101 MHz, DMSO-d_6) δ 141.63, 140.68, 127.32 (2C), 125.86 (2C), 125.72 (q, *J* = 284.3 Hz), 72.85 (q, *J* = 26.85 Hz), 71.75, 62.76. **HRMS** (ESI) m/z: $[M - H]^-$ Calcd for $C_{10}H_{10}O_3F_3$ 235.0588; Found 235.0583 (− 2.14 ppm).

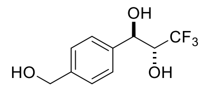

Chemical Formula: $C_{10}H_{11}F_3O_3$
Molecular Weight: 236,19

***syn*-3m (1*R*,2*S*)-3,3,3-trifluoro-1-(pyridin-3-yl)propane-1,2-diol.** Prepared from **2m** (103 mg, 0.5 mmol) according to the *General procedure for DKR-ATH* using (*S*,*S*)-**C4**, S/C = 500. Full conversion after 3 h (72.5 mg, 70% isol. crude yield) *syn*/*anti* = 92:8; ee (*syn*) = 99%. The crude product was purified by flash column chromatography, eluent dichloromethane/methanol/amonium hydroxide 9:1:0.1 to get the diastereomerically pure compound as white solid.

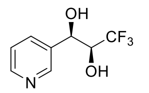

Chemical Formula: $C_8H_8F_3NO_2$
Molecular Weight: 207,1522

¹H NMR (400 MHz, Methanol-d_4) δ 8.62 (d, *J* = 2.1 Hz, 1H), 8.46 (dd, *J* = 4.9, 1.6 Hz, 1H), 7.99–7.91 (m, 1H), 7.44 (ddd, *J* = 7.9, 4.9, 0.6 Hz, 1H), 5.00 (d, *J* = 3.3 Hz, 1H), 4.13–4.00 (m, 1H). **¹⁹F NMR** (376 MHz, Methanol-d_4) δ −76.70 (d, *J* = 7.9 Hz). **¹³C NMR** (101 MHz, Methanol-d_4) δ 149.24, 148.85, 139.03, 136.99, 126.27 (q, *J* = 283 Hz), 124.92, 74.24 (q, *J* = 28.9 Hz), 70.45 (q, *J* = 2 Hz). **HRMS** (ESI) m/z: $[M + H]^+$ Calcd for $C_8H_9O_2NF_3$ 208.0580; Found 208.0577 (− 1.63 ppm).

***ent-syn*-3m (1*S*,2*R*)-3,3,3-trifluoro-1-(pyridin-3-yl)propane-1,2-diol.** Prepared from **2m** (61.0 mg, 0.3 mmol) according to the *General procedure for DKR-ATH* using (*R*,*R*)-**C4**, S/C = 100. Full conversion after 18 h. The crude product obtained after extraction was used for determination of ee of compound *syn*-**3m**. *syn*/*anti* = 92:8

***syn*-3n (1*R*,2*S*)-1-(2-aminobenzo[*d*]thiazol-6-yl)-3,3,3-trifluoropropane-1,2-diol.** Prepared from **1n** (137 mg, 0.5 mmol) according to the *General procedure for DKR-ATH*. using (*S*,*S*)-**C4**, S/C = 500. Full conversion after 2 h (55.9 mg, 40% isol. crude yield); *syn*/*anti* = 91:9; ee (*syn*) >99%. The crude product was purified by flash column chromatography, eluent dichloromethane/methanol/amonium hydroxide 9:1:0.1 to get the stereomerically pure compound as off-white solid.

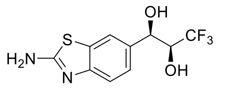

Chemical Formula: $C_{10}H_9F_3N_2O_2S$
Molecular Weight: 278,2492

Prepared from **1n** (137 mg, 0.5 mmol) according to the *General procedure for DKR-ATH* using (*S*,*S*)-**C5**, S/C = 500. Full conversion after 18 h (90.4 mg, 65% isol. crude yield). *syn*/*anti* = 96:4; ee (*syn*) >99%

¹H NMR (400 MHz, DMSO-d_6) δ 7.67 (d, *J* = 1.5 Hz, 1H), 7.43 (s, 2H), 7.24 (dt, *J* = 8.3, 4.9 Hz, 2H), 6.14 (d, *J* = 7.8 Hz, 1H), 5.57 (d, *J* = 5.6 Hz, 1H), 4.92–4.55 (m, 1H), 4.00 (ddd, *J* = 15.4, 9.6, 5.7 Hz, 1H). **¹⁹F NMR** (376 MHz, Methanol-d_4) δ −76.85 (d, *J* = 7.3 Hz). **¹³C NMR** (101 MHz, Methanol-d_4) δ 170.04, 152.67, 136.20, 131.90, 126.33 (q, *J* = 283 Hz), 125.77, 120.43, 118.34, 74.78 (q, *J* = 28.4 Hz), 72.35 (q, *J* = 2 Hz). **HRMS** (ESI) m/z: $[M + H]^+$ Calcd for $C_{10}H_{10}O_2N_2F_3S$ 279.0410; Found 279.0404 (− 1.93 ppm).

***ent-syn*-3n (1*S*,2*R*)-1-(2-aminobenzo[*d*]thiazol-6-yl)-3,3,3-trifluoropropane-1,2-diol.** Prepared from **1n** (120 mg, 0.44 mmol) according to the *General procedure for DKR-ATH* ((*R*,*R*)-**C4**-cat., S/C = 1000) to get *ent-syn*-**3n**. Full conversion after 2 h (45.0 mg, 36.75% isol. crude yield). The crude product was purified by flash column chromatography, eluent dichloromethane/methanol/amonium hydroxide 9/1/0.1 to get the stereomeric pure compound as off-white solid. *syn*/*anti* = 92:8; ee (*syn*) >99%

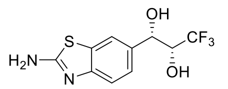

Chemical Formula: $C_{10}H_9F_3N_2O_2S$
Molecular Weight: 278,25

¹H NMR (400 MHz, DMSO-d_6) δ 7.67 (d, *J* = 1.3 Hz, 1H), 7.44 (s, 2H), 7.24 (dt, *J* = 8.3, 4.9 Hz, 2H), 6.14 (d, *J* = 7.8 Hz, 1H), 5.57 (d, *J* = 5.4 Hz, 1H), 4.76 (t, *J* = 4.1 Hz, 1H), 4.06–3.93 (m, *J* = 7.6, 3.8 Hz, 1H). **¹⁹F NMR** (376 MHz, Methanol-d_4) δ −76.85 (d, *J* = 7.4 Hz). **HRMS** (ESI) m/z: $[M + H]^+$ calcd. for $C_{10}H_{10}O_2N_2F_3S$ 279.0410; Found 279.0405 (− 1.75 ppm).

(±)-*anti*-3n (±)-*anti* 1-(2-aminobenzo[*d*]thiazol-6-yl)-3,3,3-trifluoropropane-1,2-diol. Prepared from **1n** (137 mg, 0.5 mmol) according to the *General procedure for NaBH₄ reduction* (127 mg, 91% isol. crude yield). The crude product was purified by flash column chromatography, eluent dichloromethane/methanol/amonium hydroxide 9:1:0.1 to get (±)-*anti*-**3n** as off-white solid. *anti*/*syn* = 96:4

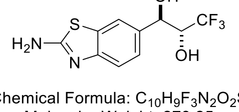

Chemical Formula: $C_{10}H_9F_3N_2O_2S$
Molecular Weight: 278,25

¹H NMR (400 MHz, DMSO-d_6) δ 7.64 (d, *J* = 1.5 Hz, 1H), 7.43 (s, 2H), 7.24 (dt, *J* = 8.3, 4.9 Hz, 2H), 6.14 (d, *J* = 7.1 Hz, 1H), 5.63 (d, *J* = 4.9 Hz, 1H), 4.57 (dd, *J* = 7.9, 4.8 Hz, 1H), 3.92 (dd, *J* = 14.9, 7.4 Hz, 1H). **¹⁹F NMR** (376 MHz, Methanol-d_4) δ −76.21 (d, *J* = 7.5 Hz). **¹³C NMR** (101 MHz, Methanol-d_4) δ 170.03, 152.63, 136.31, 131.68, 126.66 (q, *J* = 283 Hz), 126.61, 121.13, 118.21, 73.99 (q, *J* = 28.1 Hz), 73.74. **HRMS** (ESI) m/z: $[M + H]^+$ Calcd for $C_{10}H_{10}O_2N_2F_3S$ 279.0410; Found 279.0405 (− 1.54 ppm).

***syn*-3o (2*S*,3*R*)-1,1,1-trifluoro-4-phenylbutane-2,3-diol.** Prepared from **2o** (109 mg, 0.5 mmol) according to the *General procedure for DKR-ATH* using (*S*,*S*)-**C4**, S/C = 500. Full conversion after 2 h (95.8 mg, 87% isol. crude yield). The crude product was purified by preparative thin layer chromatography, eluent ethyl acetate/hexane 1:3 to get the stereomerically pure compound as white solid. *syn*/*anti* = 94:6; ee (*syn*) = 95%.

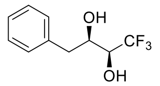

Chemical Formula: $C_{10}H_{11}F_3O_2$
Molecular Weight: 220,19

Prepared from **2o** (109 mg, 0.5 mmol) according to the *General procedure for DKR-ATH* using (*S*,*S*)-**C5**, S/C = 500. Full conversion after 2 h (91.4 mg, 83% isol. crude yield). *syn*/*anti* = 87:13; ee (*syn*) = 96%

^{1}H NMR (400 MHz, Methanol-*d$_4$*) δ 7.39–7.13 (m, 5H), 4.03 (td, *J* = 7.2, 1.6 Hz, 1H), 3.73 (qd, *J* = 7.6, 1.8 Hz, 1H), 2.89 (d, *J* = 7.2 Hz, 2H). **^{19}F NMR** (376 MHz, Methanol-*d$_4$*) δ −77.36 (d, *J* = 7.9 Hz). **^{13}C NMR** (101 MHz, Methanol-*d$_4$*) δ 139.48, 130.45, 129.49, 127.48, 126.69 (q, *J* = 283 Hz), 71.30 (q, *J* = 2 Hz), 71.29 (q, *J* = 28.9 Hz), 40.99. **HRMS** (ESI) m/z: [M − H]$^-$ Calcd for $C_{10}H_{10}O_2F_3$ 219.0638; Found 219.06320 (− 2.96 ppm).

(±)-*anti*-3o (±)-*anti* 1,1,1-trifluoro-4-phenylbutane-2,3-diol Prepared from **2o** (83 mg, 0.37 mmol) according to the *General procedure for NaBH$_4$ reduction* (66.80 mg, 82% isol. crude yield) *anti*/*syn* = 65:35. The crude product was purified by preparative thin layer chromatography, eluent dichloromethane/methanol 30/1 to get (±)-*anti*-**2o** as white solid.

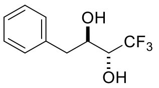

Chemical Formula: $C_{10}H_{11}F_3O_2$
Molecular Weight: 220,19

^{1}H NMR (400 MHz, Methanol-*d$_4$*) δ 7.33–7.14 (m, 5H), 3.91 (ddd, *J* = 9.5, 7.1, 2.7 Hz, 1H), 3.79 (p, *J* = 7.3 Hz, 1H), 3.09 (dd, *J* = 14.0, 2.7 Hz, 1H), 2.69 (dd, *J* = 14.0, 9.3 Hz, 1H). **^{19}F NMR** (376 MHz, Methanol-*d$_4$*) δ −76.51 (d, *J* = 6.9 Hz). **^{13}C NMR** (101 MHz, Methanol-*d$_4$*) δ 139.98, 130.72, 129.21, 127.20, 126.81 (q, *J* = 282.9 Hz), 73.67 (q, *J* = 27.9 Hz), 72.33, 40.25. **HRMS** (ESI) m/z: [M + H]$^+$ Calcd for $C_{10}H_{12}O_2F_3$ 221.0784; Found 221.0782 (− 0.77 ppm).

***syn*-3p (2*S*,3*R*)-1,1,1-trifluorononane-2,3-diol.** Prepared from **2p** (106 mg, 0.5 mmol) according to the *General procedure for DKR-ATH* using (*S*,*S*)-**C4**, S/C = 500. Full conversion after 2.5 h (107.11 mg, 100% isol. crude yield) *syn*/*anti* = 77:23; ee (*syn*) 96%. The crude product was purified by flash column chromatography, eluent ethyl acetate/hexane 1:5 to get the stereomeric pure compound as white solid.

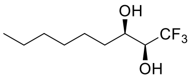

Chemical Formula: $C_9H_{17}F_3O_2$
Molecular Weight: 214,2282

Prepared from **2p** (33.3 mg, 0.16 mmol) according to the *General procedure for DKR-ATH* using (*S*,*S*)-**C5**, S/C = 100). Full conversion after 18 h (21.4 mg, 62.5% isol. crude yield). *syn*/*anti* = 90:10; ee (*syn*) = 98%

^{1}H NMR (400 MHz, Methanol-*d$_4$*) δ 3.90–3.65 (m, 2H), 1.71–1.45 (m, 3H), 1.32 (s, 7H), 0.91 (t, *J* = 6.9 Hz, 3H). **^{19}F NMR** (376 MHz, Methanol-*d$_4$*) δ −77.29 (d, *J* = 7.6 Hz). **^{13}C NMR** (101 MHz, Methanol-*d$_4$*) δ 126.67 (q, *J* = 283 Hz), 72.83 (q, *J* = 28.7 Hz), 69.94 (q, *J* = 2 Hz), 34.57, 32.97, 30.31, 26.70, 23.67, 14.40. **HRMS** (ESI) m/z: [M − H]$^-$ Calcd for $C_9H_{16}O_2F_3$ 213.1108; Found 213.1101 (−3.13 ppm). **HRMS** (ESI) m/z: [M + H]$^+$ Calcd for $C_9H_{18}O_2F_3$ 215.1253; Found 215.1251 (− 1.07 ppm).

(±)-*anti*-3p (±)-*anti* 1,1,1-trifluorononane-2,3-diol. Prepared from **2p** (106 mg, 0.5 mmol) according to the *General procedure for NaBH$_4$ reduction* (107.11 mg, 100% isol. crude yield) *anti*/*syn*: 69:31. The crude product was purified by flash column chromatography, eluent ethyl acetate/hexane 1/5 to get (±)-*anti*-**3p** as white solid.

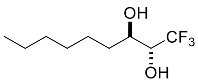

Chemical Formula: $C_9H_{17}F_3O_2$
Molecular Weight: 214,23

^{1}H NMR (400 MHz, Methanol-*d$_4$*) δ 3.74 (dd, *J* = 14.3, 7.2 Hz, 1H), 3.70–3.64 (m, 1H), 1.72 (ddd, *J* = 11.0, 8.3, 2.8 Hz, 1H), 1.62–1.52 (m, 1H), 1.52–1.43 (m, 1H), 1.38–1.29 (m, 7H), 0.91 (dd, *J* = 9.2, 4.4 Hz, 3H). **^{19}F NMR** (376 MHz, Methanol-*d$_4$*) δ −76.68 (d, *J* = 7.0 Hz). **^{13}C NMR** (101 MHz, Methanol-*d$_4$*) δ 126.80 (q, *J* = 283 Hz), 74.02 (q, *J* = 27.7 Hz), 70.95, 33.68, 33.01, 30.37, 26.33, 23.69, 14.42. **HRMS** (ESI) m/z: [M + H]$^+$ Calcd for $C_9H_{18}O_2F_3$ 215.1253; Found 215.1252 (− 0.89 ppm).

***syn*-3q *N*-((4*R*,5*S*)-6,6,6-trifluoro-4,5-dihydroxyhexyl)benzamide.** Prepared from **2q** (144.62 mg, 0.5 mmol) according to the *General procedure for DKR-ATH* using (*S*,*S*)-**C5**., S/C = 100. Full conversion after 2 h (85.0 mg, 58% isol. crude yield); *syn*/*anti* = 92:8; ee (*syn*) = 96%. The crude product was purified by preparative thin layer chromatography, eluent dichloromethane/methanol 20:1 to get the title compound as off white oil.

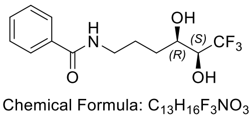

Chemical Formula: $C_{13}H_{16}F_3NO_3$
Molecular Weight: 291,27

Prepared from **2q** (26 mg, 0.09 mmol) according to the *General procedure for DKR-ATH* using (*S*,*S*)-**C4**, S/C = 100). Full conversion after 1 h (18.6 mg, 72% isol. crude yield); *syn*/*anti* = **79:21**; ee (*syn*) = 72%.

^{1}H NMR (400 MHz, Chloroform-*d*) δ 7.75 (dd, *J* = 8.3, 1.3 Hz, 2H), 7.57–7.48 (m, 1H), 7.48–7.39 (m, 2H), 6.44 (s, 1H), 4.16–3.94 (m, 1H), 3.83–3.71 (m, 1H), 3.69–3.36 (m, 4H), 1.89–1.55 (m, 4H). **^{19}F NMR** (376 MHz, Methanol-*d$_4$*) δ -77.18 (d, *J* = 7.7 Hz). **^{13}C NMR** (101 MHz, Methanol-*d$_4$*) δ 170.31, 135.79, 132.55, 129.51, 128.21, 126.60 (q, *J* = 282.8 Hz), 72.90 (q, *J* = 28.7 Hz), 69.69 (q, *J* = 1 Hz), 40.78, 31.91, 26.78. **HRMS** (ESI) m/z: [M + H]$^+$ Calcd for $C_{13}H_{17}O_3NF_3$ 292.1155; Found 292.1148 (− 2.41 ppm).

(±)-*anti*-3q (±)-*anti* 6,6,6-trifluoro-4,5-dihydroxyhexyl)benzamide. Prepared from **2q** (86 mg, 0.3 mmol) according to the *General procedure for $NaBH_4$ reduction* (44 mg, 50.7% isol. crude yield); *anti*/*syn* = 65:35. The crude product was purified by preparative thin layer chromatography, eluent dichloromethane/methanol 20/1 to get (±)-*anti*-**2r** as white solid.

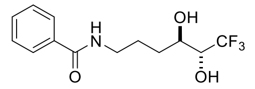

Chemical Formula: $C_{13}H_{16}F_3NO_3$
Molecular Weight: 291,27

^{1}H NMR (400 MHz, Methanol-*d_4*) δ 7.76 – 7.63 (m, 2H), 7.48 – 7.39 (m, 1H), 7.39 – 7.29 (m, 2H), 3.71 – 3.60 (m, 2H), 3.32 (t, *J* = 6.7 Hz, 2H), 1.88 – 1.69 (m, 2H), 1.66 – 1.54 (m, 1H), 1.52 – 1.34 (m, 1H). **^{19}F NMR** (376 MHz, Methanol-*d_4*) δ -76.72 (d, *J* = 7.1 Hz). **^{13}C NMR** (101 MHz, Methanol-*d_4*) δ 170.32, 135.86, 132.55, 129.52, 128.22, 126.78 (q, *J* = 282.8 Hz), 73.98 (q, *J* = 27.9 Hz), 70.72, 40.93, 31.13, 26.49. **HRMS** (ESI) m/z: [M + H]$^+$ Calcd for $C_{13}H_{17}O_3NF_3$ 292.1155; Found 292.1148 (− 2.31 ppm).

***syn*-3r (2*R*,3*S*,*E*)-1,1,1-trifluoro-5-phenylpent-4-ene-2,3-diol.** Prepared from **2r** (115 mg, 0.5 mmol) according to the *General procedure for DKR-ATH* using (*R*,*R*)-**C4**, S/C = 500. Full conversion after 2 h (108 mg, 93% isol. crude yield) *syn*/*anti* = 96:4; ee (*syn*) 97%. The major product **3r** was accompanied by 30 mol% of double bond reduction products (by ^{1}H NMR). The crude product was purified by flash column chromatography, eluent dichloromethane/MeOH 40:1 to get the title compound as white solid (50 mg, 43% isol. yield).

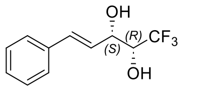

Chemical Formula: $C_{11}H_{11}F_3O_2$
Molecular Weight: 232,20

(2*S*,3*R*,*E*)-3r. Prepared from **2r** (115 mg, 0.5 mmol) according to the *General procedure for DKR-ATH* using (*S*,*S*)-**C5**, S/C = 500). Full conversion after 2 h (112 mg, 96% isol. crude yield). *syn*/*anti* = 97:3; The major product **3r** was accompanied by 16 mol% of double bond reduction products (by ^{1}H NMR). The crude product was purified by flash column chromatography, eluent dichloromethane/MeOH 40:1 to get the title compound as white solid (77 mg, 66% isol. yield).

^{1}H NMR (400 MHz, Chloroform-*d*) δ 7.43–7.28 (m, 5H), 6.76 (d, *J* = 15.9 Hz, 1H), 6.28 (dd, *J* = 15.9, 7.0 Hz, 1H), 4.65 (dt, *J* = 6.9, 2.9 Hz, 1H), 4.05–3.82 (m, 1H), 3.08 (d, *J* = 8.1 Hz, 1H), 2.16 (d, *J* = 3.8 Hz, 1H). **^{19}F NMR** (376 MHz, Chloroform-*d*) δ −76.45 (d, *J* = 7.6 Hz). **^{13}C NMR** (101 MHz, Chloroform-*d*) δ 135.84, 133.91, 128.84, 128.55, 126.87, 126.06, 124.42 (q, *J* = 283.1 Hz), 72.67 (q, *J* = 29.7 Hz), 70.02 (q, *J* = 2.0 Hz). **HRMS** (ESI) m/z: [M + Na]$^+$ Calcd for $C_{11}H_{11}O_2F_3Na$ 255.0603; Found 255.0601 (− 0.96 ppm).

5 Further synthetic transformations of the diols 3e and 3n

*Synthesis of the glycogen phosphorylase inhibitor **5**.*

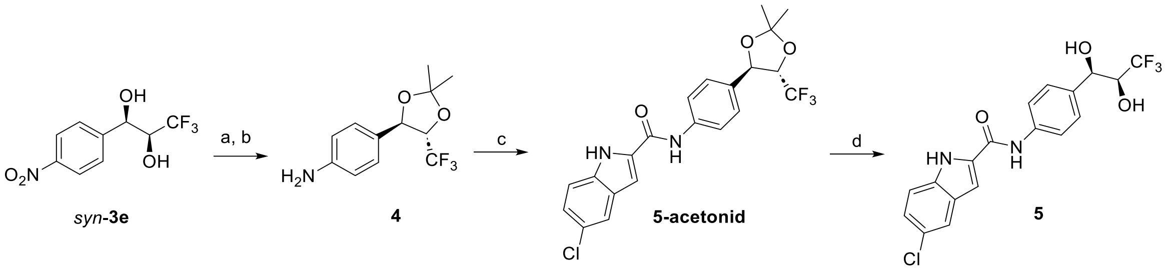

Reagents and conditions: (a) 2,2-dimethoxypropane, acetone, *p*TsOH x H_2O, 22 °C, 20 h; (b) Fe^0, AcOH, 22 °C, 2 h; (c) 5-chloro-1*H*-indole-2-carbonyl chloride, DCM, pyridine, 22 °C, 20 h; (d) 4 M/HCl in dioxane, EtOH, 22 °C, 20 h.

Synthesis:

Step a: **(4*R*,5*S*)-2,2-dimethyl-4-(4-nitrophenyl)-5-(trifluoromethyl)-1,3-dioxolane.** To a solution of *syn*-**3e** (0.59 g, 2.35 mmol) in acetone (15 mL), 30 mL of 2,2-dimethoxypropane and *p*-toluenesulfonic acid monohydrate (0.47 mmol, 0.2 eq) were added. The reaction mixture was stirred at 22 °C for 20 h. Et_3N (0.47 mmol, 0.2 eq) was then added and the reaction mixture was concentrated. The crude oily residue was dissolved in ethyl acetate. The organic layer was washed with H_2O, brine, dried over Na_2SO_4, filtered and concentrated. The crude residue was further purified by flash column chromatography, eluent ethyl acetate/hexane 1:8 to obtain the title compound as white solid (0.45 g, 66% yield). ^{1}**H NMR** (400 MHz, Chloroform-*d*) δ 8.31 – 8.18 (m, 2H), 7.61 (d, *J* = 8.7 Hz, 2H), 5.29 (d, *J* = 7.5 Hz, 1H), 4.45 – 3.84 (m, 1H), 1.63 (s, 3H), 1.58 (s, 3H). ^{19}**F NMR** (376 MHz, Chloroform-*d*) δ -75.35 (d, *J* = 6.0 Hz).

Step b: **4-((4*R*,5*S*)-2,2-dimethyl-5-(trifluoromethyl)-1,3-dioxolan-4-yl)aniline (4).** To a solution of the above nitro compound (0.45 g, 1.54 mmol) in AcOH (10 mL/mmol), Fe^0 (15.45 mmol, 10 eq) was added and the resulting suspension vigorously stirred for 2 h. Water (20 mL) and methanol (20 mL) were added, the resulting suspension filtered through cotton and concentrated under reduced pressure. The oily residue was partitioned between water (20 mL) and ethyl acetate (3 x 20 mL). The combined organic layers were washed with saturated aqueous $NaHCO_3$, brine, dried over Na_2SO_4, filtered and concentrated to obtain the title compound as brown oil (0.245 g, 63.7% yield). ^{1}**H NMR** (400 MHz, Chloroform-*d*) δ 7.21 – 7.16 (m, 2H), 6.72 – 6.65 (m, 2H), 5.08 (d, *J* = 7.9 Hz, 1H), 4.15 (dq, *J* = 7.8, 6.2 Hz, 1H), 3.75 (s, 2H), 1.59 (s, 3H), 1.53 (s, 3H). ^{19}**F NMR** (376 MHz, Chloroform-*d*) δ -75.42 (d, *J* = 5.7 Hz). ^{13}**C NMR** (101 MHz, $CDCl_3$) δ 147.26, 128.27 (2C), 125.97, 123.91 (q, *J* = 280.5 Hz), 115.23 (2C), 111.94, 80.49 (q, *J* = 31.0 Hz), 78.59 (q, *J* = 2.0 Hz), 27.43, 25.92.

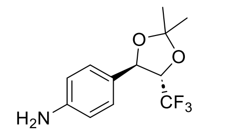

Chemical Formula: $C_{12}H_{14}F_3NO_2$
Molecular Weight: 261,24

Step c: 5-chloro-*N*-(4-((4*R*,5*S*)-2,2-dimethyl-5-(trifluoromethyl)-1,3-dioxolan-4-yl)phenyl)-1*H*-indole-2-carboxamide (5-acetonid). To a solution of 5-chloro-1*H*-indole-2-carbonyl chloride [prepared by stirring the corresponding acid (94.1 mg, 0.48 mmol) in $SOCl_2$ (1.2 mL) at 75 °C for 2 h, then concentrating at reduced pressure] in dry dichloromethane (4.8 mL) were added the above aniline **4** (100 mg, 0.40 mmol) and dry pyrridine (0.48 mL). The reaction mixture was stirred overnight at room temperature, then concentrated and dissolved in ethyl acetate. The organic layer was washed with 10% citric acid, saturated aqueous $NaHCO_3$, H_2O, brine, dried over Na_2SO_4, filtered and concentrated to obtain the title compound as yellow solid (118 mg, 67% yield). ^{1}**H NMR** (400 MHz, DMSO-*d₆*) δ 11.99 (s, 1H), 10.39 (s, 1H), 7.85 (d, *J* = 8.6 Hz, 2H), 7.79 (d, *J* = 2.0 Hz, 1H), 7.56 – 7.37 (m, 4H), 7.23 (dd, *J* = 8.7, 2.1 Hz, 1H), 5.23 (d, *J* = 8.0 Hz, 1H), 4.95 – 4.32 (m, 1H), 1.56 (s, 3H), 1.49 (s, 3H). ^{19}**F NMR** (376 MHz, DMSO-*d₆*) δ -74.14 (d, *J* = 6.1 Hz). ^{13}**C NMR** (101 MHz, DMSO) δ 159.47, 139.43, 135.26, 132.83, 131.04, 128.03 (2C), 124.45, 123.99, 123.96 (q, *J* = 280.5 Hz), 120.86, 120.13 (2C), 118.92, 114.03, 111.53, 103.59, 78.71 (q, *J* = 30.3 Hz), 77.42, 27.00, 25.75.

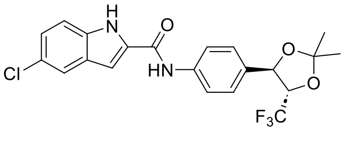

Chemical Formula: $C_{21}H_{18}ClF_3N_2O_3$
Molecular Weight: 438,83

Step d: **5-chloro-*N*-(4-((1*R*,2*S*)-3,3,3-trifluoro-1,2-dihydroxypropyl)phenyl)-1*H*-indole-2-carboxamide (5).**[16] To a solution of the above acetonide (50 mg, 0.11 mmol) in absolute ethanol (1.2 mL), 4 M HCl in dioxane (0.28 mL, 10 eq) was added. After 4 hours, additional 10 eq of 4 M HCl in dioxane were added. The reaciton mixture was stirred overnight at room temperature and concentrated. The crude residue was dissolved in ethyl acetate, washed with saturated aqueous $NaHCO_3$, H_2O, brine, dried over Na_2SO_4, filtered and concentrated. The crude solid was triturated with chloroform to obtain the title compound as an off white solid (28 mg, 62% yield). **^{1}H NMR** (400 MHz, DMSO-*d$_6$*) δ 11.96 (s, 1H), 10.29 (s, 1H), 7.87–7.68 (m, 3H), 7.44 (dd, *J* = 26.9, 8.4 Hz, 4H), 7.23 (d, *J* = 8.6 Hz, 1H), 6.19 (d, *J* = 7.7 Hz, 1H), 5.61 (d, *J* = 5.6 Hz, 1H), 4.86–4.53 (m, 1H), 4.04 (dd, *J* = 8.9, 5.3 Hz, 1H). **^{19}F NMR** (376 MHz, DMSO-*d$_6$*) δ −73.30 (d, *J* = 7.6 Hz). **^{13}C NMR** (101 MHz, DMSO-*d$_6$*) δ 159.30, 137.86, 136.99, 135.18, 133.01, 128.07, 127.20, 125.27 (q, *J* = 285 Hz) 124.40, 123.86, 120.80, 119.68, 113.99, 103.34, 72.74 (q, *J* = 27.2 Hz), 70.48. **HRMS** (ESI) m/z: [M − H]$^-$ Calcd for $C_{18}H_{13}O_3N_2ClF_3$ 397.0572; Found 397.0575 (+ 0.58 ppm).

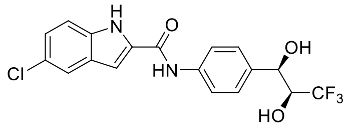

Chemical Formula: $C_{18}H_{14}ClF_3N_2O_3$
Molecular Weight: 398,7662

Synthesis of stereoarrayed analogs of the topoisomerase inhibitor ULD1.

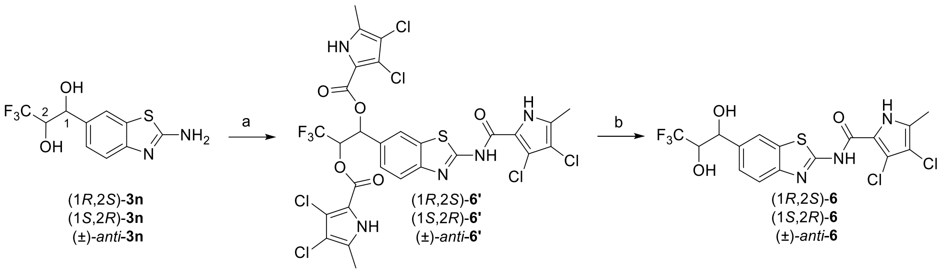

Reagents and conditions: (a) 3,4-dichloro-5-methyl-1*H*-pyrrole-2-carbonyl chlorid, toluene, 130 °C, 20 h; (b) 2 M NaOH (aq), MeOH, 22 °C, 18 h.

(1*R*,2*S*)-1-(2-(3,4-dichloro-5-methyl-1*H*-pyrrole-2-carboxamido)benzo[*d*]thiazol-6-yl)-3,3,3-trifluoropropane-1,2-diyl bis(3,4-dichloro-5-methyl-1*H*-pyrrole-2-carboxylate), (1*R*,2*S*)-**6'.** To a fresh batch of 3,4-dichloro-5-methyl-1*H*-pyrrole-2-carbonyl chloride [prepared by stirring the corresponding acid (953 mg, 4.91 mmol) in $SOCl_2$ (12.5 mL) at 75 °C for 2 h, then concentrating at reduced pressure] were added (1*R*,2*S*)-**3n** (414 mg, 1.5 mmol) and toluene (7.4 mL) and the reaction was stirred at 130 °C overnight. The precipitate was collected and washed with cold toluene. The crude product thus obtained was purified by flash column shromatography, eluent dichloromethane/methanol 30/1 to get the title compound as black solid (546 mg, 46% yield). **^{1}H NMR** (400 MHz, DMSO-*d$_6$*) δ 12.49 (s, 1H), 12.43 (s, 1H), 12.35 (s, 1H), 11.81 (s, 1H), 8.25 (s, 1H), 7.75 (s, 1H), 7.67 (dd, *J* = 8.4, 1.4 Hz, 1H), 6.65 (d, *J* = 5.0 Hz, 1H), 6.29–6.21 (m, 1H), 2.27 (s, 3H), 2.20 (s, 3H), 2.18 (s, 3H). **^{19}F NMR** (376 MHz, DMSO-*d$_6$*) δ −71.28 (d, *J* = 6.7 Hz). **HRMS** (ESI) m/z: [M − H]$^-$ Calcd for $C_{28}H_{17}O_5N_5Cl_6F_3S$ 801.9039; Found 801.90533 (+ 1.77 ppm).

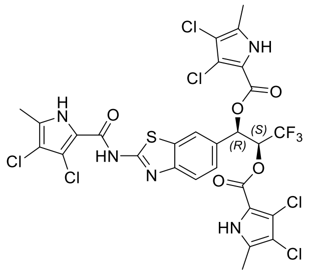

Chemical Formula: $C_{28}H_{18}Cl_6F_3N_5O_5S$
Molecular Weight: 806,24

(1*S*,2*R*)-1-(2-(3,4-dichloro-5-methyl-1*H*-pyrrole-2-carboxamido)benzo[*d*]thiazol-6-yl)-3,3,3-trifluoropropane-1,2-diyl bis(3,4-dichloro-5-methyl-1H-pyrrole-2-carboxylate), (1*S*,2*R*)-**6'.** Prepared analogously to its enantiomer, from (1*S*,2*R*)-**3n.** **^{1}H NMR** (400 MHz, Acetone-*d$_6$*) δ 11.52 (s, 1H), 11.42 (s, 1H), 11.31 (s, 1H), 10.23 (s, 1H), 8.25 (s, 1H), 7.75 (d, *J* = 0.5 Hz, 1H), 7.75 (d, *J* = 1.6 Hz, 1H), 6.65 (d, *J* = 6.0 Hz, 1H), 6.18 (p, *J* = 6.7 Hz, 1H), 2.36 (s, 3H), 2.27 (s, 3H), 2.26 (s, 3H). **^{19}F NMR** (376 MHz, Acetone-*d$_6$*) δ -72.81 (d, *J* = 6.8 Hz). **^{13}C NMR** (101 MHz, Acetone-*d$_6$*) δ 159.48, 158.29, 157.55, 149.94, 133.38, 132.99, 132.26, 132.15, 131.46, 126.72, 123.93 (q, *J* = 281.3 Hz), 122.01 (2C), 121.52, 119.04, 118.31, 118.17, 115.95, 114.77, 114.45, 112.05, 111.69, 111.35, 72.40, 71.47 (q, *J* = 30.9 Hz), 11.22, 11.19, 11.18. **HRMS** (ESI) m/z: [M − H]$^-$ Calcd for $C_{28}H_{17}O_5N_5Cl_6F_3S$ 801.90391; Found 801.90552 (+ 2.00 ppm).

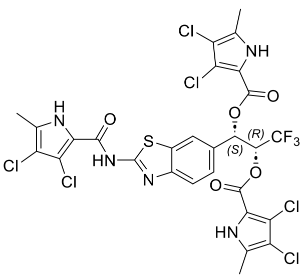

Chemical Formula: $C_{28}H_{18}Cl_6F_3N_5O_5S$
Molecular Weight: 806,24

(±)-*anti*-1-(2-(3,4-dichloro-5-methyl-1*H*-pyrrole-2-carboxamido)benzo[*d*]thiazol-6-yl)-3,3,3-trifluoropropane-1,2-diyl bis(3,4-dichloro-5-methyl-1*H*-pyrrole-2-carboxylate), (±)-*anti*-**6'.** To a fresh batch of 3,4-dichloro-5-methyl-1*H*-pyrrole-2-carbonyl chloride [prepared by stirring the corresponding acid (880 mg, 4.54 mmol) in $SOCl_2$ (11.0 mL) at 75 °C for 2 h, then concentrating at reduced pressure] were added (±)-*anti*-**3n** (383 mg, 1.4 mmol) and toluene (7.0 mL) and the reaction was stirred at 130 °C overnight. The precipitate was collected and washed with cold toluene (45 mg, 4.0% yield). The crude product thus obtained was used directly in the next step, *anti*/*syn* = 2:1. **^{19}F NMR** (376 MHz, DMSO-d_6) δ (−71.20)–(−71.39) (m, *syn*-**6'**), (−71.92)–(−72.20) (m, *anti*-**6'**). **HRMS** (ESI) m/z: [M − H]⁻ Calcd for $C_{28}H_{17}O_5N_5Cl_6F_3S$ [M−H]⁻ 801.90391; Found 801.90549 (+ 1.97 ppm).

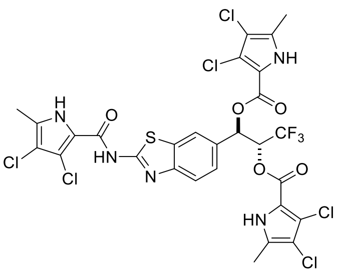

Chemical Formula: $C_{28}H_{18}Cl_6F_3N_5O_5S$
Molecular Weight: 806,24

3,4-dichloro-5-methyl-*N*-(6-((1*R*,2*S*)-3,3,3-trifluoro-1,2-dihydroxypropyl)benzo[*d*]thiazol-2-yl)-1*H*-pyrrole-2-carboxamide, (1*R*,2*S*)-**6.** To a suspension of (1*R*,2*S*)-**6'** (533 mg, 0.66 mmol) in methanol (3.3 mL) was added 2 M NaOH (1.0 mL, 4 eq). The reaction mixture was stirred at 22 °C overnight, then it was neutralised by adding 1 M HCl until pH 7 and concentrated under reduced pressure to get the crude product which was further purified by flash column chromatography, eluent dichloromethane/methanol 30:1 gradient to 4:1 to get the title compound as beige solid (129 mg, 43% yield). **^{1}H NMR** (400 MHz, DMSO-d_6) δ 12.31 (s, 1H), 11.73 (s, 1H), 8.01 (s, 1H), 7.69 (d, *J* = 7.9 Hz, 1H), 7.51 (d, *J* = 8.2 Hz, 1H), 6.22 (s, 1H), 5.76 (s, 1H), 4.91 (d, *J* = 2.9 Hz, 1H), 4.11 (d, *J* = 3.8 Hz, 1H), 2.27 (s, 3H). **^{19}F NMR** (376 MHz, DMSO-d_6) δ -73.33 (d, *J* = 7.6 Hz). **^{13}C NMR** (101 MHz, DMSO-d_6) (representative peaks) δ 137.56, 129.76, 126.69, 125.42, 123.86, 120.01, 114.74, 109.70, 72.73 (q, *J* = 28.0, 26.6 Hz), 70.65, 11.02. **HRMS** (ESI) m/z: [M − H]⁻ Calcd for $C_{16}H_{11}O_3N_3Cl_2F_3S$ 451.98558; Found 451.98518 (− 0.87 ppm).

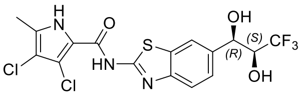

Chemical Formula: $C_{16}H_{12}Cl_2F_3N_3O_3S$
Molecular Weight: 454,25

3,4-dichloro-5-methyl-*N*-(6-((1*S*,2*R*)-3,3,3-trifluoro-1,2-dihydroxypropyl)benzo[*d*]thiazol-2-yl)-1*H*-pyrrole-2-carboxamide, (1*S*,2*R*)-**6.** Prepared analogously to its enantiomer, from (1*S*,2*R*)-**6'**. **^{1}H NMR** (400 MHz, Methano-d_4) δ 8.00 (s, 1H), 7.73 (d, *J* = 8.4 Hz, 1H), 7.54 (dd, *J* = 8.4, 1.5 Hz, 1H), 5.05 (d, *J* = 3.5 Hz, 1H), 4.23 – 3.92 (m, 1H), 2.33 (s, 3H). **^{19}F NMR** (376 MHz, Methanol-d_4) δ −76.92 (d, *J* = 7.4 Hz). **HRMS** (ESI) m/z: [M − H]⁻ Calcd for $C_{16}H_{11}O_3N_3Cl_2F_3S$ 451.98558; Found 451.98523 (− 0.76 ppm).

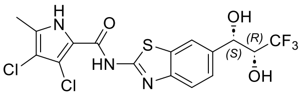

Chemical Formula: $C_{16}H_{12}Cl_2F_3N_3O_3S$
Molecular Weight: 454,25

(±)-*anti*-3,4-dichloro-5-methyl-*N*-(6-(3,3,3-trifluoro-1,2-dihydroxypropyl)benzo[*d*]thiazol-2-yl)-1*H*-pyrrole-2-carboxamide, (±)-*anti*-**6.** To a suspension of (±)-*anti*-**6'** (45 mg, 0.056 mmol) in methanol (0.5 mL) was added 2 M NaOH (0.1 mL, 4 eq). The reaction mixture was stirred at 22 °C overnight, then it was neutralised by adding 1 M HCl until pH 7 and concentrated under reduced pressure to get the crude product which was further purified by flash column chromatography, eluent dichloromethane/methanol 30:1 gradient to 4:1 to get the title compound as beige solid (13 mg, 51% yield); *anti*/*syn* = 73:27. **^{1}H NMR** (400 MHz, DMSO-d_6) δ 12.36 (s, 1H), 11.76 (s, 1H), 7.99 (s, 1H), 7.69 (d, *J* = 8.2 Hz, 1H), 7.52 – 7.46 (m, 1H), 6.24 (d, *J* = 7.1 Hz, 1H), 5.82 (d, *J* = 3.5 Hz, 1H), 4.70 (d, *J* = 7.8 Hz, 1H), 4.01 (dd, *J* = 14.1, 7.0 Hz, 1H), 2.27 (s, 3H). **^{19}F NMR** (376 MHz, DMSO-d_6) δ −73.07 (d, *J* = 7.2 Hz). **^{13}C NMR** (101 MHz, DMSO-d_6) (representative peaks) δ 138.18, 127.44, 127.12, 126.17, 124.28, 121.46 (d, *J* = 1.1 Hz), 120.77, 71.90 (q, *J* = 13.3 Hz). **HRMS** (ESI) m/z: [M − H]⁻ Calcd for $C_{16}H_{11}O_3N_3Cl_2F_3S$ 451.98558; Found 451.98536 (− 0.48 ppm)

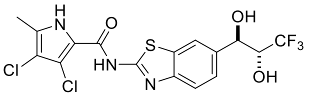

Chemical Formula: $C_{16}H_{12}Cl_2F_3N_3O_3S$
Molecular Weight: 454,25

6 Determination of stereomeric ratios

6.1 Determination of diastereomeric ratios by ^{19}F NMR

$NaBH_4$ reduction products were used as a standard, and for the DKR-ATH products, the stereomeric ratios were determined for crude, non-recrystallized products, preferably directly after extraction.

3a. 3,3,3-trifluoro-1-phenylpropane-1,2-diol

Top: $NaBH_4$ reduction, *anti*/*syn* = 97:3; **Middle:** (*S*,*S*)-**C4**-cat. DKR-ATH, *syn*/*anti* = 96:4; **Bottom:** (*S*,*S*)-**C5**-cat. DKR-ATH, *syn*/*anti* = 97:3

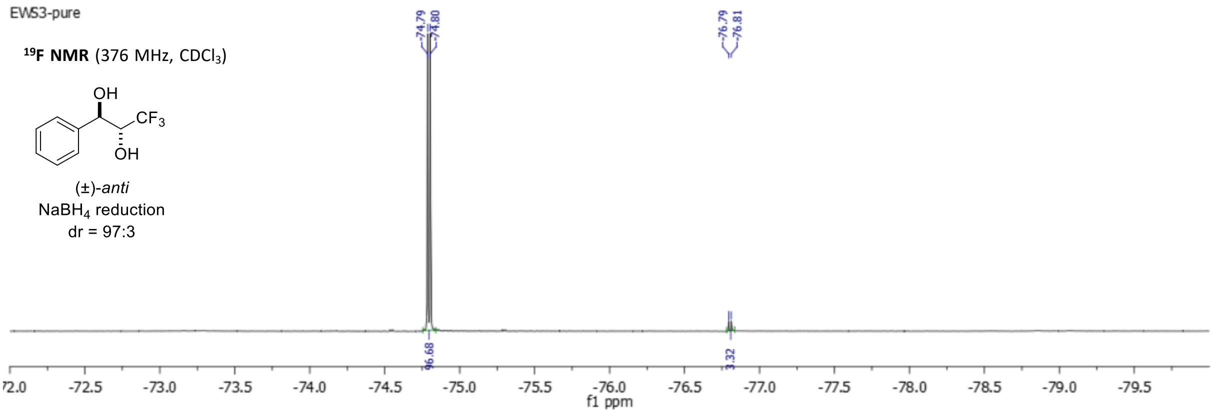

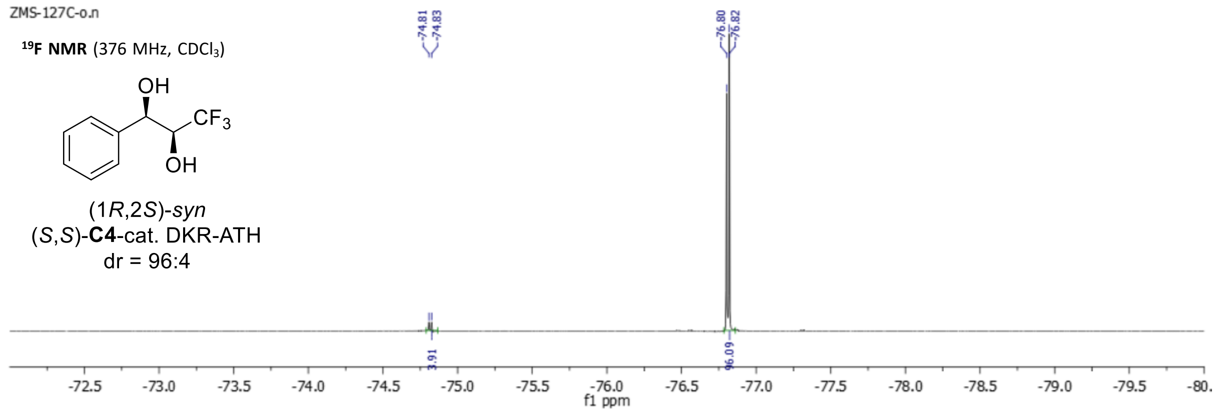

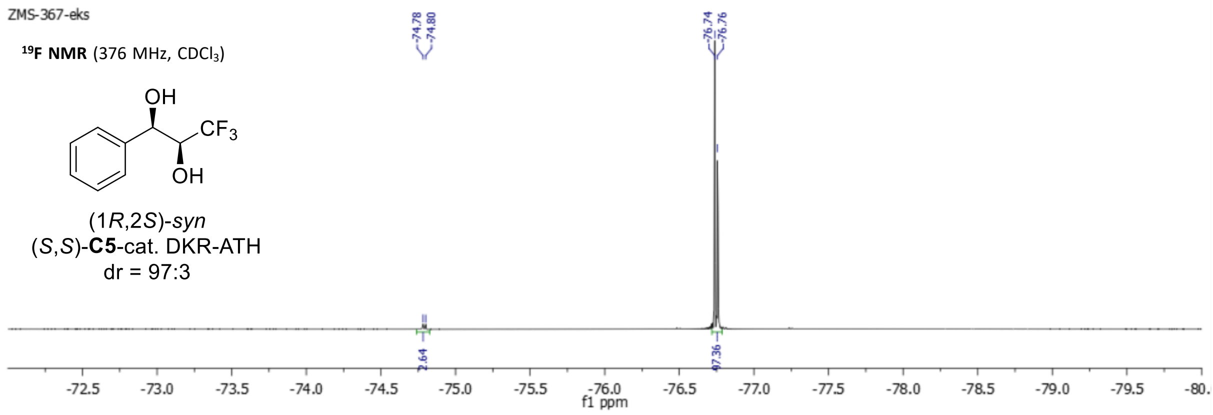

3b. 1-(4-bromophenyl)-3,3,3-trifluoropropane-1,2-diol

Top: $NaBH_4$ reduction, *anti*/*syn* = 86:14; **Middle:** (*S*,*S*)-**C4**-cat. DKR-ATH, *syn*/*anti* = 96:4; **Bottom:** (*S*,*S*)-**C5**-cat. DKR-ATH, *syn*/*anti* = 97:3

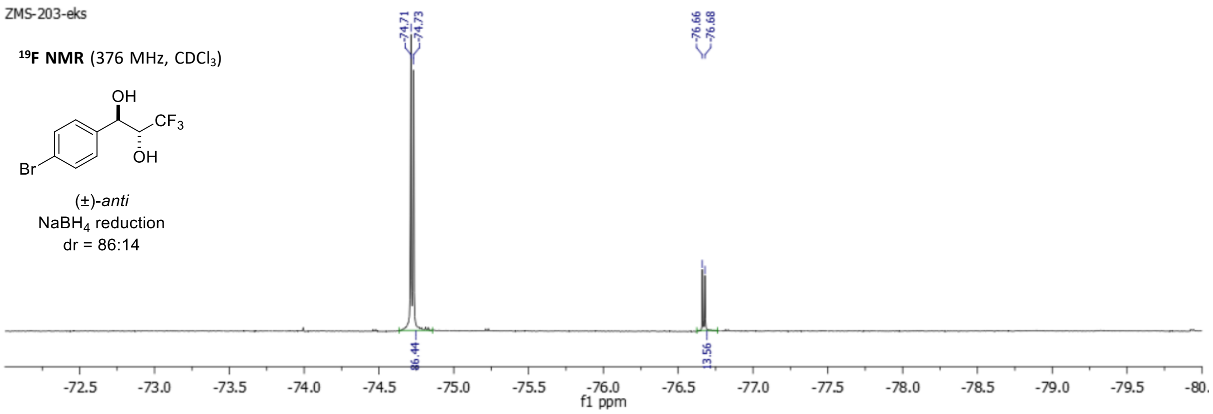

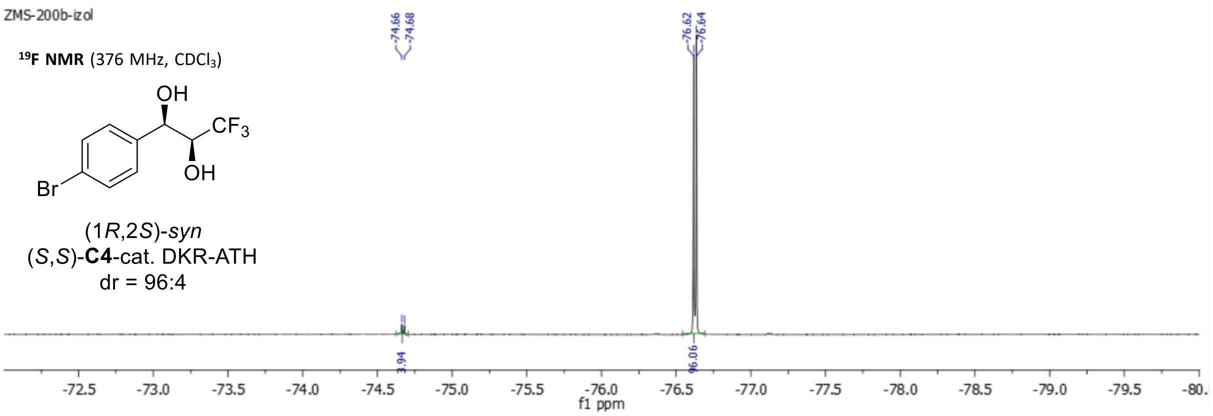

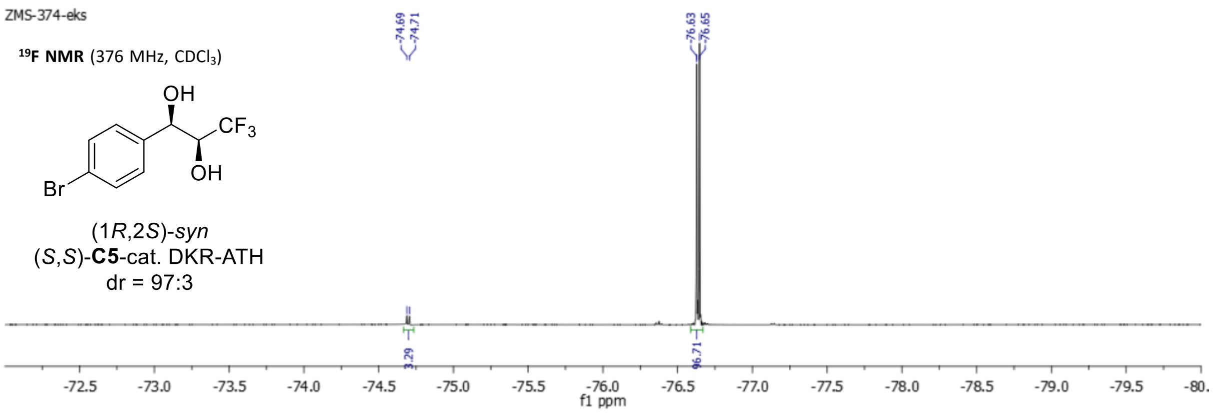

3c. 1-(4-chlorophenyl)-3,3,3-trifluoropropane-1,2-diol

Top: $NaBH_4$ reduction, *anti*/*syn* = 89:11; **Middle:** (*S*,*S*)-**C4**-cat. DKR-ATH, *syn*/*anti* = 95:5; **Bottom:** (*S*,*S*)-**C5**-cat. DKR-ATH, *syn*/*anti* = 96:4

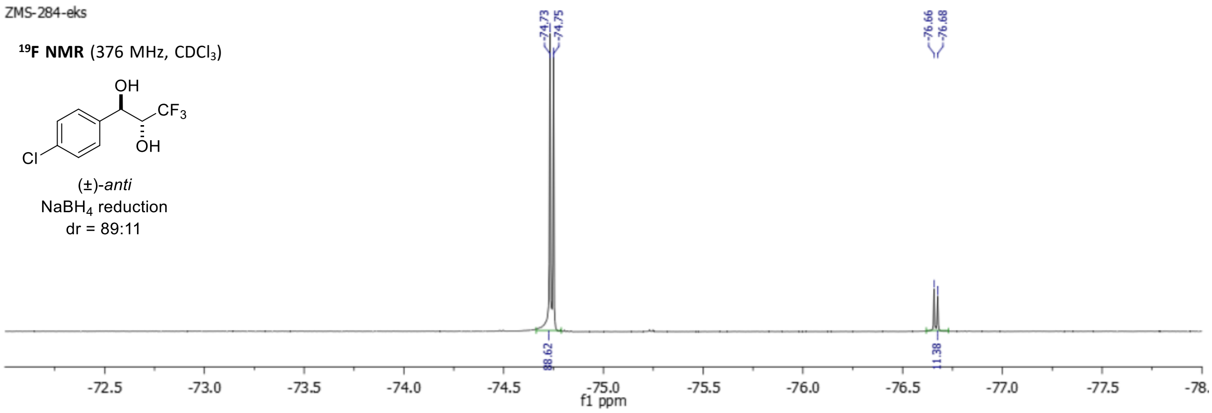

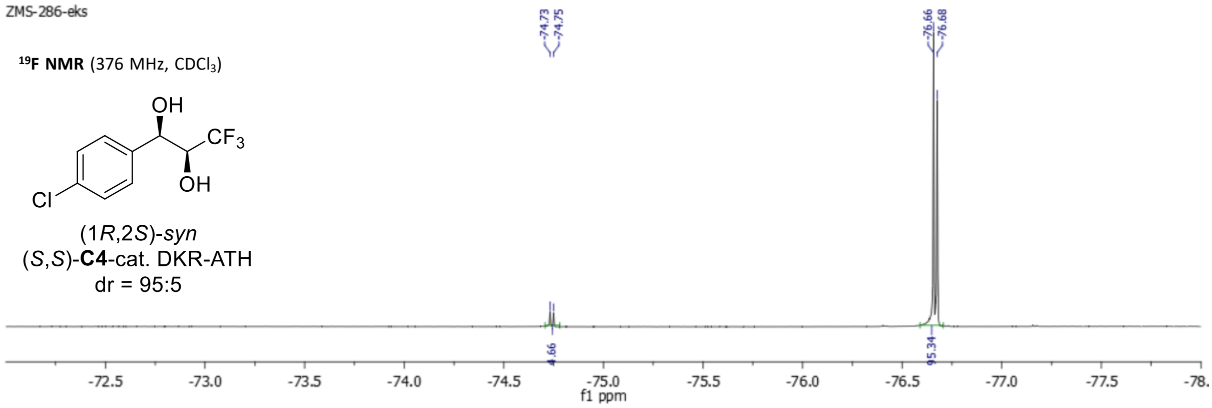

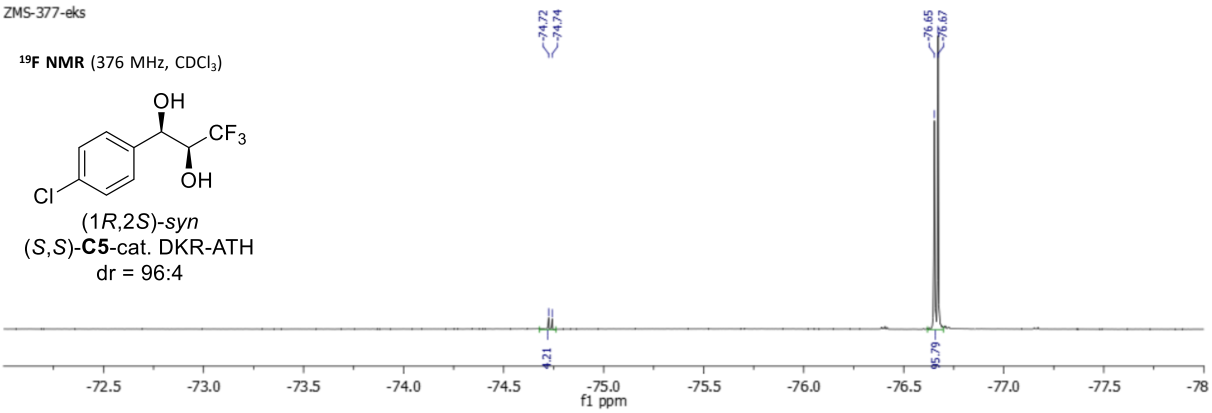

3d. 3,3,3-trifluoro-1-(4-fluorophenyl)propane-1,2-diol

Top: $NaBH_4$ reduction, *anti*/*syn* = 91:9; **Middle:** (*S*,*S*)-**C4**-cat. DKR-ATH, *syn*/*anti* = 95:5; **Bottom:** (*S*,*S*)-**C5**-cat. DKR-ATH, *syn*/*anti* = 96:4

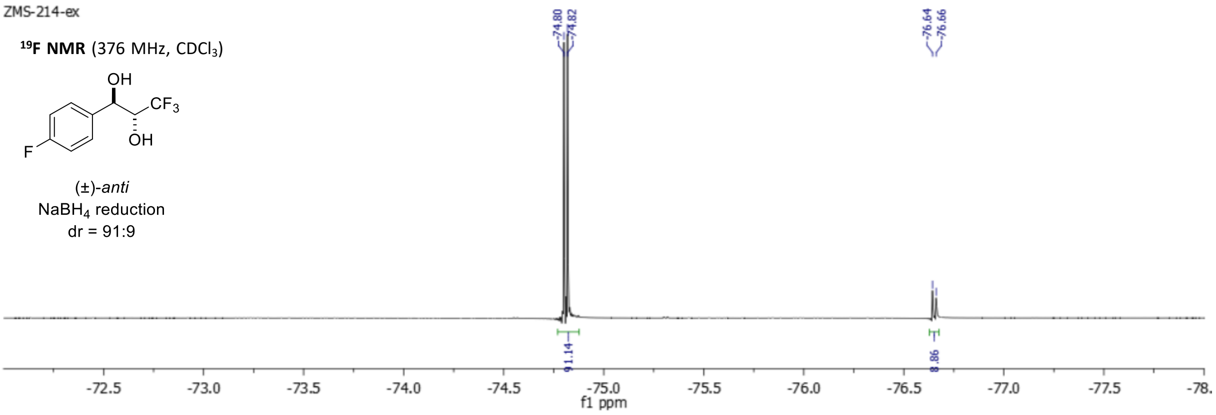

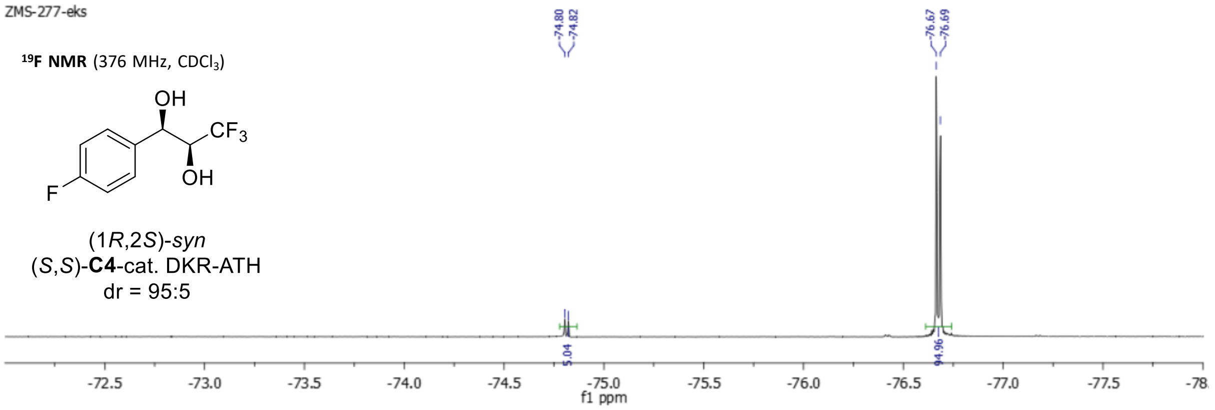

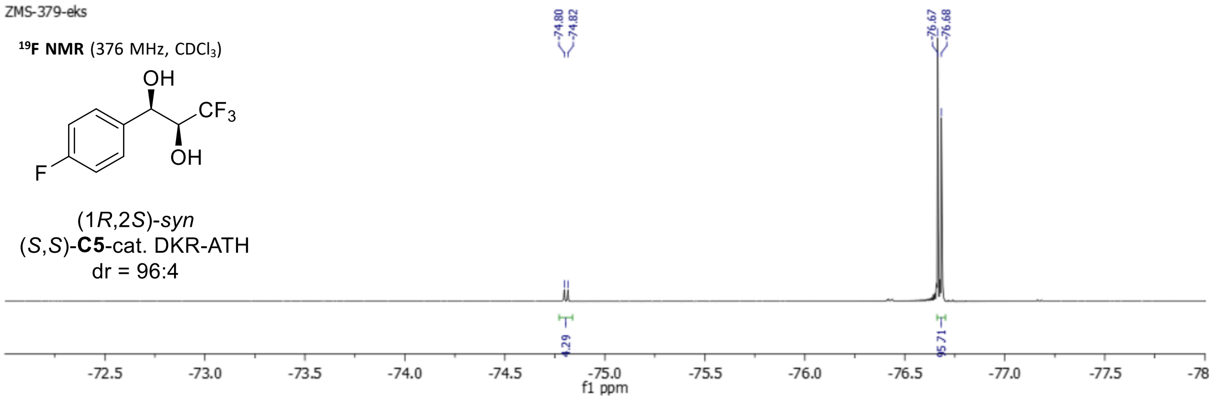

3e. 3,3,3-trifluoro-1-(4-nitrophenyl)propane-1,2-diol

Top: $NaBH_4$ reduction, *anti*/*syn* = 67:33; **Middle:** (*S*,*S*)-**C4**-cat. DKR-ATH, *syn*/*anti* = 95:5; **Bottom:** (*S*,*S*)-**C5**-cat. DKR-ATH, *syn*/*anti* = 94:6

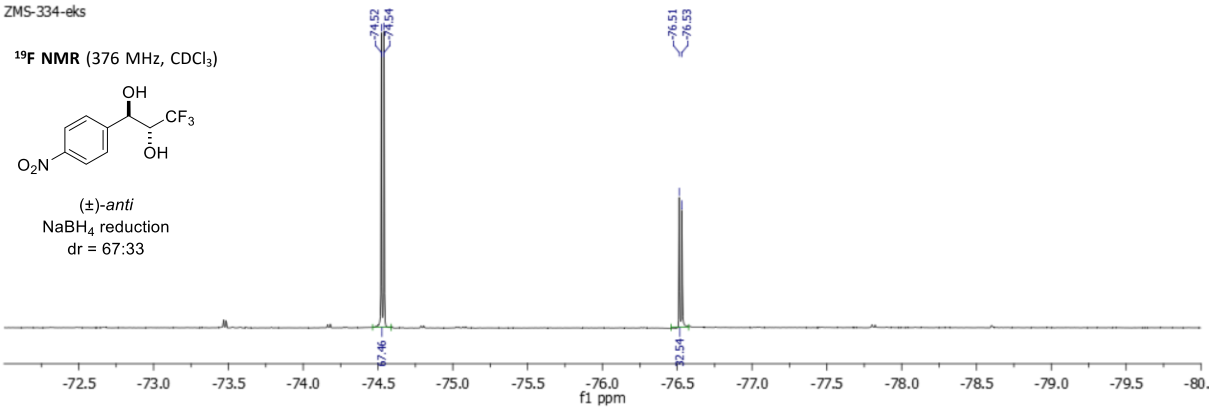

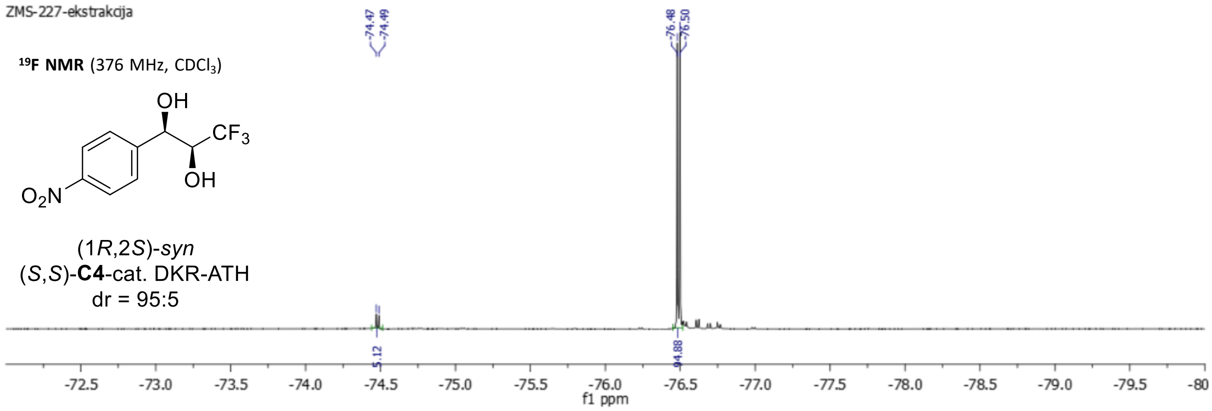

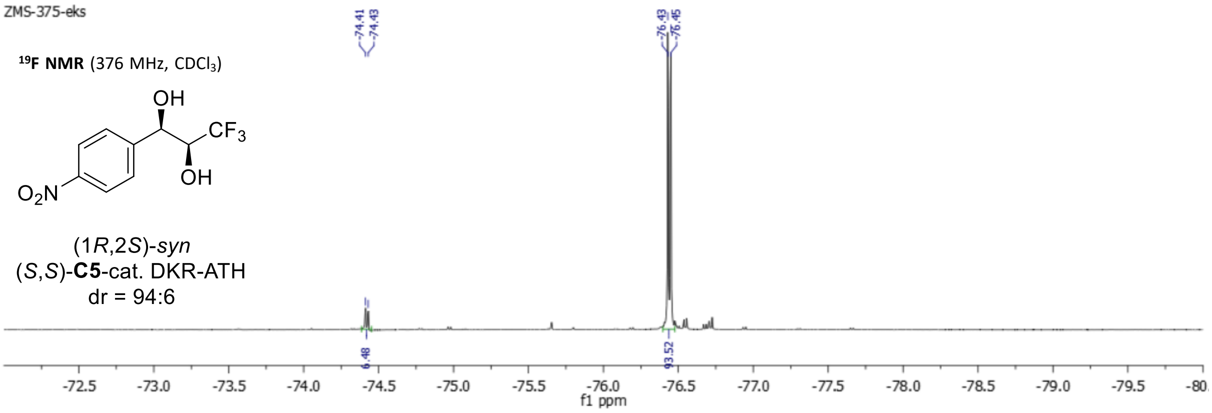

3f. 3,3,3-trifluoro-1-(3-hydroxy-4-nitrophenyl)propane-1,2-diol

Top: $NaBH_4$ reduction, *anti*/*syn* = 96:4; **Middle:** (*S*,*S*)-**C4**-cat. DKR-ATH, *syn*/*anti* = 96:4; **Bottom:** (*S*,*S*)-**C5**-cat. DKR-ATH, *syn*/*anti* = 96:4

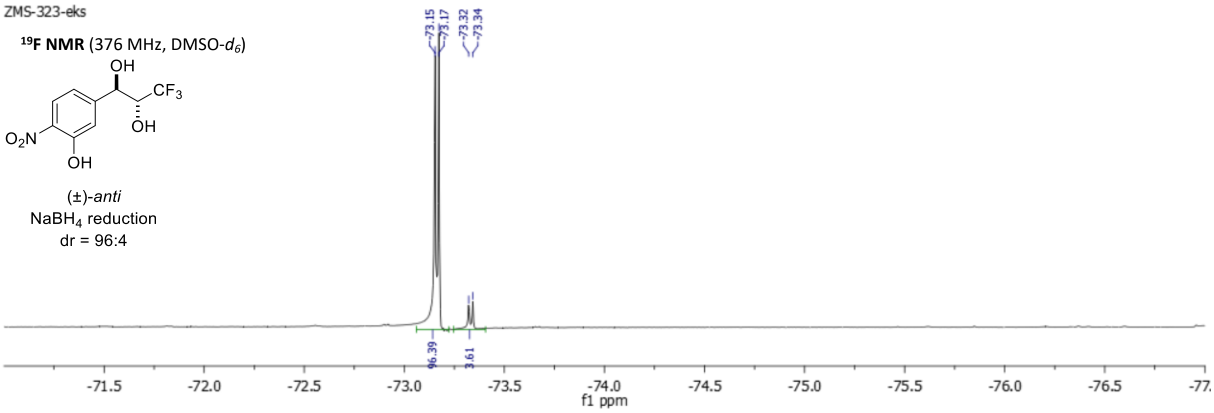

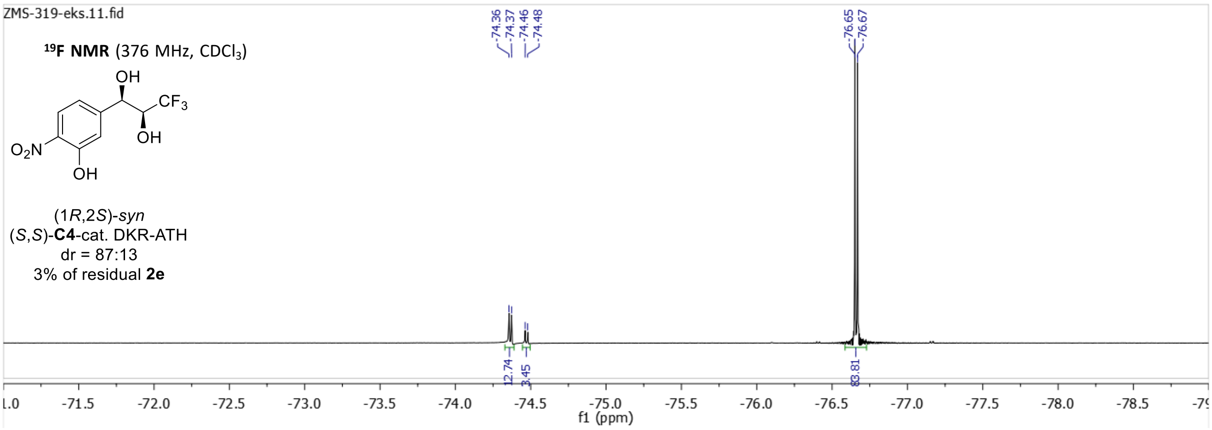

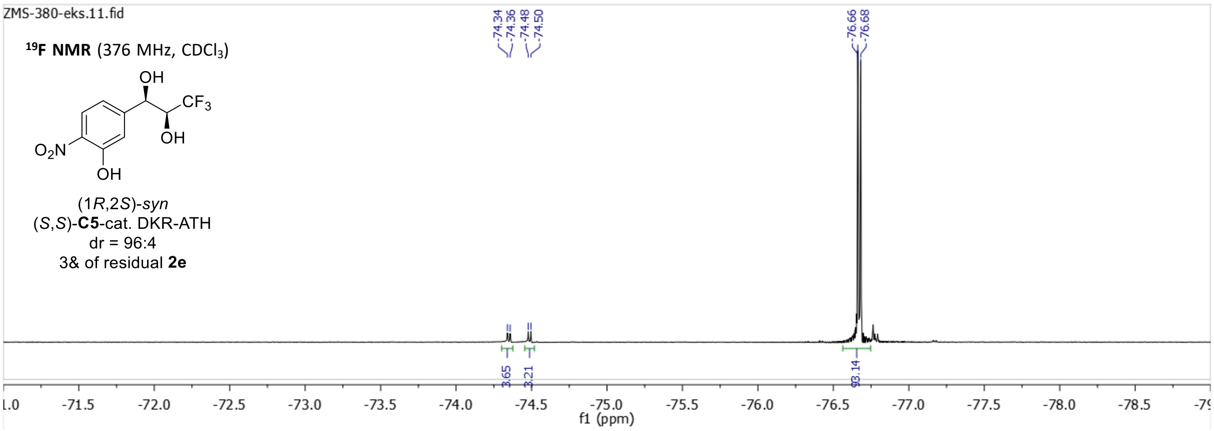

3g. 3,3,3-trifluoro-1-(4-hydroxyphenyl)propane-1,2-diol

Top: $NaBH_4$ reduction, *anti/syn* = 89:11; **Top Middle:** (*S*,*S*)-**C4**-cat. DKR-ATH, *syn/anti* = 84:16; **Bottom Middle:** (*S*,*S*)-**C4**-cat. DKR-ATH, *syn/anti* = 84:16; **Bottom:** (*S*,*S*)-**C5**-cat. DKR-ATH, *syn/anti* = 92:8

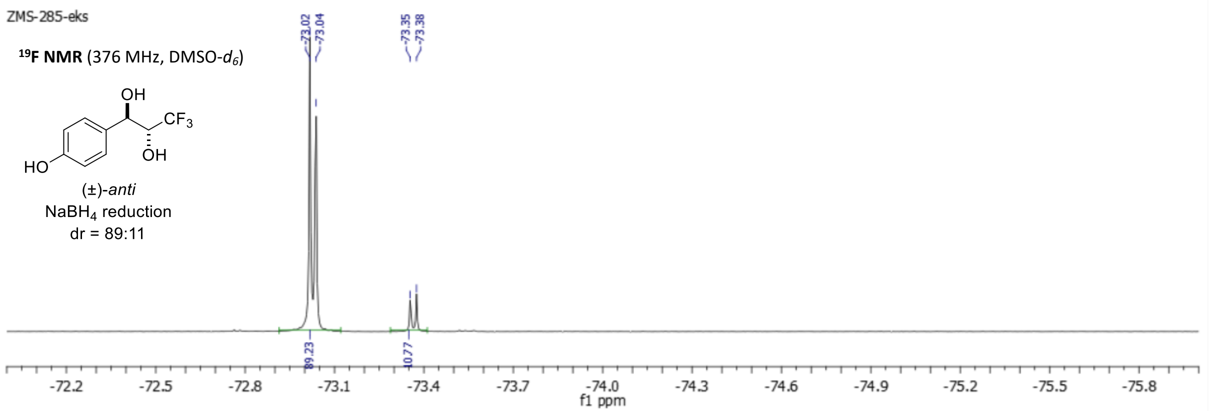

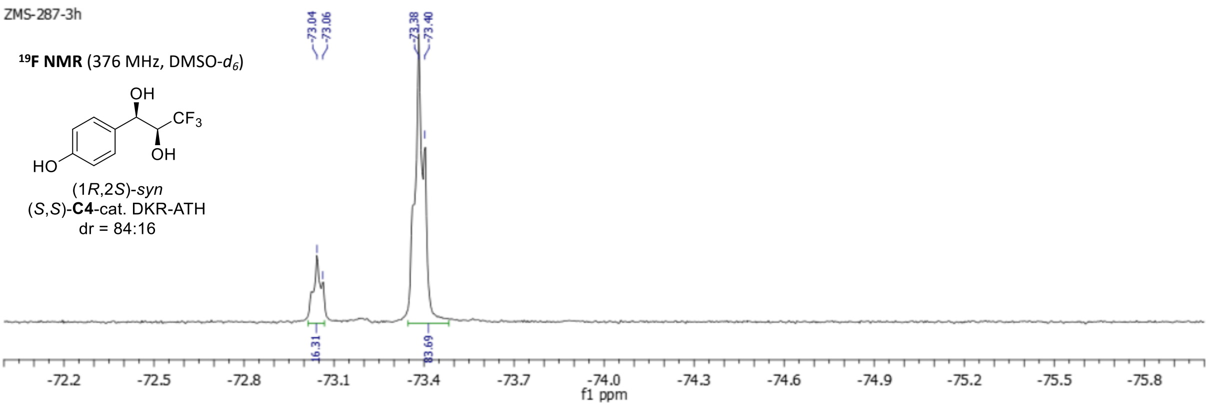

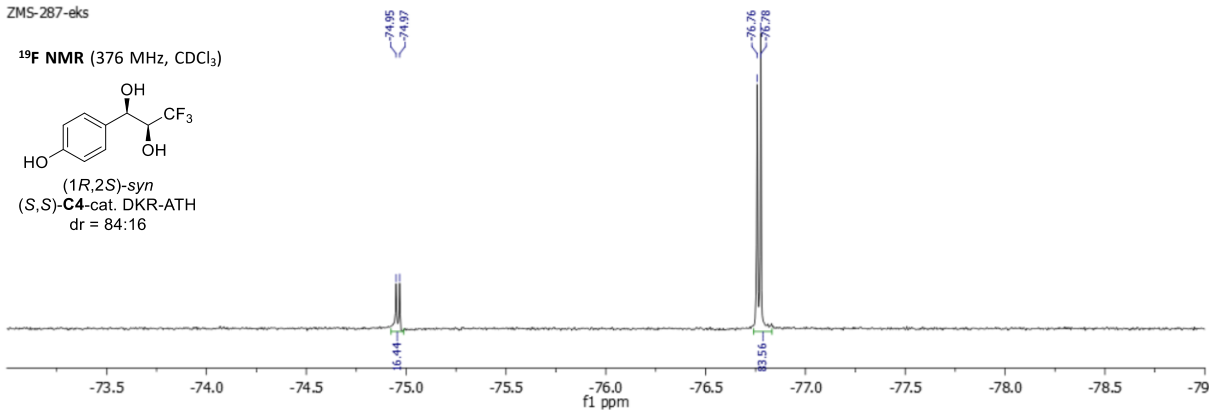

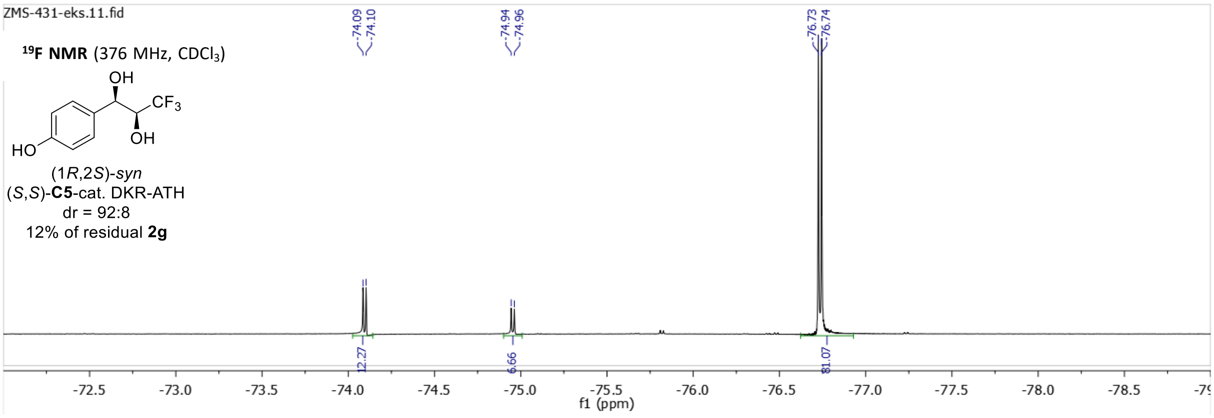

3h. 3,3,3-trifluoro-1-(3-hydroxyphenyl)propane-1,2-diol

Top: $NaBH_4$ reduction, *anti*/*syn* = 93:7; **Middle:** (*S*,*S*)-**C4**-cat. DKR-ATH, *syn*/*anti* = 93:7; **Bottom:** (*S*,*S*)-**C5**-cat. DKR-ATH, *syn*/*anti* = 96:4

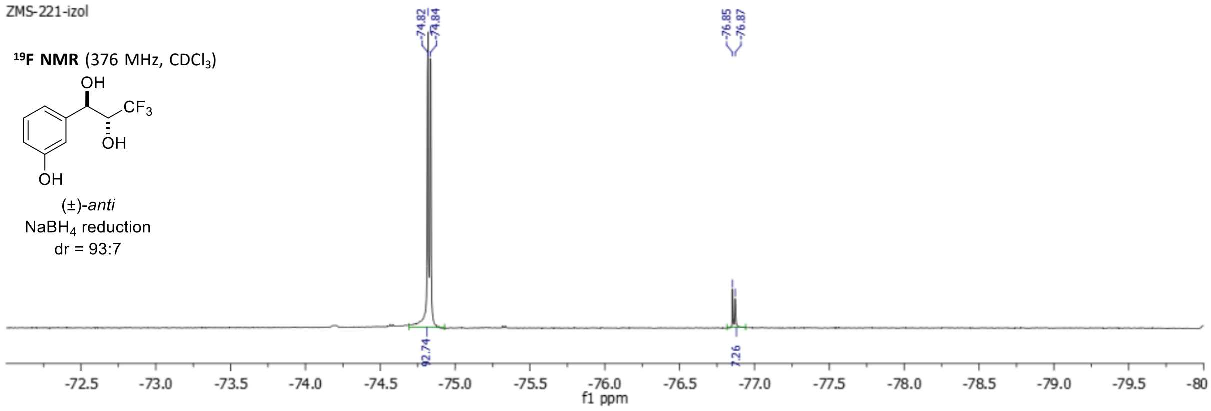

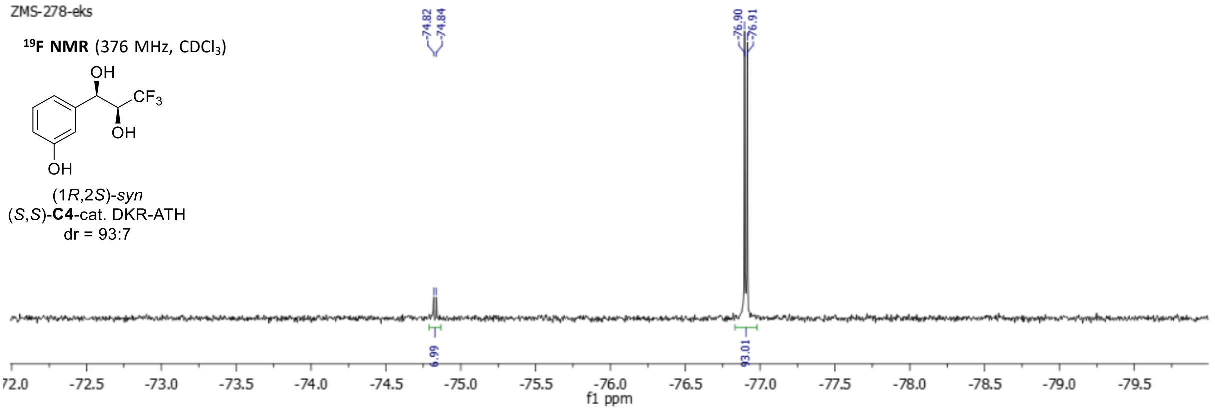

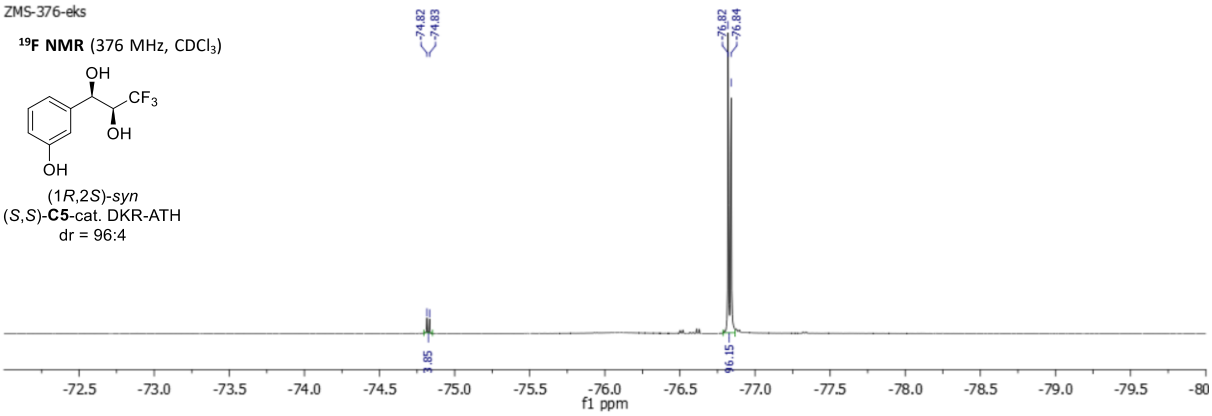

3i. 3,3,3-trifluoro-1-(2-hydroxyphenyl)propane-1,2-diol

Top: $NaBH_4$ reduction, *anti*/*syn* = 91:9; **Middle:** (*S*,*S*)-**C4**-cat. DKR-ATH, *syn*/*anti* = 87:13; **Bottom:** (*S*,*S*)-**C5**-cat. DKR-ATH, *syn*/*anti* = 69:31

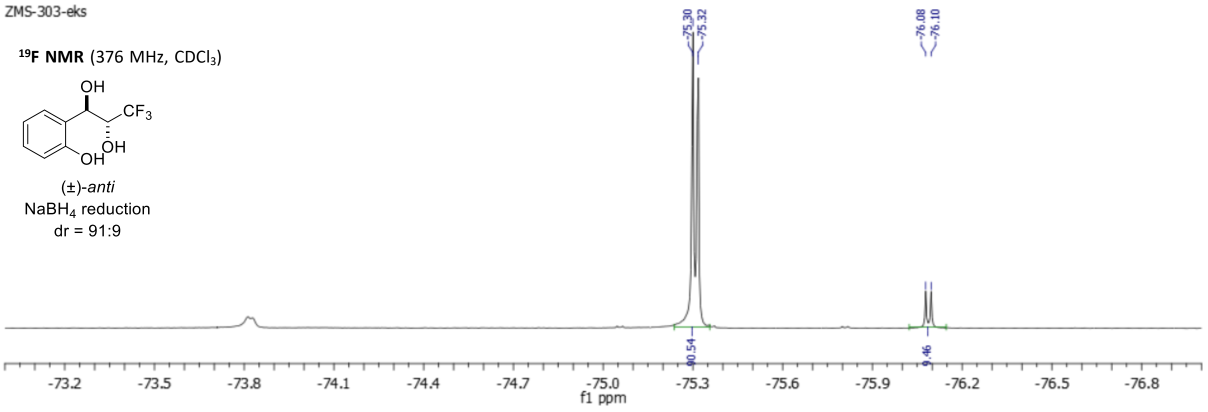

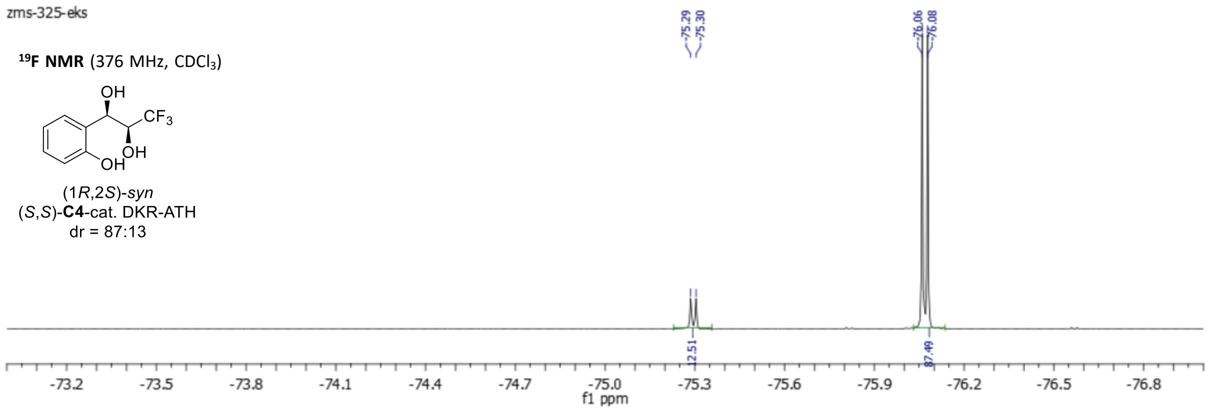

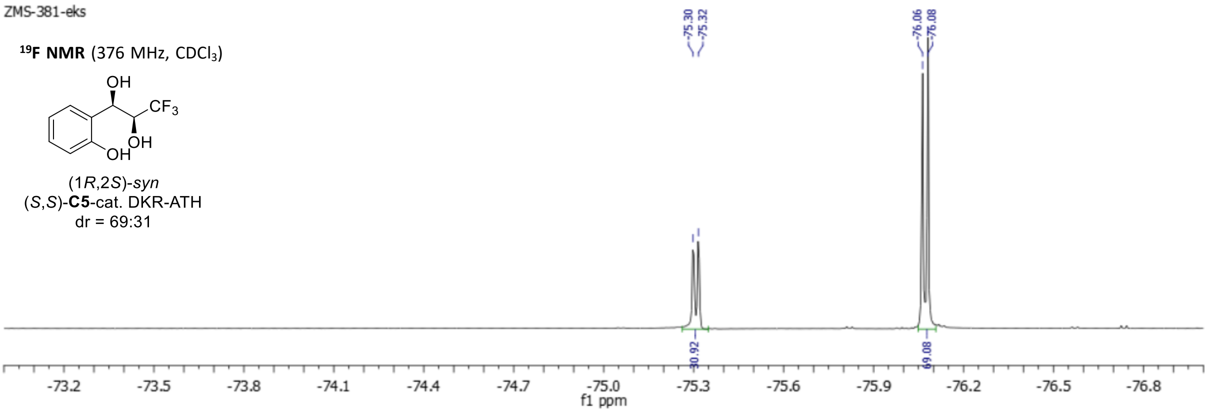

3j. 3,3,3-trifluoro-1-(4-methoxyphenyl)propane-1,2-diol

Top: $NaBH_4$ reduction, *anti*/*syn* = 97:3; **Middle:** (*S*,*S*)-**C4**-cat. DKR-ATH, *syn*/*anti* = 95:5; **Bottom:** (*S*,*S*)-**C5**-cat. DKR-ATH, *syn*/*anti* = 96:4

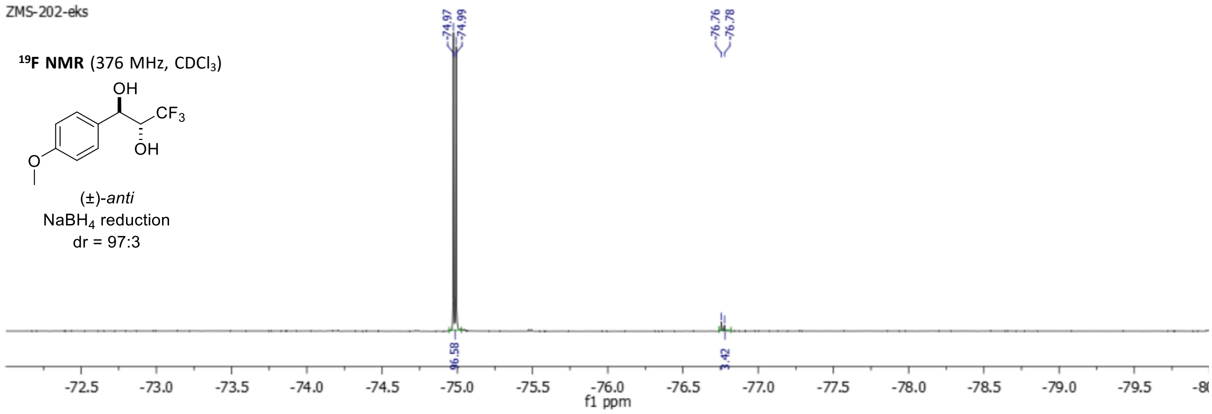

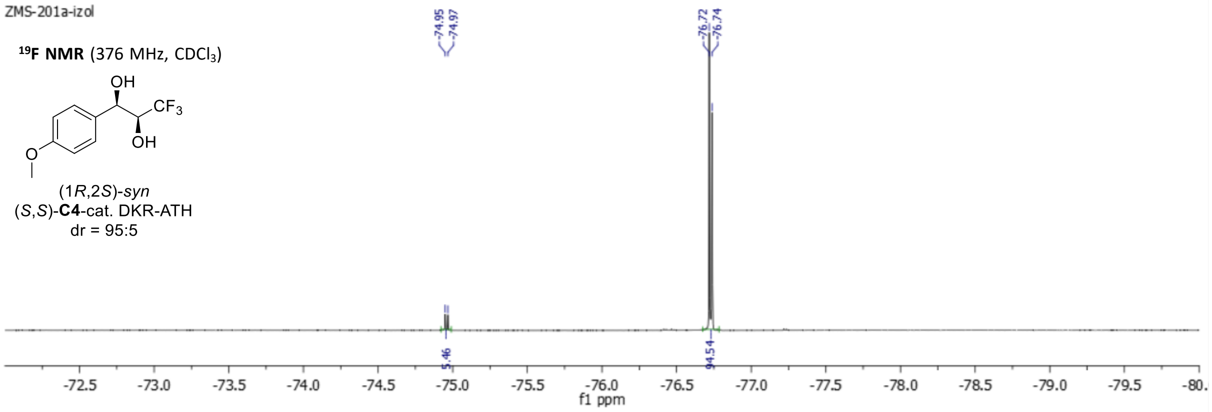

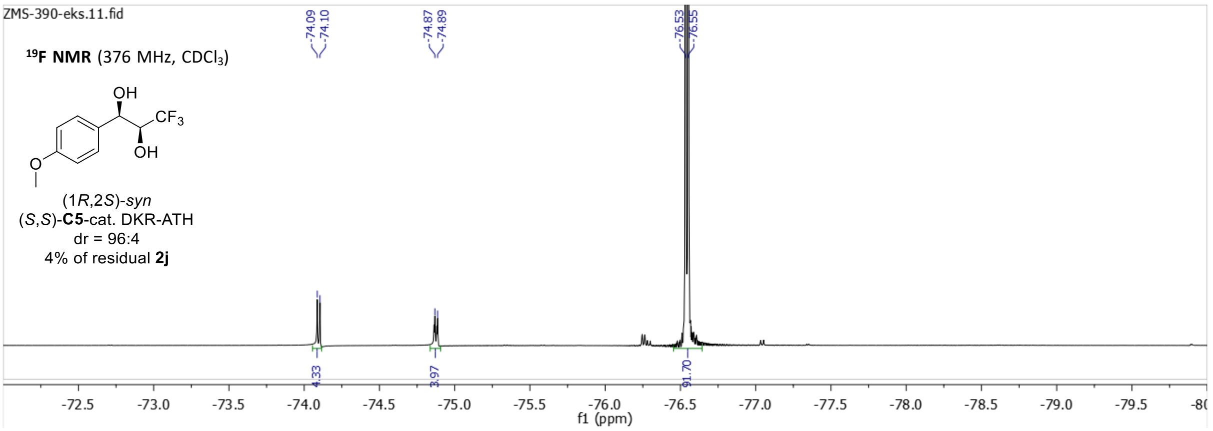

3k. 3,3,3-trifluoro-1,2-dihydroxypropyl)phenyl acetate

Top: (*S*,*S*)-**C4**-cat. DKR-ATH, *syn*/*anti* = 96:4; **Bottom:** (*S*,*S*)-**C5**-cat. DKR-ATH, *syn*/*anti* = 98:2

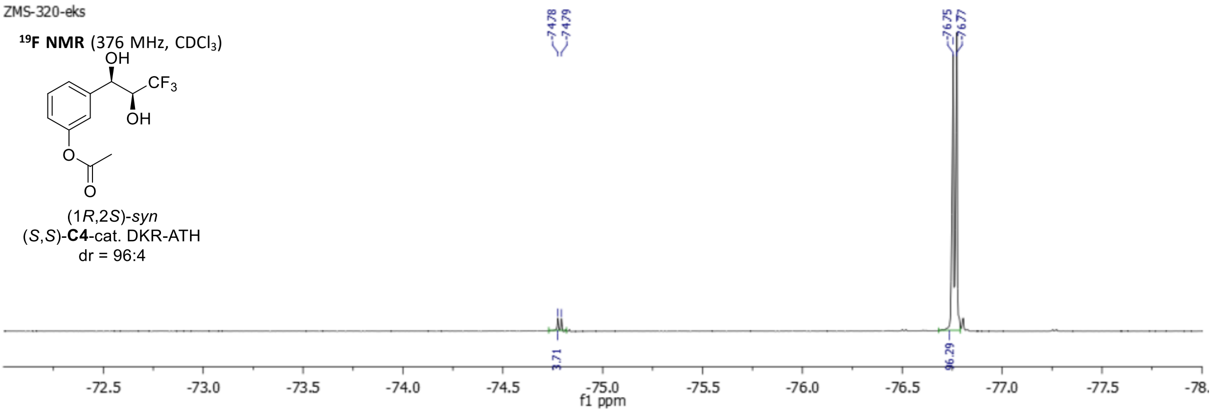

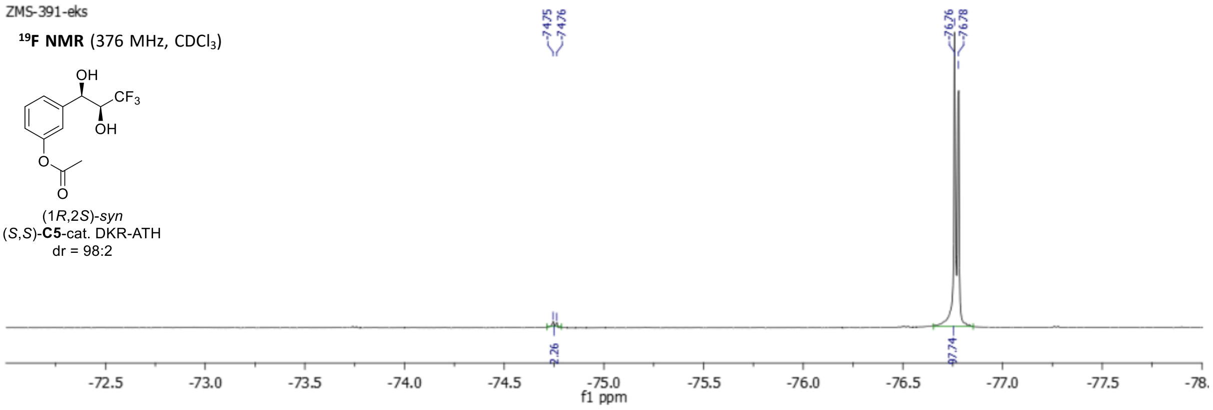

3l. 3,3,3-trifluoro-1-(4-(hydroxymethyl)phenyl)propane-1,2-diol

Top: $NaBH_4$ reduction, *anti*/*syn* = 96:4; **Middle:** (*S*,*S*)-**C5**-cat. DKR-ATH, *syn*/*anti* = 93:7 **Bottom:** (*S*,*S*)-**C4**-cat. DKR-ATH, *syn*/*anti* = 75:25

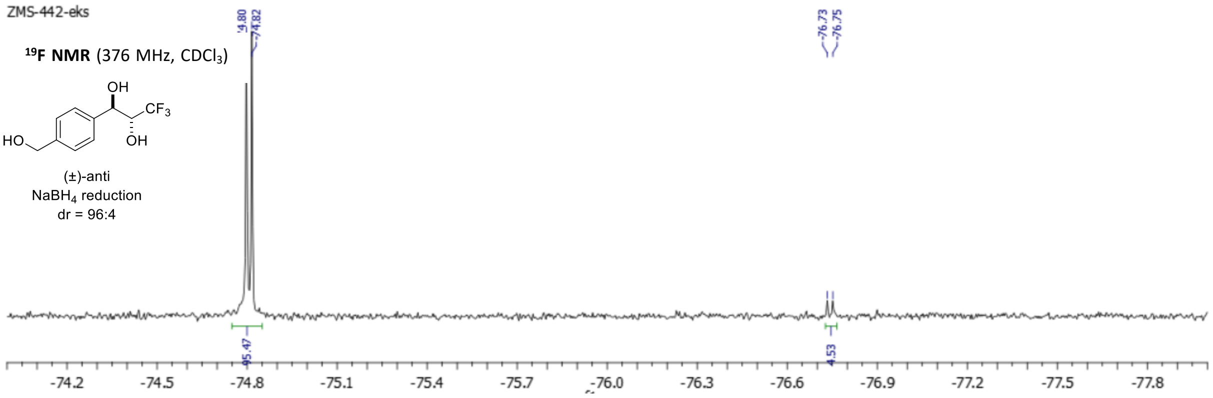

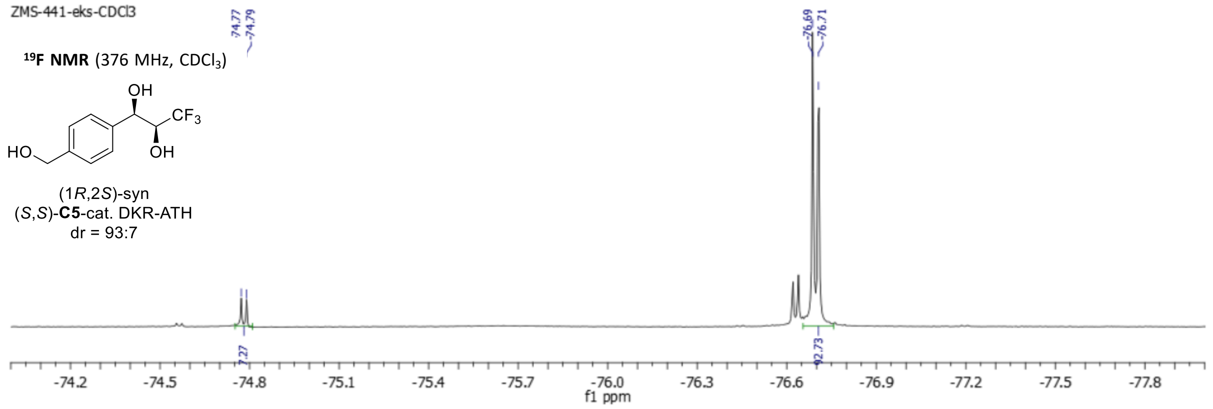

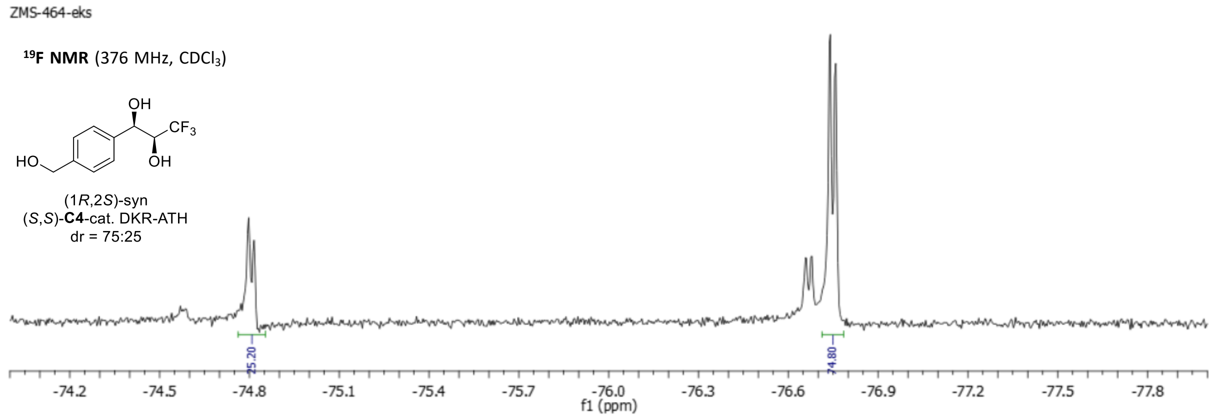

3m. 3,3,3-trifluoro-1-(pyridin-3-yl)propane-1,2-diol

Top: (*S*,*S*)-**C4**-cat. DKR-ATH, *syn*/*anti* = 92:8 (3 h, full conv.); **Bottom:** (*R*,*R*)-**C4**-cat. DKR-ATH, *syn*/*anti* = 92:8 (overnight reaction, decomposition occurs)

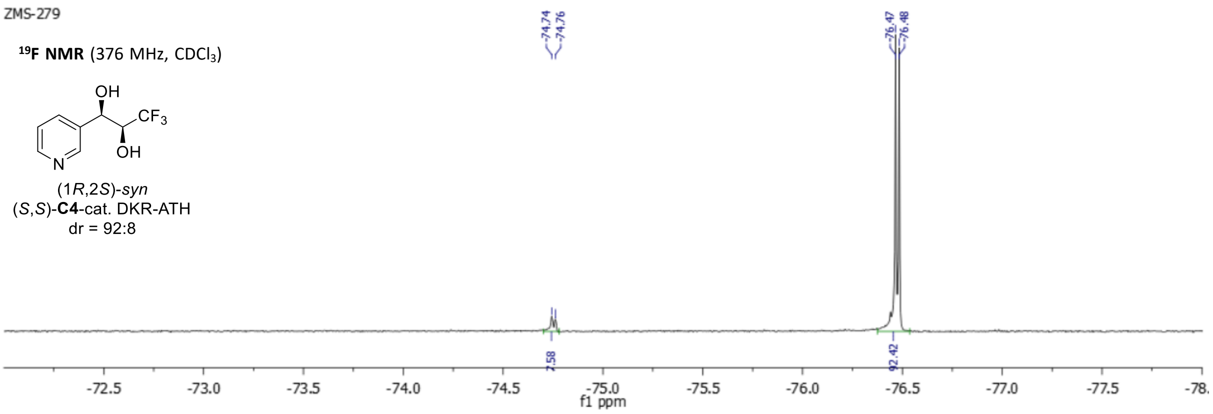

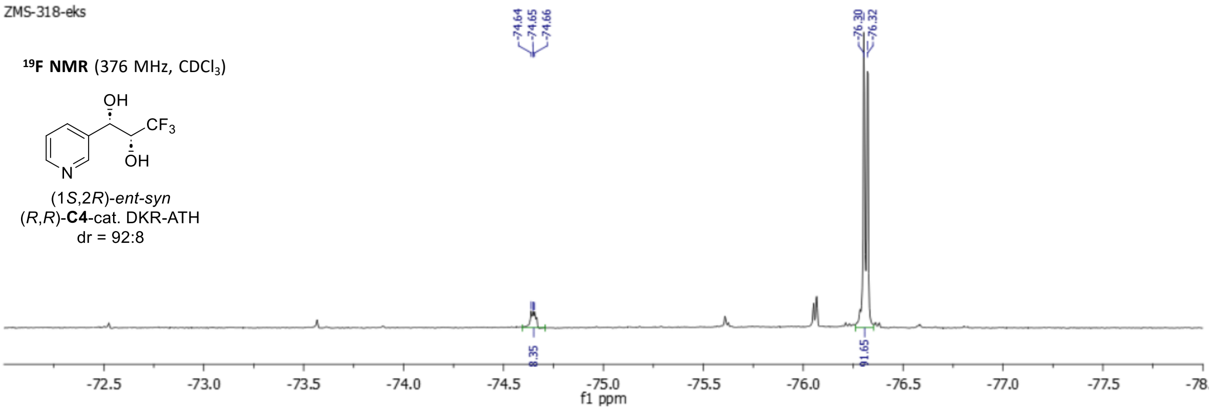

3n. 1-(2-aminobenzo[*d*]thiazol-6-yl)-3,3,3-trifluoropropane-1,2-diol

Top: $\mathbf{NaBH_4}$ reduction, *anti*/*syn* = 96:4; **Middle:** (*S*,*S*)-**C4**-cat. DKR-ATH, *syn*/*anti* = 91:9; **Bottom:** (*R*,*R*)-**C4**-cat. DKR-ATH, *syn*/*anti* = 92:8. **Bottom 2:** (*S*,*S*)-**C5**-cat. DKR-ATH, *syn*/*anti* = 96:4

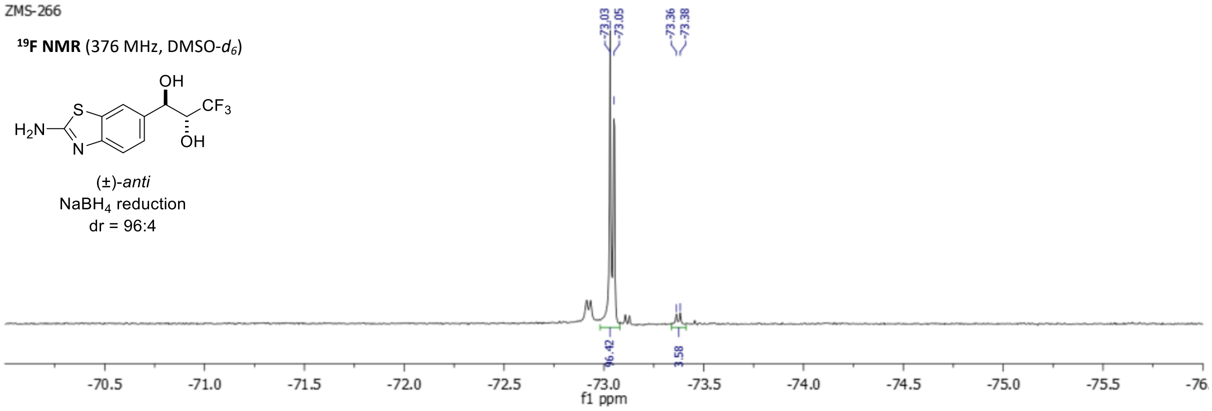

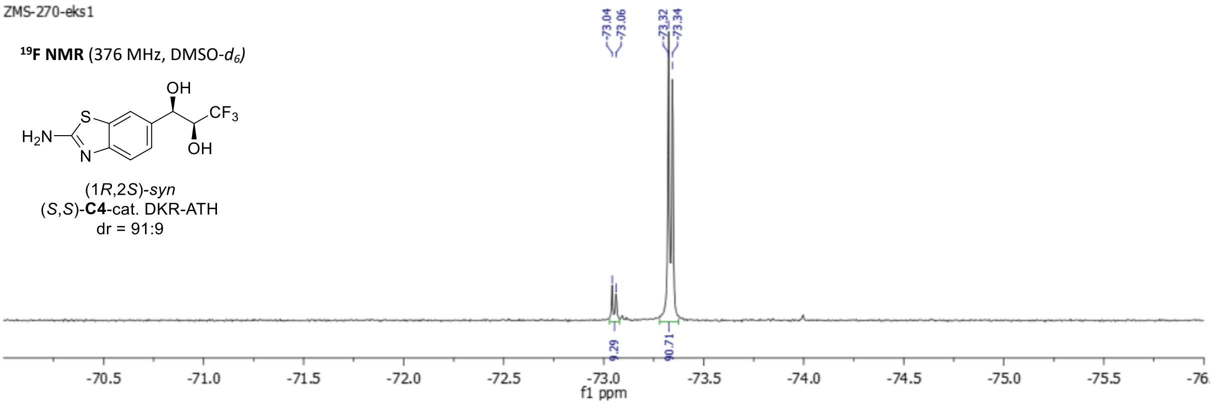

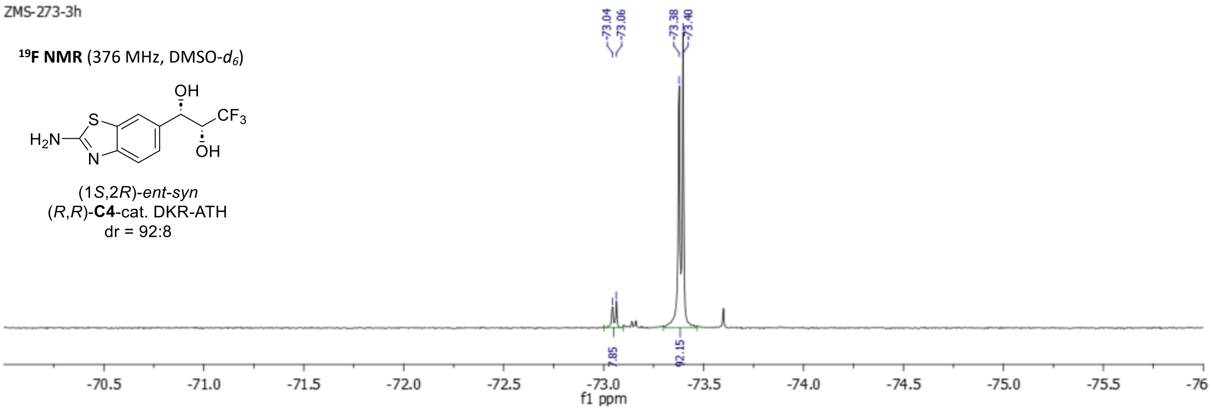

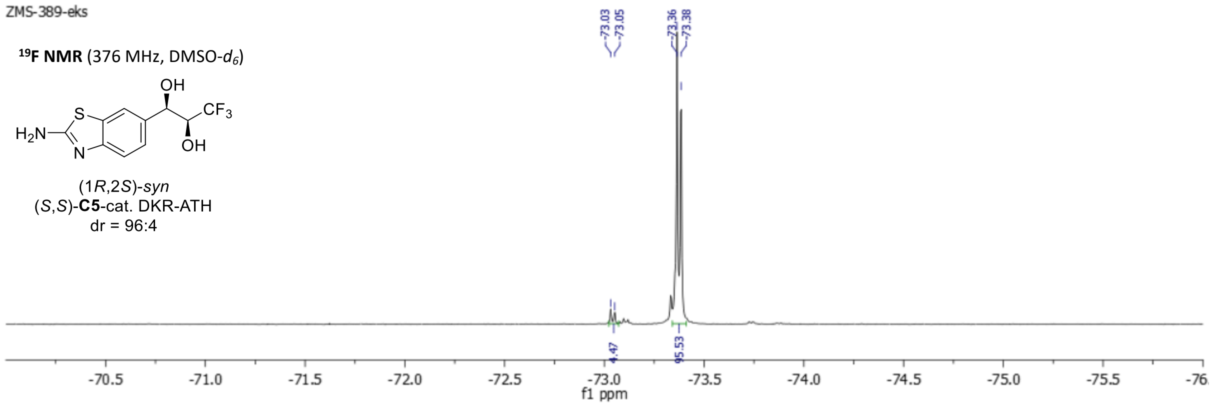

3o. 1,1,1-trifluoro-4-phenylbutane-2,3-diol

Top: NaBH$_4$ reduction, *anti*/*syn* = 65:35; **Middle:** (*S*,*S*)-**C4**-cat. DKR-ATH, *syn*/*anti* = 94:6; **Bottom:** (*S*,*S*)-**C5**-cat. DKR-ATH, *syn*/*anti* = 87:13

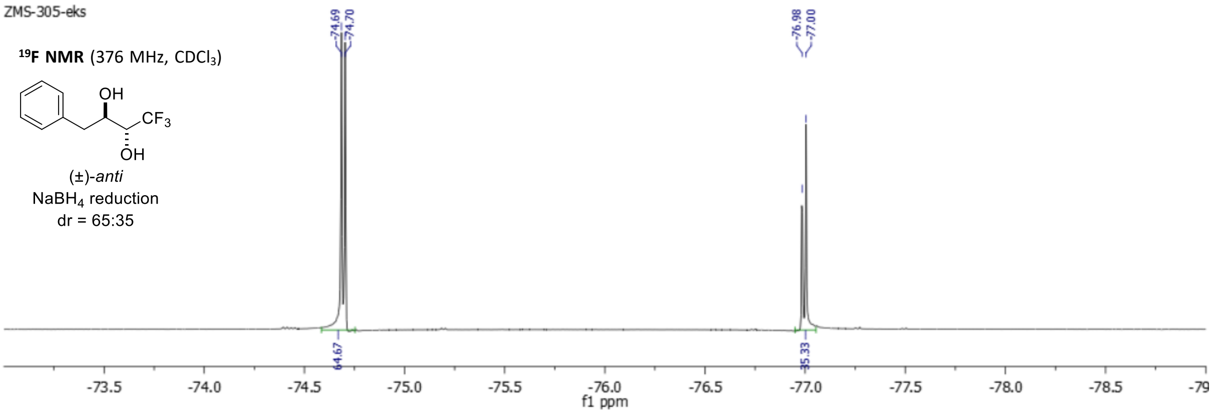

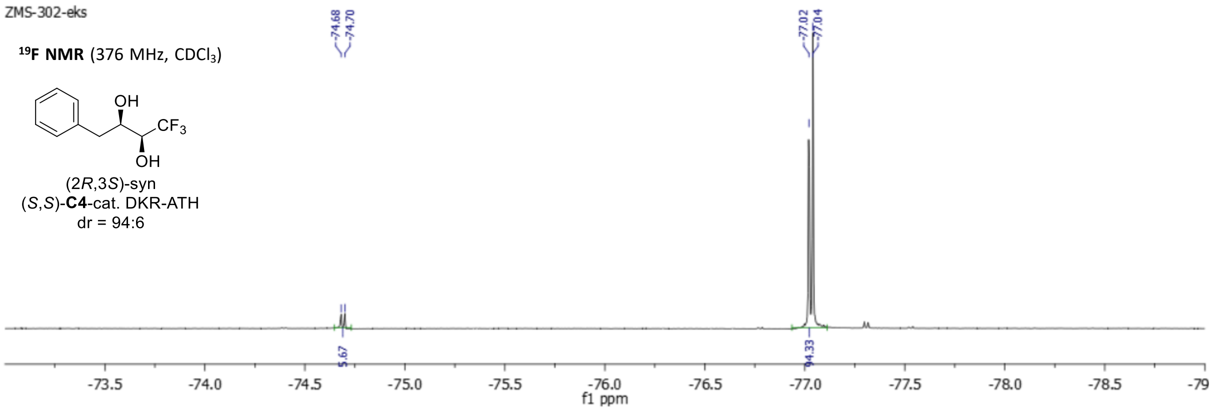

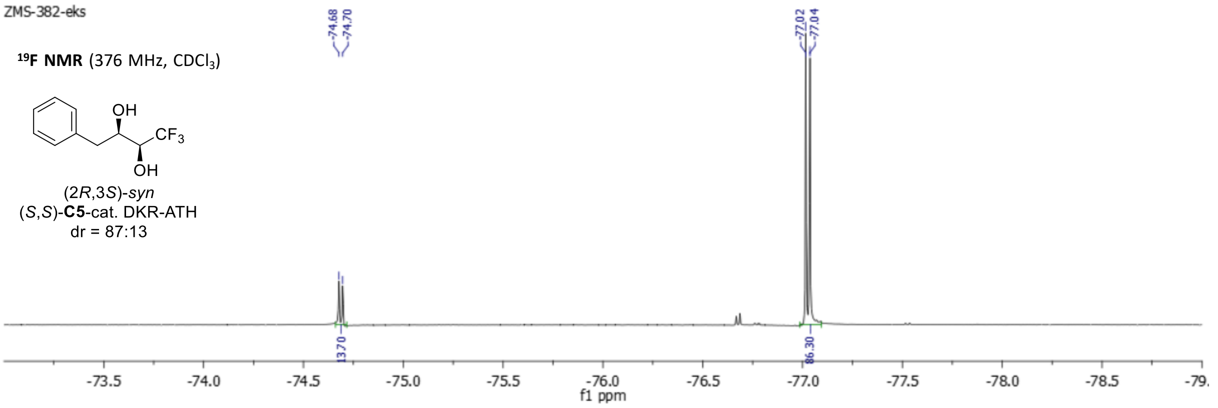

3p. 1,1,1-trifluorononane-2,3-diol

Top: $NaBH_4$ reduction, *anti*/*syn* = 69:31; **Middle:** (*S*,*S*)-**C4**-cat. DKR-ATH, *syn*/*anti* = 77:23; **Bottom:** (*S*,*S*)-**C5**-cat. DKR-ATH, *syn*/*anti* = 90:10

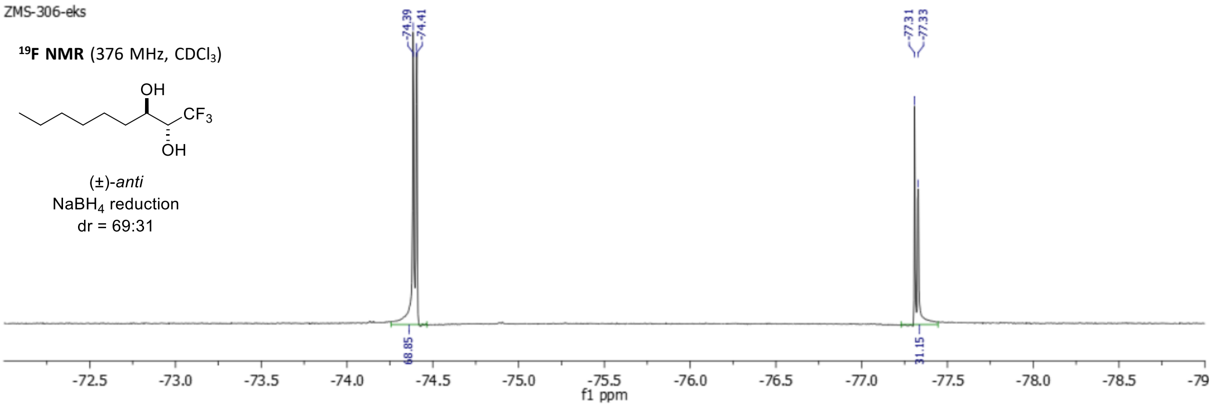

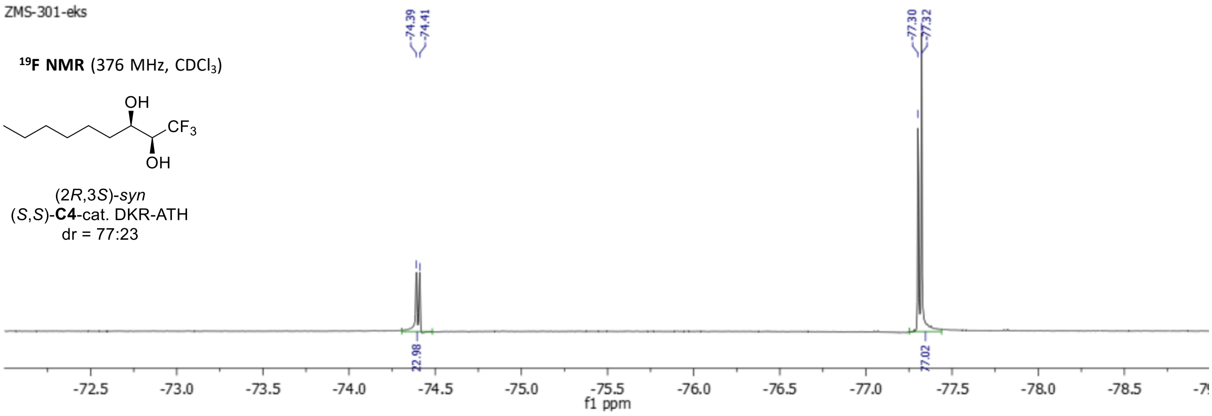

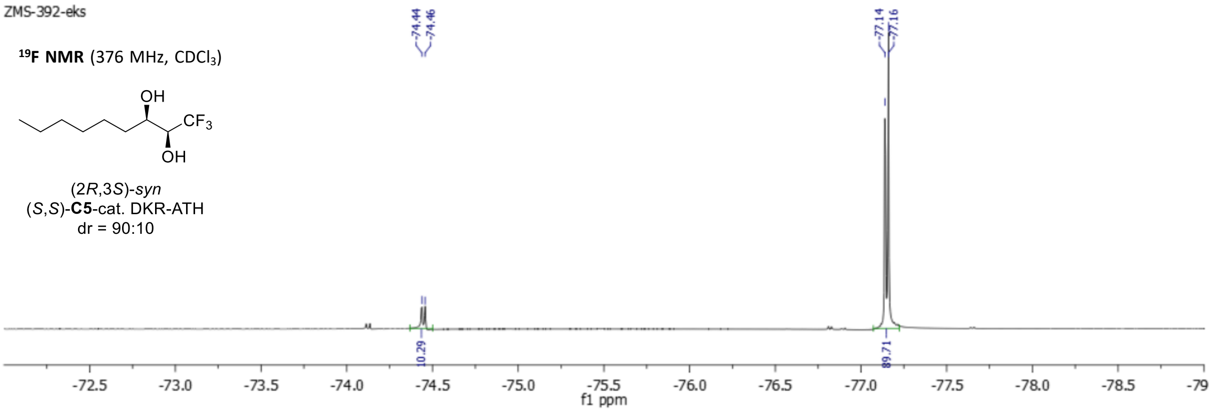

3q. 6,6,6-trifluoro-4,5-dihydroxyhexyl)benzamide

Top: NaBH$_4$ reduction, *anti*/*syn* = 65:35; **Bottom:** (*S*,*S*)-**C5**-cat. DKR-ATH, *syn*/*anti* = 92:8

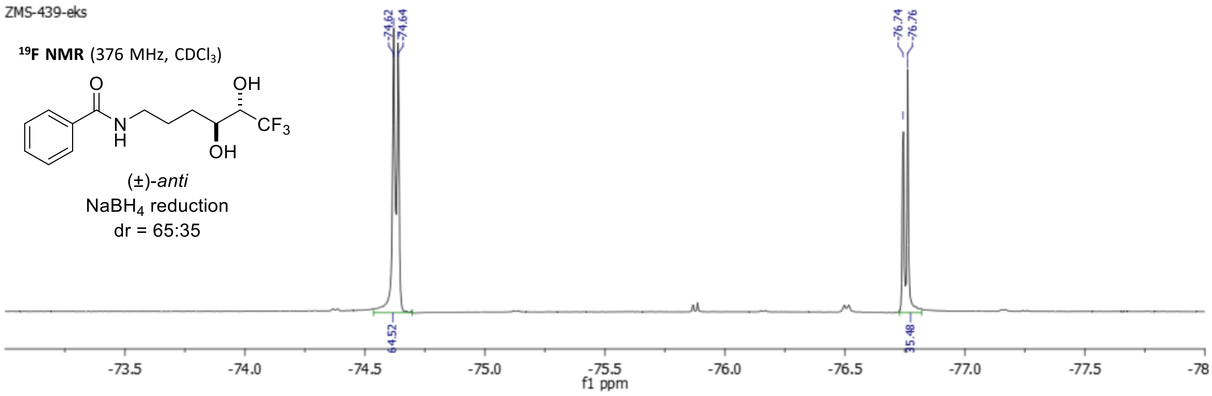

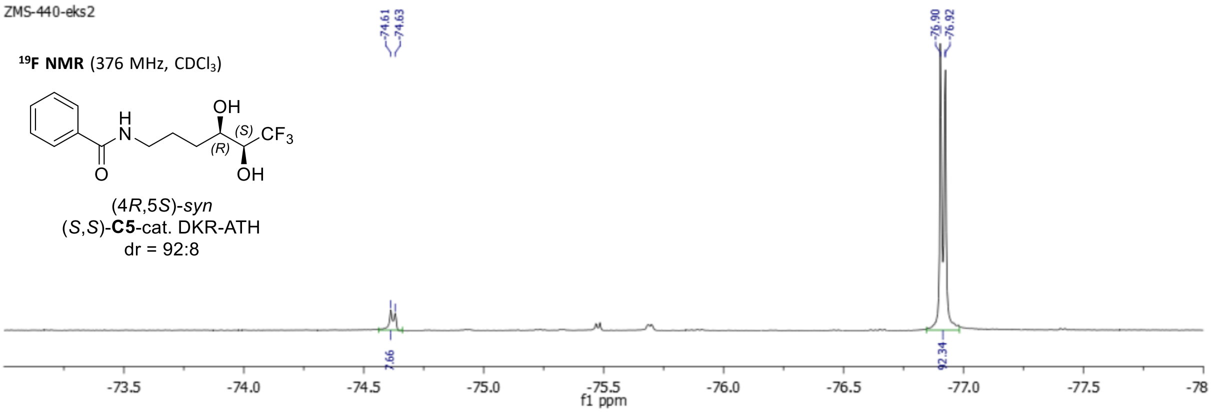

3r. 1,1,1-trifluoro-5-phenylpent-4-ene-2,3-diol.

Top: Impure fraction from flash chromatography of (*R*,*R*)-**C4**-cat. DKR-ATH, enriched in *anti*-**3r**. **Middle:** (*R*,*R*)-**C4**-cat. DKR-ATH, *syn*/*anti* = 96:4; **Bottom:** (*S*,*S*)-**C5**-cat. DKR-ATH, *syn*/*anti* = 97:3

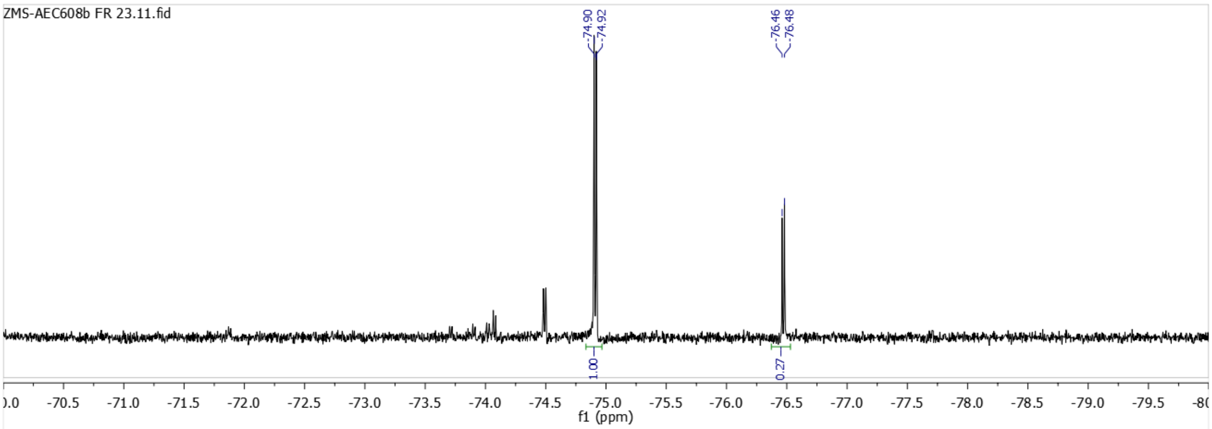

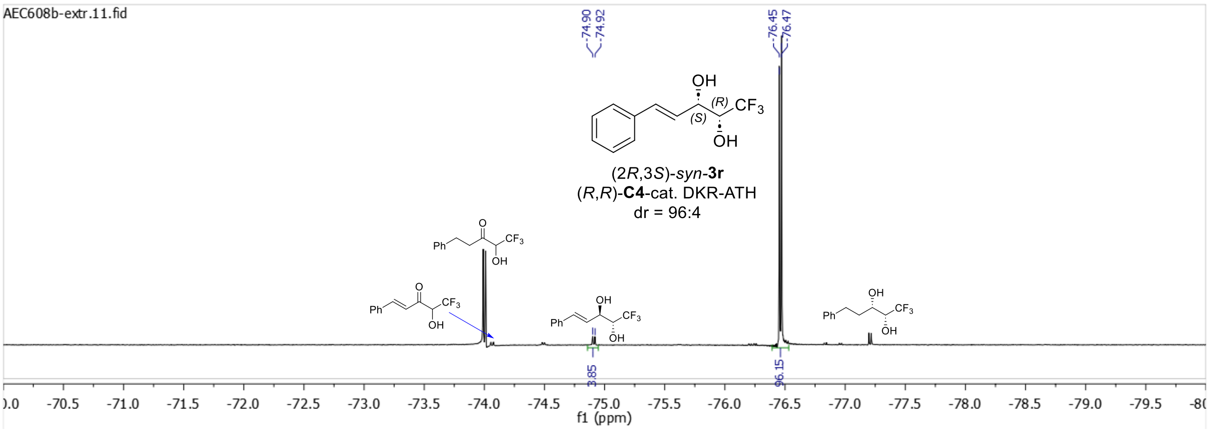

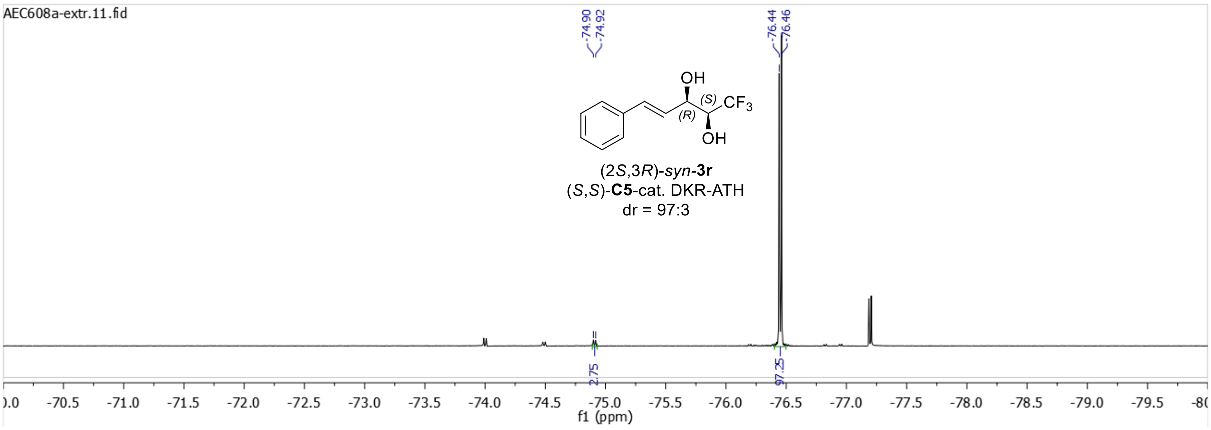

6.2 *Determination of enantiomeric ratios by chiral GC and HPLC chromatography*

Enantiomeric excess of *syn*-3a (Table 1 and Table S1) was determined by GC analysis using chiral stationary phase. (CP-ChiraSil-DEX CB column (25 m x 0.25 cm), isothermal elution with helium at 150 °C

3,3,3-trifluoro-1-phenylpropane-1,2-diol (3a)
Prepared via $NaBH_4$ reduction, *trans*-**3a**/*cis*-**3a** = 97:3

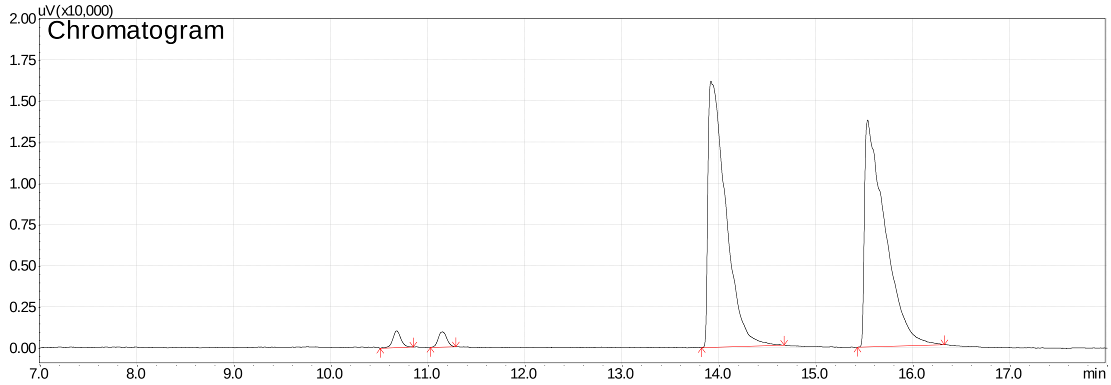

Peak	t_r (min)	Area	Height	Area %
(1*S*,2*R*)-**3a**	10.673	6081.5	1020.8	1.4432
(1*R*,2*S*)-**3a**	11.144	5718.9	920.4	1.3572
anti-**3a**	13.914	206795.8	16171.0	49.0760
ent-anti-**3a**	15.529	202782.5	13779.7	48.1236

3,3,3-trifluoro-1-phenylpropane-1,2-diol (3a)
Prepared via DKR-ATH using (*R*,*R*)-**C1**, Table S1, entry 1. ee *syn* = 91.3%

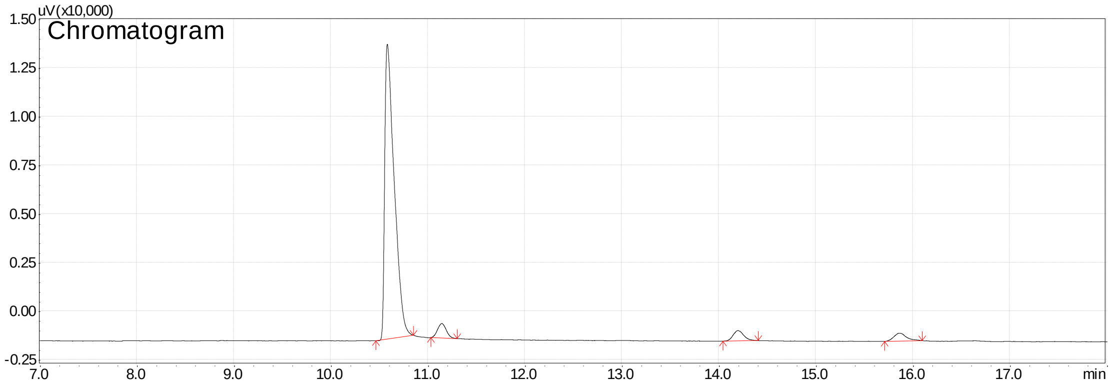

Peak	t_r (min)	Area	Height	Area %
(1*S*,2*R*)-**3a**	10.577	103343.2	15141.7	89.2774
(1*R*,2*S*)-**3a**	11.138	4690.1	746.7	4.0518
anti-**3a**	14.193	4048.3	528.9	3.4973
ent-anti-**3a**	15.857	3673.6	406.3	3.1736

3,3,3-trifluoro-1-phenylpropane-1,2-diol (3a)
Prepared via DKR-ATH using (*S*,*S*)-**C2**, Table S1, entry 2. ee *syn* = 97.7%

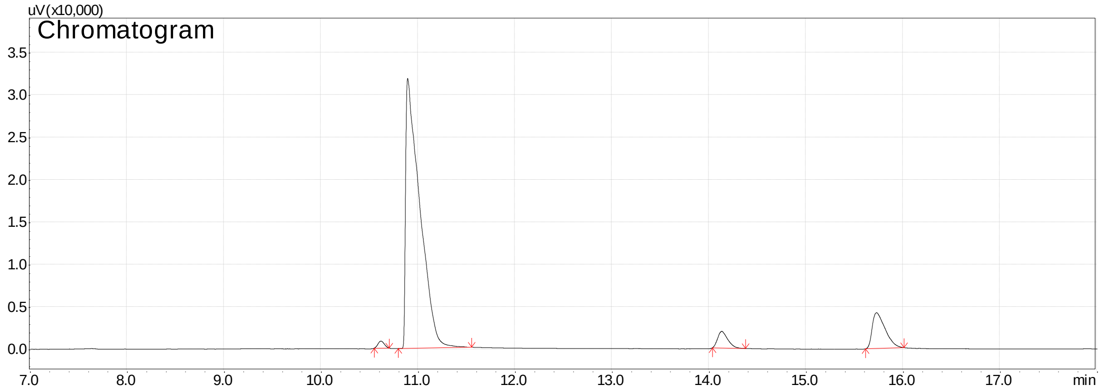

Peak	t_r (min)	Area	Height	Area %
(1*S*,2*R*)-**3a**	10.615	3799.2	834.4	1.0006
(1*R*,2*S*)-**3a**	10.891	322619.9	31806.9	84.9697
anti-**3a**	14.129	14133.6	1965.6	3.7224
ent-anti-**3a**	15.724	39135.3	4214.2	10.3072

3,3,3-trifluoro-1-phenylpropane-1,2-diol (3a)
Prepared via DKR-ATH using (*S*,*S*)-**C3**, Table S1, entry 3. ee *syn* = 99.1%

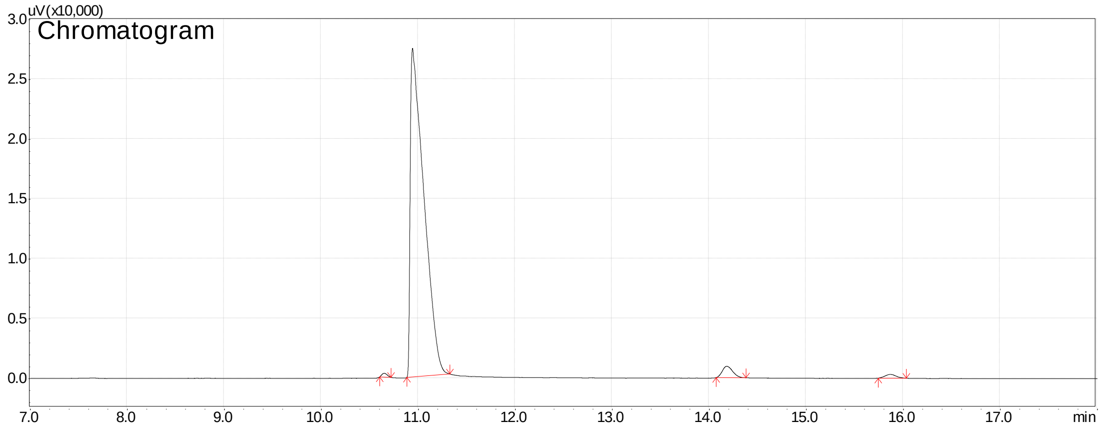

Peak	t_R (min)	Area	Height	Area %
(1*S*,2*R*)-**3a**	10.648	1189.2	311.1	0.4245
(1*R*,2*S*)-**3a**	10.940	269242.6	27427.7	96.1062
anti-**3a**	14.180	7142.8	948.5	2.5496
ent-anti-**3a**	15.866	2576.4	317.4	0.9197

3,3,3-trifluoro-1-phenylpropane-1,2-diol (3a)
Prepared via DKR-ATH using (*S*,*S*)-**C4**, Table S1, entry 4. ee *syn* = 99.5%

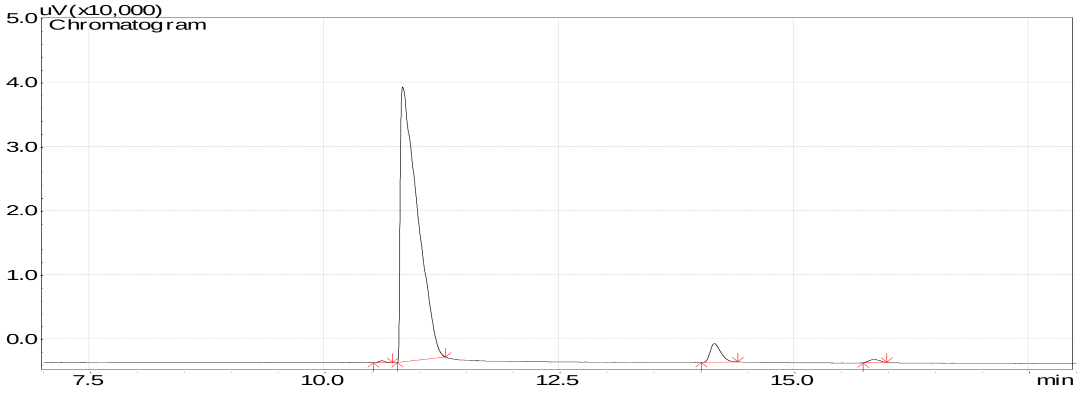

Peak	t_r (min)	Area	Height	Area %
(1*S*,2*R*)-**3a**	10.603	1345.3	299.2	0.2349
(1*R*,2*S*)-**3a**	10.825	544421.2	42863.9	95.0616
anti-**3a**	14.148	23053.8	2899.1	4.0254
ent-anti-**3a**	15.850	3883.4	482.5	0.6781

3,3,3-trifluoro-1-phenylpropane-1,2-diol (3a)
Prepared via DKR-ATH using (*S*,*S*)-**C5**, Table S1, entry 5. ee *syn* = 99.8%

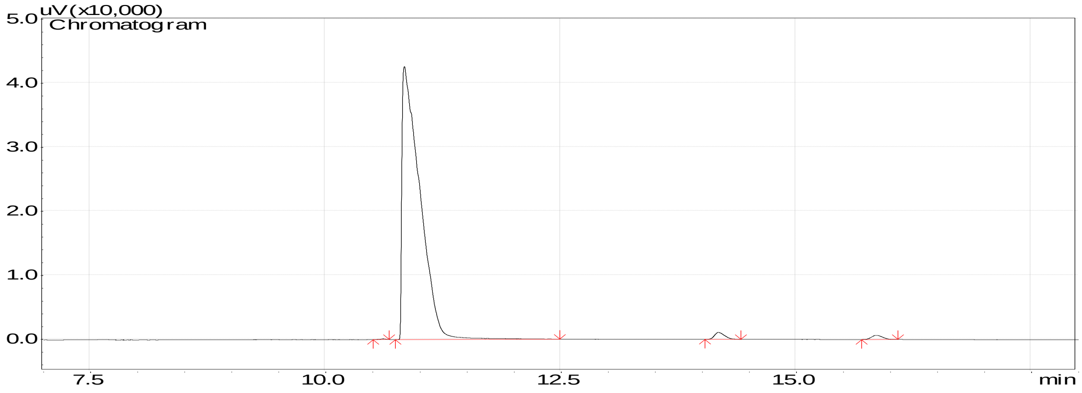

Peak	t_r (min)	Area	Height	Area %
(1*S*,2*R*)-**3a**	10.608	589.5	141.8	0.1016
(1*R*,2*S*)-**3a**	10.834	565256.6	42624.9	97.4051
anti-**3a**	14.171	8426.1	1105.1	1.4520
ent-anti-**3a**	15.850	6042.7	683.4	1.0413

3,3,3-trifluoro-1-phenylpropane-1,2-diol (3a)
Prepared via DKR-ATH using (*R*,*R*)-**C6**, Table S1, entry 6. ee *syn* = 97.3%

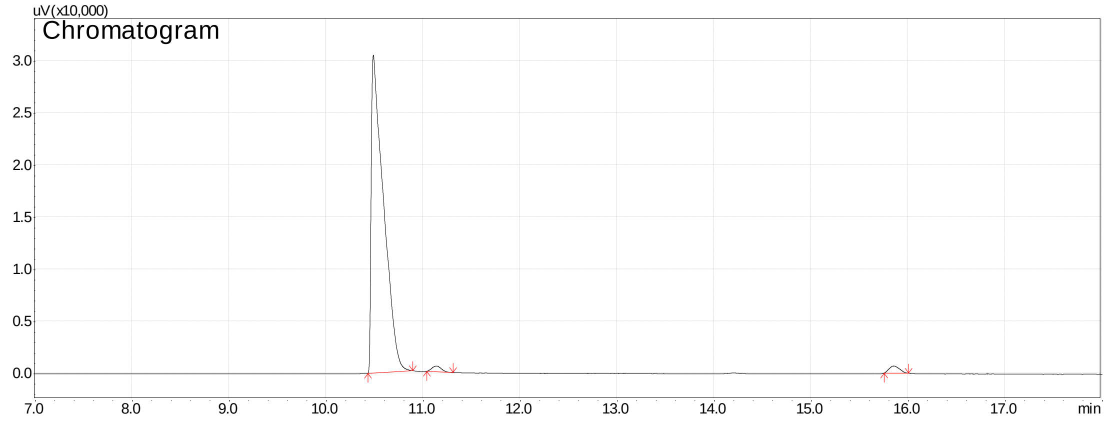

Peak	t_R (min)	Area	Height	Area %
(1*S*,2*R*)-**3a**	10.485	268270.2	30455.2	96.8386
(1*R*,2*S*)-**3a**	11.134	3637.6	543.0	1.3131
ent-anti-**3a**	15.850	5120.4	684.2	1.8483

3,3,3-trifluoro-1-phenylpropane-1,2-diol (3a)
Prepared via DKR-ATH using (*S*,*S*)-**C7**, Table S1, entry 7. ee *syn* = 98.5%

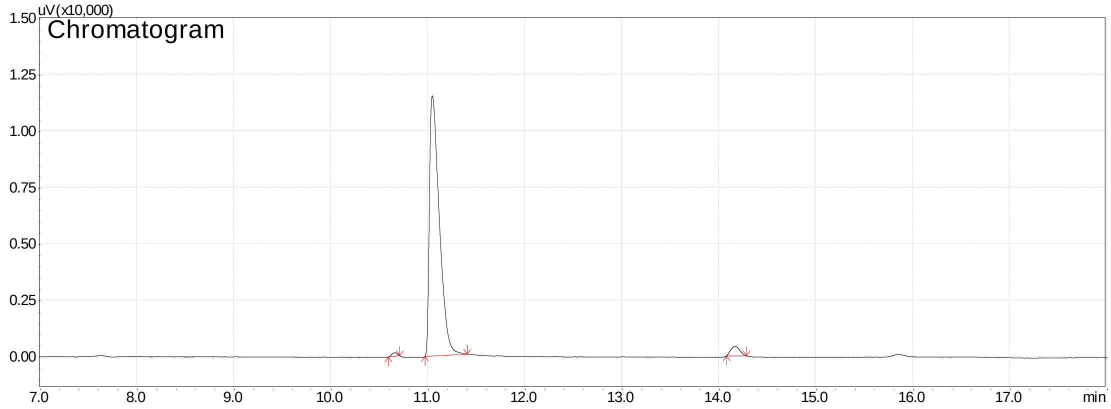

Peak	t_r (min)	Area	Height	Area %
(1*S*,2*R*)-**3a**	10.655	637.5	160.6	0.7469
(1*R*,2*S*)-**3a**	11.041	81996.8	11534.6	96.0686
anti-**3a**	14.162	2718.1	424.3	3.1846

3,3,3-trifluoro-1-phenylpropane-1,2-diol (3a)

Prepared via DKR-ATH using ((3*R*,1'*S*)-**C8**. Table S1, entry 8. ee *syn* = 74.9%

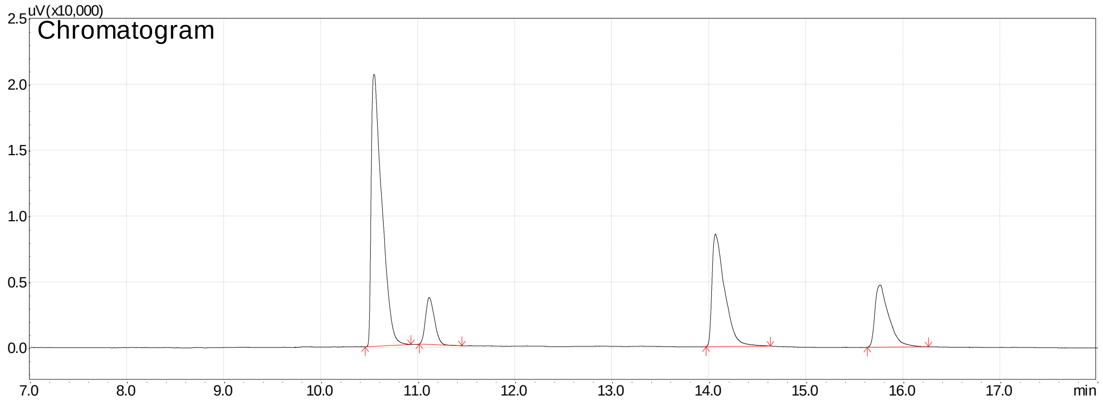

Peak	t_r (min)	Area	Height	Area %
(1*S*,2*R*)-**3a**	10.541	157938.8	20631.1	51.8089
(1*R*,2*S*)-**3a**	11.110	22684.1	3557.0	7.4411
anti-**3a**	14.059	77512.7	8536.8	25.4266
ent-anti-**3a**	15.758	46713.2	4708.3	15.3234

3,3,3-trifluoro-1-phenylpropane-1,2-diol (3a)

Prepared via DKR-ATH using (*S,S*)-**C4** in HCOOH/Et_3N 5:2. Table S1, entry 10. ee *syn* > 99.9%

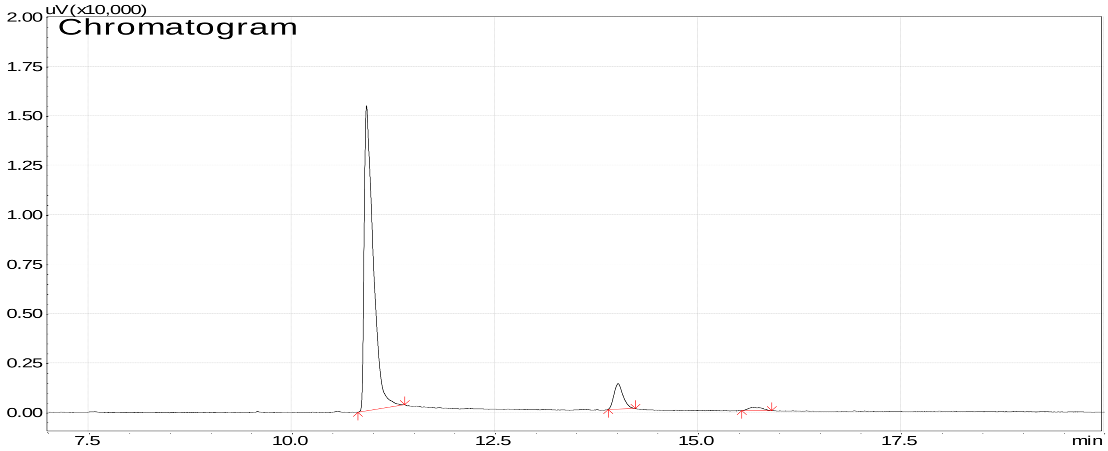

Peak	t_r (min)	Area	Height	Area %
1	10.913	119068.5	15428.5	90.8391
2	14.010	10077.1	1294.2	7.6879
3	15.668	1930.7	155.3	1.4729

3,3,3-trifluoro-1-phenylpropane-1,2-diol (3a)
Prepared via DKR-ATH using (*S,S*)-**C4**, S/C = 2000. Table S2, entry 11. ee *syn* = 99.5%

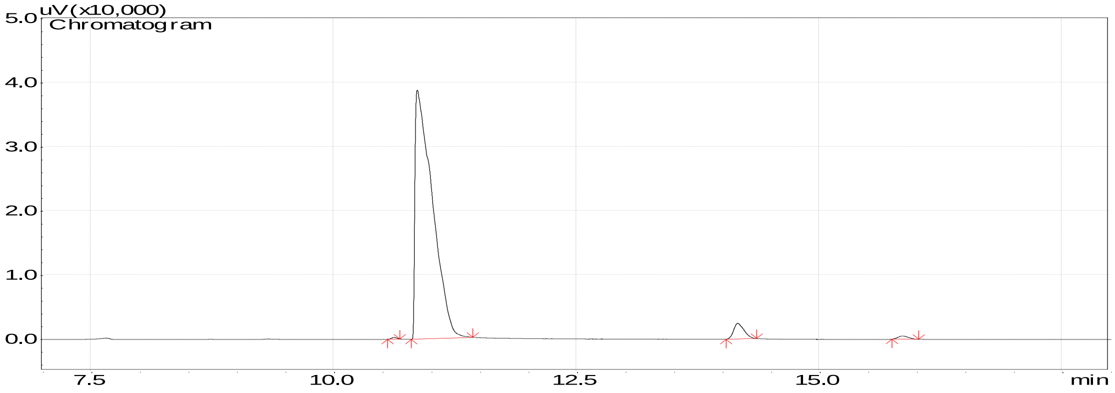

Peak	t_r (min)	Area	Height	Area %
(1*S*,2*R*)-**3a**	10.614	1086.7	274.6	0.2151
(1*R*,2*S*)-**3a**	10.851	482051.5	38817.8	95.4323
(±)-anti-**3a**	14.151	18175.1	2434.9	3.5982
ent-anti-**3a**	15.851	3810.9	500.6	0.7544

3,3,3-trifluoro-1-phenylpropane-1,2-diol (3a)
Prepared via DKR-ATH using (*S,S*)-**C5**, S/C = 2000. Table S2, entry 12. ee *syn* = 99.8%

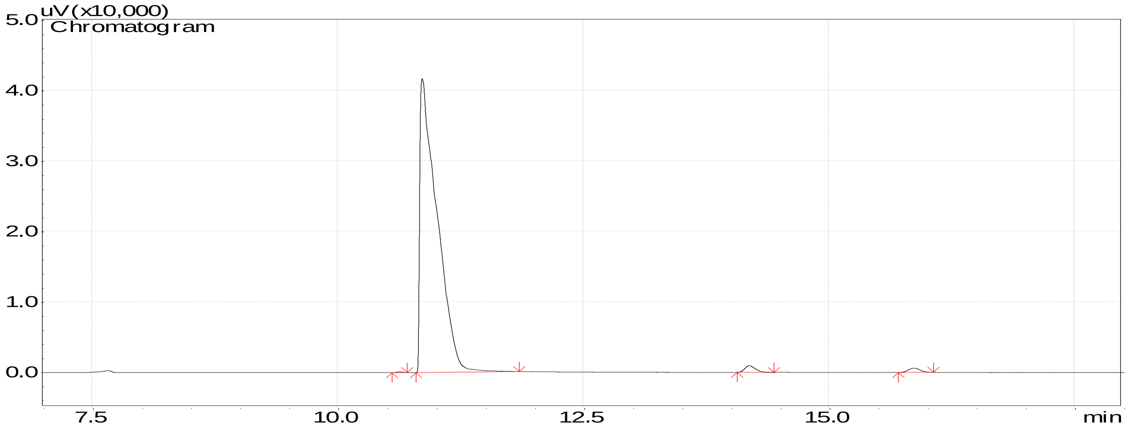

Peak	t_r (min)	Area	Height	Area %
(1*S*,2*R*)-**3a**	10.617	517.6	120.6	0.0990
(1*R*,2*S*)-**3a**	10.848	509734.4	41681.1	97.5176
(±)-anti-**3a**	14.178	7254.7	964.3	1.3879
ent-anti-**3a**	15.854	5203.5	620.4	0.9955

3,3,3-trifluoro-1-phenylpropane-1,2-diol (3a)

Prepared via DKR-ATH using (*S,S*)-**C5** at 40 °C. Table S2, entry 13. ee *syn* > 99.9%

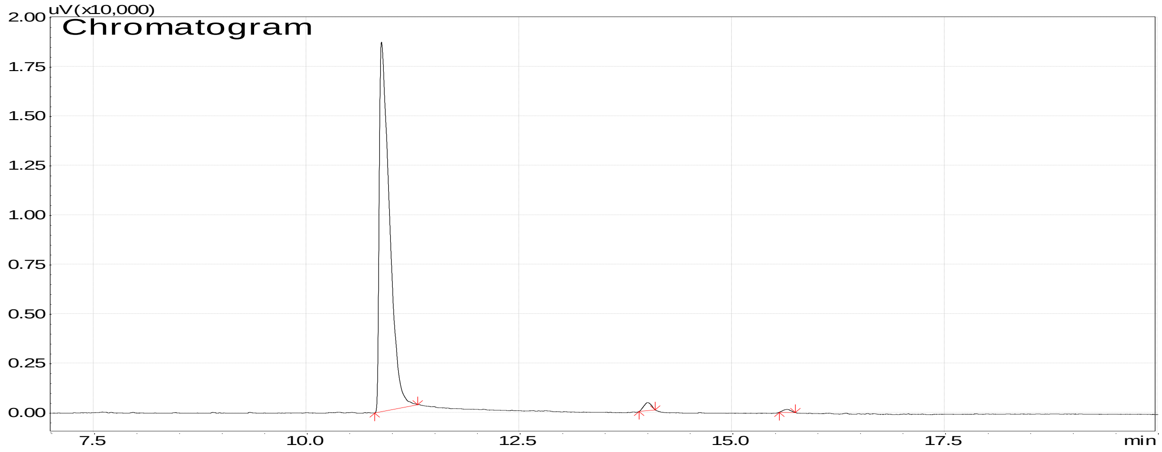

Peak	t_r (min)	Area	Height	Area %
(1*R*,2*S*)-**3a**	10.872	156401.6	18687.6	97.7867
(±)-anti-**3a**	13.997	2544.9	406.7	1.5911
ent-anti-**3a**	15.634	995.1	154.2	0.6222

1-(4-bromophenyl)-3,3,3-trifluoropropane-1,2-diol (3b)

1-(4-bromophenyl)-3,3,3-trifluoropropane-1,2-diol ($NaBH_4$ reduction)
Chiralpak IB-3 (25 cm) hexane/2-PrOH 98:2 to 90:10 in 20 min (Volume of injection = 5 µL, 25 °C, 1 mL/min, sample dissolved in 2-PrOH).

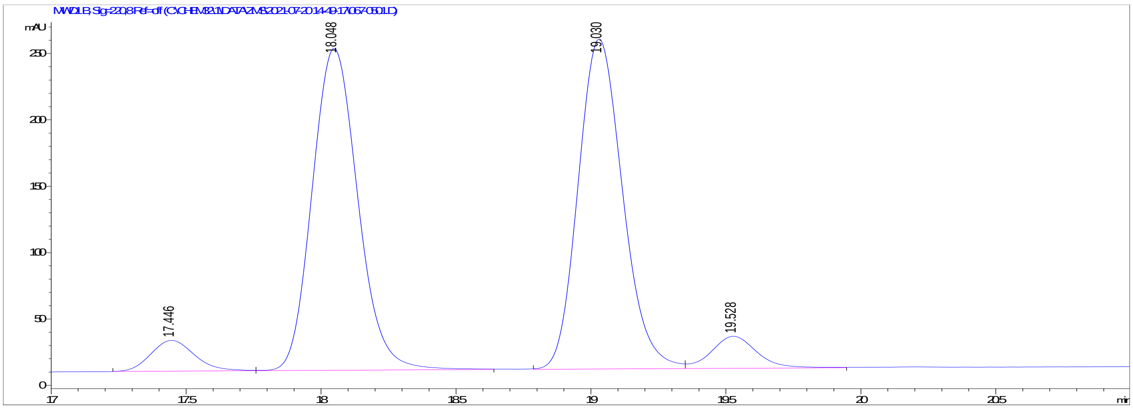

Peak	t_r (min)	Area	Height	Width	Area%	Symmetry
(1*R*,2*S*)-**3b**	17.446	276.7	23.9	0.1931	4.582	0.852
anti-**3b**	18.048	2771.5	241.1	0.1916	45.895	0.922
ent-anti-**3b**	19.03	2765.4	246.9	0.1866	45.794	0.888
(1*S*,2*R*)- **3b**	19.527	225.2	21.9	0.171	3.729	0.915

(1*R*,2*S*)-1-(4-bromophenyl)-3,3,3-trifluoropropane-1,2-diol ((*S*,*S*)-**C4**-cat., ee (*syn*) >99%)

Chiralpak IB-3 (25 cm) hexane/2-PrOH 98:2 to 90:10 in 20 min (Volume of injection = 5 µL, 25 °C, 1 mL/min, sample dissolved in 2-PrOH).

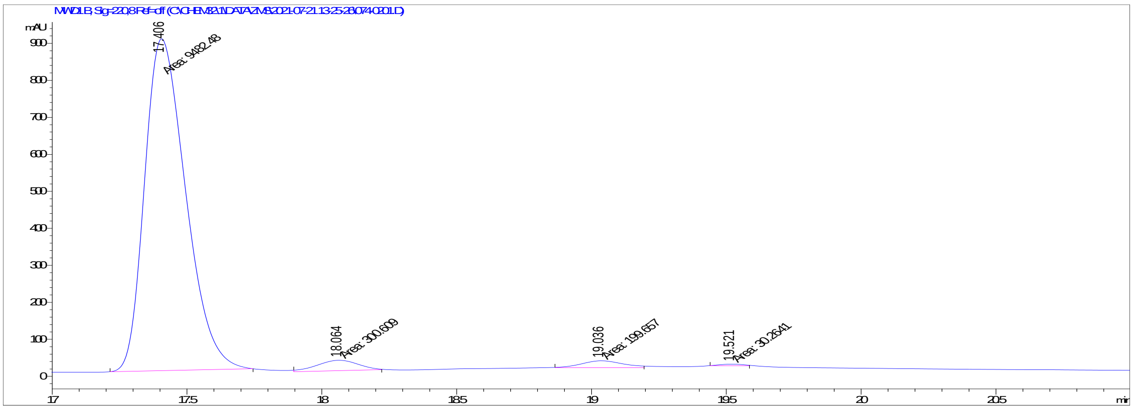

Peak	t_r (min)	Area	Height	Width	Area%	Symmetry
(1*R*,2*S*)-**3b**	17.406	9482.5	898.4	0.1759	94.702	0.703
anti-**3b**	18.064	300.6	28.4	0.1763	3.002	1.117
ent-anti-**3b**	19.036	199.7	18.6	0.1786	1.994	0.923
(1*S*,2*R*)-**3b**	19.521	30.3	4.8	0.1047	0.302	1.062

1-(4-bromophenyl)-3,3,3-trifluoropropane-1,2-diol ($NaBH_4$ reduction)
Chiralpak IB-3 (25 cm) hexane/2-PrOH 98:2 to 90:10 in 20 min (Volume of injection = 5 µL, 25 °C, 1 mL/min, sample dissolved in 2-PrOH).

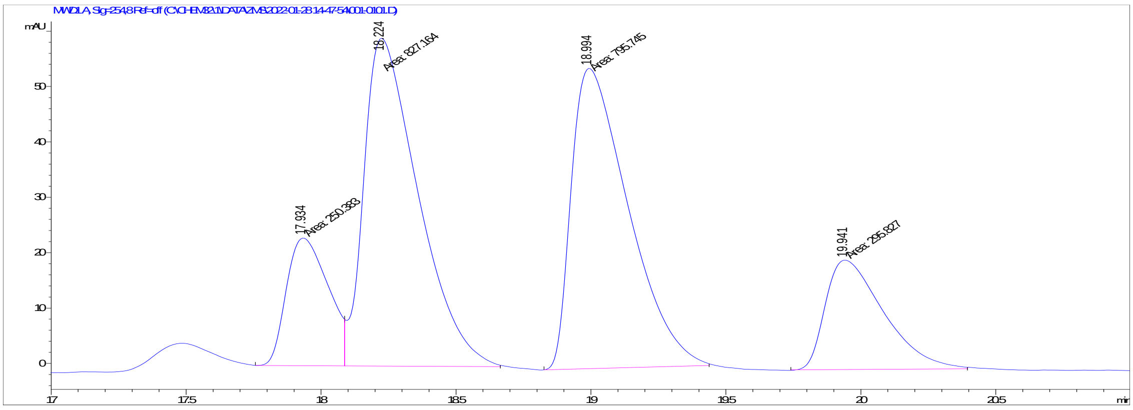

Peak	t_r (min)	Area	Height	Width	Area%	Symmetry
(1*R*,2*S*)-**3b**	17.934	250.4	23.1	0.1806	11.543	0.637
anti-**3b**	18.224	827.2	59.3	0.2326	38.134	0.48
ent-anti-**3b**	18.994	795.7	54.3	0.2442	36.685	0.437
(1*S*,2*R*)-**3b**	19.941	295.8	1.98E+01	0.2492	13.638	0.51

(1*R*,2*S*)-1-(4-bromophenyl)-3,3,3-trifluoropropane-1,2-diol ((*S*,*S*)-**C5**-cat., ee (*syn*) >99%)

Chiralpak IB-3 (25 cm) hexane/2-PrOH 98:2 to 90:10 in 20 min (Volume of injection = 5 µL, 25 °C, 1 mL/min, sample dissolved in 2-PrOH).

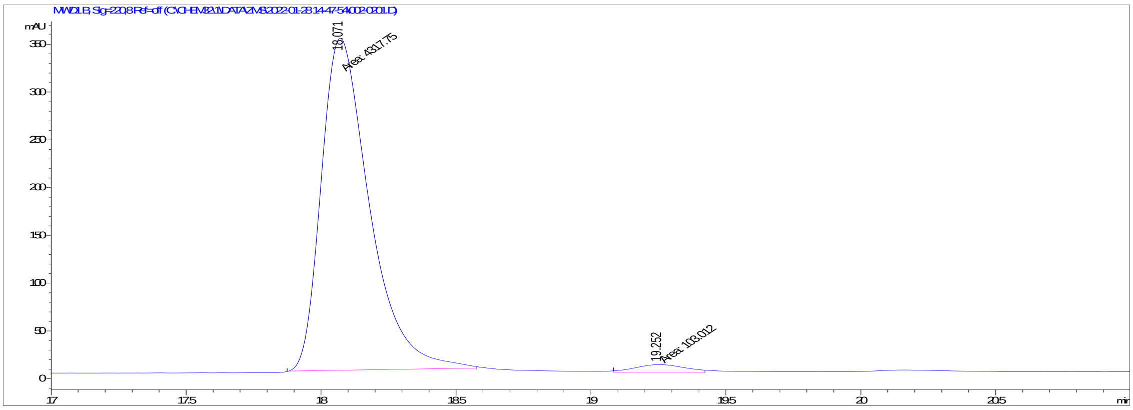

Peak	t_r (min)	Area	Height	Width	Area%	Symmetry
(1*R*,2*S*)-**3b**	18.071	4317.8	3.48E+02	0.2065	97.67	0.608
anti-**3b**	19.252	103	8.00E+00	0.2141	2.33	0.864

1-(4-chlorophenyl)-3,3,3-trifluoropropane-1,2-diol (3c)

1-(4-chlorophenyl)-3,3,3-trifluoropropane-1,2-diol ($NaBH_4$ reduction)
Chiralpak IB-3 (25 cm) hexane/2-PrOH 98:2 to 90:10 in 20 min (Volume of injection = 5 µL, 25 °C, 1 mL/min, sample dissolved in 2-PrOH).

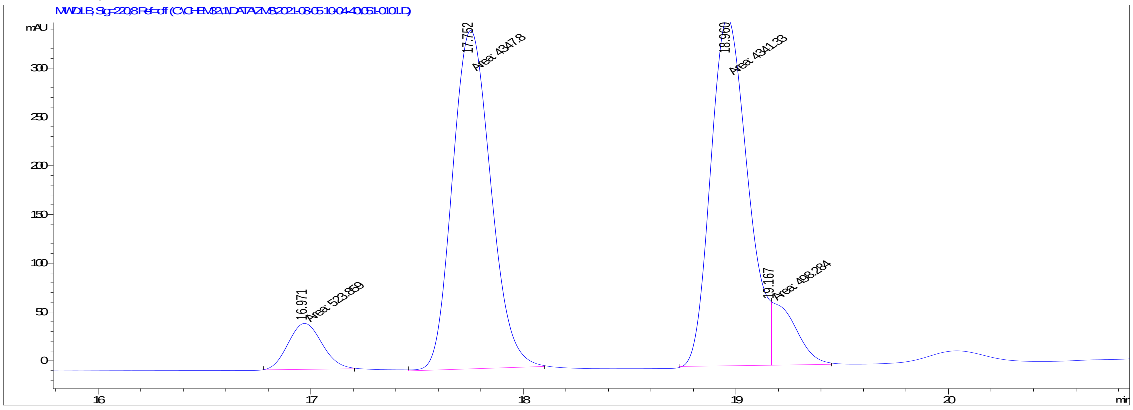

Peak	t_r (min)	Area	Height	Width	Area%	Symmetry
(1*R*,2*S*)-**3c**	16.971	523.9	47.3	0.1848	5.394	0.893
anti-**3c**	17.752	4347.8	347.7	0.2084	44.771	0.906
ent-anti-**3c**	18.96	4341.3	357.2	0.2025	44.704	0.814
(1*S*,2*R*)-**3c**	19.167	498.3	64.8	0.1281	5.131	0

(1*R*,2*S*)-1-(4-chlorophenyl)-3,3,3-trifluoropropane-1,2-diol ((*S*,*S*)-**C4**-cat., ee (*syn*) = 97%)
Chiralpak IB-3 (25 cm) hexane/2-PrOH 98:2 to 90:10 in 20 min (Volume of injection = 5 µL, 25 °C, 1 mL/min, sample dissolved in 2-PrOH).

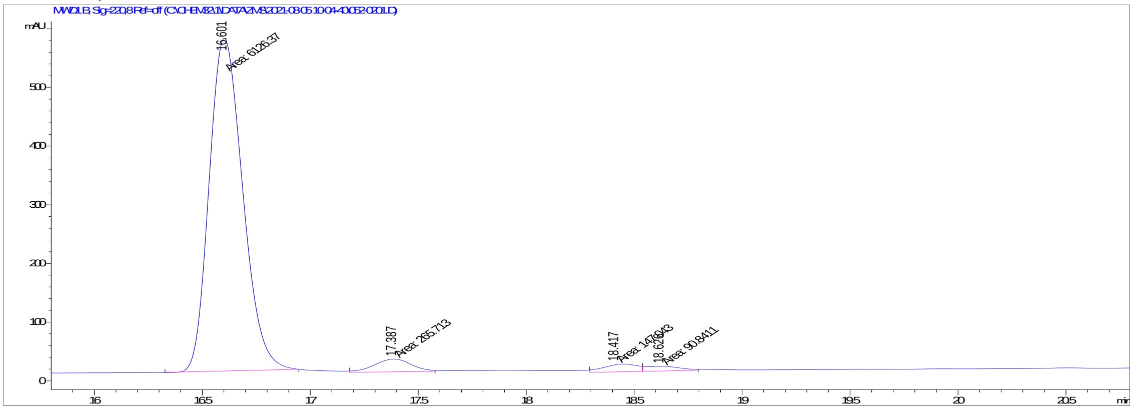

Peak	t_r (min)	Area	Height	Width	Area%	Symmetry
(1*R*,2*S*)-**3c**	16.601	6126.4	568.8	0.1795	92.404	0.759
anti-**3c**	17.387	265.7	22.1	0.2003	4.008	1.075
ent-anti-**3c**	18.417	147	13.4	0.1827	2.218	1.273
(1*S*,2*R*)-**3c**	18.626	90.8	8.30E+00	0.1599	1.37	0.786

1-(4-chlorophenyl)-3,3,3-trifluoropropane-1,2-diol ($NaBH_4$ reduction)

Chiralpak IB-3 (25 cm) hexane/2-PrOH 98:2 to 90:10 in 20 min (Volume of injection = 5 µL, 25 °C, 1 mL/min, sample dissolved in 2-PrOH).

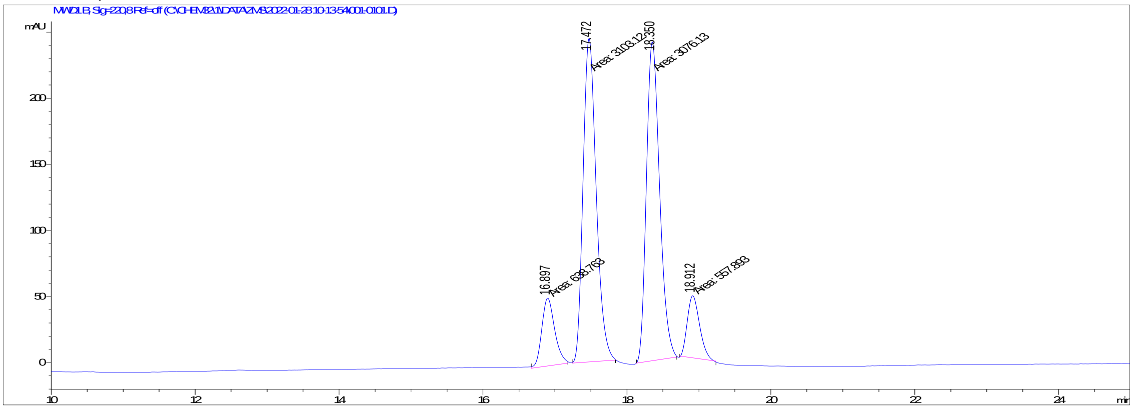

Peak	t_r (min)	Area	Height	Width	Area%	Symmetry
(1*R*,2*S*)-**3c**	16.897	638.8	51.4	0.207	8.66	0.83
anti-**3c**	17.472	3103.1	245.2	0.211	42.071	0.798
ent-anti-**3c**	18.35	3076.1	241.6	0.2122	41.705	0.735
(1*S*,2*R*)-**3c**	18.912	557.9	47	0.1978	7.564	0.811

(1*R*,2*S*)-1-(4-chlorophenyl)-3,3,3-trifluoropropane-1,2-diol ((*S*,*S*)-**C5**-cat., ee (*syn*) >99%)

Chiralpak IB-3 (25 cm) hexane/2-PrOH 98:2 to 90:10 in 20 min (Volume of injection = 5 µL, 25 °C, 1 mL/min, sample dissolved in 2-PrOH).

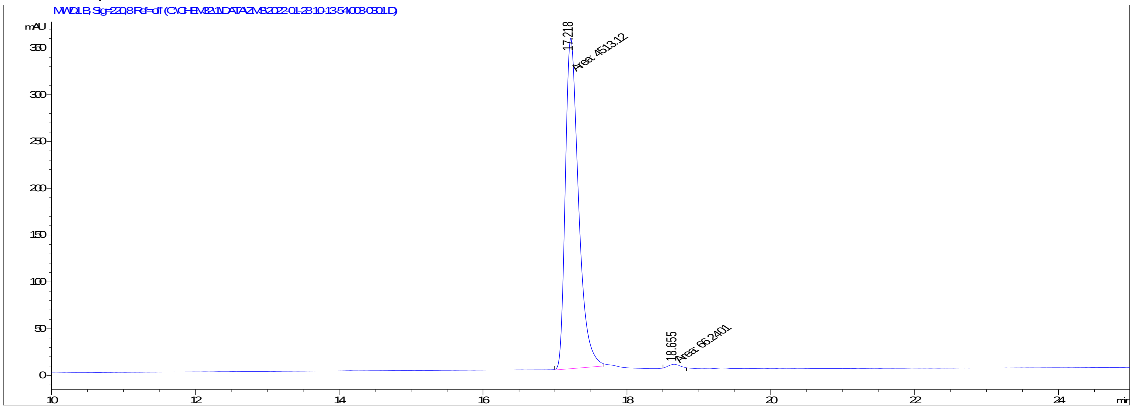

Peak	t_r (min)	Area	Height	Width	Area%	Symmetry
(1*R*,2*S*)-**3c**	17.218	4513.1	353.4	0.2128	98.554	0.663
ent-anti-**3c**	18.655	66.2	5.1	0.2145	1.446	0.856

3,3,3-trifluoro-1-(4-fluorophenyl)propane-1,2-diol (3d)

3,3,3-trifluoro-1-(4-fluorophenyl)propane-1,2-diol ($NaBH_4$ reduction)
Chiralpak IB-3 (25 cm) hexane/2-PrOH 98:2 to 90:10 in 20 min (Volume of injection = 5 µL, 25 °C, 1 mL/min, sample dissolved in 2-PrOH).

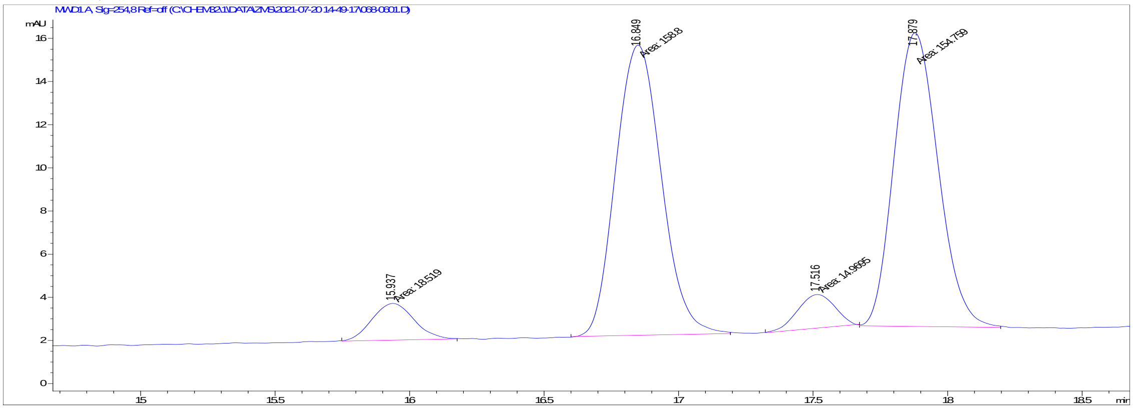

Peak	t_r (min)	Area	Height	Width	Area%	Symmetry
(1*R*,2*S*)-**3d**	15.937	18.5	1.7	0.1807	5.336	0.945
anti-**3d**	16.849	158.8	13.5	0.1964	45.757	0.863
(1*S*,2*R*)-**3d**	17.516	15	1.6	0.1592	4.313	1.054
ent-anti-**3d**	17.879	154.8	13.6	0.1892	44.593	0.845

(1*R*,2*S*)-3,3,3-trifluoro-1-(4-fluorophenyl)propane-1,2-diol ((*S*,*S*)-**C4**-cat., ee (*syn*) = 98%)
Chiralpak IB-3 (25 cm) hexane/2-PrOH 98:2 to 90:10 in 20 min (Volume of injection = 5 µL, 25 °C, 1 mL/min, sample dissolved in 2-PrOH).

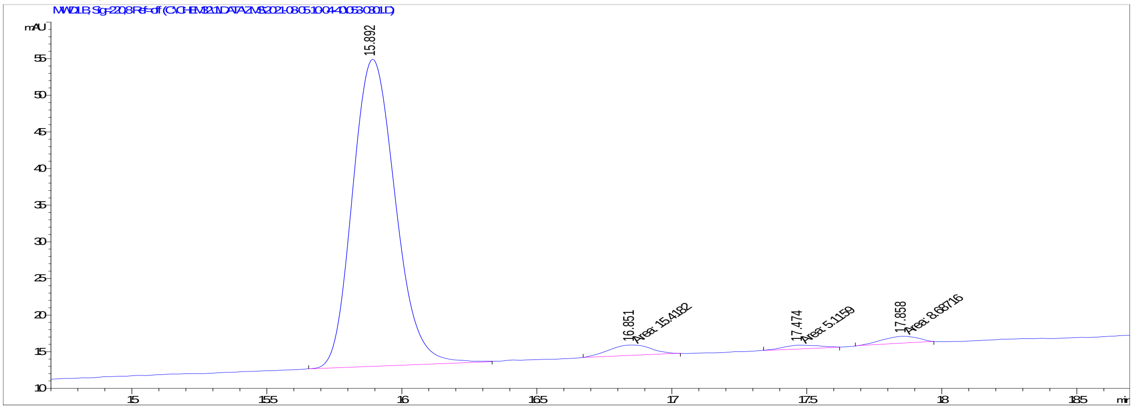

Peak	t_r (min)	Area	Height	Width	Area%	Symmetry
(1*R*,2*S*)-**3d**	15.892	463.2	41.9	0.177	94.065	0.841
anti-**3d**	16.851	15.4	1.4	0.1795	3.131	1.214
(1*S*,2*R*)-**3d**	17.474	5.1	5.50E-01	0.1552	1.039	0.977
ent-anti-**3d**	17.858	8.7	9.50E-01	0.153	1.764	1.535

(1*R*,2*S*)-3,3,3-trifluoro-1-(4-fluorophenyl)propane-1,2-diol ((*S*,*S*)-**C5**-cat., ee (*syn*) >99%)
Chiralpak IB-3 (25 cm) hexane/2-PrOH 98:2 to 90:10 in 20 min (Volume of injection = 5 μL, 25 °C, 1 mL/min, sample dissolved in 2-PrOH).

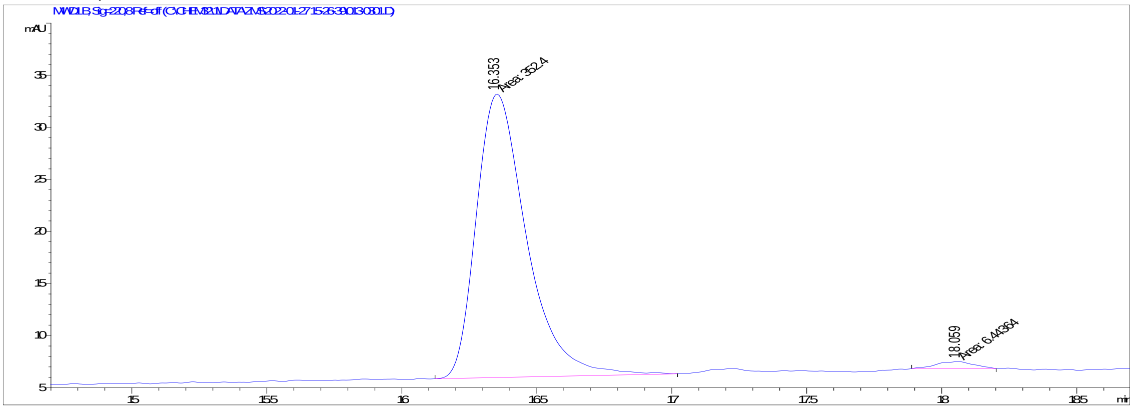

Peak	t_r (min)	Area	Height	Width	Area%	Symmetry
(1*R*,2*S*)-**3d**	16.353	352.4	27.2	0.2156	98.204	0.681
ent-anti-**3d**	18.059	6.4	6.80E-01	0.1582	1.796	1.177

3,3,3-trifluoro-1-(4-nitrophenyl)propane-1,2-diol (3e)

A refference mixture of the DKR-ATH products from runs with (*S,S*)-**C4** and (*R,R*)-**C4**.
Chiralpak IA-3 (25 cm) hexane/2-PrOH 95:5 (Volume of injection = 10 µL, 25 °C, 1 mL/min, λ = 254 nm)

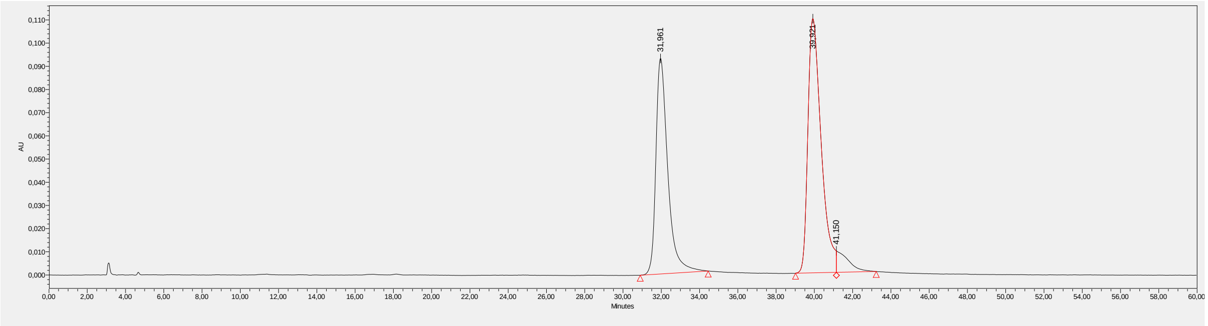

	Name	Retention Time	Area	% Area	Height
1	(1*R*,2*S*)-**3e** + *anti*-**3e**	31,961	4020997	41,38	93001
2	(1*S*,2*R*)-**3e**	39,921	5291872	54,46	109659
3	*ent-anti*-**3e**	41,150	403843	4,16	9275

(1*R*,2*S*)-3,3,3-trifluoro-1-(4-nitrophenyl)propane-1,2-diol ((*S,S*)-**C4**-cat., ee (*syn*) 99.4%).
Chiralpak IA-3 (25 cm) hexane/2-PrOH 95:5 (Volume of injection = 10 µL, 25 °C, 1 mL/min, λ = 254 nm)

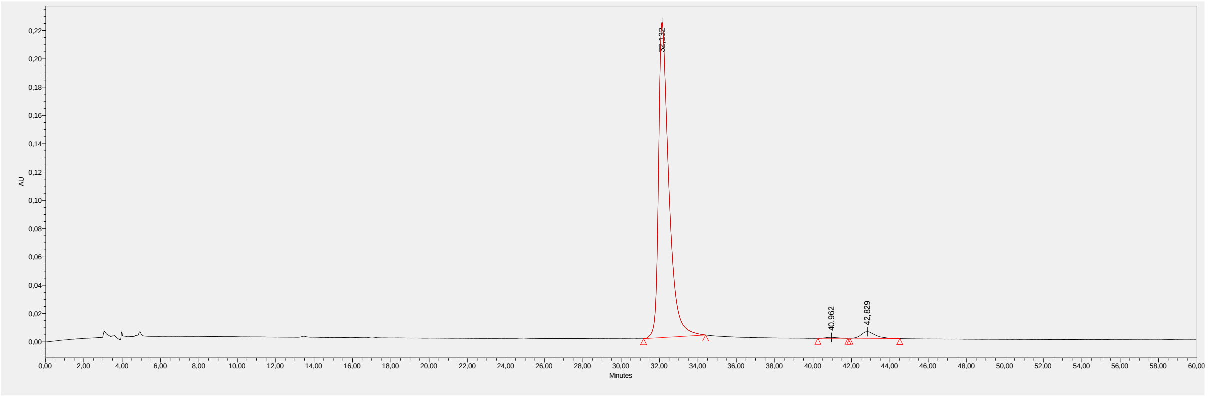

	Name	Retention Time	Area	% Area	Height
1	(1*R*,2*S*)-**3e** + *anti*-**3e**	32,132	8310130	96,88	222914
2	(1*S*,2*R*)-**3e**	40,962	28813	0,34	680
3	*ent-anti*-**3e**	42,829	238740	2,78	4698

(1*R*,2*S*)-3,3,3-trifluoro-1-(4-nitrophenyl)propane-1,2-diol ((*S*,*S*)-**C5**-cat., ee (*syn*) 98%).
Chiralpak IA-3 (25 cm) hexane/2-PrOH 95:5 (Volume of injection = 10 µL, 25 °C, 1 mL/min, λ = 254 nm)

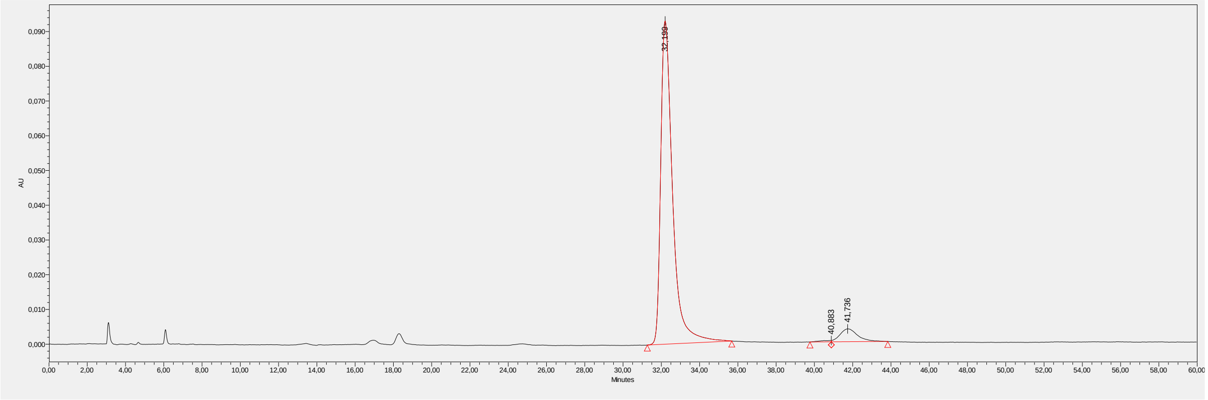

	Name	Retention Time	Area	% Area	Height
1	(1*R*,2*S*)-**3e** + *anti*-**3e**	32,199	3998931	94,00	93031
2	(1*S*,2*R*)-**3e**	40,883	16580	0,39	381
3	*ent-anti*-**3e**	41,736	238683	5,61	3704

3,3,3-trifluoro-1-(3-hydroxy-4-nitrophenyl)propane-1,2-diol (3f)

3,3,3-trifluoro-1-(3-hydroxy-4-nitrophenyl)propane-1,2-diol ($NaBH_4$ reduction)
Chiralpak IB-3 (25 cm) hexane/2-PrOH + 0.1% TFA 98:2 to 90:10 in 20 min (Volume of injection = 5 µL, 25 °C, 1 mL/min, sample dissolved in 2-PrOH).

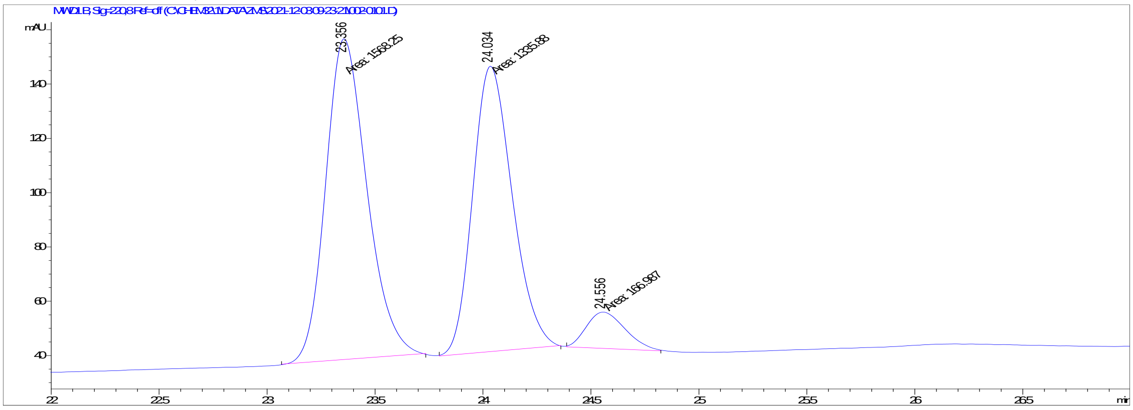

Peak	t_r (min)	Area	Height	Width	Area%	Symmetry
anti-**3f** + (1*R*,2*S*)-**3f**	23.356	1568.2	118.3	0.2209	51.064	0.813
ent-anti-**3f**	24.034	1335.9	105.3	0.2114	43.498	0.837
(1*S*,2*R*)-**3f**	24.556	167	13.4	0.2081	5.437	0.71

(1*R*,2*S*)-3,3,3-trifluoro-1-(3-hydroxy-4-nitrophenyl)propane-1,2-diol ((*S*,*S*)-**C4**-cat., ee (*syn*) >99%)

Chiralpak IB-3 (25 cm) hexane/2-PrOH + 0.1% TFA 98:2 to 90:10 in 20 min (Volume of injection = 5 µL, 25 °C, 1 mL/min, sample dissolved in 2-PrOH).

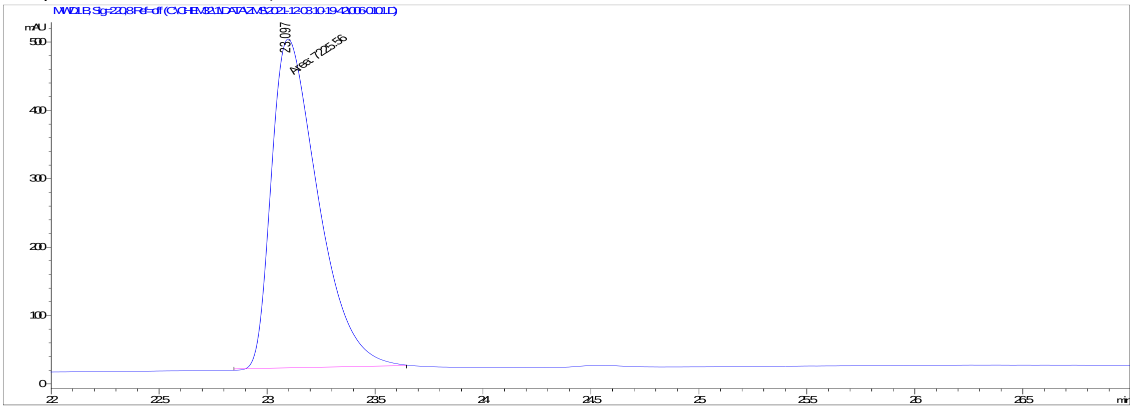

Peak	t_r (min)	Area	Height	Width	Area%	Symmetry
(1*R*,2*S*)-**3f** + *anti*-**3f**	23.097	7225.6	481.6	0.25	100	0.56

(1*R*,2*S*)-3,3,3-trifluoro-1-(3-hydroxy-4-nitrophenyl)propane-1,2-diol ((*S*,*S*)-**C5**-cat., ee (*syn*) >99%)

Chiralpak IB-3 (25 cm) hexane/2-PrOH + 0.1% TFA 98:2 to 90:10 in 20 min (Volume of injection = 5 µL, 25 °C, 1 mL/min, sample dissolved in 2-PrOH).

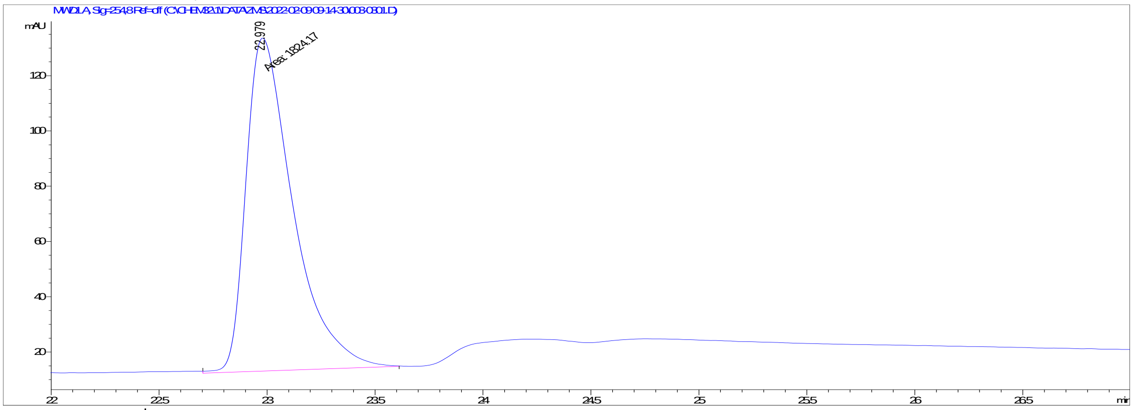

Peak	t_r (min)	Area	Height	Width	Area%	Symmetry
(1*R*,2*S*)-**3f**	22.979	1824.2	120.7	0.2519	100	0.55

3,3,3-trifluoro-1-(4-hydroxyphenyl)propane-1,2-diol (3g)

3,3,3-trifluoro-1-(4-hydroxyphenyl)propane-1,2-diol ($NaBH_4$ reduction)

Chiralpak IB-3 (25 cm) hexane/2-PrOH 90:10 (Volume of injection = 5 µL, 25 °C, 1 mL/min, sample dissolved in 2-PrOH).

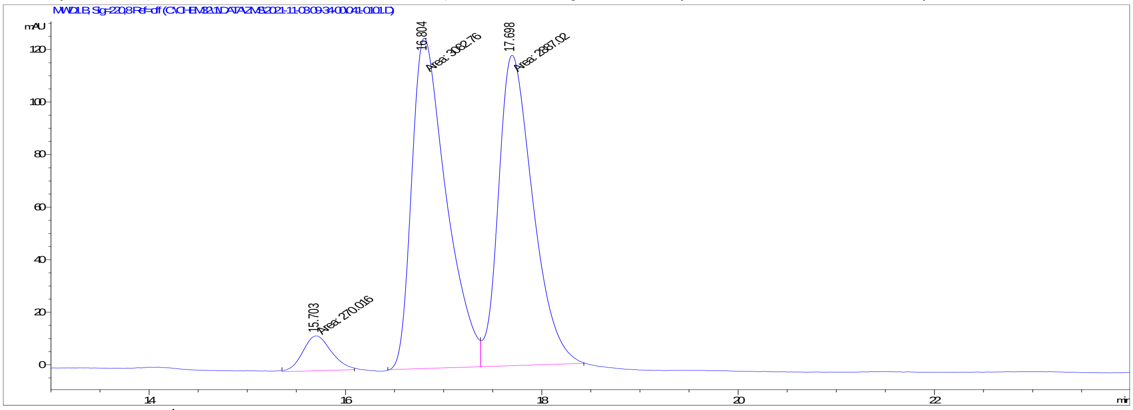

Peak	t_r (min)	Area	Height	Width	Area%	Symmetry
(1*R*,2*S*)-**3g**	15.703	270	13.3	0.3377	4.327	0.902
(1*S*,2*R*)-**3g** + *anti*-**3g**	16.804	3082.8	125.8	0.4083	49.405	0.565
ent-anti-**3g**	17.698	2887	118.3	0.4069	46.268	0.648

(1*R*,2*S*)-3,3,3-trifluoro-1-(4-hydroxyphenyl)propane-1,2-diol ((*S*,*S*)-**C4**-cat.). Sample after extraction.

Chiralpak IB-3 (25 cm) hexane/2-PrOH 90:10 (Volume of injection = 5 µL, 25 °C, 1 mL/min, sample dissolved in 2-PrOH).

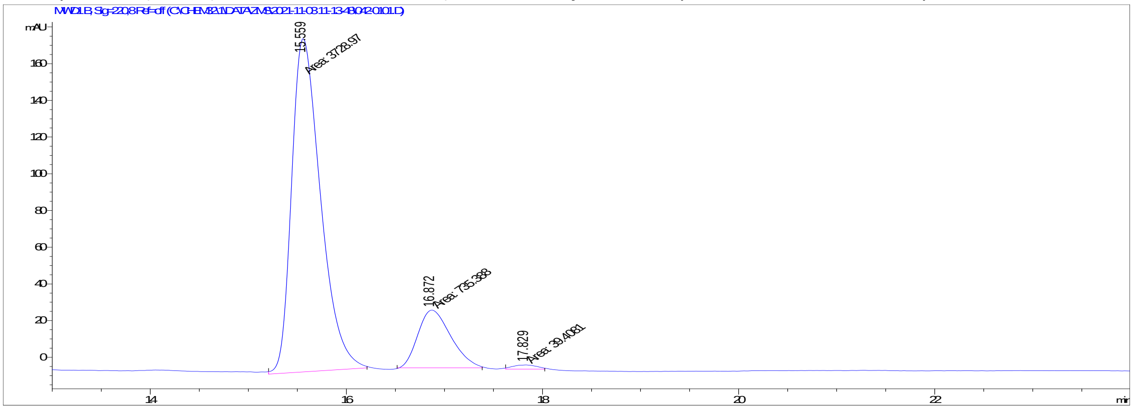

Peak	t_r (min)	Area	Height	Width	Area%	Symmetry
(1*R*,2*S*)-**3g**	15.559	3729	181.8	0.3419	82.797	0.634
(1*S*,2*R*)-**3g** + *anti*-**3g**	16.872	735.4	31.5	0.3894	16.328	0.707
ent-anti-**3g**	17.829	39.4	2.3	0.2828	0.875	1.125

(1*R*,2*S*)-3,3,3-trifluoro-1-(4-hydroxyphenyl)propane-1,2-diol ((*S*,*S*)-**C4**-cat., ee (*syn*) ≥99%). Diastereomerically pure sample after column chromatography on silica.
Chiralpak IB-3 (25 cm) hexane/2-PrOH 90:10 (Volume of injection = 5 µL, 25 °C, 1 mL/min, sample dissolved in 2-PrOH).

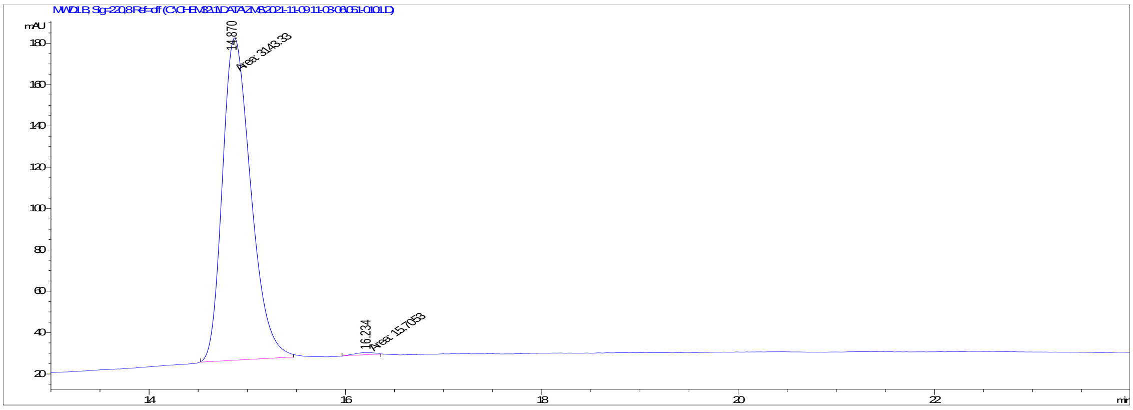

Peak	t_r (min)	Area	Height	Width	Area%	Symmetry
(1*R*,2*S*)- **3g**	14.87	3143.3	156.1	0.3357	99.503	0.732
(1*S*,2*R*)- **3g**	16.234	15.7	1.1	0.189	0.497	0.424

(1*R*,2*S*)-3,3,3-trifluoro-1-(4-hydroxyphenyl)propane-1,2-diol ((*S*,*S*)-**C5**-cat., ee (*syn*) >99%). Diastereomerically pure sample after column chromatography on silica.
Chiralpak IB-3 (25 cm) hexane/2-PrOH 90:10 (Volume of injection = 5 µL, 25 °C, 1 mL/min, sample dissolved in 2-PrOH).

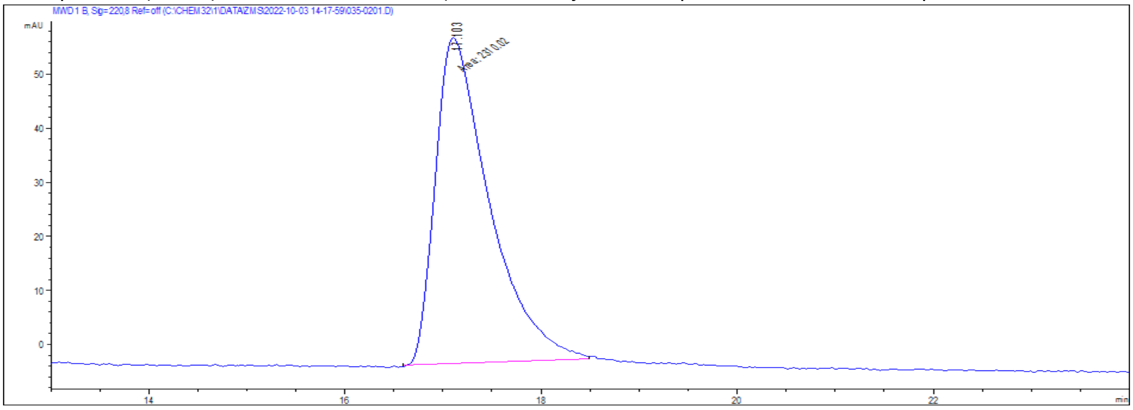

Peak	t_r (min)	Area	Height	Width	Area%	Symmetry
(1*R*,2*S*)-**3g**	17.103	2310	6.03E+01	0.639	100	0.467

3,3,3-trifluoro-1-(3-hydroxyphenyl)propane-1,2-diol (3h)

3,3,3-trifluoro-1-(3-hydroxyphenyl)propane-1,2-diol ($NaBH_4$ reduction)
Chiralpak IB-3 (25 cm) hexane/2-PrOH 90:10 in 30 min (Volume of injection = 5 µL, 25 °C, 1 mL/min, sample dissolved in 2-PrOH).

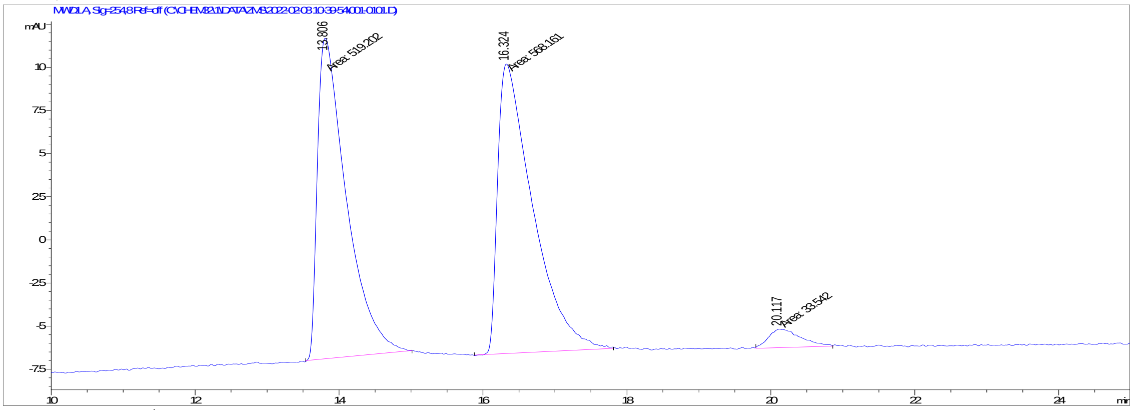

Peak	t_r (min)	Area	Height	Width	Area%	Symmetry
anti-**3h**	13.806	519.2	18.6	0.4654	46.32	0.349
ent-anti-**3h** + (1*R*,2*S*)-**3h**	16.324	568.2	16.8	0.5641	50.688	0.322
(1*S*,2*R*)-**3h**	20.117	33.5	1.1	0.5178	2.992	0.502

(1*R*,2*S*)-3,3,3-trifluoro-1-(3-hydroxyphenyl)propane-1,2-diol ((*S*,*S*)-**C4**-cat., ee (*syn*) >99%)
Chiralpak IB-3 (25 cm) hexane/2-PrOH 90:10 in 30 min (Volume of injection = 5 µL, 25 °C, 1 mL/min, sample dissolved in 2-PrOH).

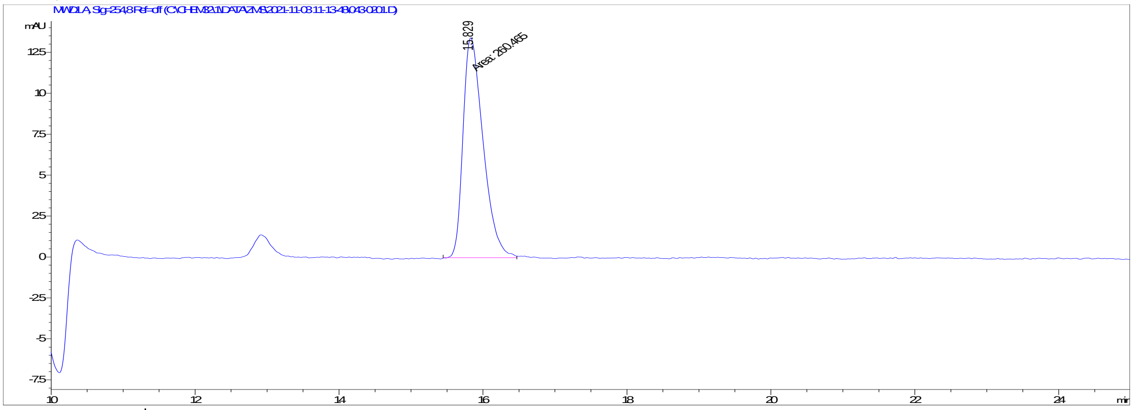

Peak	t_r (min)	Area	Height	Width	Area%	Symmetry
(1*R*,2*S*)-**3h**	15.829	260.5	13.4	0.3231	100	0.592

(1*R*,2*S*)-3,3,3-trifluoro-1-(3-hydroxyphenyl)propane-1,2-diol ((*S*,*S*)-**C5**-cat., ee (*syn*) >99%)

Chiralpak IB-3 (25 cm) hexane/2-PrOH 90:10 in 30 min (Volume of injection = 5 µL, 25 °C, 1 mL/min, sample dissolved in 2-PrOH).

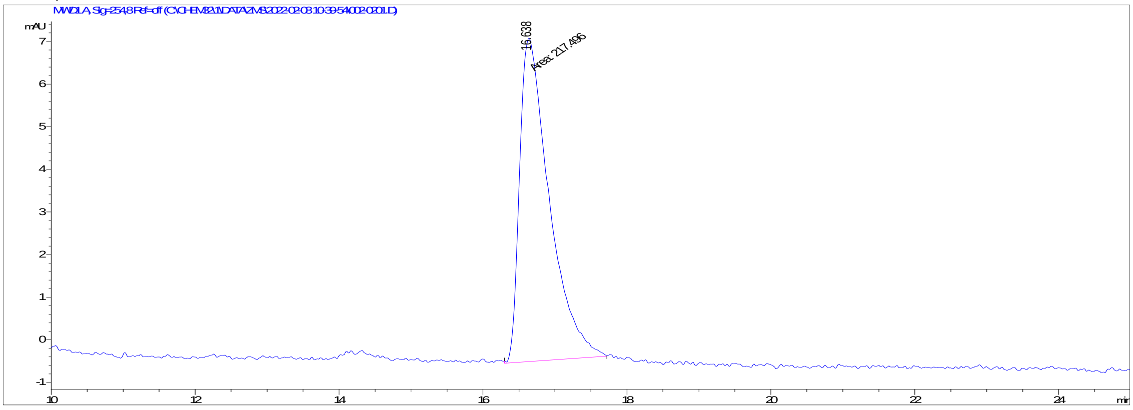

Peak	t_r (min)	Area	Height	Width	Area%	Symmetry
(1*R*,2*S*)-**3h**	16.638	217.5	7.6	0.4775	100	0.445

3,3,3-trifluoro-1-(2-hydroxyphenyl)propane-1,2-diol (3i)

3,3,3-trifluoro-1-(2-hydroxyphenyl)propane-1,2-diol ($NaBH_4$ reduction)
Chiralpak IA (25 cm) hexane/2-PrOH 90:10 (Volume of injection = 5 µL, 25 °C, 1 mL/min, sample dissolved in 2-PrOH).

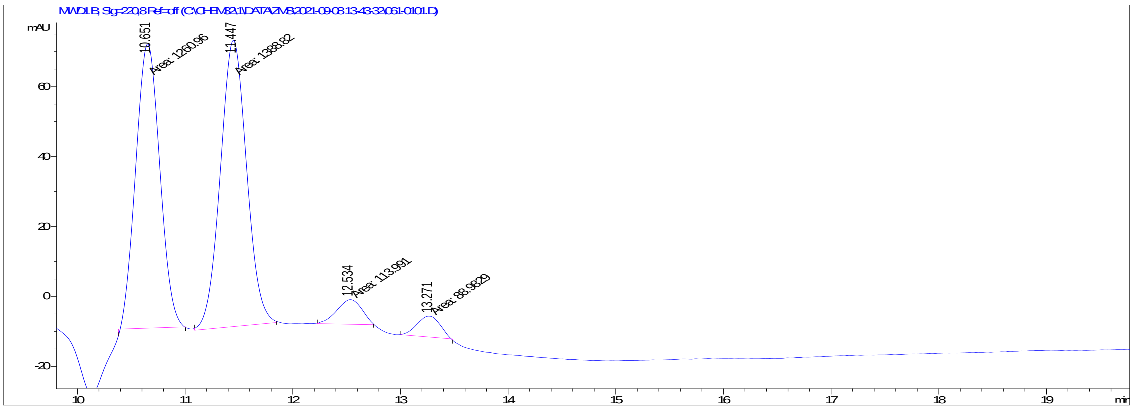

Peak	t_r (min)	Area	Height	Width	Area%	Symmetry
anti-**3i**	10.651	1261	81.3	0.2584	44.202	0.95
ent-anti-**3i**	11.447	1388.8	81.9	0.2827	48.683	0.987
(1*R*,2*S*)-**3i**	12.534	114	7.1	0.2689	3.996	1.181
(1*S*,2*R*)-**3i**	13.271	89	6	0.2481	3.119	0.983

(1*R*,2*S*)-3,3,3-trifluoro-1-(2-hydroxyphenyl)propane-1,2-diol ((*S*,*S*)-**C4**-cat., ee (*syn*) >99%)
Chiralpak IA (25 cm) hexane/2-PrOH 90:10 (Volume of injection = 5 µL, 25 °C, 1 mL/min, sample dissolved in 2-PrOH).

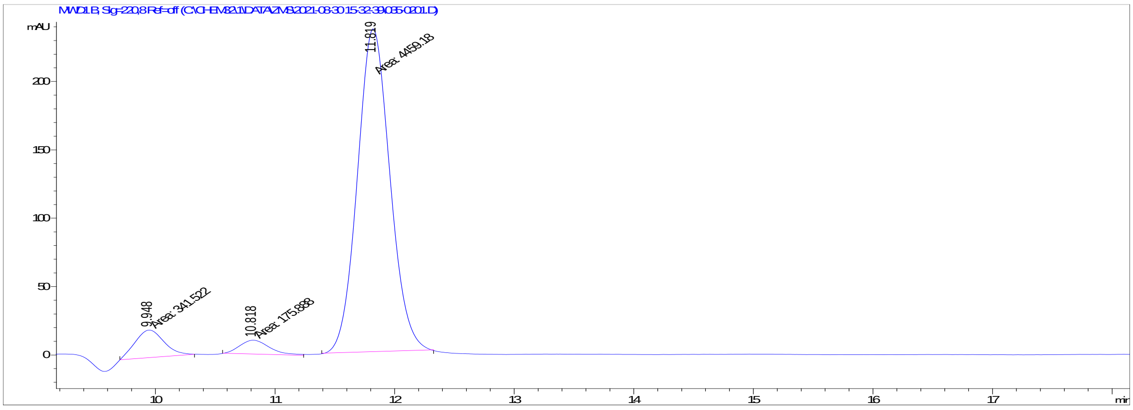

Peak	t_r (min)	Area	Height	Width	Area%	Symmetry
anti-**3i**	9.948	341.5	20.2	0.2814	6.863	0.932
ent-anti-**3i**	10.818	175.9	10.2	0.2888	3.534	0.781
(1*R*,2*S*)-**3i**	11.819	4459.2	236.3	0.3145	89.603	0.899

3,3,3-trifluoro-1-(2-hydroxyphenyl)propane-1,2-diol ($NaBH_4$ reduction)
Chiralpak IA (25 cm) hexane/2-PrOH 90:10 (Volume of injection = 5 µL, 25 °C, 1 mL/min, sample dissolved in 2-PrOH).

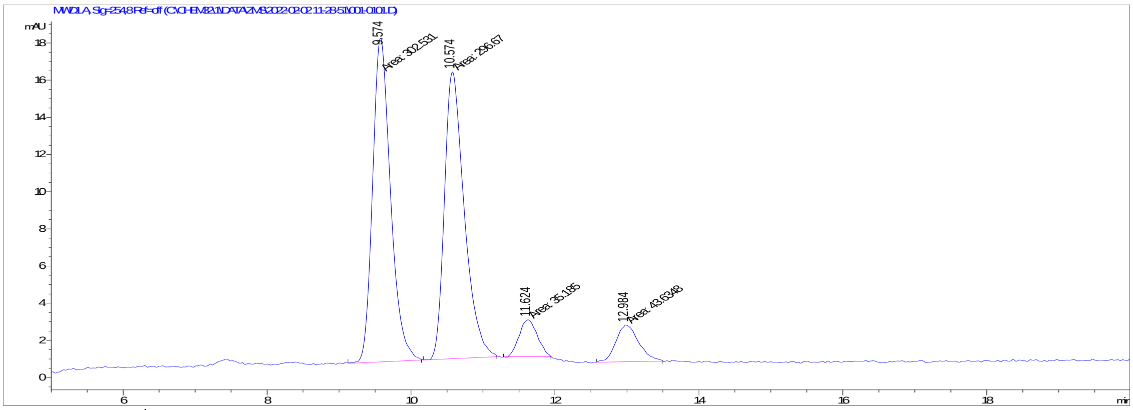

Peak	t_r (min)	Area	Height	Width	Area%	Symmetry
anti-**3i**	9.574	302.5	17.4	0.2893	44.62	0.773
ent-anti-**3i**	10.574	296.7	15.4	0.3203	43.755	0.641
(1*R*,2*S*)- **3i**	11.624	35.2	2	0.2945	5.189	0.92
(1*S*,2*R*)- **3i**	12.984	43.6	2	0.3669	6.436	0.768

(1*R*,2*S*)-3,3,3-trifluoro-1-(2-hydroxyphenyl)propane-1,2-diol ((*S*,*S*)-**C5**-cat., ee (*syn*) >99%)
Chiralpak IA (25 cm) hexane/2-PrOH 90:10 (Volume of injection = 5 µL, 25 °C, 1 mL/min, sample dissolved in 2-PrOH).

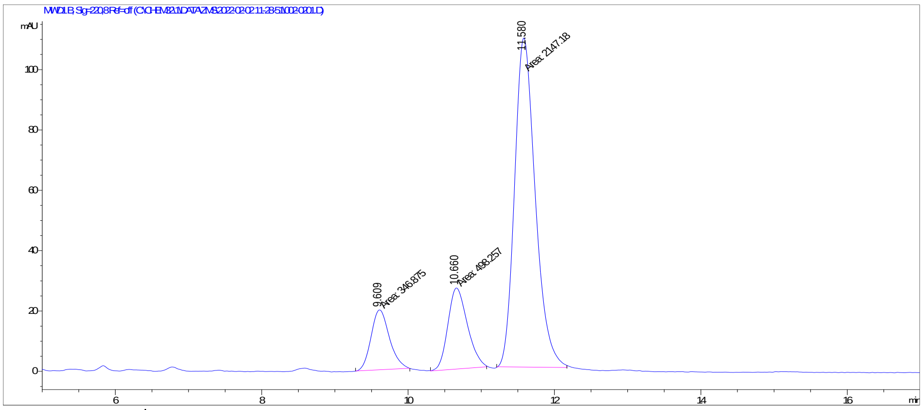

Peak	t_r (min)	Area	Height	Width	Area%	Symmetry
anti-**3i**	9.609	346.9	1.99E+01	0.291	11.592	0.802
ent-anti-**3i**	10.66	498.3	2.69E+01	0.3084	16.651	0.749
(1*R*,2*S*)-**3i**	11.58	2147.2	109.1	0.3279	71.757	0.755

3,3,3-trifluoro-1-(4-methoxyphenyl)propane-1,2-diol (3j)

3,3,3-trifluoro-1-(4-methoxyphenyl)propane-1,2-diol ($NaBH_4$ reduction)
Chiralpak IB-3 (25 cm) hexane/2-PrOH 98:2 to 90:10 in 20 min (Volume of injection = 5 µL, 25 °C, 1 mL/min, sample dissolved in 2-PrOH).

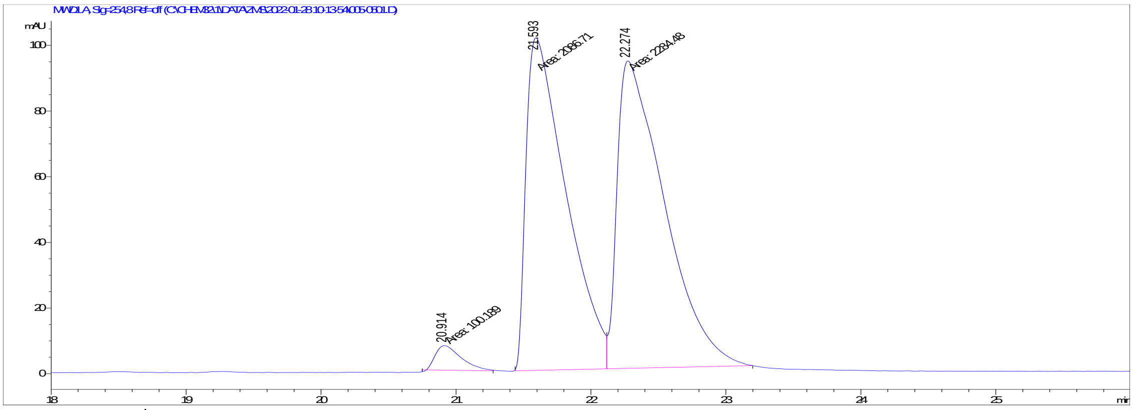

Peak	t_r (min)	Area	Height	Width	Area%	Symmetry
(1*R*,2*S*)-**3j**	20.914	100.2	7.5	0.2227	2.241	0.502
anti-**3j**	21.593	2086.7	101.4	0.343	46.668	0.315
(1*S*,2*R*)-**3j** +*ent-anti*-**3j**	22.274	2284.5	93.7	0.4062	51.091	0.281

A refference mixture of the DKR-ATH products from runs with (*S,S*)-**C4** and (*R,R*)-**C4**.
Chiralpak IB-3 (25 cm) hexane/2-PrOH 98:2 to 90:10 in 20 min (Volume of injection = 5 µL, 25 °C, 1 mL/min, sample dissolved in 2-PrOH).

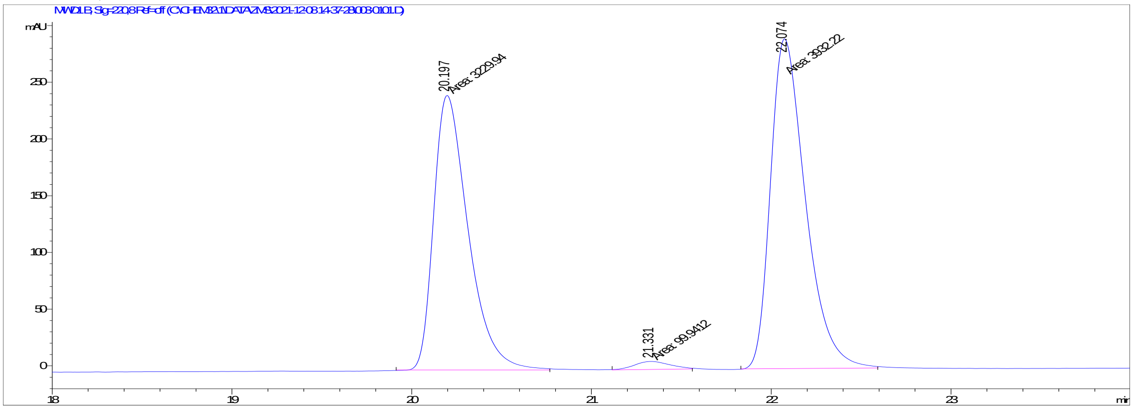

Peak	t_r (min)	Area	Height	Width	Area%	Symmetry
(1*R*,2*S*)-**3j**	20.197	3229.9	242.4	0.2221	44.477	0.625
anti-**3j**	21.331	99.9	7.2	0.2316	1.376	0.882
(1*S*,2*R*)- **3j** +*ent-anti*-**3j**	22.074	3932.2	291.2	0.225	54.147	0.637

(1*R*,2*S*)-3,3,3-trifluoro-1-(4-methoxyphenyl)propane-1,2-diol ((*S*,*S*)-**C4**-cat., ee (*syn*) ≥98%)
Chiralpak IB-3 (25 cm) hexane/2-PrOH 98:2 to 90:10 in 20 min (Volume of injection = 5 µL, 25 °C, 1 mL/min, sample dissolved in 2-PrOH).

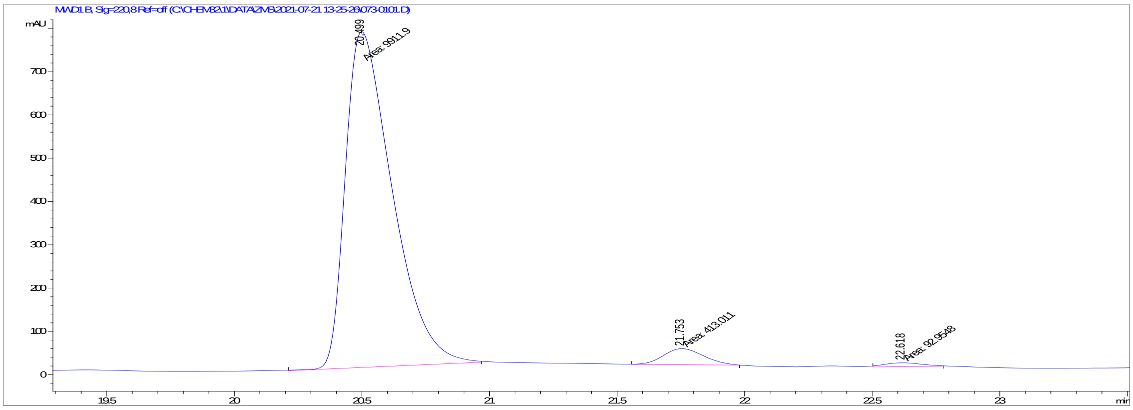

Peak	t_r (min)	Area	Height	Width	Area%	Symmetry
(1*R*,2*S*)- **3j**	20.499	9911.9	778	0.2123	95.143	0.484
anti-**3j**	21.753	413	37.5	0.1837	3.964	0.887
(1*S*,2*R*)-**3j** +*ent-anti*-**3j**	22.618	93	9.1	0.171	0.892	0.823

(1*R*,2*S*)-3,3,3-trifluoro-1-(4-methoxyphenyl)propane-1,2-diol ((*S*,*S*)-**C5**-cat., (ee *syn*) >99%)
Chiralpak IB-3 (25 cm) hexane/2-PrOH 98:2 to 90:10 in 20 min (Volume of injection = 5 µL, 25 °C, 1 mL/min, sample dissolved in 2-PrOH).

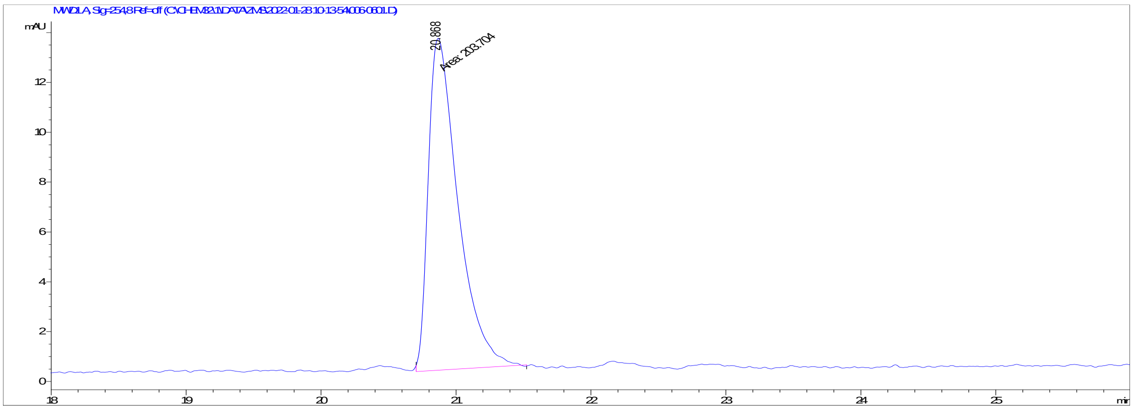

Peak	t_r (min)	Area	Height	Width	Area%	Symmetry
(1*R*,2*S*)- **3j**	20.868	203.7	13.3	0.2545	100	0.485

3,3,3-trifluoro-1,2-dihydroxypropyl)phenyl acetate (3k)

A refference mixture of the DKR-ATH products from runs with (*S*,*S*)-**C4** (pure by NMR) and (*R*,*R*)-**C4** (by NMR it contains 20% of deacetylated monoalcohol **2h**)
Chiralpak IA (25 cm) hexane/2-PrOH 90:10 (Volume of injection = 5 µL, 25 °C, 1 mL/min, sample dissolved in 2-PrOH).

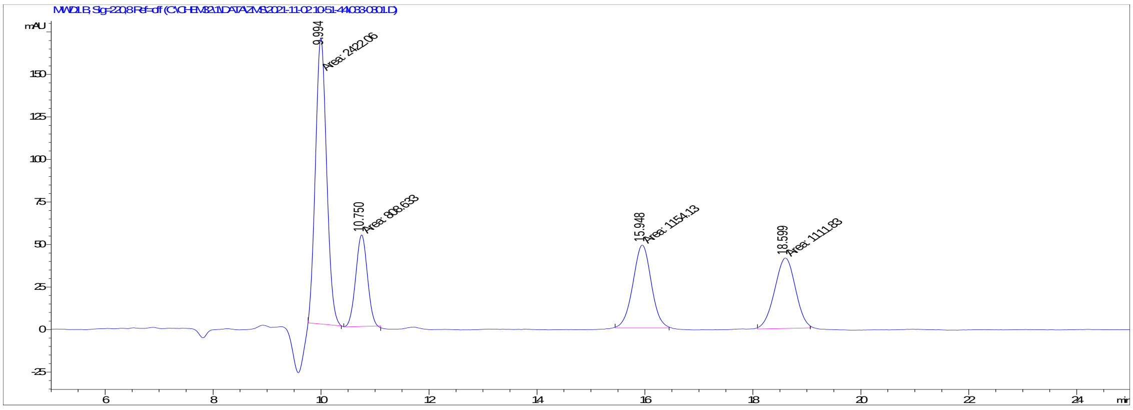

Peak	t_r (min)	Area	Height	Width	Area%	Symmetry
(1*S*,2*R*)-**3k**	9.994	2422.1	168.5	0.2396	44.064	0.877
(1*R*,2*S*)-**3k**	10.75	808.6	53.9	0.2499	14.711	0.97
**2h**	15.948	1154.1	48.8	0.394	20.997	0.994
ent-**2h**	18.599	1111.8	41.4	0.4471	20.227	1.033

3-((1*R*,2*S*)-3,3,3-trifluoro-1,2-dihydroxypropyl)phenyl acetate ((*S*,*S*)-**C4**-cat., ee (*syn*) = 96%)
Chiralpak IA (25 cm) hexane/2-PrOH 90:10 (Volume of injection = 5 µL, 25 °C, 1 mL/min, sample dissolved in 2-PrOH).

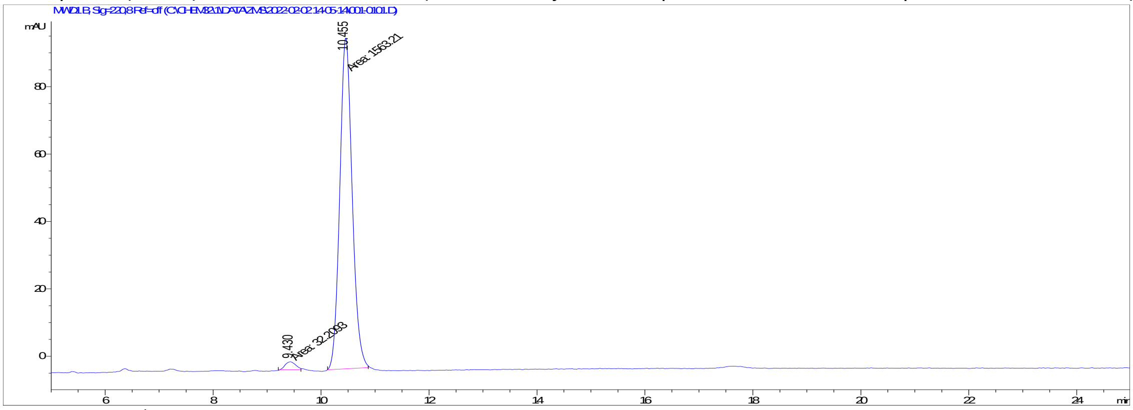

Peak	t_r (min)	Area	Height	Width	Area%	Symmetry
(1*S*,2*R*)-**3k**	9.43	32.2	2.40	0.2268	2.019	0.891
(1*R*,2*S*)-**3k**	10.455	1563.2	98.2	0.2653	97.981	0.865

3-((1*R*,2*S*)-3,3,3-trifluoro-1,2-dihydroxypropyl)phenyl acetate ((*S*,*S*)-**C5**-cat., ee (*syn*) = 97%)
Chiralpak IA (25 cm) hexane/2-PrOH 90:10 (Volume of injection = 5 µL, 25 °C, 1 mL/min, sample dissolved in 2-PrOH).

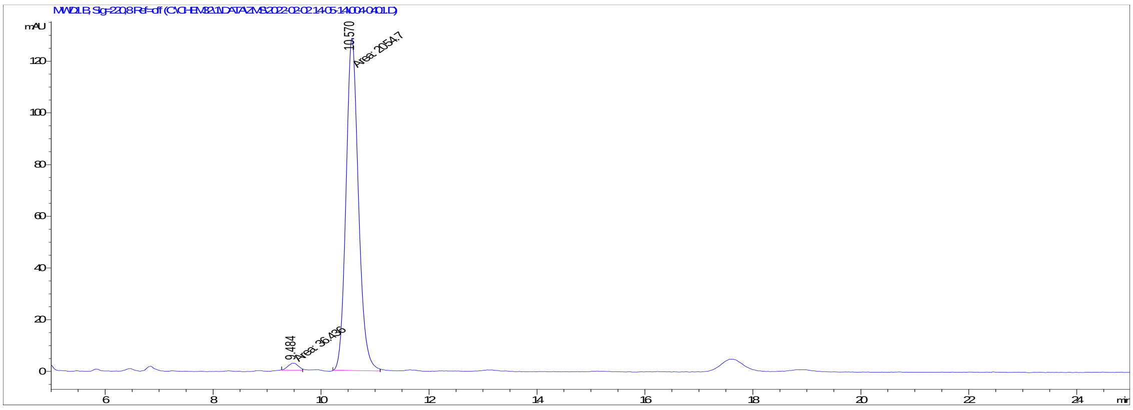

Peak	t_r (min)	Area	Height	Width	Area%	Symmetry
(1*S*,2*R*)-**3k**	9.484	36.4	2.7	0.2225	1.742	1.013
(1*R*,2*S*)-**3k**	10.57	2054.7	128.6	0.2663	98.258	0.862

3,3,3-trifluoro-1-(4-(hydroxymethyl)phenyl)propane-1,2-diol (3l)

3,3,3-trifluoro-1-(4-(hydroxymethyl)phenyl)propane-1,2-diol ($NaBH_4$ reduction)
Chiralpak IB-3 (25 cm) hexane/2-PrOH 90:10 in 50 min (Volume of injection = 5 µL, 25 °C, 1 mL/min, sample dissolved in 2-PrOH).

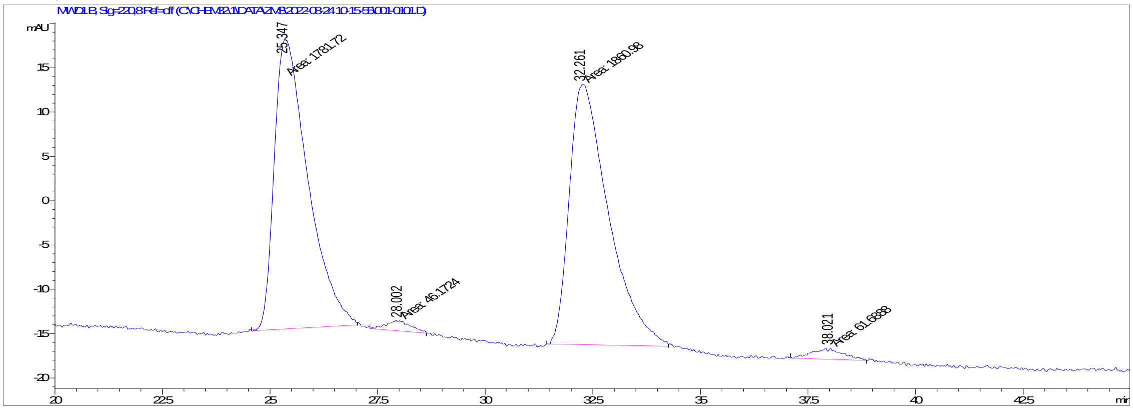

Peak	t_r (min)	Area	Height	Width	Area%	Symmetry
anti-**3l**	25.347	1781.7	32.7	0.9082	47.505	0.455
(1*R*,2*S*)-**3l**	28.002	46.2	1.2	0.665	1.231	0.633
ent-anti-**3l**	32.261	1861	29.4	1.0564	49.619	0.494
(1*S*,2*R*)-**3l**	38.021	61.7	1.3	0.8141	1.645	1.261

(1*R*,2*S*)-3,3,3-trifluoro-1-(4-(hydroxymethyl)phenyl)propane-1,2-diol ((*S*,*S*)-**C4**-cat., ee (*syn*) >99%)

Chiralpak IB-3 (25 cm) hexane/2-PrOH 90:10 in 50 min (Volume of injection = 5 µL, 25 °C, 1 mL/min, sample dissolved in 2-PrOH).

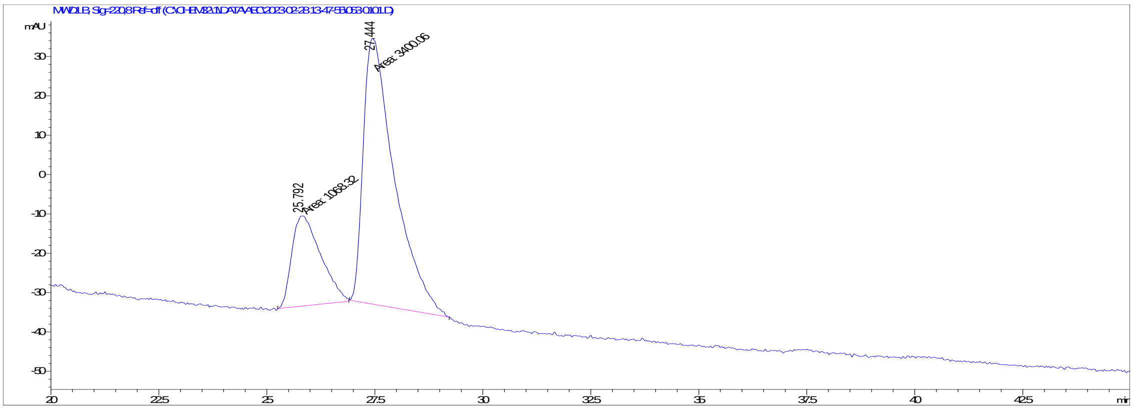

Peak	t_r (min)	Area	Height	Width	Area%	Symmetry
anti-**3l**	25.792	1068.3	23	0.7749	23.908	0.469
(1*R*,2*S*)-**3l**	27.444	3400.1	67.6	0.8384	76.092	0.402

(1*R*,2*S*)-3,3,3-trifluoro-1-(4-(hydroxymethyl)phenyl)propane-1,2-diol ((*S*,*S*)-**C5**-cat., ee (*syn*) >99%)

Chiralpak IB-3 (25 cm) hexane/2-PrOH 90:10 in 50 min (Volume of injection = 5 µL, 25 °C, 1 mL/min, sample dissolved in 2-PrOH).

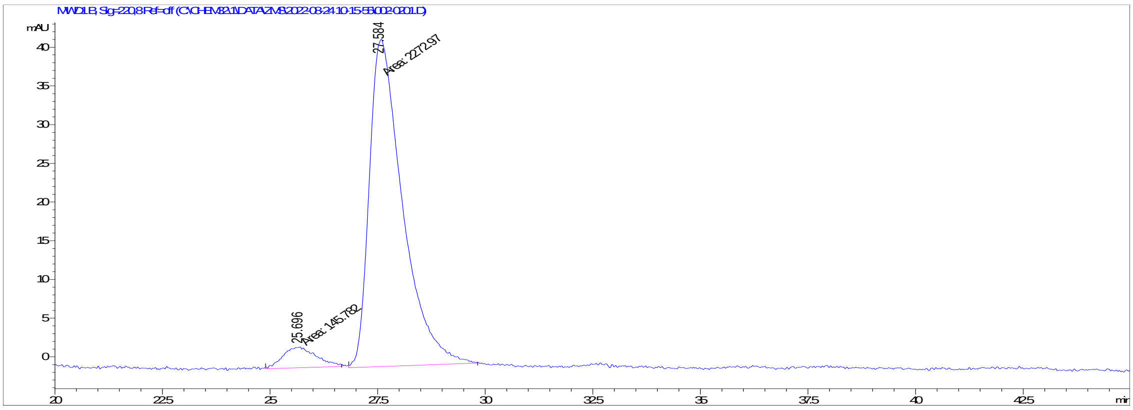

Peak	t_r (min)	Area	Height	Width	Area%	Symmetry
anti-**3l**	25.696	145.8	2.7	0.8935	6.027	1.015
(1*R*,2*S*)-**3l**	27.584	2273	42.3	0.8956	93.973	0.538

3,3,3-trifluoro-1-(pyridin-3-yl)propane-1,2-diol (3m)

A refference mixture of the DKR-ATH products from runs with (*S,S*)-**C4** and (*R,R*)-**C4**
GC analysis using CP-ChiraSil-DEX CB column (25 m x 0.25 cm), isothermal elution with helium at 150 °C

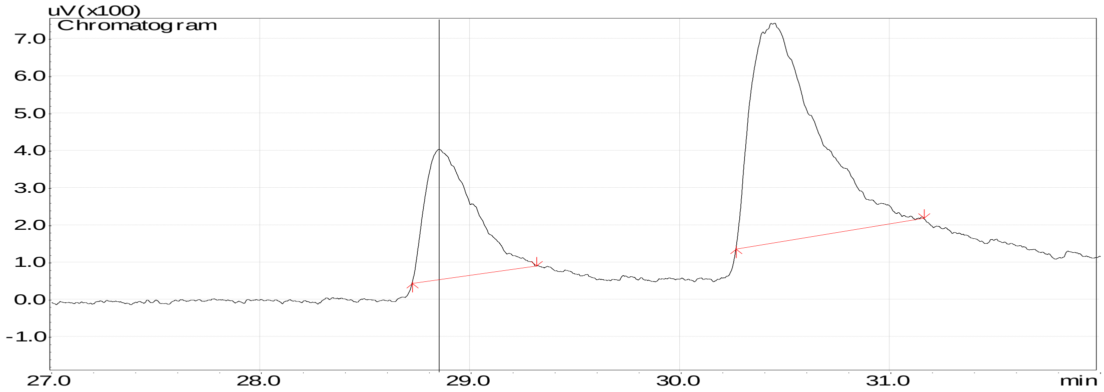

Peak	t_r (min)	Area	Height	Area %
(1*S*,2*R*)-**3m**	28.847	5623.5	349.7	30.2549
(1*R*,2*S*)-**3m**	30.447	12963.5	588.9	69.7451

(1*R*,2*S*)-3,3,3-trifluoro-1-(pyridin-3-yl)propane-1,2-diol ((*S*,*S*)-**C4**-cat., ee (*syn*) = 99%)

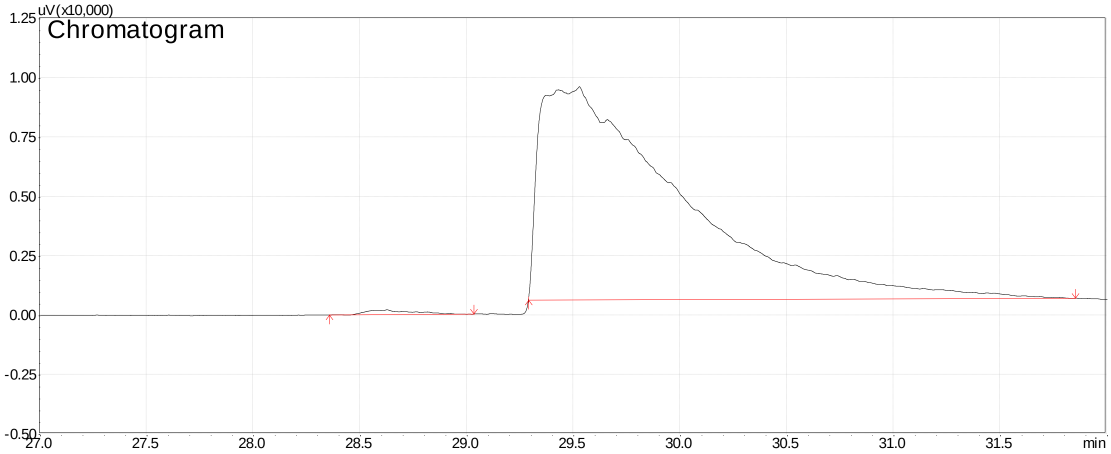

Peak	t_r (min)	Area	Height	Area %
(1*S*,2*R*)-**3m**	28.625	3009.1	202.3	0.7177
(1*R*,2*S*)-**3m**	29.526	416290.7	8970.8	99.2823

1-(2-aminobenzo[*d*]thiazol-6-yl)-3,3,3-trifluoropropane-1,2-diol (3n)

A refference mixture of the DKR-ATH products from runs with (*S,S*)-**C4** and (*R,R*)-**C4**

Chiralpak IB-3 (25 cm) hexane/2-PrOH + 0.1% Et_3N 85:15 (Volume of injection = 5 µL, 25 °C, 1 mL/min, sample dissolved in 2-PrOH).

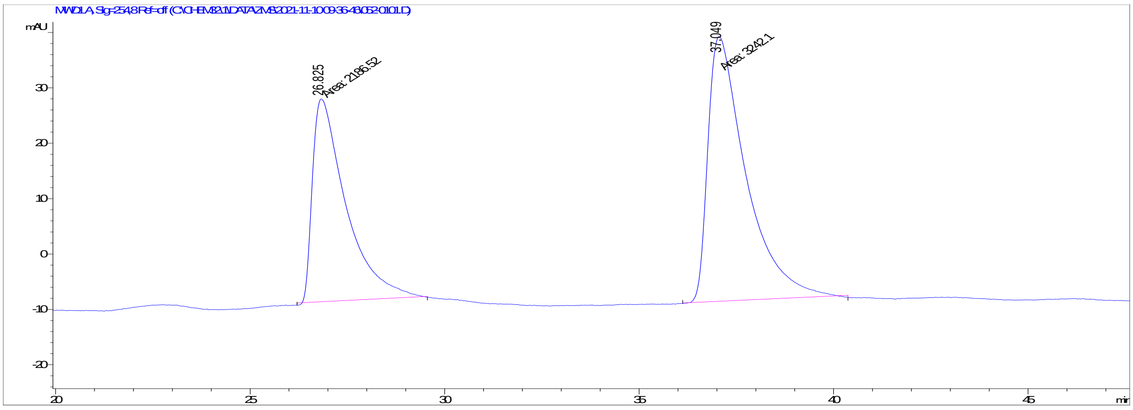

Peak	t_r (min)	Area	Height	Width	Area%	Symmetry
(1*R*,2*S*)-**3n**	26.825	2186.5	36.6	0.9946	40.278	0.344
(1*S*,2*R*)-**3n**	37.049	3242.1	47.8	1.1297	59.722	0.392

(1*R*,2*S*)-1-(2-aminobenzo[*d*]thiazol-6-yl)-3,3,3-trifluoropropane-1,2-diol ((*S*,*S*)-**C4**-cat., ee (*syn*) >99%)
Chiralpak IB-3 (25 cm) hexane/2-PrOH + 0.1% Et_3N 85:15 (Volume of injection = 5 µL, 25 °C, 1 mL/min, sample dissolved in 2-PrOH).

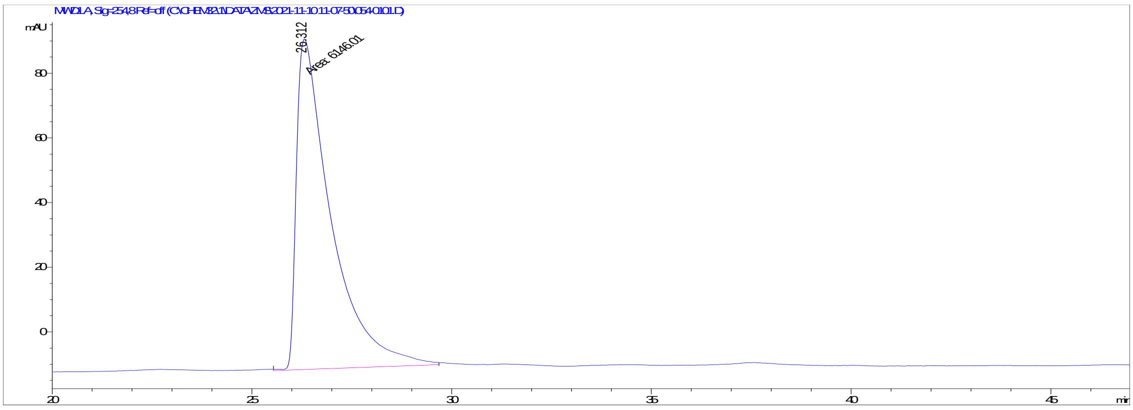

Peak	t_r (min)	Area	Height	Width	Area%	Symmetry
(1*R*,2*S*)-**3n**	26.312	6146	102.2	1.0019	100	0.283

A refference mixture of the DKR-ATH products from runs with (*S,S*)-**C4** and (*R,R*)-**C4**

Chiralpak IB-3 (25 cm) hexane/2-PrOH + 0.1% Et_3N 85:15 (Volume of injection = 5 µL, 25 °C, 1 mL/min, sample dissolved in 2-PrOH).

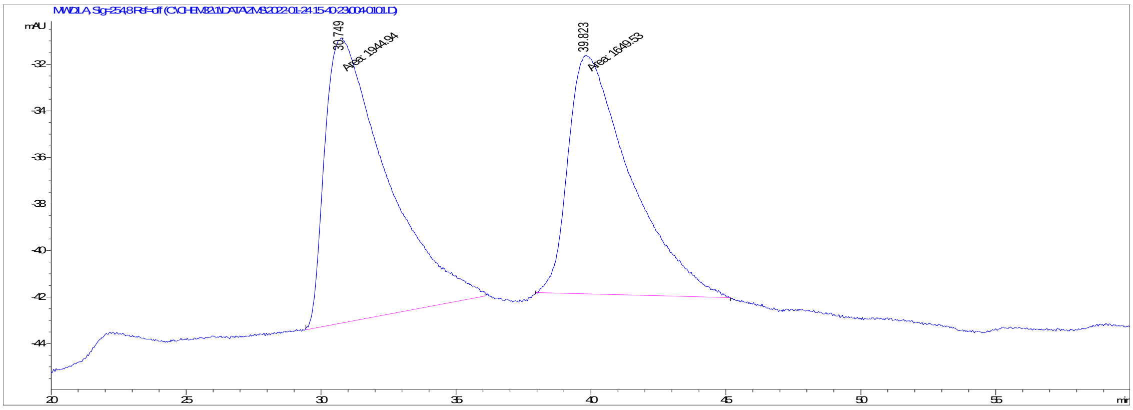

Peak	t_r (min)	Area	Height	Width	Area%	Symmetry
(1*R*,2*S*)-**3n**	30.749	1944.9	12.3	2.6458	54.109	0.355
(1*S*,2*R*)-**3n**	39.823	1649.5	10.3	2.6798	45.891	0.4

(1*R*,2*S*)-1-(2-aminobenzo[*d*]thiazol-6-yl)-3,3,3-trifluoropropane-1,2-diol ((*S,S*)-**C5**-cat., ee (*syn*) >99%)
Chiralpak IB-3 (25 cm) hexane/2-PrOH + 0.1% Et_3N 85:15 (Volume of injection = 5 µL, 25 °C, 1 mL/min, sample dissolved in 2-PrOH).

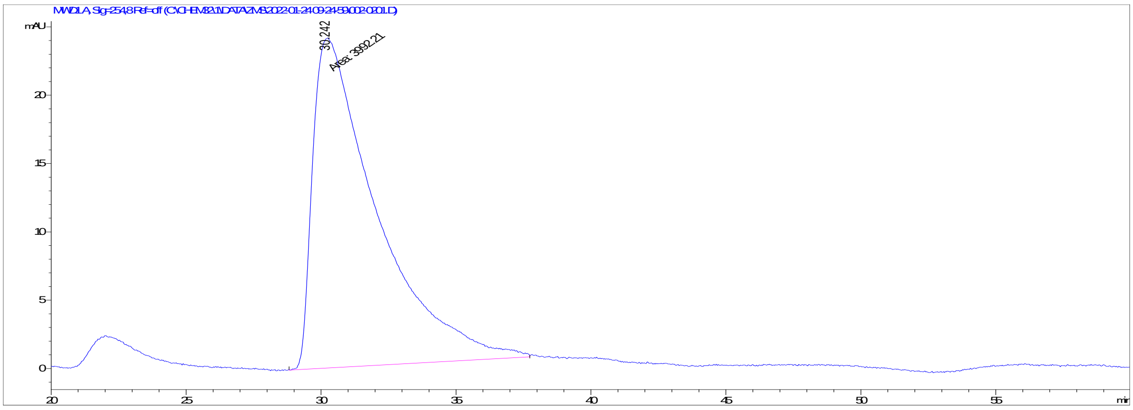

Peak	t_r (min)	Area	Height	Width	Area%	Symmetry
(1*R*,2*S*)-**3n**	30.242	3992.2	24.2	2.7544	100	0.294

1,1,1-trifluoro-4-phenylbutane-2,3-diol (3o)

1,1,1-trifluoro-4-phenylbutane-2,3-diol ($NaBH_4$ reduction)
GC analysis using CP-ChiraSil-DEX CB column (25 m x 0.25 cm), isothermal elution with helium at 140 °C

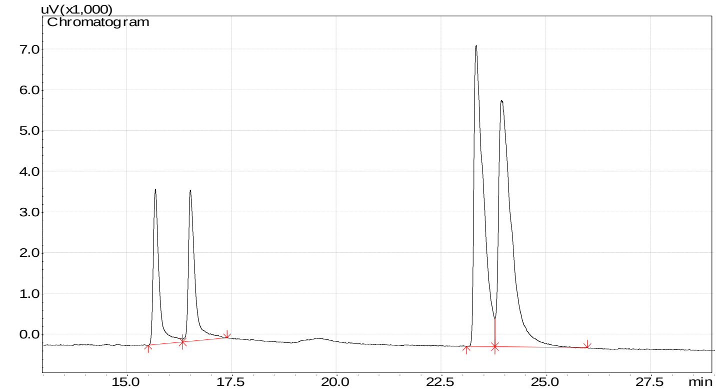

Peak	t_r (min)	Area	Height	Area %
(2*R*,3*S*)-**3o**	15.668	37799.7	3814.7	11.6649
(2*S*,3*R*)-**3o**	16.502	40277.5	3711.9	12.4295
anti-**3o**	23.318	115379.2	7393.3	35.6056
ent-anti-**3o**	23.924	130591.5	6052.5	40.3001

(2*S*,3*R*)-1,1,1-trifluoro-4-phenylbutane-2,3-diol ((*S*,*S*)-**C4**-cat., ee (*syn*) = 95%)
CP-ChiraSil-DEX CB column (25 m x 0.25 cm); isothermal elution with He, T = 140 °C: temperature of injection = 220 °C

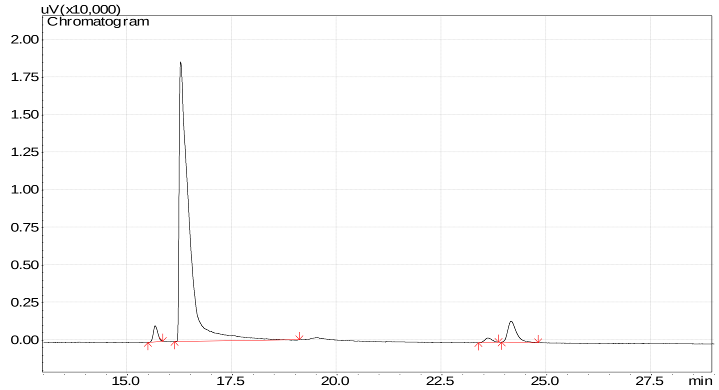

Peak	t_r (min)	Area	Height	Area %
(2*R*,3*S*)-**3o**	15.661	8113.1	1080.3	2.4210
(2*S*,3*R*)-**3o**	16.268	302708.9	18628.3	90.3315
anti-**3o**	23.591	3785.7	316.6	1.1297
ent-anti-**3o**	24.146	20501.3	1417.5	6.1178

(2*S*,3*R*)-1,1,1-trifluoro-4-phenylbutane-2,3-diol ((*S*,*S*)-**C5**-cat., ee (*syn*)= 96%)

CP-ChiraSil-DEX CB column (25 m x 0.25 cm); isothermal elution with He, T = 140 °C: temperature of injection = 220 °C

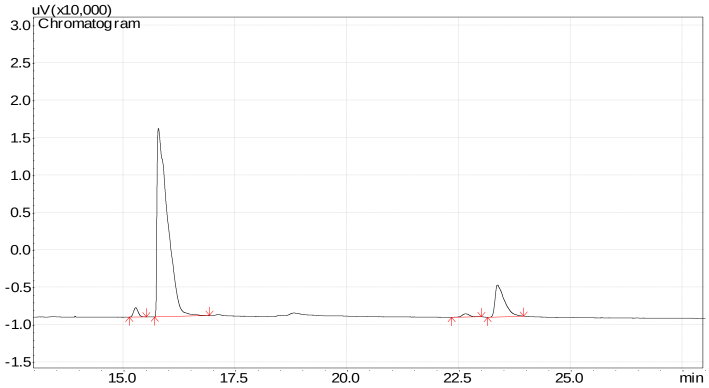

Peak	t_r (min)	Area	Height	Area %
(2*R*,3*S*)-**3o**	15.265	9223.4	1261.3	1.8871
(2*S*,3*R*)-**3o**	15.773	412119.6	25177.6	84.3185
anti-**3o**	22.639	5185.8	436.7	1.0610
ent-anti-**3o**	23.358	62236.8	4254.1	12.7335

1,1,1-trifluorononane-2,3-diol (3p)

1,1,1-trifluorononane-2,3-diol ($NaBH_4$ reduction)
CP-ChiraSil-DEX CB column (25 m x 0.25 cm); isothermal elution with He, T = 140 °C: temperature of injection = 220 °C

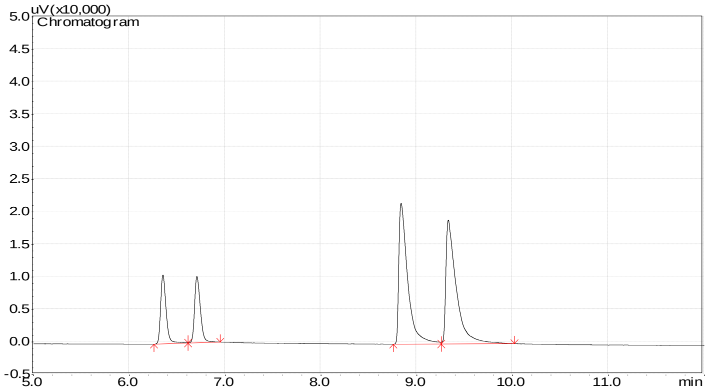

Peak	t_r (min)	Area	Height	Area %
(2*R*,3*S*)-**3p**	6.351	42792.4	10603.7	11.5132
(2*S*,3*R*)-**3p**	6.705	43404.4	10208.9	11.6778
anti-**3p**	8.837	141302.1	21640.9	38.0168
ent-anti-**3p**	9.329	144184.0	19035.4	38.7922

(2*S*,3*R*)-1,1,1-trifluorononane-2,3-diol ((*S*,*S*)-**C4**-cat., ee (*syn*) = 96%)
CP-ChiraSil-DEX CB column (25 m x 0.25 cm); isothermal elution with He, T = 140 °C: temperature of injection = 220 °C

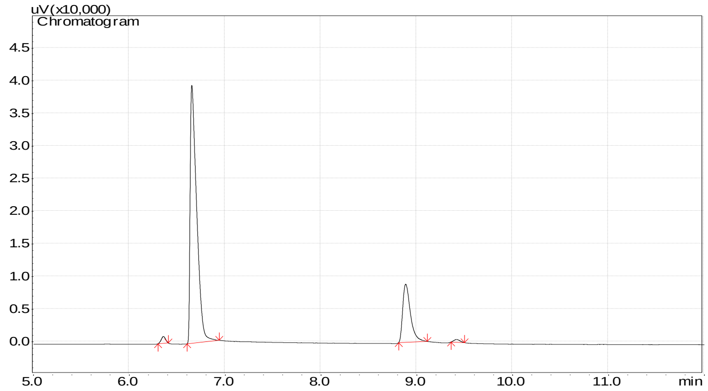

Peak	t_r (min)	Area	Height	Area %
(2*R*,3*S*)-**3p**	6.356	3459.8	1073.6	1.4231
(2*S*,3*R*)-**3p**	6.653	187171.2	39506.3	76.9897
anti-**3p**	8.884	50404.3	8945.4	20.7330
ent-anti-**3p**	9.413	2076.8	454.3	0.8542

(2*S*,3*R*)-1,1,1-trifluorononane-2,3-diol ((*S*,*S*)-**C5**-cat., ee (*syn*) = 98%)
CP-ChiraSil-DEX CB column (25 m x 0.25 cm); isothermal elution with He, T = 140 °C: temperature of injection = 220 °C

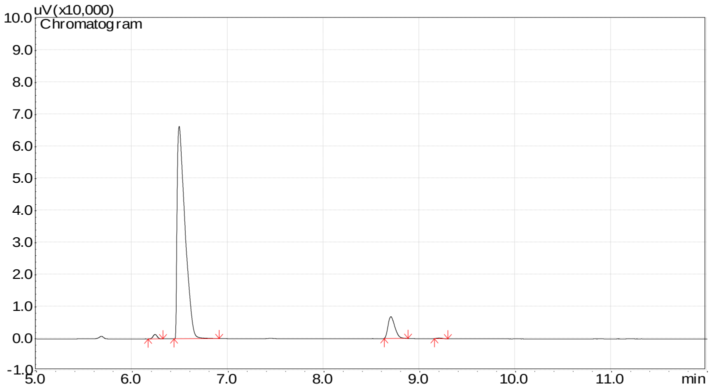

Peak	t_r (min)	Area	Height	Area %
(2*R*,3*S*)-**3p**	6.237	4687.5	1475.7	1.1167
(2*S*,3*R*)-**3p**	6.489	380215.3	66381.9	90.5815
anti-**3p**	8.698	33913.3	6818.4	8.0794
ent-anti-**3p**	9.203	933.4	223.9	0.2224

6,6,6-trifluoro-4,5-dihydroxyhexyl)benzamide (3q)

***N*-(6,6,6-trifluoro-4,5-dihydroxyhexyl)benzamide** ($NaBH_4$ reduction)
Chiralpak IB-3 (25 cm) hexane/2-PrOH 91:9 in 60 min (Volume of injection = 5 µL, 25 °C, 1 mL/min, sample dissolved in 2-PrOH).

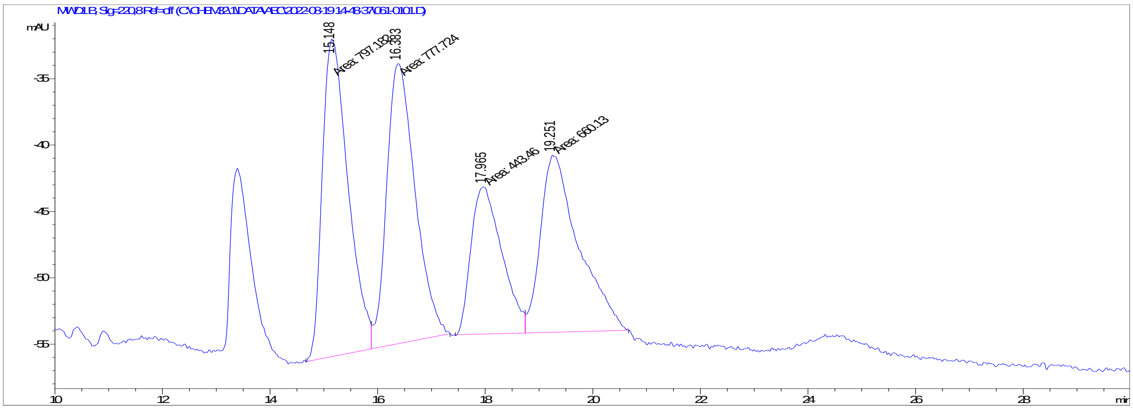

Peak	t_r (min)	Area	Height	Width	Area%	Symmetry
anti-**3q**	15.148	797.2	23.9	0.5552	29.762	0.589
ent-anti-**3q**	16.383	777.7	21.1	0.6138	29.036	0.669
(4*R*,5*S*)-**3q**	17.965	443.5	11.1	0.6664	16.556	0.651
(4*S*,5*R*)-**3q**	19.251	660.1	13.3	0.8243	24.646	0.456

***N*-((4*R*,5*S*)-6,6,6-trifluoro-4,5-dihydroxyhexyl)benzamide** ((*S*,*S*)-**C4**-cat., ee (*syn*) = 70%)

Chiralpak IB-3 (25 cm) hexane/2-PrOH 91:9 in 60 min (Volume of injection = 5 µL, 25 °C, 1 mL/min, sample dissolved in 2-PrOH)

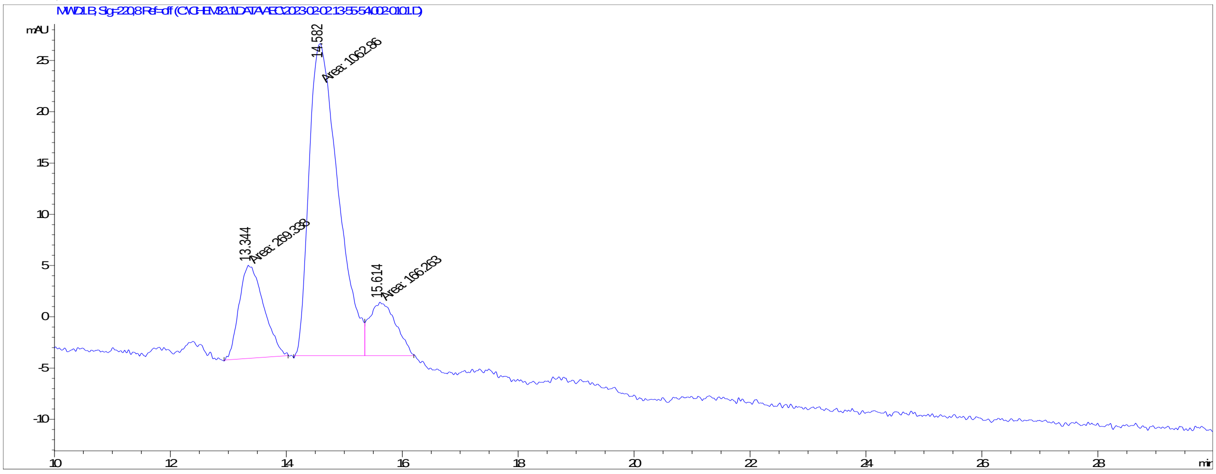

Peak	t_r (min)	Area	Height	Width	Area%	Symmetry
ent-anti-**3q**	13.344	269.3	9.1	0.4925	17.974	0.598
(4*R*,5*S*)-**3q**	14.582	1062.9	30.5	0.5814	70.93	0.589
(4*S*,5*R*)-**3q**	15.614	166.3	5.2	0.5292	11.096	0.639

***N*-((4*R*,5*S*)-6,6,6-trifluoro-4,5-dihydroxyhexyl)benzamide** ((*S*,*S*)-**C5**-cat., ee (*syn*) = 96%)

Chiralpak IB-3 (25 cm) hexane/2-PrOH 91:9 in 60 min (Volume of injection = 5 µL, 25 °C, 1 mL/min, sample dissolved in 2-PrOH).

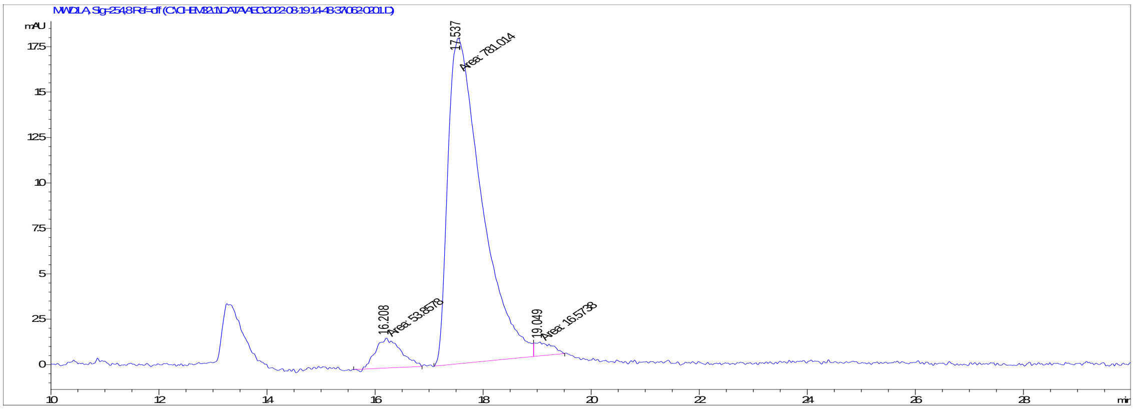

Peak	t_r (min)	Area	Height	Width	Area%	Symmetry
ent-anti-**3q**	16.208	53.9	1.7	0.5428	6.325	0.734
(4*R*,5*S*)-**3q**	17.537	781	17.9	0.7256	91.728	0.418
(4*S*,5*R*)-**3q**	19.049	16.6	7.80E-01	0.3562	1.947	0.44

***syn*-(*E*)-1,1,1-trifluoro-5-phenylpent-4-ene-2,3-diol (3r)** A reference mixture prepared by mixing the DKR-ATH pfoducts from runs with (*R*,*R*)-**C4** and (*S*,*S*)-**C5**.

Chiralpak IA (25 cm) hexane/2-PrOH 90:10 (Volume of injection = 5 µL, 25 °C, 1 mL/min, sample dissolved in chloroform)

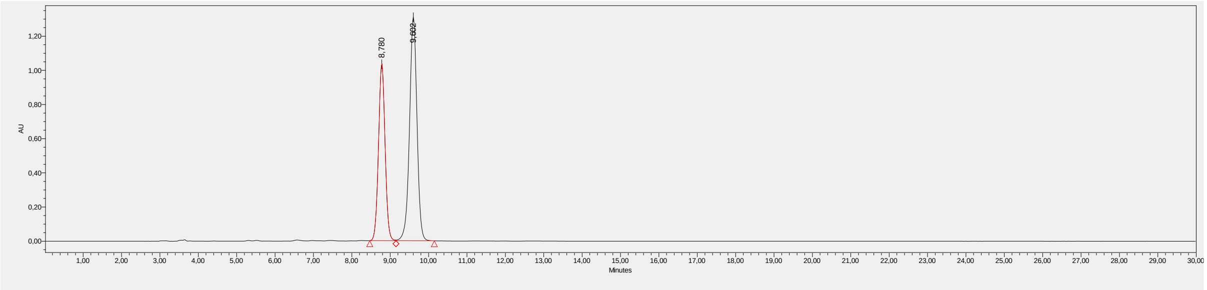

	Name	Retention Time	Area	% Area	Height
1	**(2*R*,3*S*)-3r**	8,780	12316194	41,01	1036358
2	**(2*S*,3*R*)-3r**	9,602	17714929	58,99	1310263

(2*R*,3*S*)-*syn*-(*E*)-1,1,1-trifluoro-5-phenylpent-4-ene-2,3-diol (3r) DKR-ATH product from run with (*R*,*R*)-**C4**; 97% ee.

Chiralpak IA (25 cm) hexane/2-PrOH 90:10 (Volume of injection = 5 µL, 25 °C, 1 mL/min, sample dissolved in chloroform)

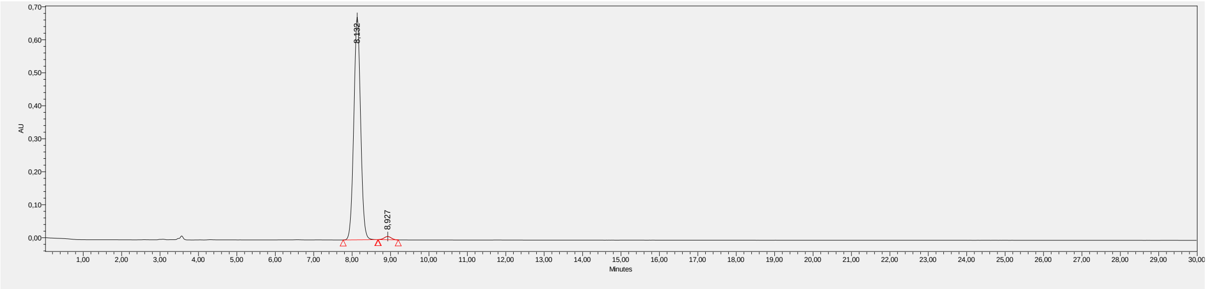

	Name	Retention Time	Area	% Area	Height
1	**(2*R*,3*S*)-3r**	8,132	8271323	98,51	674656
2	**(2*S*,3*R*)-3r**	8,927	125007	1,49	9920

(2*S*,3*R*)-*syn*-(*E*)-1,1,1-trifluoro-5-phenylpent-4-ene-2,3-diol (3r) DKR-ATH product from run with (*S*,*S*)-**C5**; 98% ee.

Chiralpak IA (25 cm) hexane/2-PrOH 90:10 (Volume of injection = 5 µL, 25 °C, 1 mL/min, sample dissolved in chloroform)

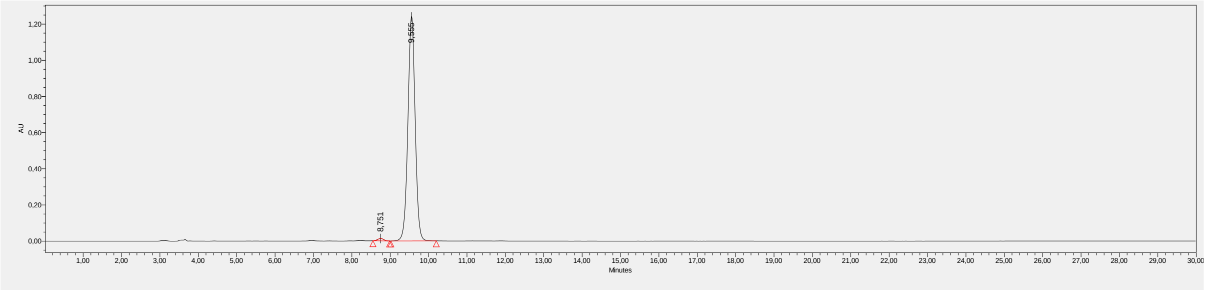

	Name	Retention Time	Area	% Area	Height
1	**(2*R*,3*S*)-3r**	8,751	143425	0,87	13174
2	**(2*S*,3*R*)-3r**	9,555	16321243	99,13	1243466

3,4-dichloro-5-methyl-*N*-(6-(3,3,3-trifluoro-1,2-dihydroxypropyl)benzo[*d*]thiazol-2-yl)-1*H*-pyrrole-2-carboxamide (6)

(±)-*anti*-**6**/(±)-*syn*-**6** = 73:27.

Chiralpak IA (25 cm) hexane/2-PrOH 85:15 (Volume of injection = 5 µL, 25 °C, 1 mL/min, sample dissolved in 2-PrOH).

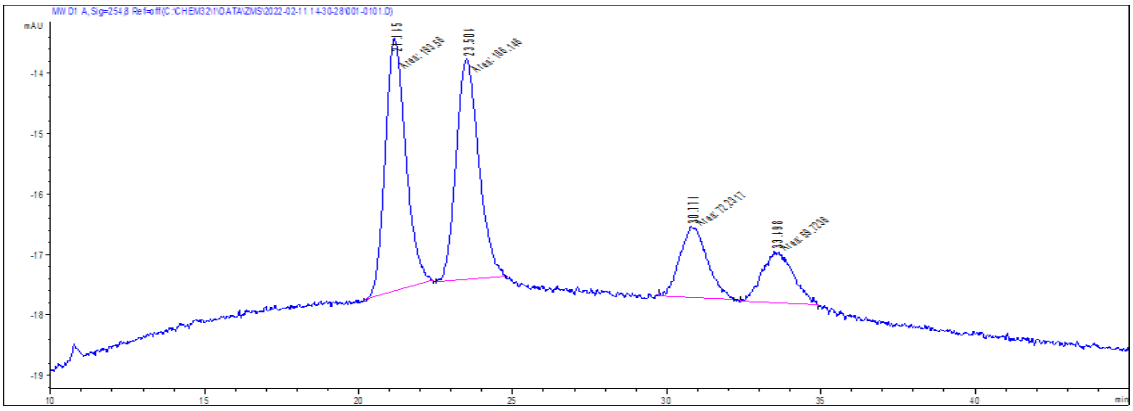

Peak	t_r (min)	Area	Height	Width	Area%	Symmetry
anti-**6**	21.145	193.6	4.2	0.7701	37.83	0.739
ent-anti-**6**	23.504	186.1	3.7	0.8488	36.381	0.841
(1*S*,2*R*)-**6**	30.771	72.2	1.2	1.0253	14.117	0.647
(1*R*,2*S*)-**6**	33.498	59.7	8.50E-01	1.1686	11.673	0.692

(1*R*,2*S*)-**6**, >99% ee.

Chiralpak IA (25 cm) hexane/2-PrOH 85:15 (Volume of injection = 5 µL, 25 °C, 1 mL/min, sample dissolved in 2-PrOH).

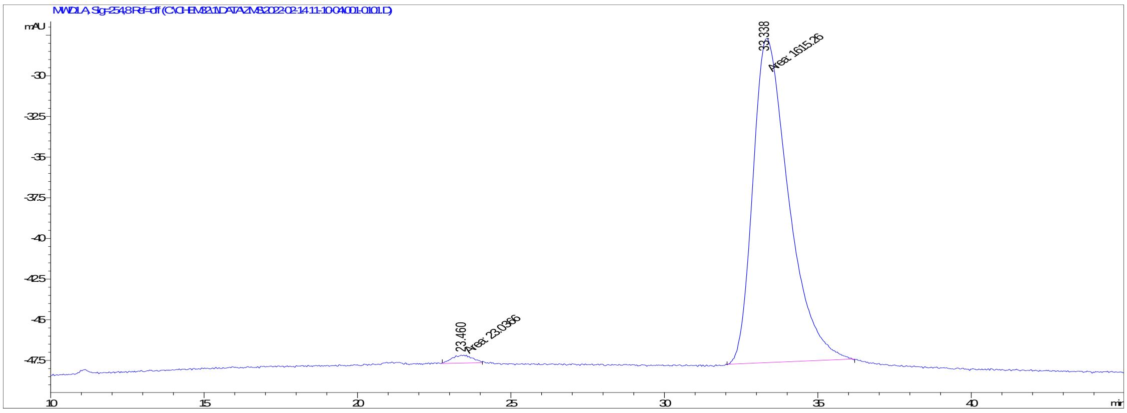

Peak	t_r (min)	Area	Height	Width	Area%	Symmetry
ent-anti-**6**	23.46	23	5.00E-01	0.7747	1.406	1.272
(1*R*,2*S*)-**6**	33.338	1615.3	19.9	1.3499	98.594	0.623

(1*S*,2*R*)-**6**, 99% ee.

Chiralpak IA (25 cm) hexane/2-PrOH 85:15 in 50 min (Volume of injection = 5 µL, 25 °C, 1 mL/min, sample dissolved in 2-PrOH).

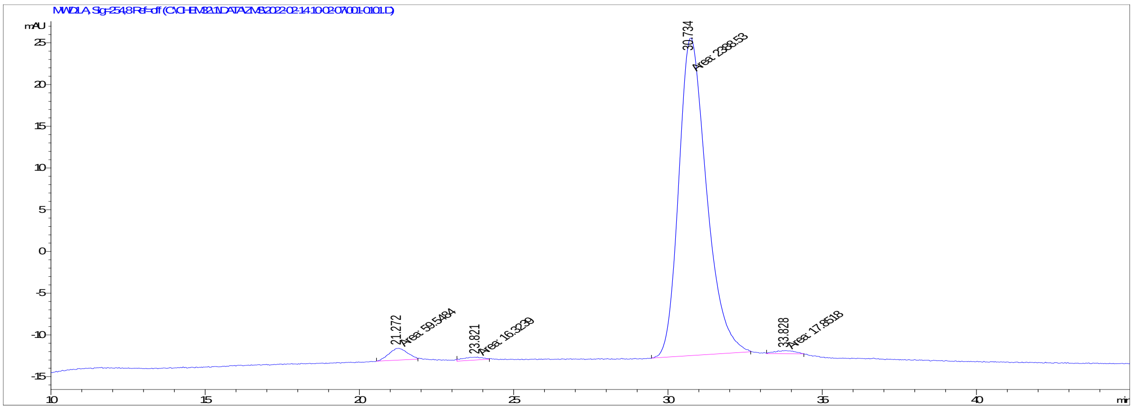

Peak	t_r (min)	Area	Height	Width	Area%	Symmetry
anti-**6**	21.272	59.5	1.4	0.6973	2.399	1.096
ent-anti-**6**	23.821	16.3	3.60E-01	0.7573	0.658	2.801
(1*S*,2*R*)-**6**	30.734	2388.5	3.81E+01	1.046	96.224	0.723
(1*R*,2*S*)-**6**	33.828	17.9	3.80E-01	0.7748	0.719	1.199

7 NMR spectra

2-hydroxy-2-(pyridin-3-yl)acetic acid, ^{1}H NMR:

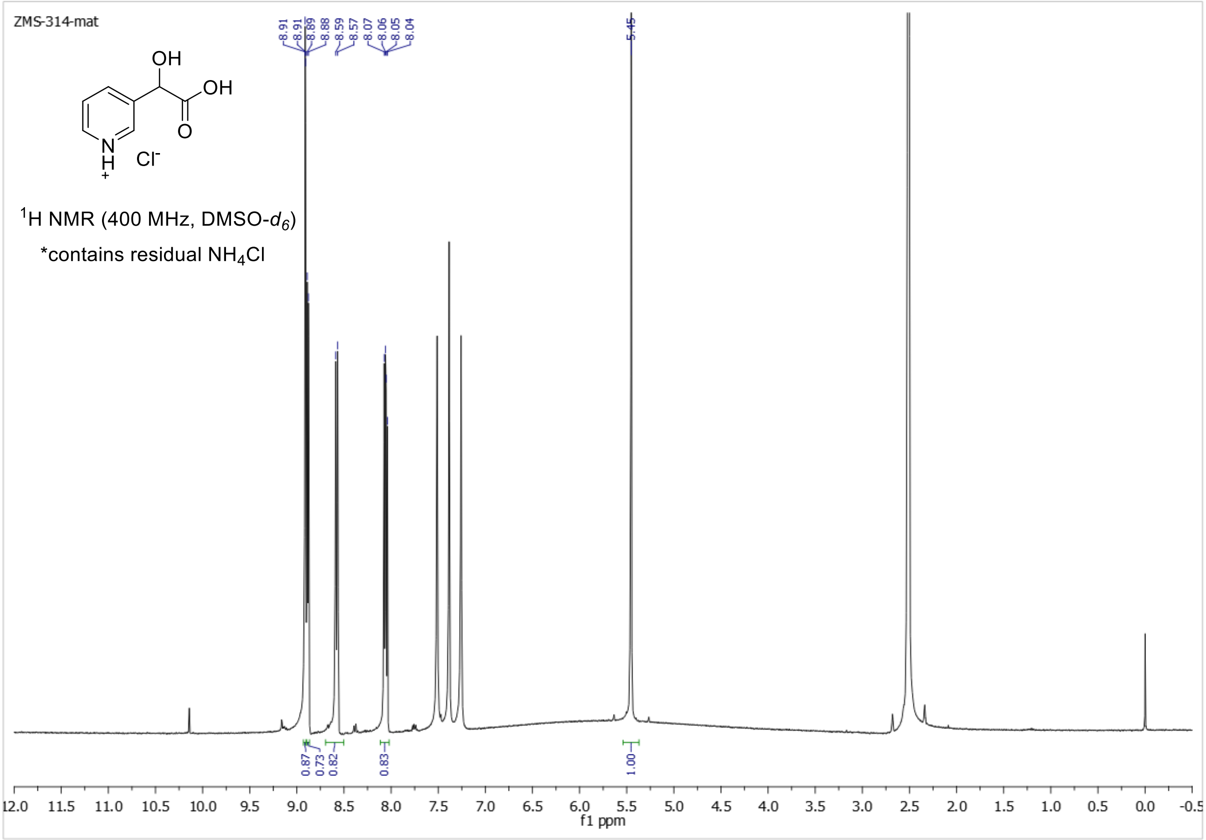

2-(4-bromobenzylidene)-1,1-dimethylhydrazine, 1H NMR:

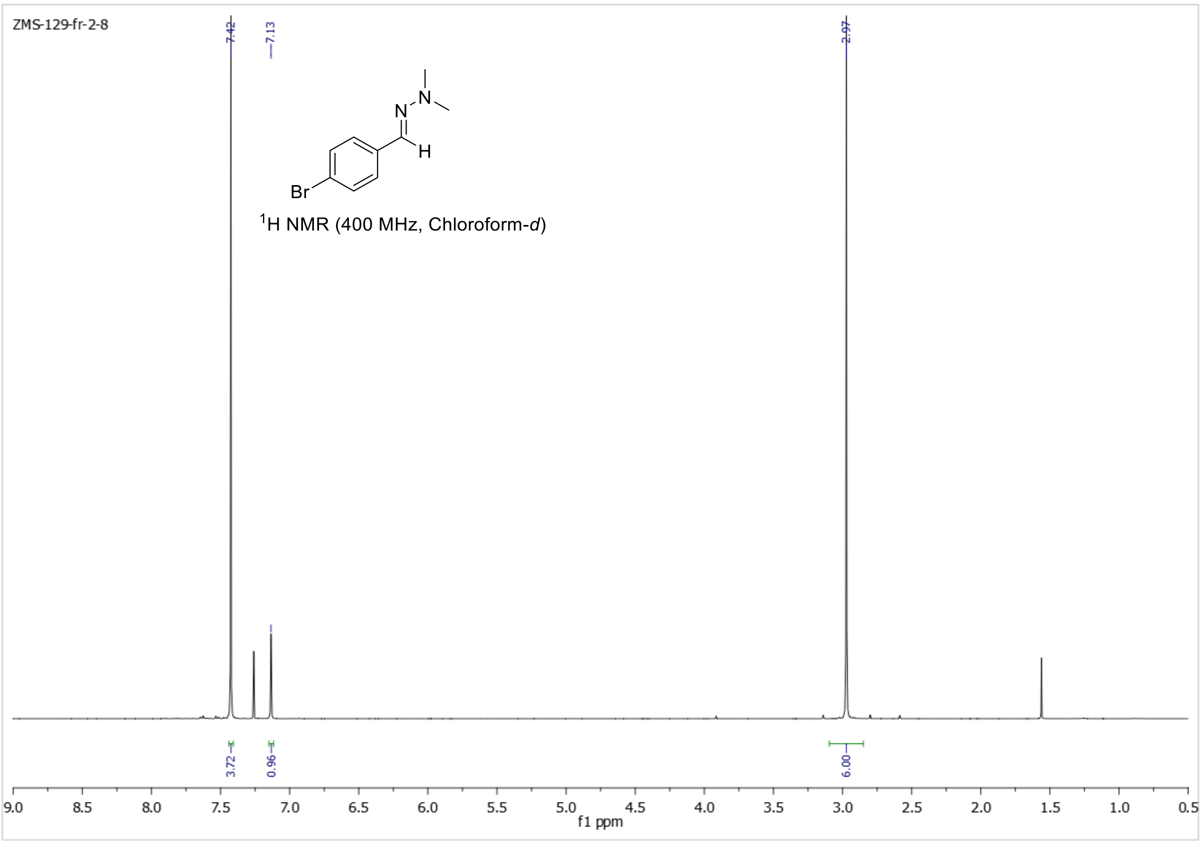

1,1-dimethyl-2-(4-nitrobenzylidene)hydrazine, ^{1}H NMR:

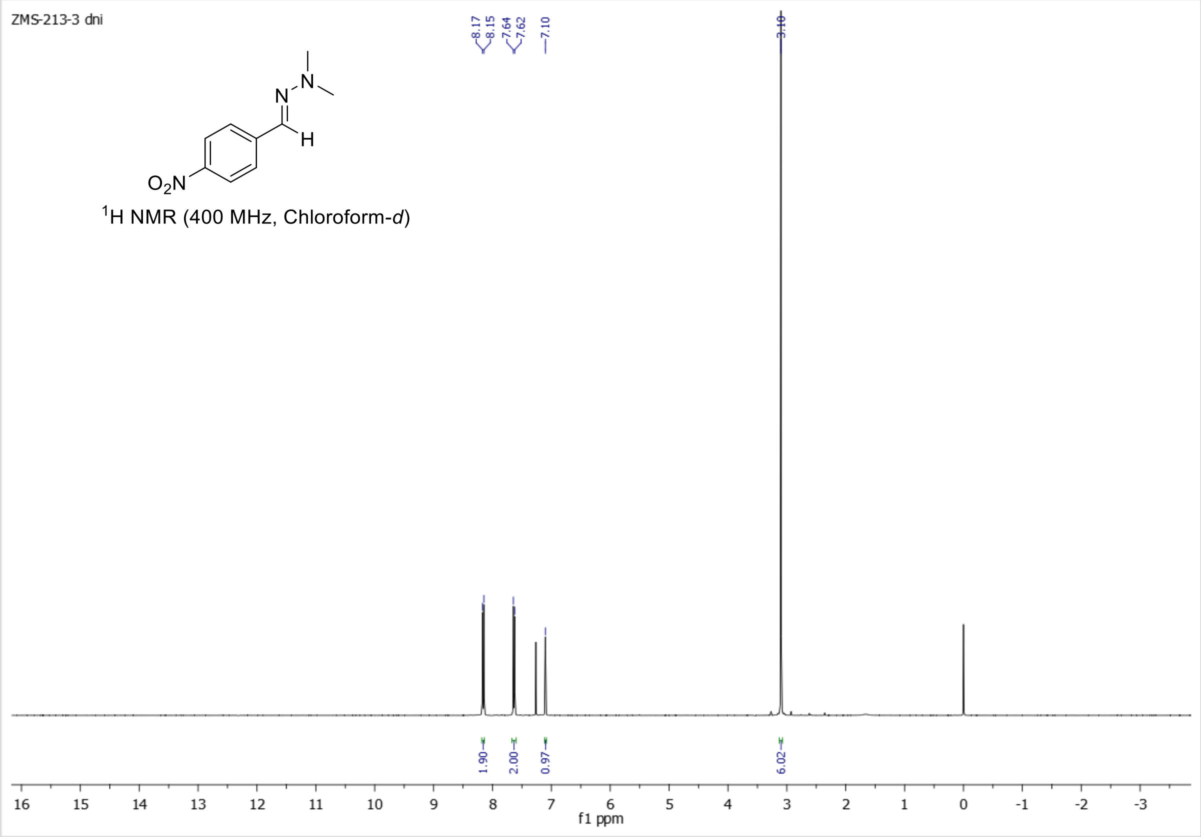

5-((2,2-dimethylhydrazineylidene)methyl)-2-nitrophenol, ^{1}H NMR:

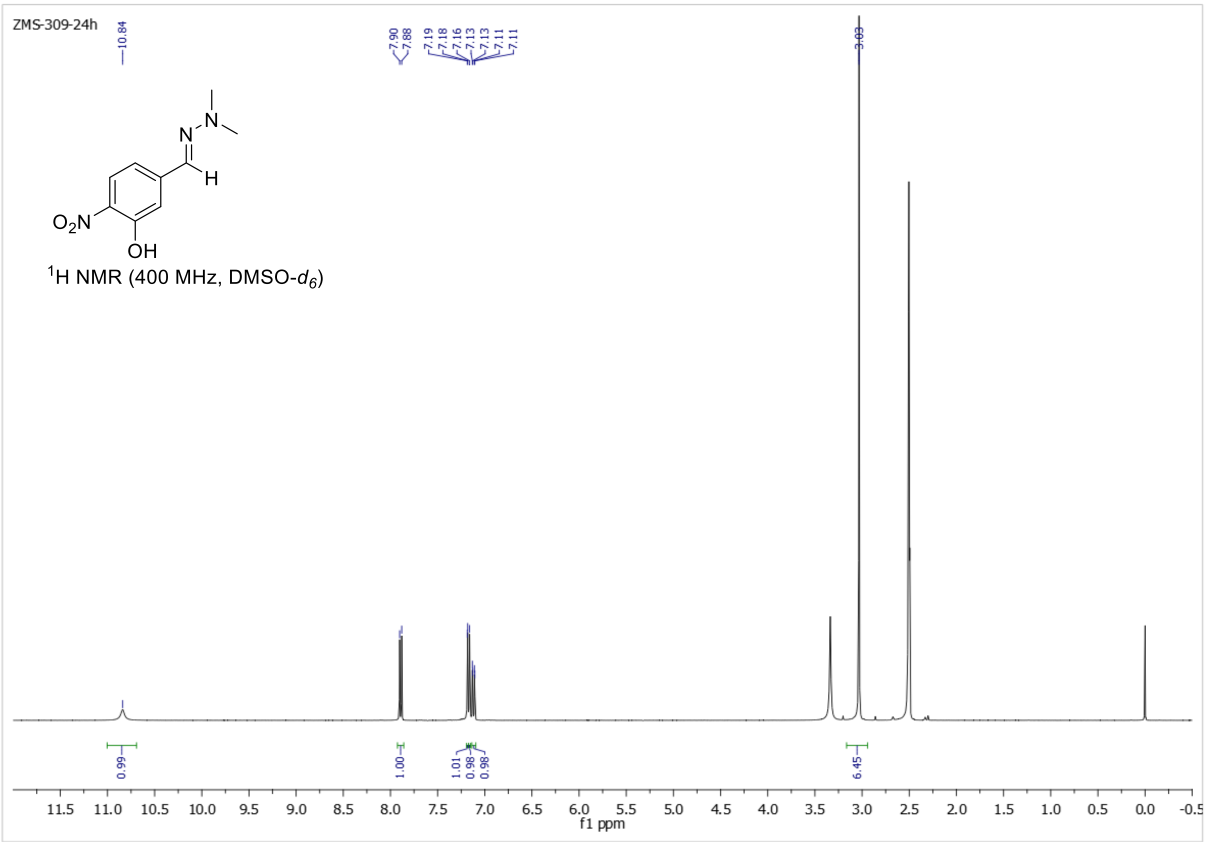

3-((2,2-dimethylhydrazineylidene)methyl)phenol, ^{1}H NMR:

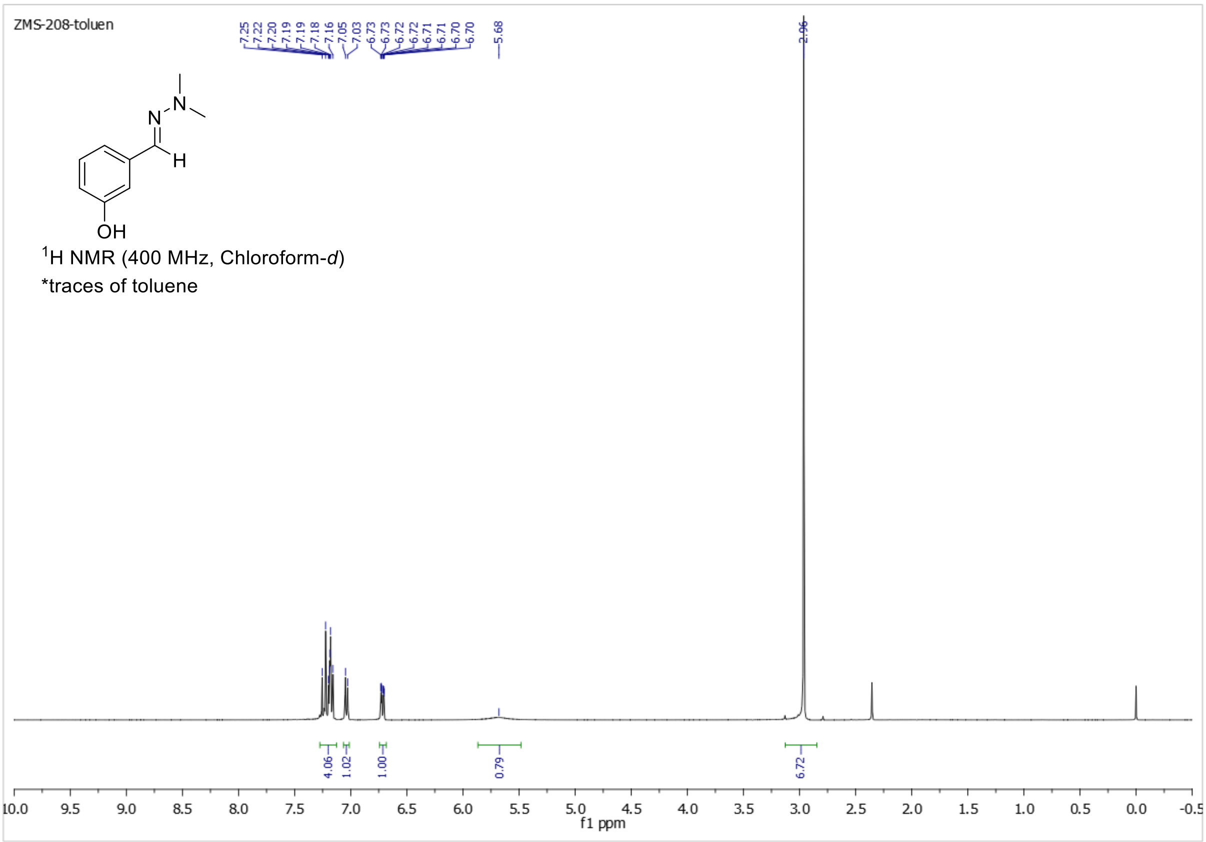

2-((2,2-dimethylhydrazineylidene)methyl)phenol, ^{1}H NMR:

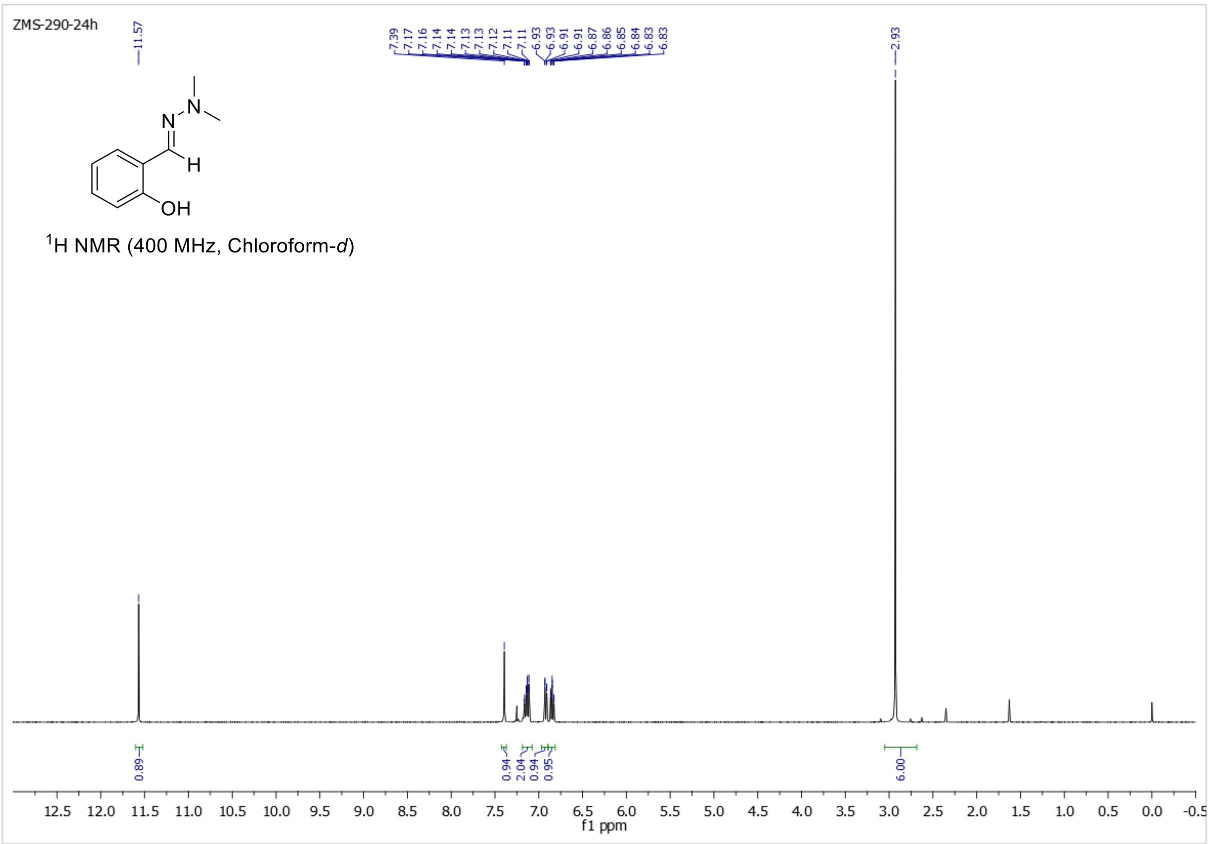

2-(4-methoxybenzylidene)-1,1-dimethylhydrazine, ^{1}H NMR:

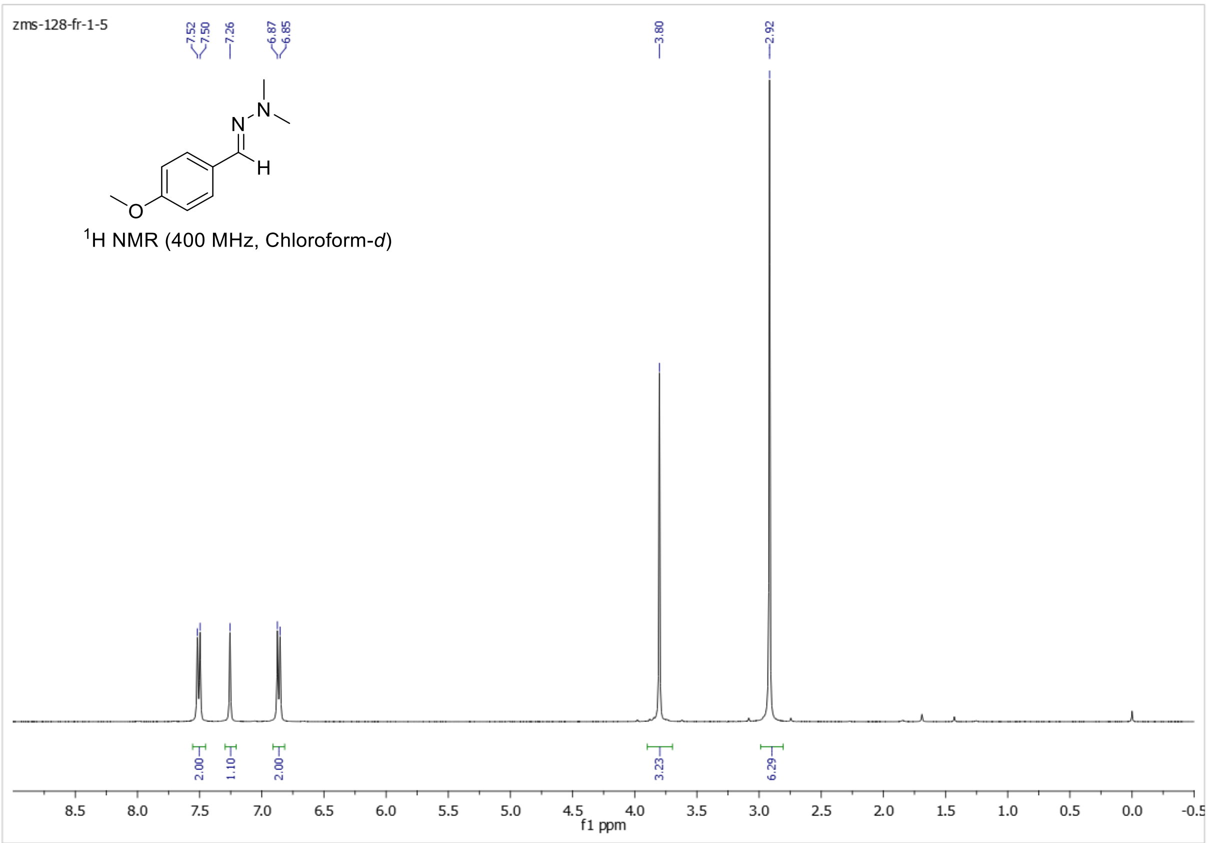

2-(4-(diethoxymethyl)benzylidene)-1,1-dimethylhydrazine, ^{1}H NMR:

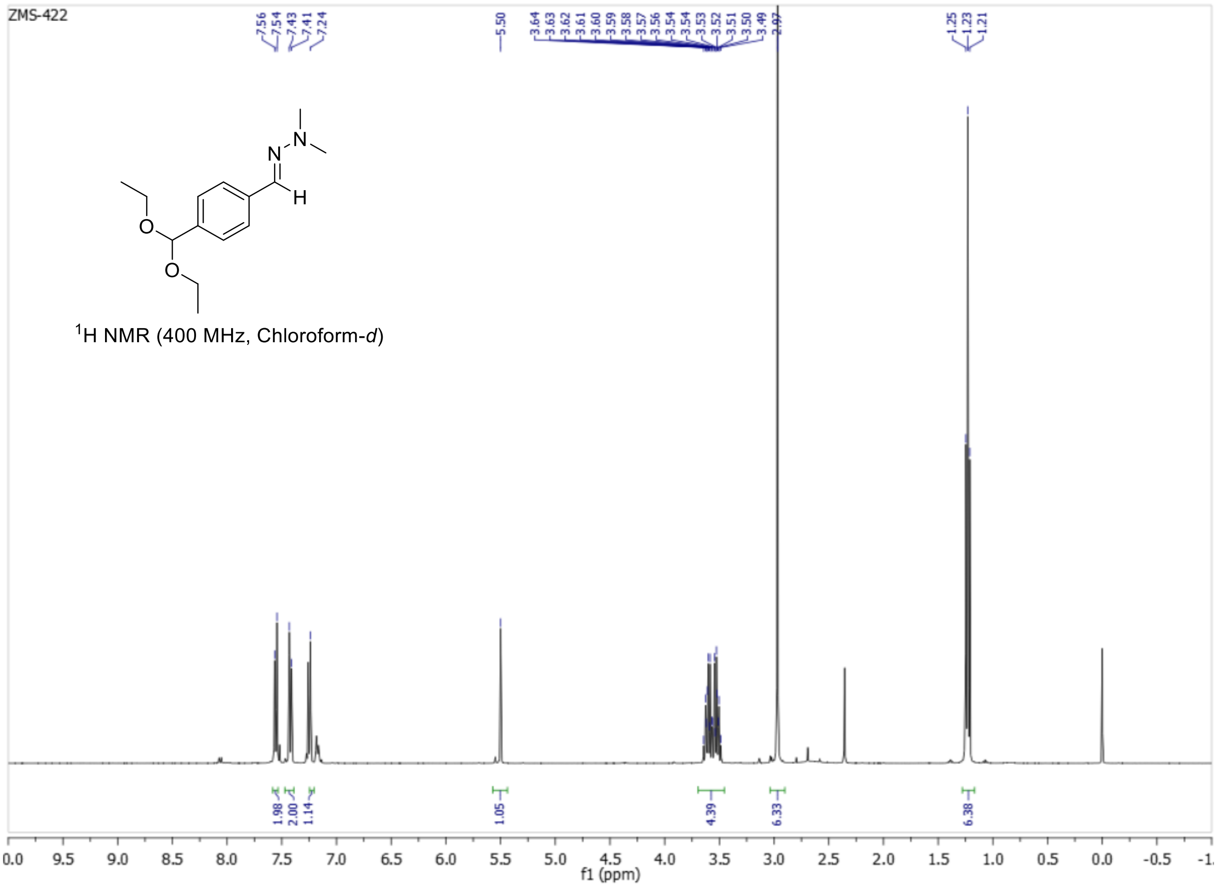

6-((2,2-dimethylhydrazineylidene)methyl)benzo[*d*]thiazol-2-amine, ^{1}H NMR:

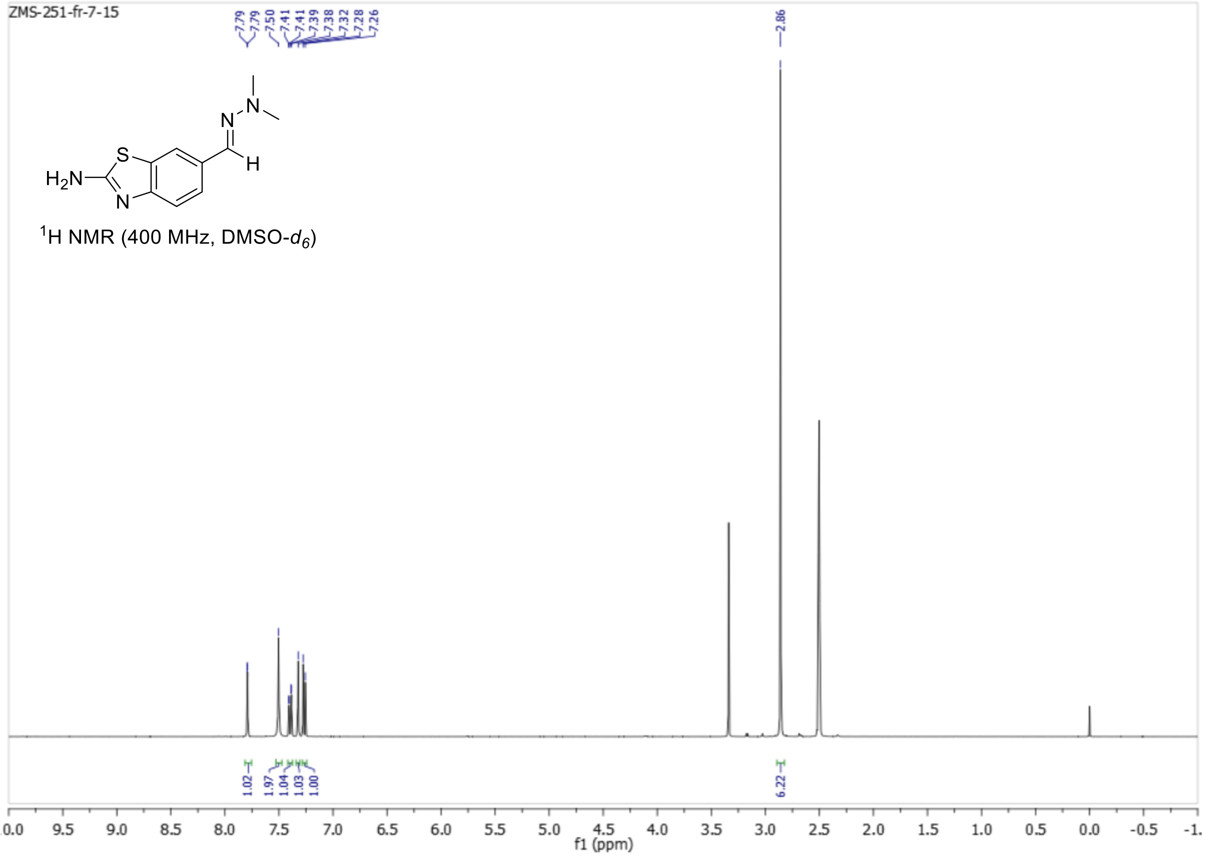

3-(4-bromophenyl)-3-(2,2-dimethylhydrazineylidene)-1,1,1-trifluoropropan-2-one, ^{1}H NMR:

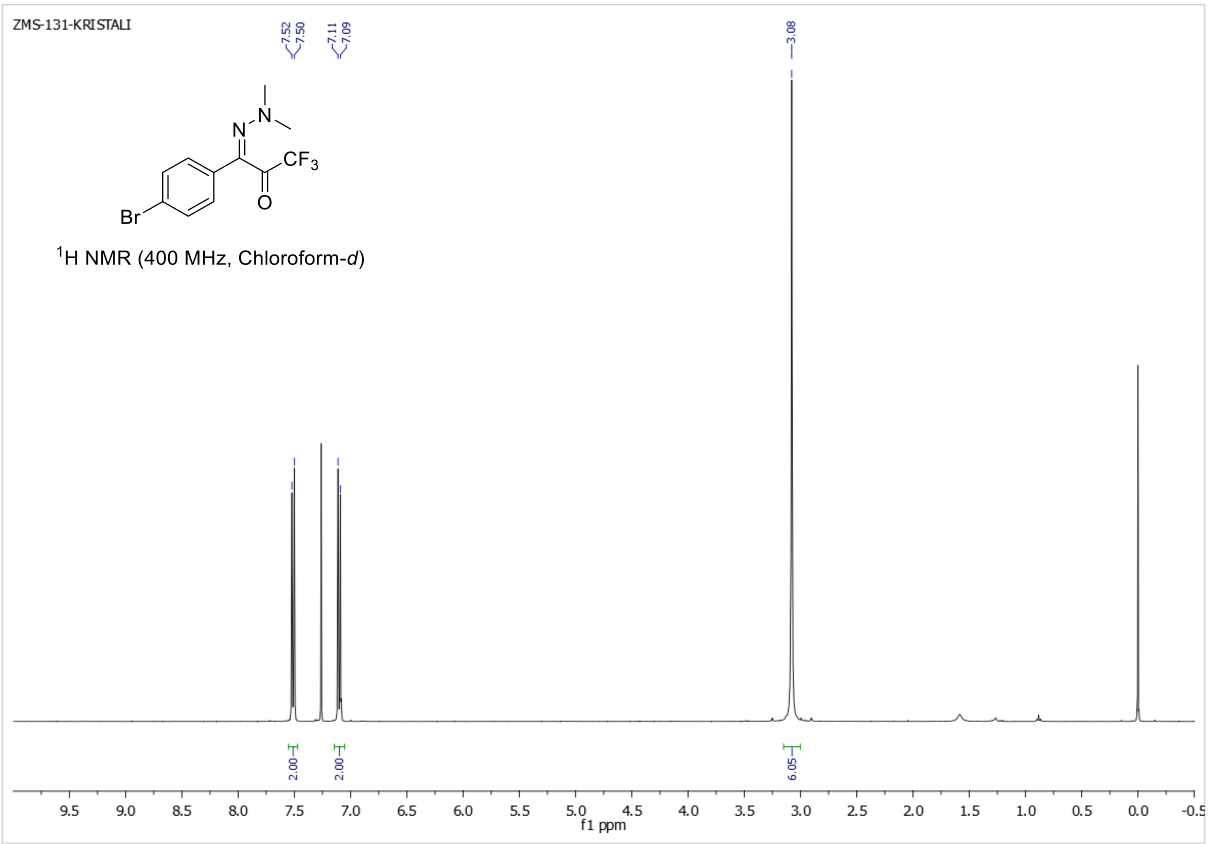

3-(4-bromophenyl)-3-(2,2-dimethylhydrazineylidene)-1,1,1-trifluoropropan-2-one, ^{19}F NMR:

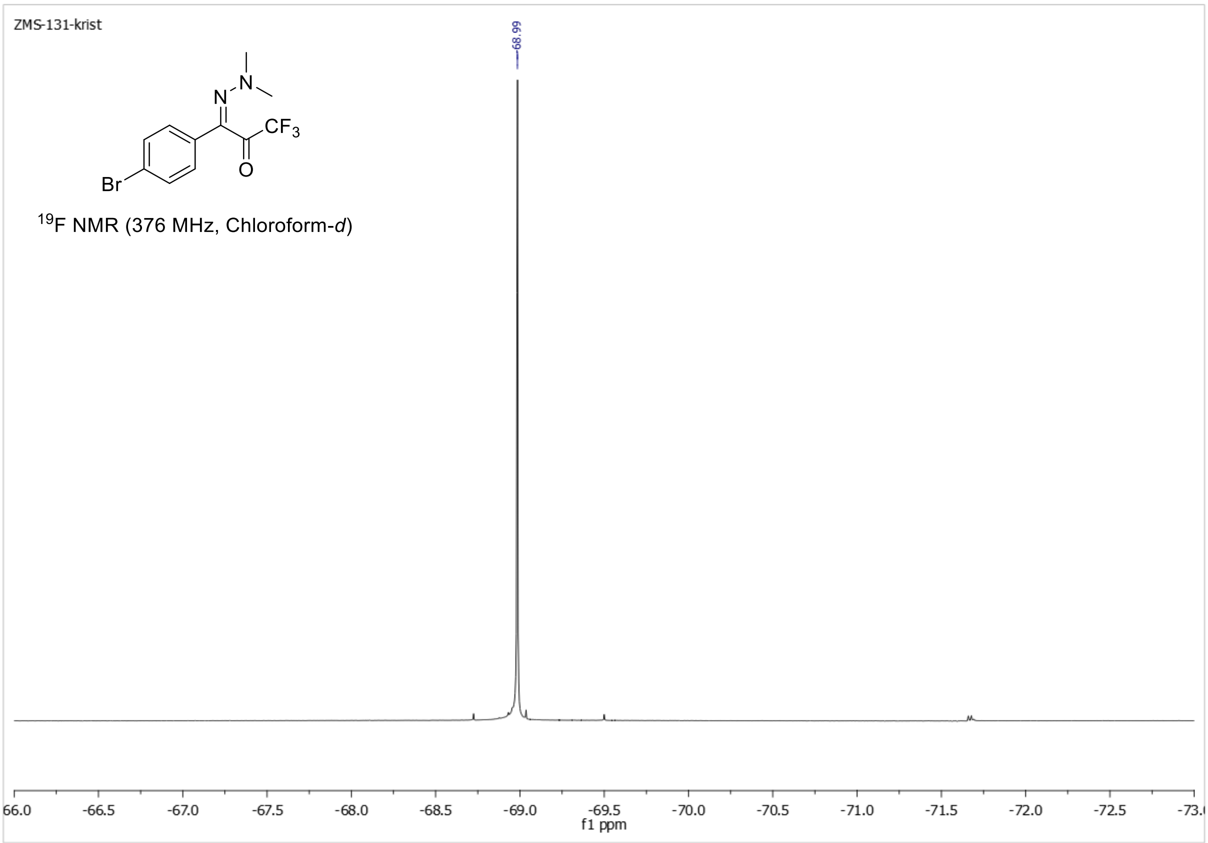

3-(2,2-dimethylhydrazineylidene)-1,1,1-trifluoro-3-(4-nitrophenyl)propan-2-one, ^{1}H NMR:

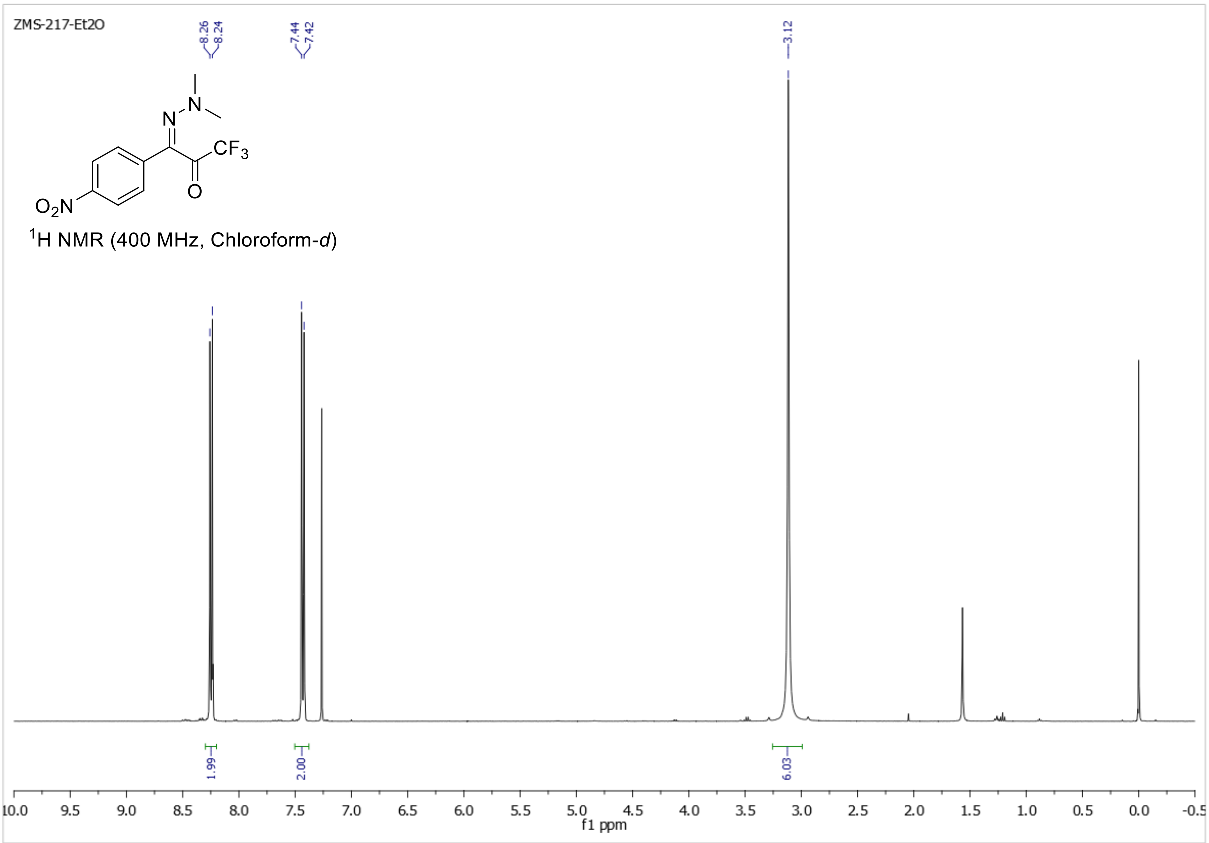

3-(2,2-dimethylhydrazineylidene)-1,1,1-trifluoro-3-(4-nitrophenyl)propan-2-one, ^{19}F NMR:

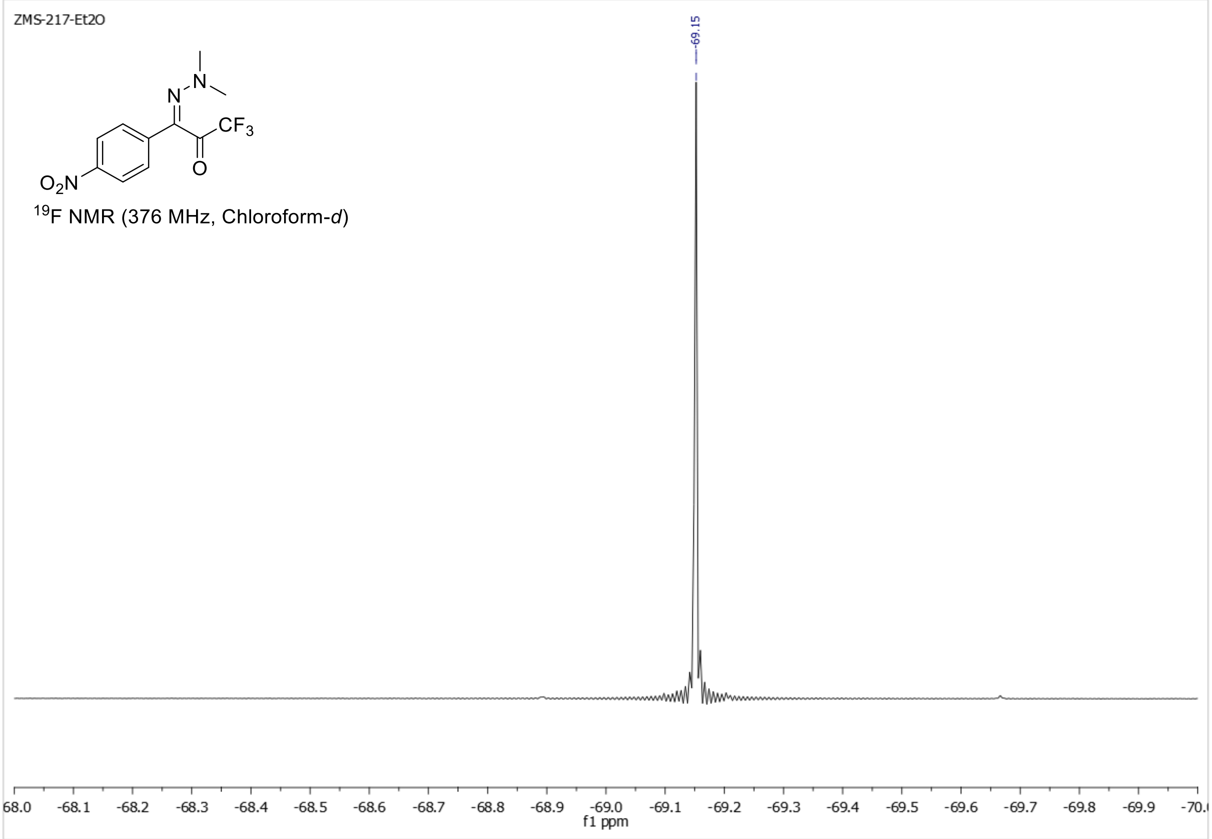

3-(2,2-dimethylhydrazineylidene)-1,1,1-trifluoro-3-(3-hydroxy-4-nitrophenyl)propan-2-one, ^{1}H NMR:

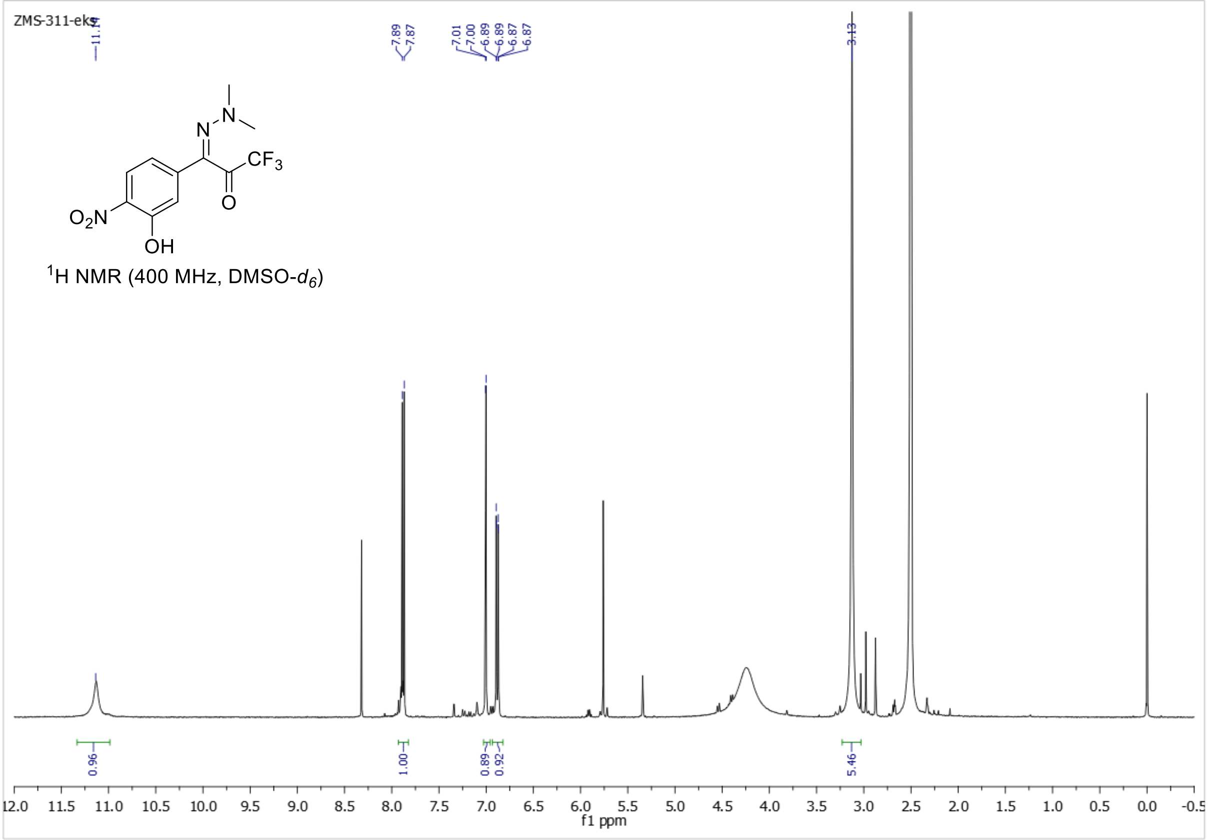

3-(2,2-dimethylhydrazineylidene)-1,1,1-trifluoro-3-(3-hydroxy-4-nitrophenyl)propan-2-one, ^{19}F NMR:

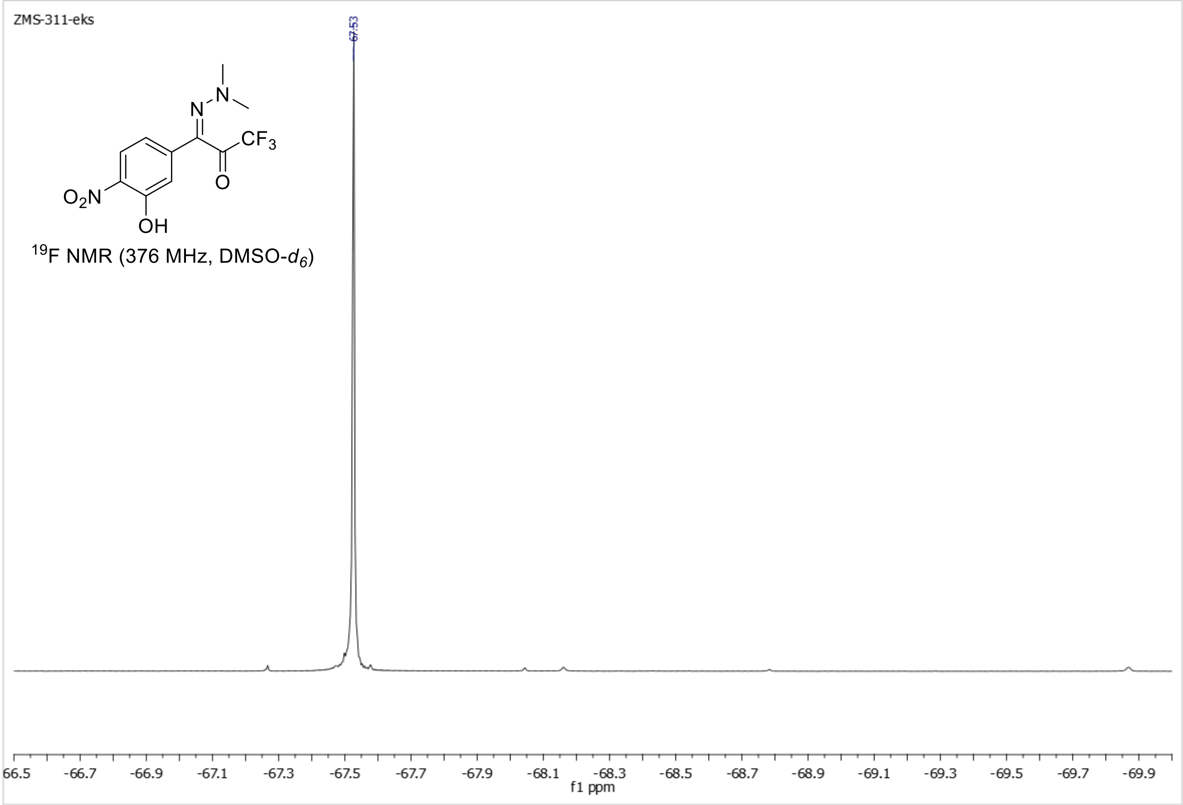

3-(2,2-dimethylhydrazineylidene)-1,1,1-trifluoro-3-(3-hydroxyphenyl)propan-2-one, ^{1}H NMR:

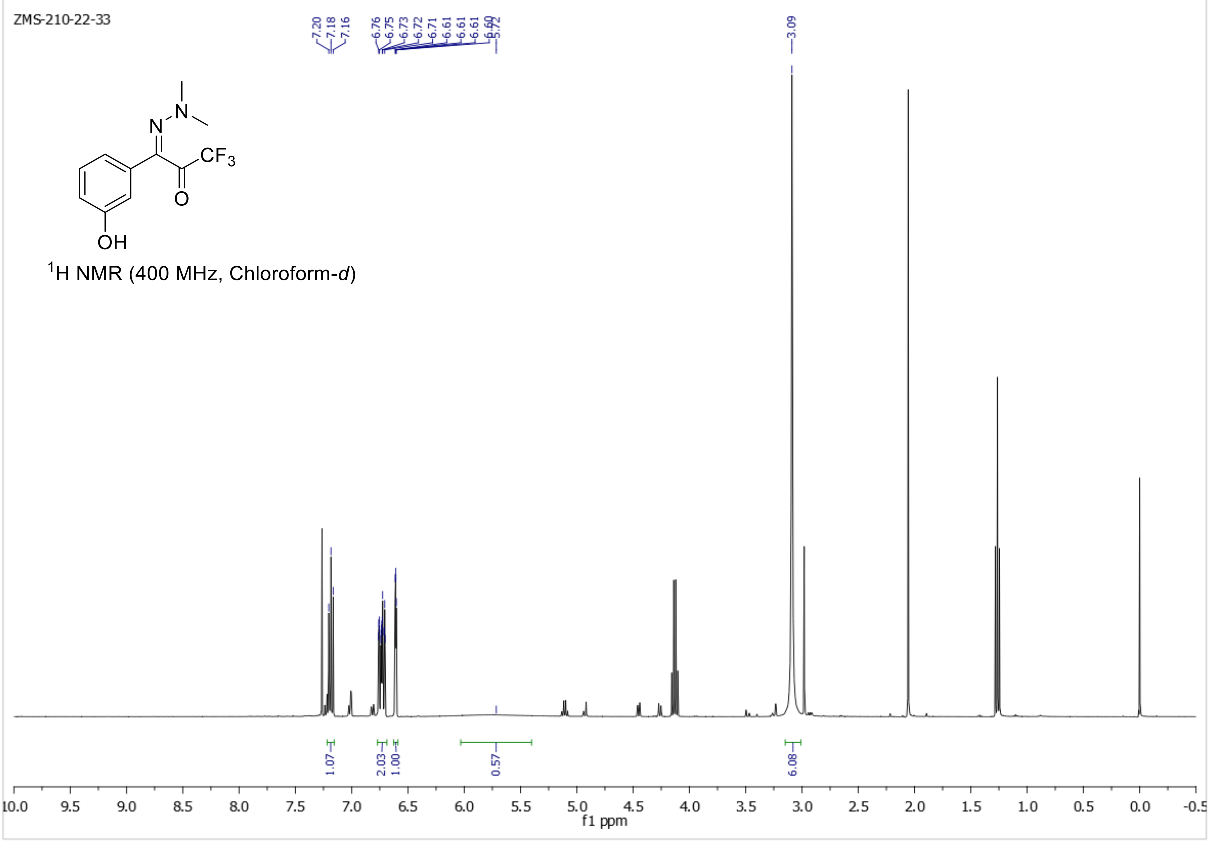

3-(2,2-dimethylhydrazineylidene)-1,1,1-trifluoro-3-(3-hydroxyphenyl)propan-2-one, ^{19}F NMR:

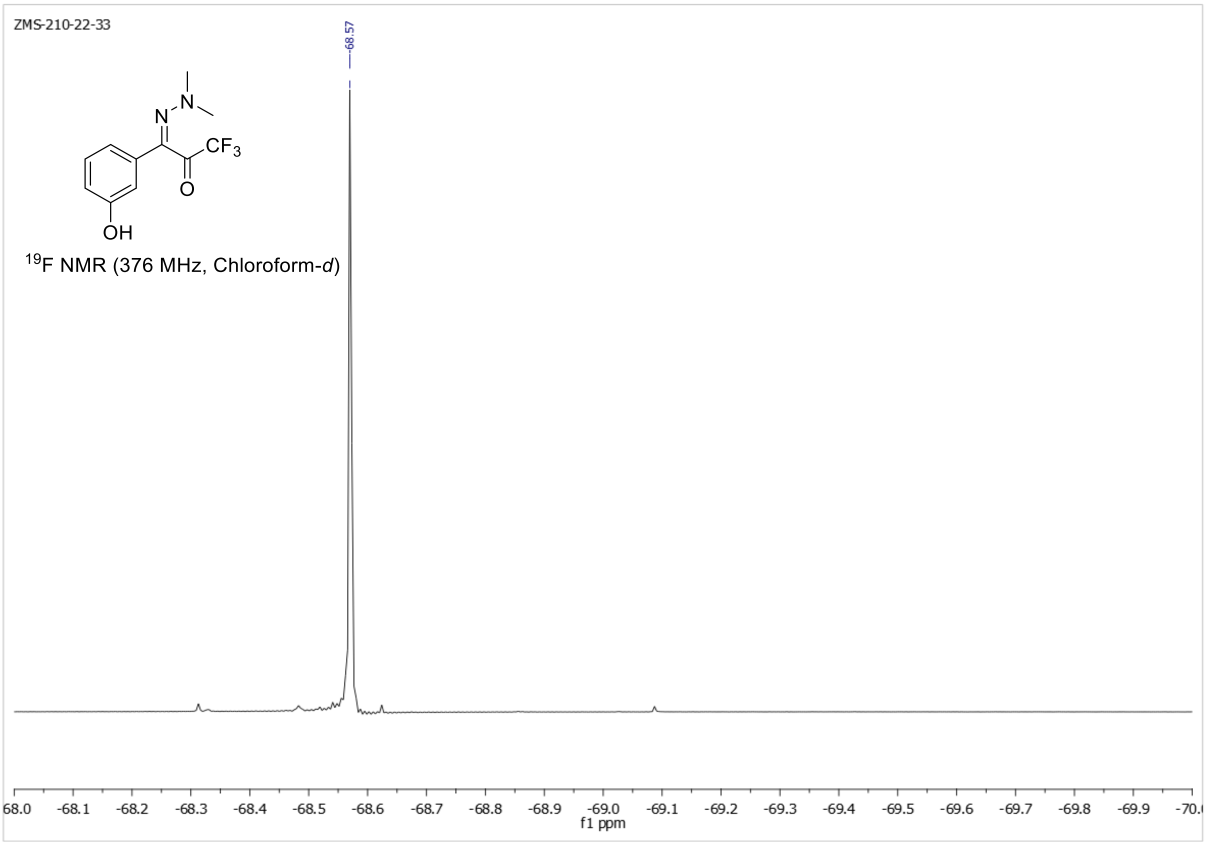

3-(2,2-dimethylhydrazineylidene)-1,1,1-trifluoro-3-(4-methoxyphenyl)propan-2-one, ^{1}H NMR:

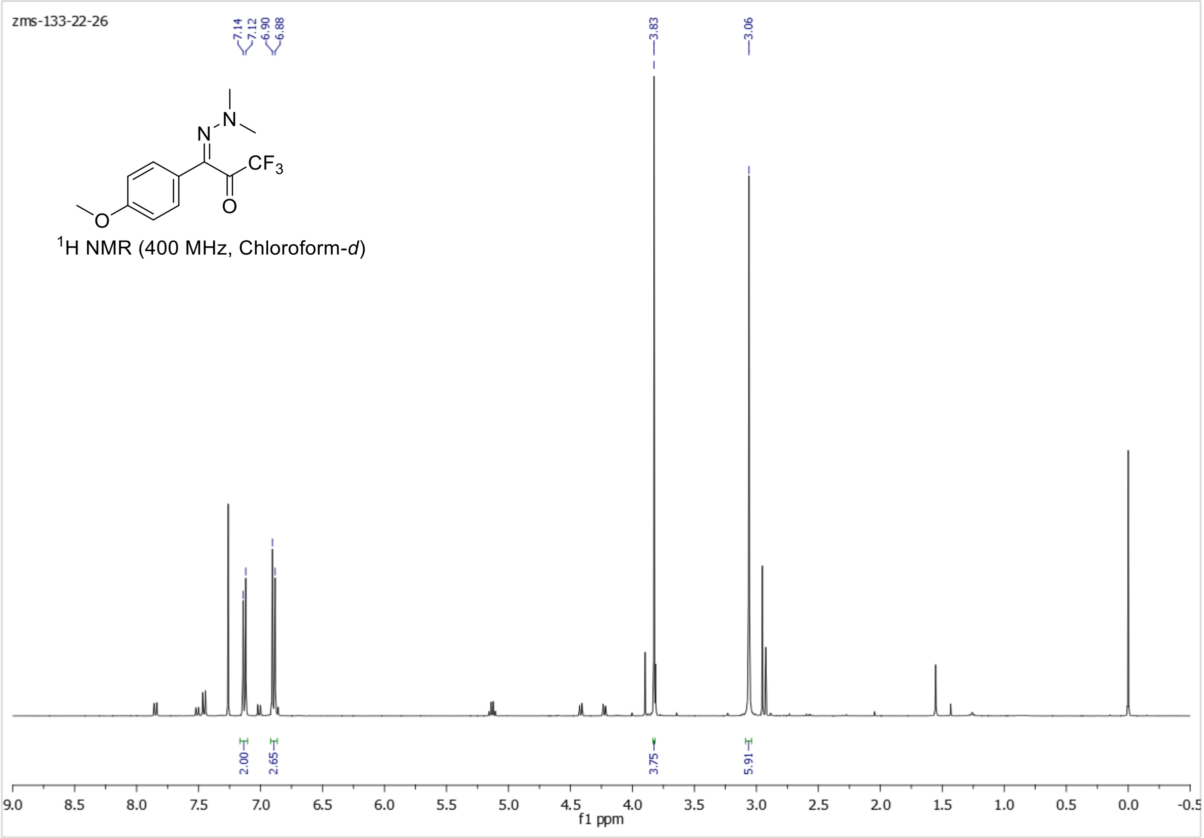

3-(2,2-dimethylhydrazineylidene)-1,1,1-trifluoro-3-(4-methoxyphenyl)propan-2-one, ^{19}F NMR:

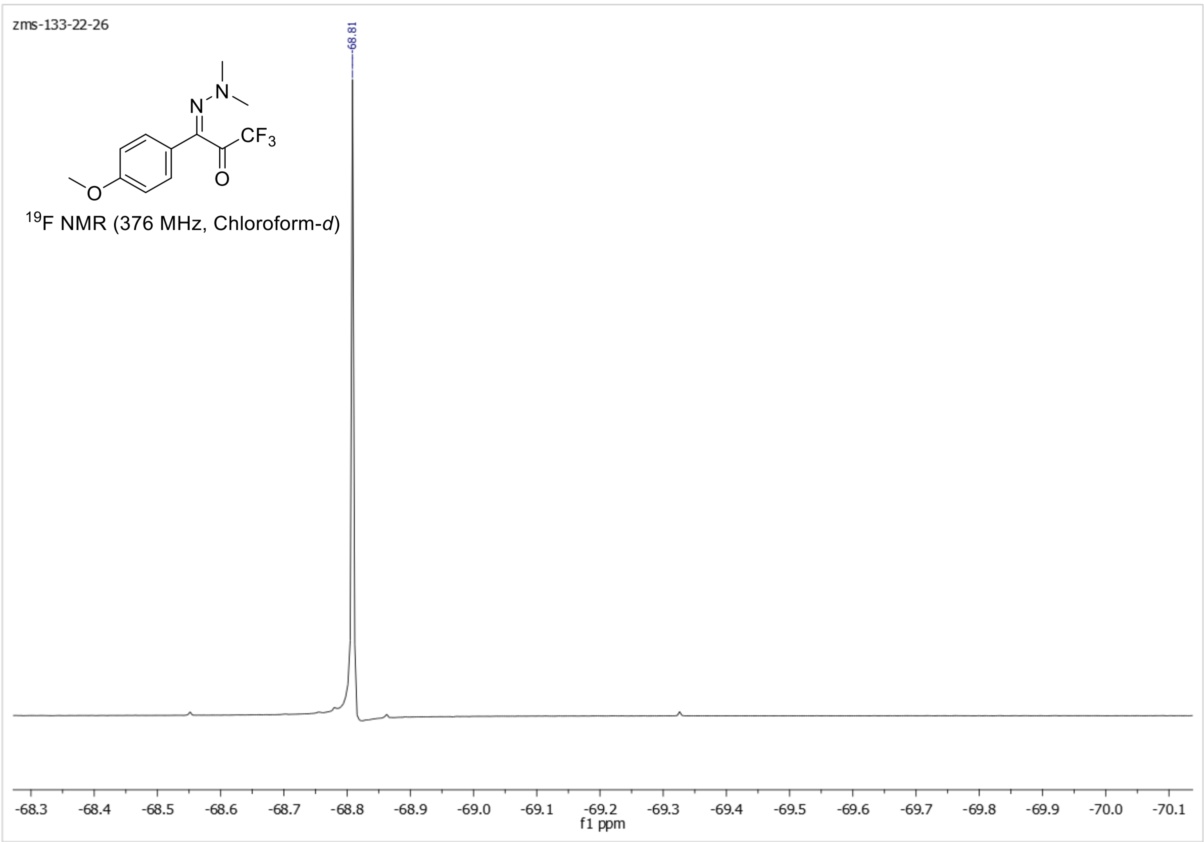

N-(6-(1-(2,2-dimethylhydrazineylidene)-3,3,3-trifluoro-2-oxopropyl)benzo[*d*]thiazol-2-yl)-2,2,2-trifluoroacetamide, ^{1}H NMR:

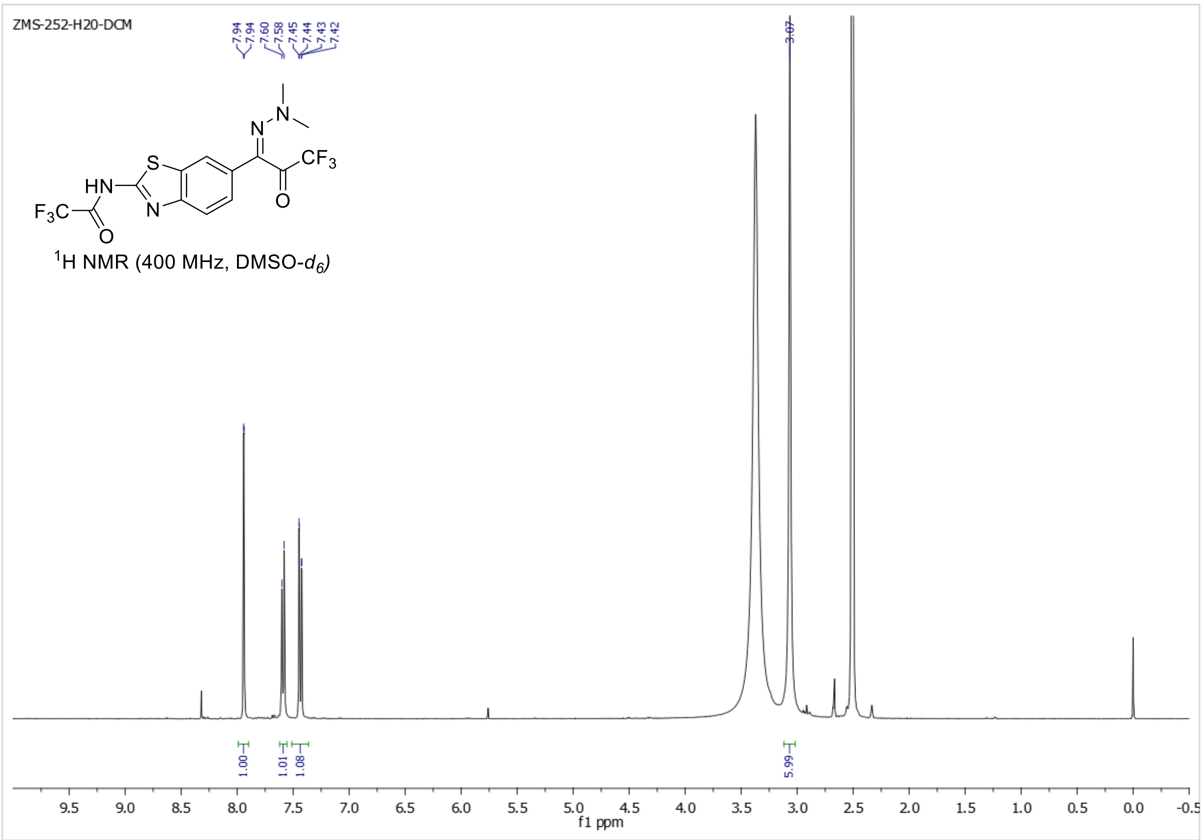

N-(6-(1-(2,2-dimethylhydrazineylidene)-3,3,3-trifluoro-2-oxopropyl)benzo[*d*]thiazol-2-yl)-2,2,2-trifluoroacetamide, ^{19}F NMR:

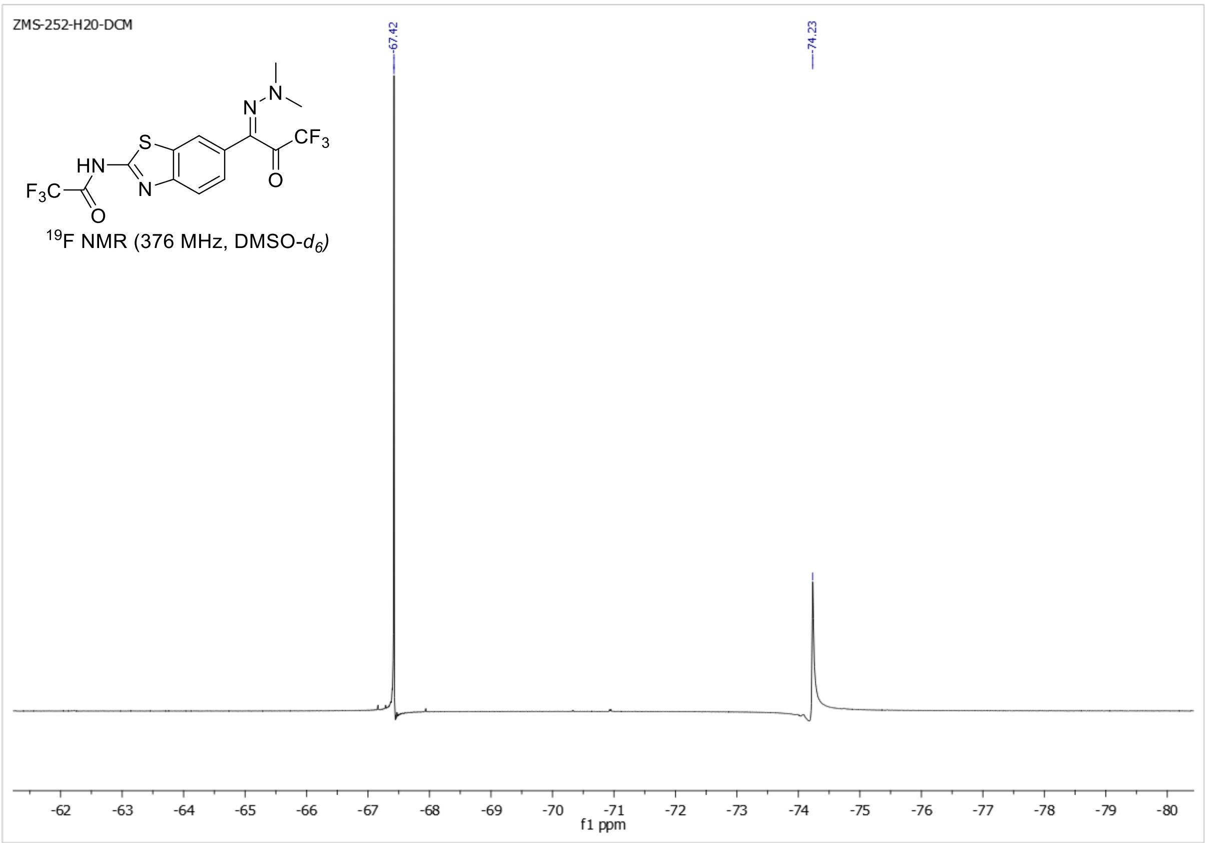

Compound **1a**, ^{1}H NMR:

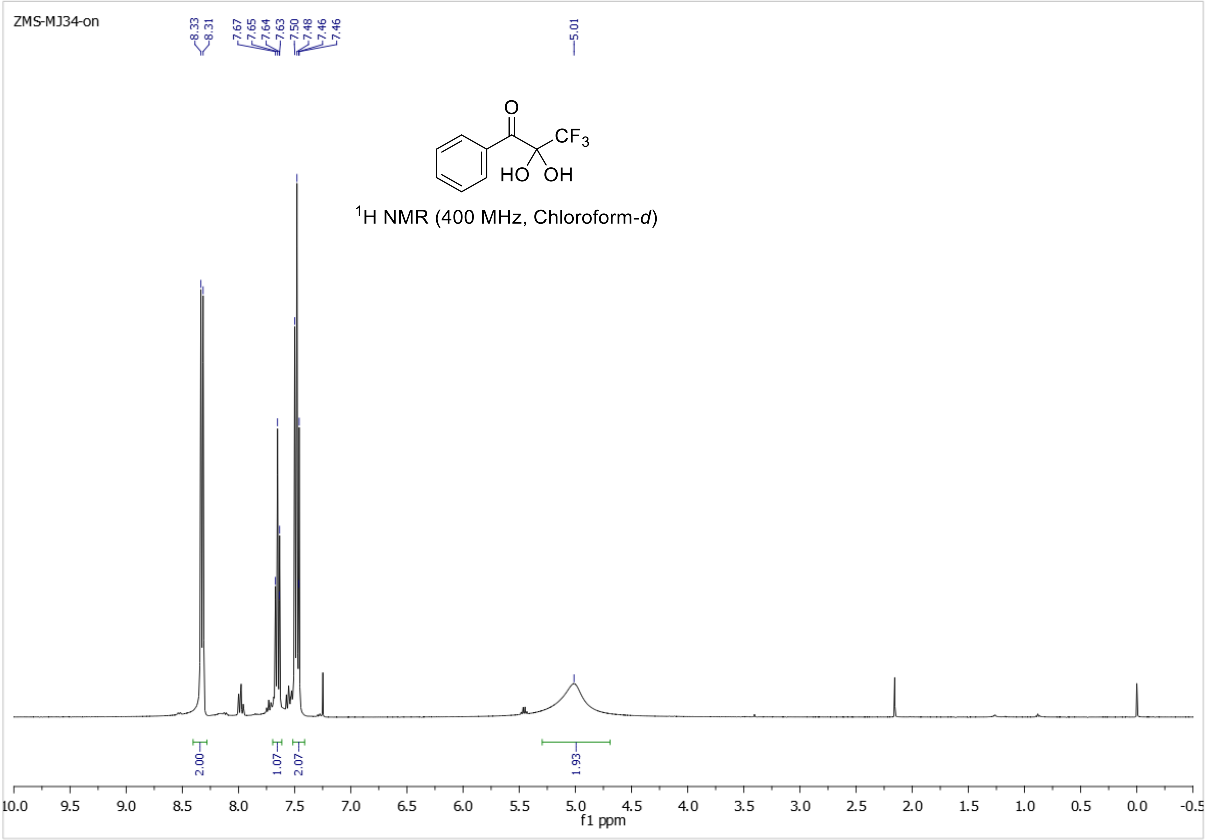

Compound **1a**, ^{19}F NMR:

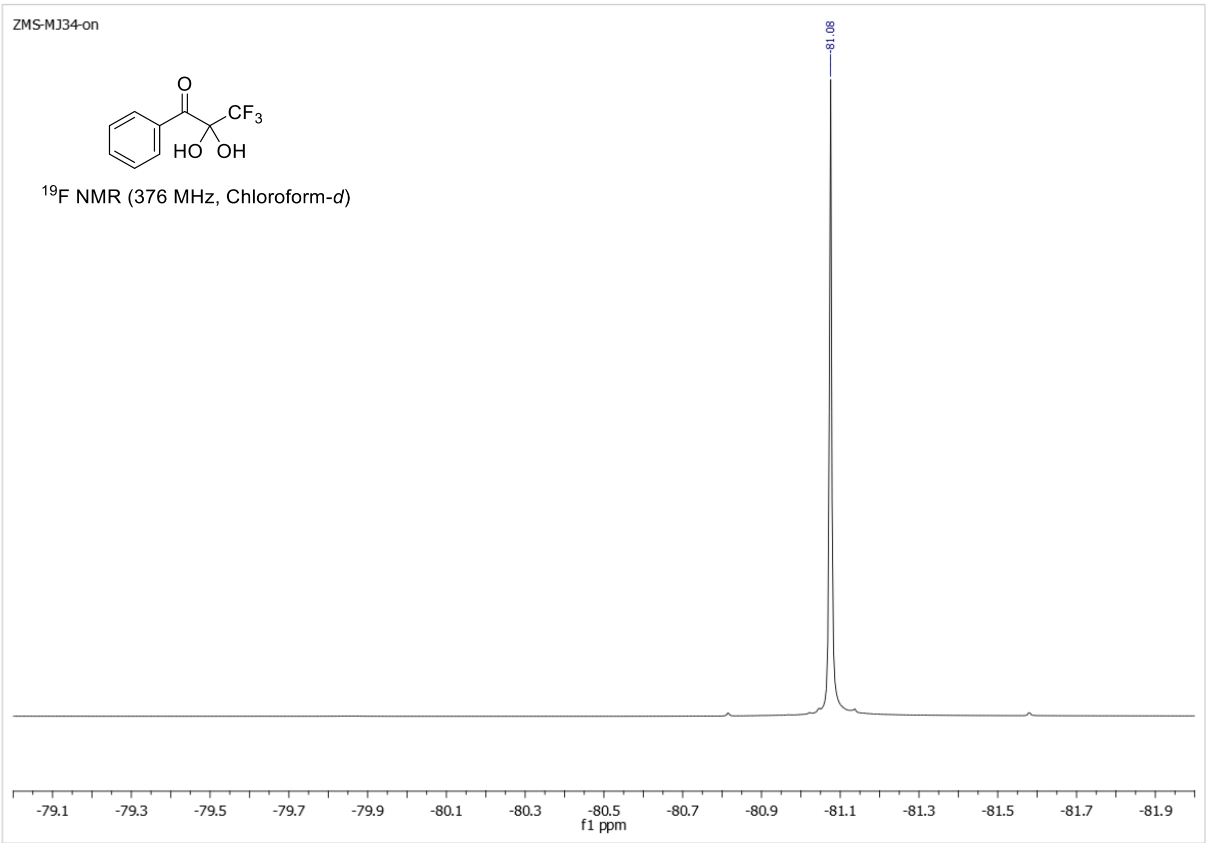

Compound **1a**, ^{13}C NMR:

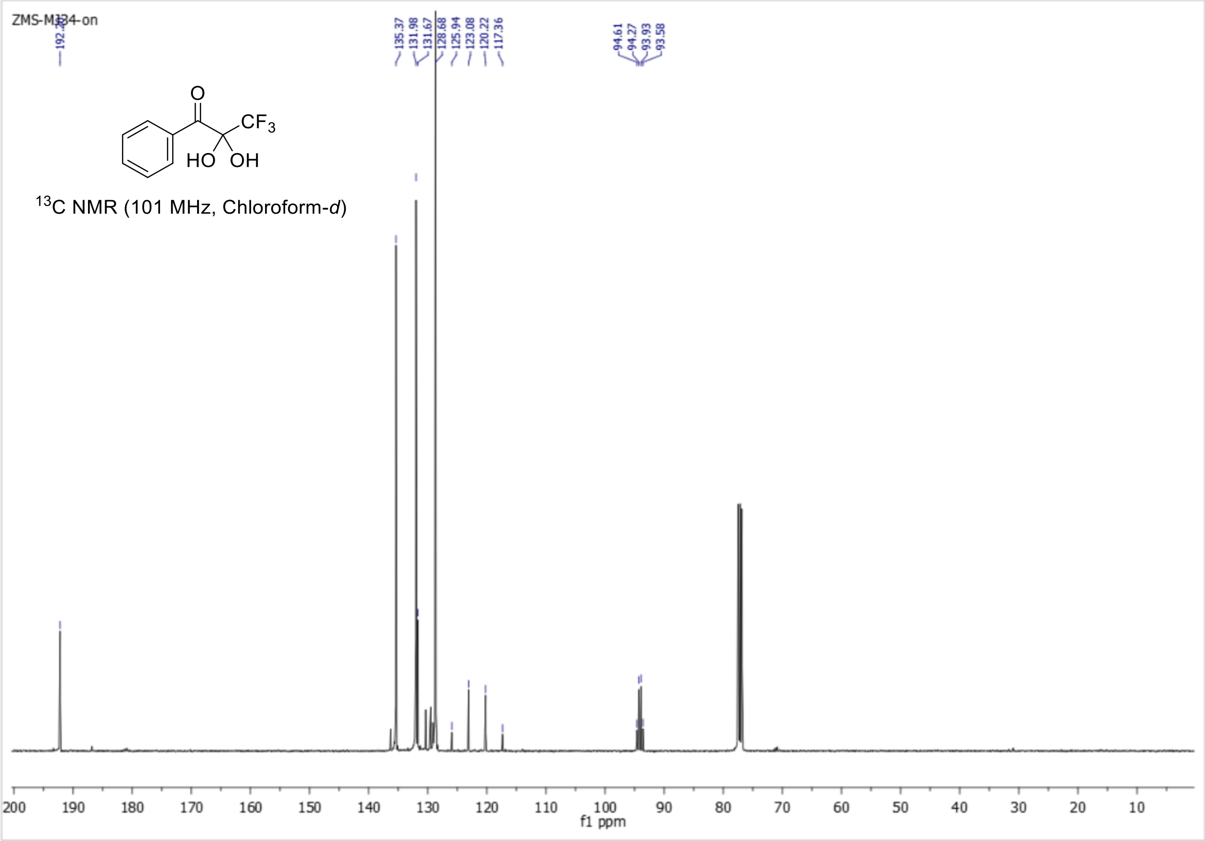

Compound **1b**, ^{1}H NMR:

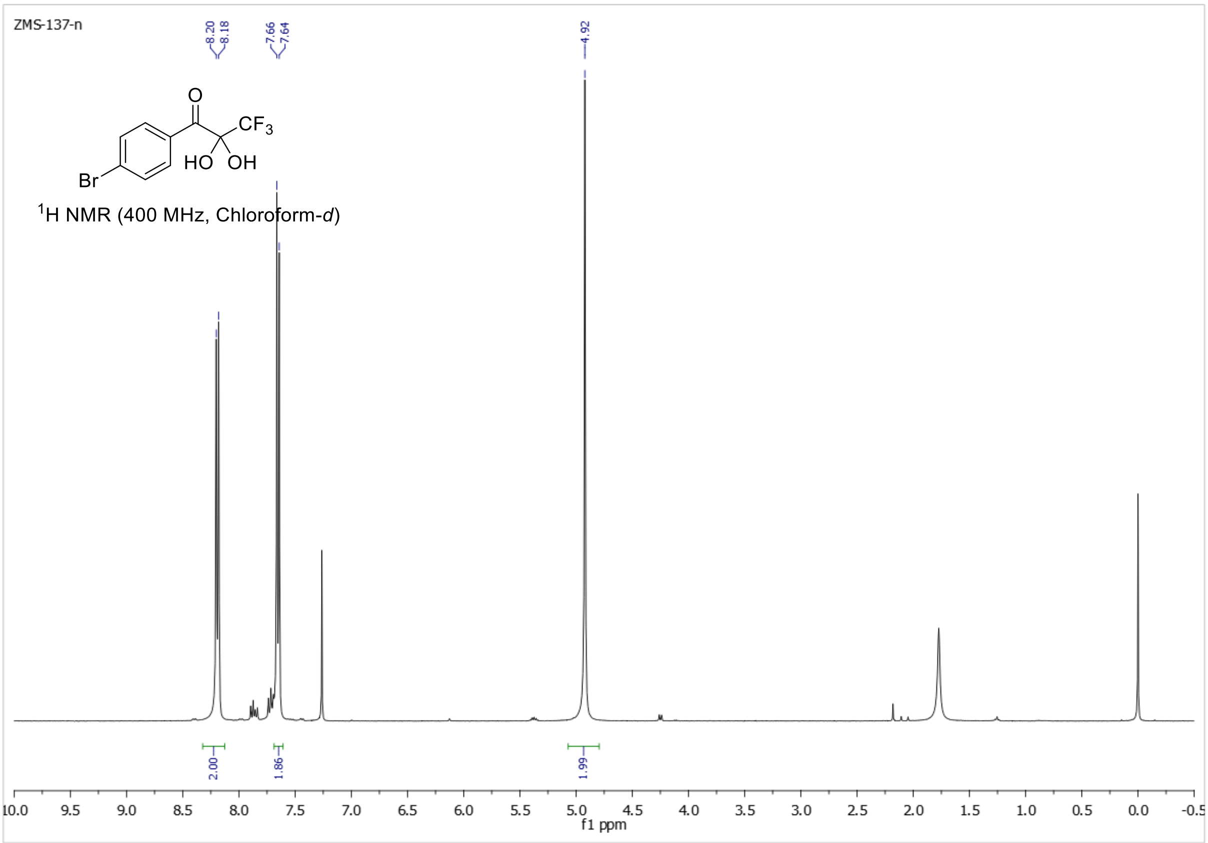

Compound **1b**, ^{19}F NMR:

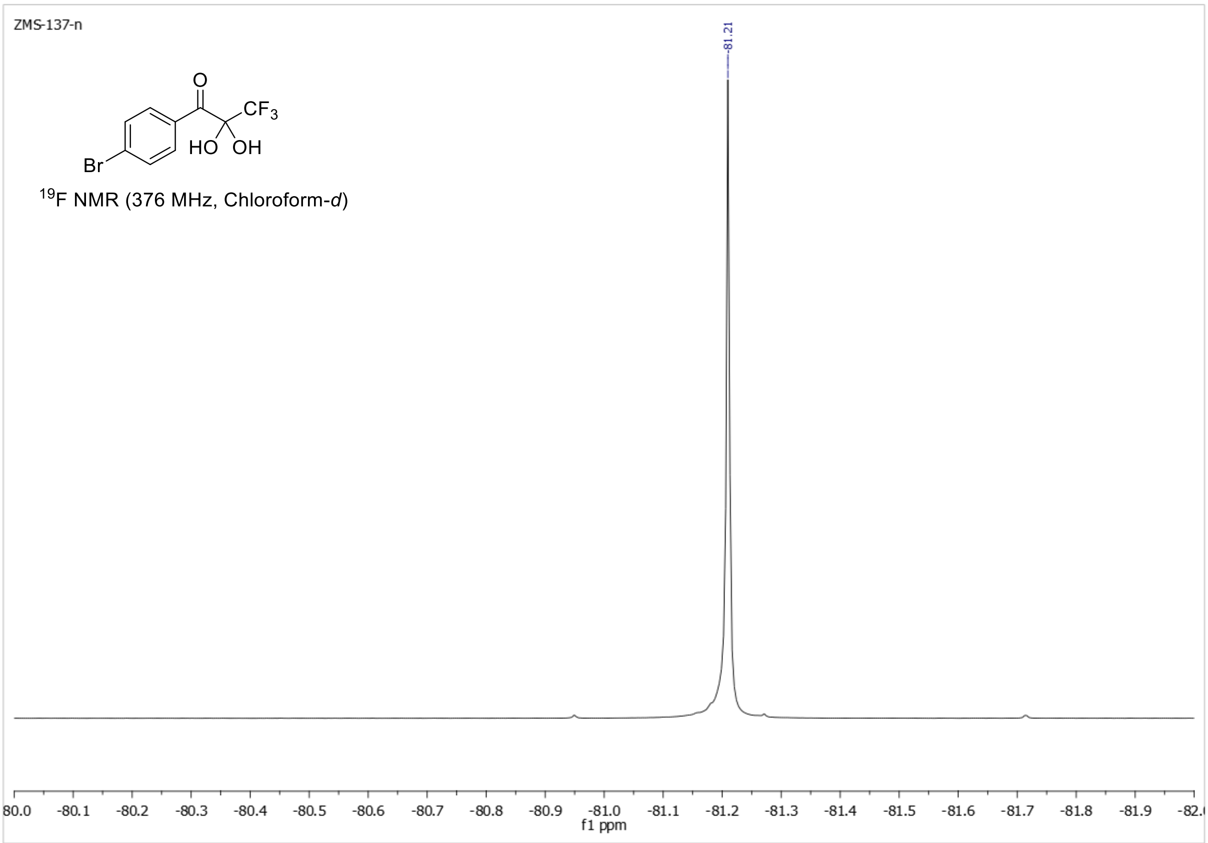

Compound **1b**, ^{13}C NMR:

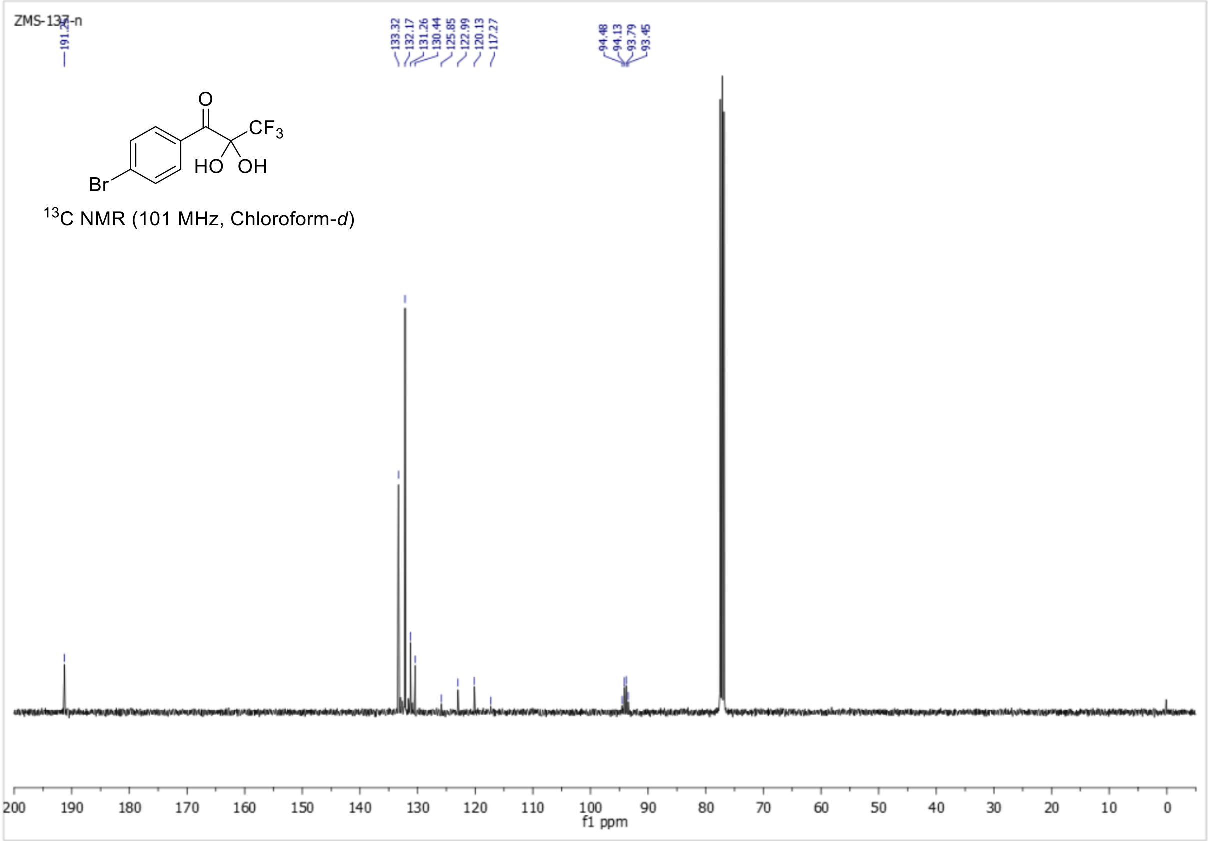

Compound **1e**, ^{1}H NMR:

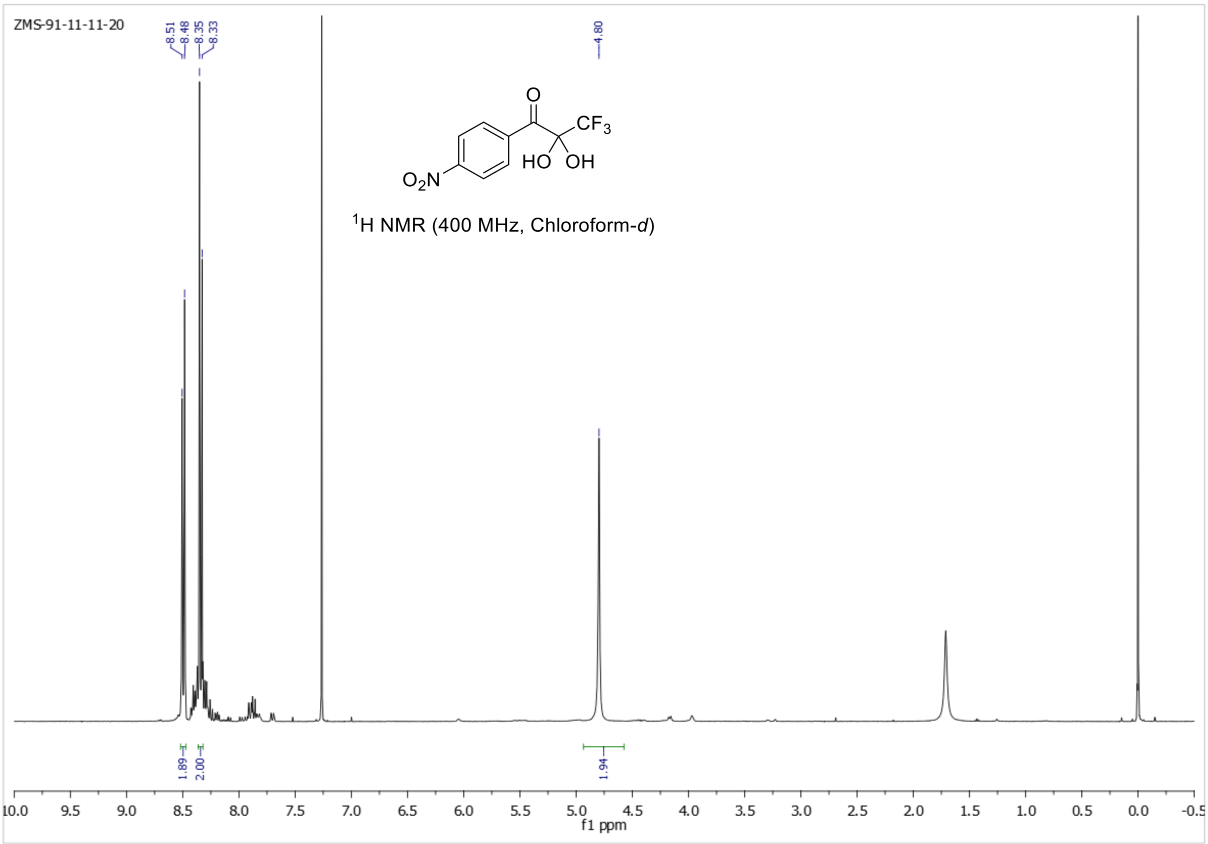

Compound **1e**, ^{19}F NMR:

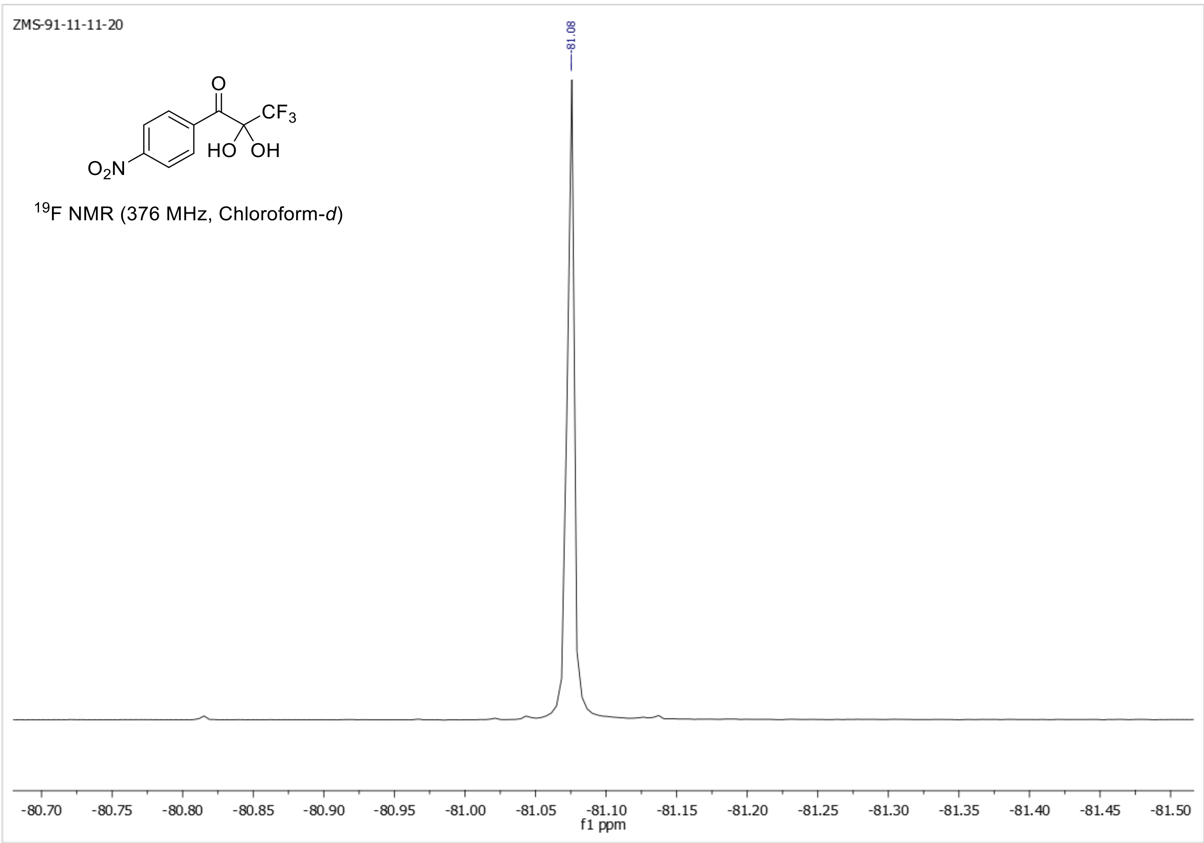

Compound **1e**, ^{13}C NMR:

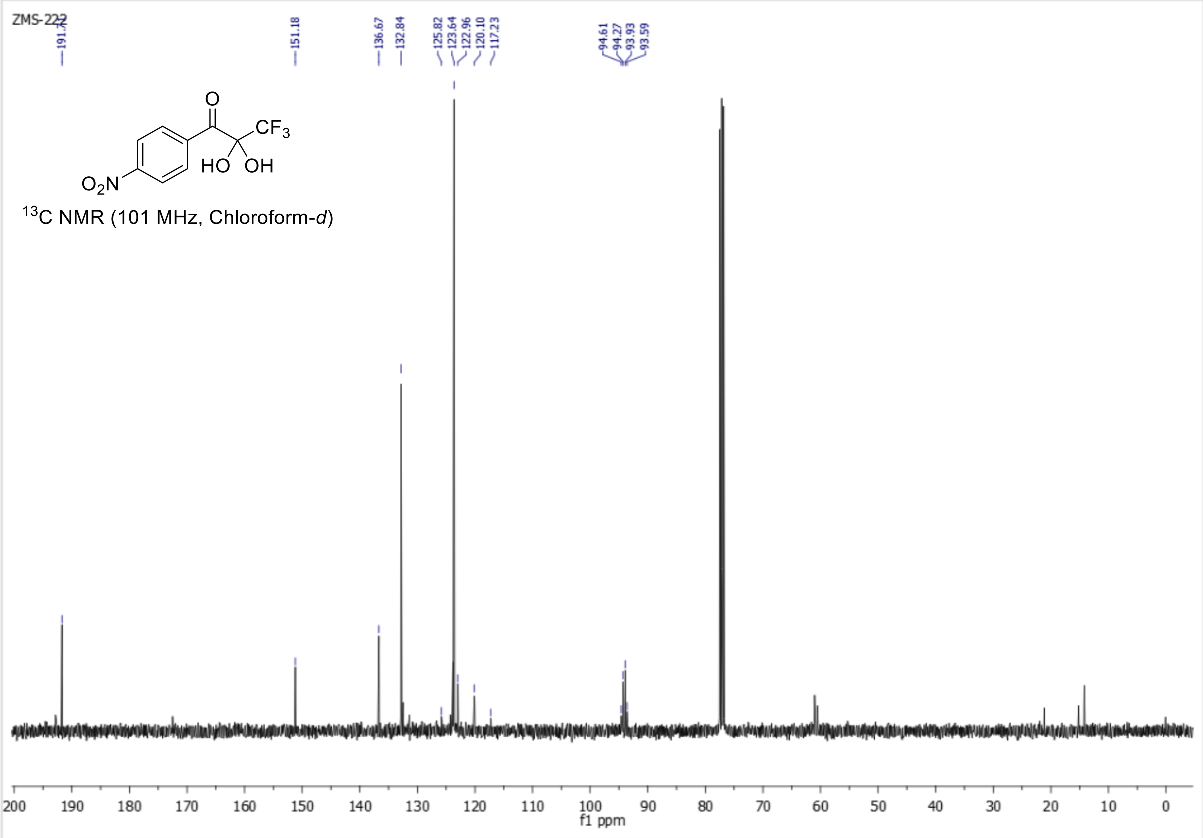

Compound **1f**, ^{1}H NMR:

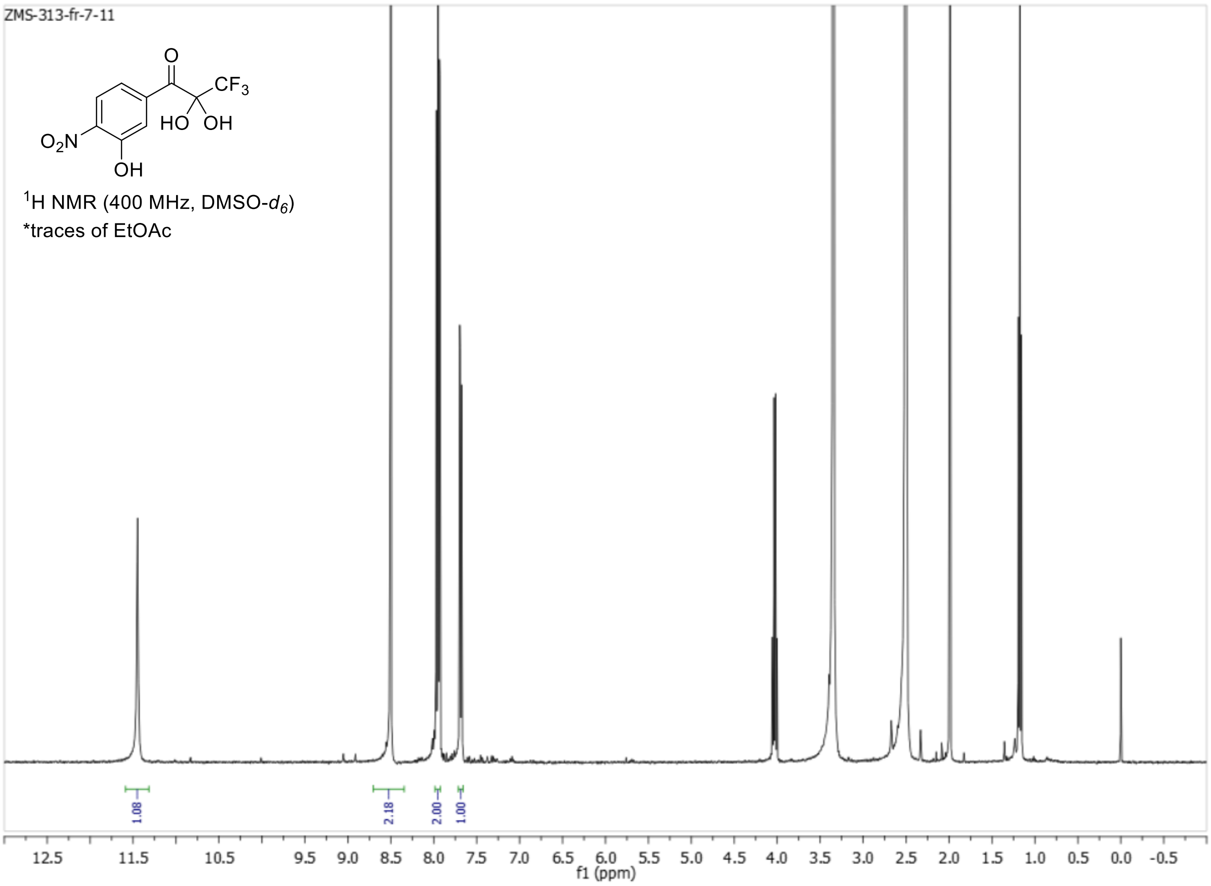

Compound **1f**, ^{19}F NMR:

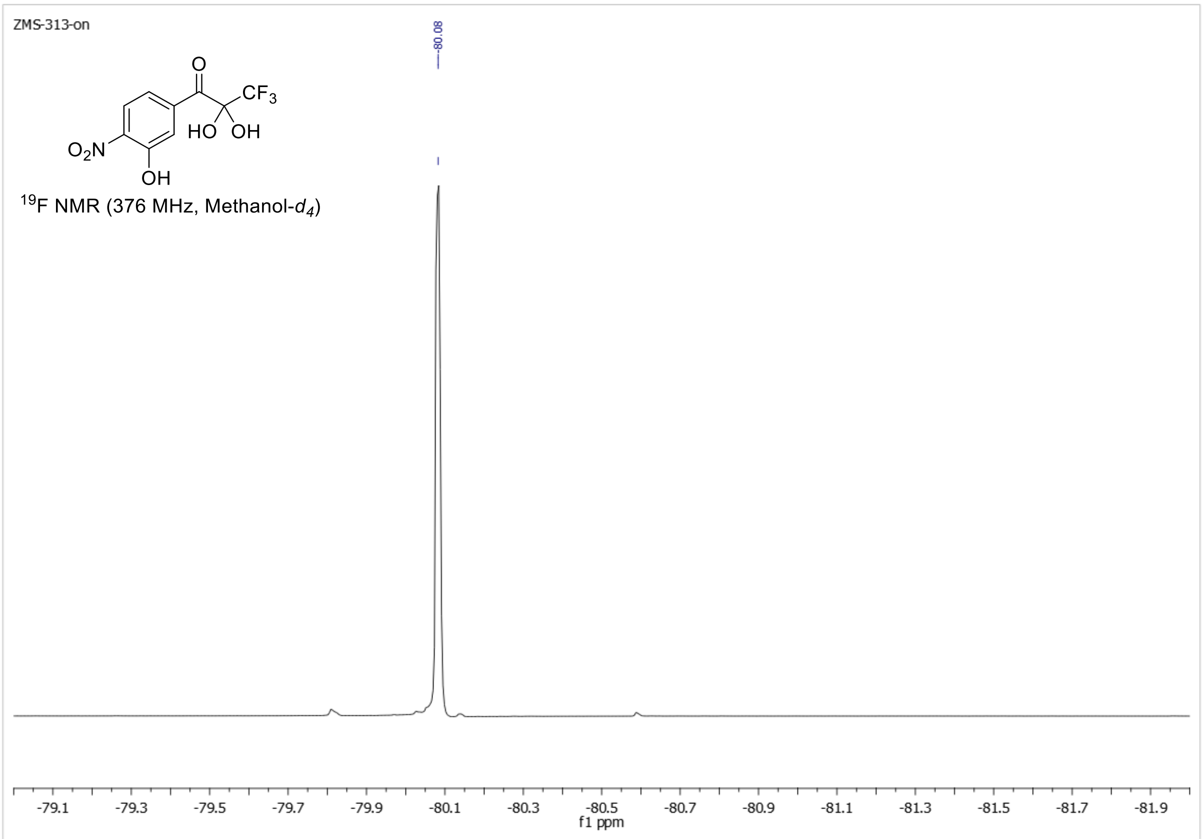

Compound **1f**, ^{13}C NMR:

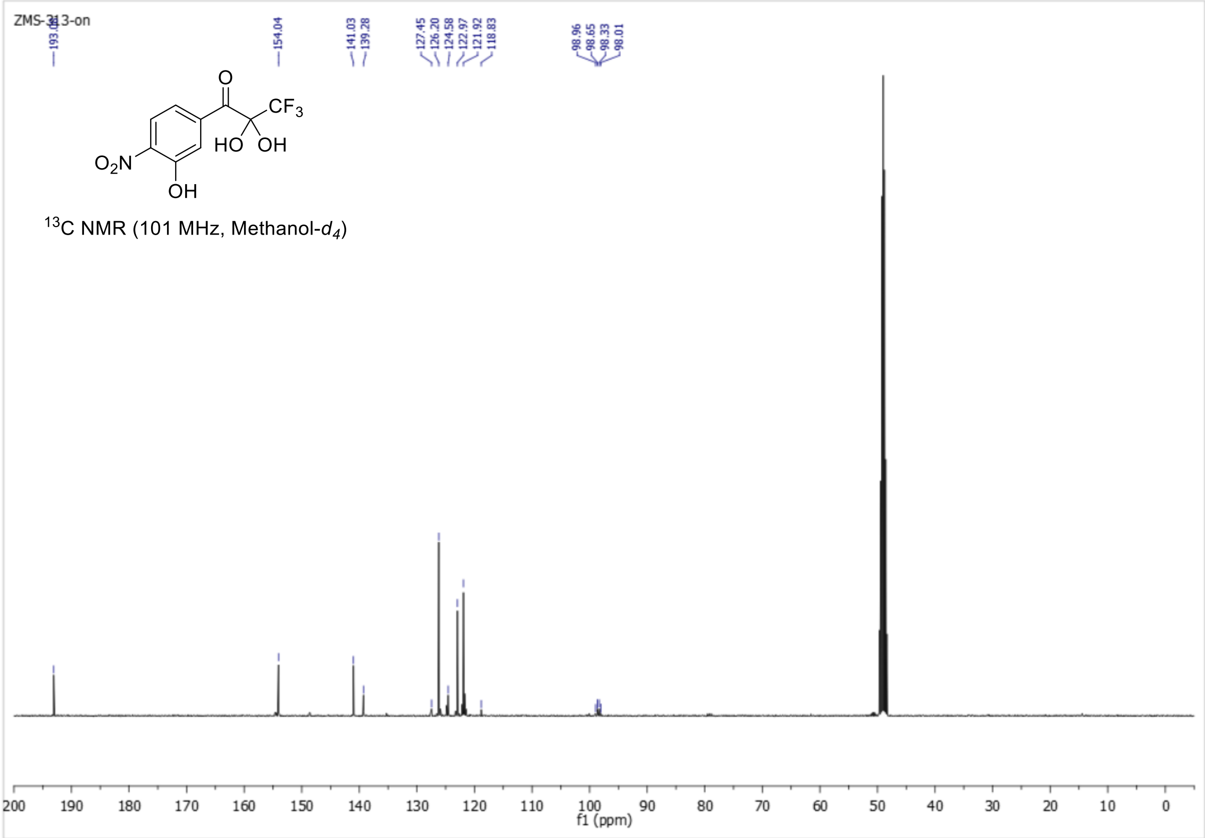

Compound **1h**, ^{1}H NMR:

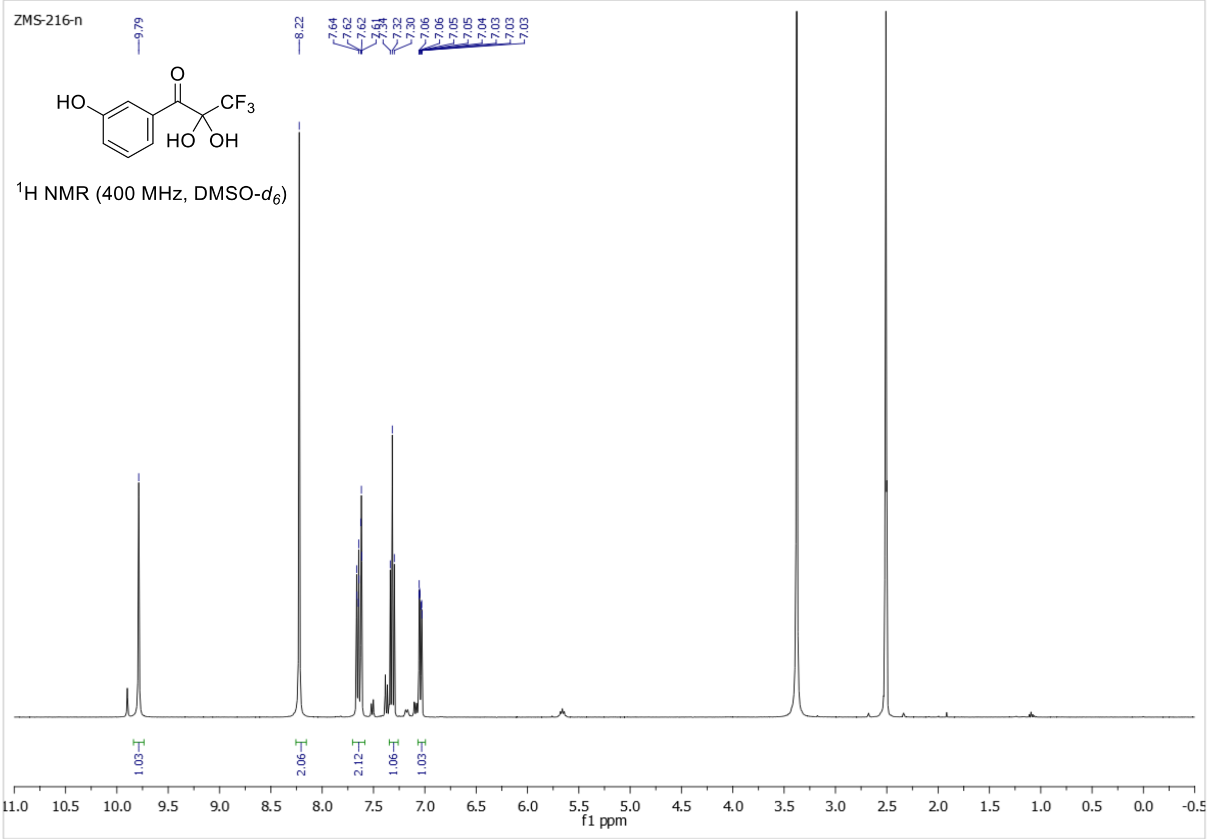

Compound **1h**, ^{19}F NMR:

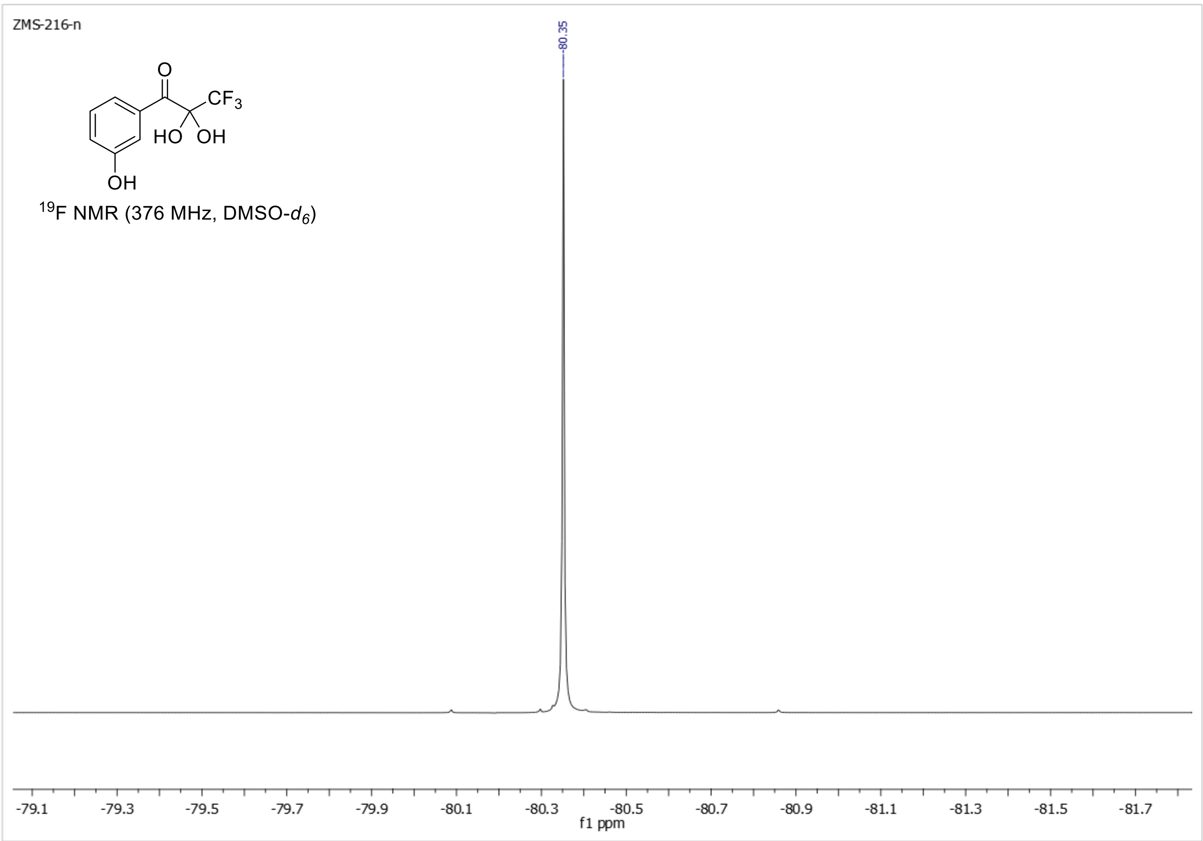

Compound **1h**, ^{13}C NMR:

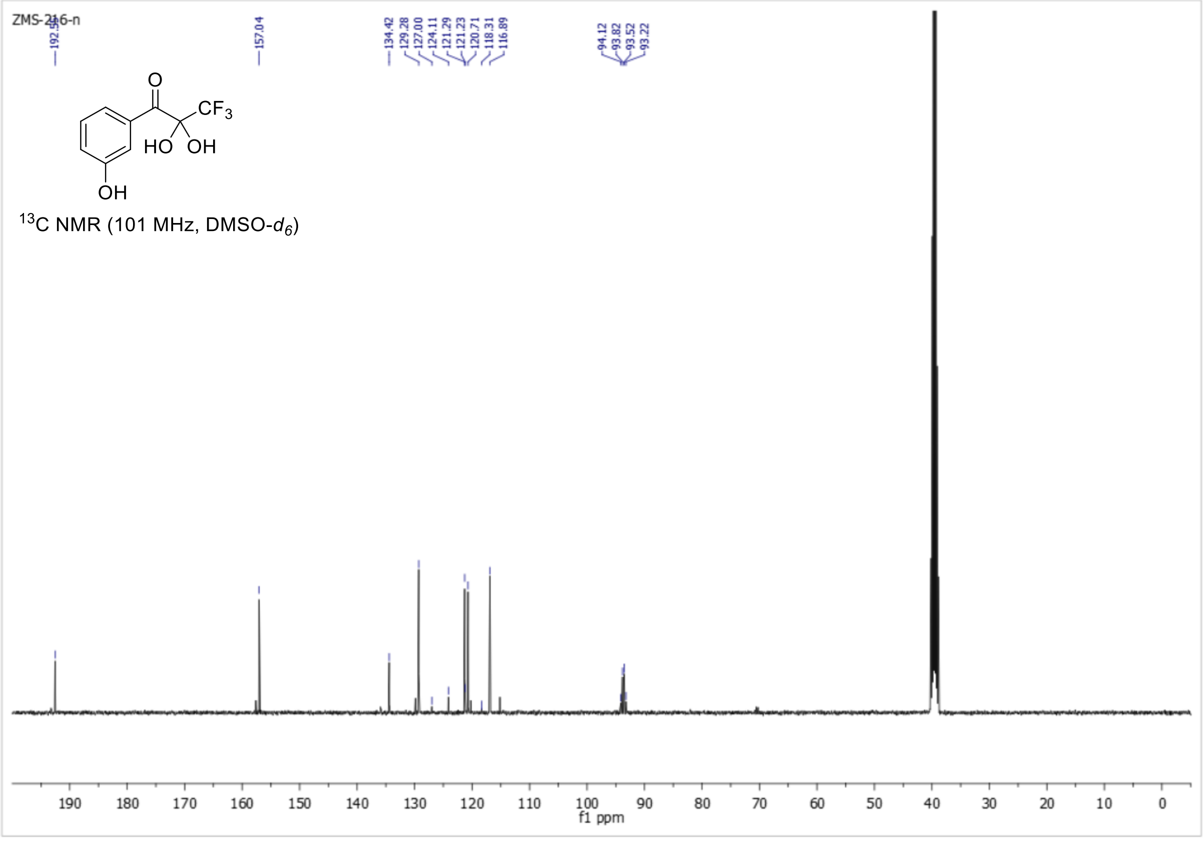

Compound **1i**, ^{1}H NMR:

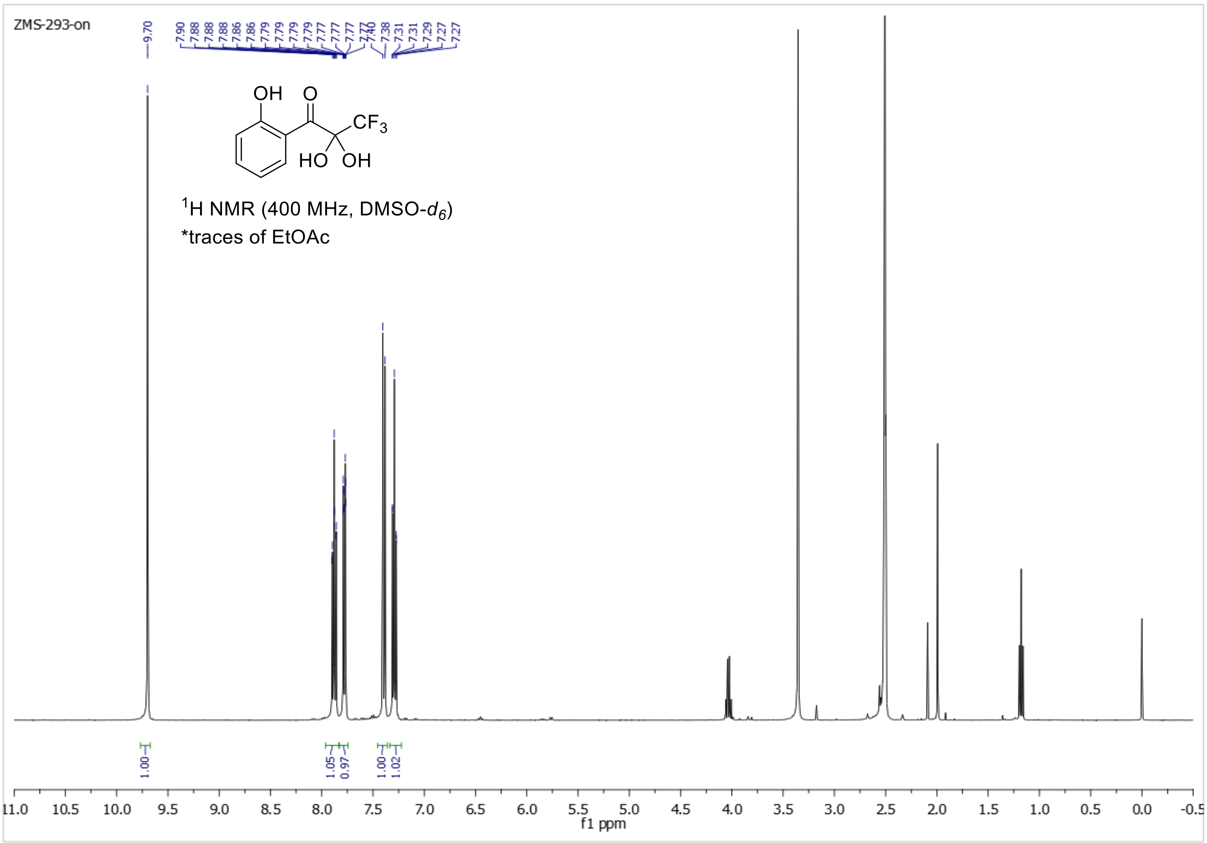

Compound **1i**, ^{19}F NMR:

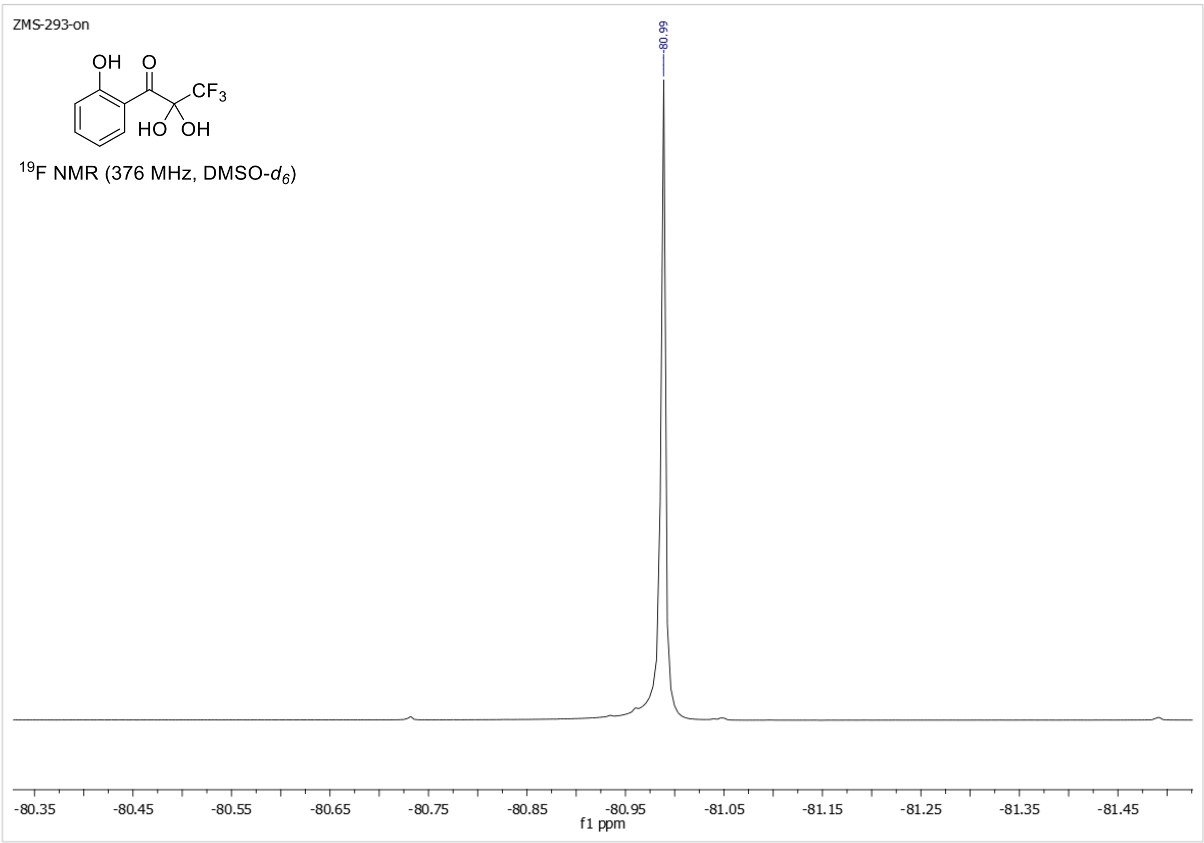

Compound **1i**, ^{13}C NMR:

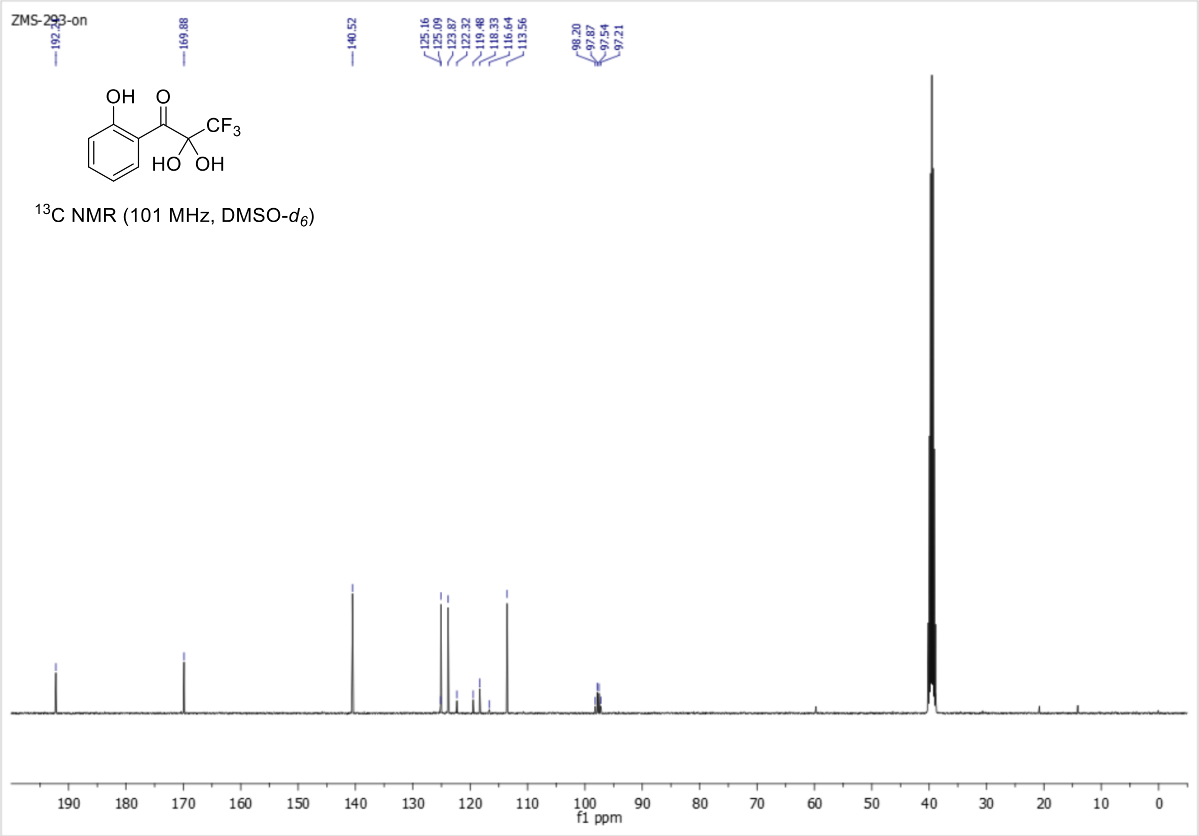

Compound **1j**, ^{1}H NMR:

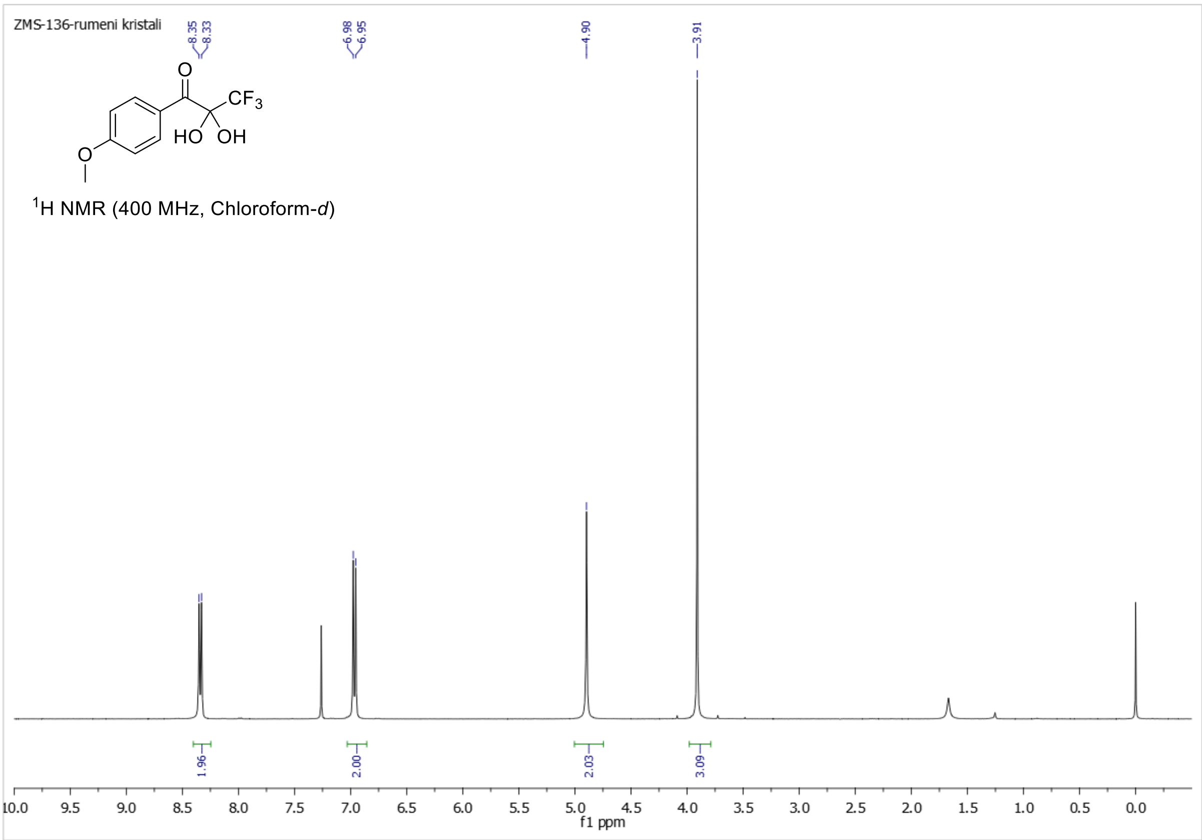

Compound **1j**, ^{19}F NMR:

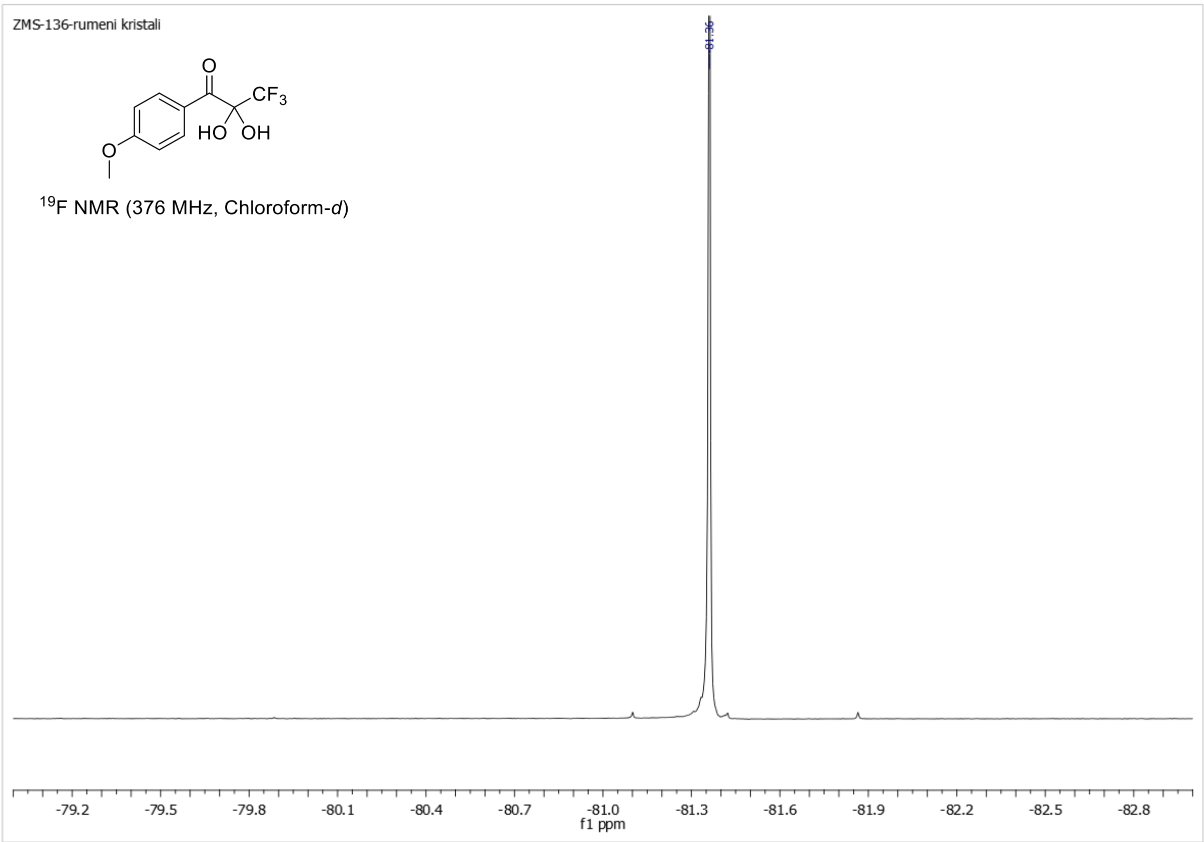

Compound **1j**, ^{13}C NMR:

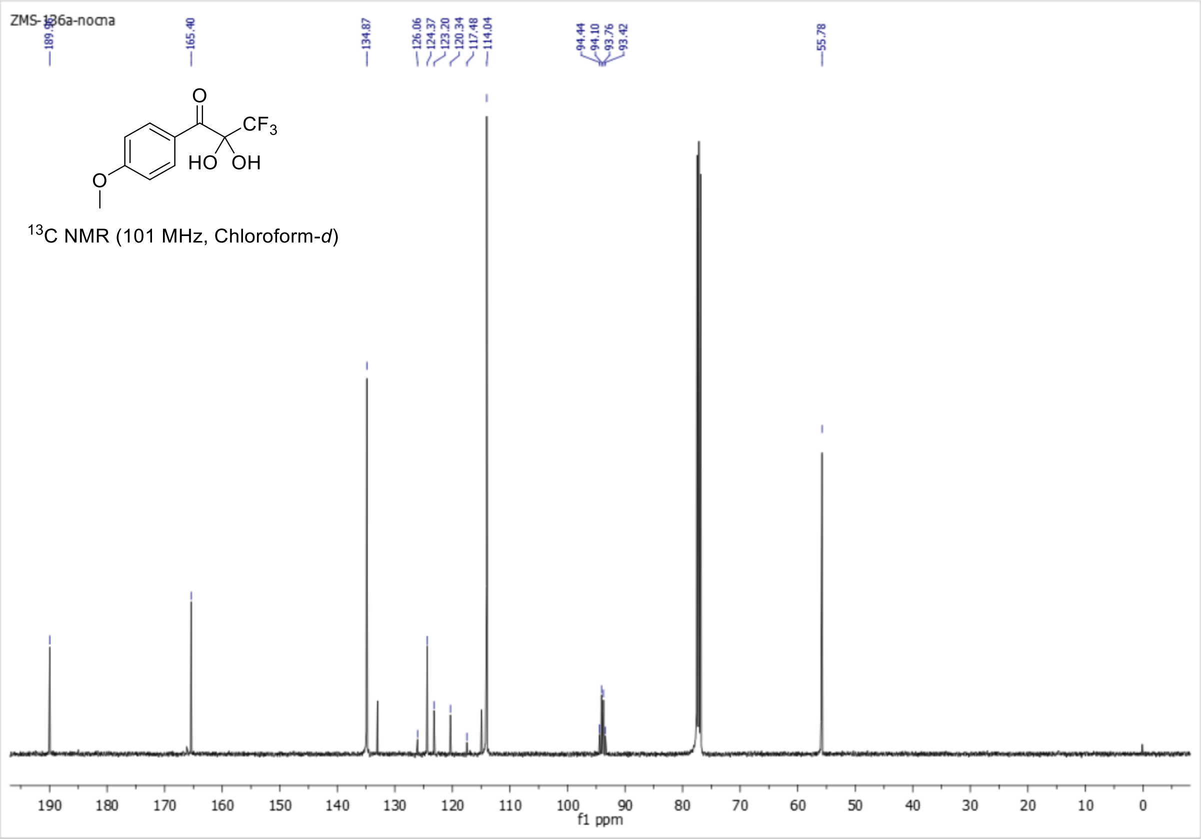

Compound **1k**, ^{1}H NMR:

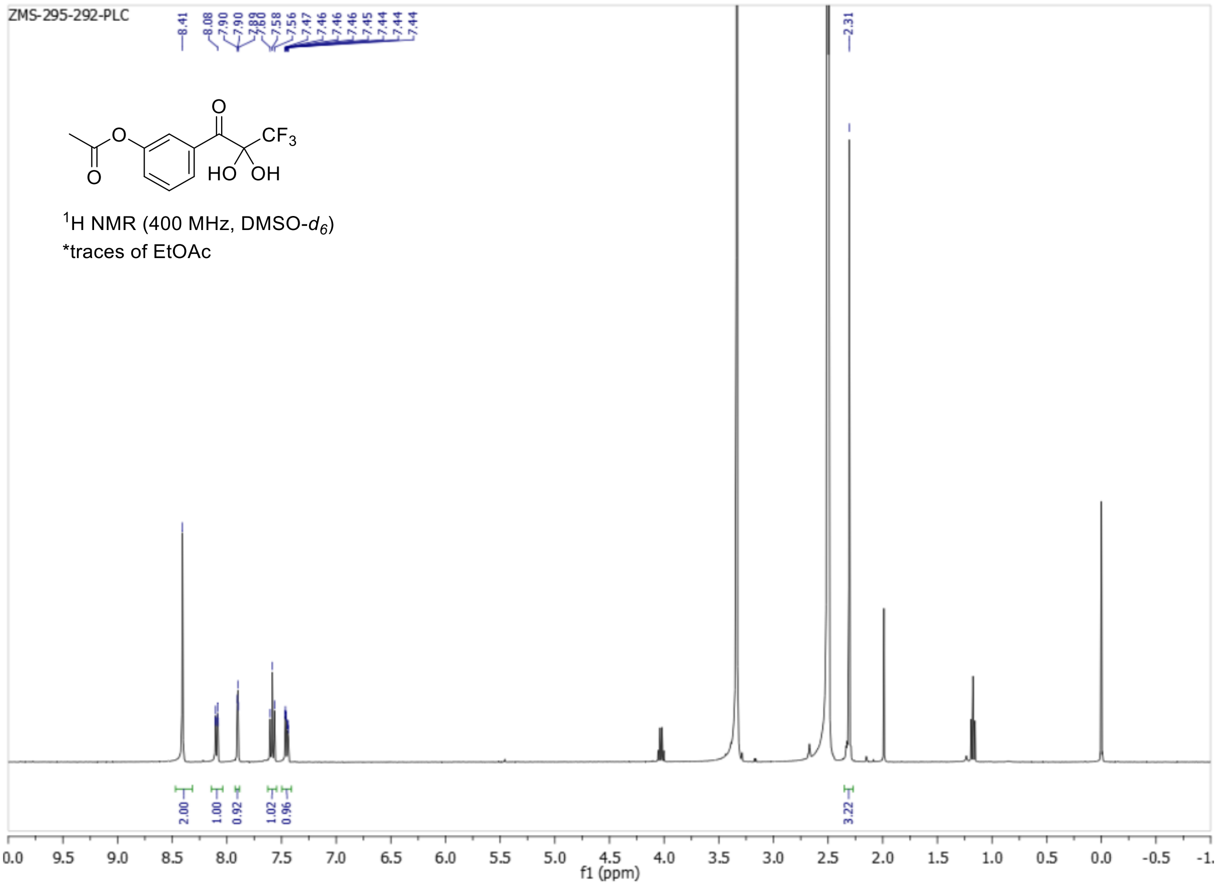

Compound **1k**, ^{19}F NMR:

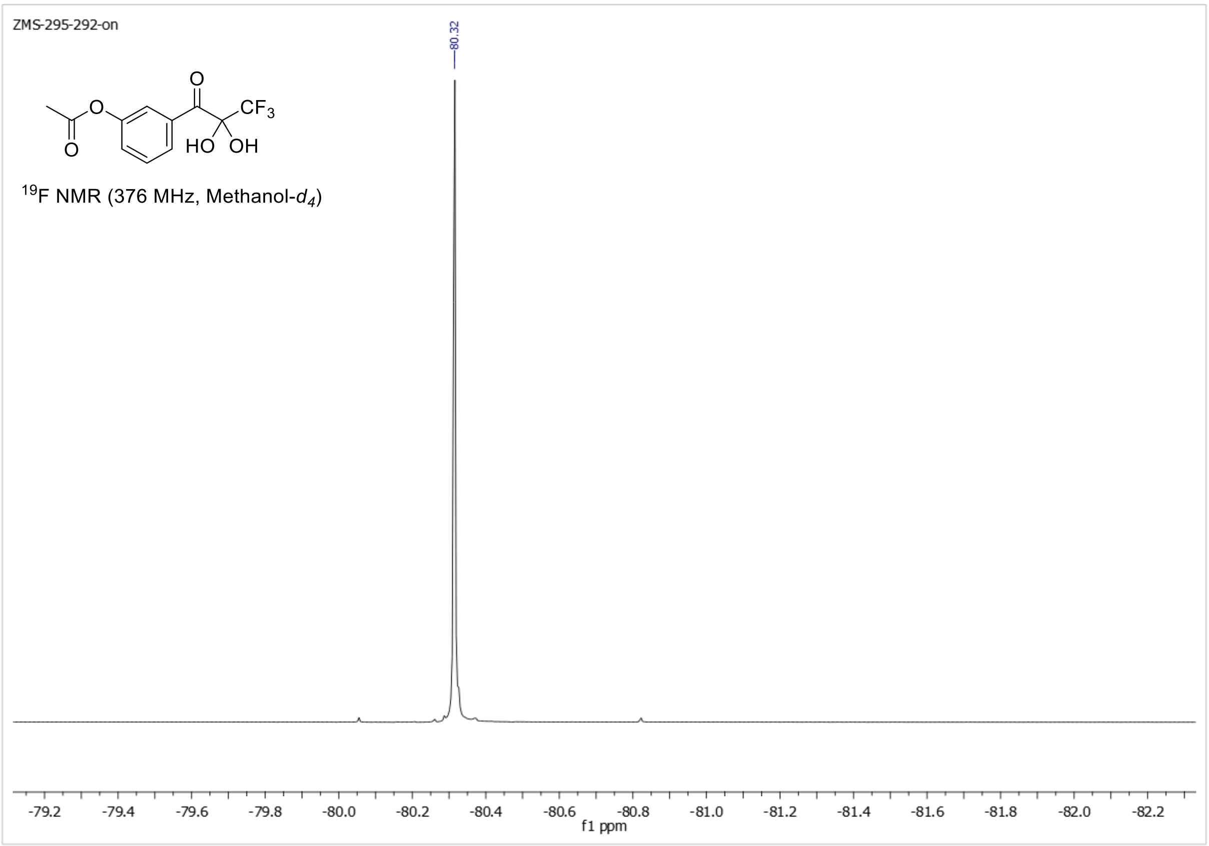

Compound **1k**, ^{13}C NMR:

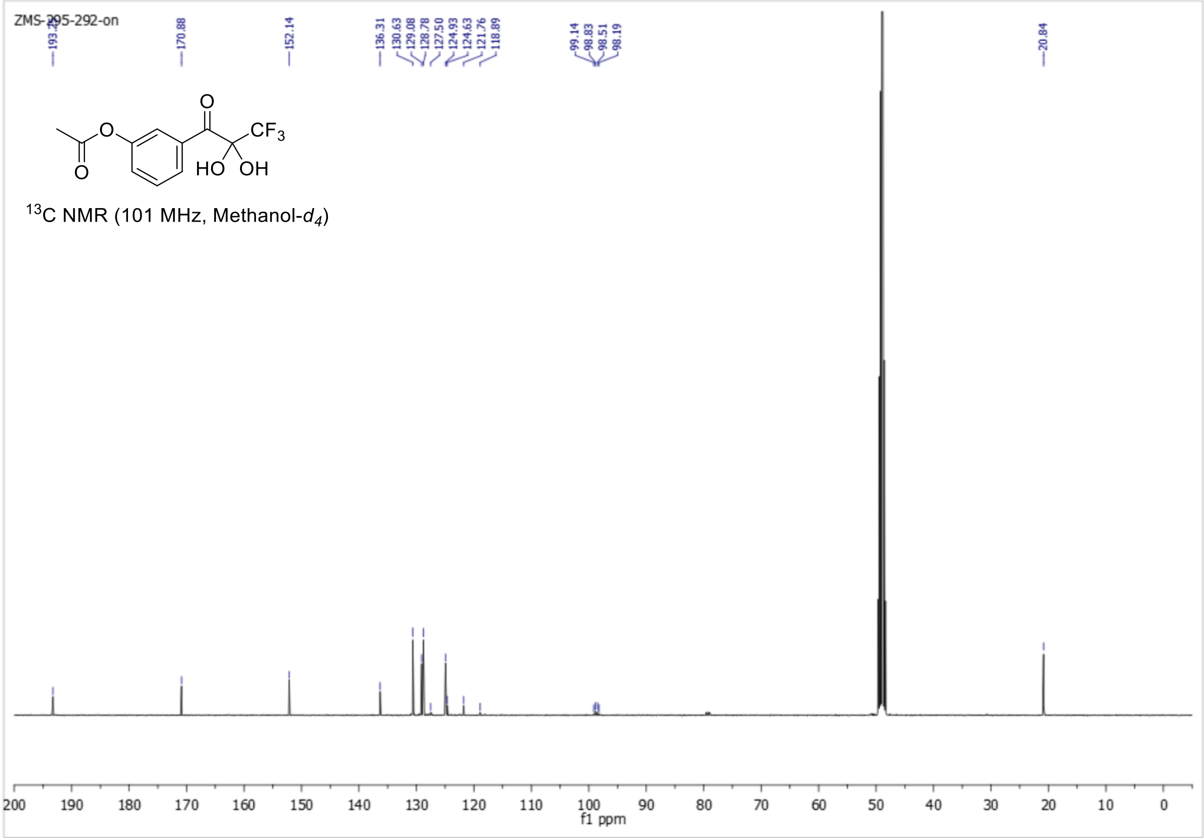

Compound **1l**, ^{1}H NMR:

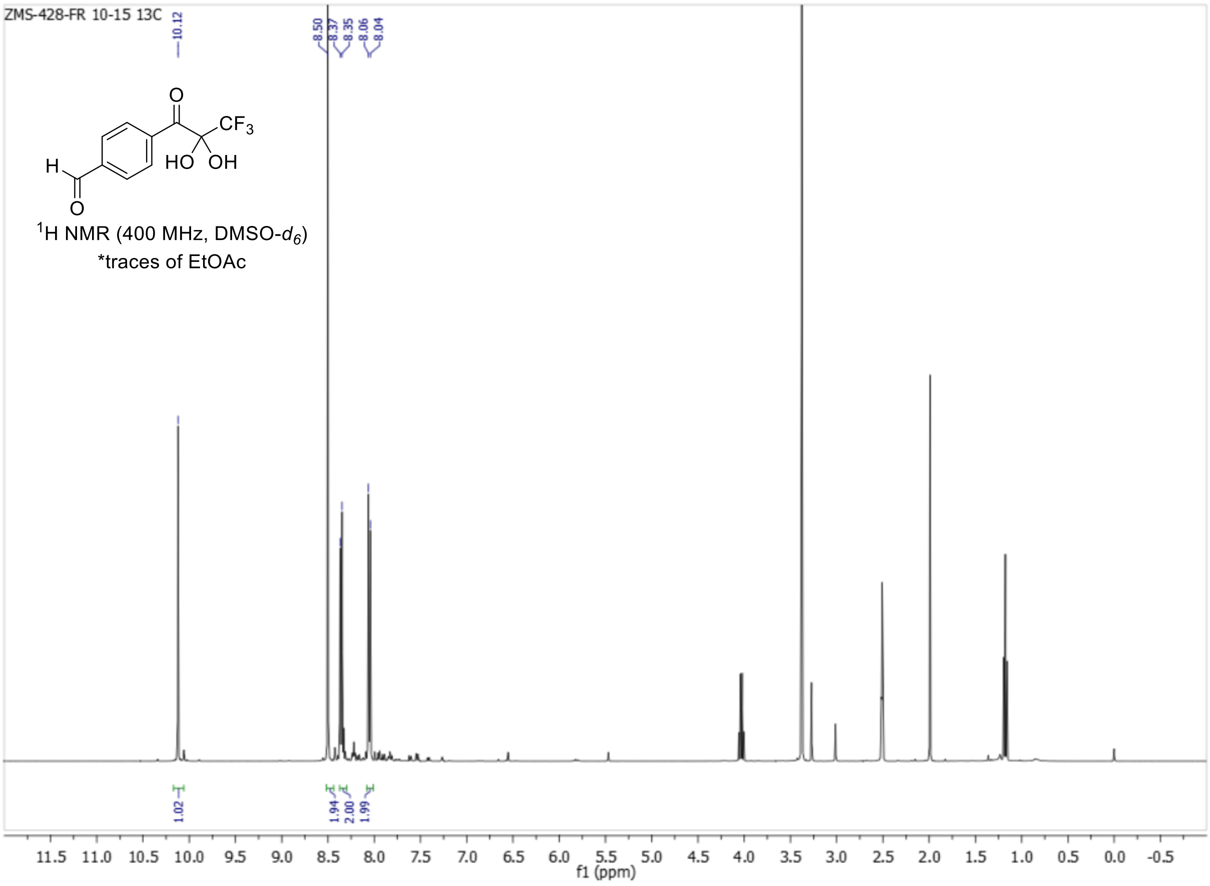

Compound **1l**, ^{19}F NMR:

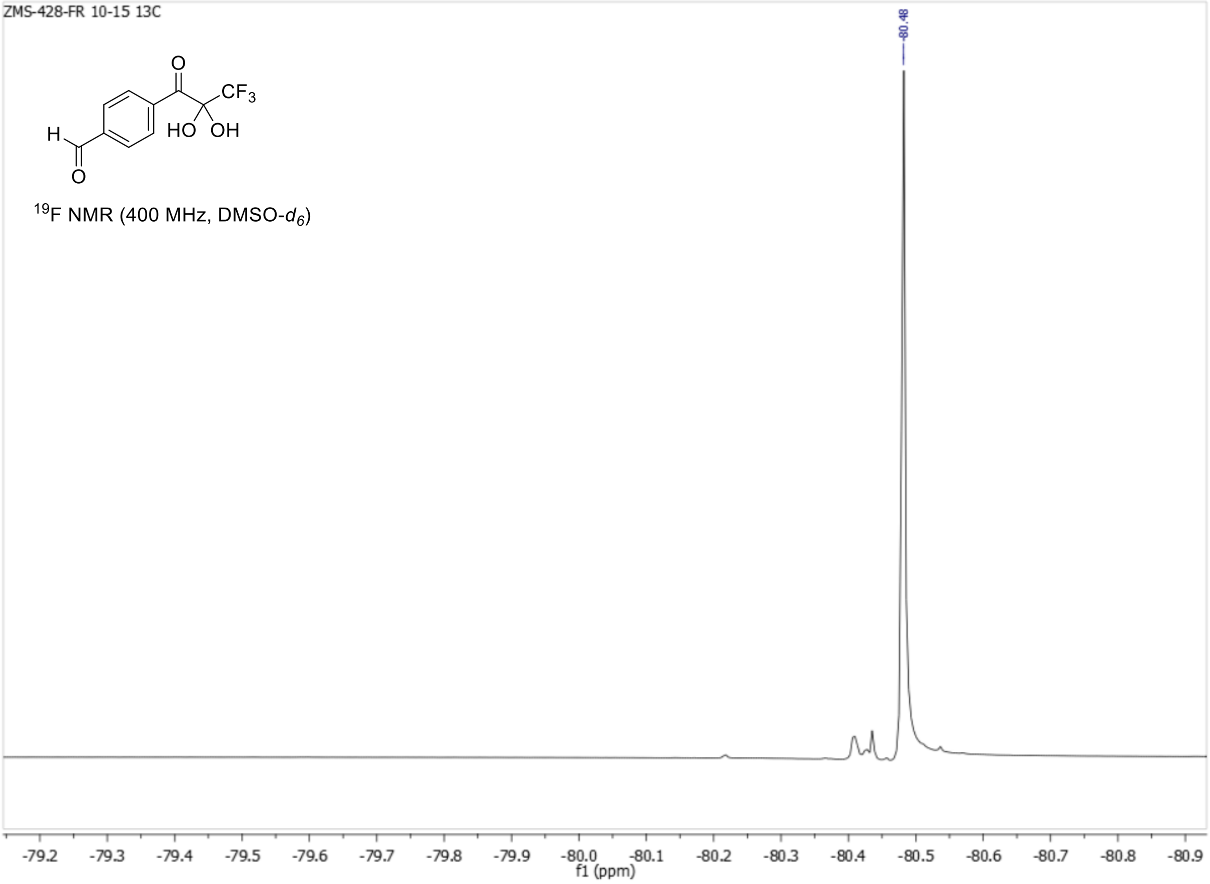

Compound **1l**, ^{13}C NMR:

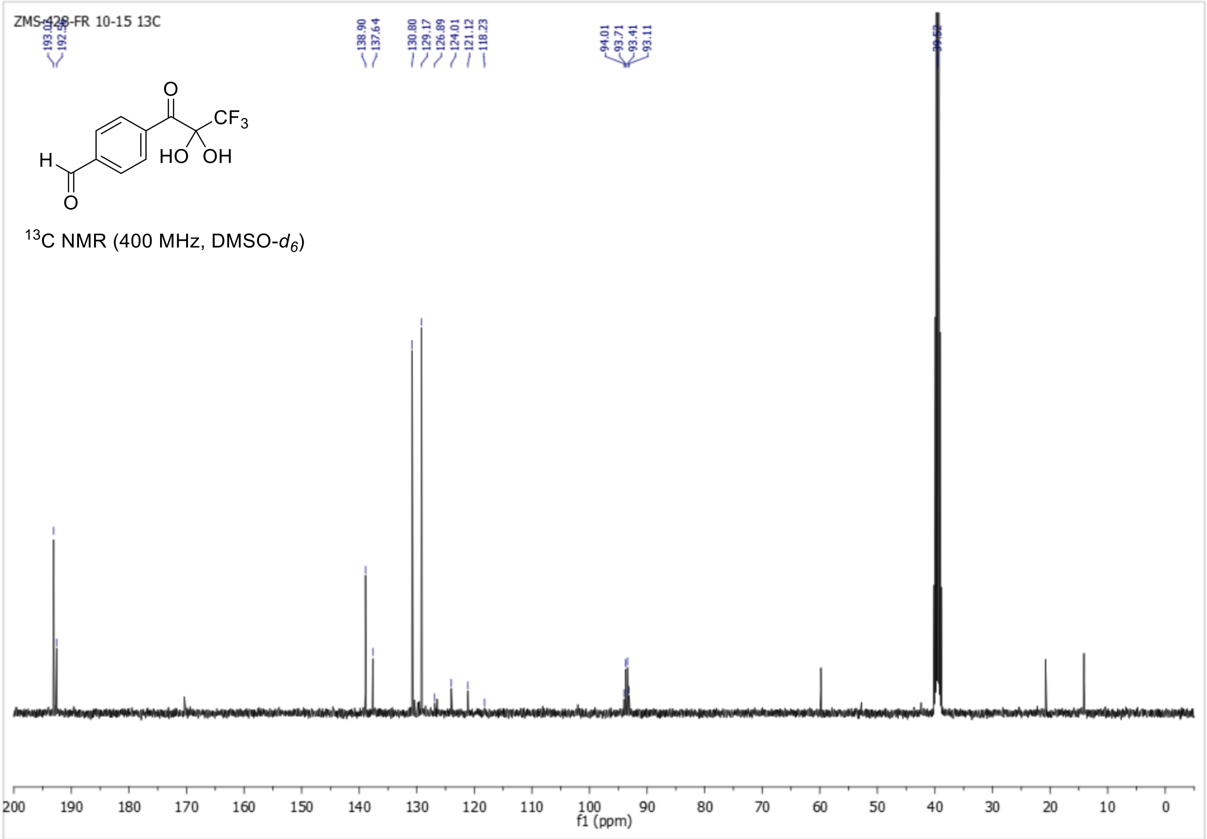

Compound **1n**, ^{1}H NMR:

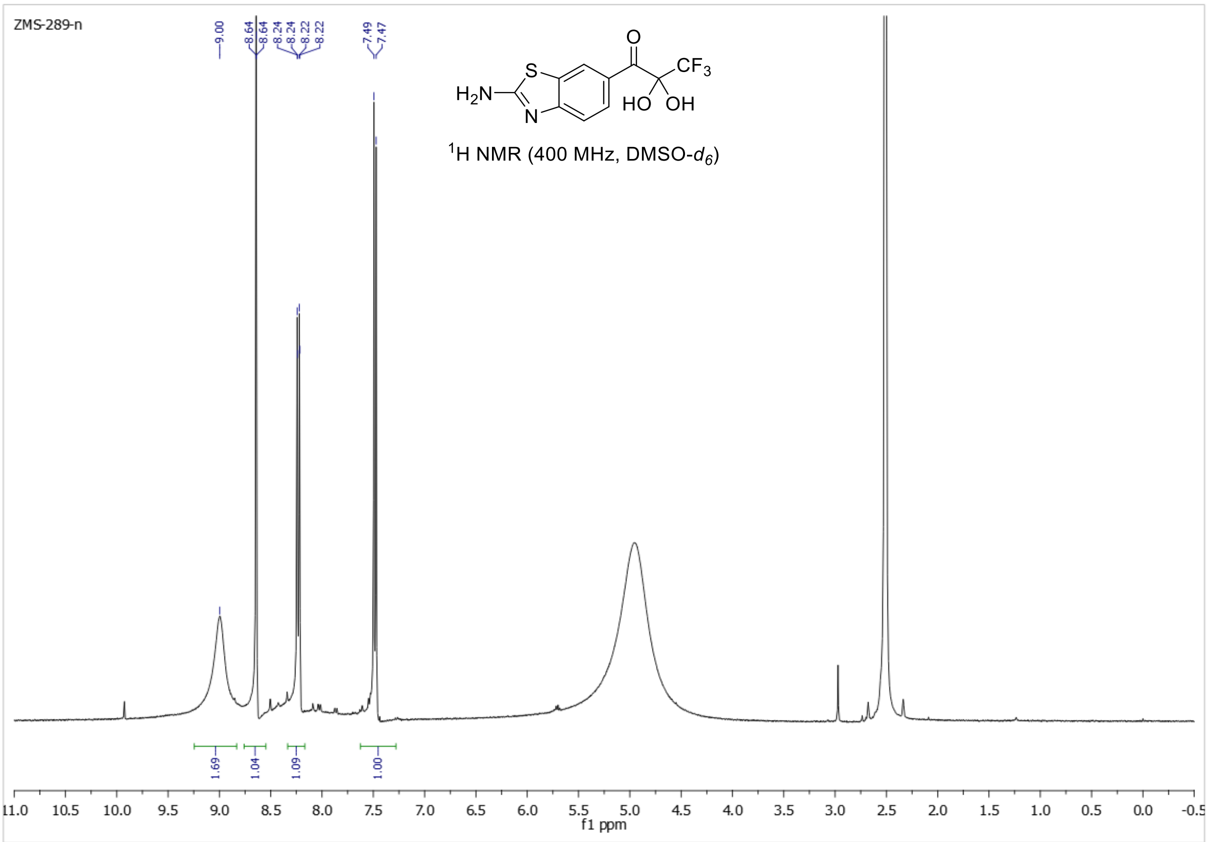

Compound **1n**, ^{19}F NMR:

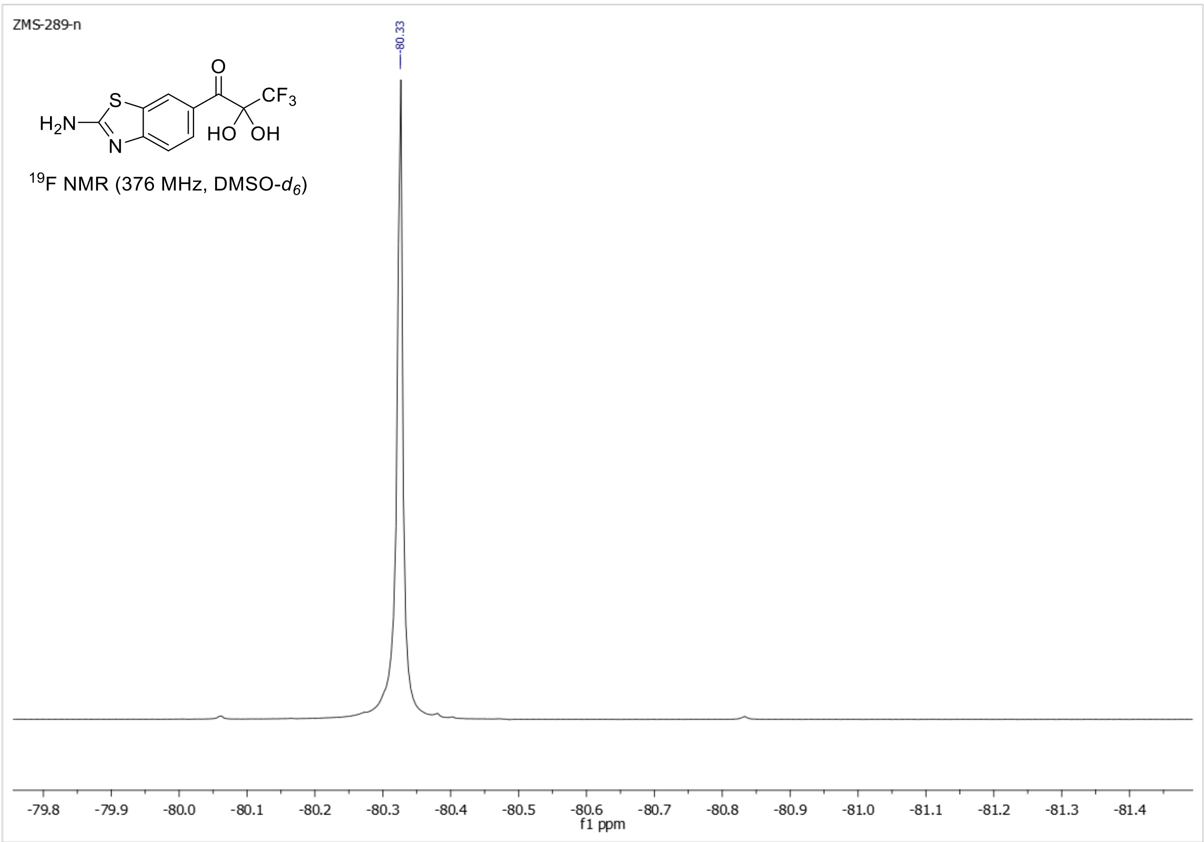

Compound **1n**, ^{13}C NMR:

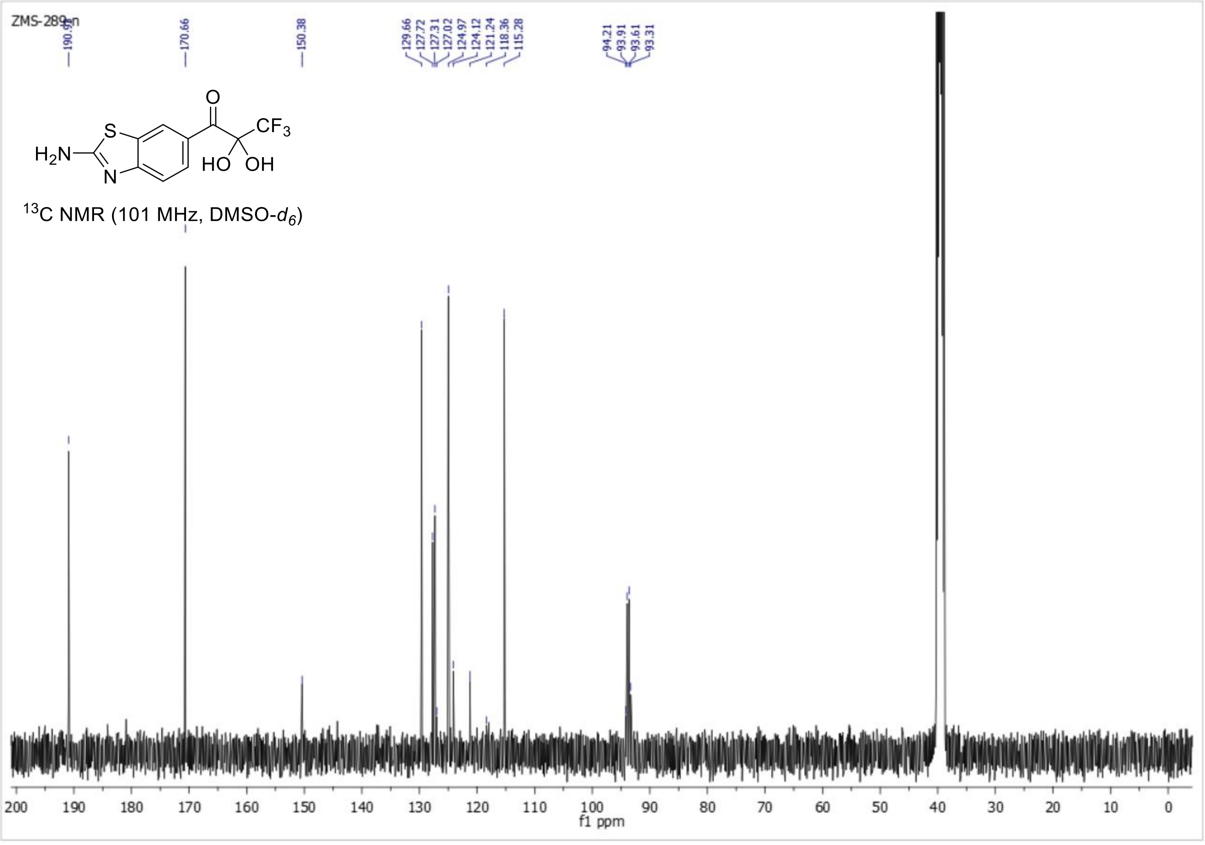

Compound **2a**, ^{1}H NMR:

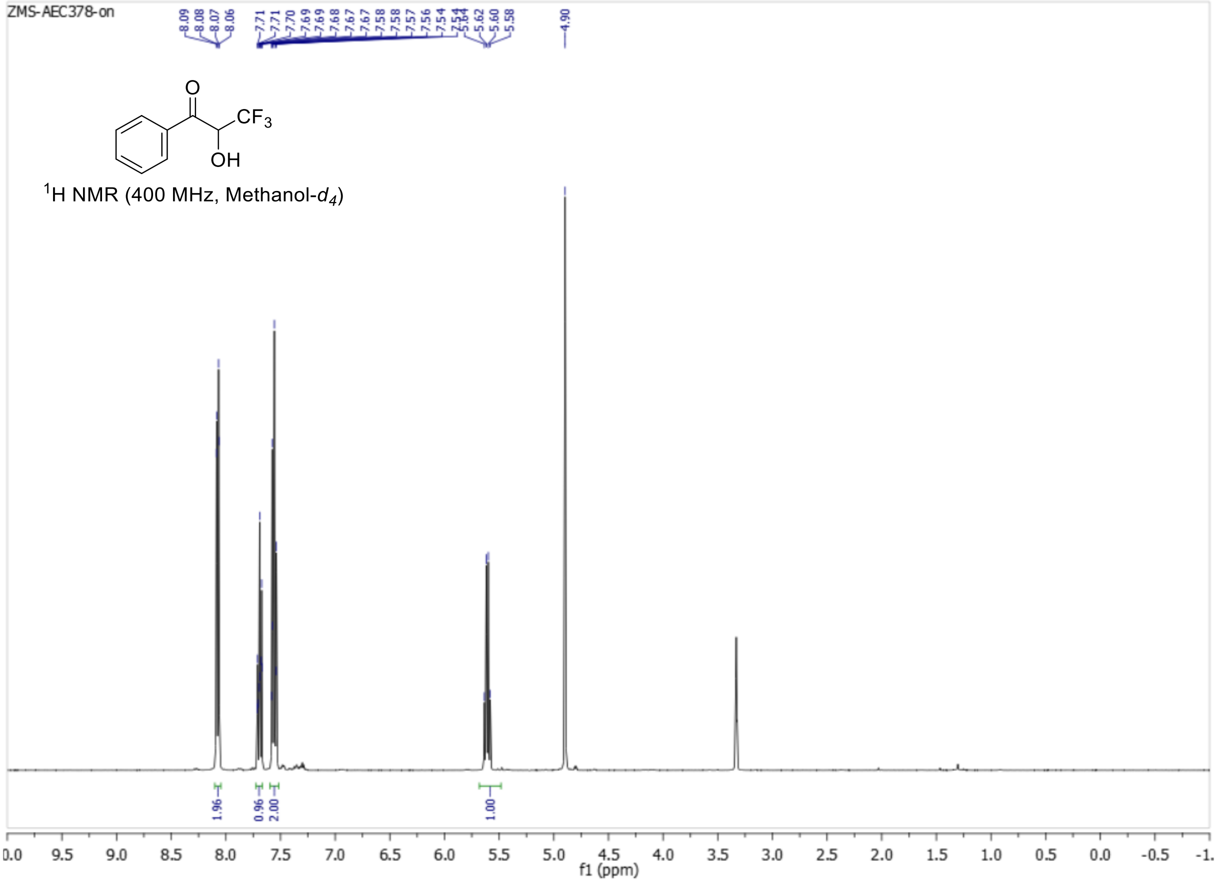

Compound **2a**, ^{19}F NMR:

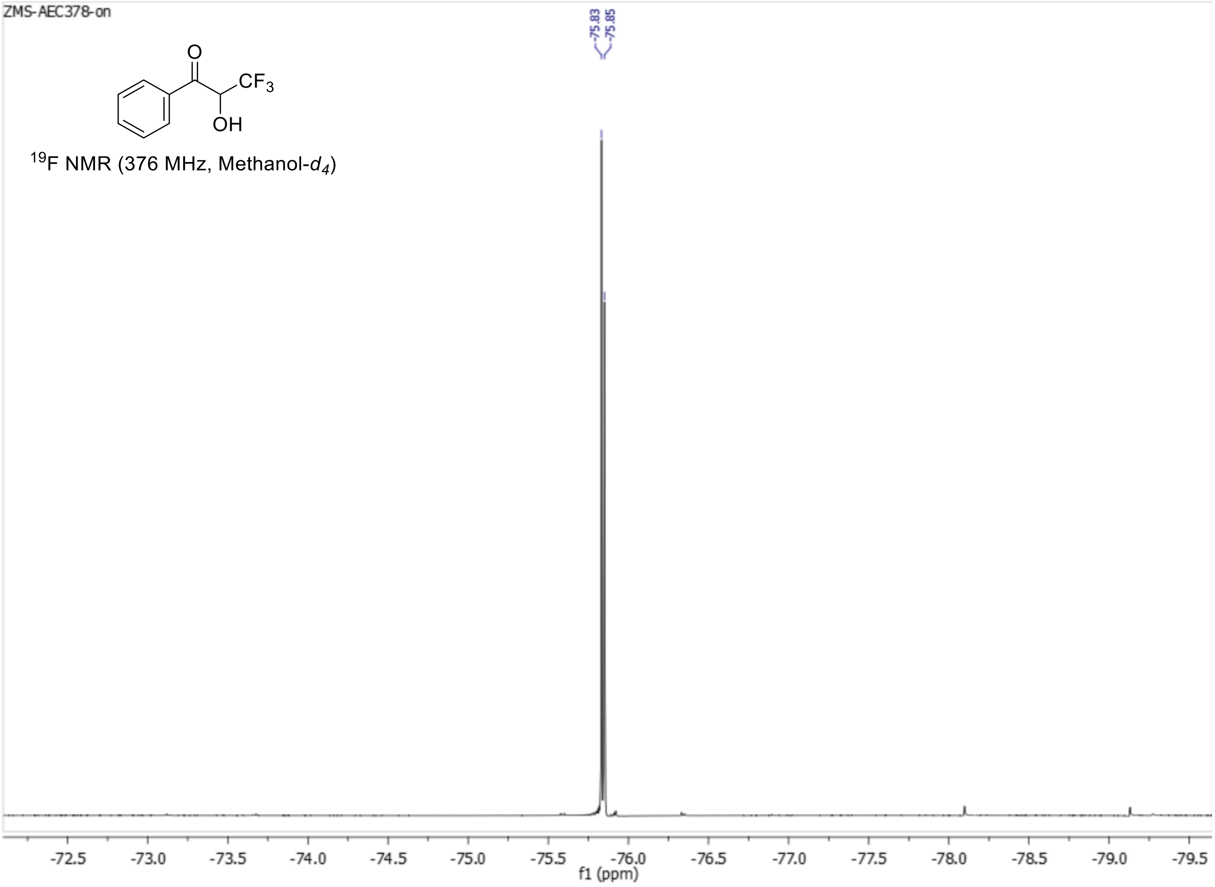

Compound **2a**, ^{13}C NMR:

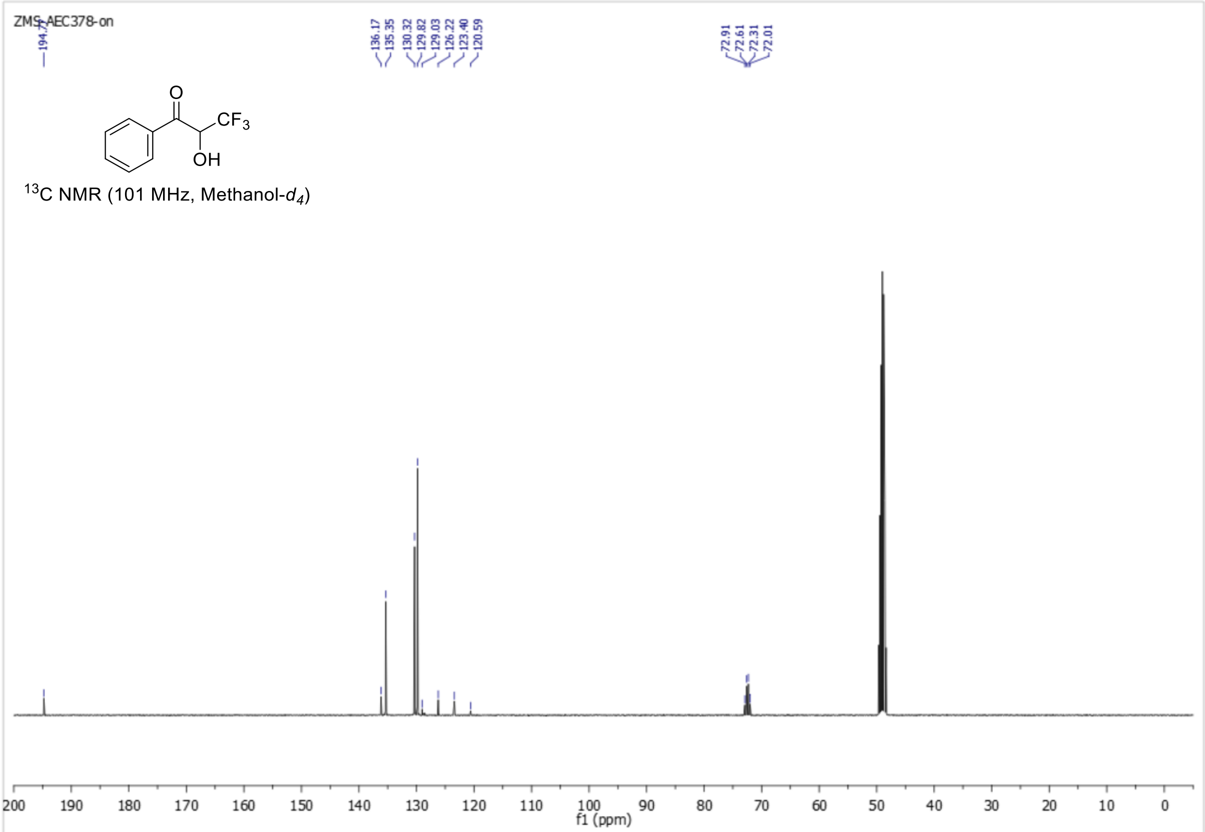

Compound **2a** ^{1}H NMR:

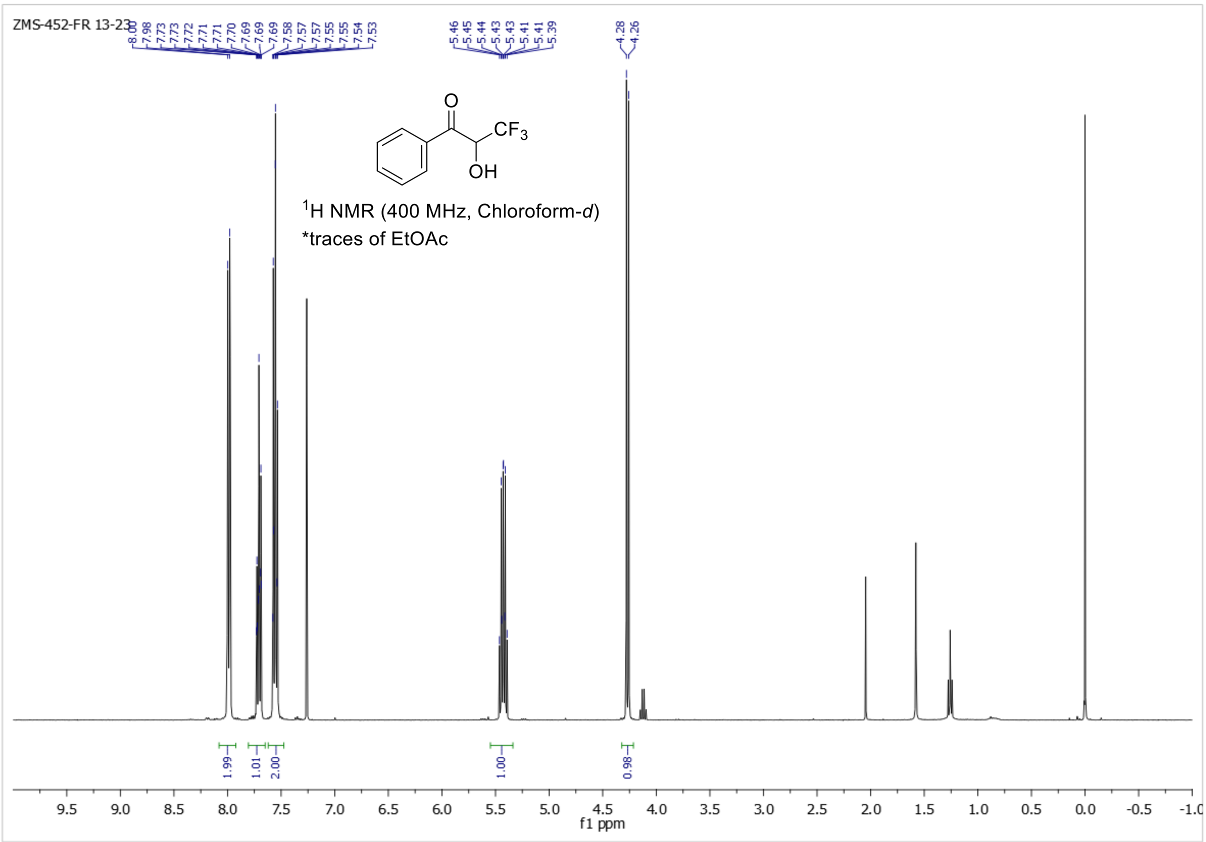

Compound **2a**, ^{19}F NMR:

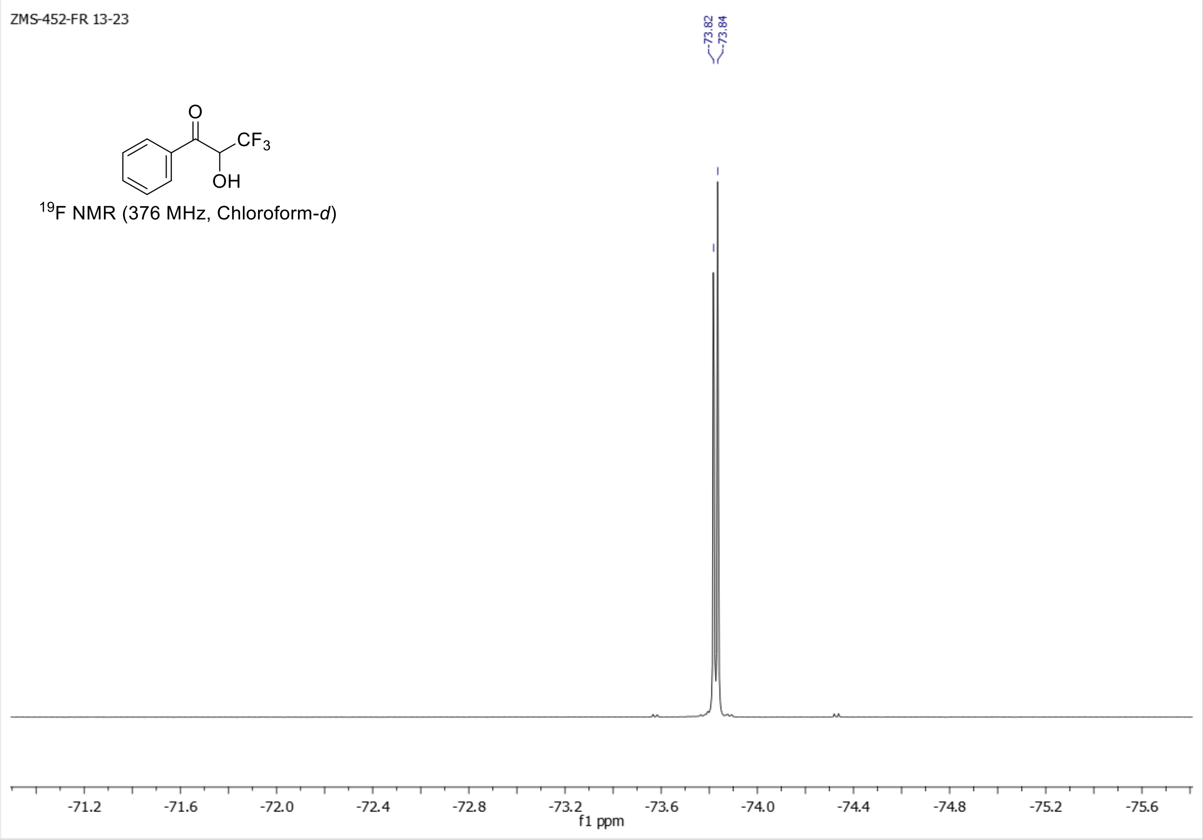

Compound **2c**, ^{1}H NMR:

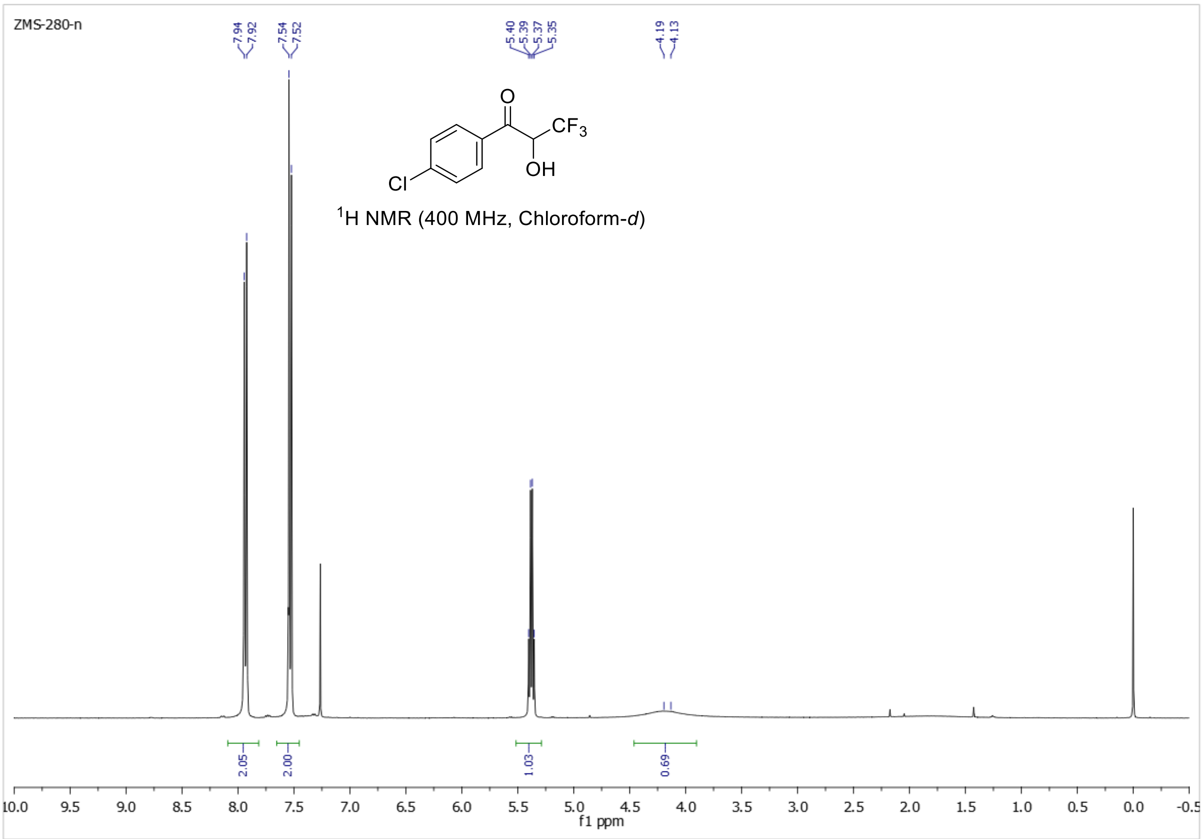

Compound **2c**, ^{19}F NMR:

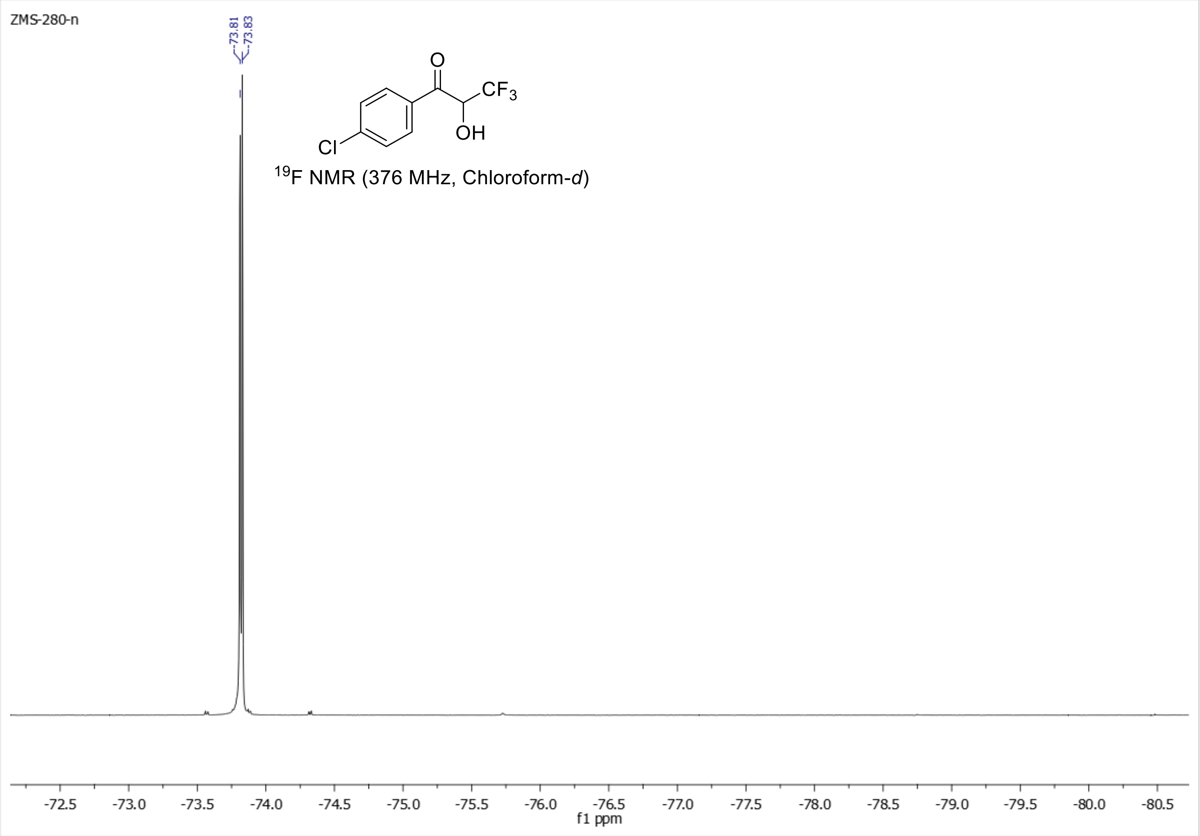

Compound **2c**, ^{13}C NMR:

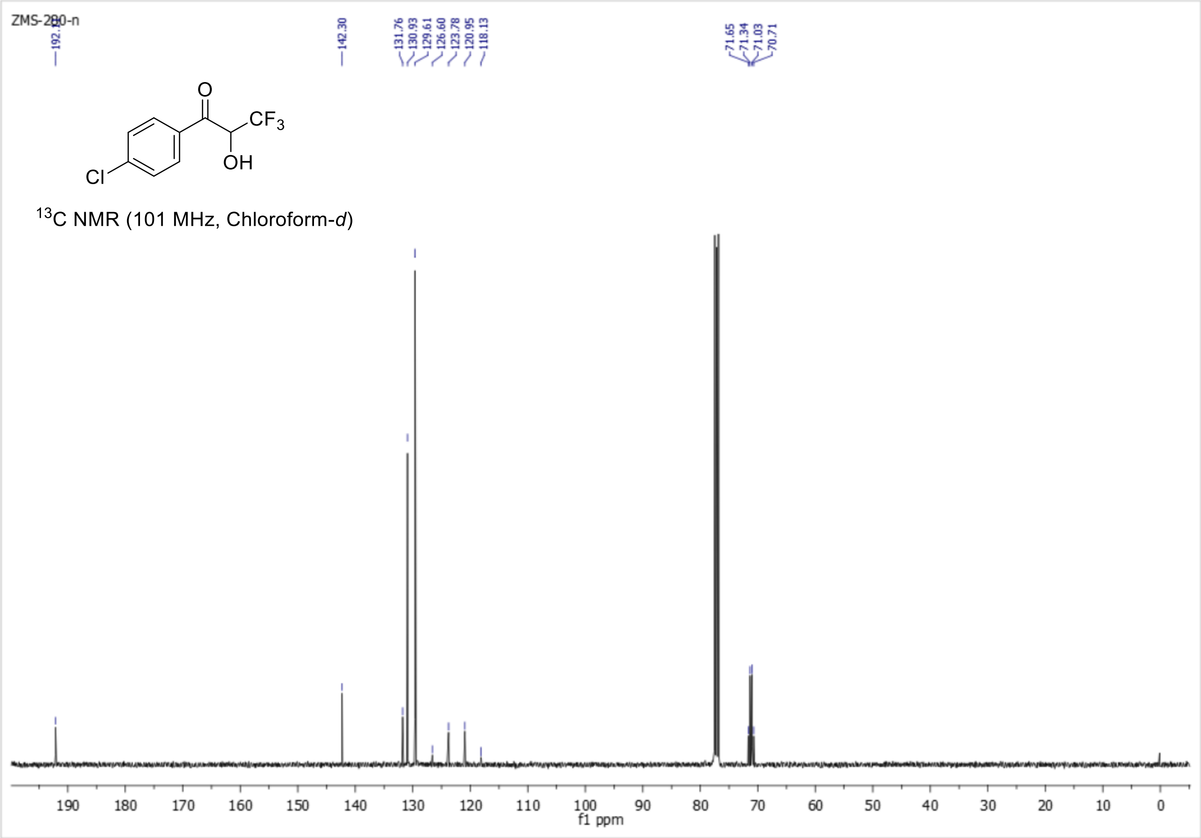

Compound **2d**, ^{1}H NMR:

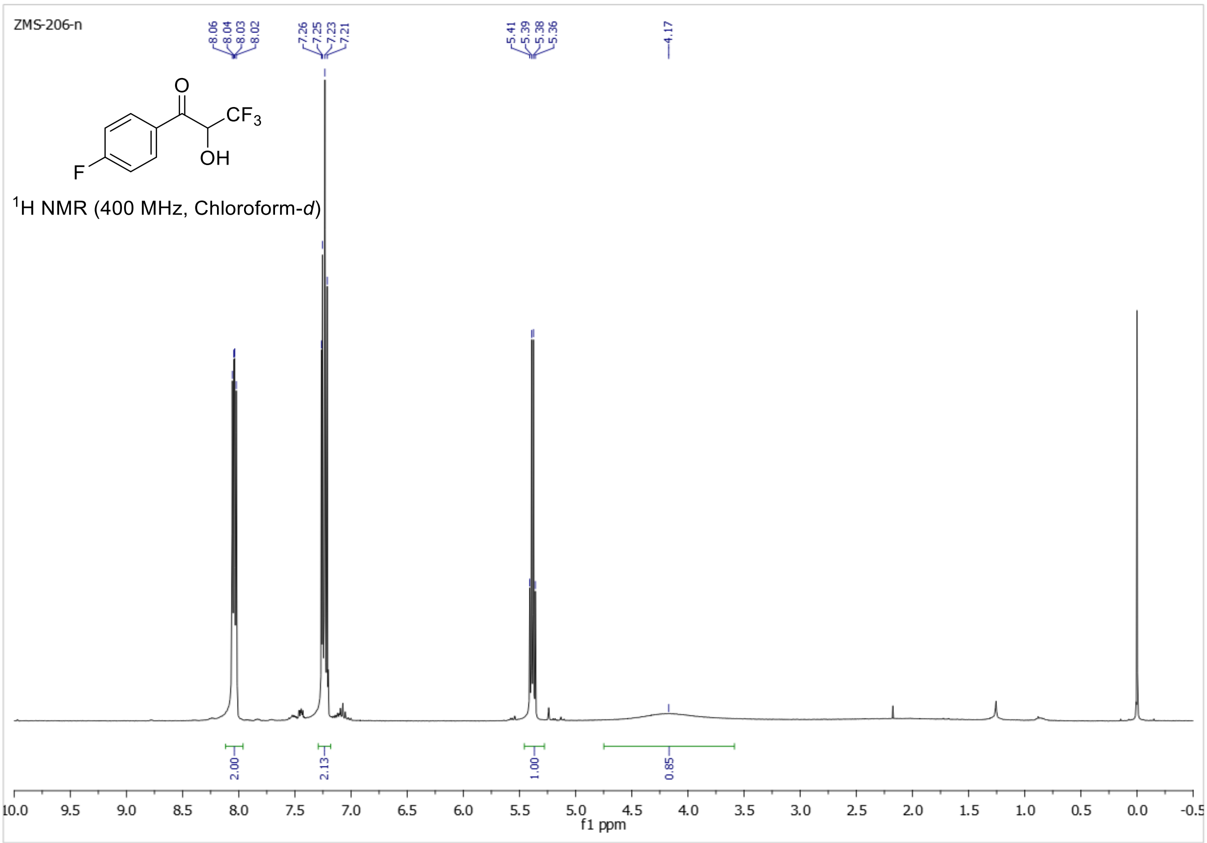

Compound **2d**, ^{19}F NMR:

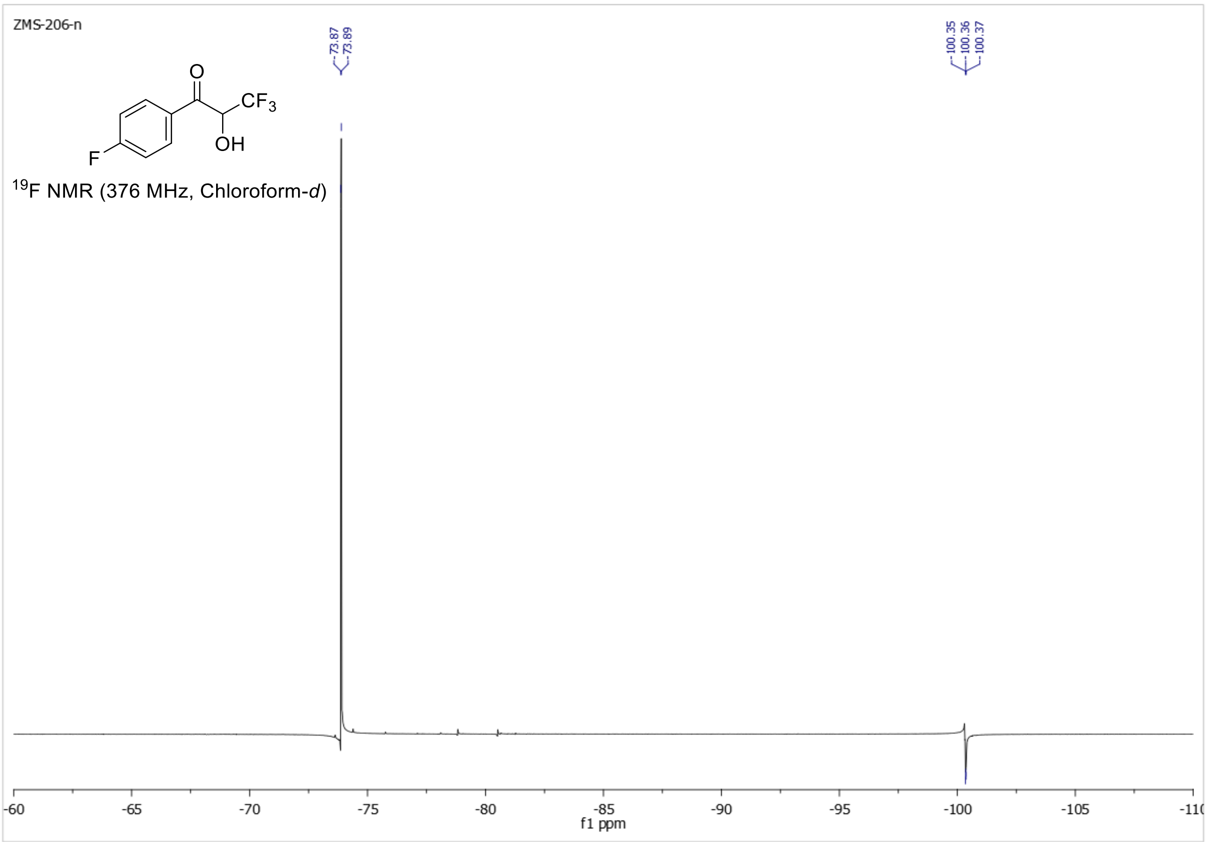

Compound **2d**, ^{13}C NMR:

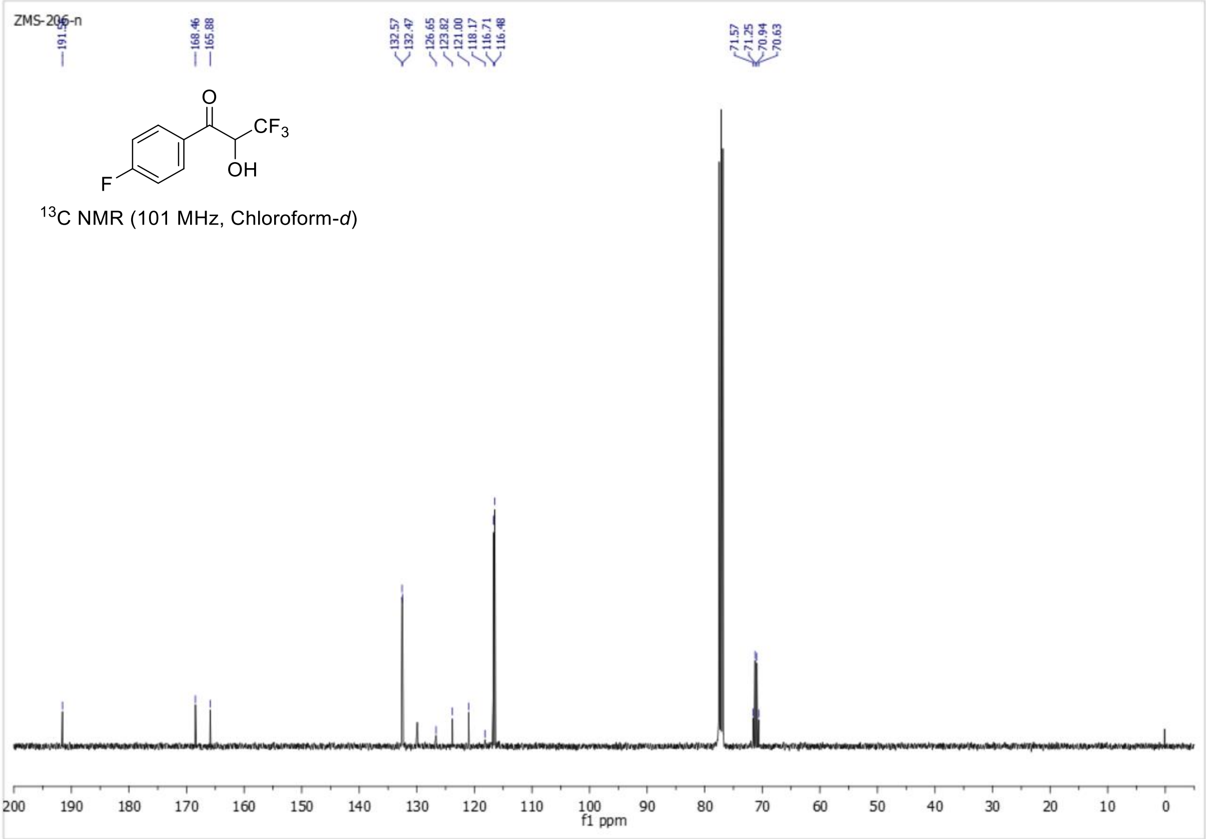

Compound **2g**, ^{1}H NMR:

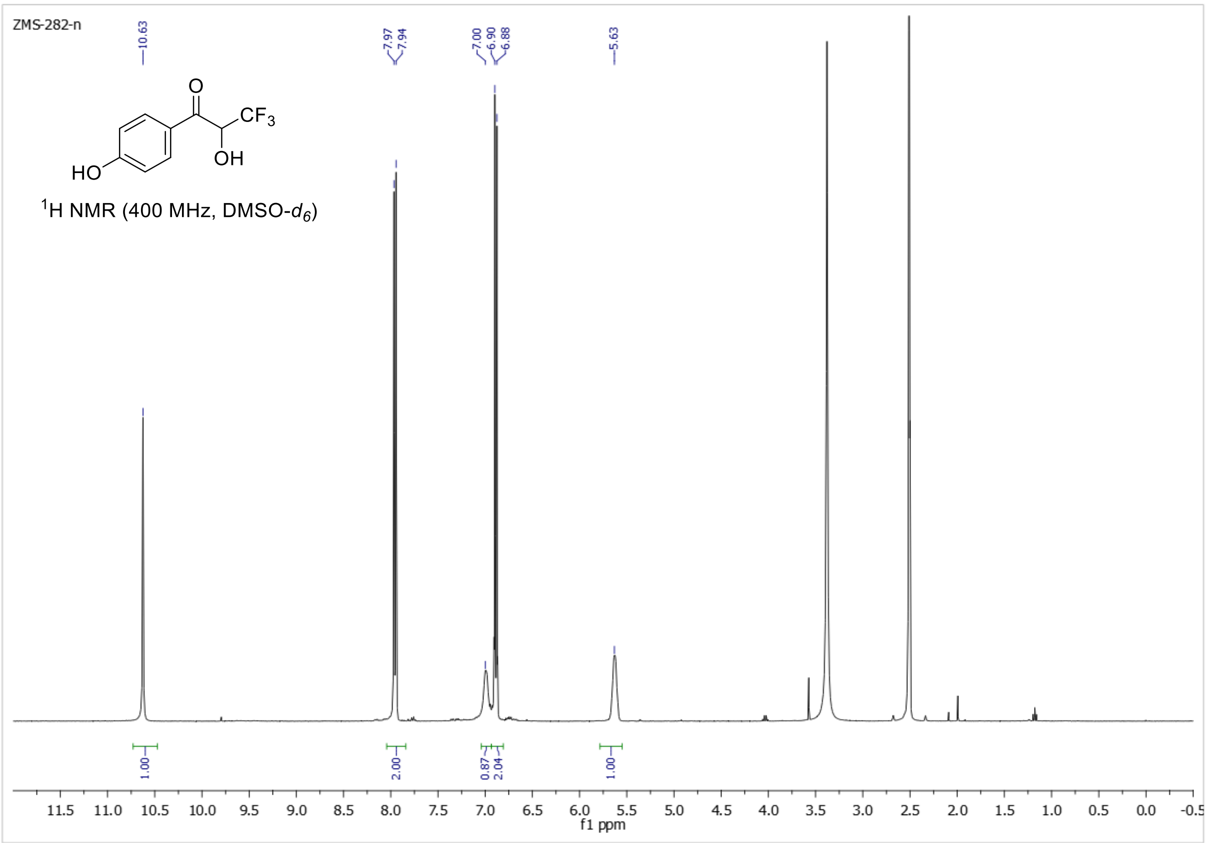

Compound **2g**, ^{19}F NMR:

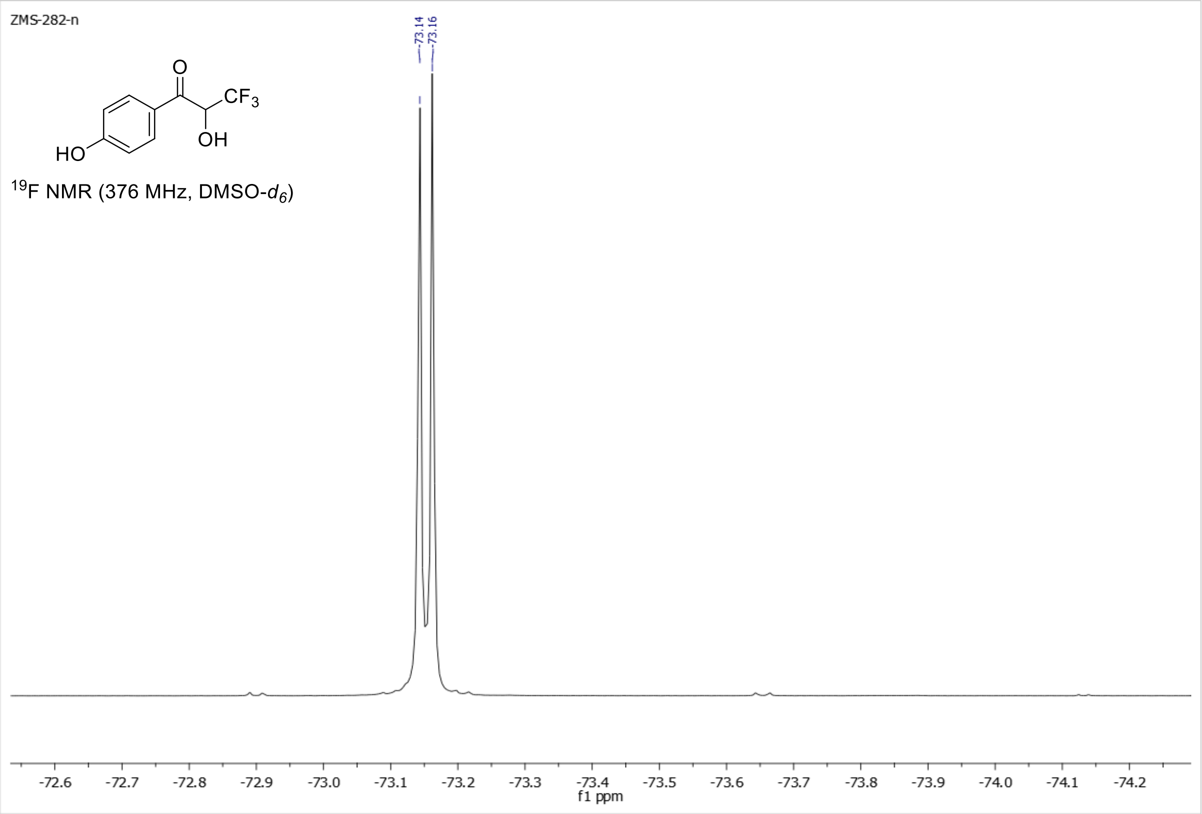

Compound **2g**, ^{13}C NMR:

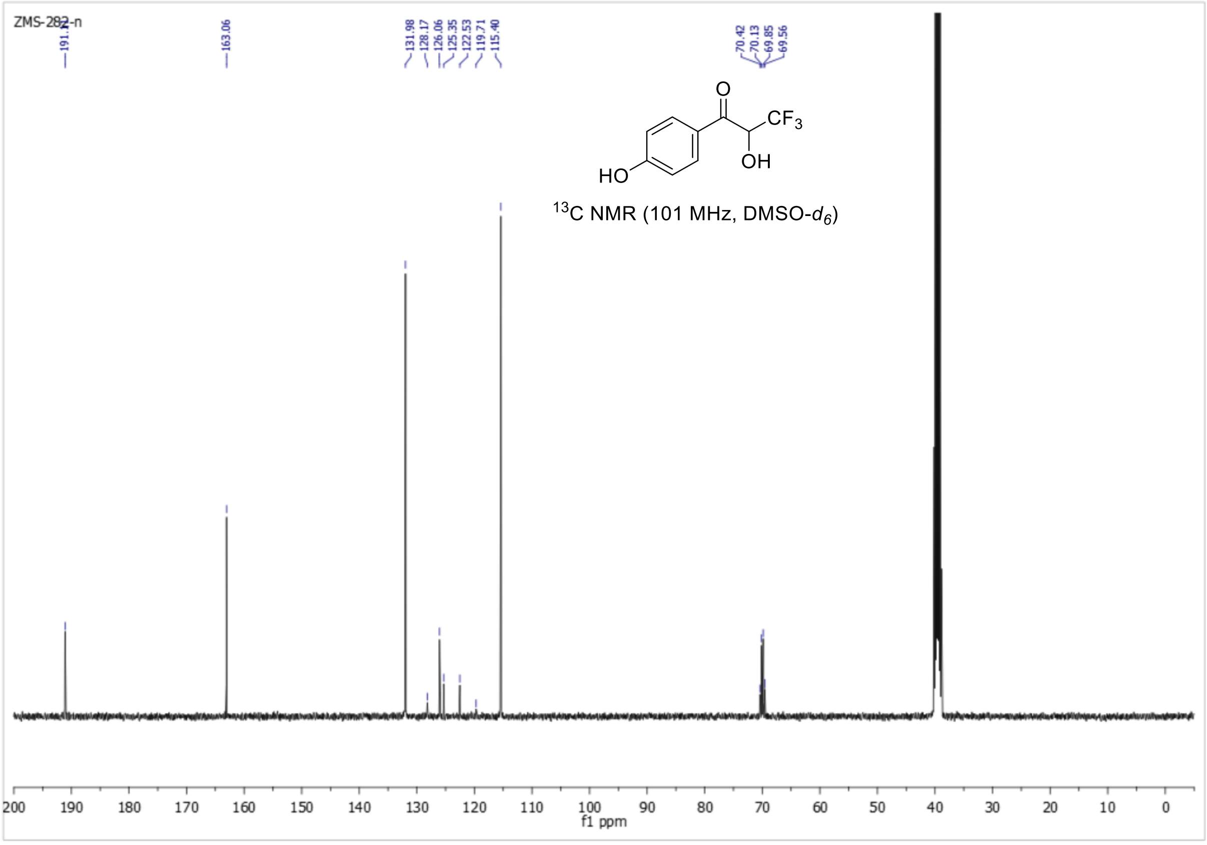

Compound **2m,** ^{1}H NMR:

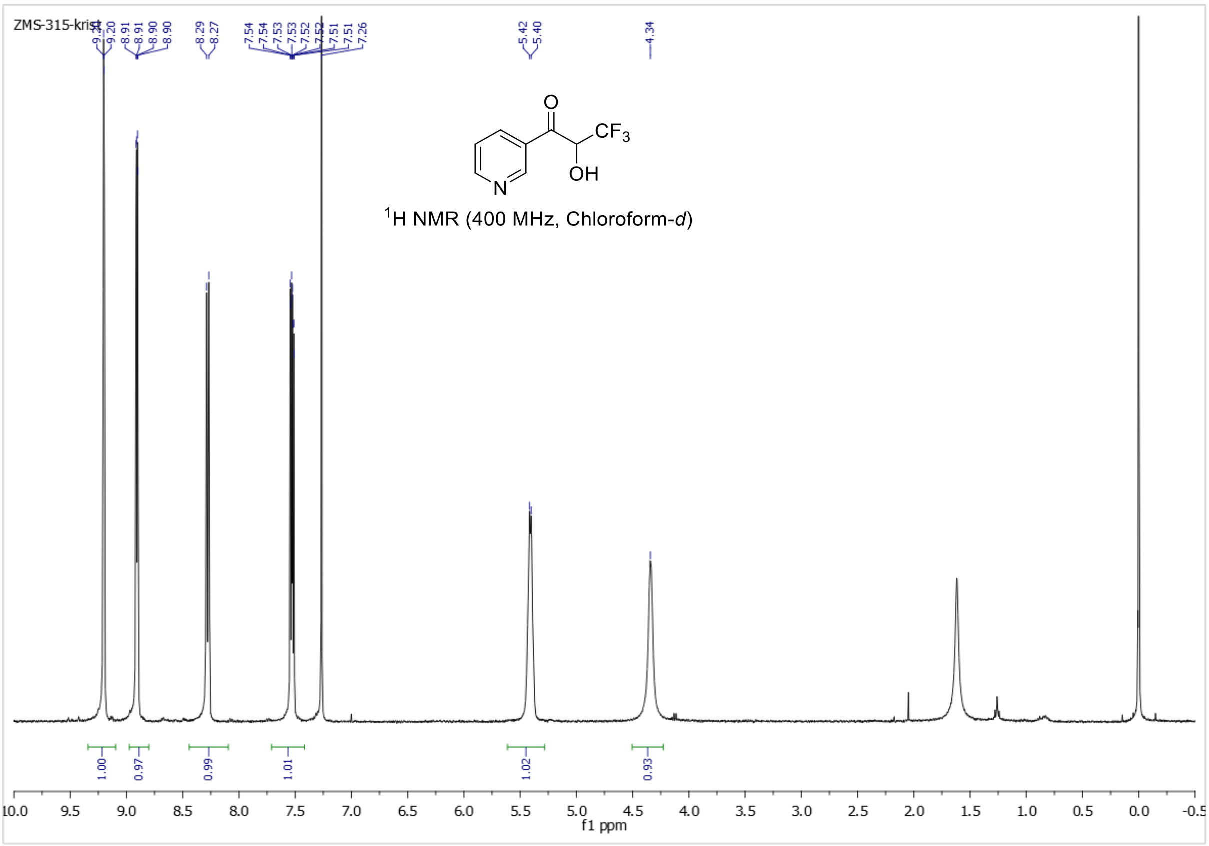

Compound **2m**, ^{19}F NMR:

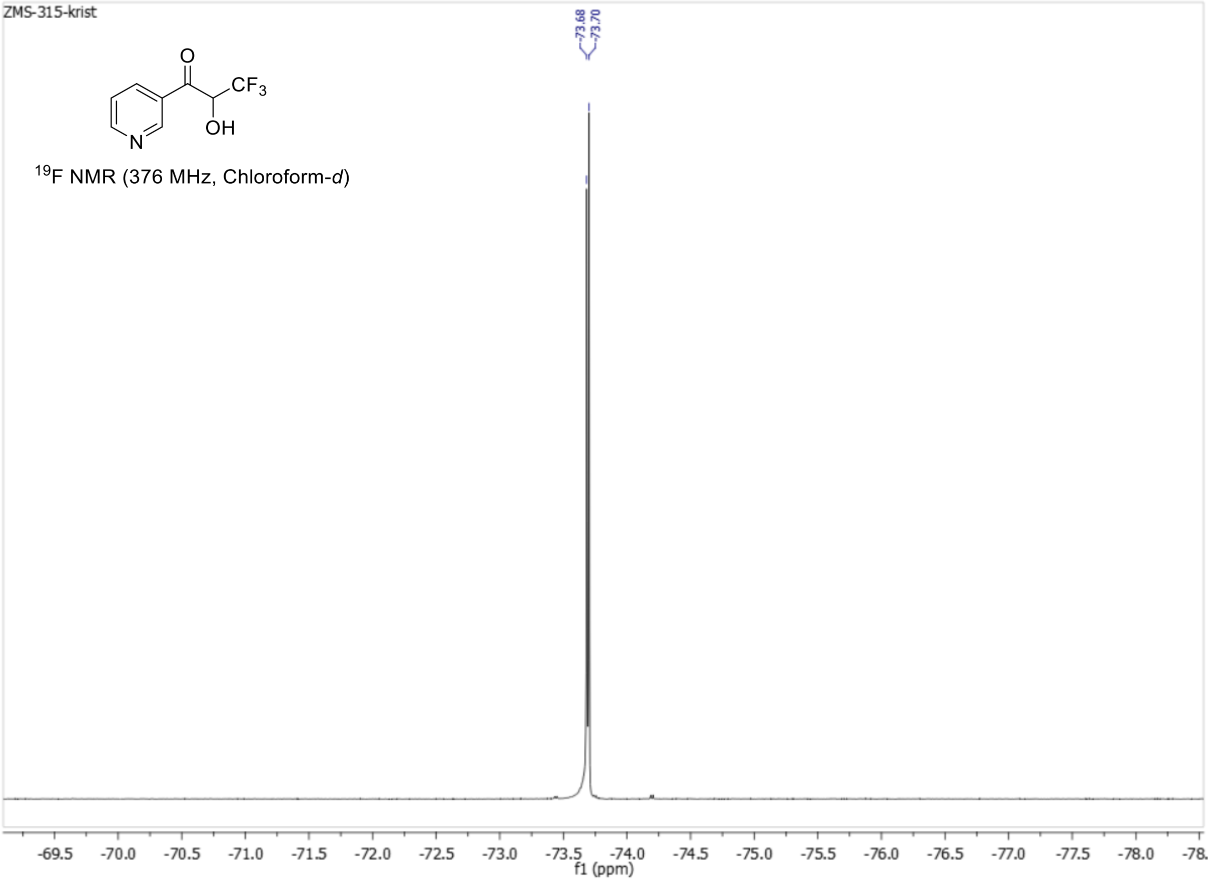

Compound **2m**, ^{13}C NMR:

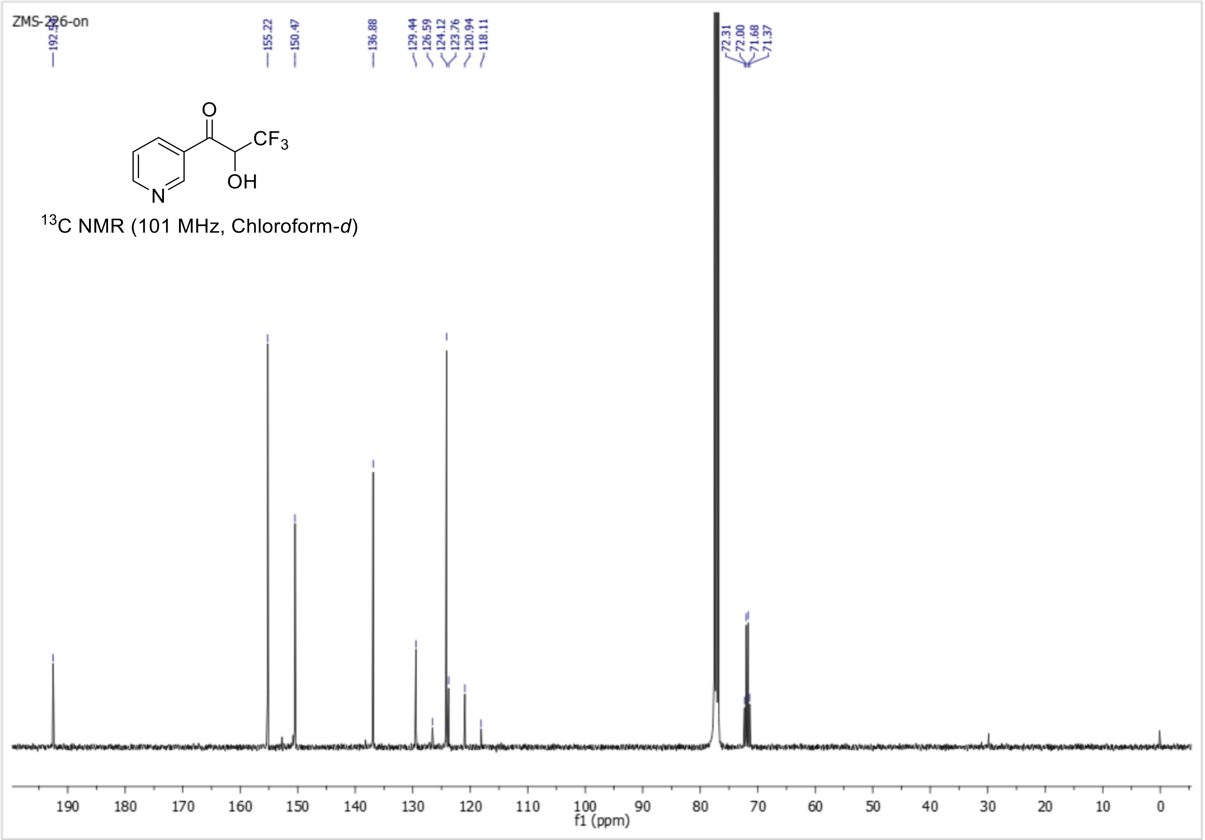

Compound **2n,** ^{1}H NMR:

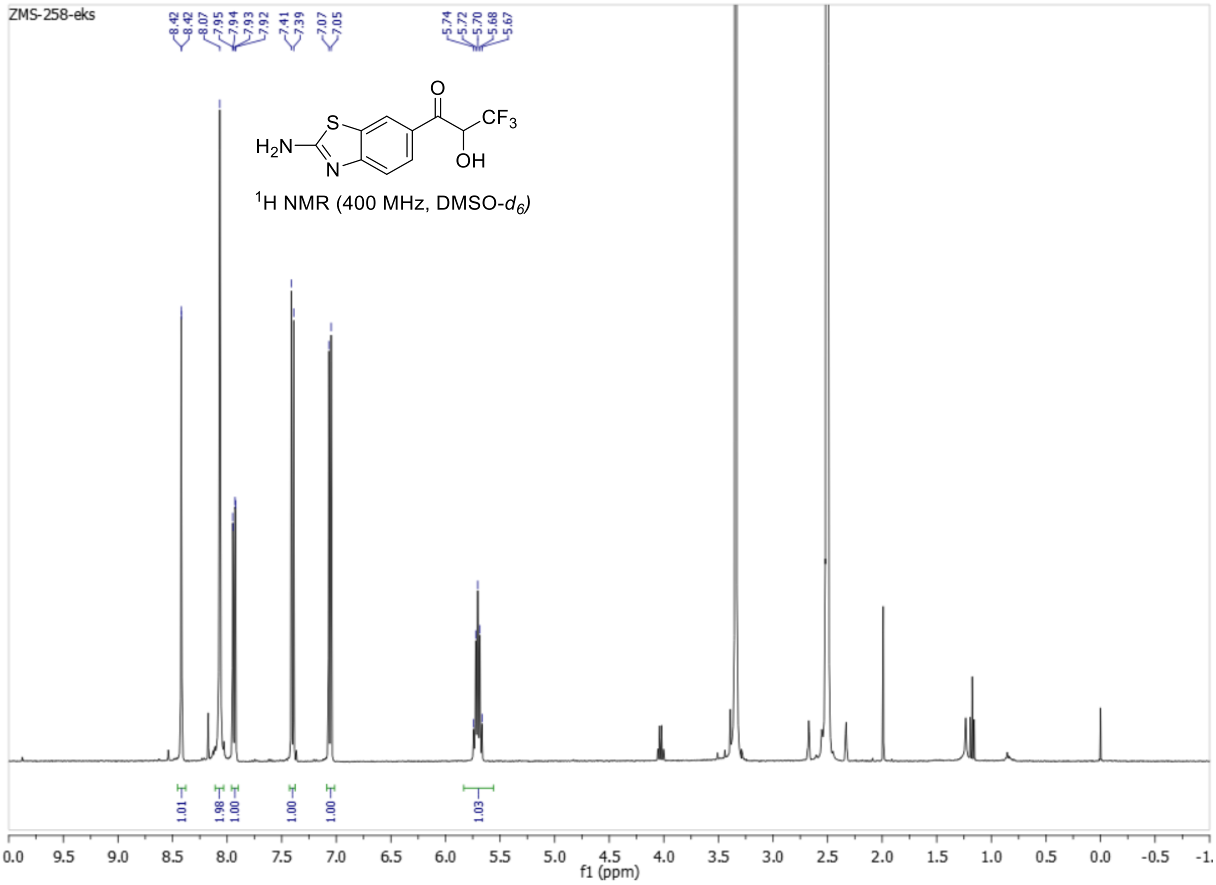

Compound **2n,** ^{19}F NMR:

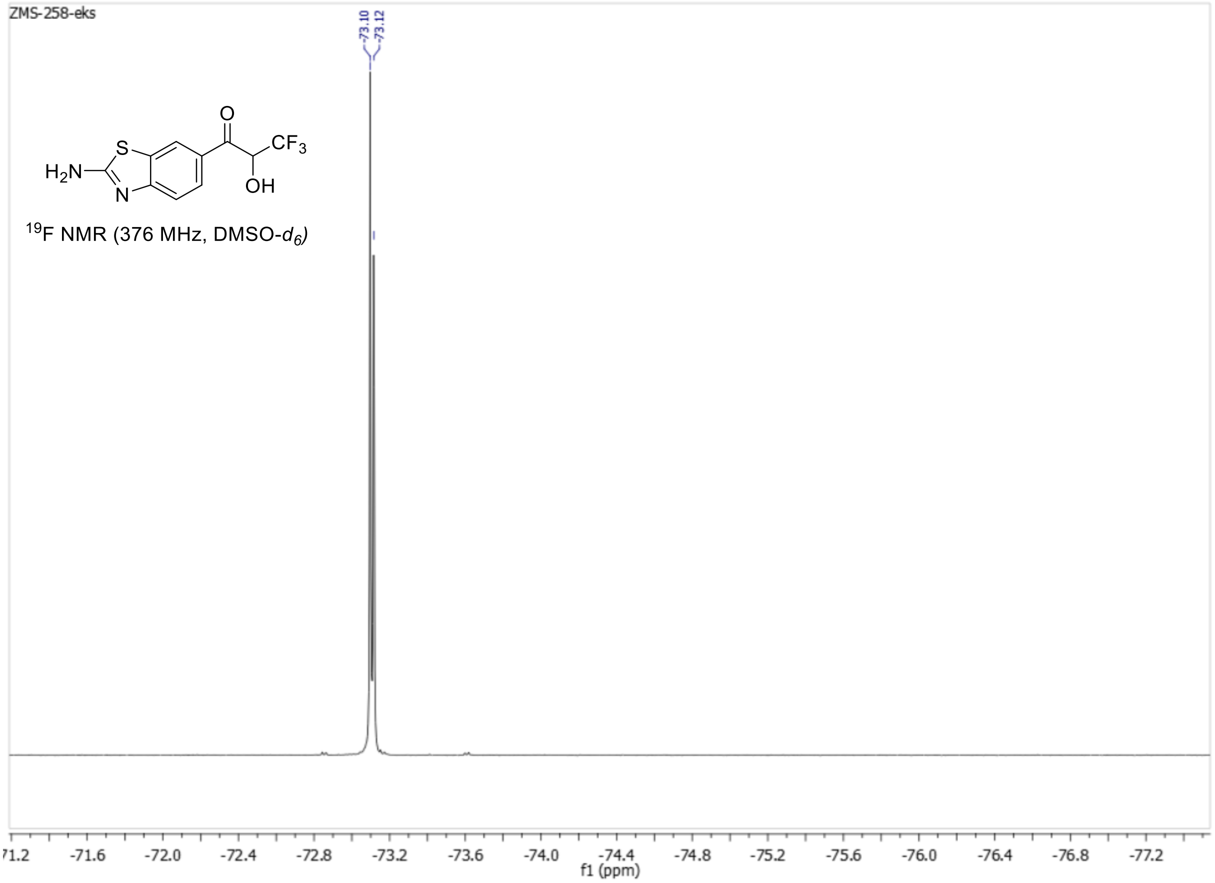

Compound **2n**, ^{13}C NMR:

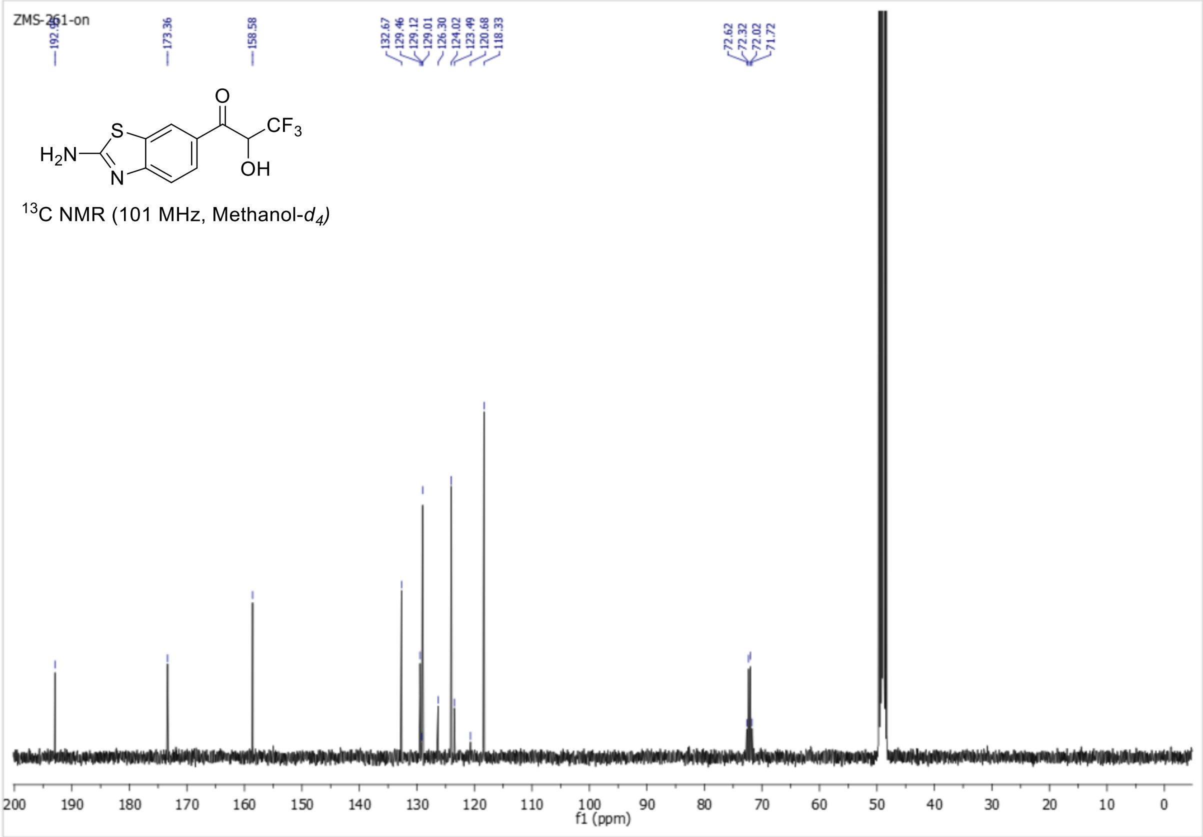

Compound **2o**, ^{1}H NMR:

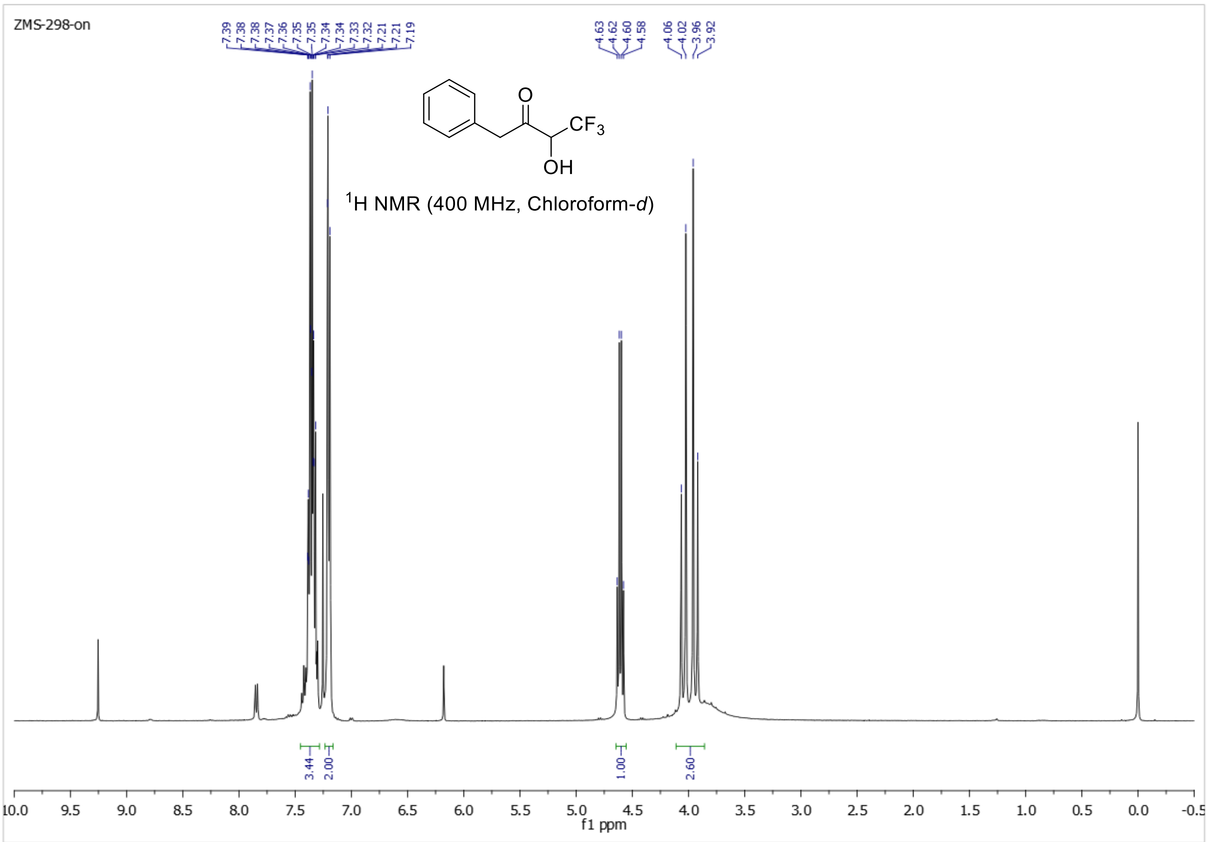

Compound **2o**, ^{19}F NMR:

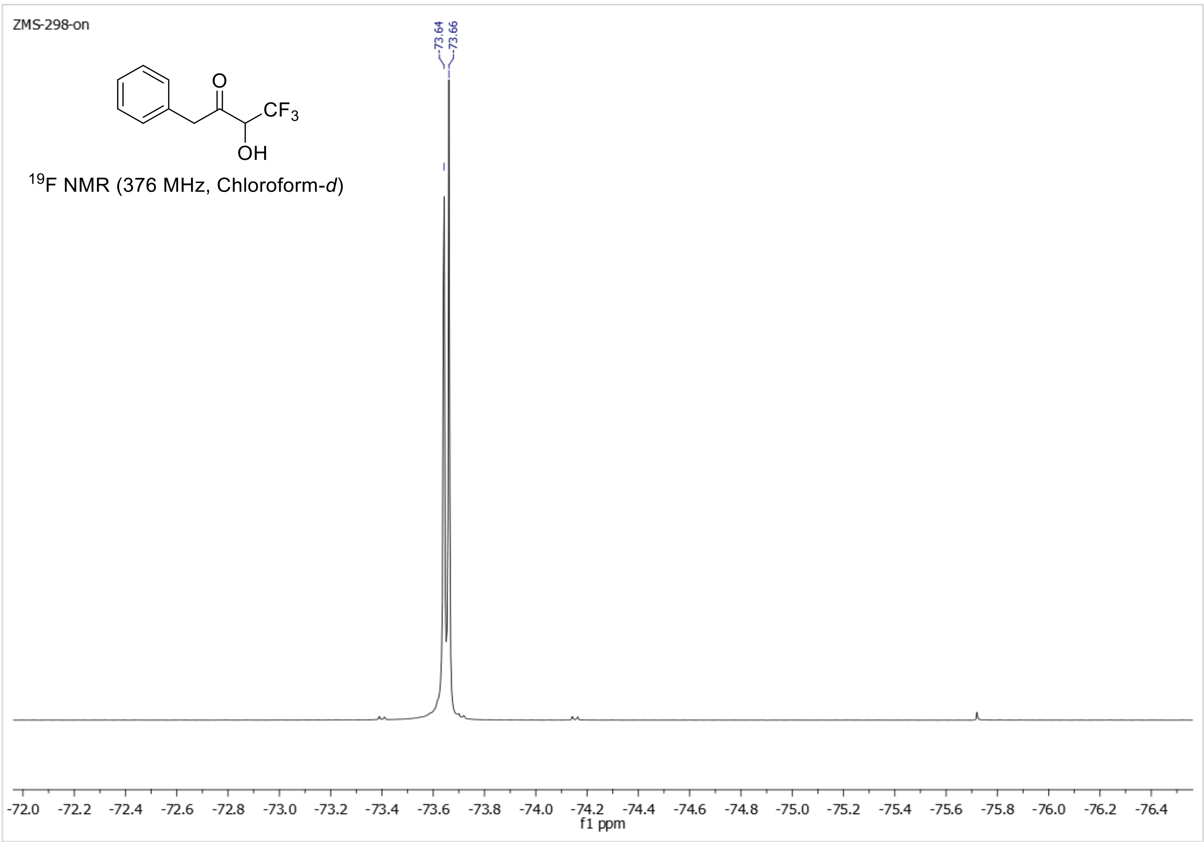

Compound **2o**, ^{13}C NMR:

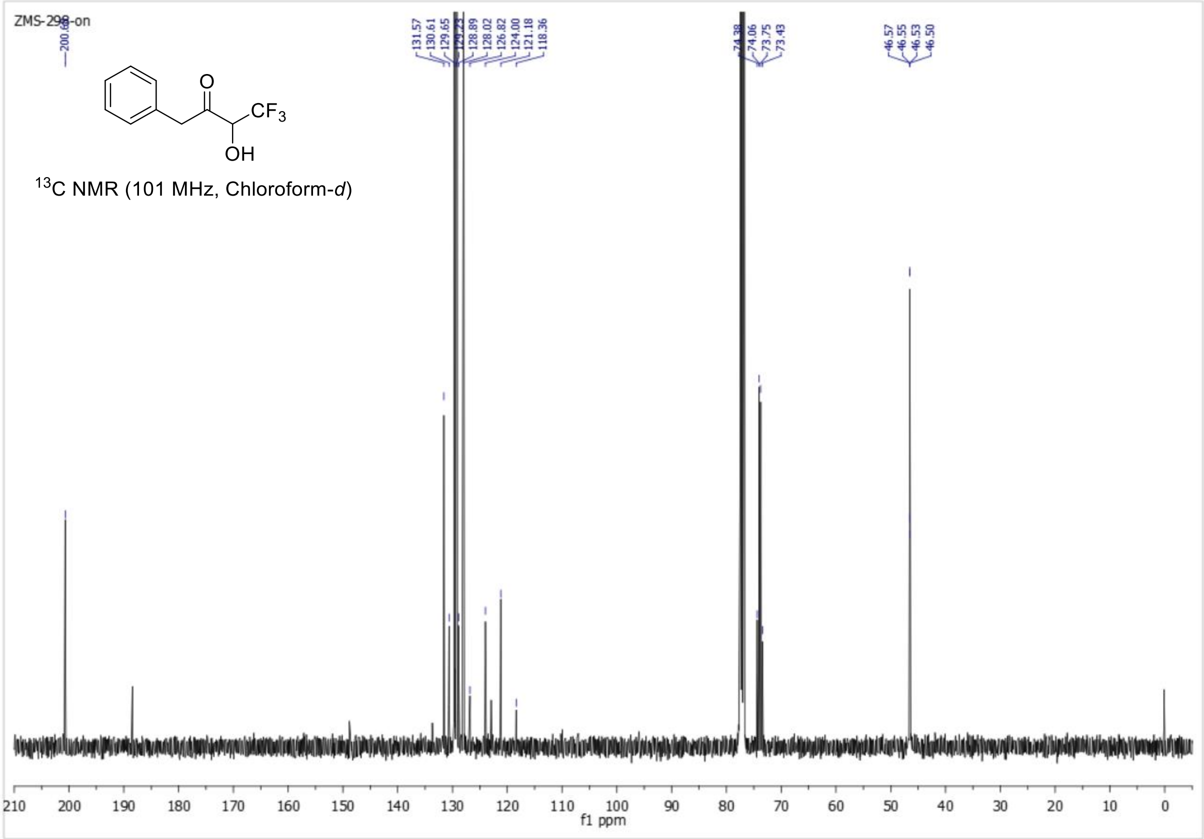

Compound **2p**, ^{1}H NMR:

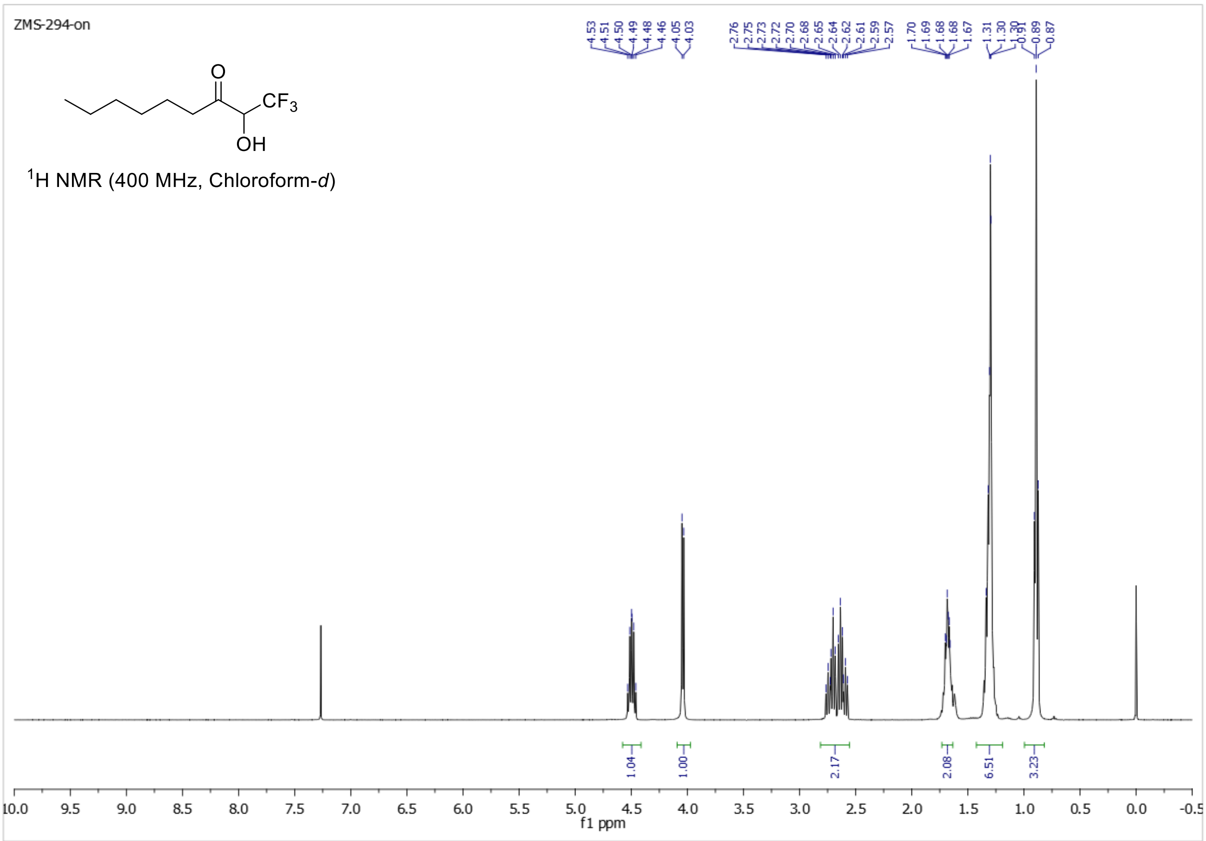

Compound **2p**, ^{19}F NMR:

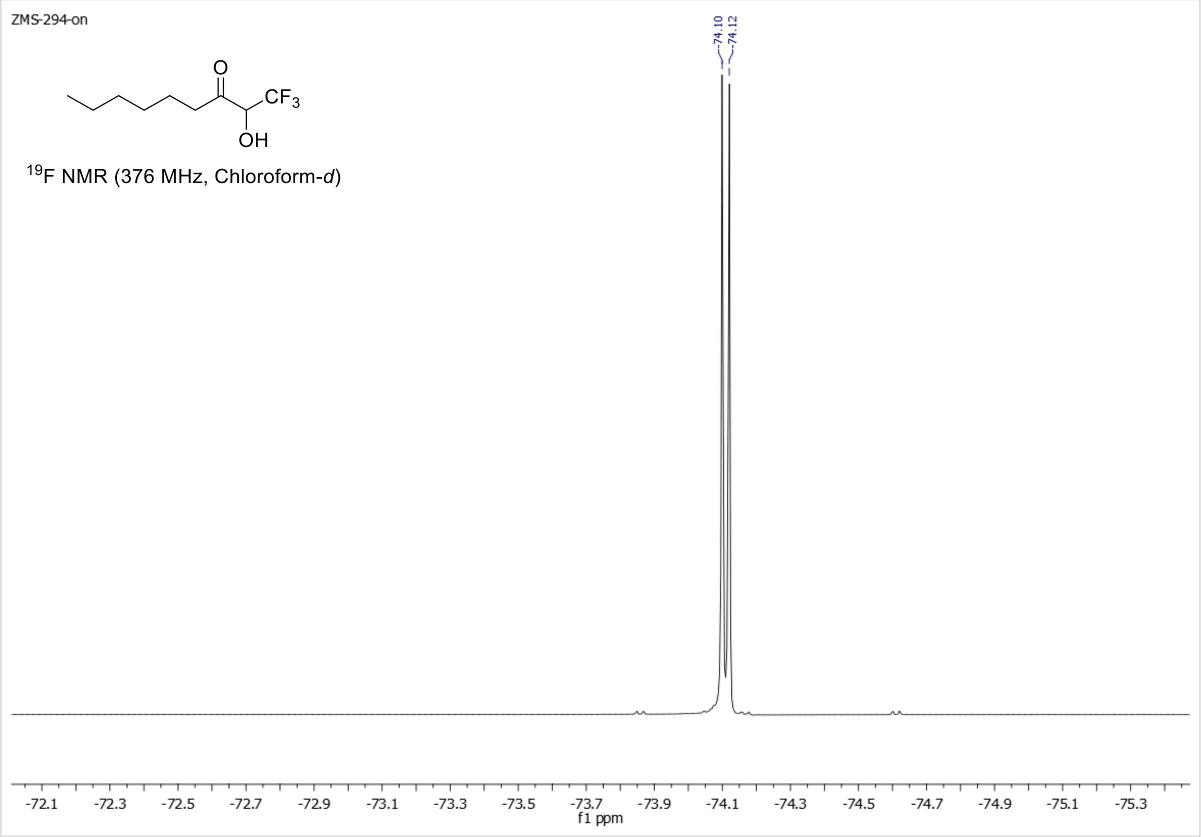

Compound **2p**, ^{13}C NMR:

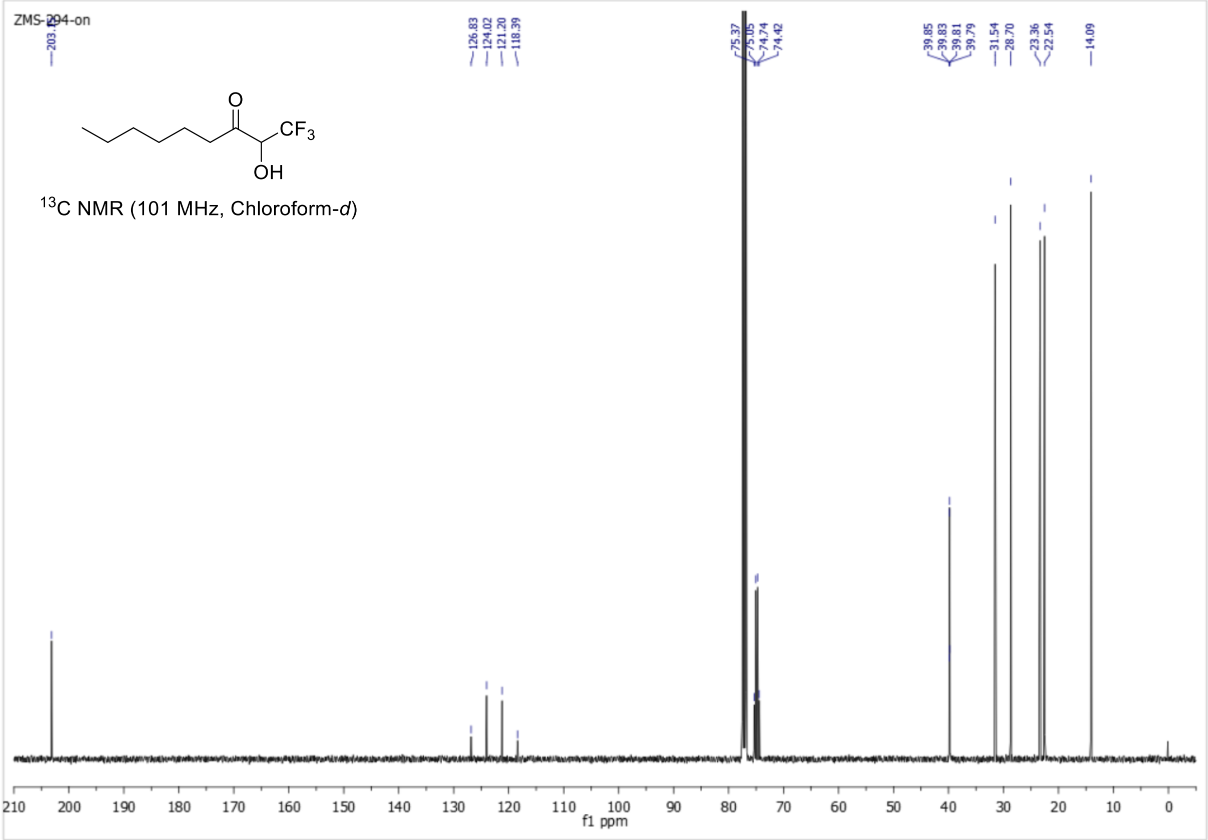

Compound **2q**, ^{1}H NMR:

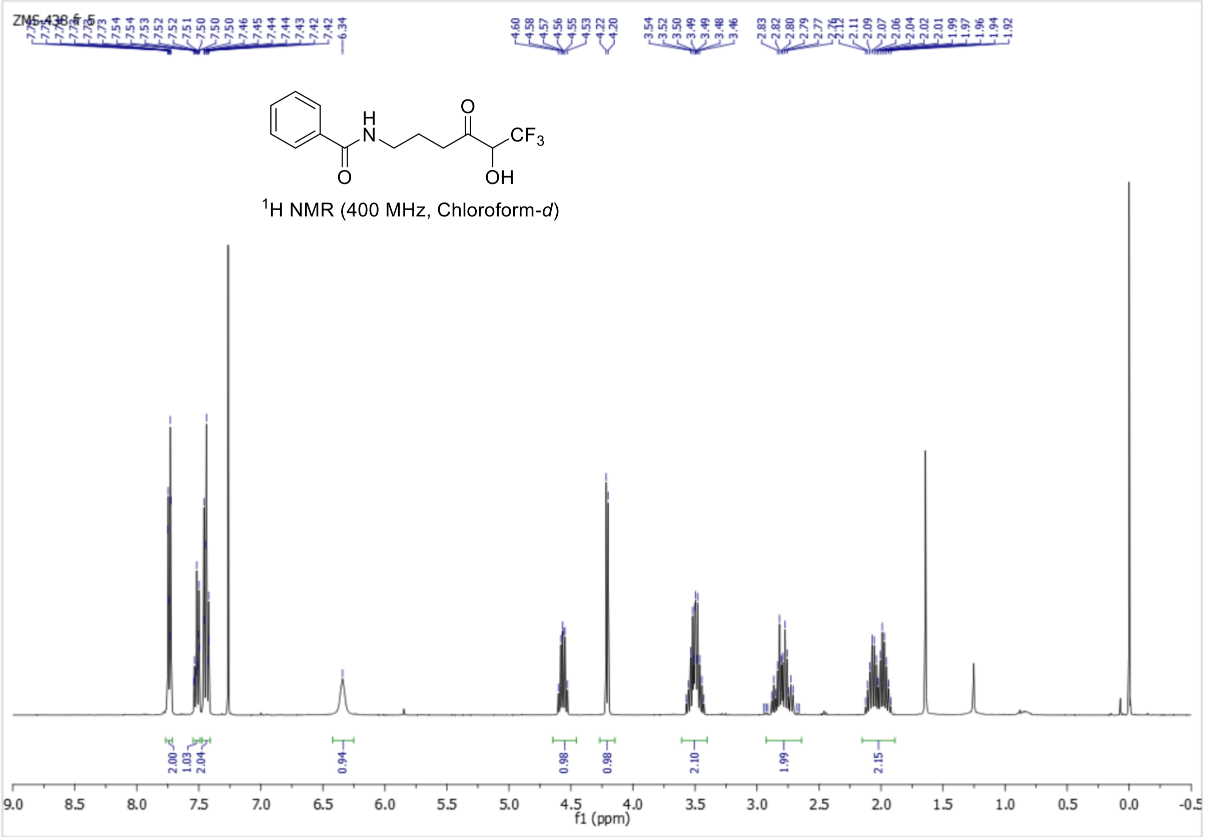

Compound **2q**, ^{19}F NMR:

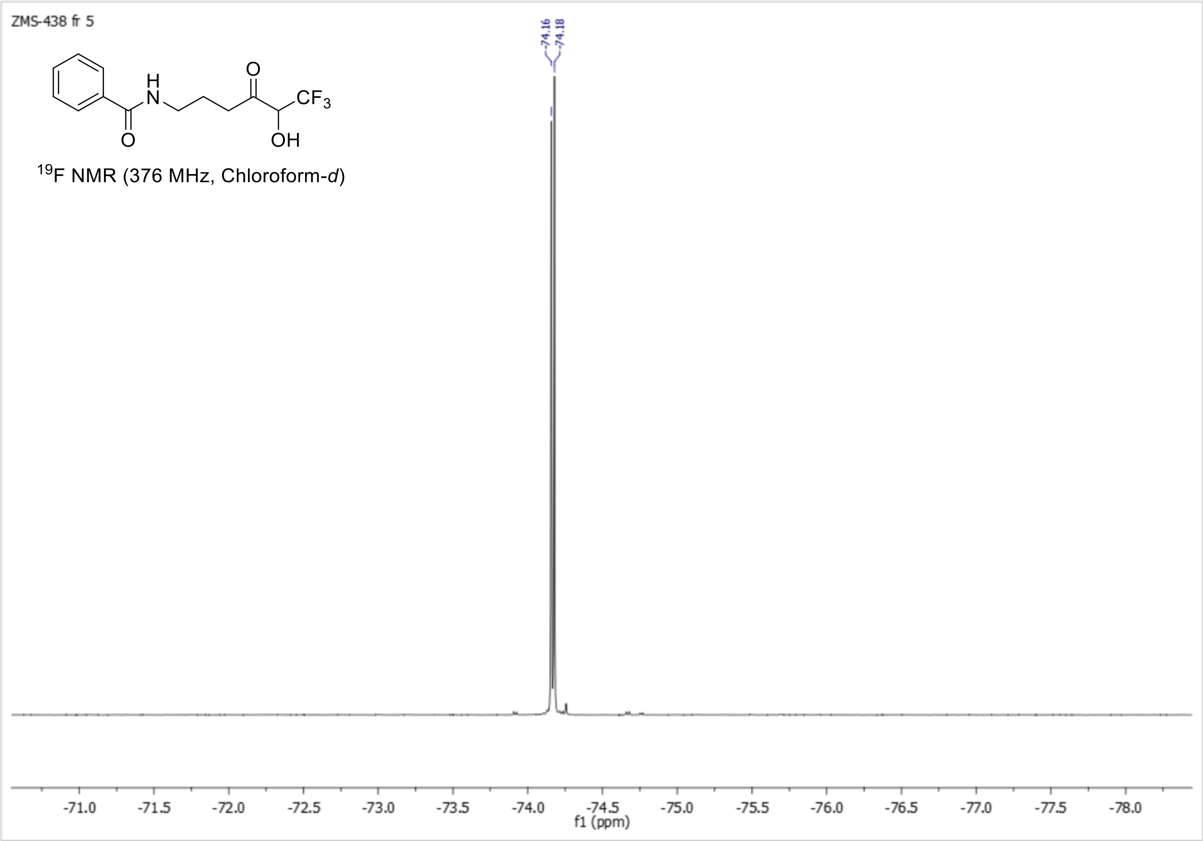

Compound **2q,** ^{13}C NMR:

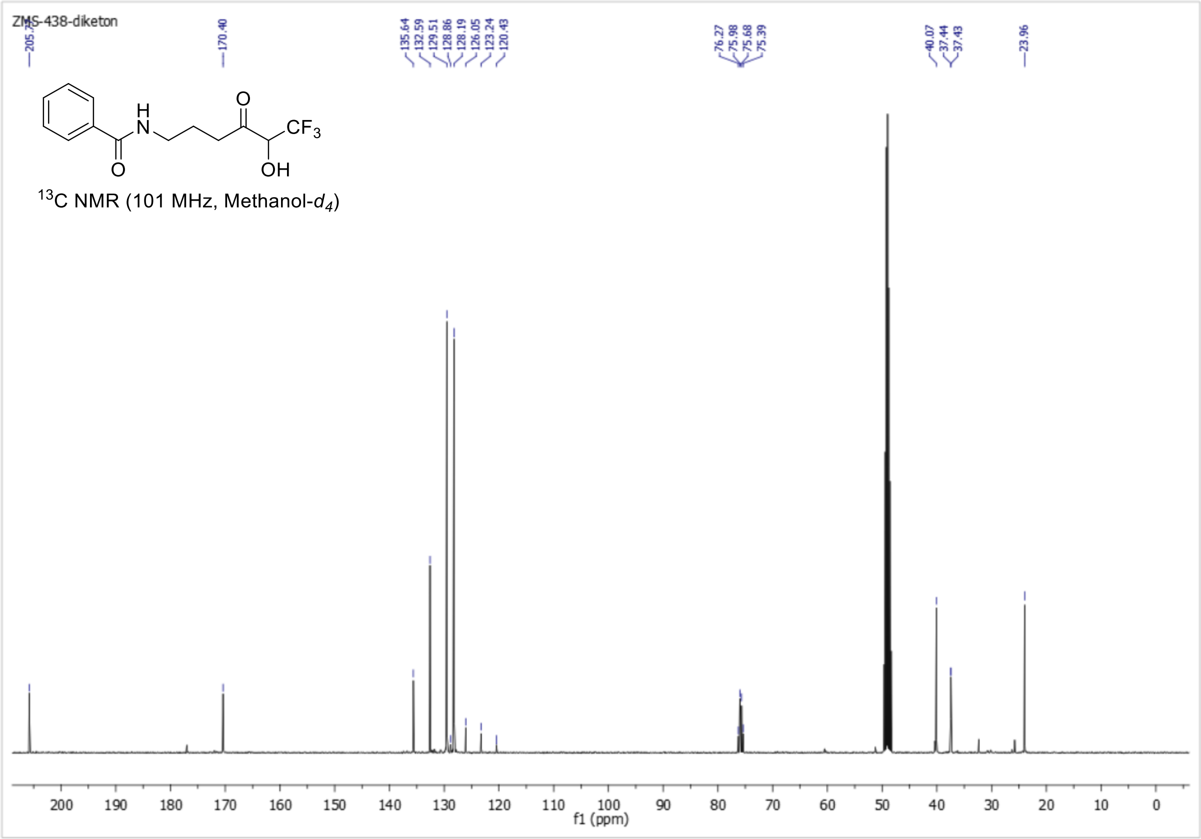

Compound ***syn*-3a**, ^{1}H NMR:

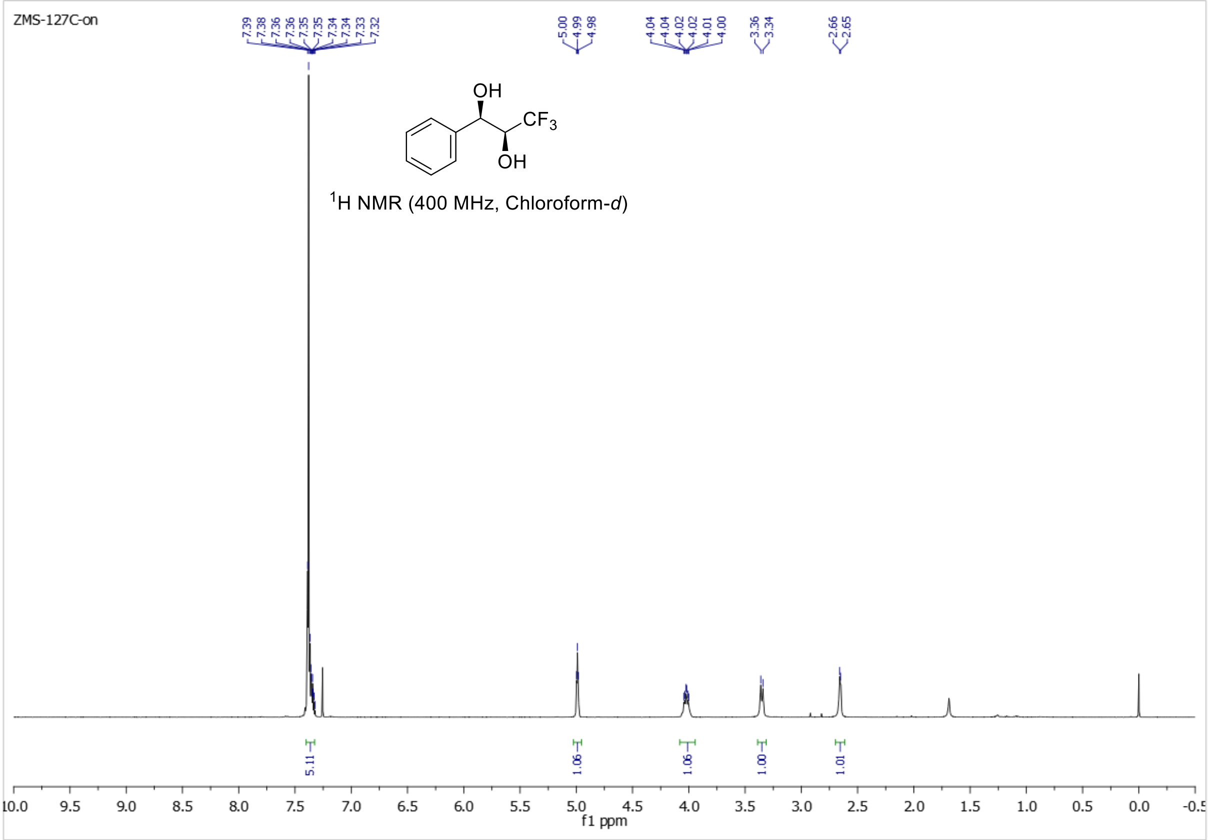

Compound ***syn*-3a**, ^{19}F NMR:

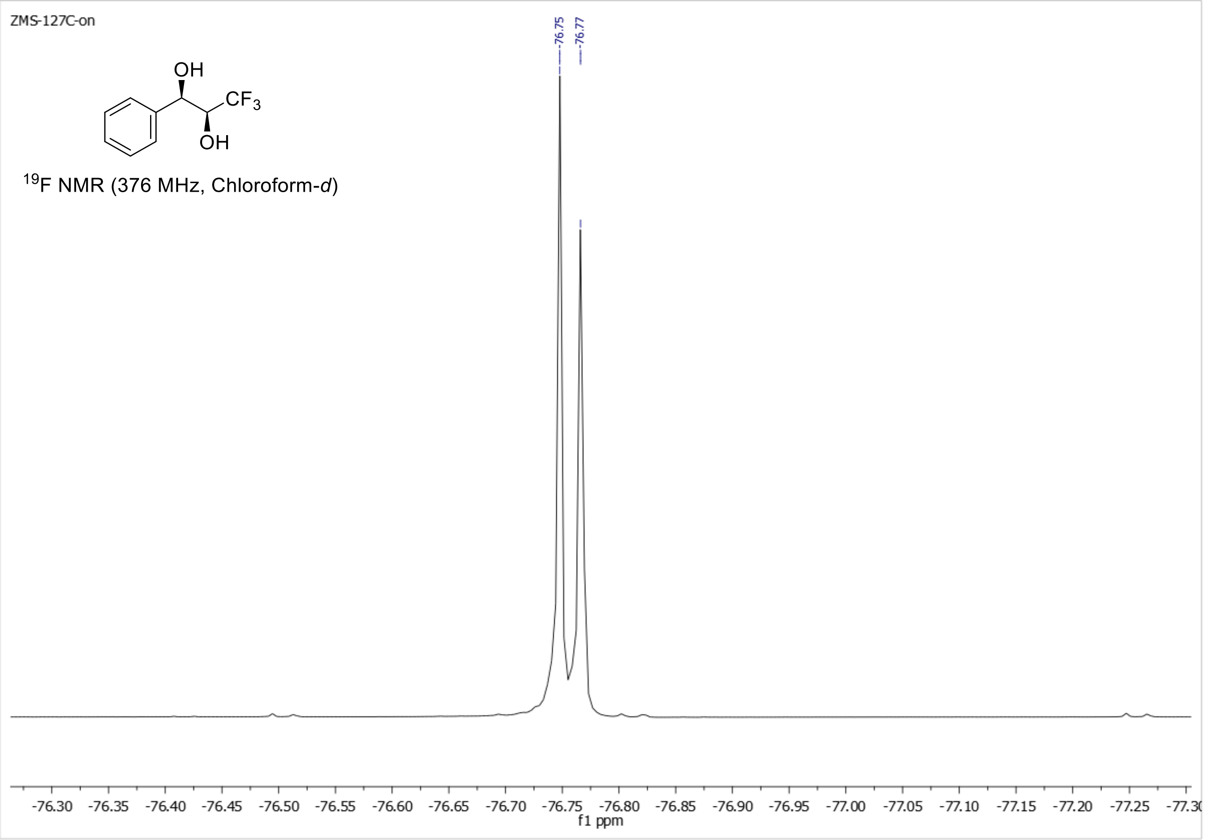

Compound ***syn*-3a**, ^{13}C NMR:

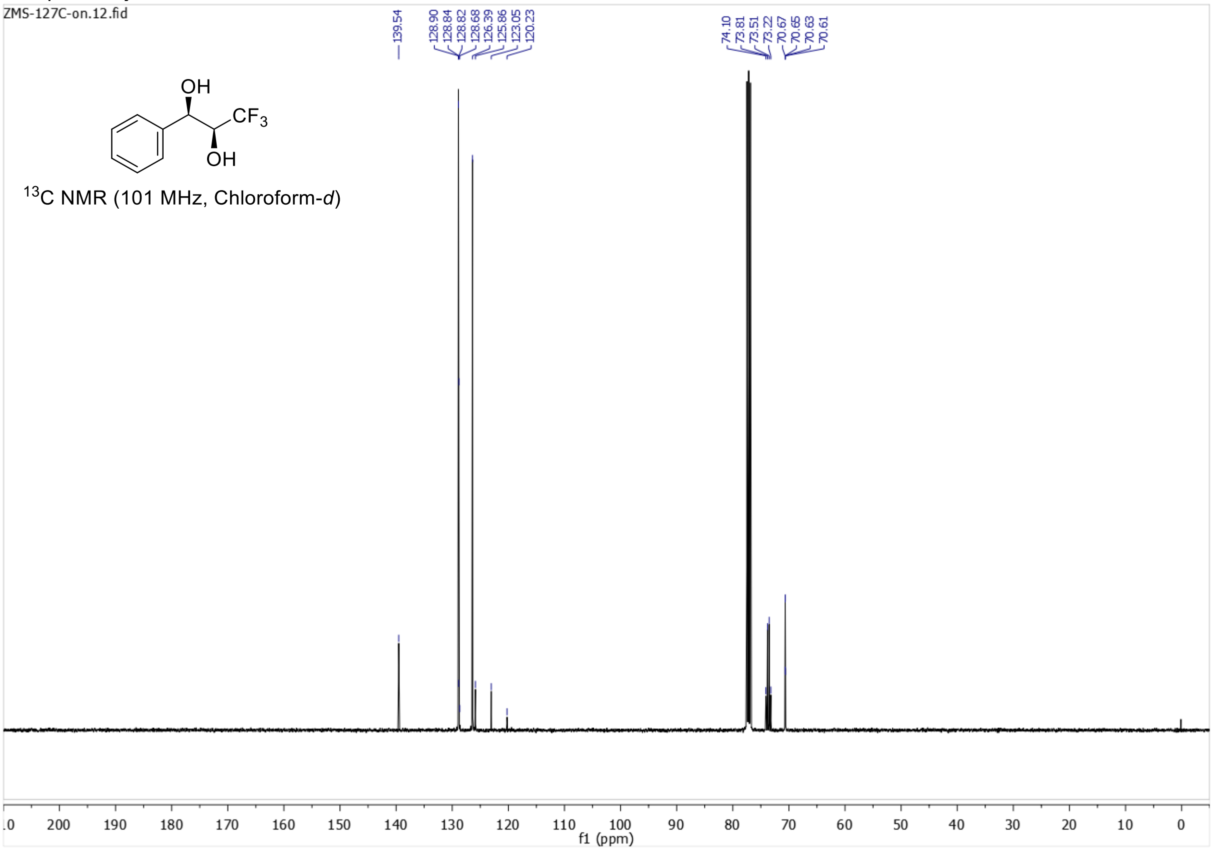

Compound **(±)-*anti*-3a**, ^{1}H NMR:

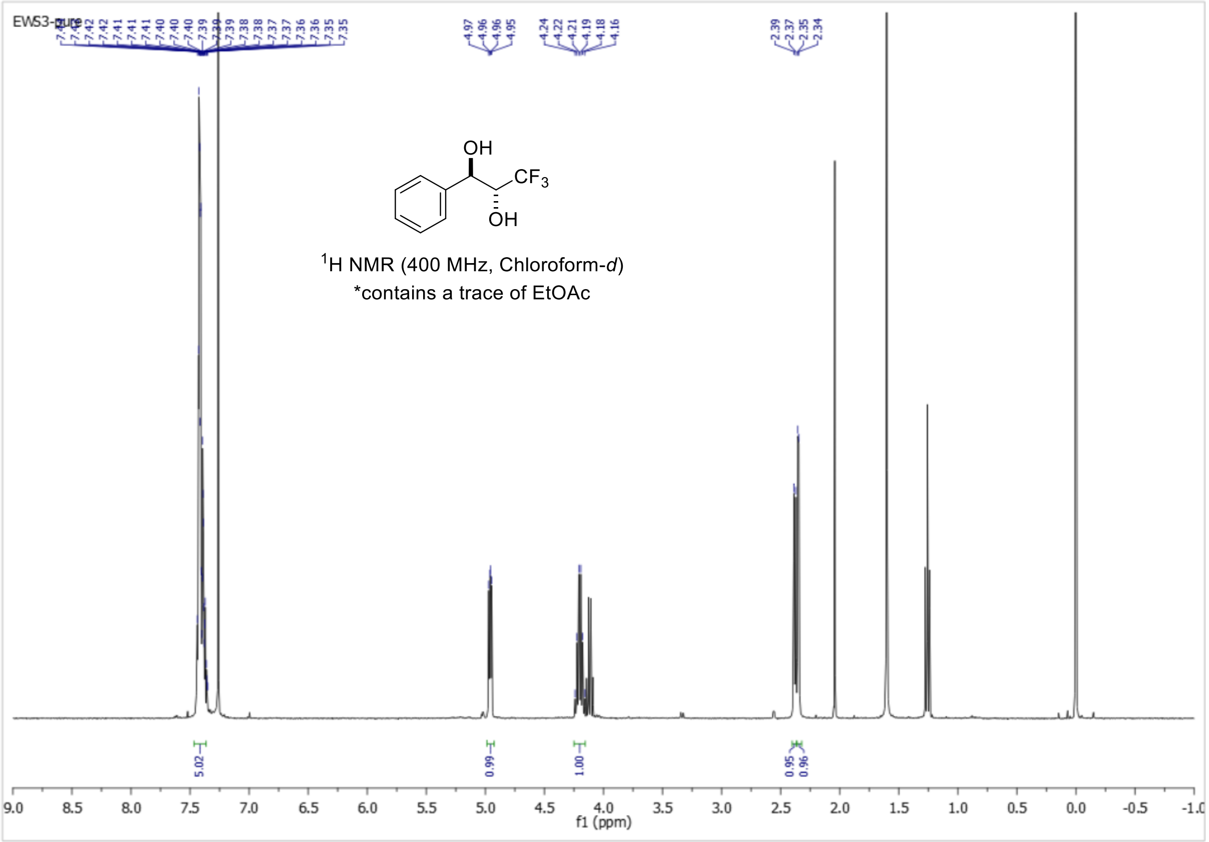

Compound **(±)-*anti*-3a**, ^{19}F NMR:

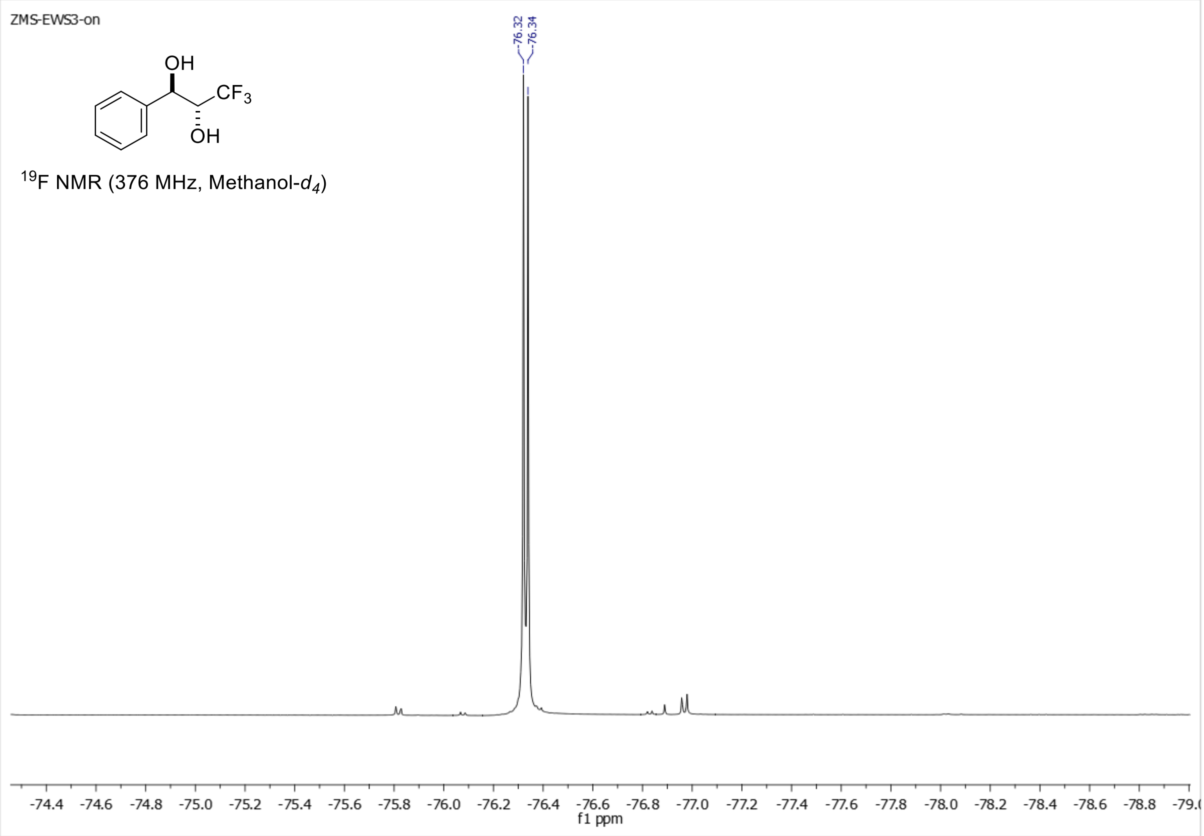

Compound **(±)-*anti*-3a**, ^{13}C NMR:

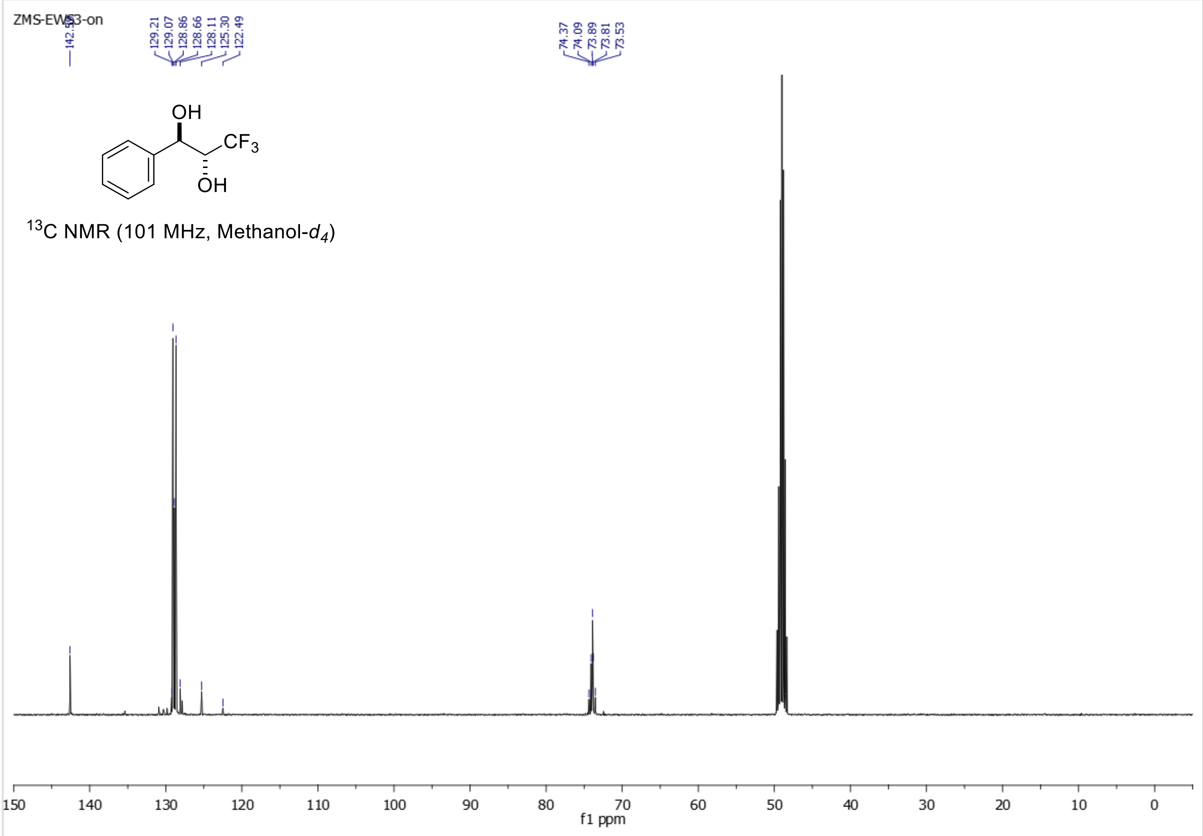

Compound ***syn*-3b**, ^1H NMR:

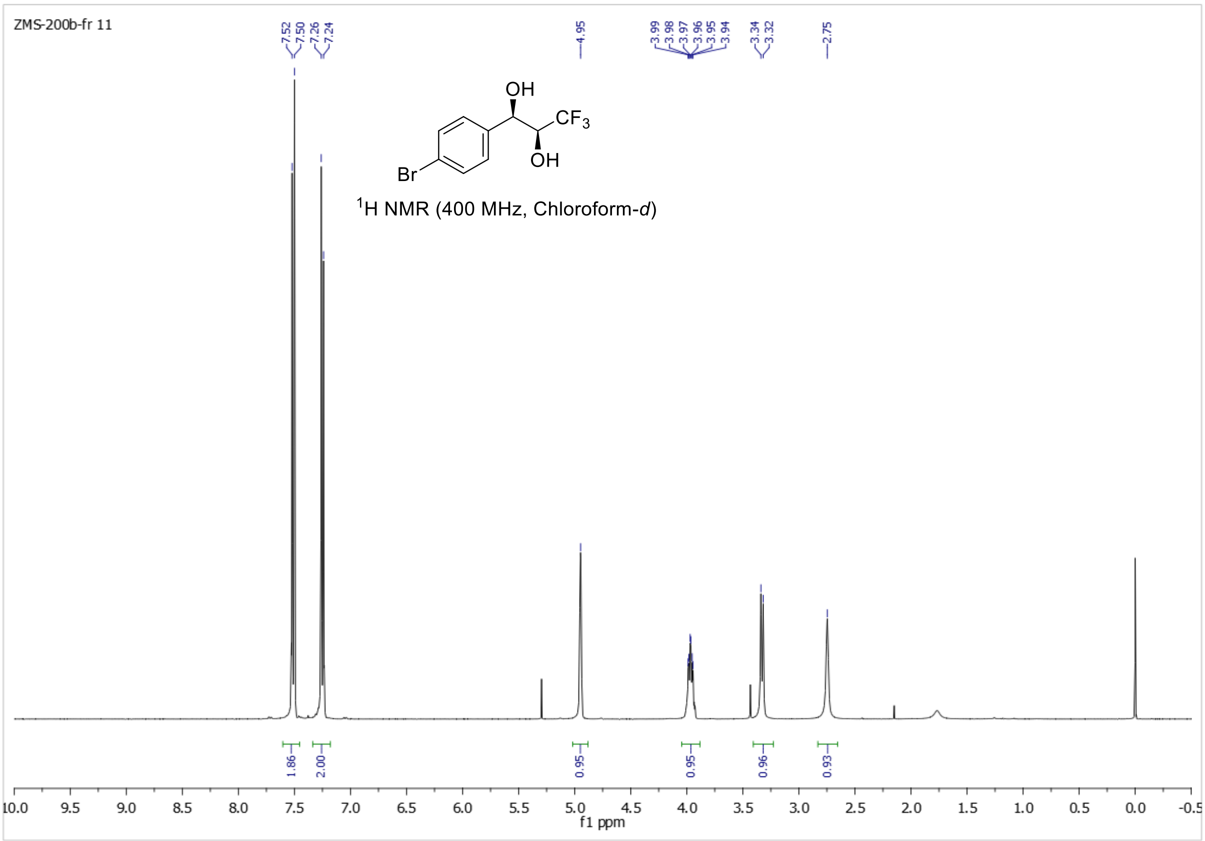

Compound ***syn*-3b**, ^{19}F NMR:

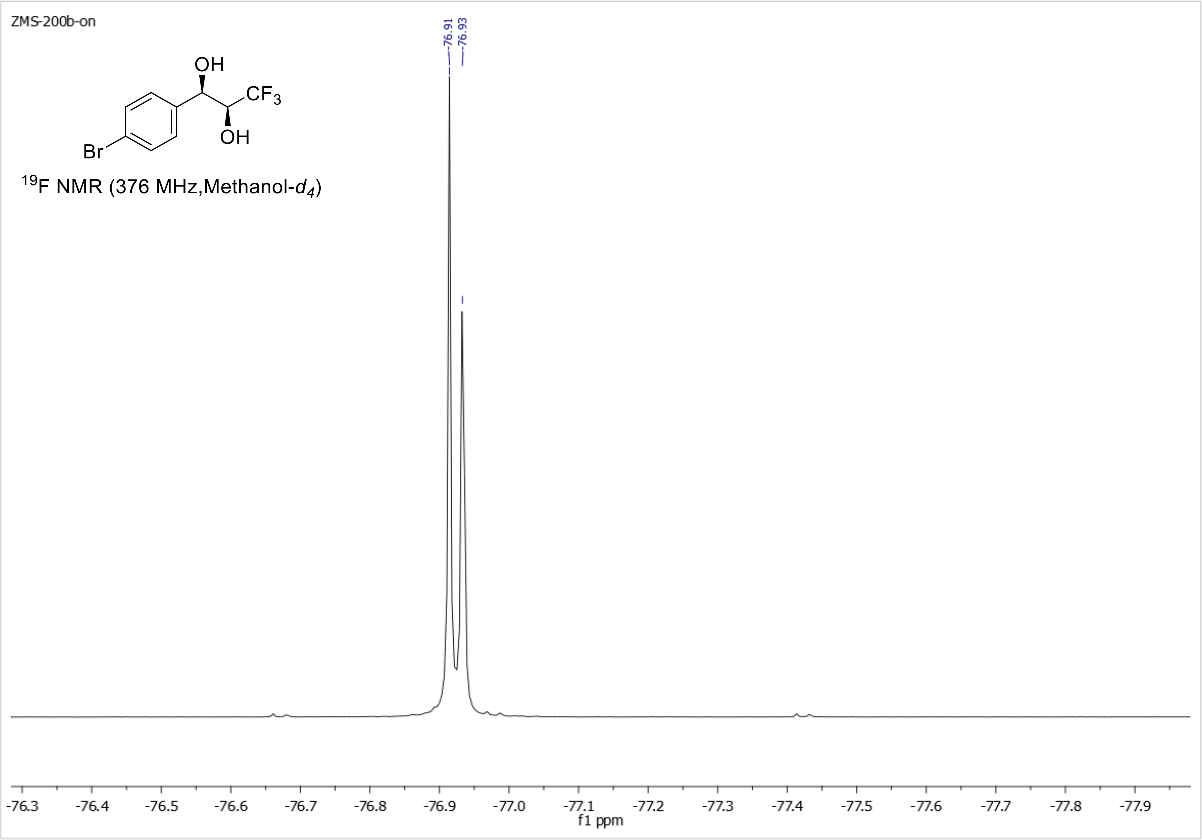

Compound ***syn*-3b**, ^{13}C NMR:

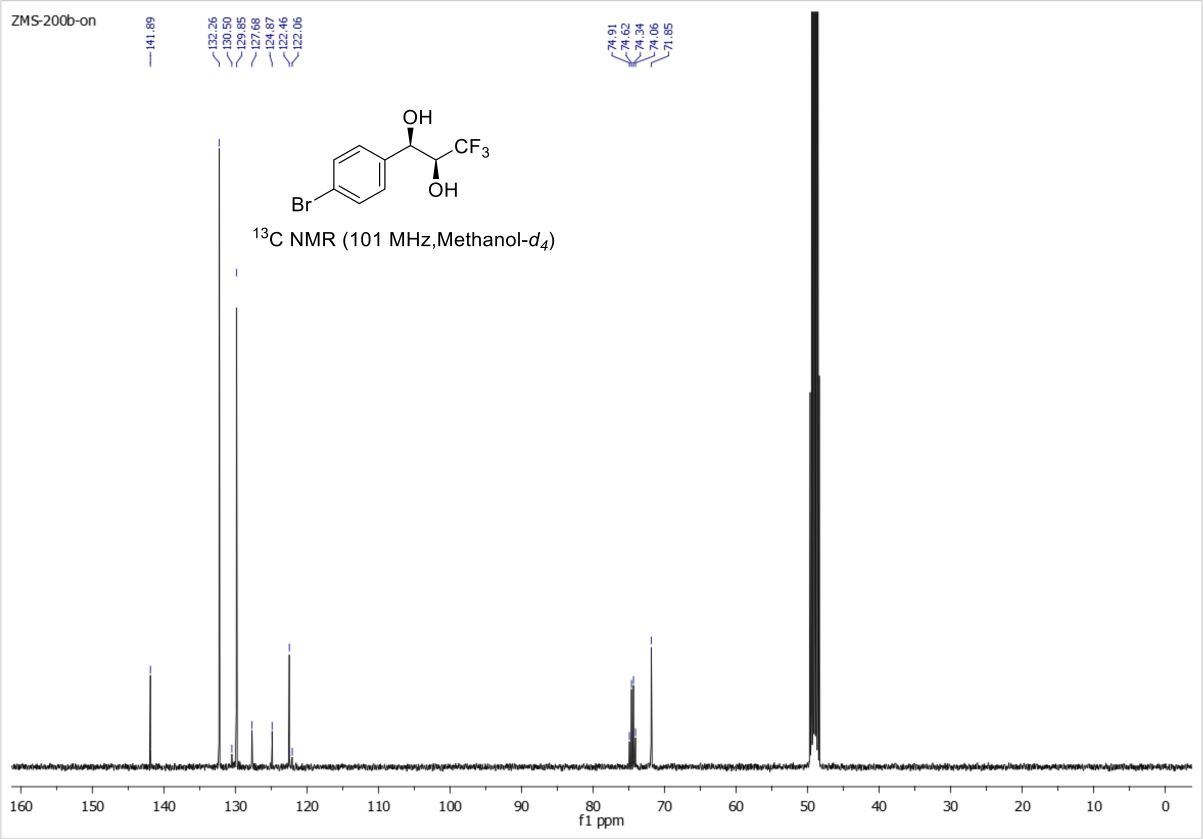

Compound **(±)-*anti*-3b**, ^{1}H NMR:

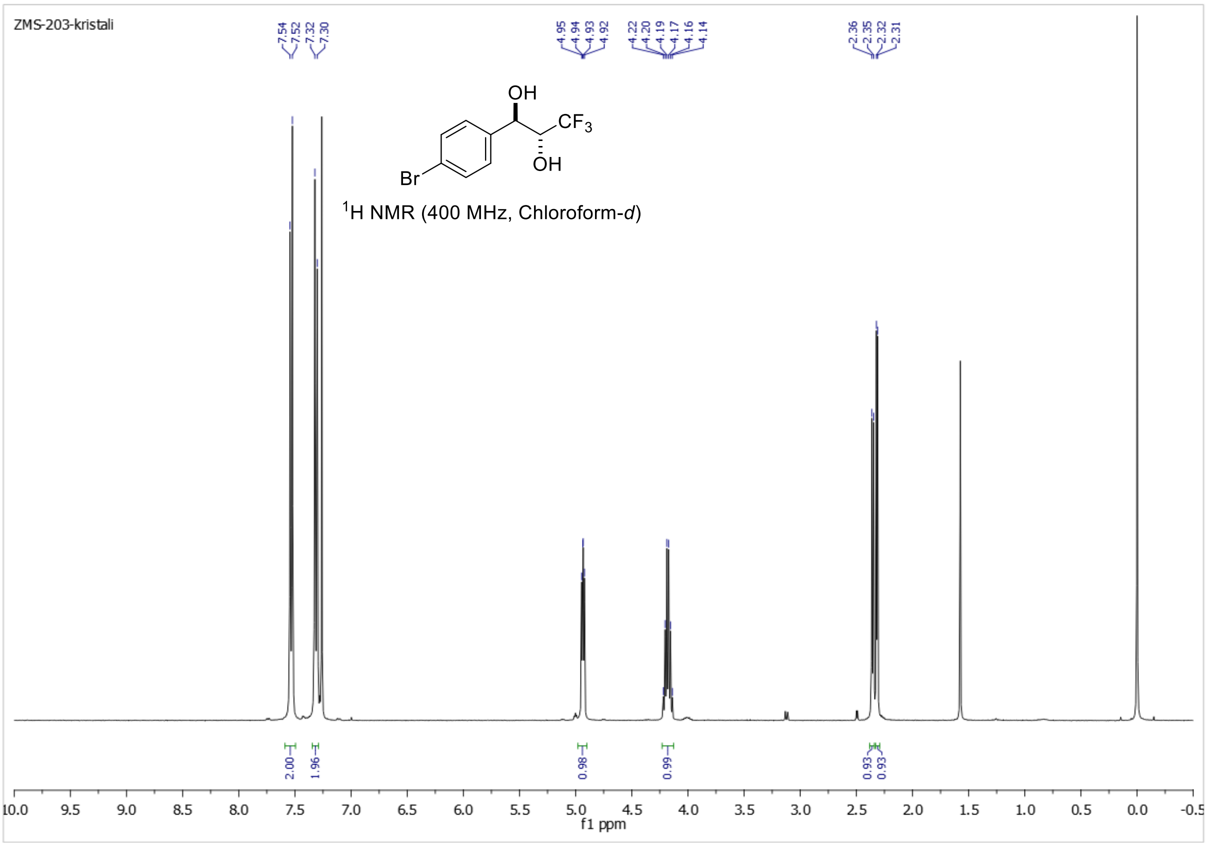

Compound **(±)-*anti*-3b**, ^{19}F NMR:

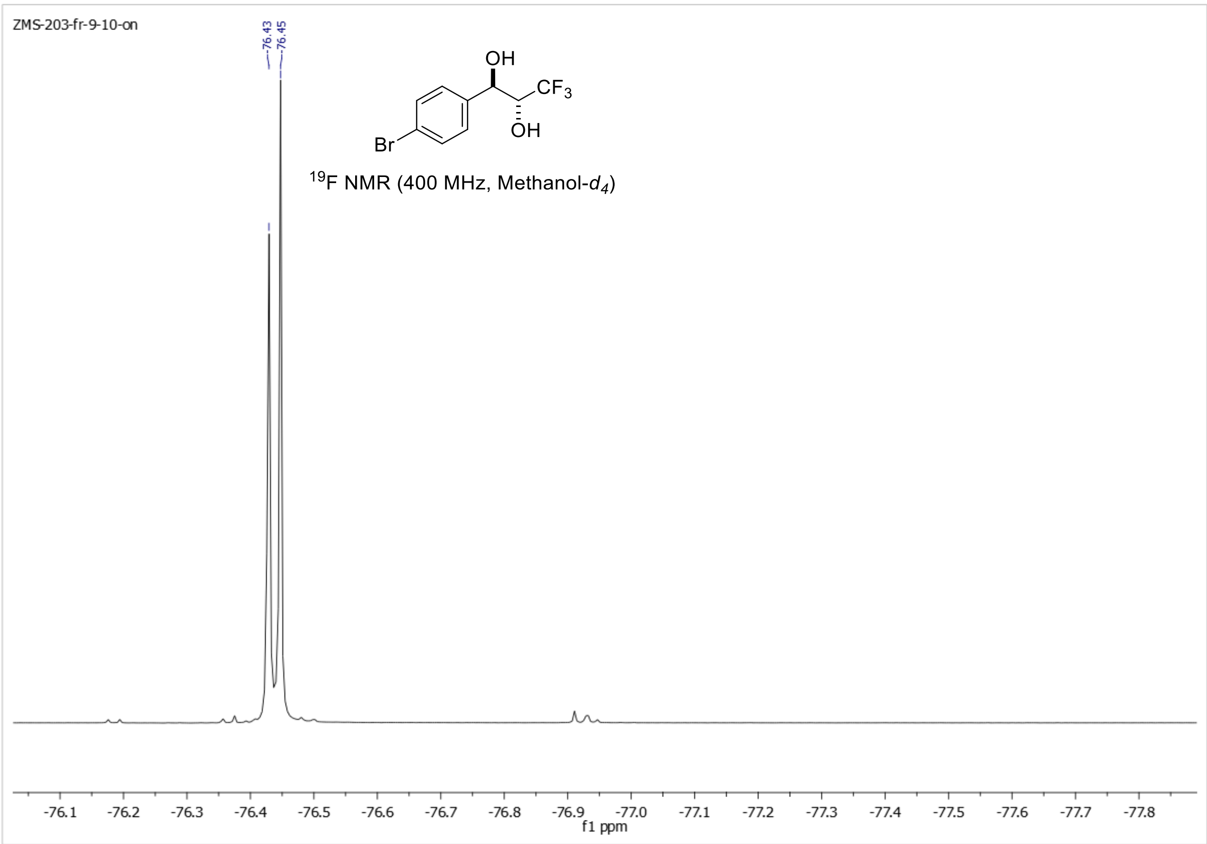

Compound **(±)-*anti*-3b**, ^{13}C NMR:

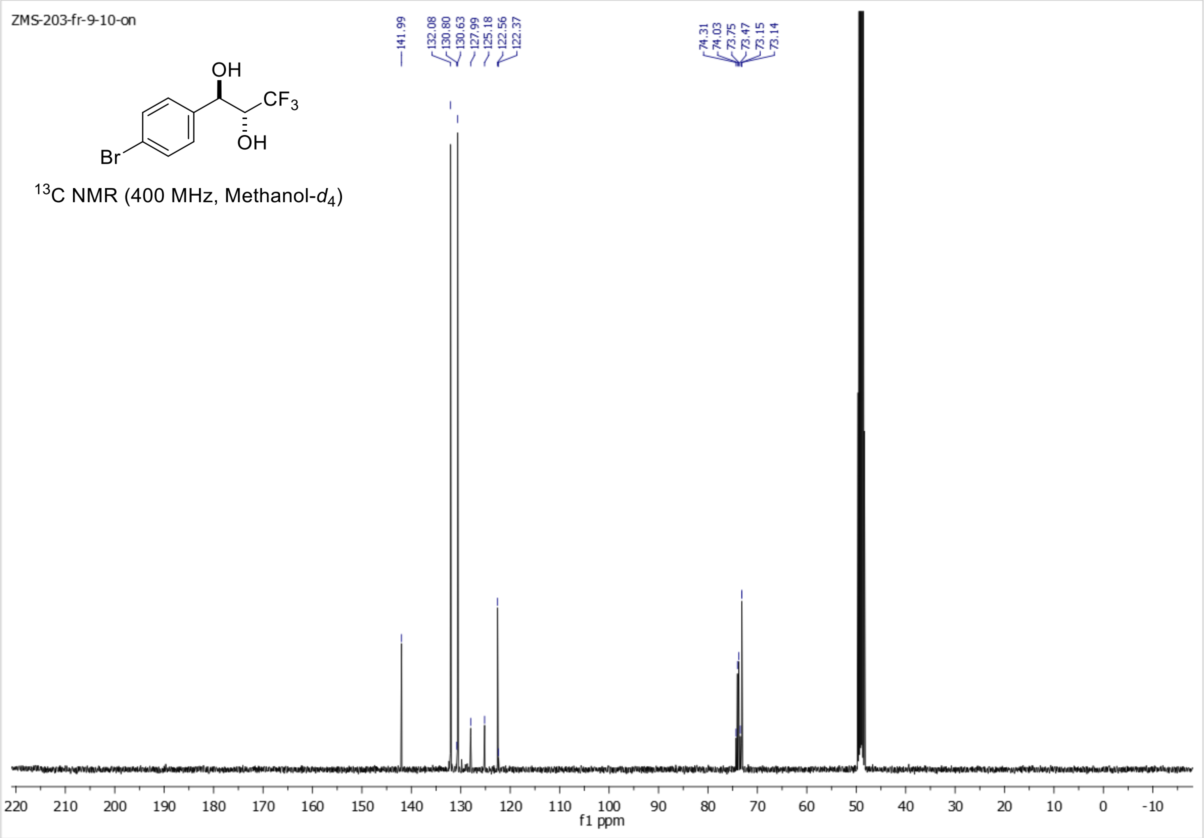

Compound ***syn*-3c**, ^{1}H NMR:

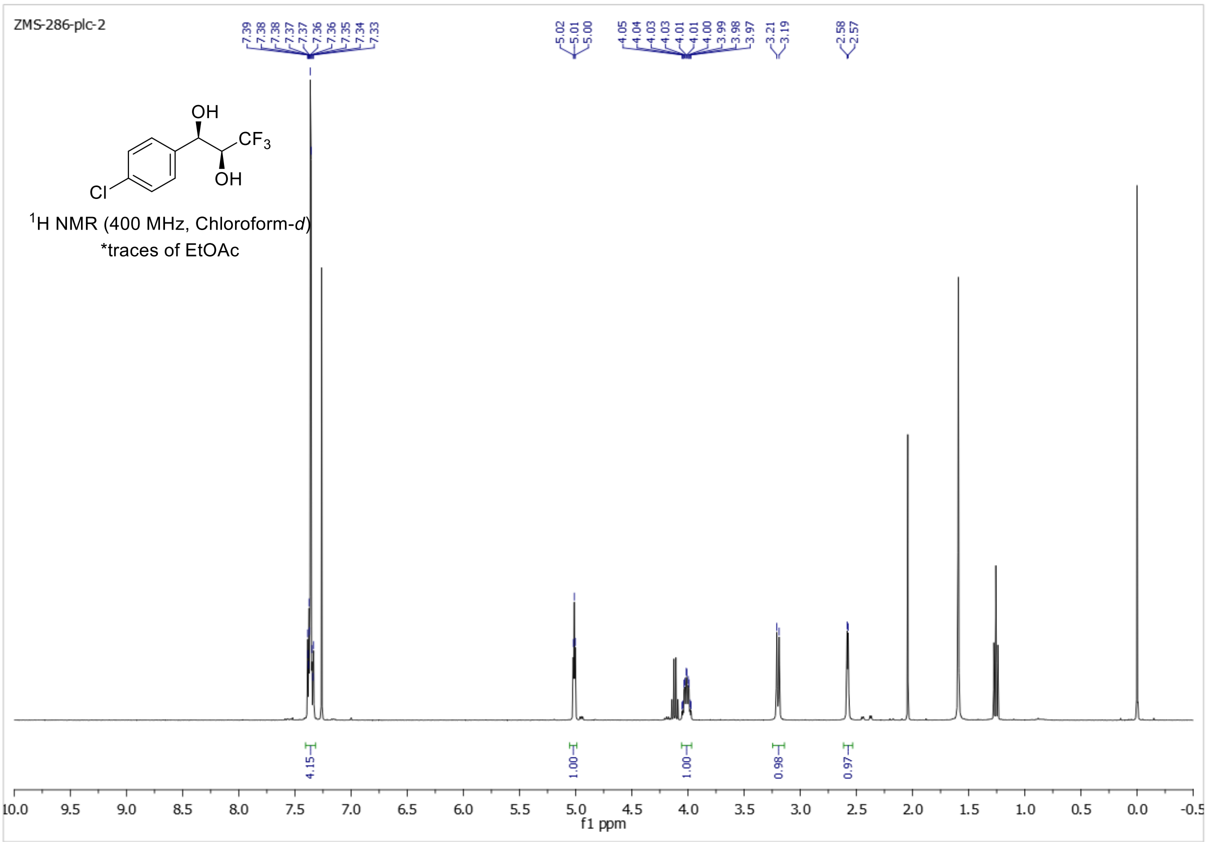

Compound ***syn*-3c**, ^{19}F NMR:

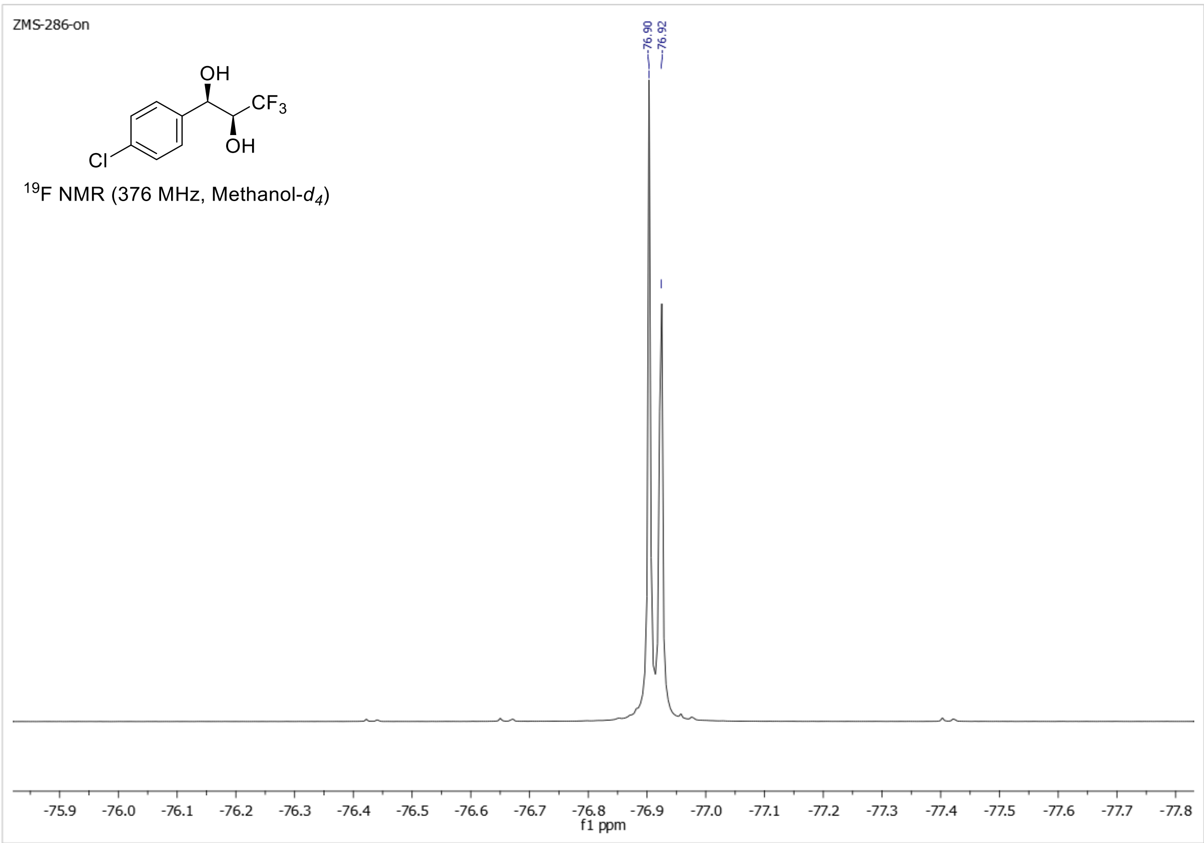

Compound ***syn*-3c**, ^{13}C NMR:

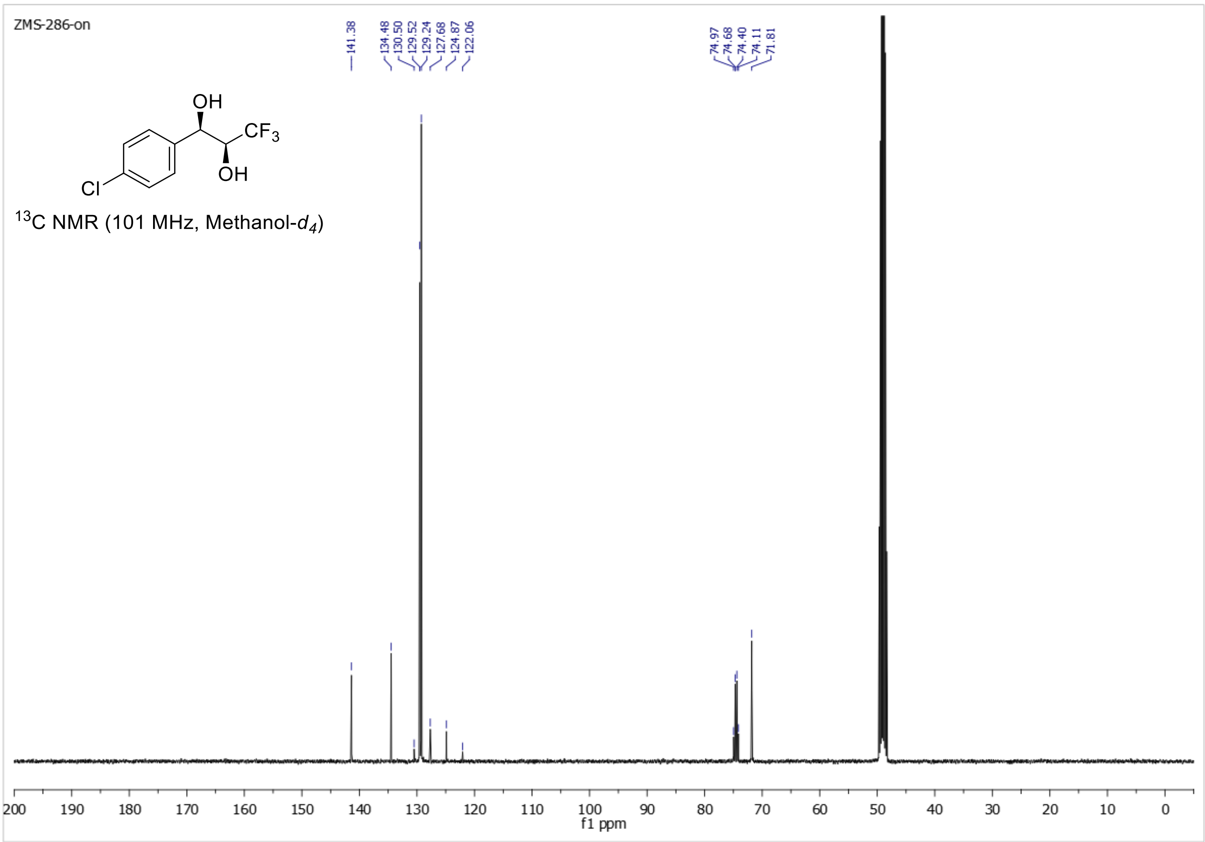

Compound **(±)-*anti*-3c**, ^{1}H NMR:

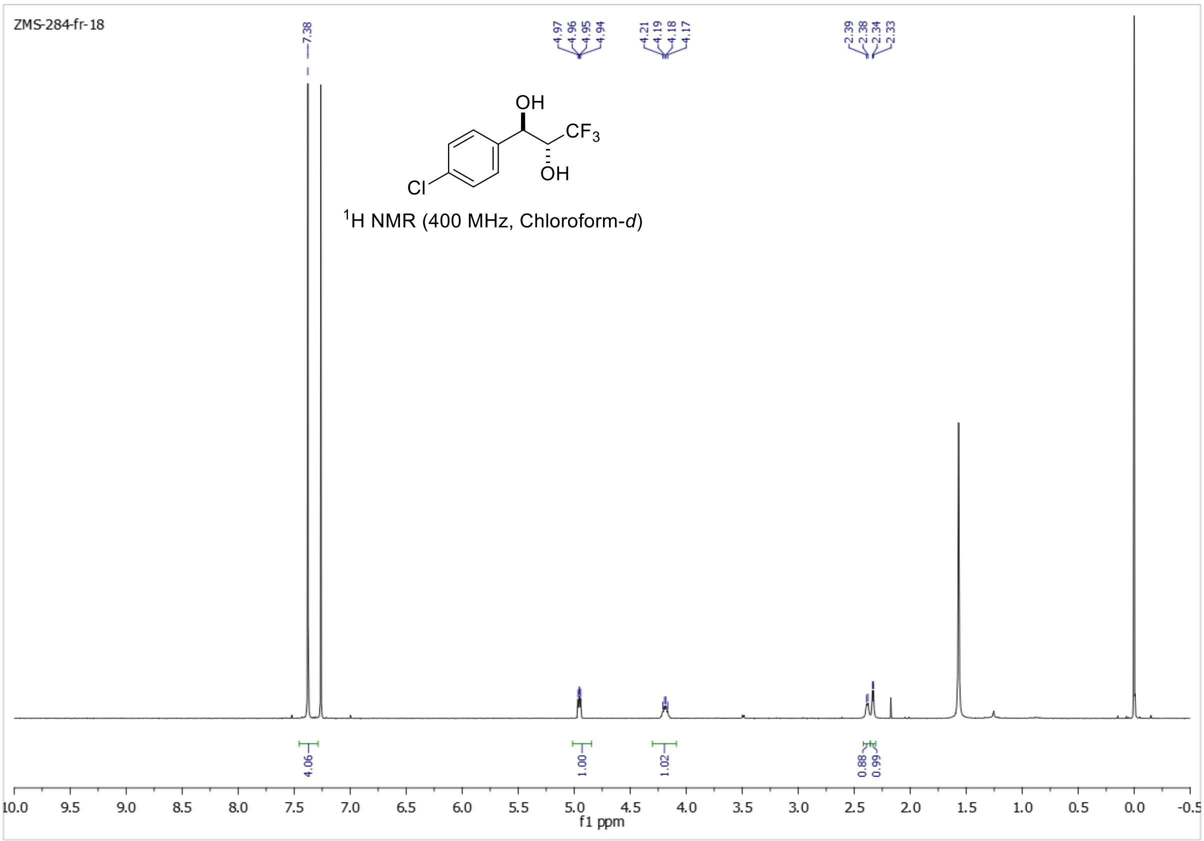

Compound **(±)-*anti*-3c**, ^{19}F NMR:

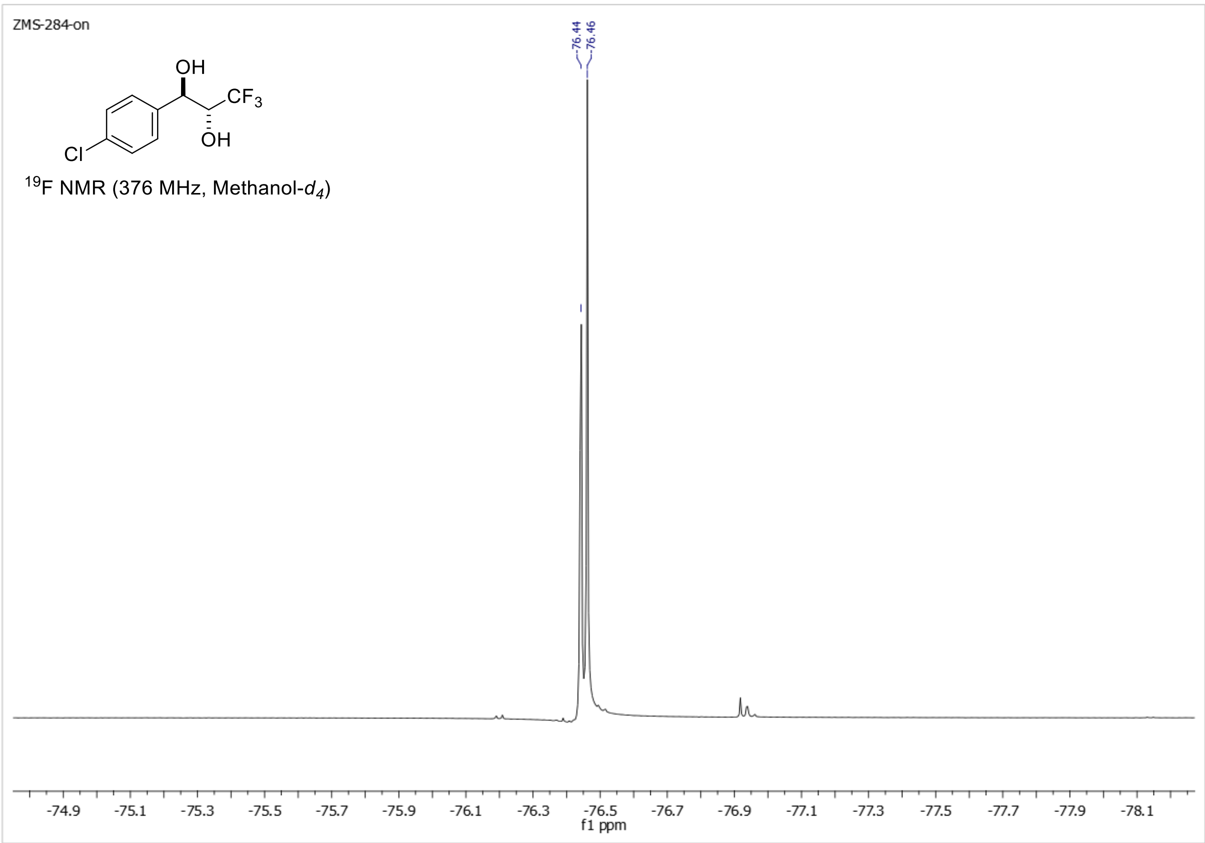

Compound **(±)-*anti*-3c**, ^{13}C NMR:

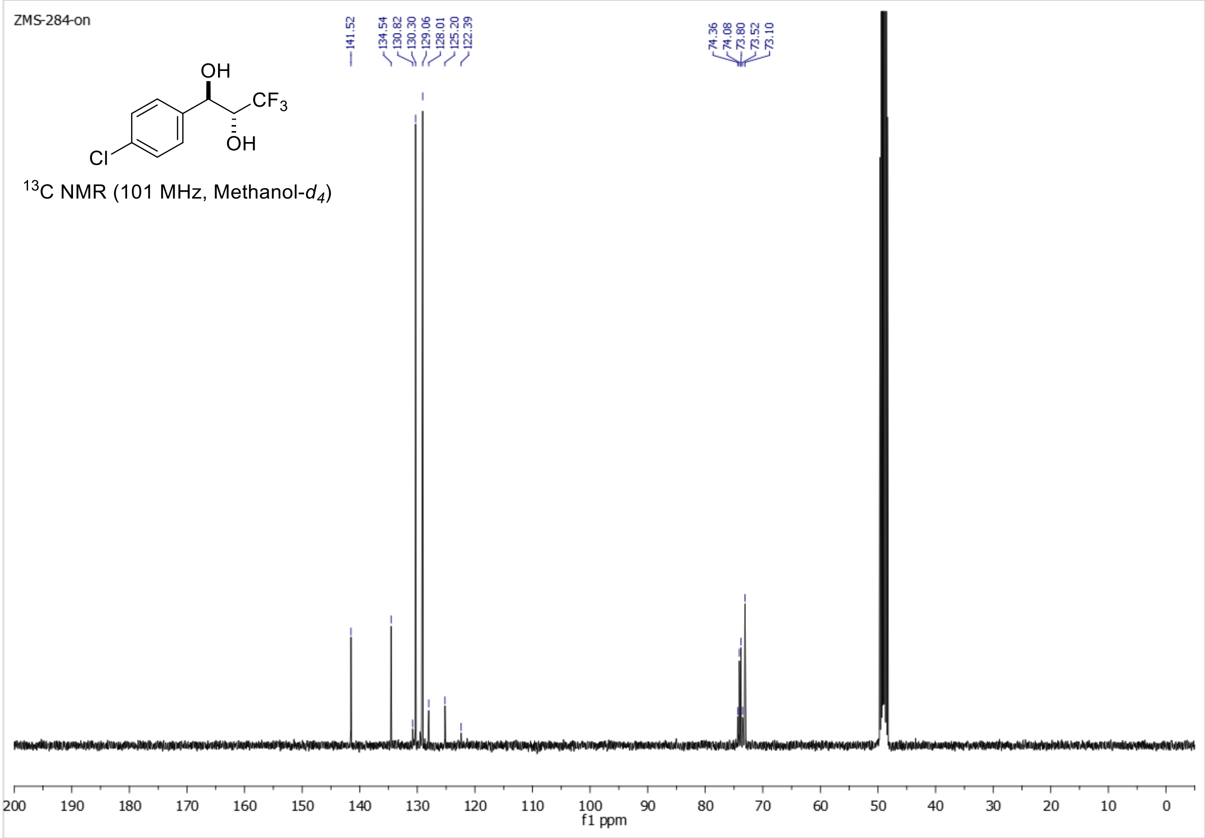

Compound ***syn*-3d**, ^{1}H NMR:

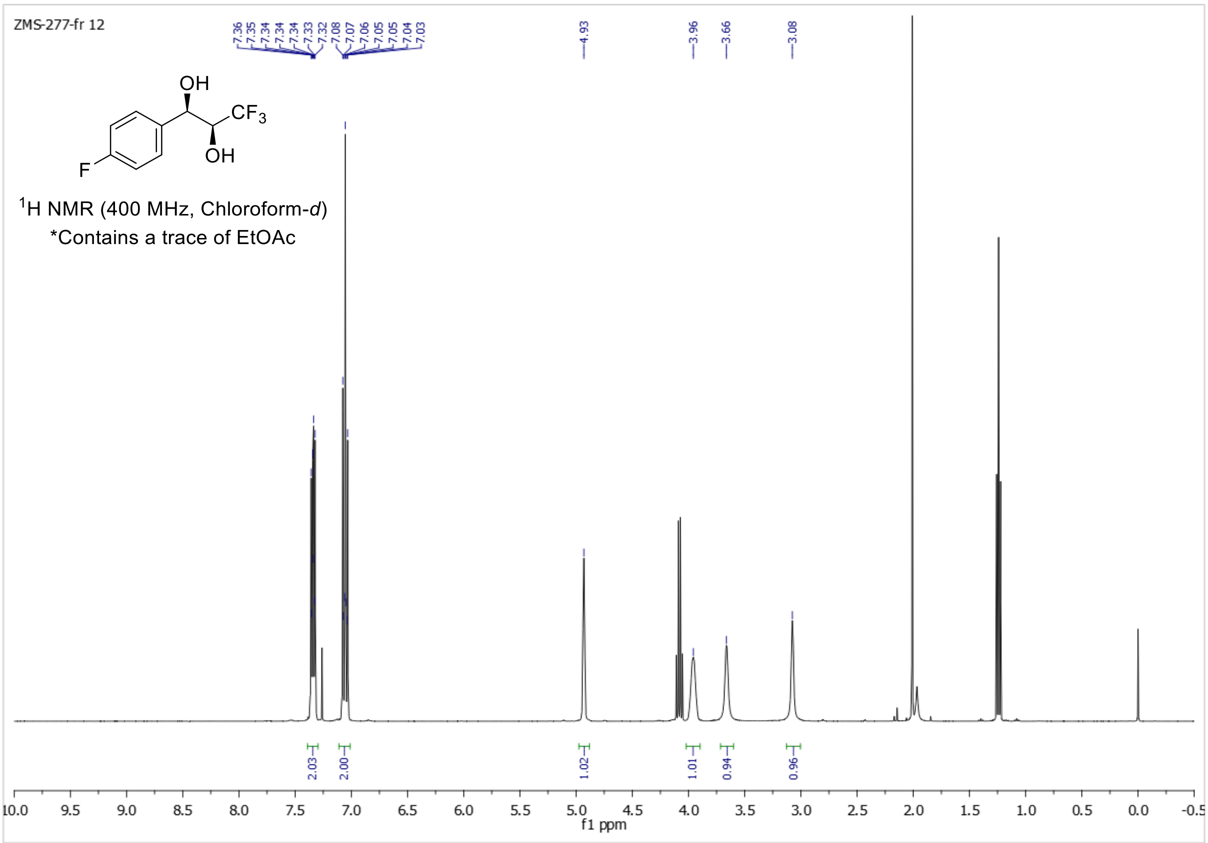

Compound ***syn*-3d**, ^{19}F NMR:

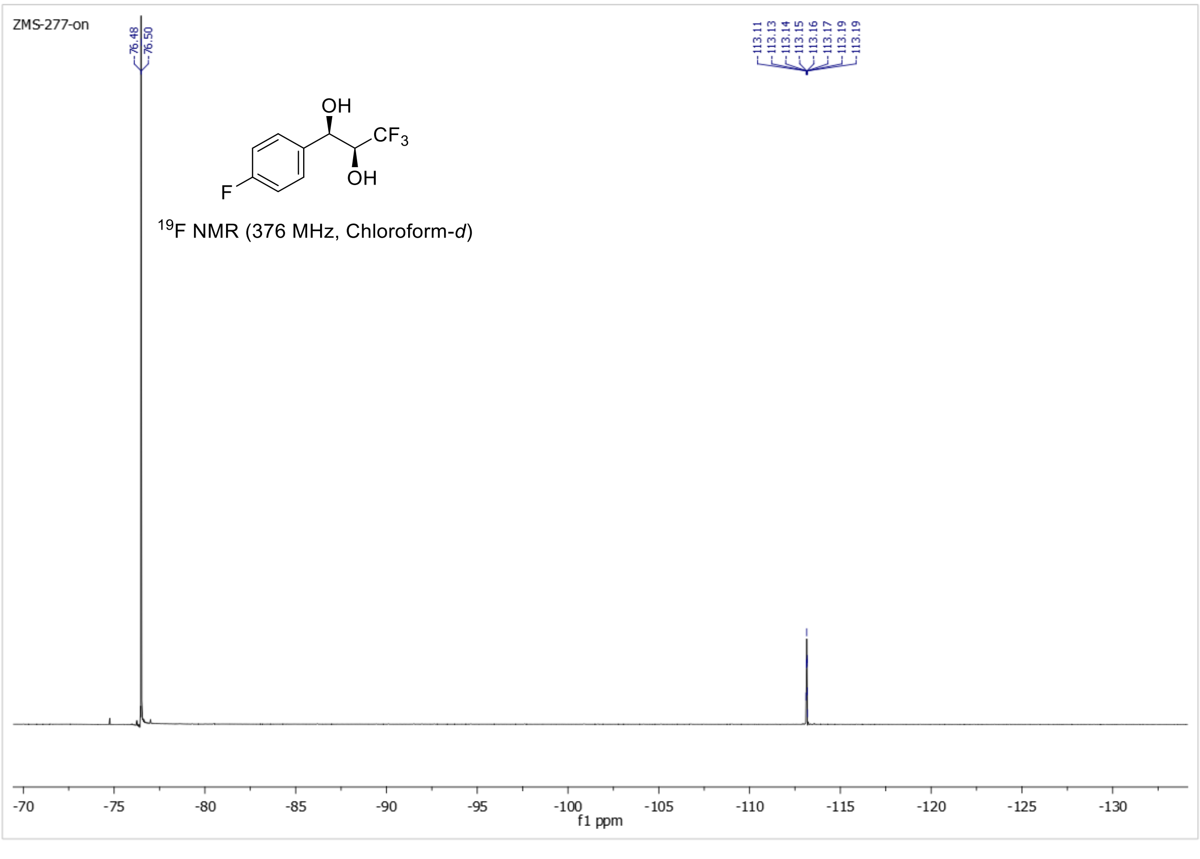

Compound ***syn*-3d**, ^{13}C NMR:

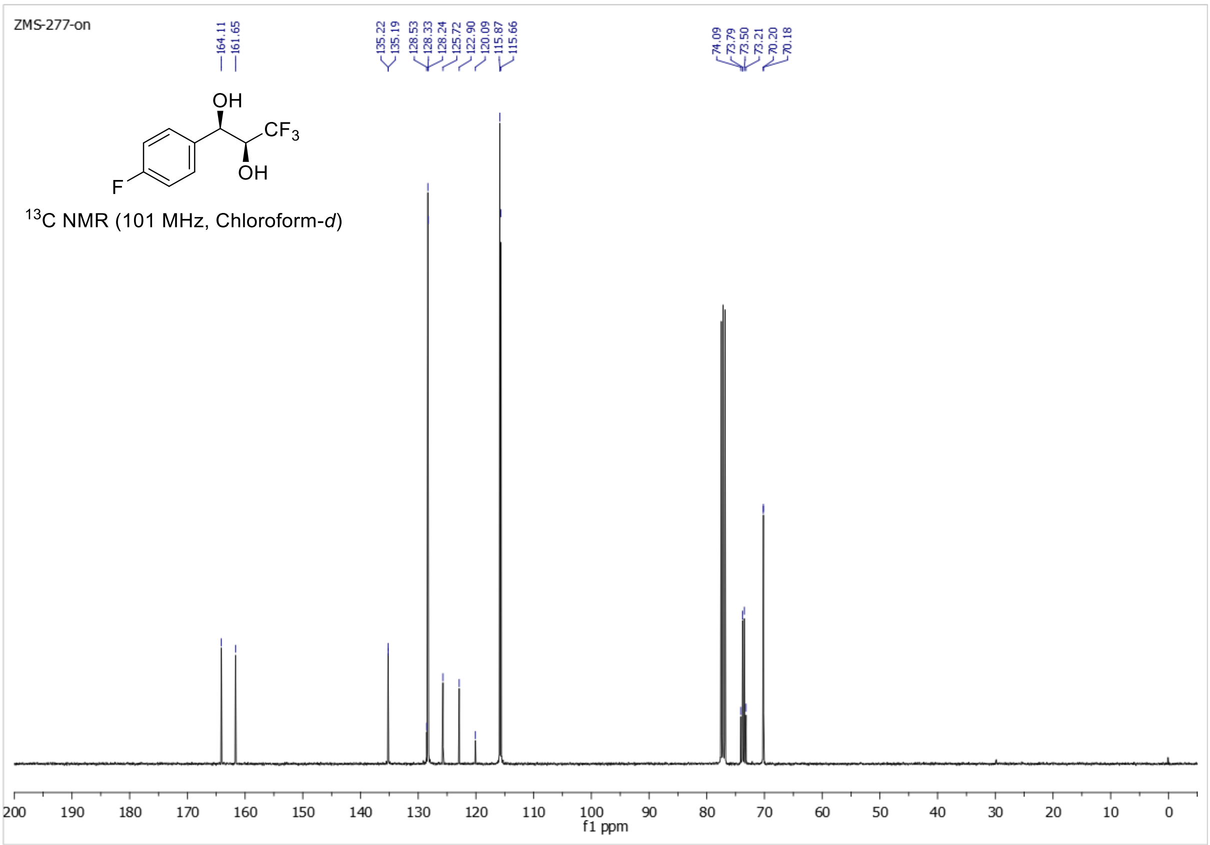

Compound **(±)-*anti*-3d**, ^{1}H NMR:

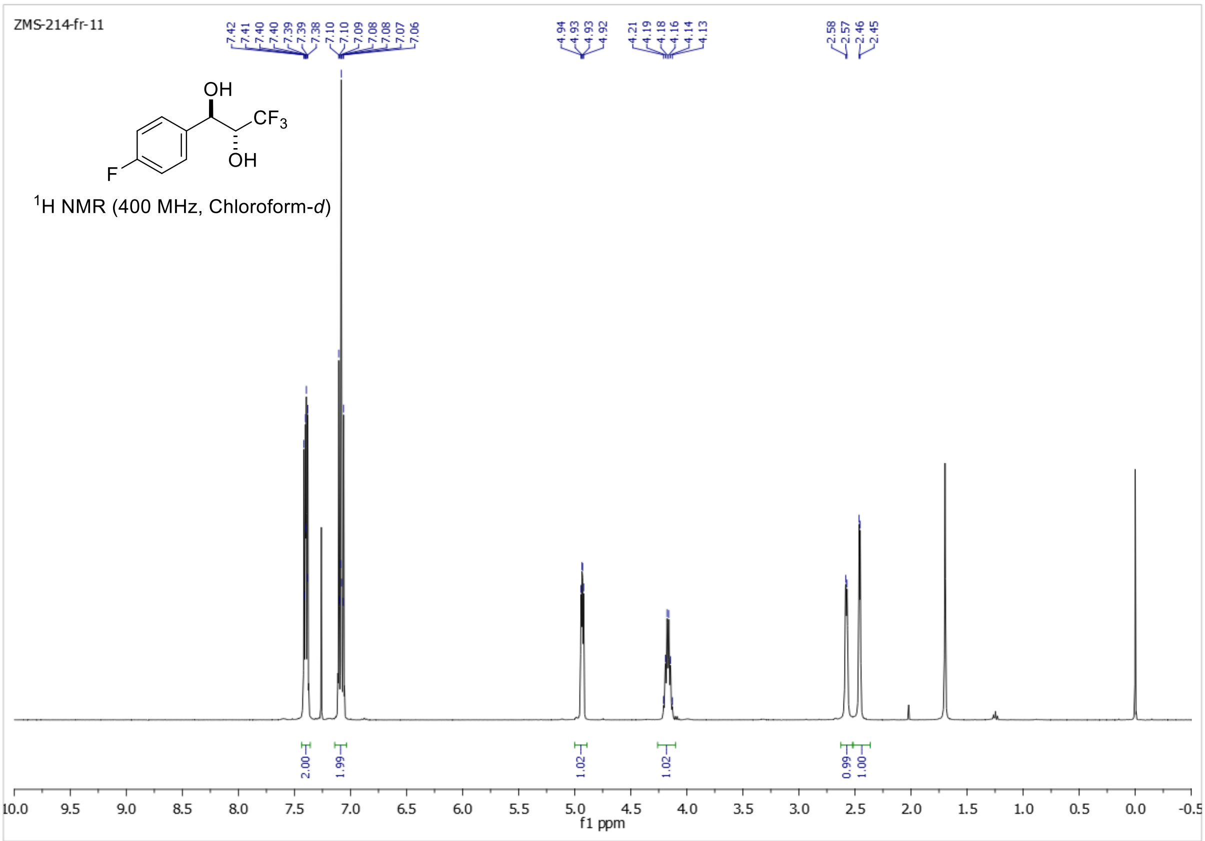

Compound **(±)-*anti*-3d**, ^{19}F NMR:

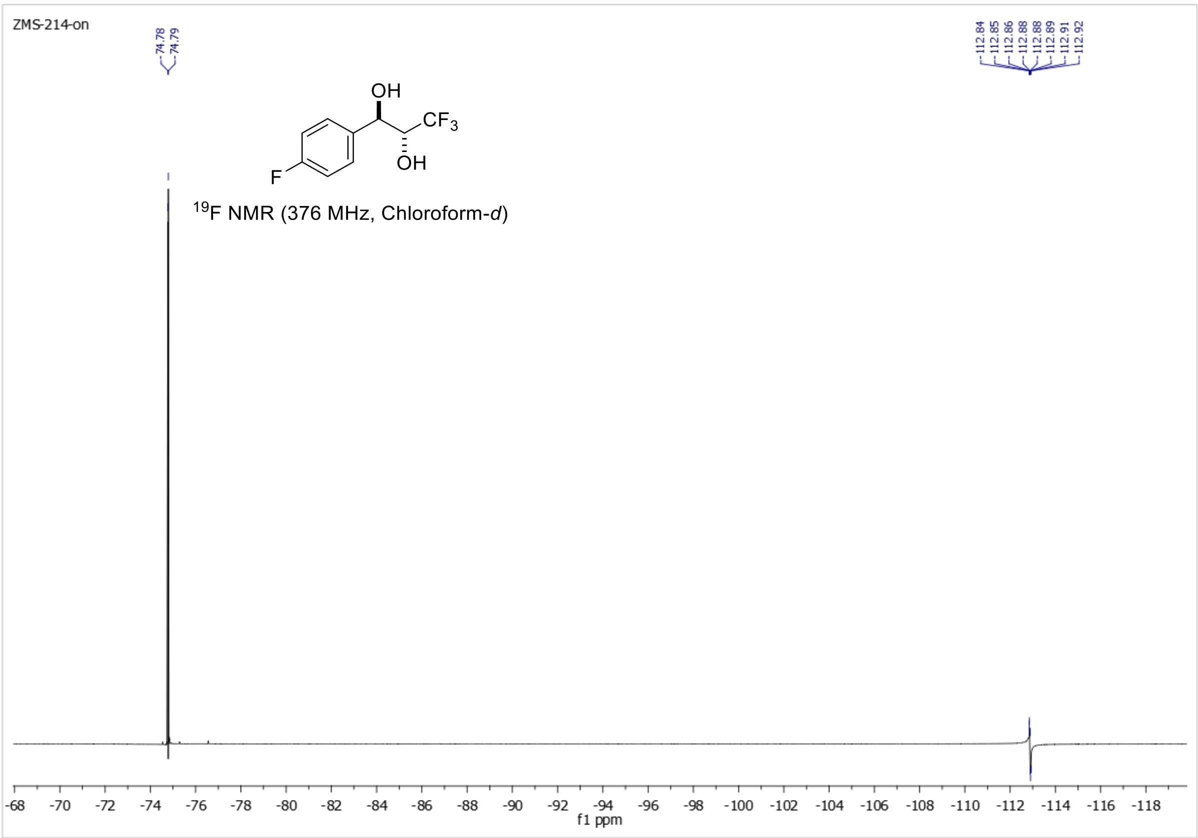

Compound **(±)-*anti*-3d**, ^{13}C NMR:

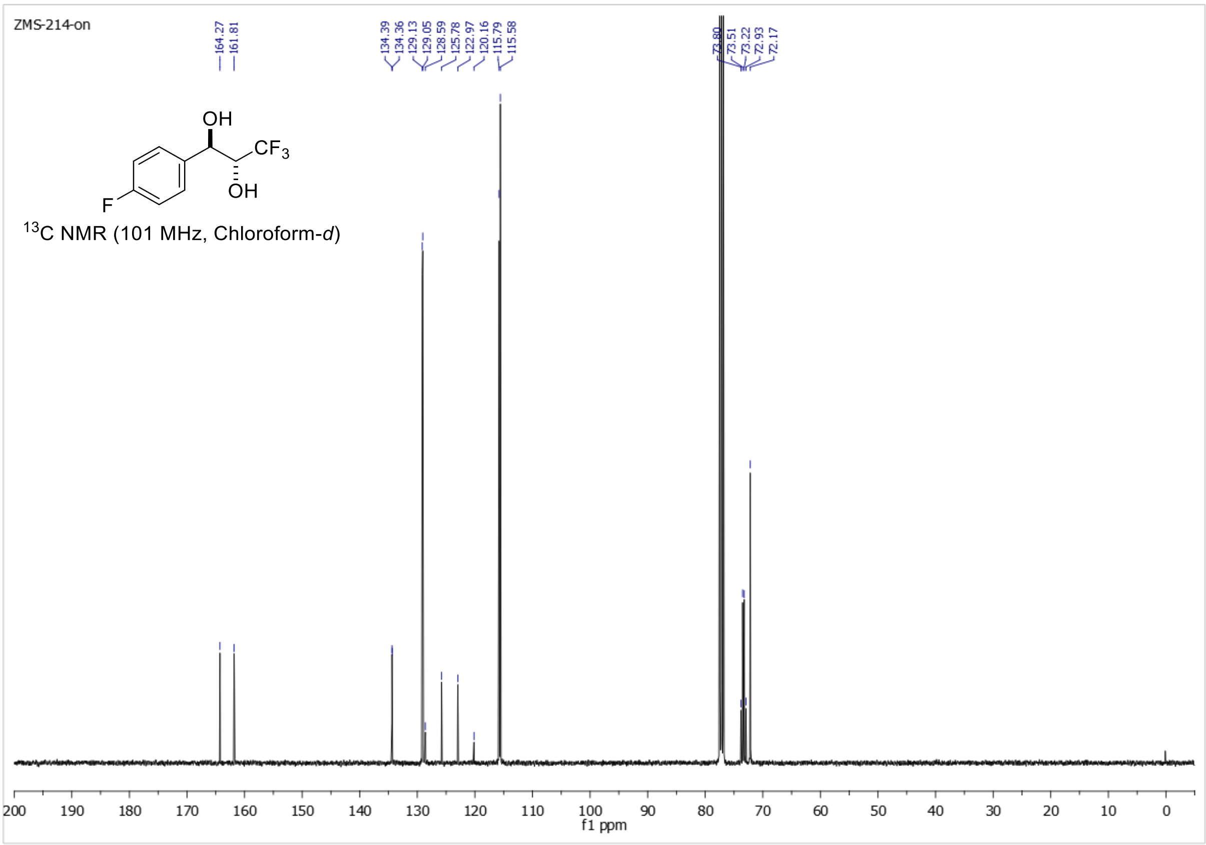

Compound ***syn*-3e**, ^{1}H NMR:

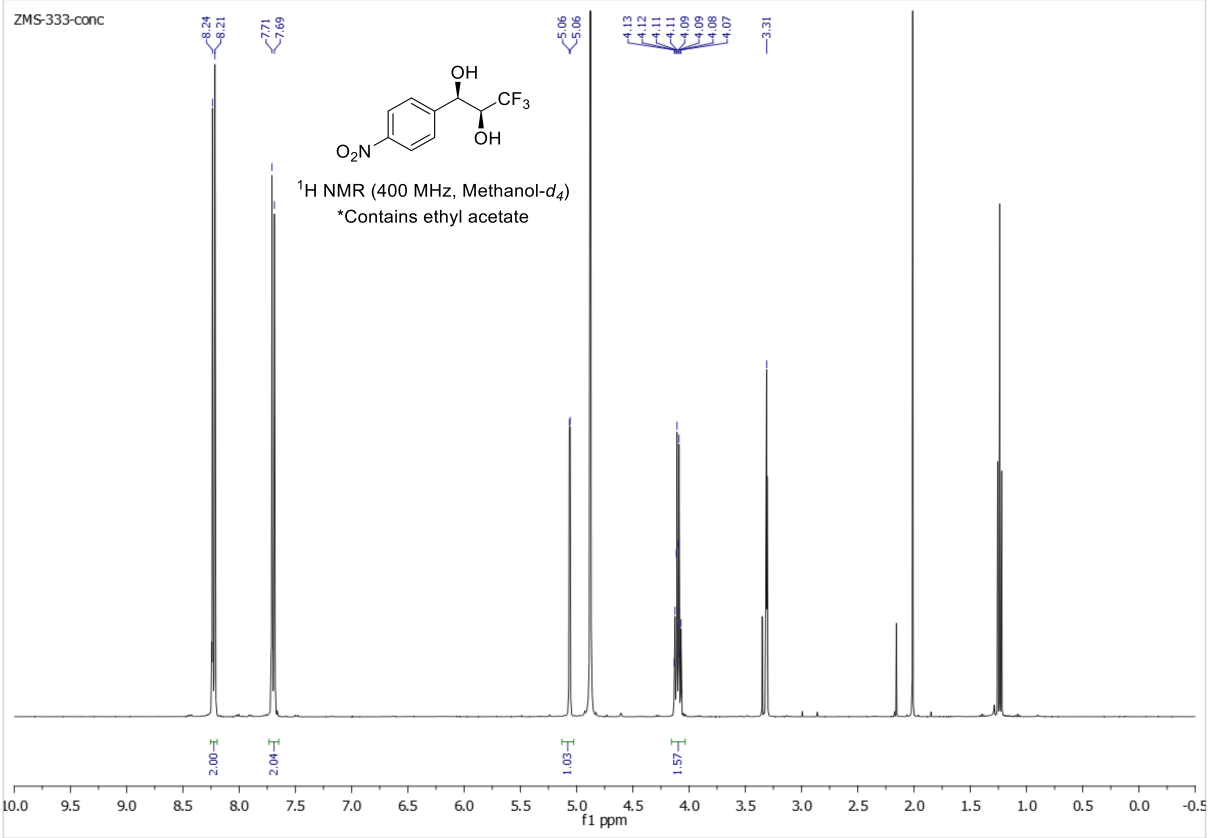

Compound ***syn*-3e**, ^{19}F NMR:

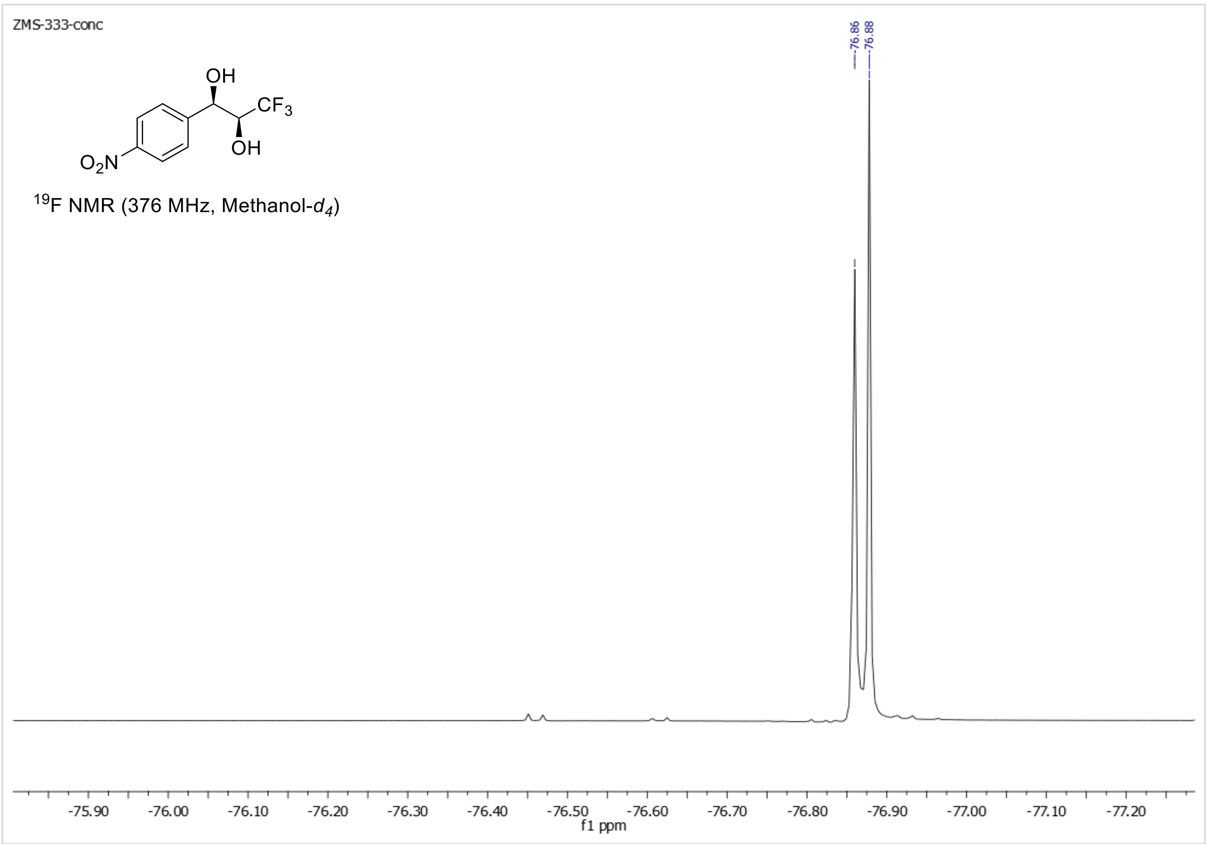

Compound ***syn*-3e**, ^{13}C NMR:

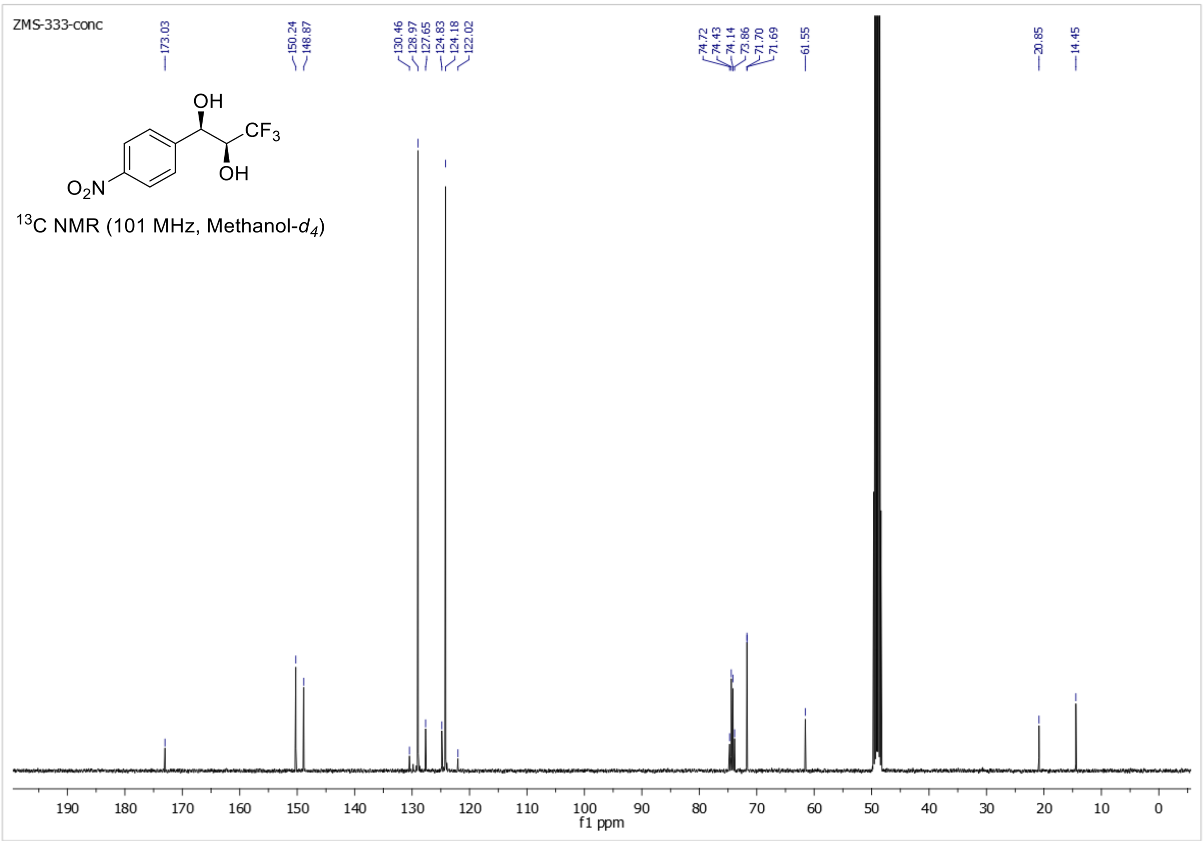

Compound **(±)-*anti*-3e**, ^{1}H NMR:

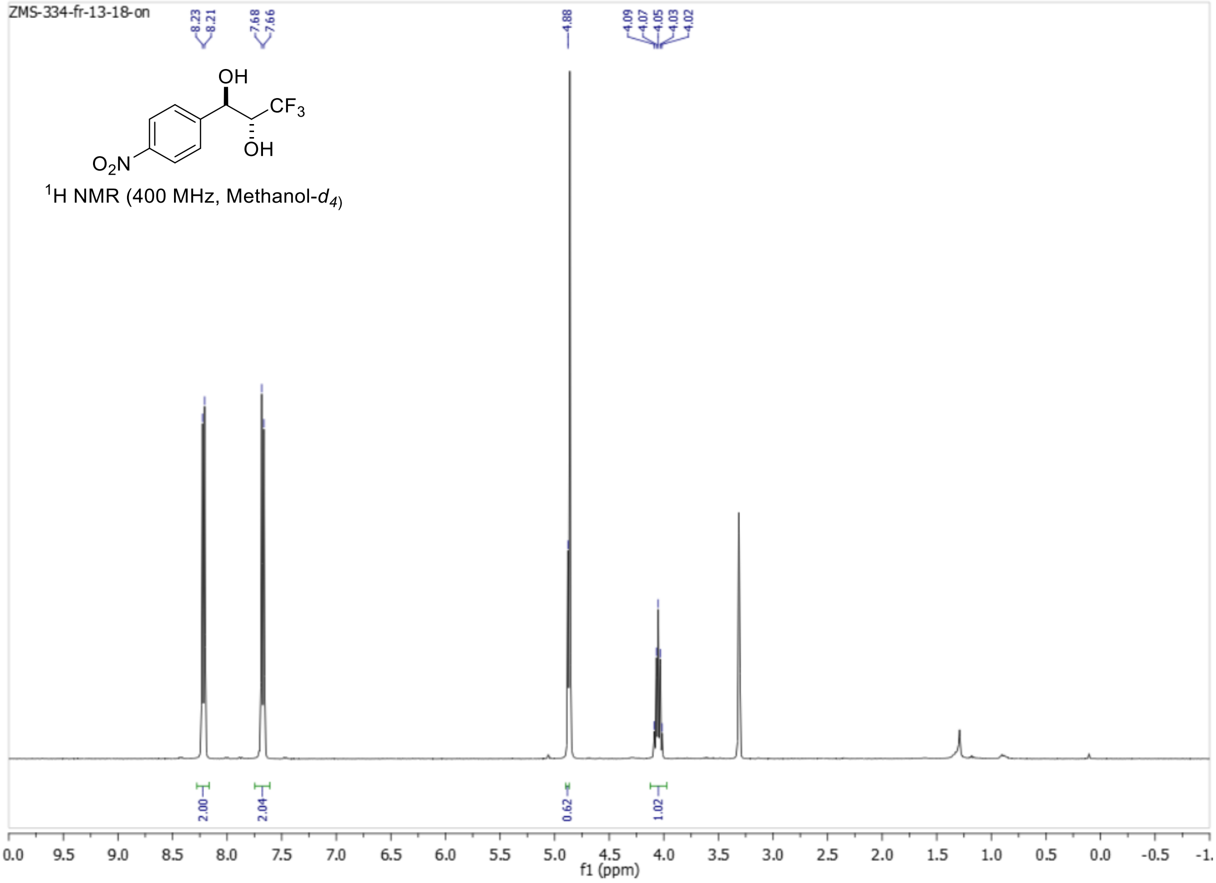

Compound **(±)-*anti*-3e**, ^{19}F NMR:

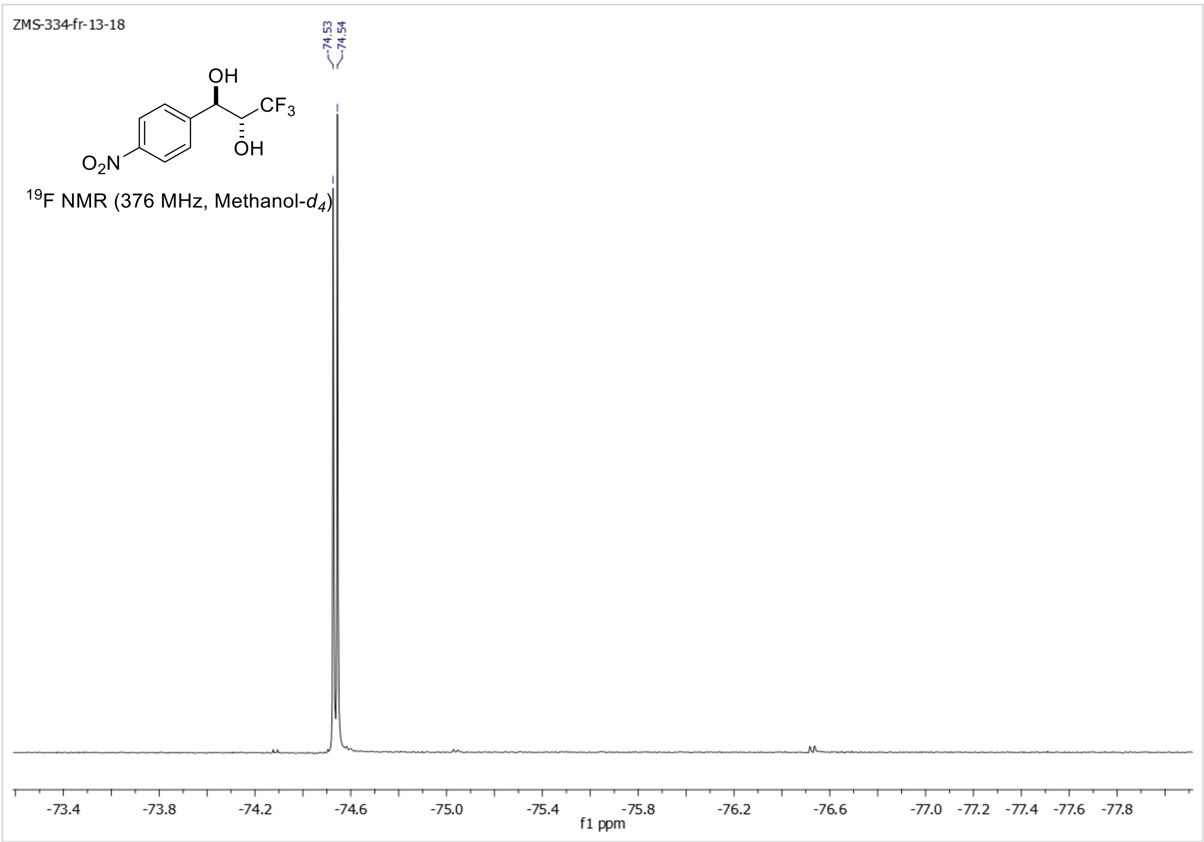

Compound **(±)-*anti*-3e**, ^{13}C NMR:

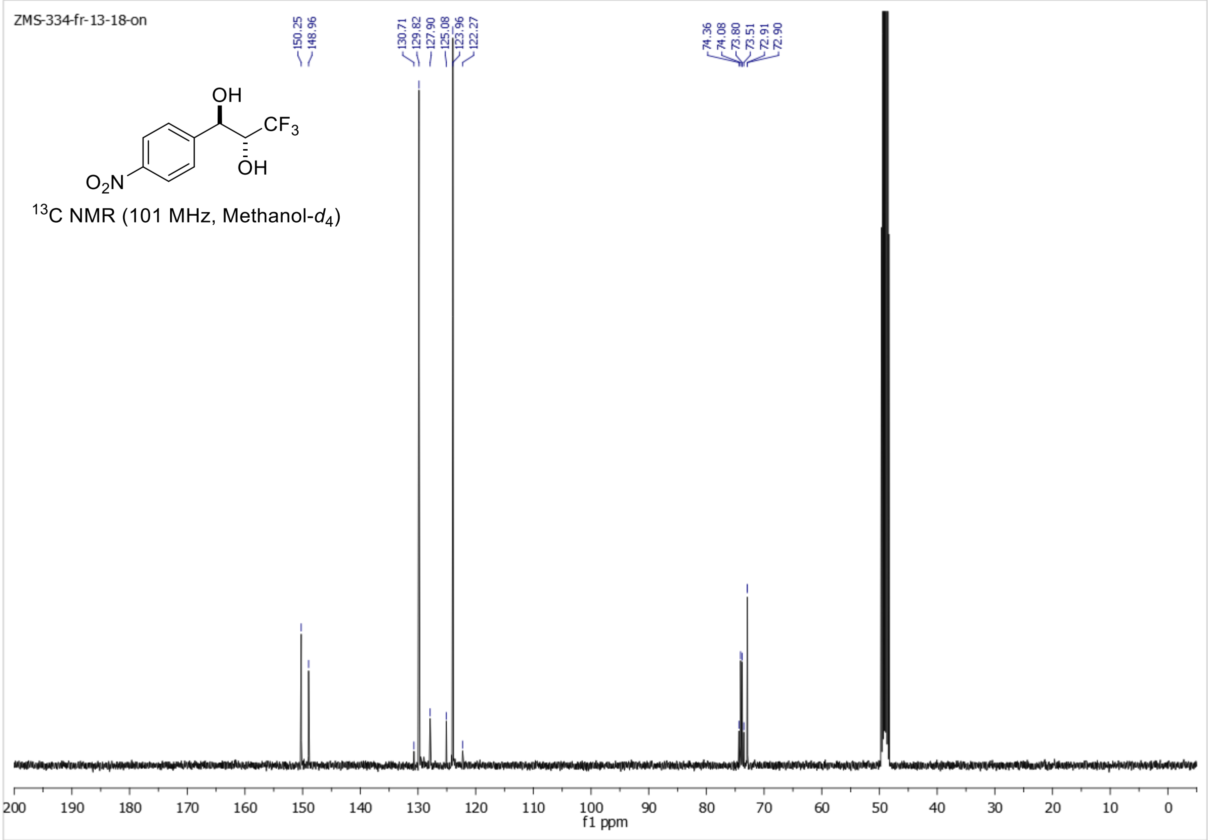

Compound ***syn*-3f**, ^{1}H NMR:

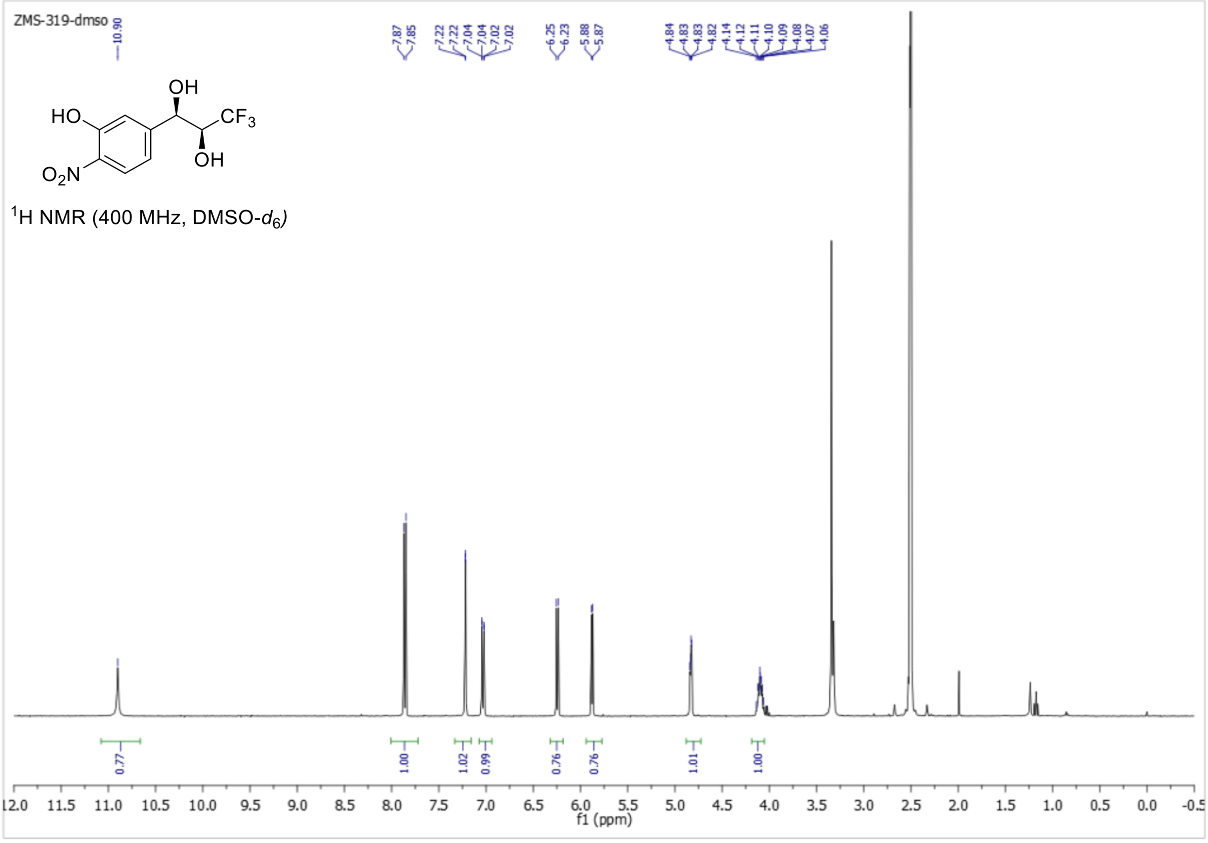

Compound ***syn*-3f**, ^{19}F NMR:

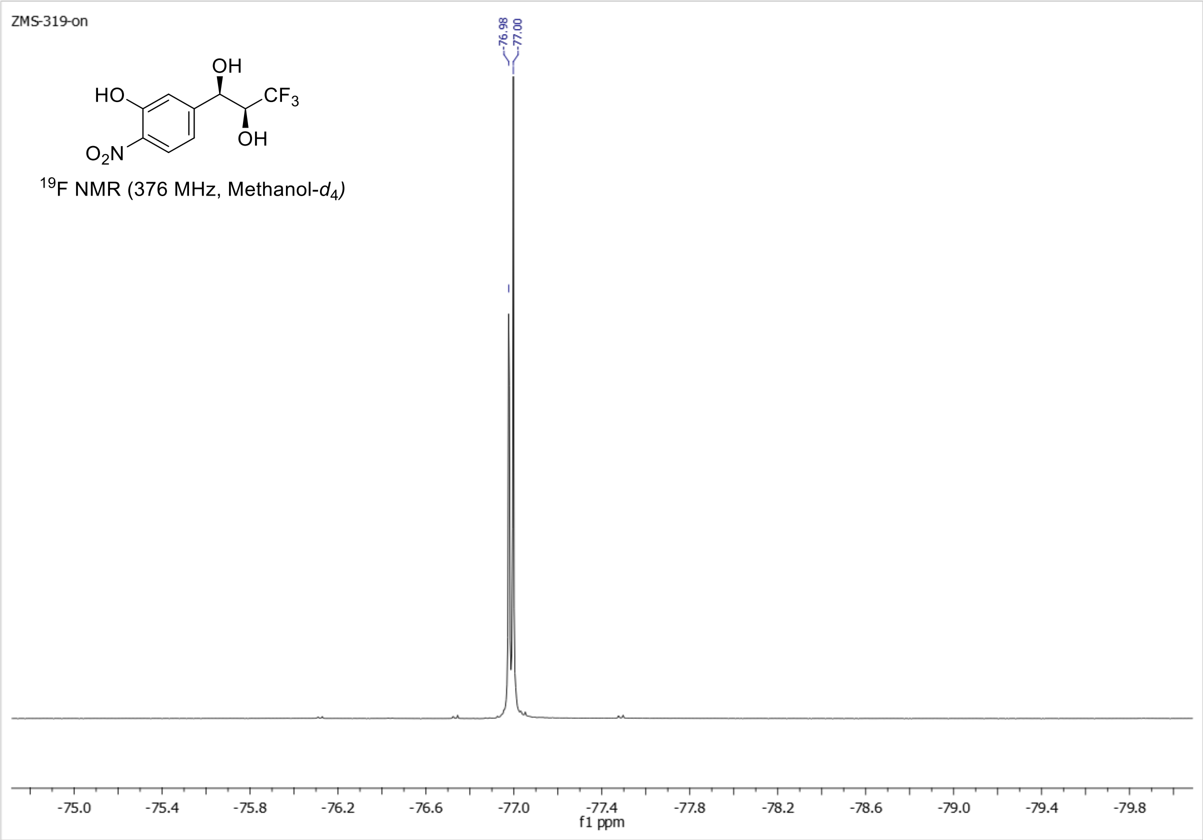

Compound ***syn*-3f**, ^{13}C NMR:

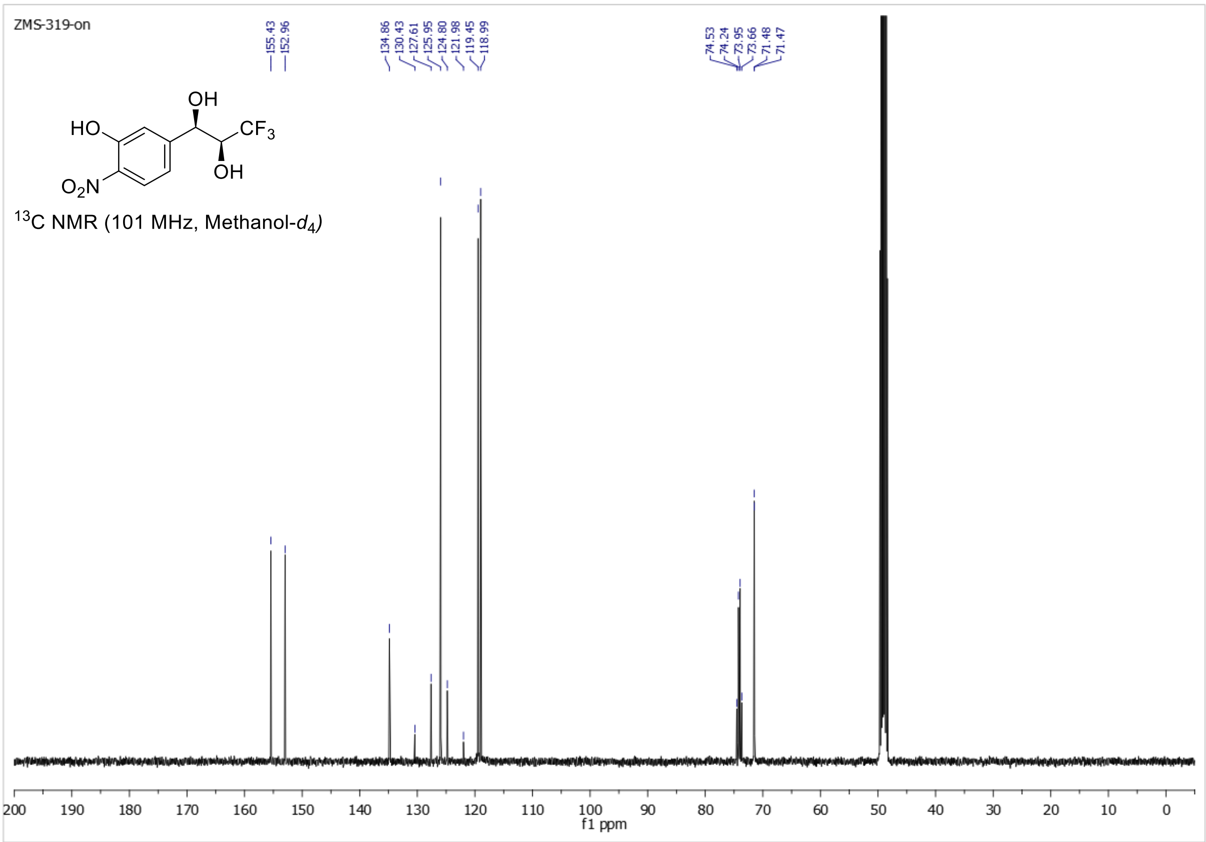

Compound **(±)-*anti*-3f**, ^{1}H NMR:

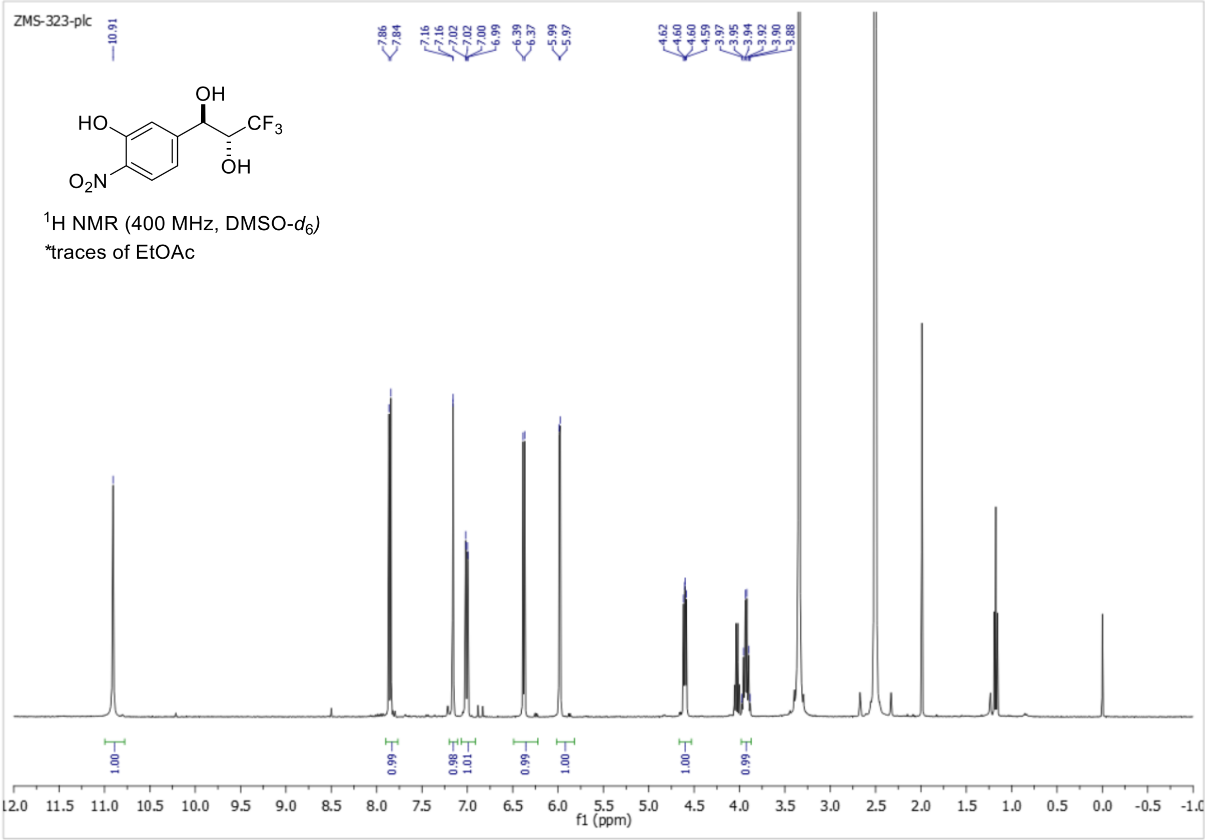

Compound **(±)-*anti*-3f**, ^{19}F NMR:

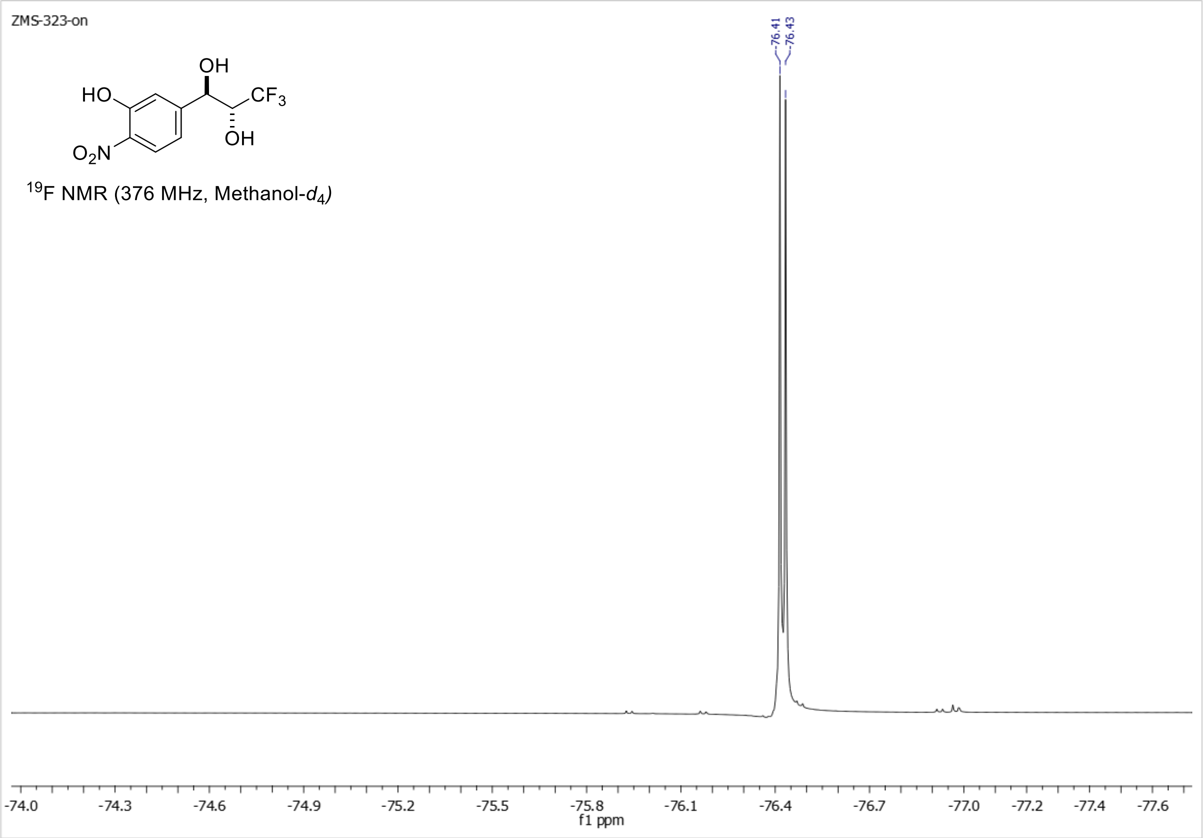

Compound **(±)-*anti*-3f**, ^{13}C NMR:

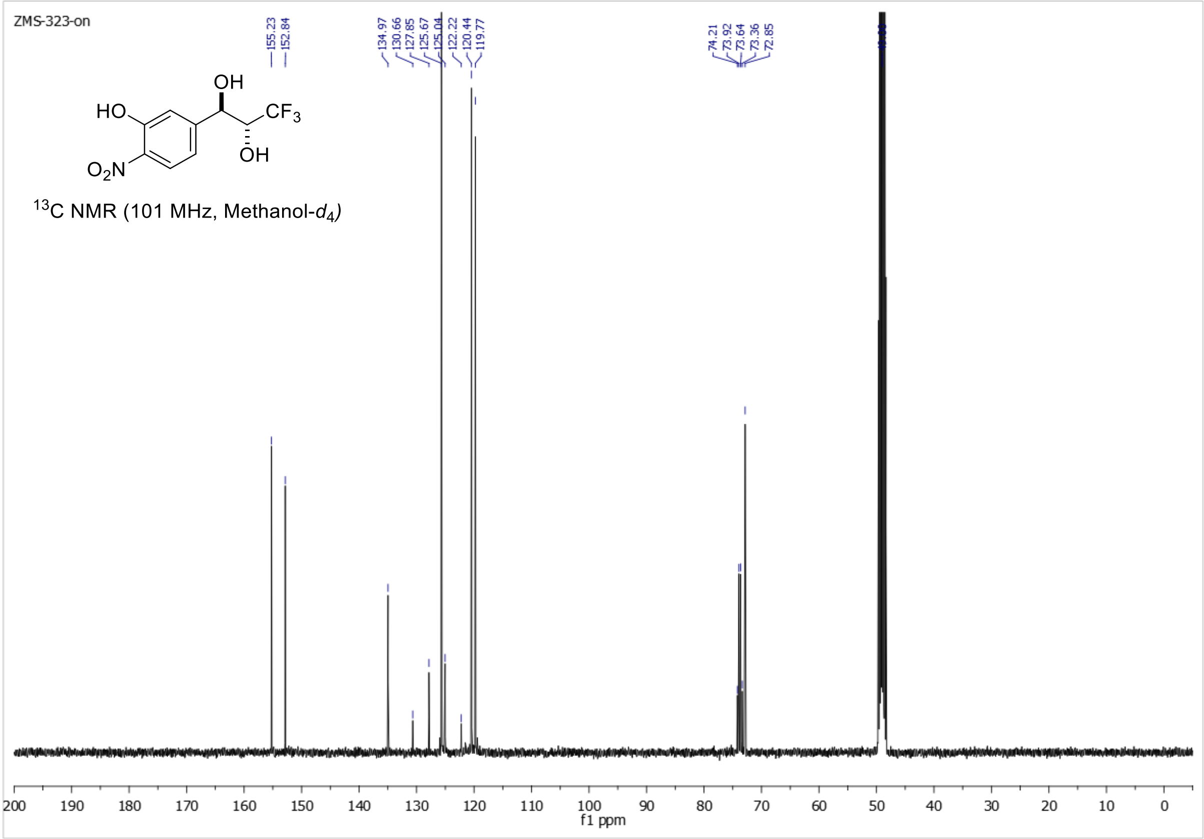

Compound ***syn*-3g**, ^{1}H NMR:

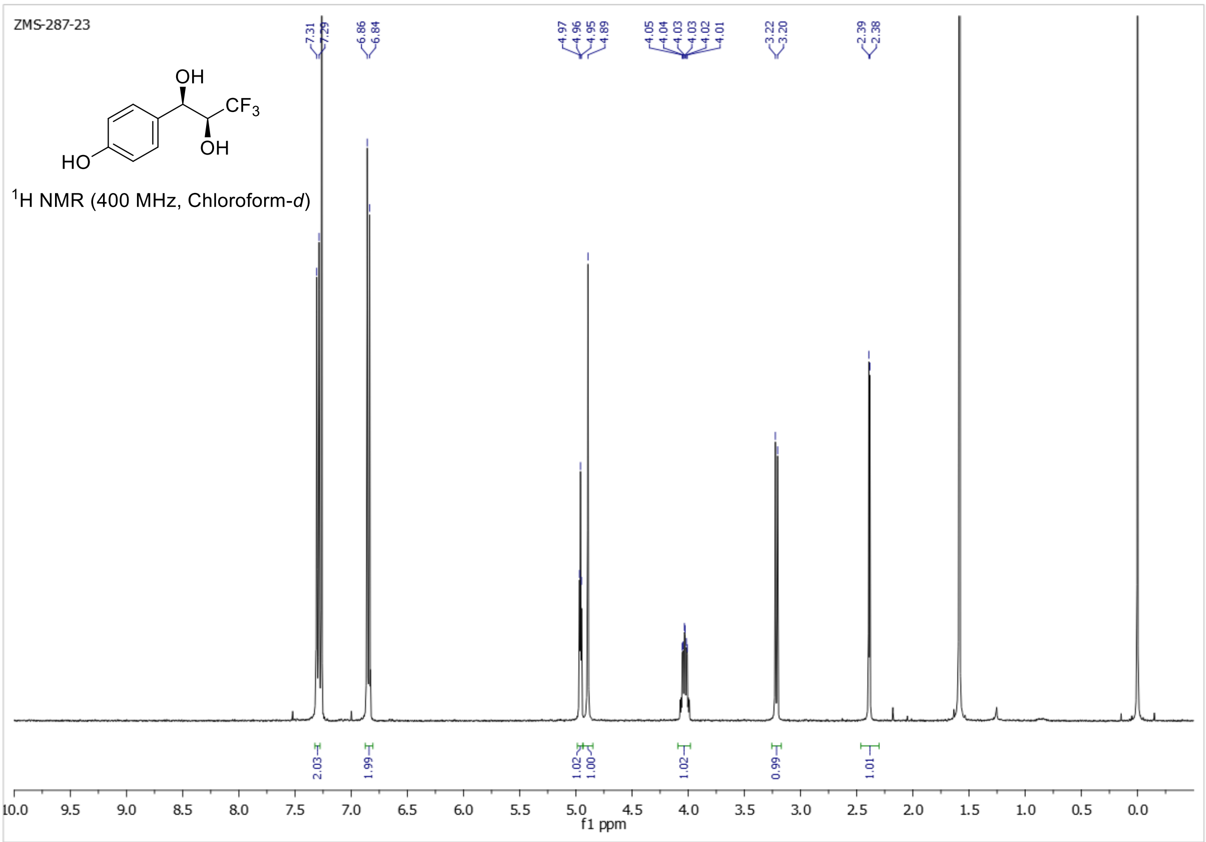

Compound ***syn*-3g**, ^{19}F NMR:

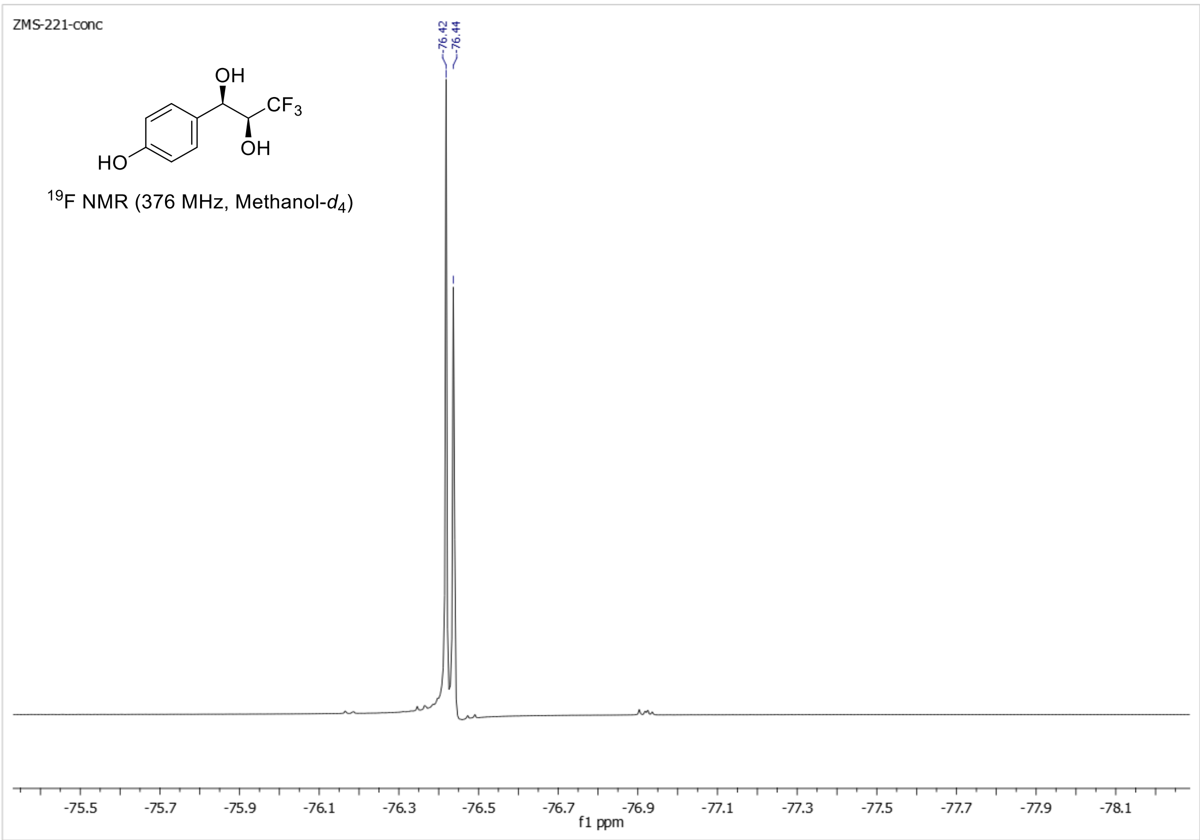

Compound ***syn*-3g**, ^{13}C NMR:

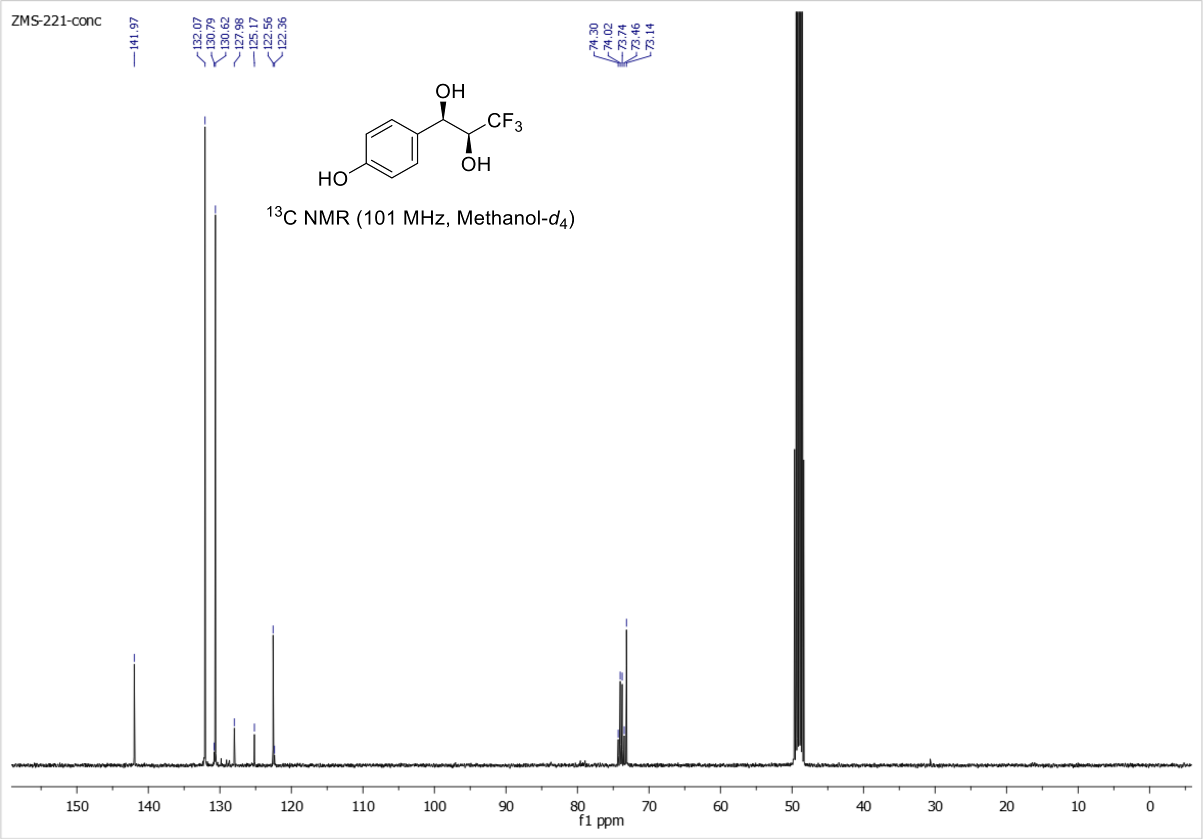

Compound **(±)-*anti*-3g**, ^{1}H NMR:

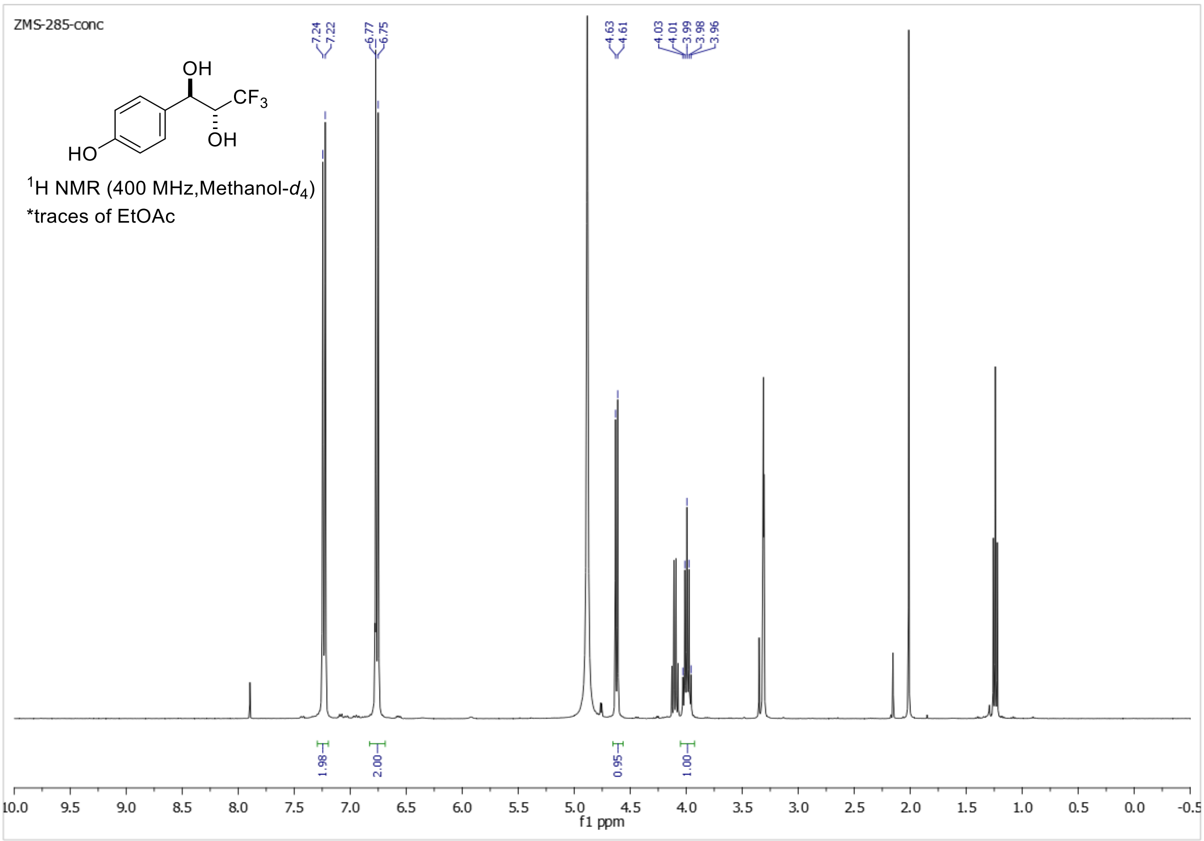

Compound **(±)-*anti*-3g**, ^{19}F NMR:

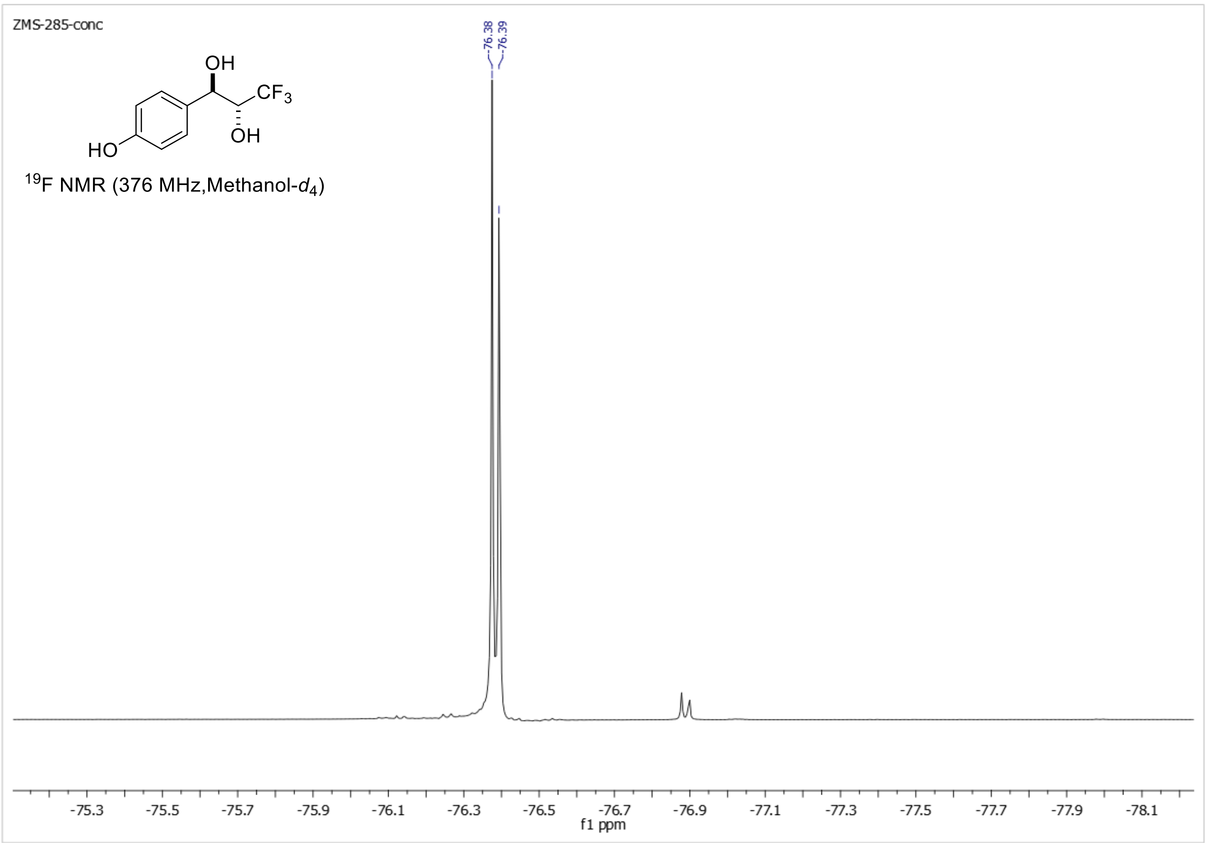

Compound **(±)-*anti*-3g**, ^{13}C NMR:

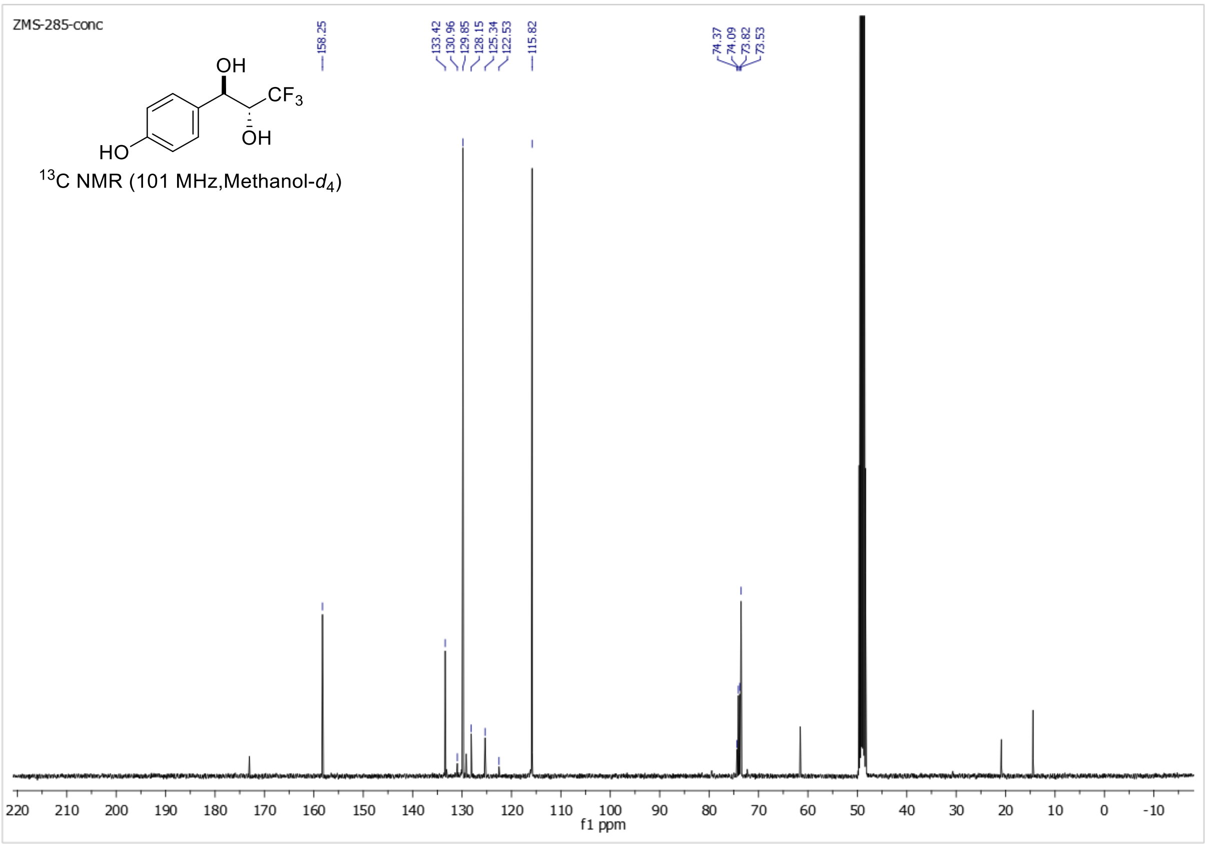

Compound ***syn*-3h**, ^{1}H NMR:

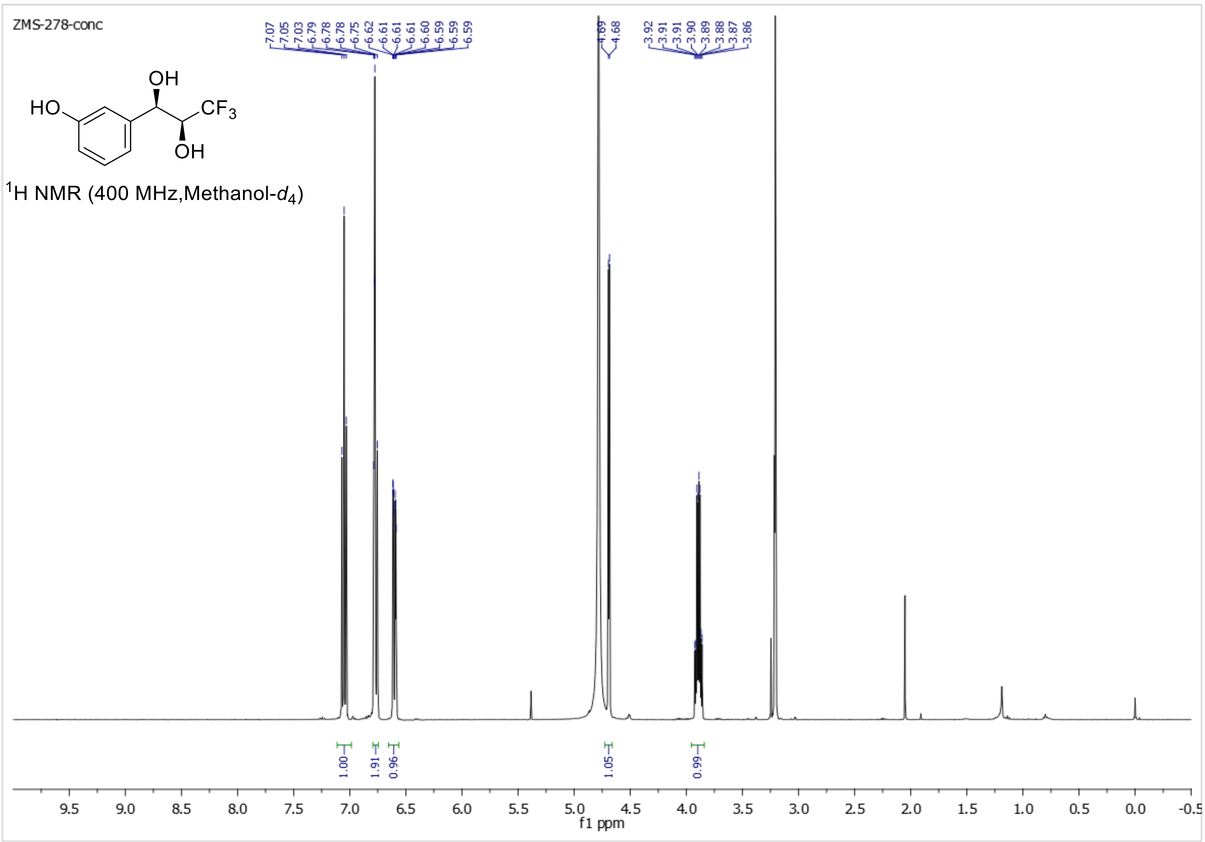

Compound ***syn*-3h**, ^{19}F NMR:

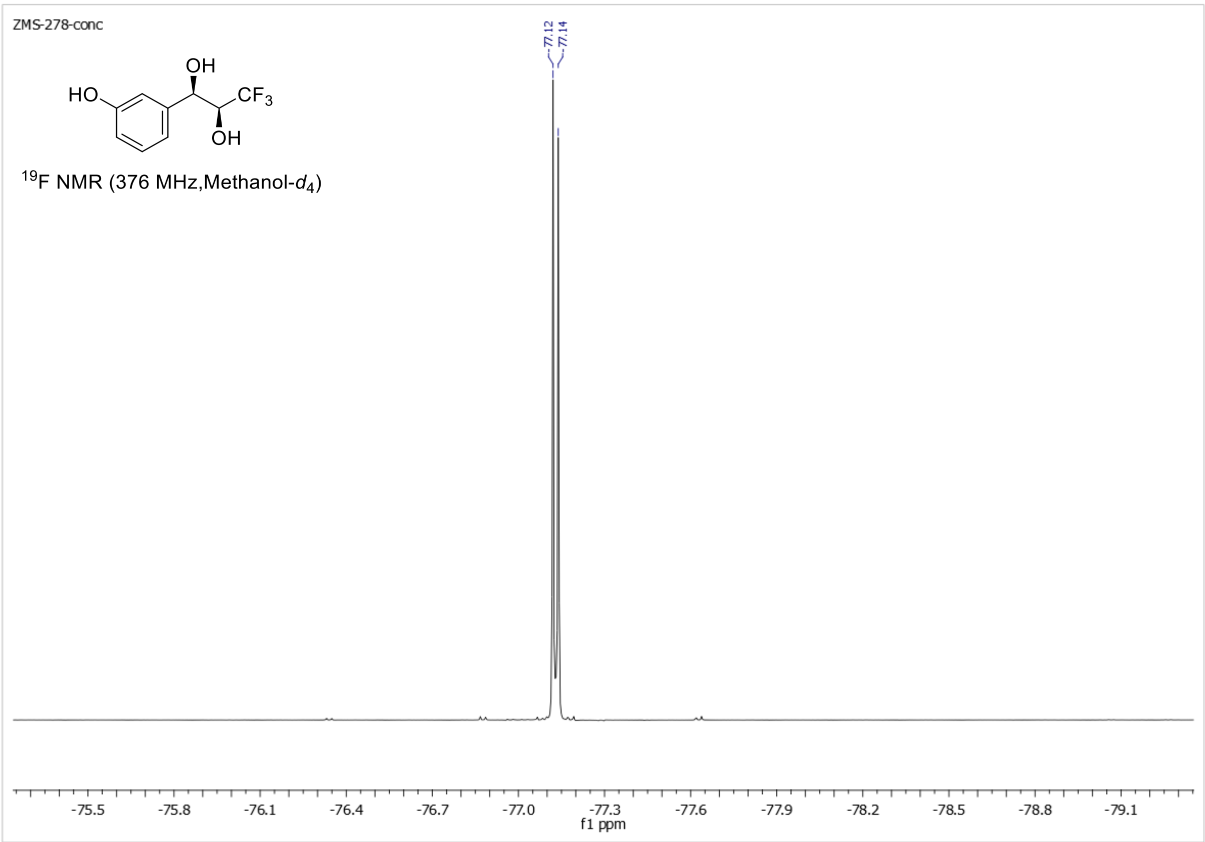

Compound ***syn*-3h**, ^{13}C NMR:

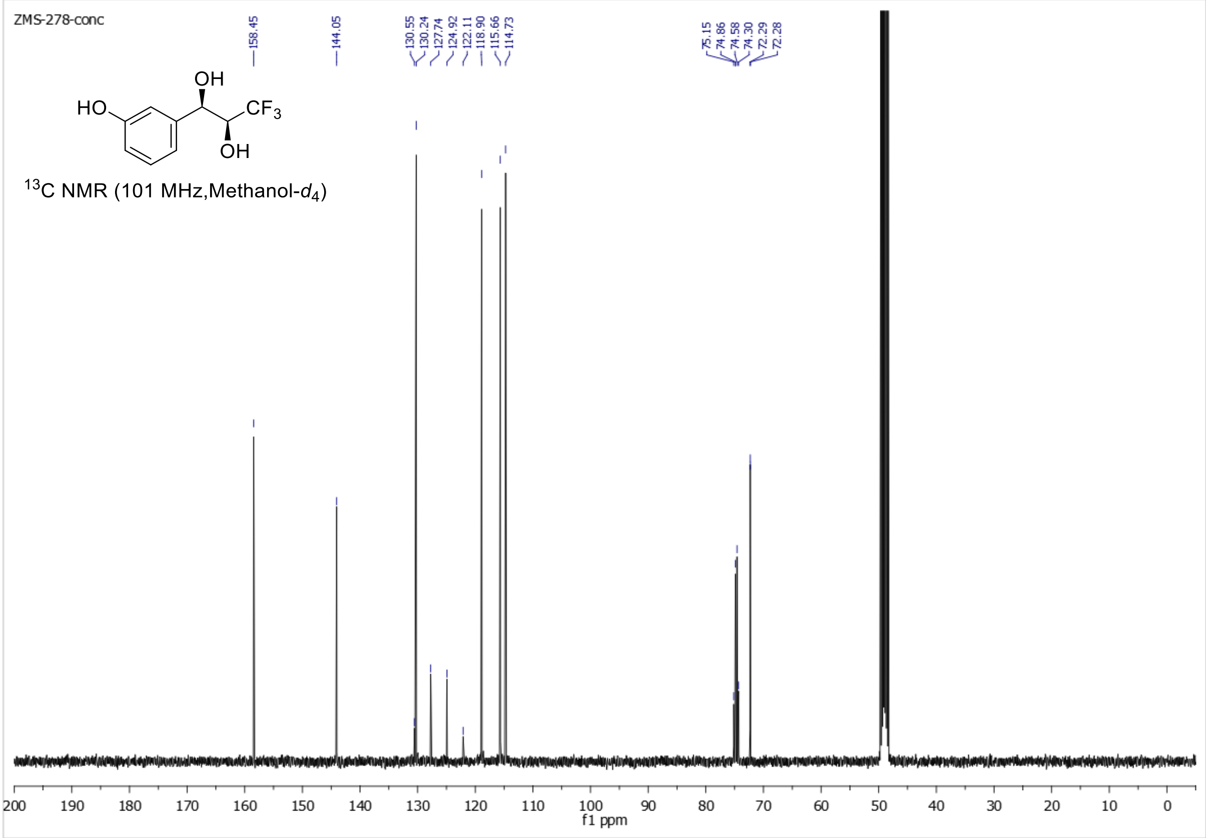

Compound **(±)-*anti*-3h**, ^{1}H NMR:

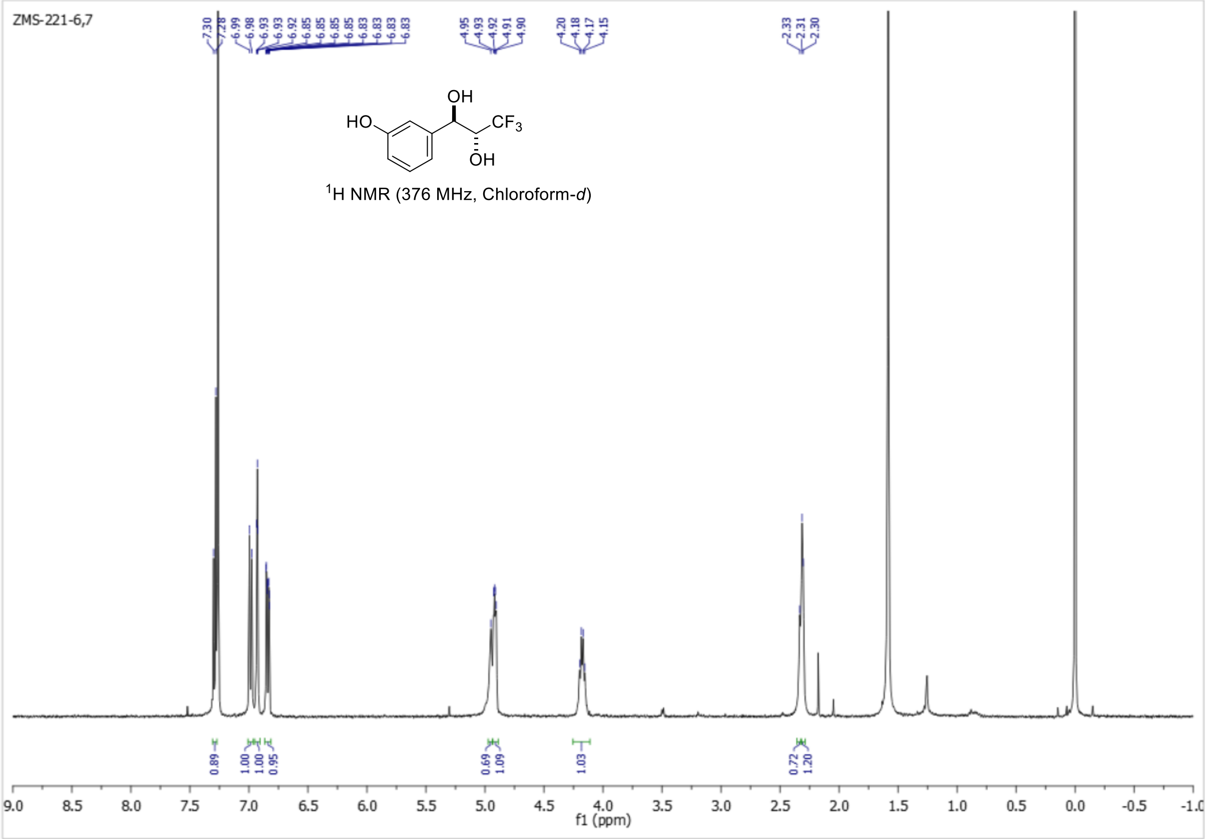

Compound **(±)-*anti*-3h**, ^{19}F NMR:

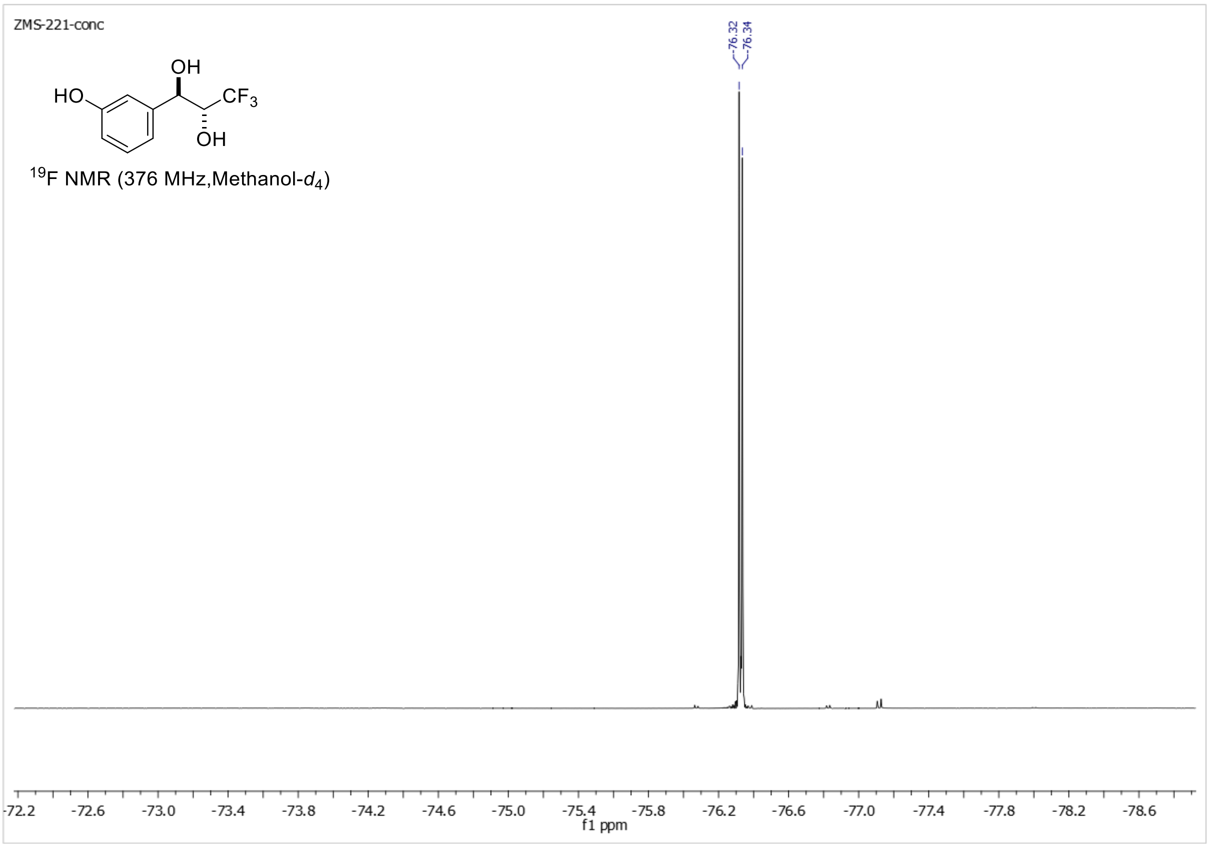

Compound **(±)-*anti*-3h**, ^{13}C NMR:

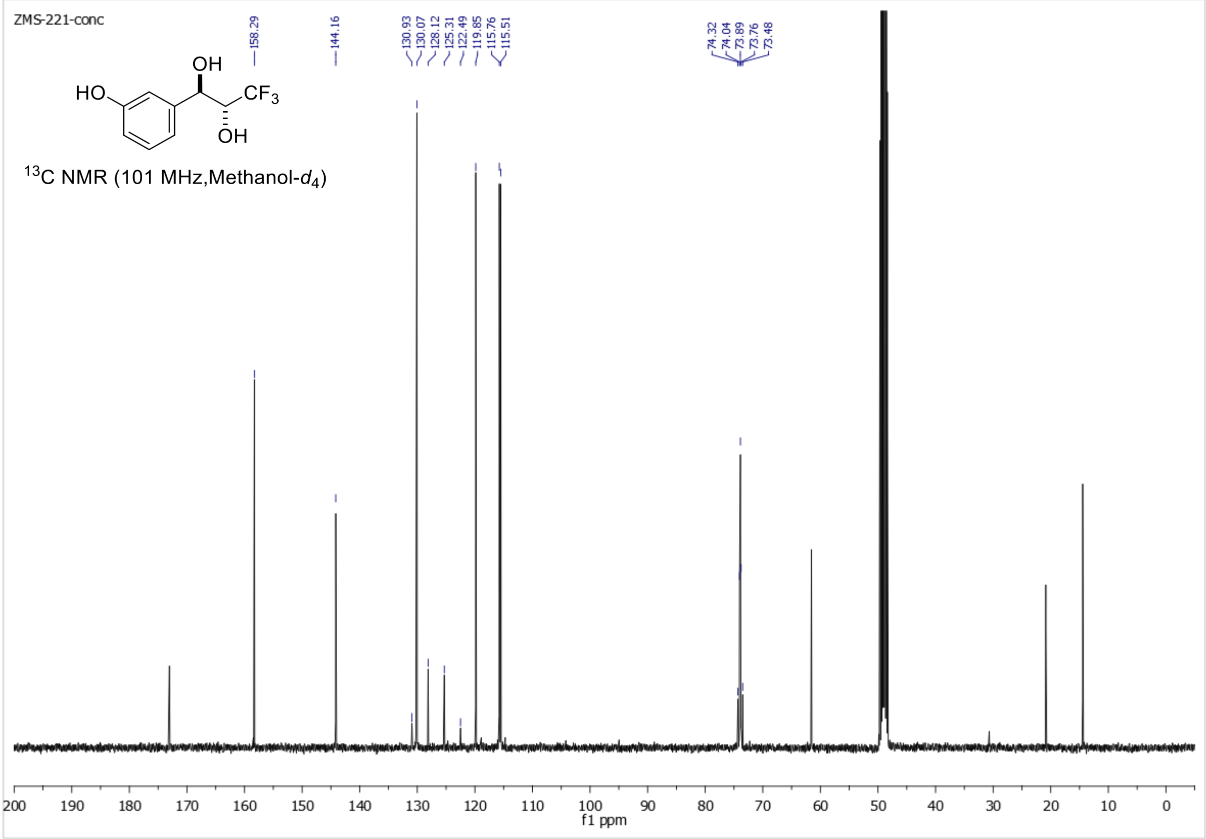

Compound ***syn*-3i**, ^{1}H NMR:

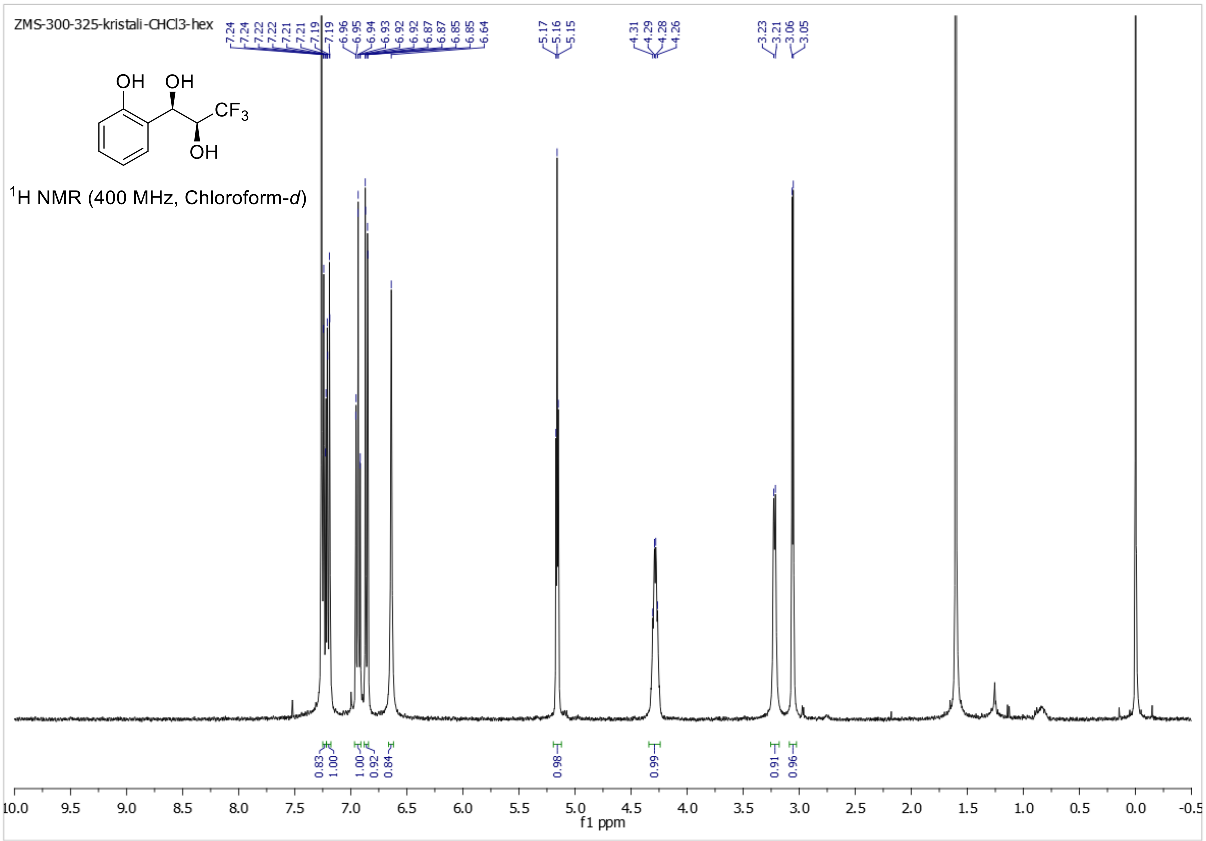

Compound ***syn*-3i**, ^{19}F NMR:

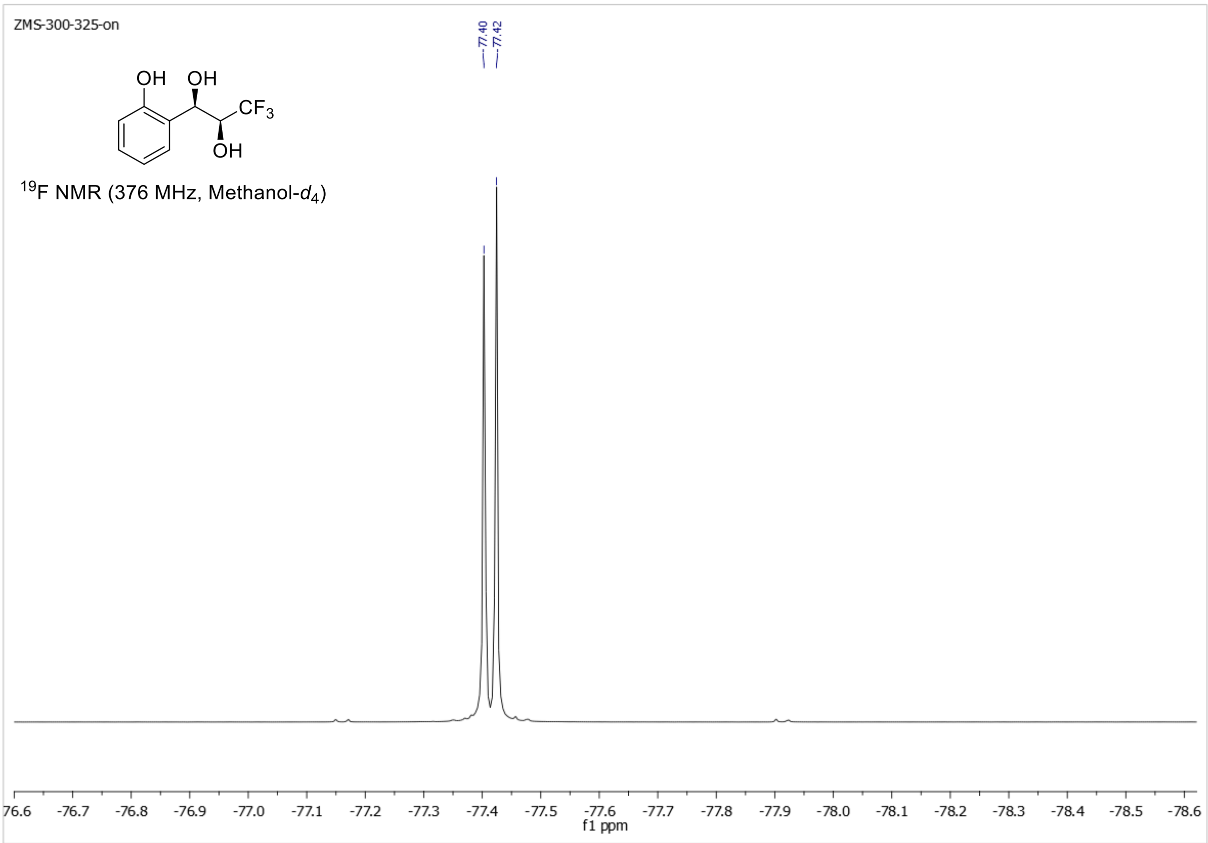

Compound ***syn*-3i**, ^{13}C NMR:

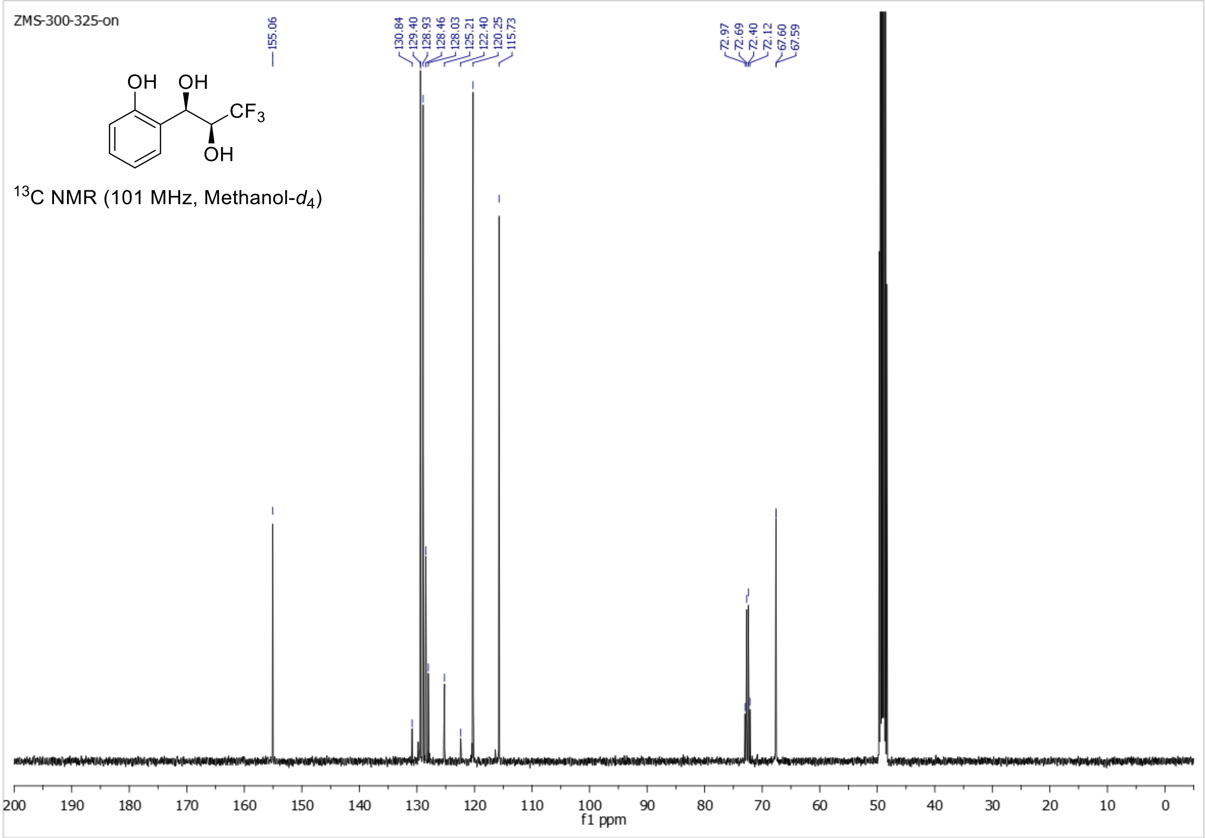

Compound **(±)-*anti*-3i**, ^{1}H NMR:

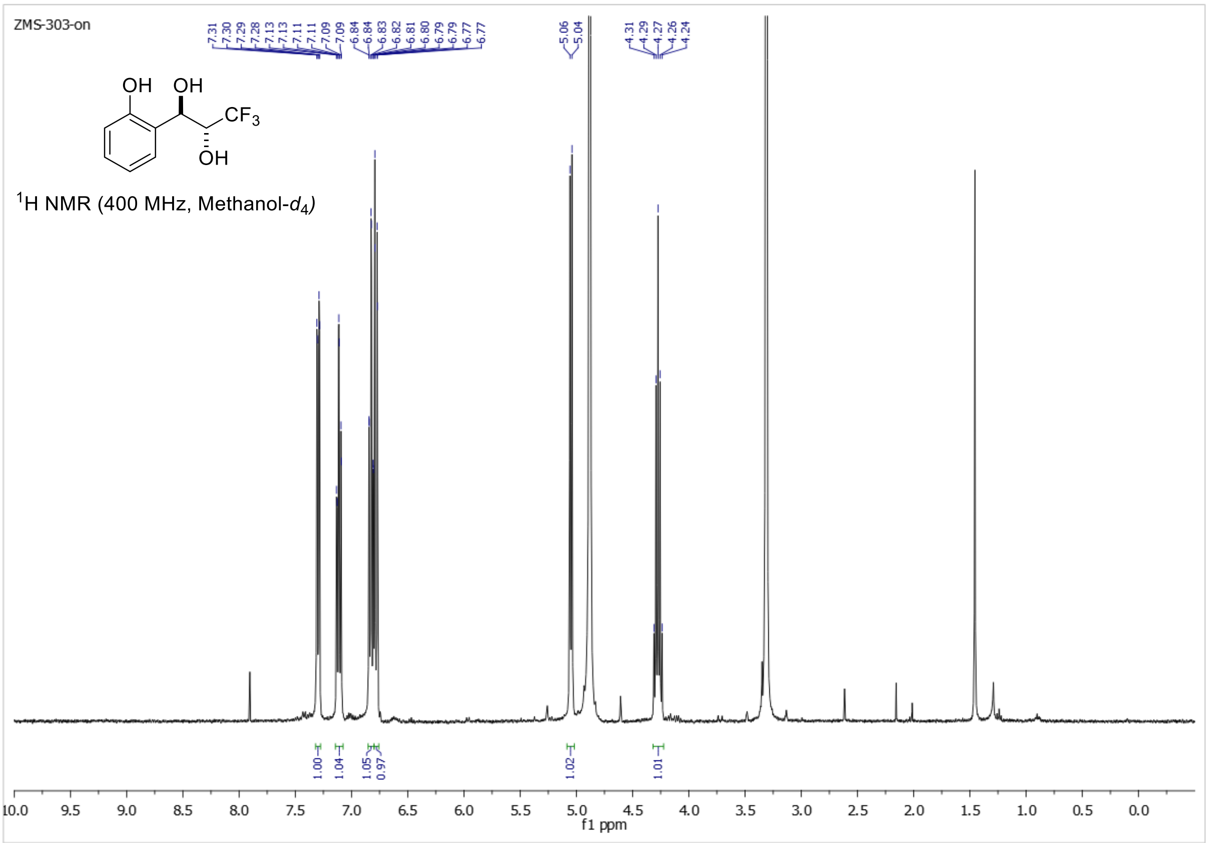

Compound **(±)-*anti*-3i**, ^{19}F NMR:

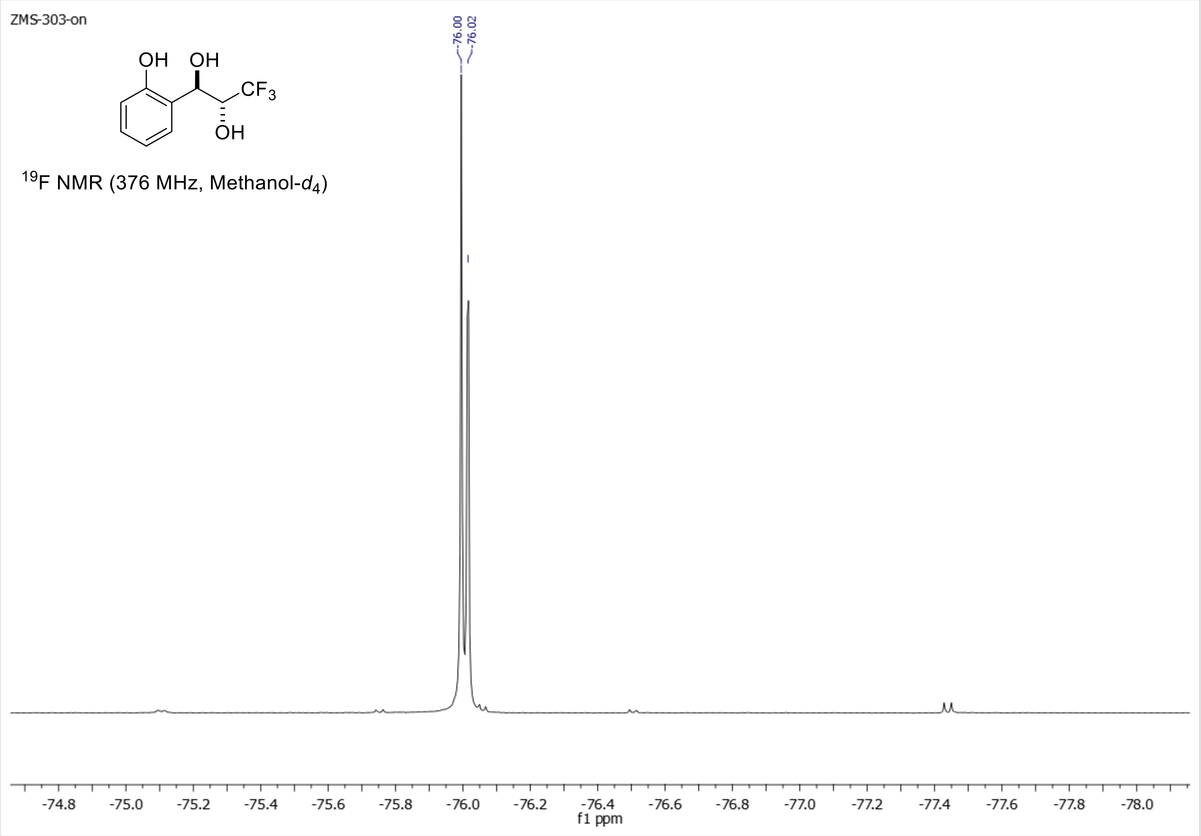

Compound **(±)-*anti*-3i**, ^{13}C NMR:

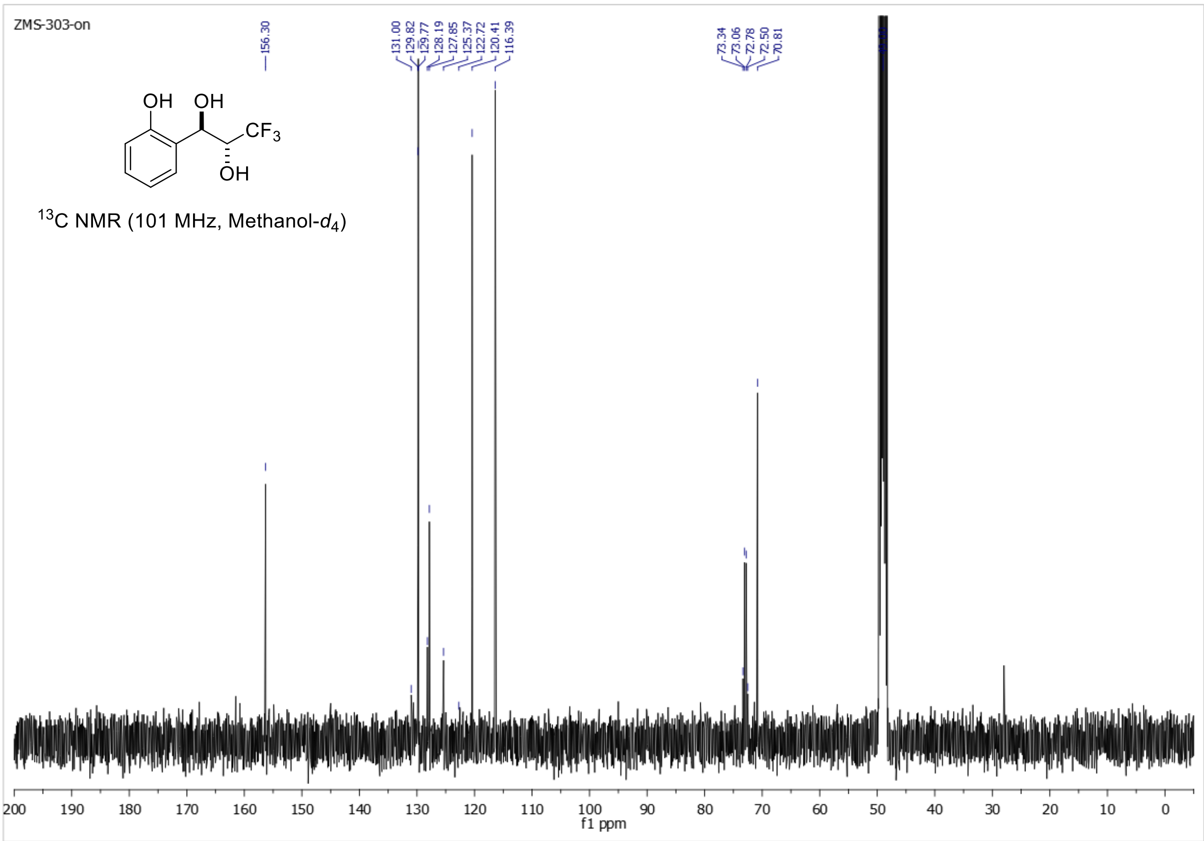

Compound ***syn*-3j**, ^{1}H NMR:

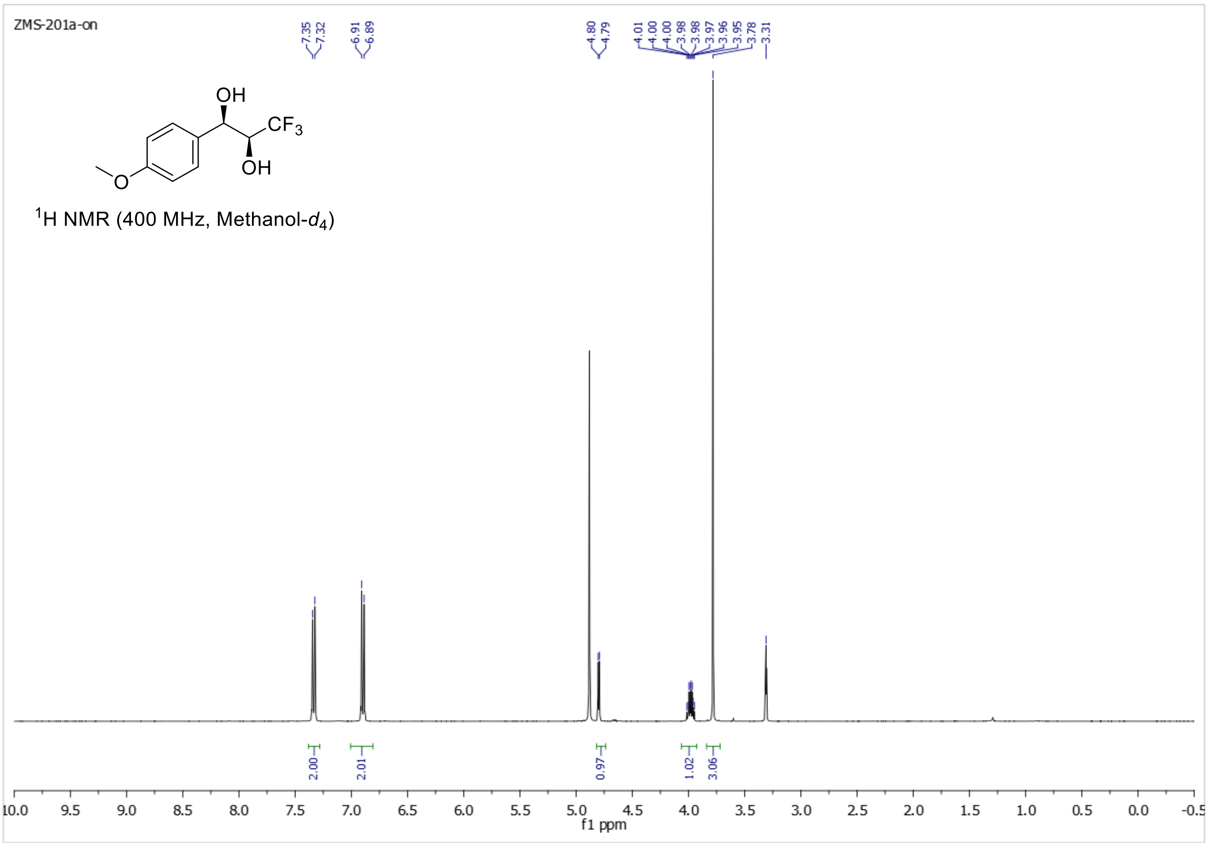

Compound ***syn*-3a**, ^{19}F NMR:

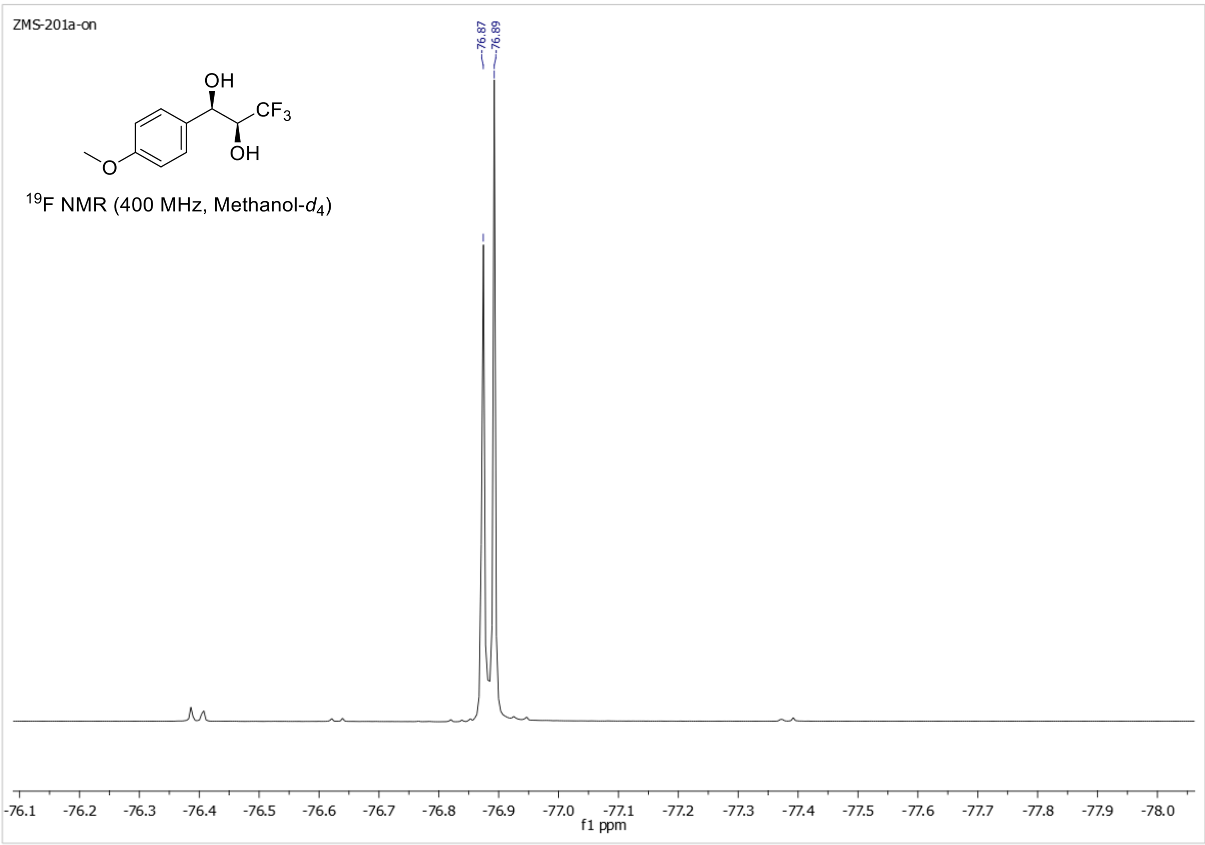

Compound ***syn*-3j**, ^{13}C NMR:

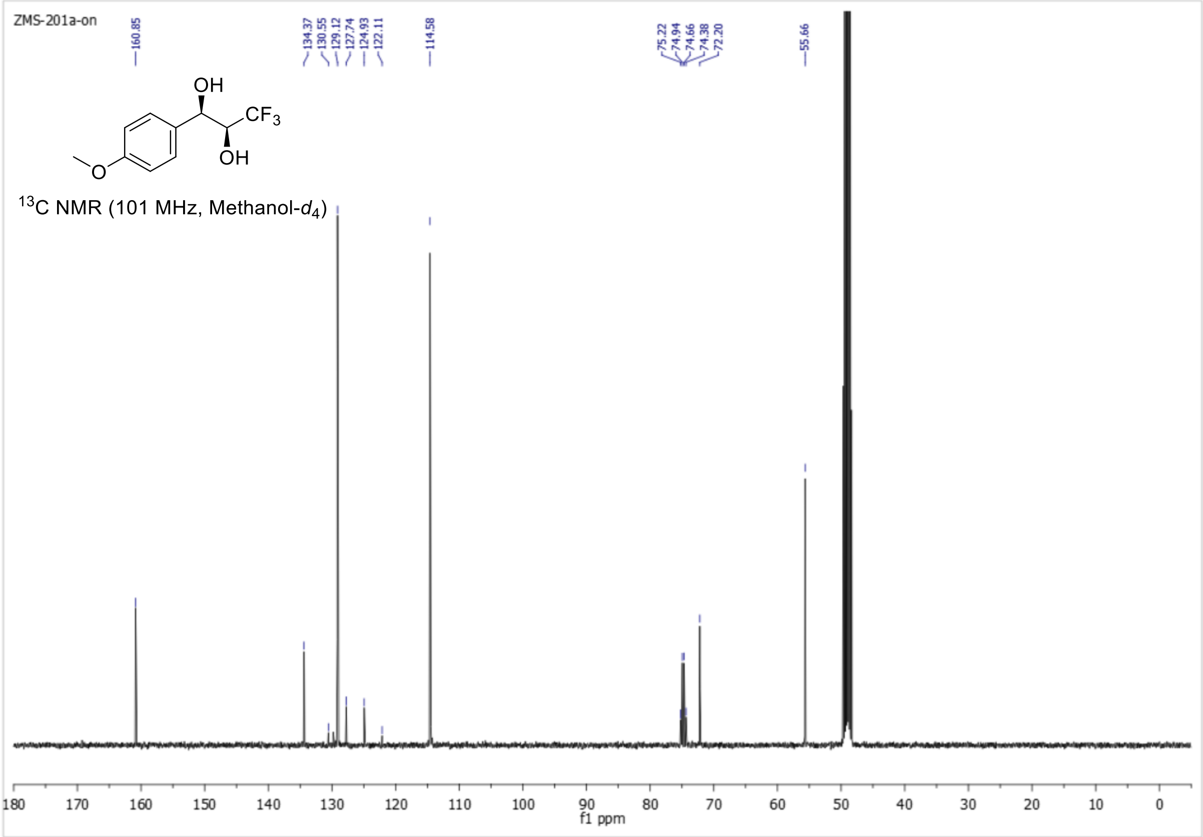

Compound **(±)-*anti*-3j**, ^{1}H NMR:

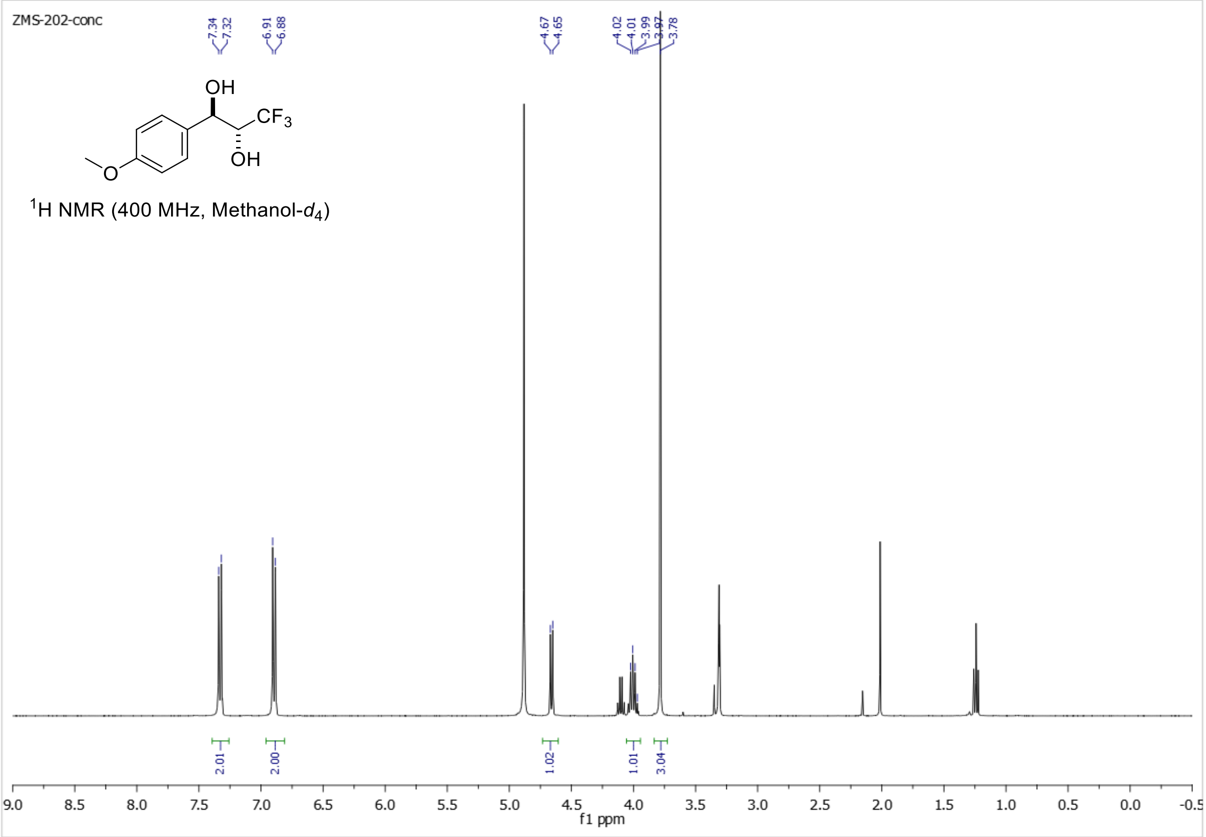

Compound **(±)-*anti*-3j**, ^{19}F NMR:

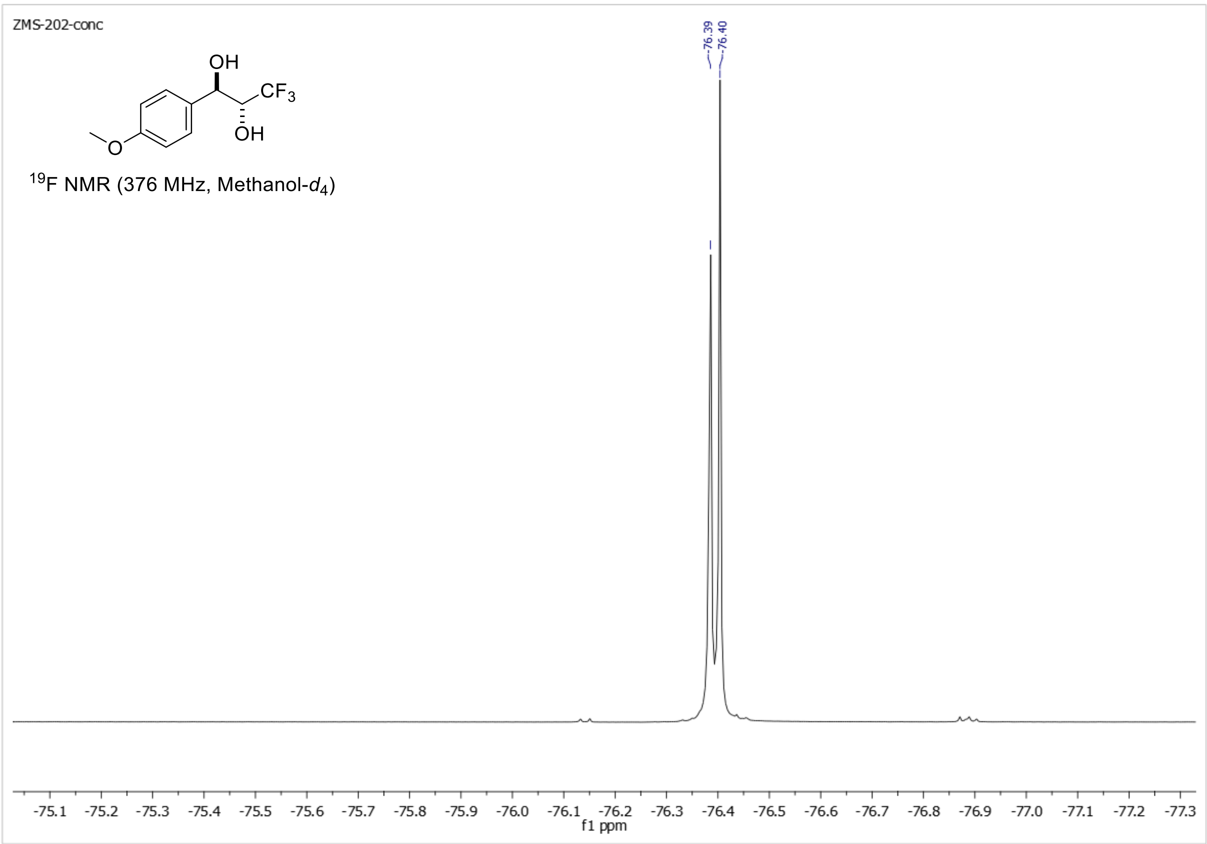

Compound **(±)-*anti*-3j**, ^{13}C NMR:

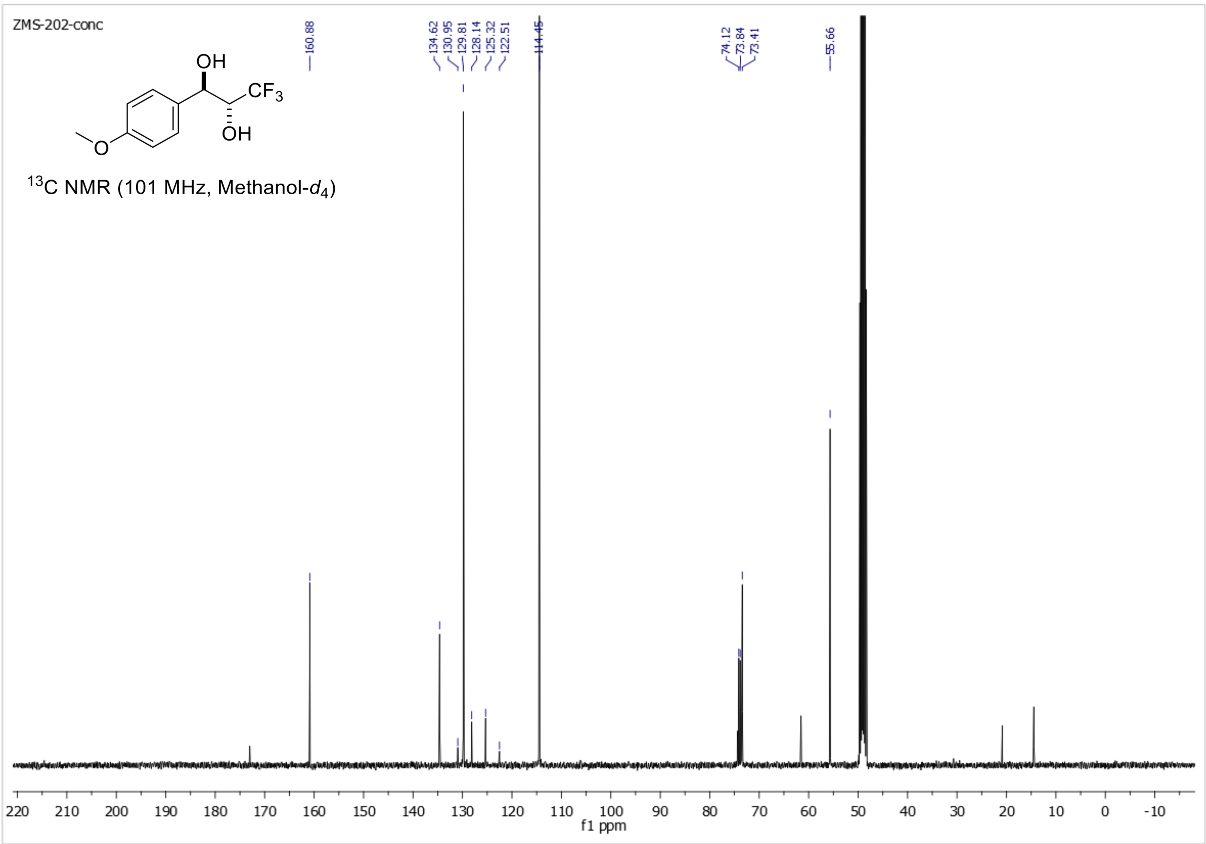

Compound ***syn*-3k**, ^{1}H NMR:

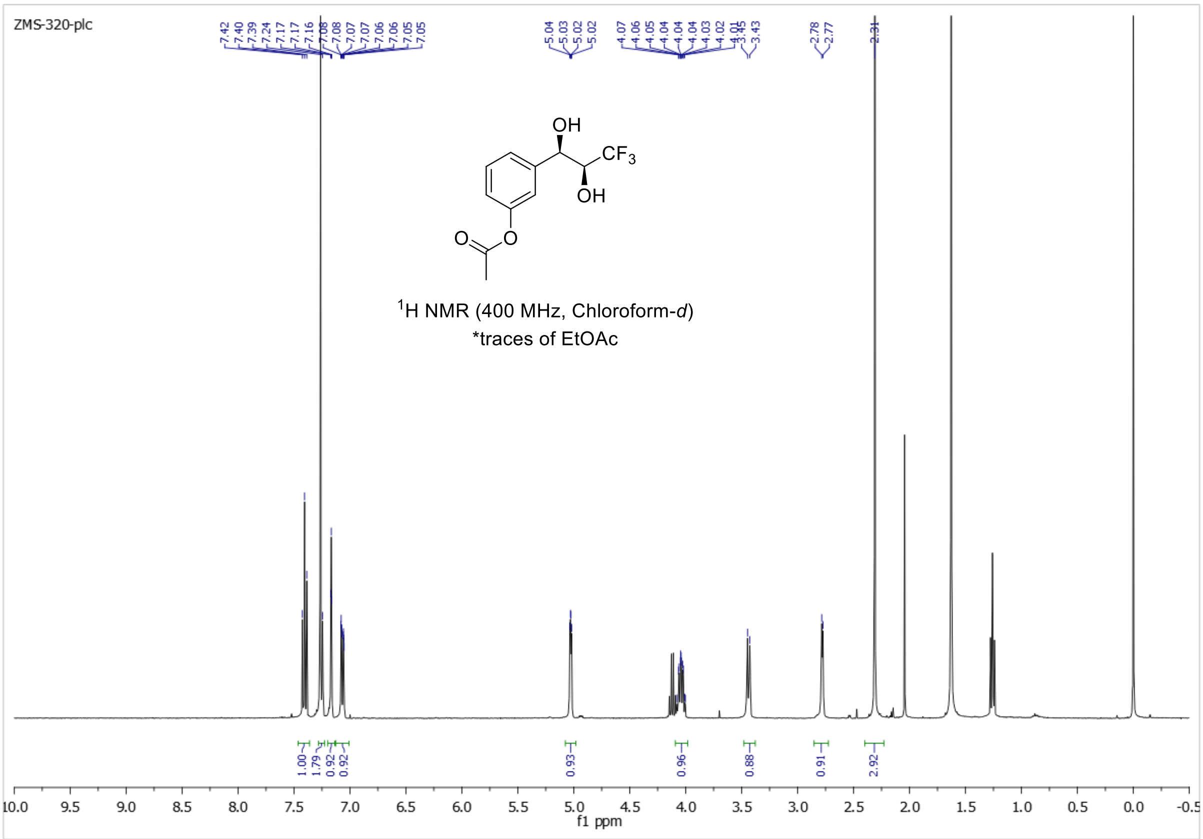

Compound ***syn*-3k**, ^{19}F NMR:

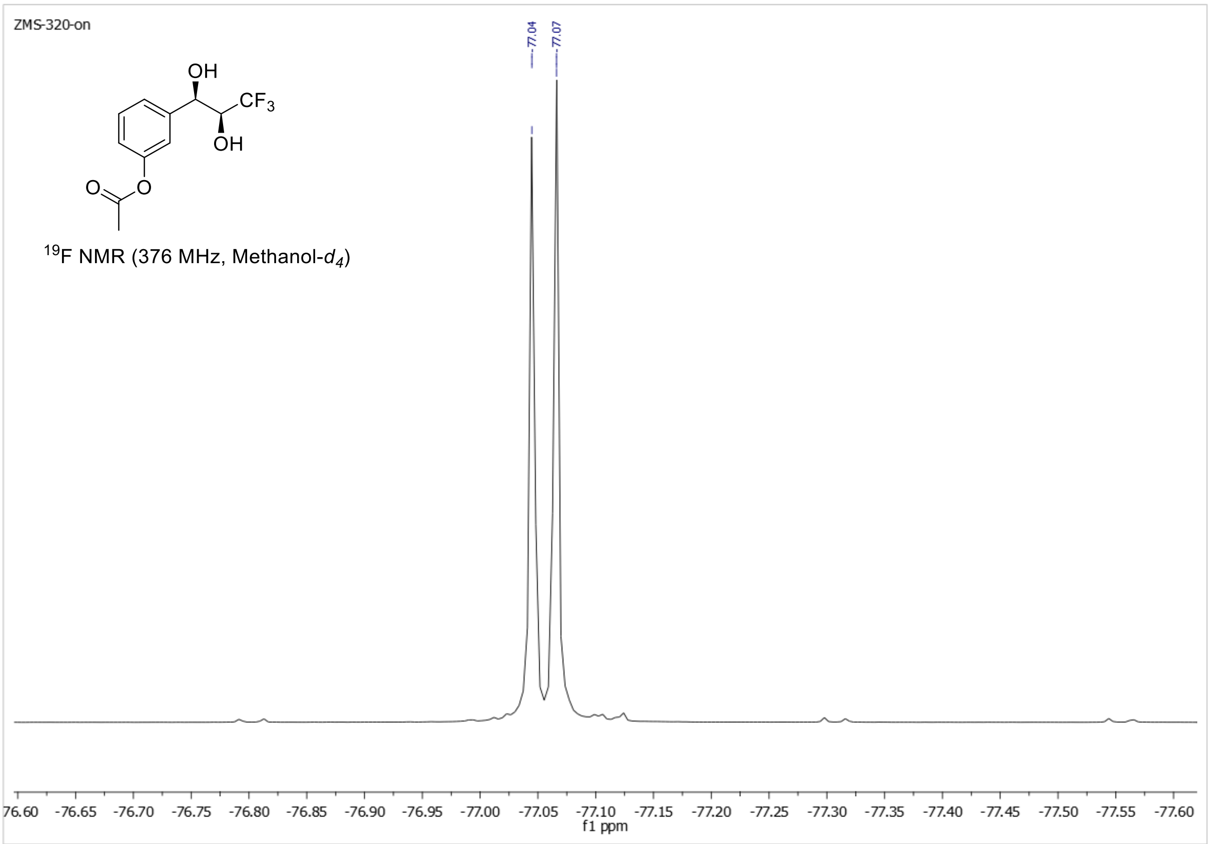

Compound ***syn*-3k**, ^{13}C NMR:

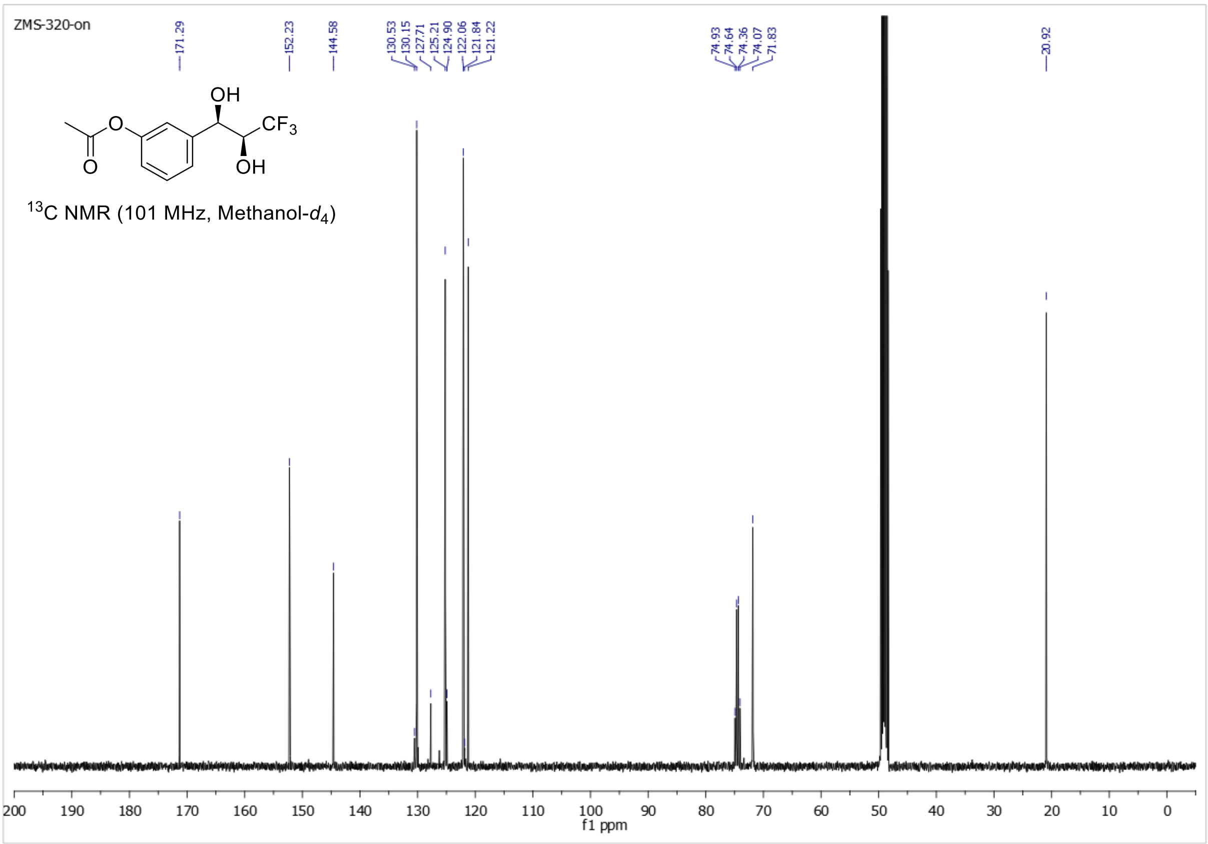

Compound ***syn*-3l**, ^{1}H NMR:

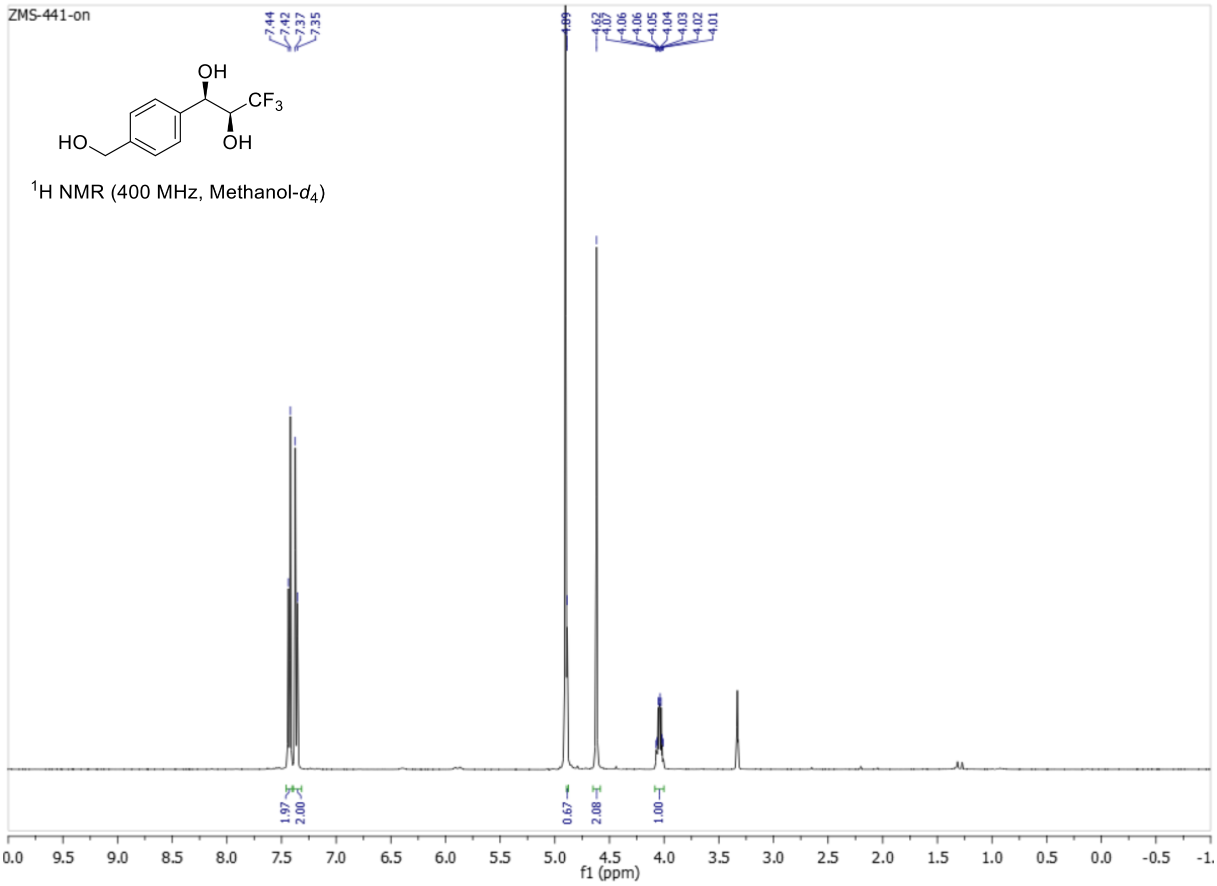

Compound ***syn*-3l**, ^{19}F NMR:

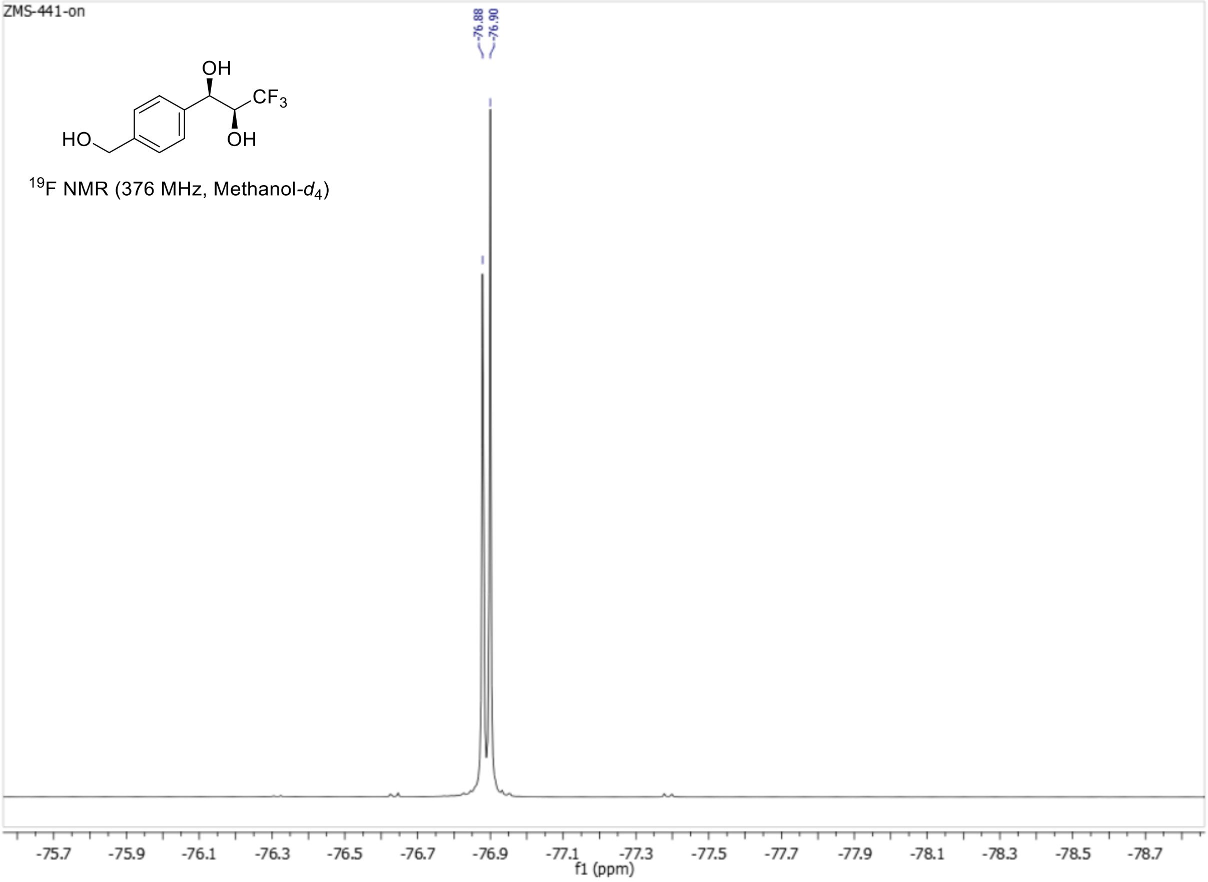

Compound ***syn*-3l**, ^{13}C NMR:

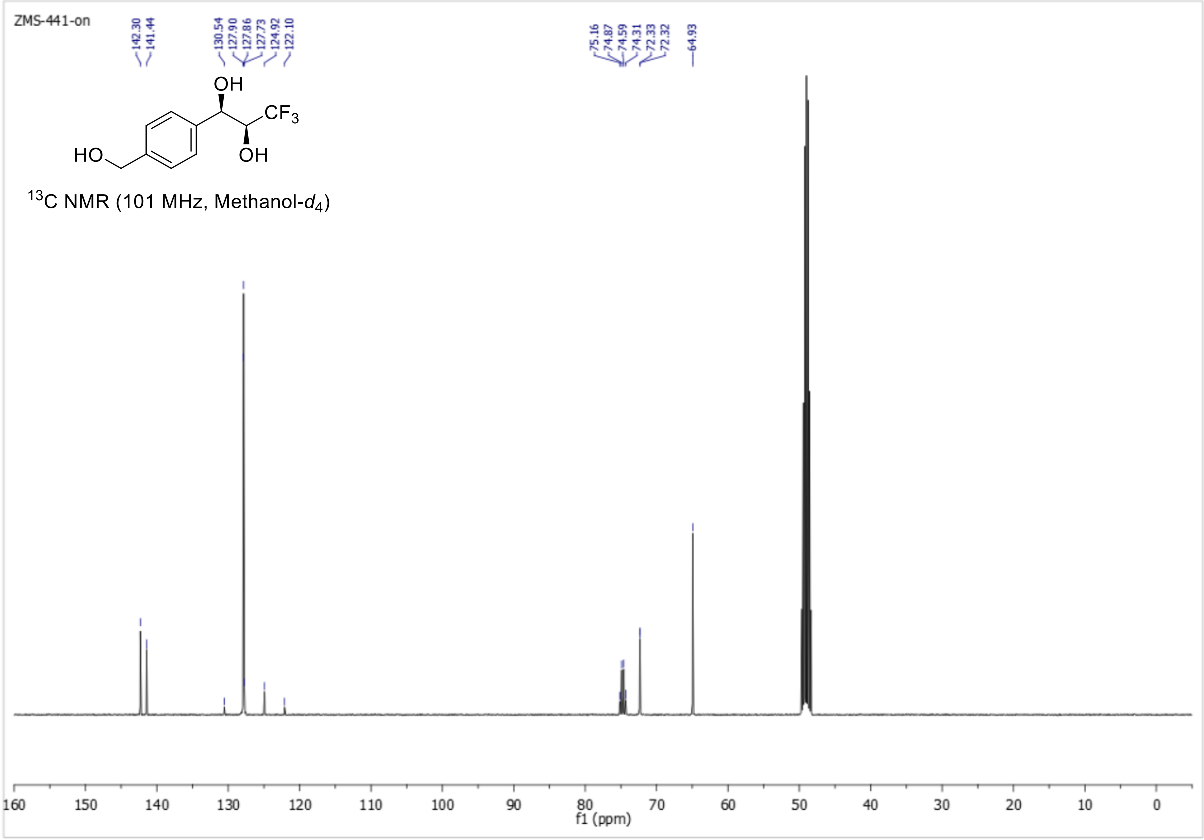

Compound **(±)-*anti*-3l**, ^{1}H NMR:

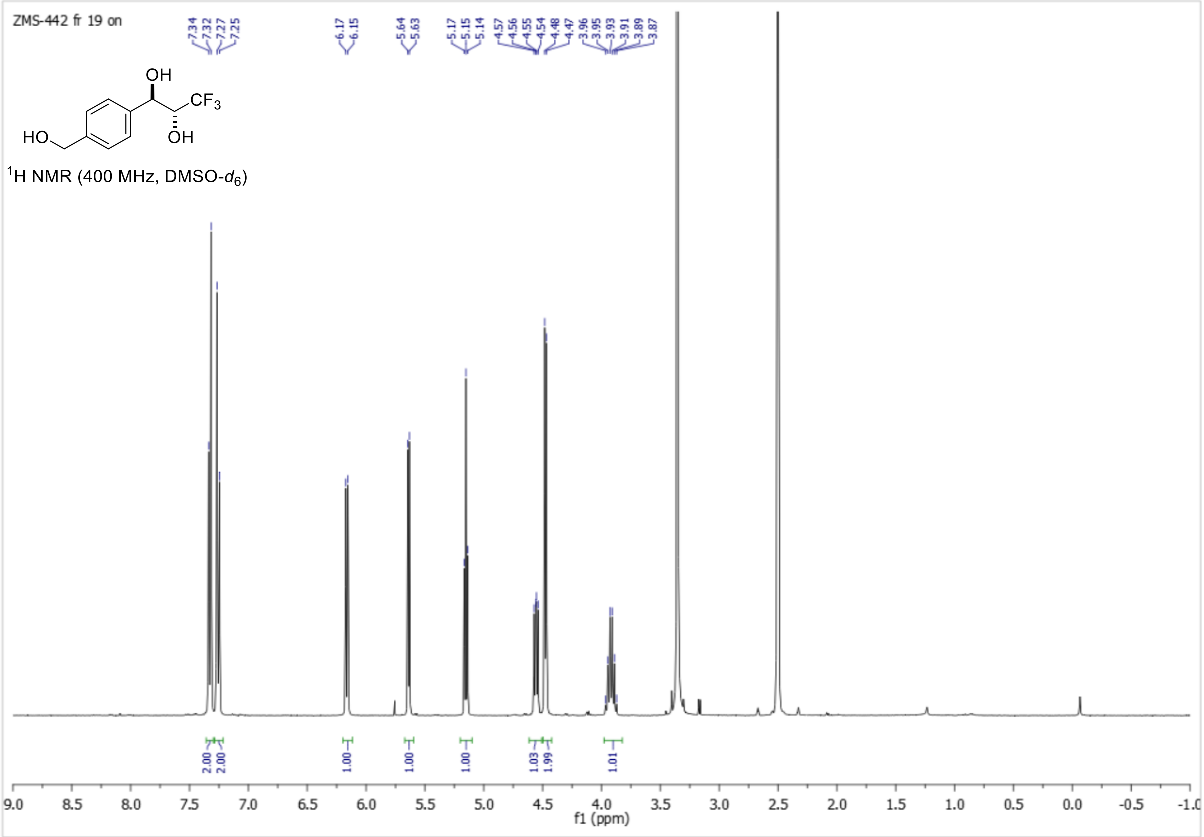

Compound **(±)-*anti*-3l**, ^{19}F NMR:

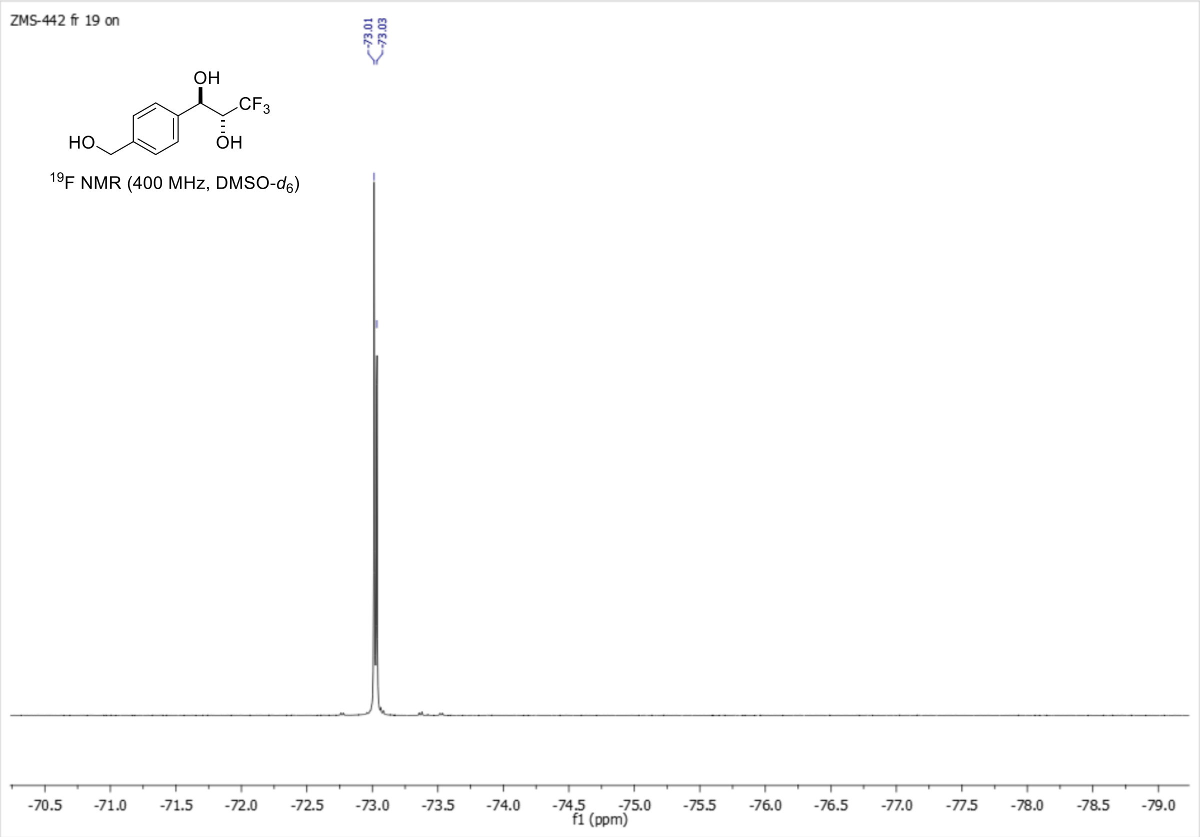

Compound **(±)-*anti*-3l**, ^{13}C NMR:

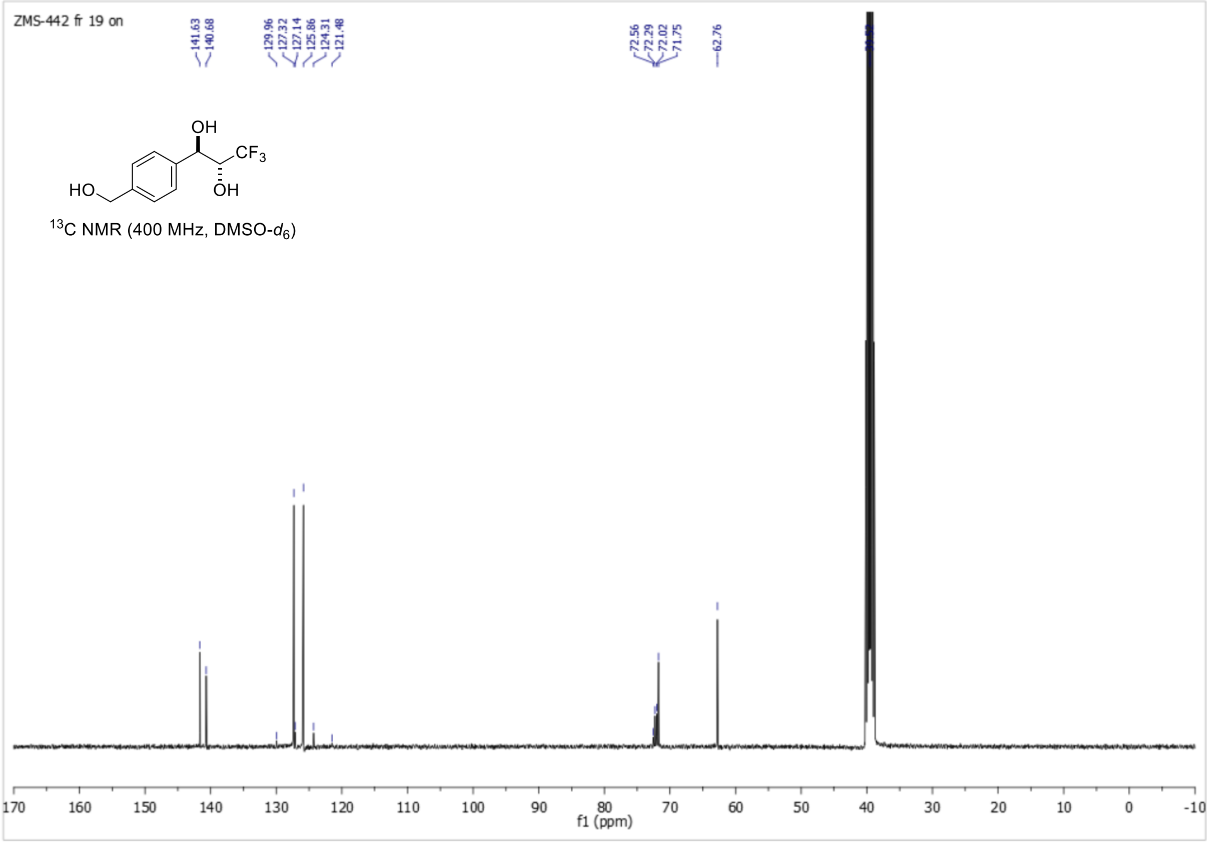

Compound ***syn*-3m**, ^{1}H NMR:

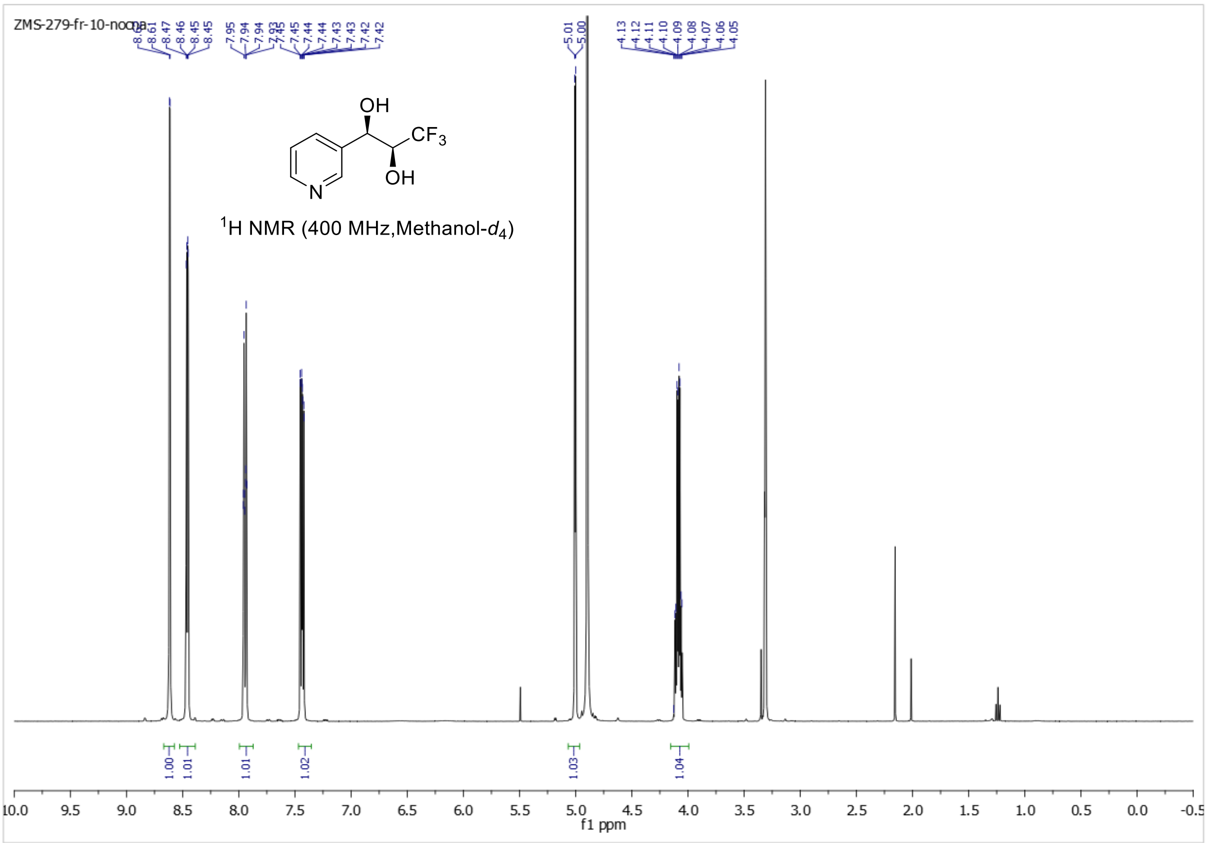

Compound ***syn*-3m**, ^{19}F NMR:

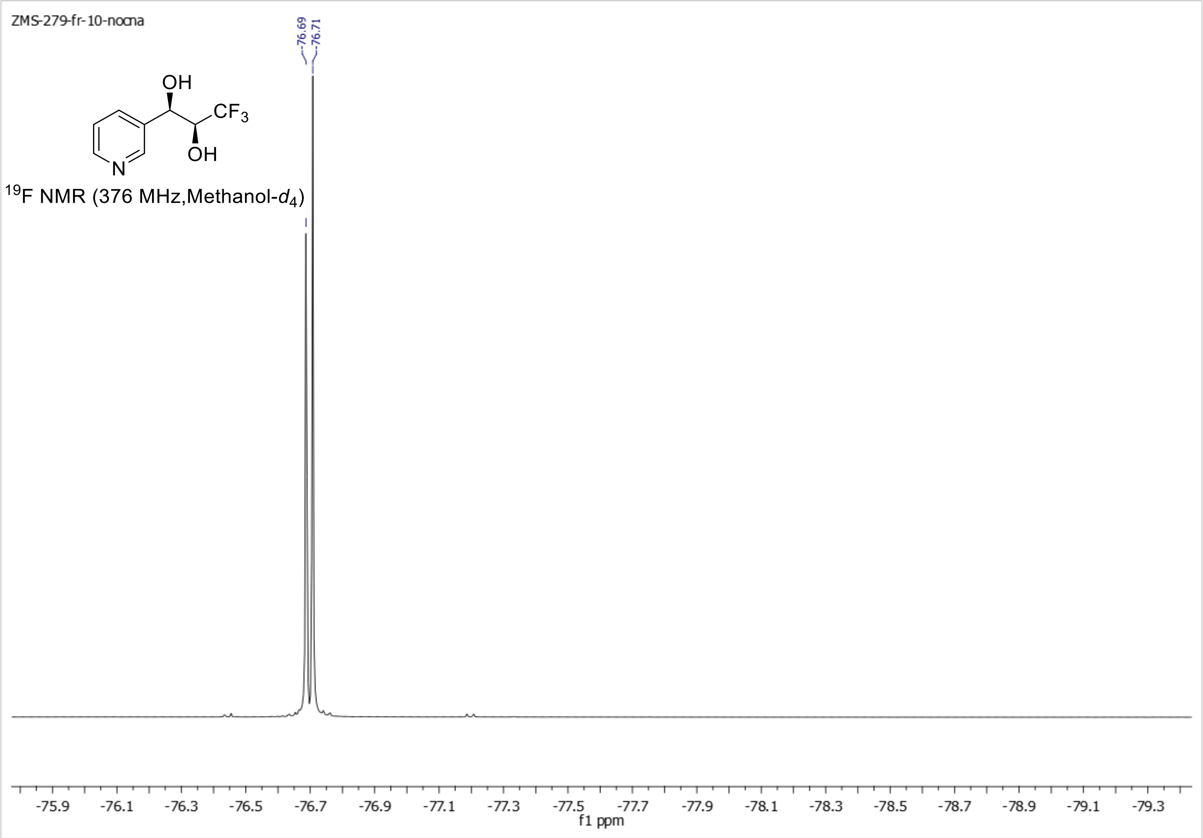

Compound ***syn*-3m**, ^{13}C NMR:

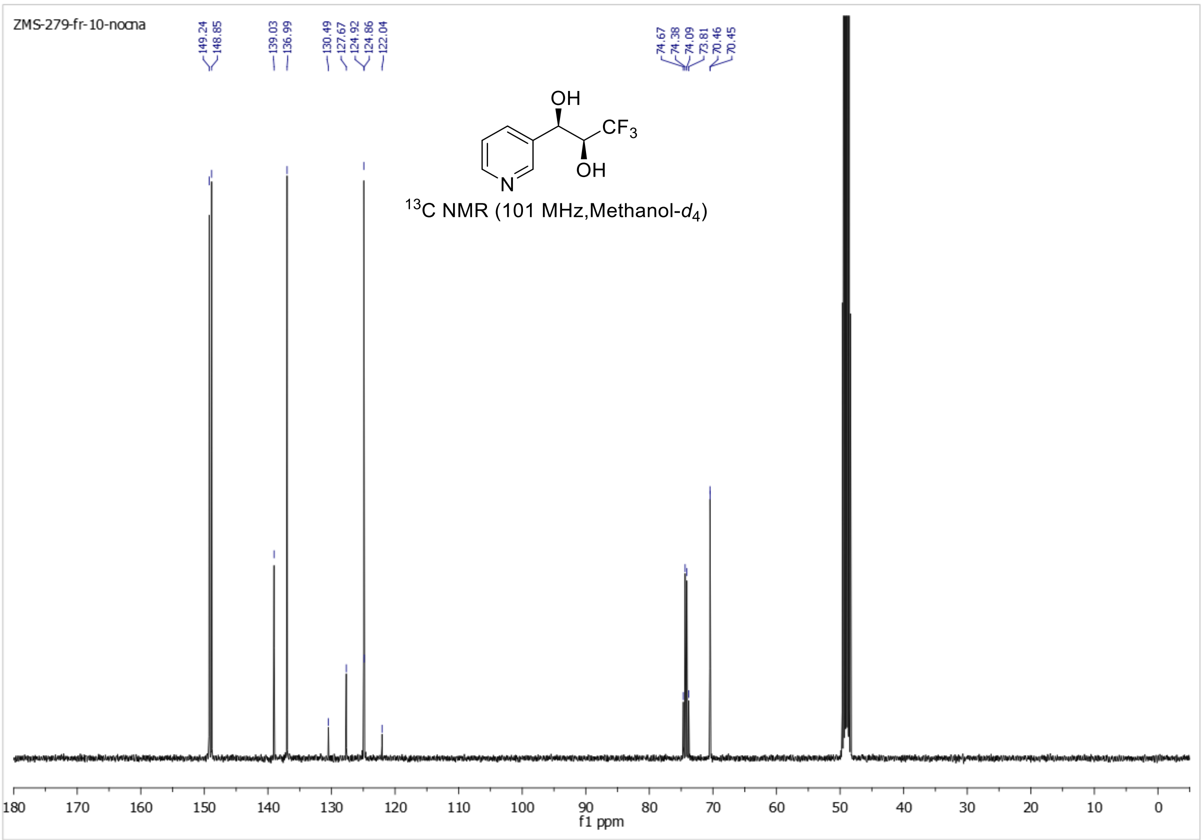

Compound ***syn*-3n**, ^{1}H NMR:

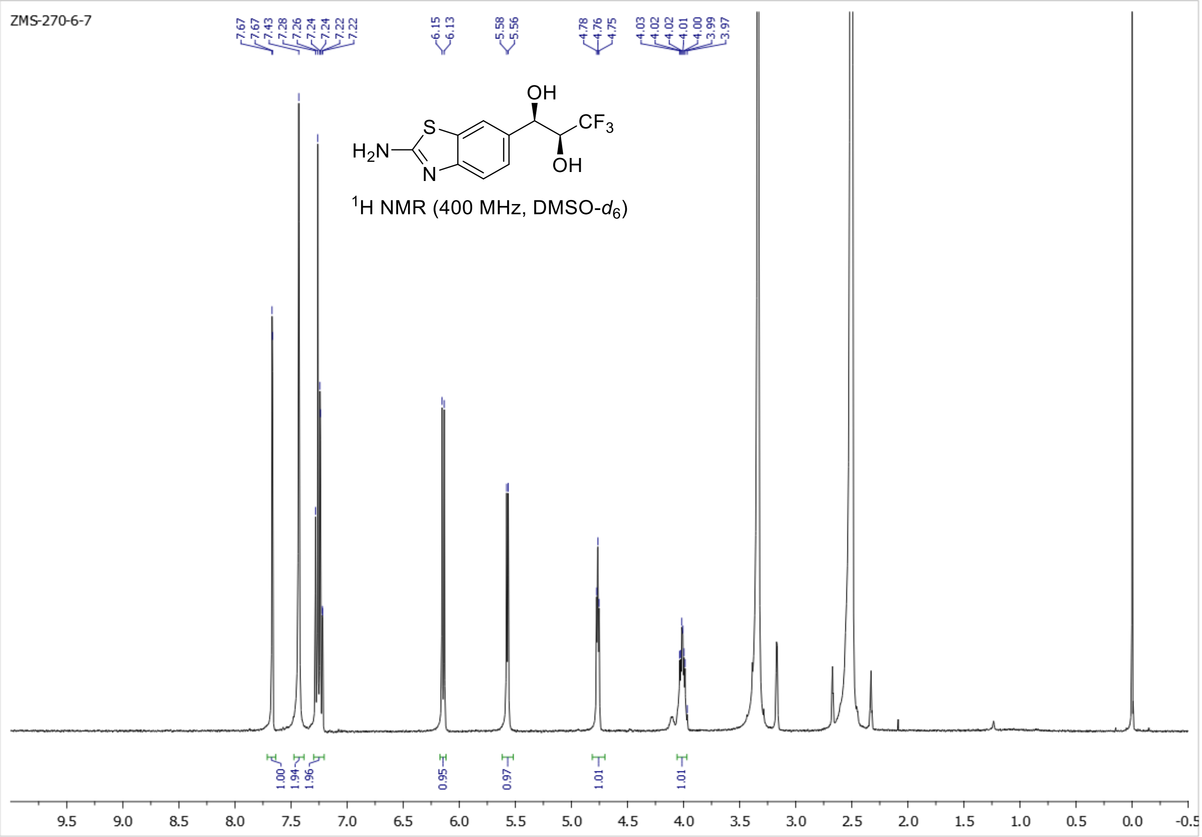

Compound ***syn*-3n**, ^{19}F NMR:

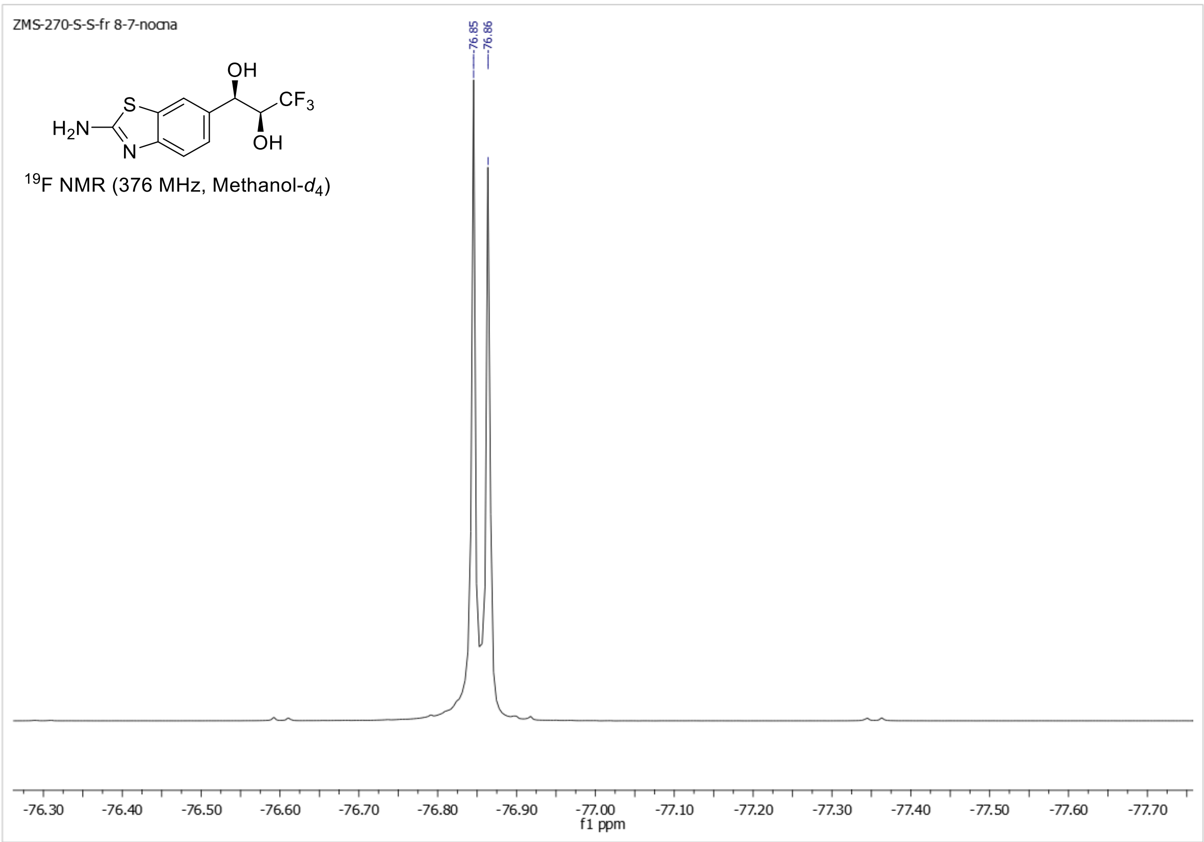

Compound ***syn*-3n**, ^{13}C NMR:

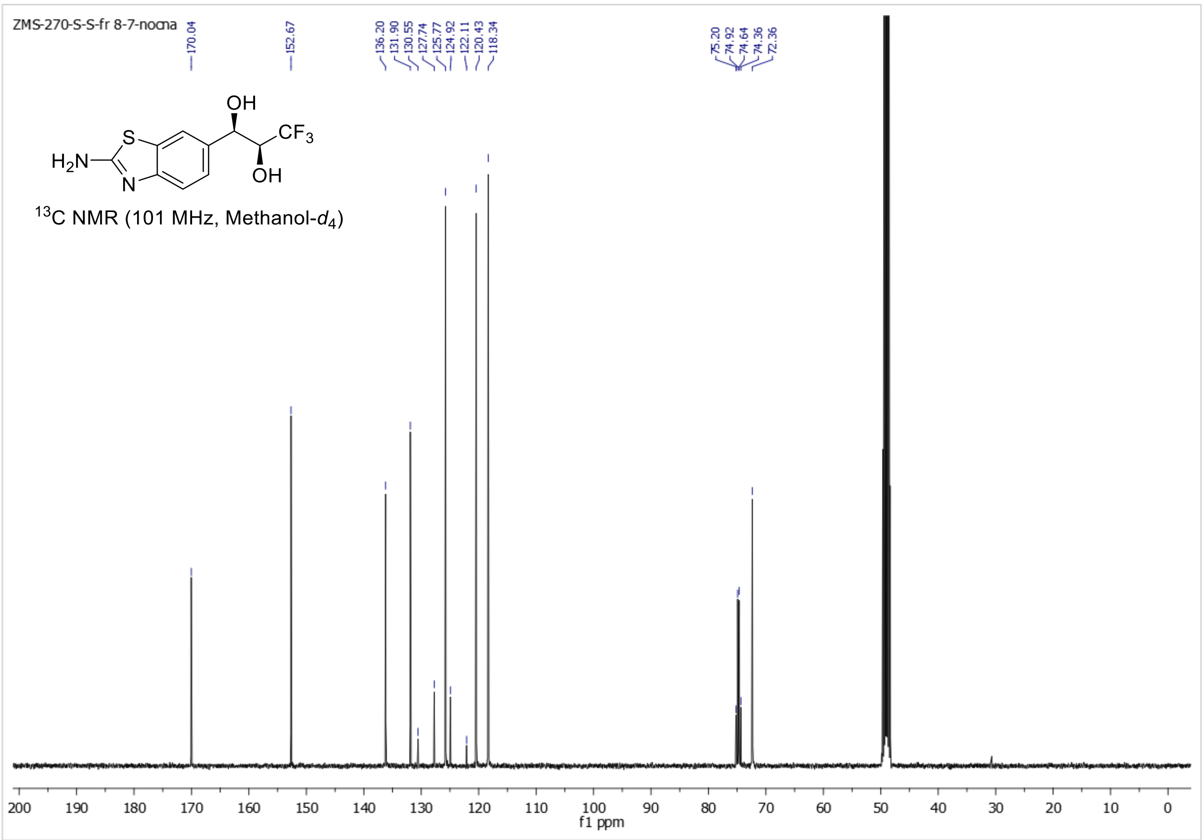

Compound ***ent*-*syn*-3n**, ^{1}H NMR:

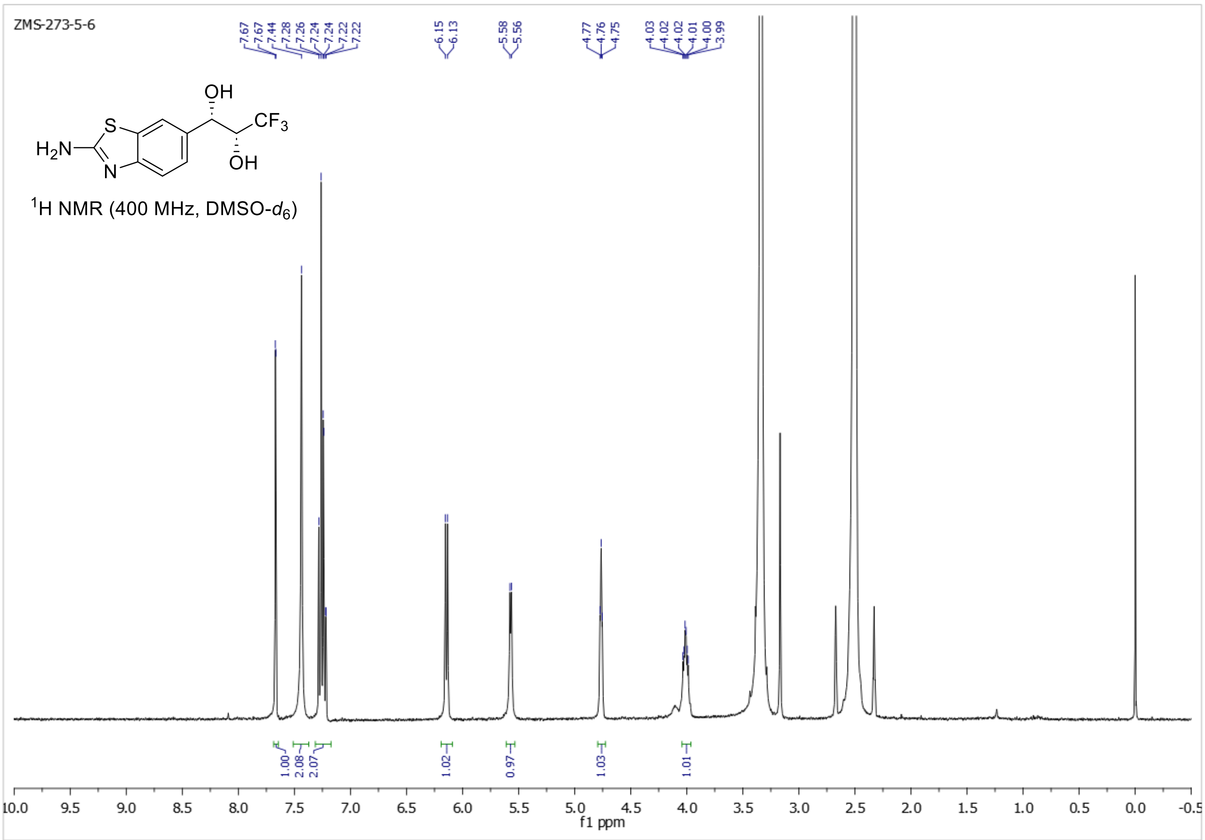

Compound **(±)-*anti*-3n**, ^{1}H NMR:

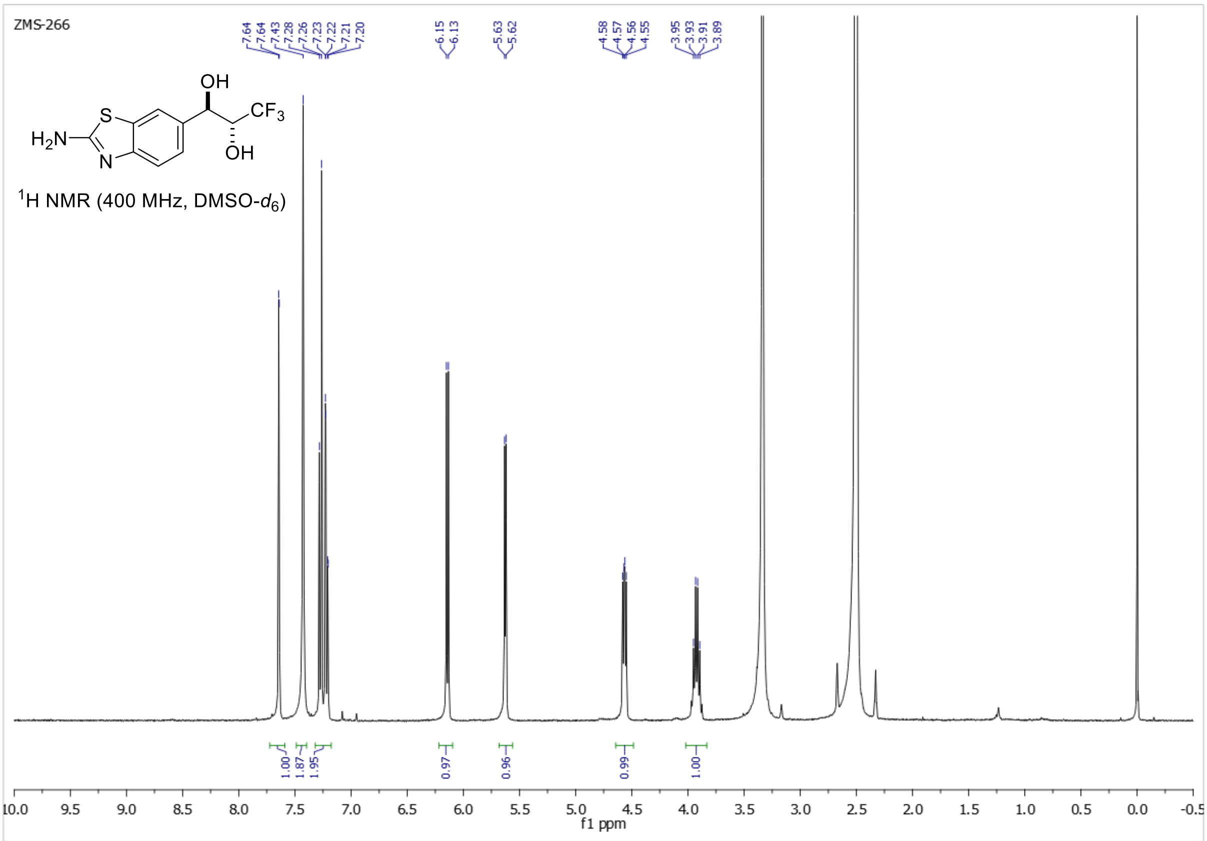

Compound **(±)-*anti*-3n**, ^{19}F NMR:

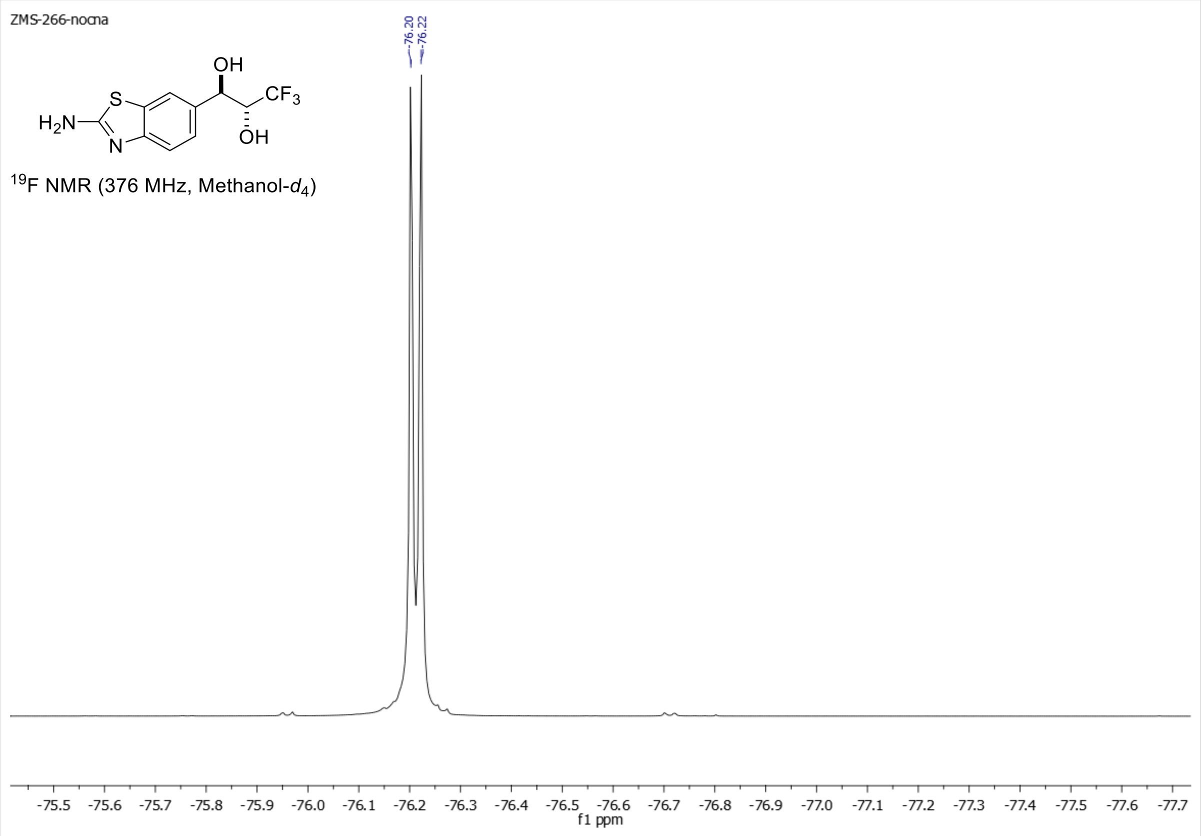

Compound **(±)-*anti*-3n**, ^{13}C NMR:

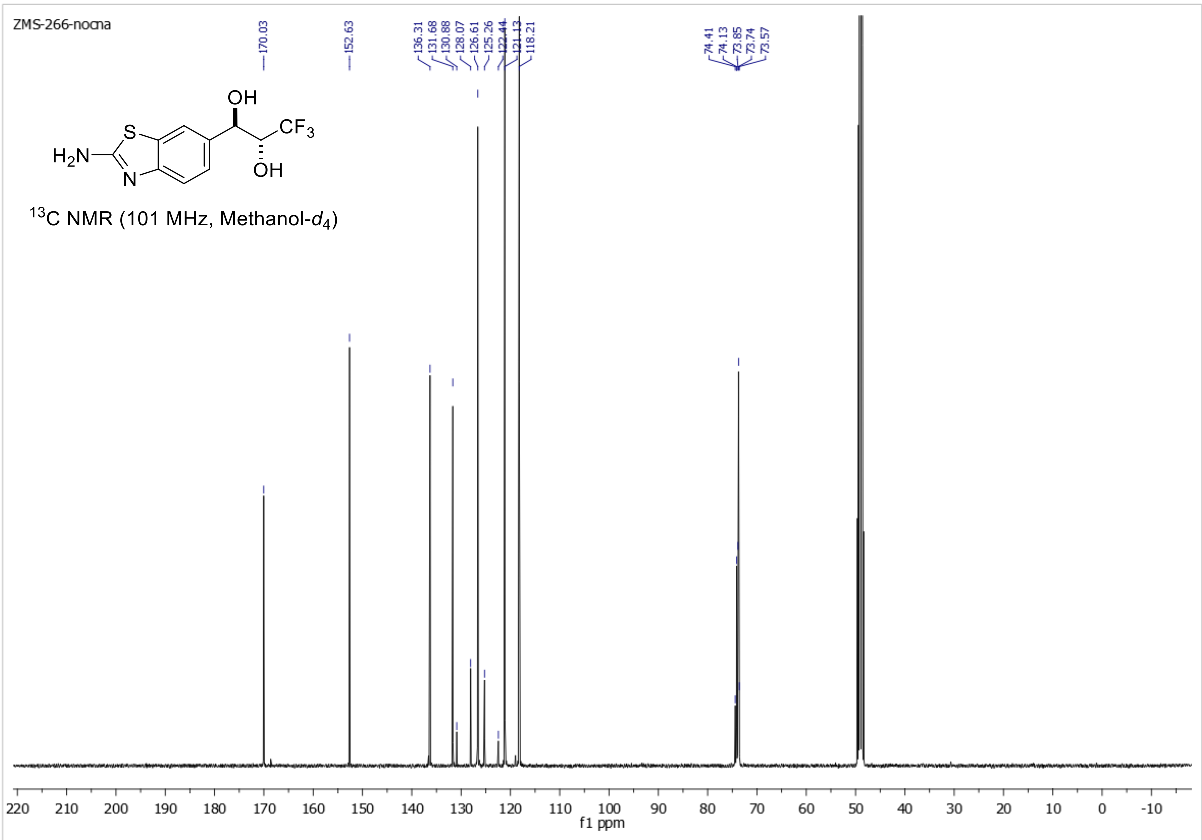

Compound ***syn*-3o,** ^{1}H NMR:

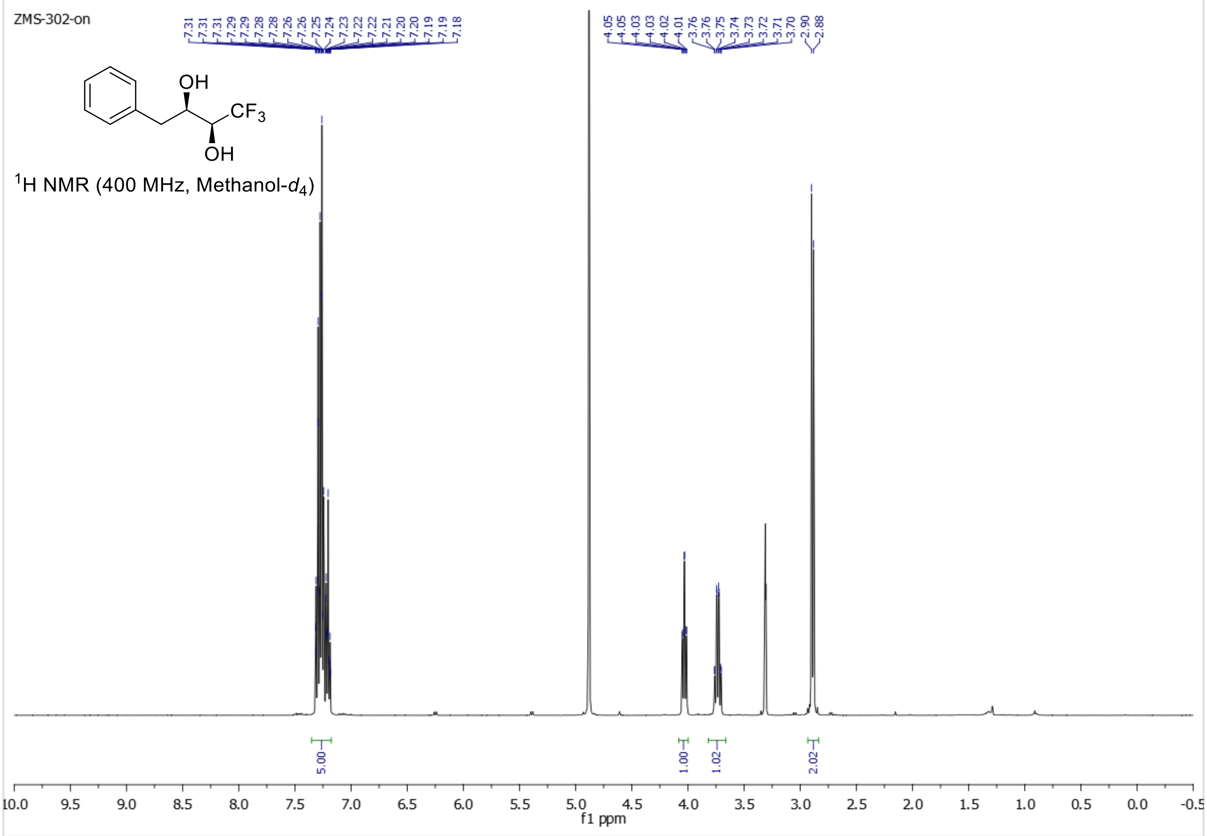

Compound ***syn*-3o**,^{19}F NMR:

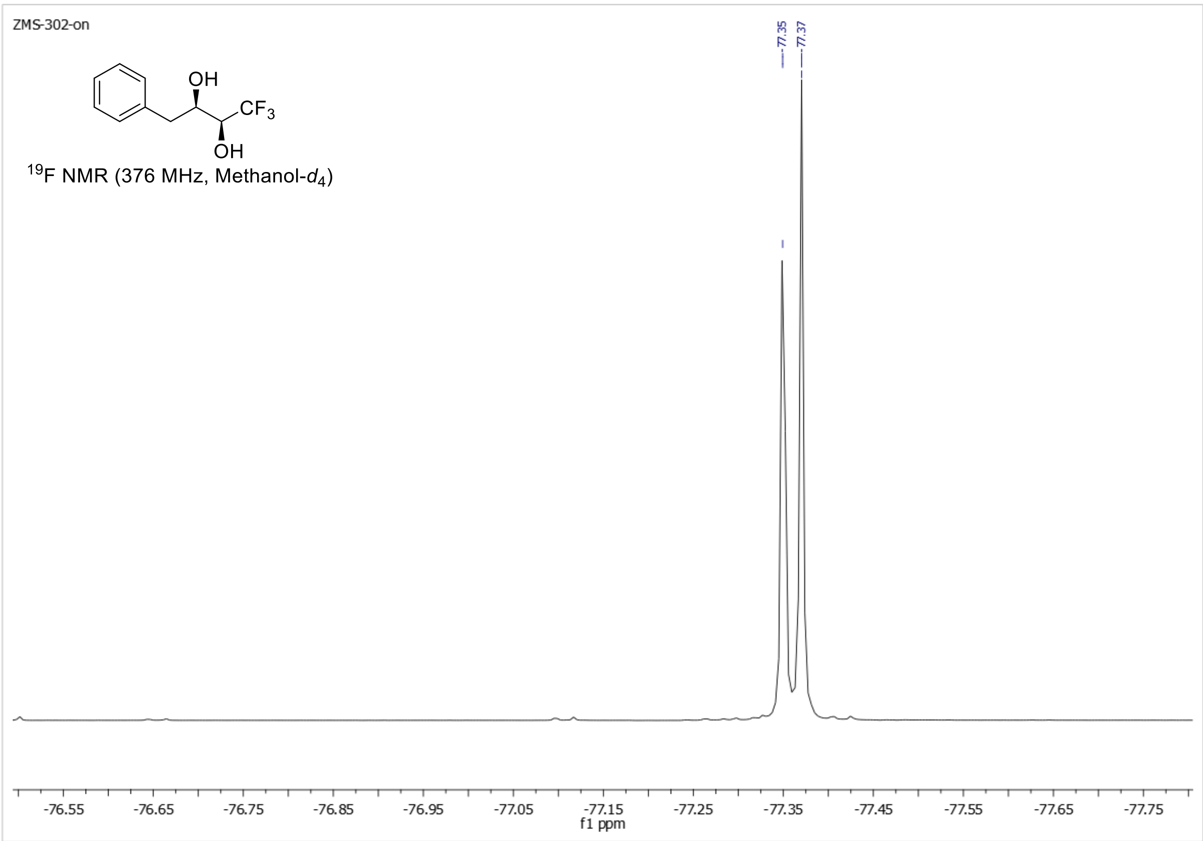

Compound ***syn*-3o**, ^{13}C NMR:

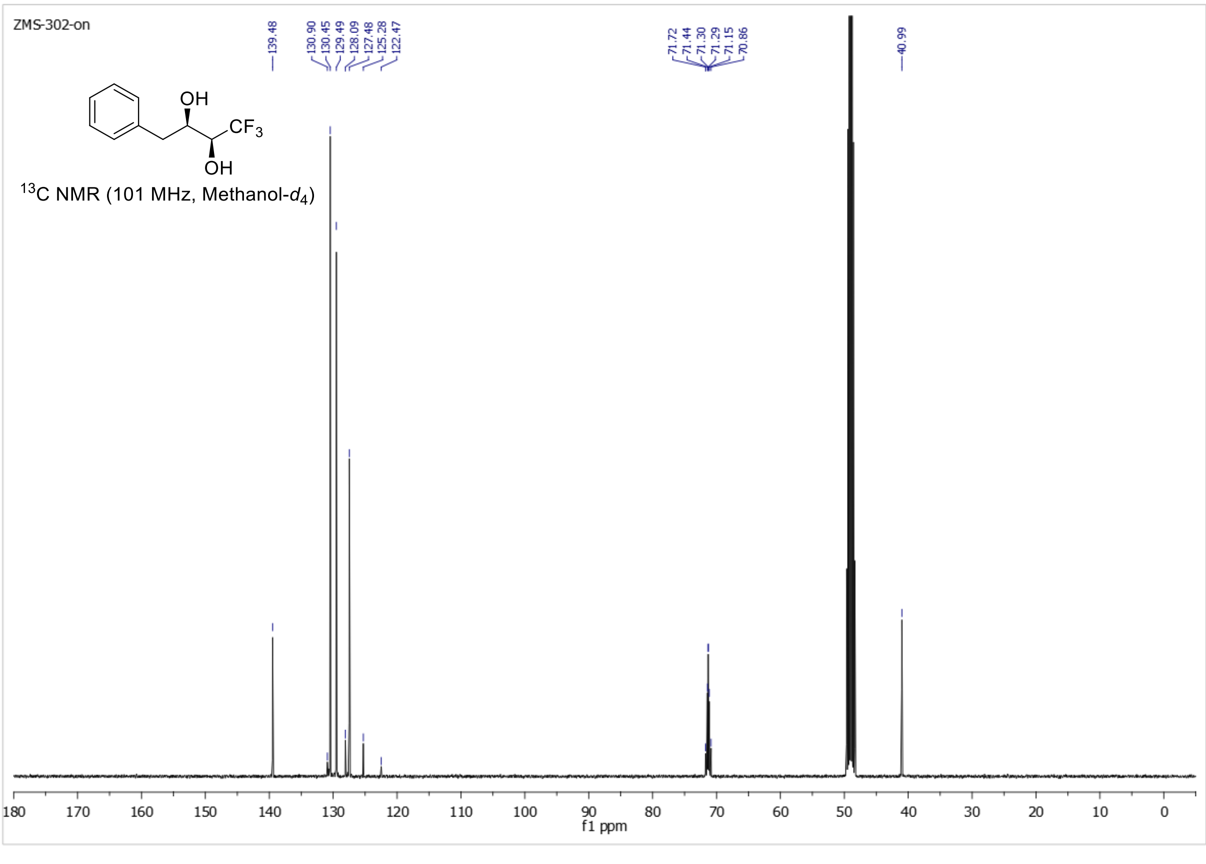

Compound **(±)-*anti*-3o**, ^{1}H NMR:

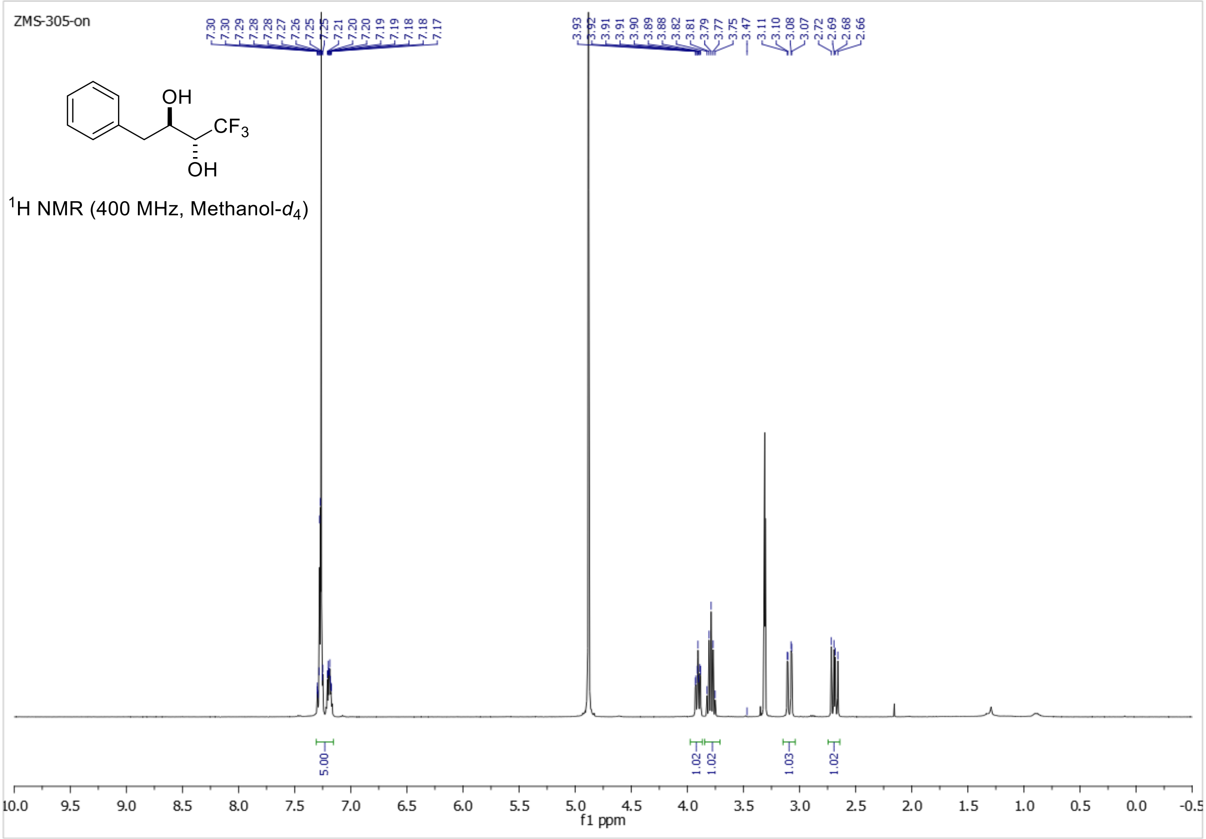

Compound **(±)-*anti*-3o**, ^{19}F NMR:

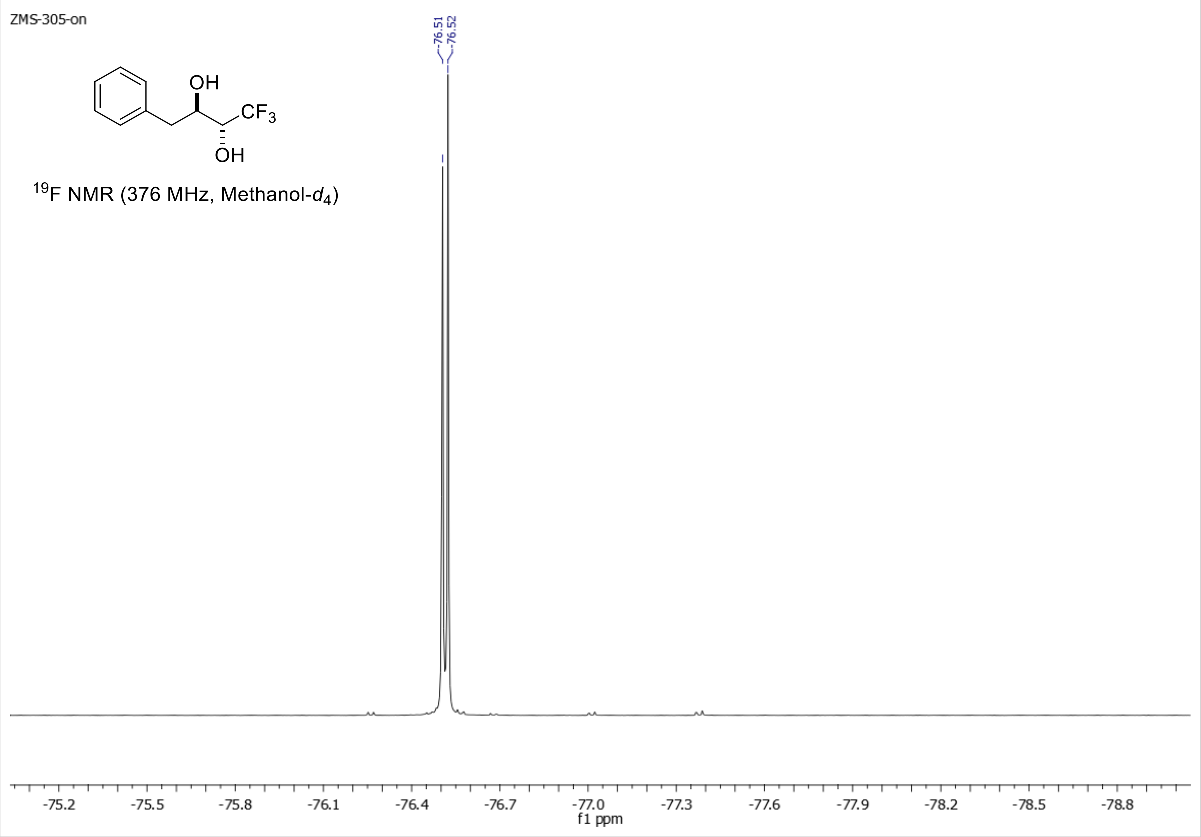

Compound **(±)-*anti*-3o**, ^{13}C NMR:

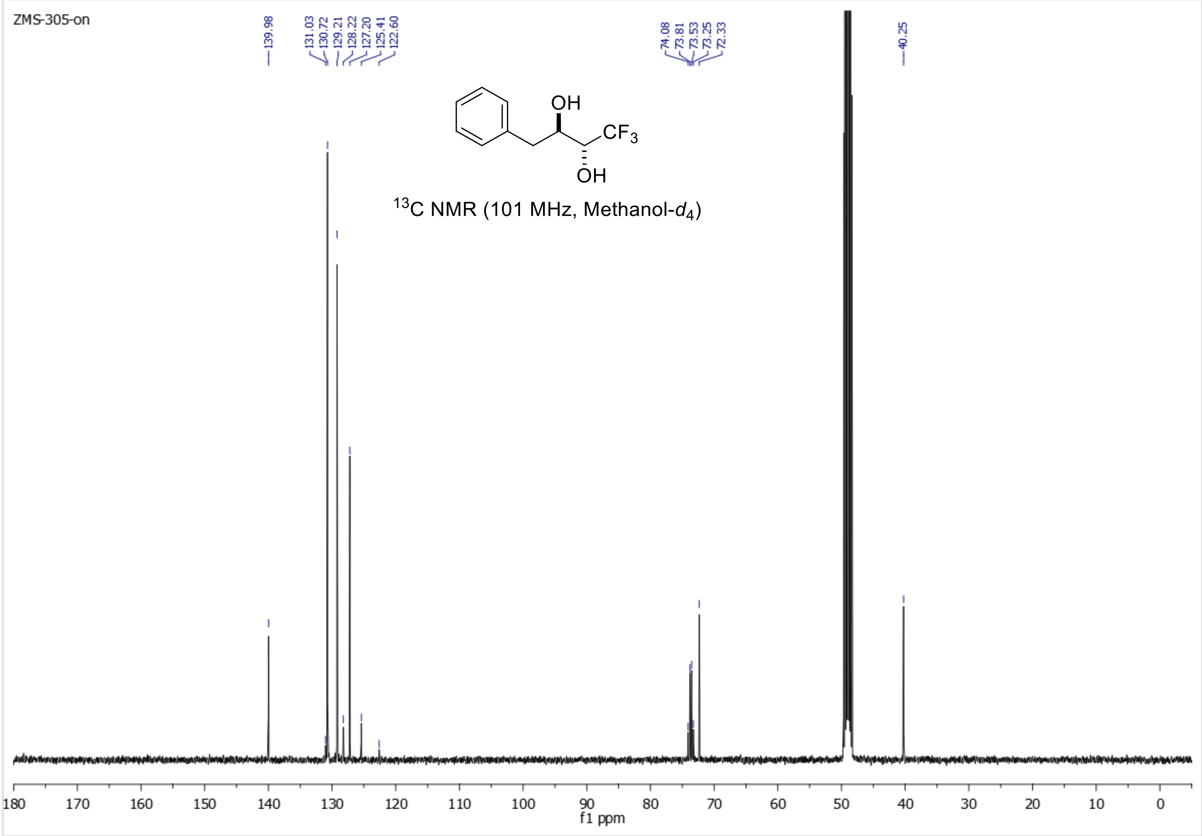

Compound ***syn*-3p**, ^{1}H NMR:

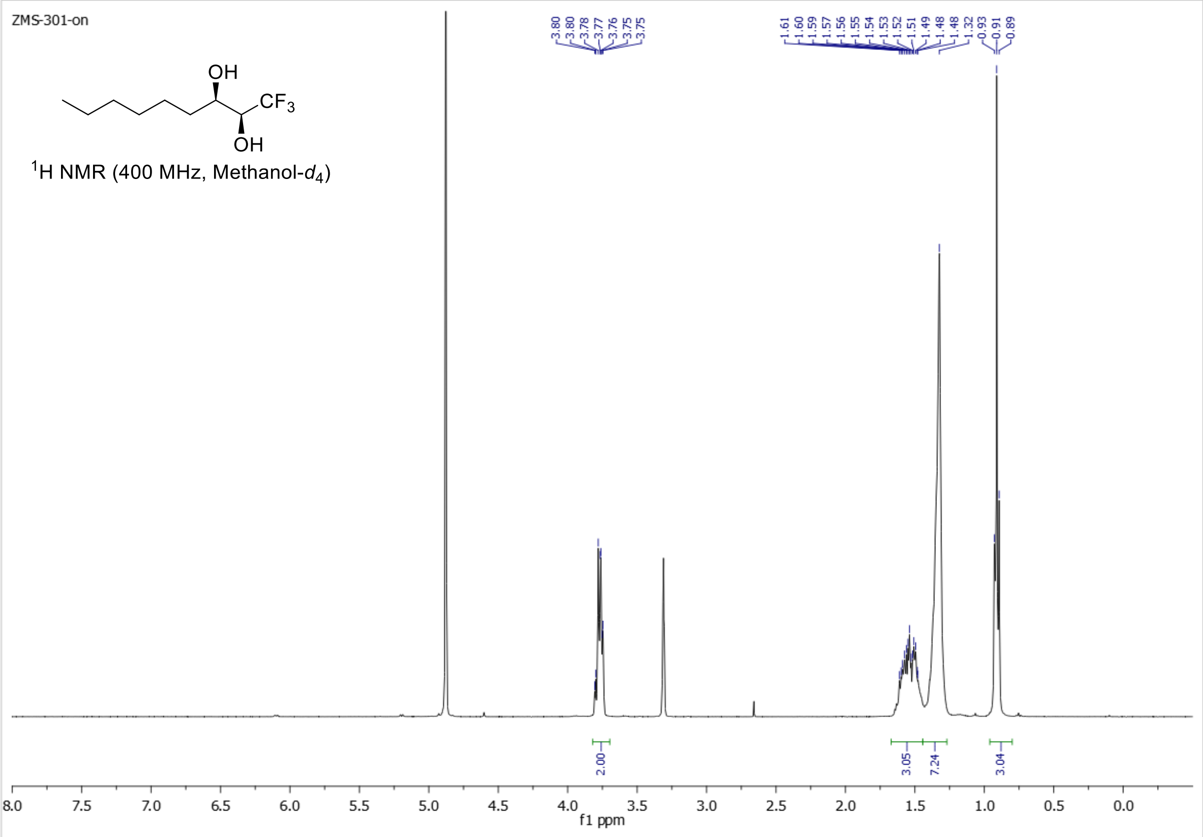

Compound ***syn*-3p**, ^{19}F NMR:

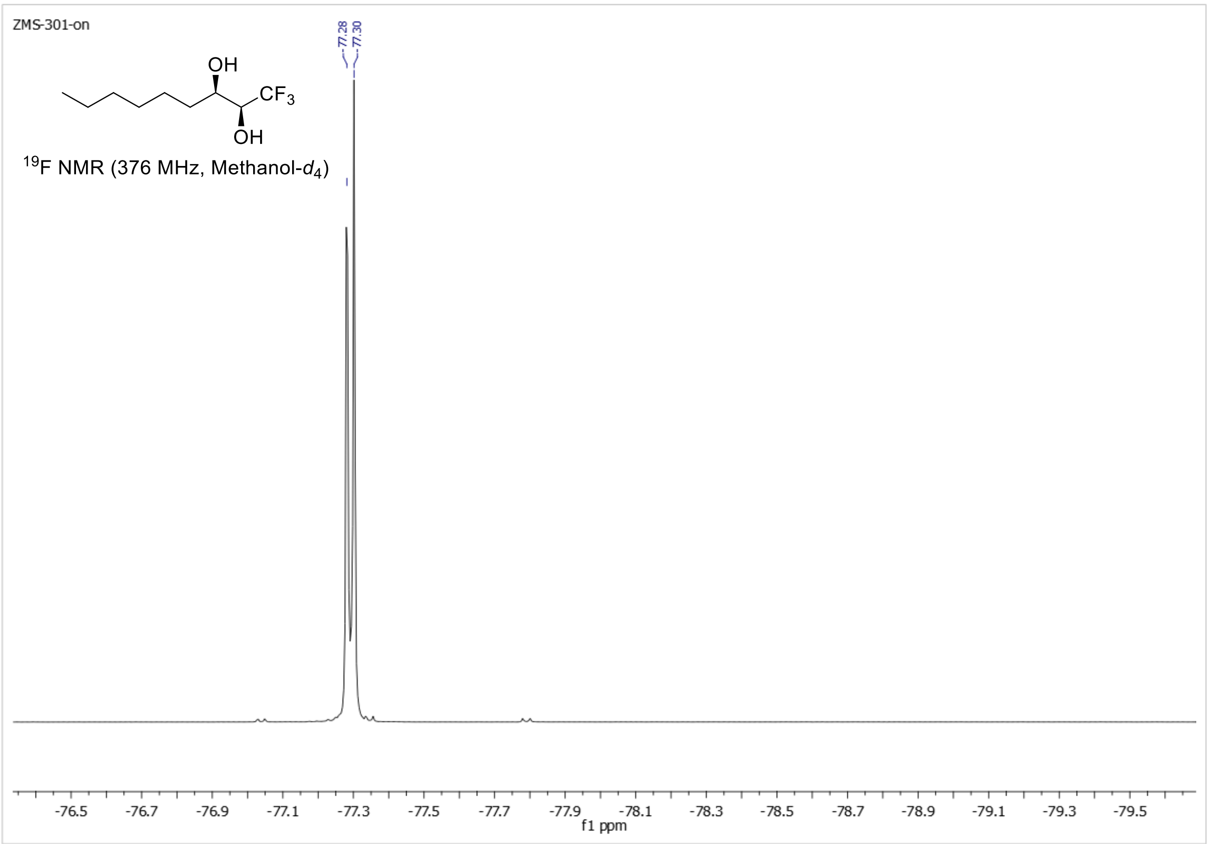

Compound ***syn*-3p**, ^{13}C NMR:

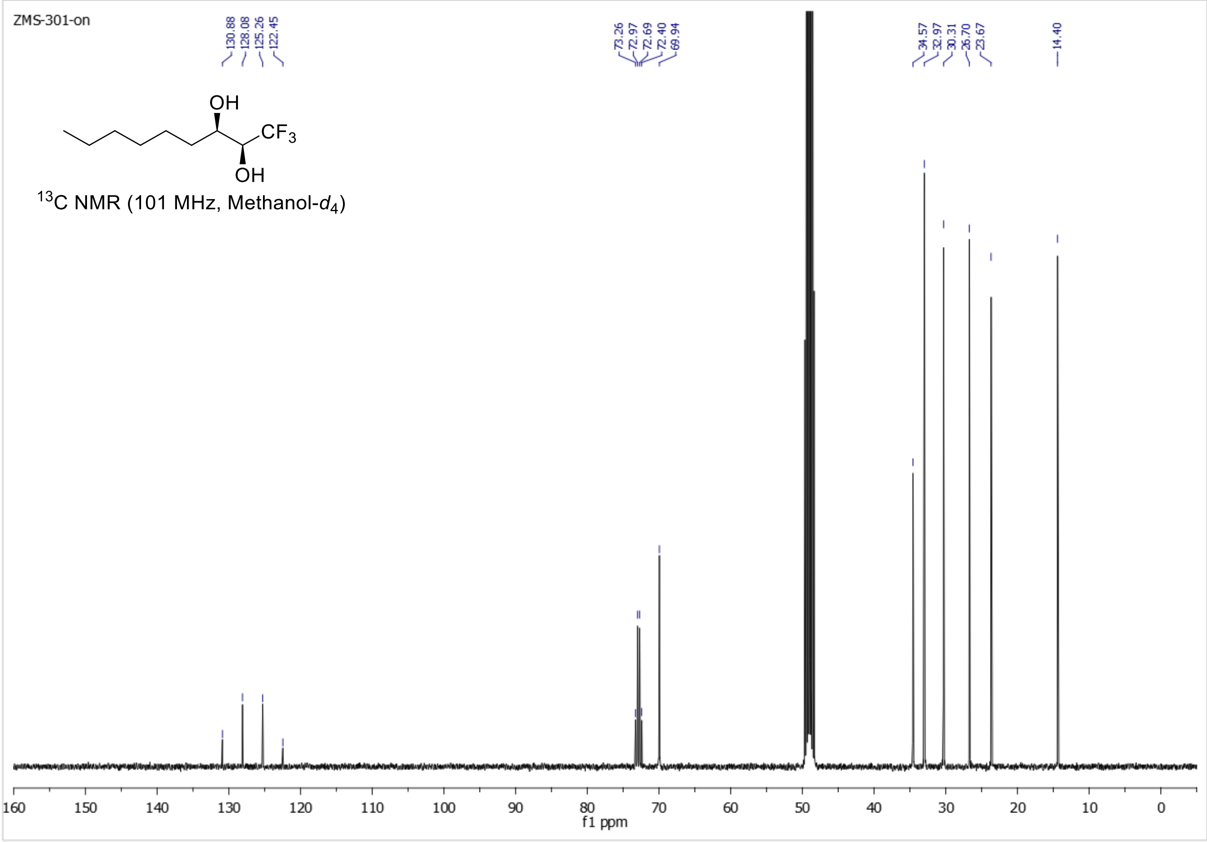

Compound **(±)-*anti*-3p**,^{1}H NMR:

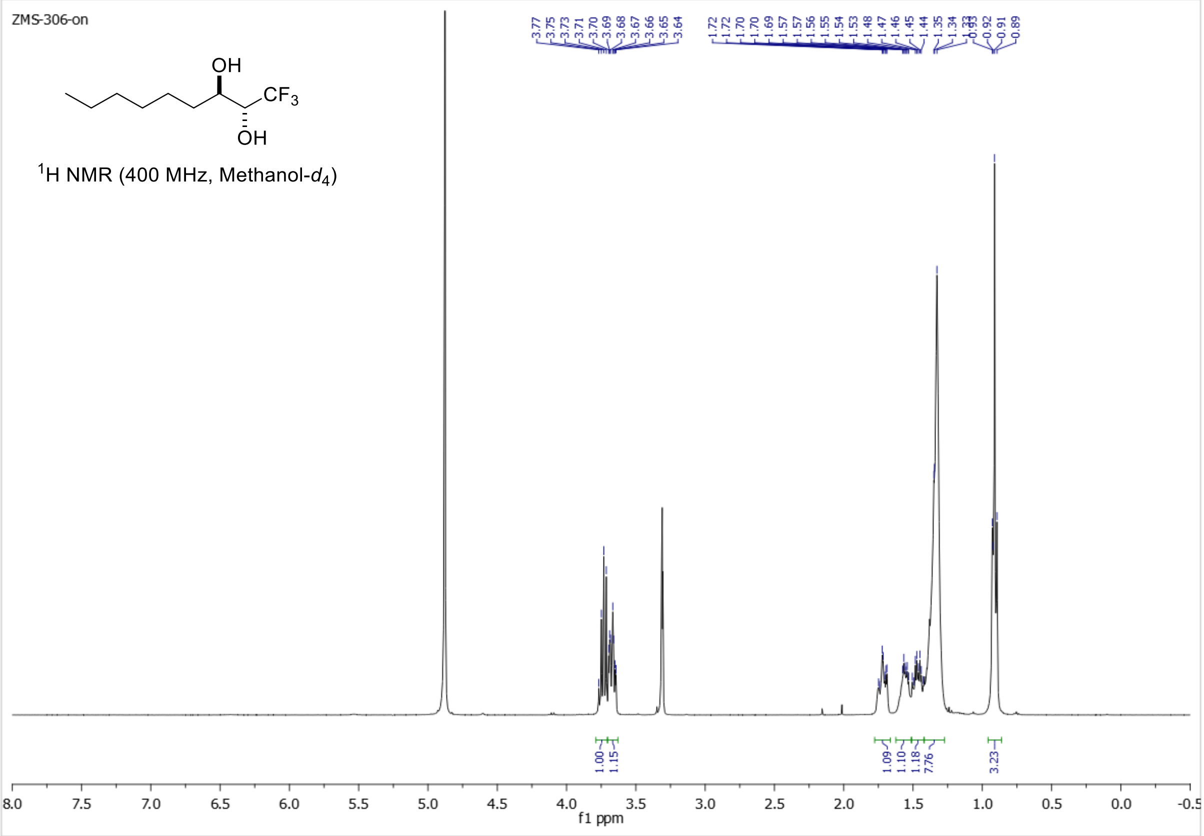

Compound **(±)-*anti*-3p**, ^{19}F NMR:

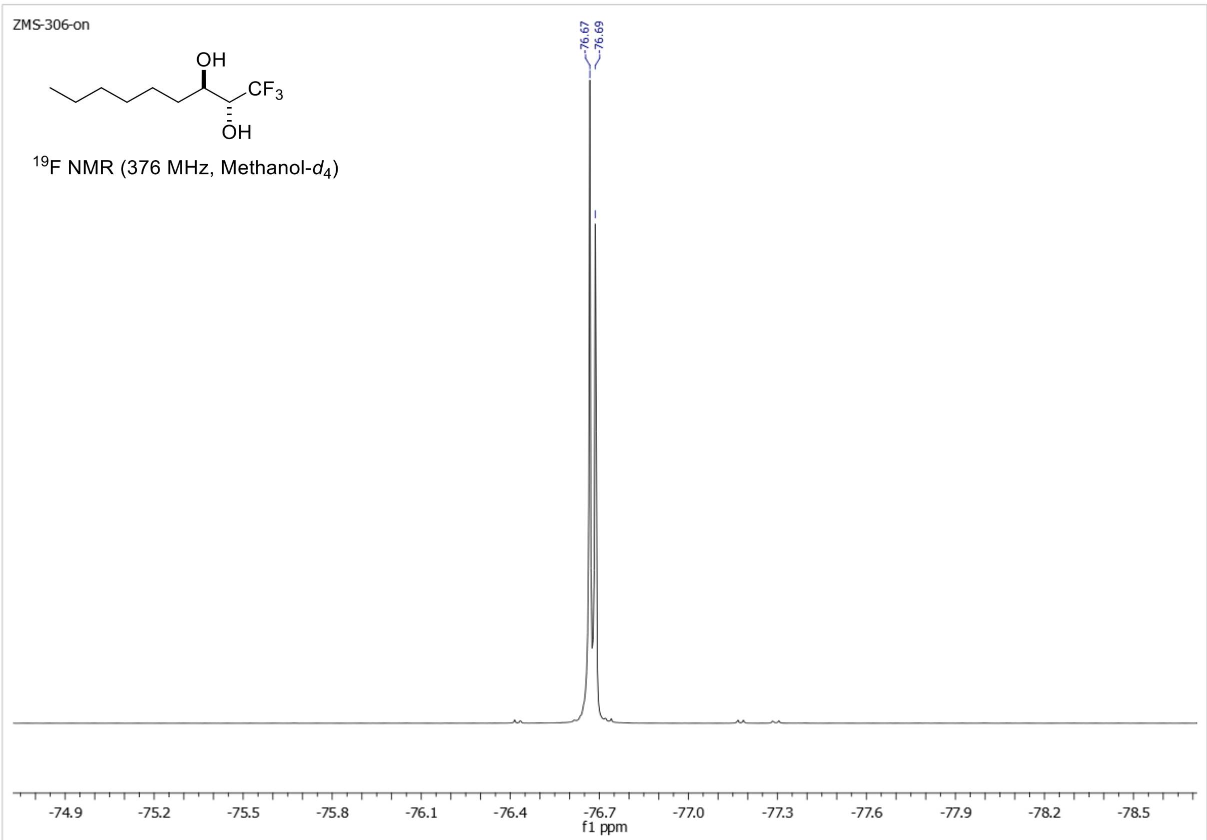

Compound **(±)-*anti*-3p**, ^{13}C NMR:

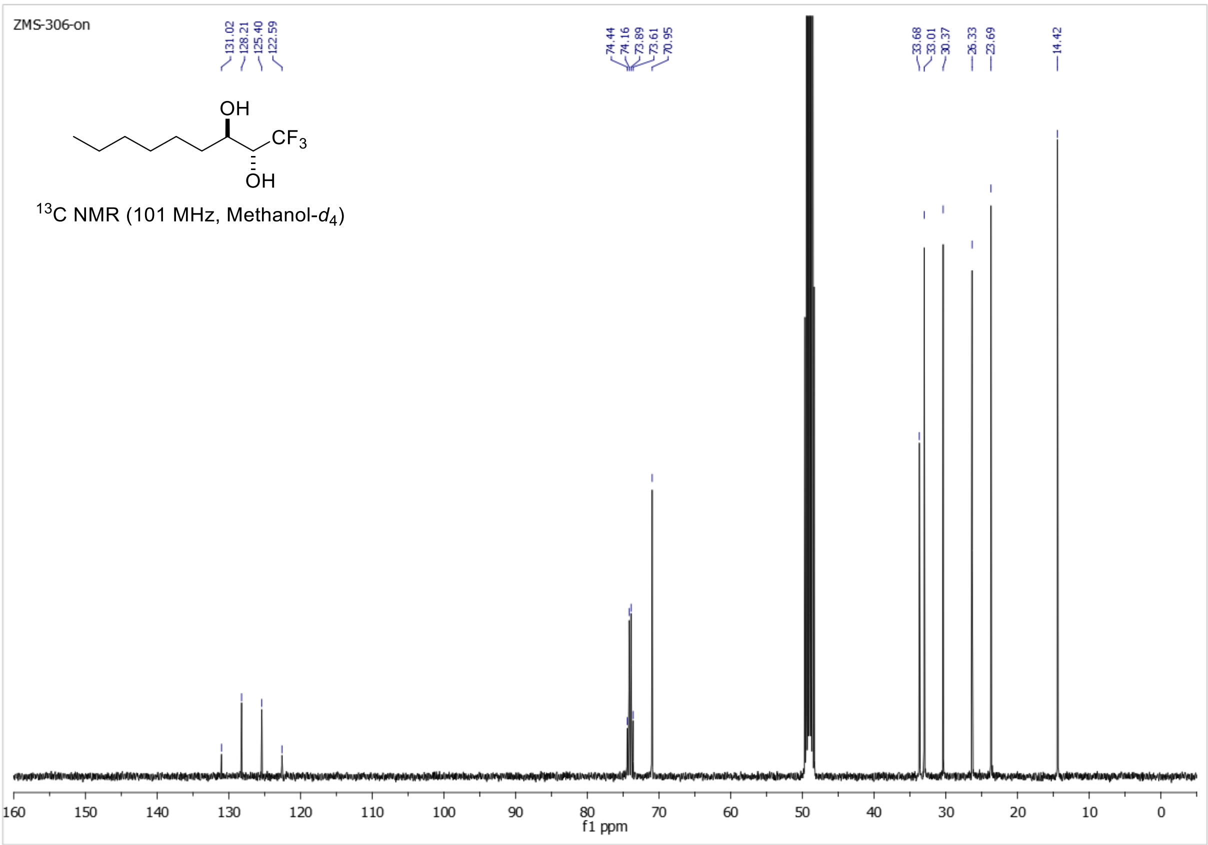

Compound ***syn*-3q**, ^{1}H NMR:

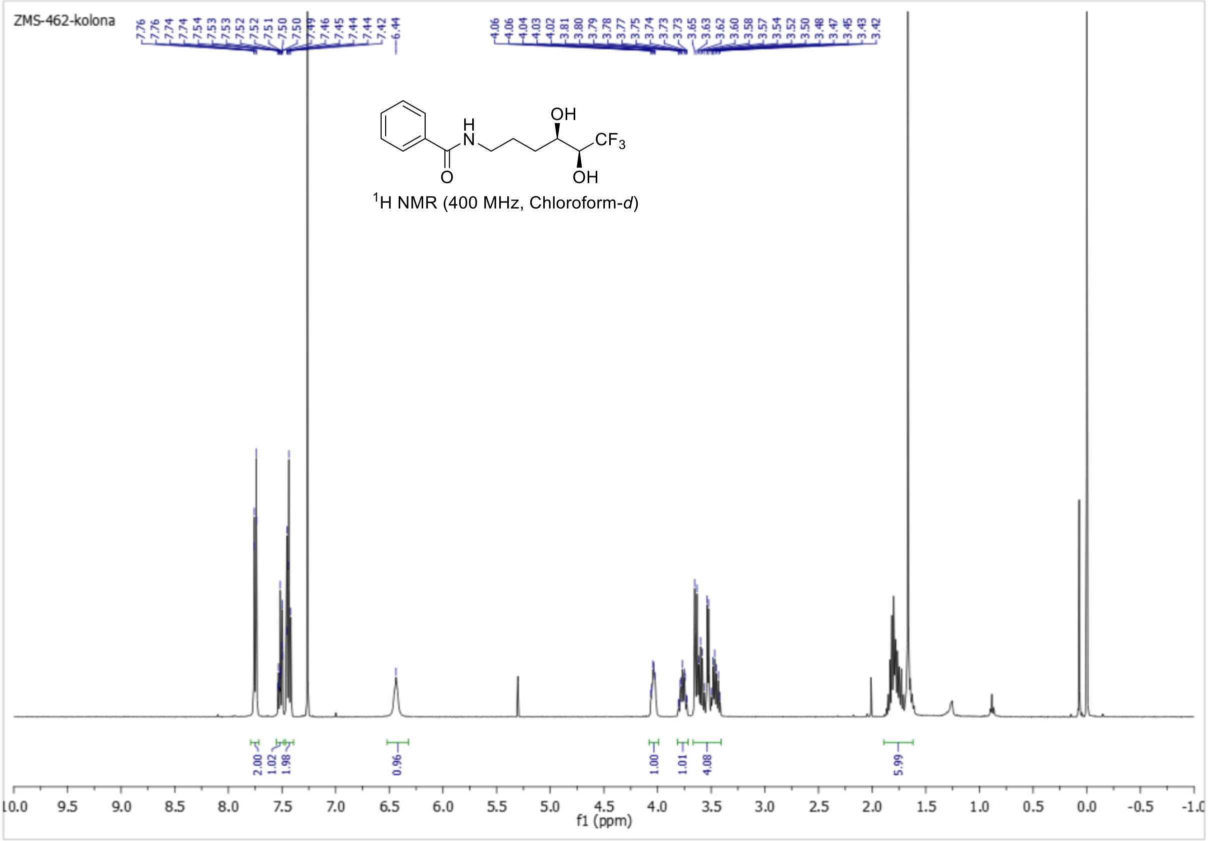

Compound ***syn*-3q**, ^{19}F NMR:

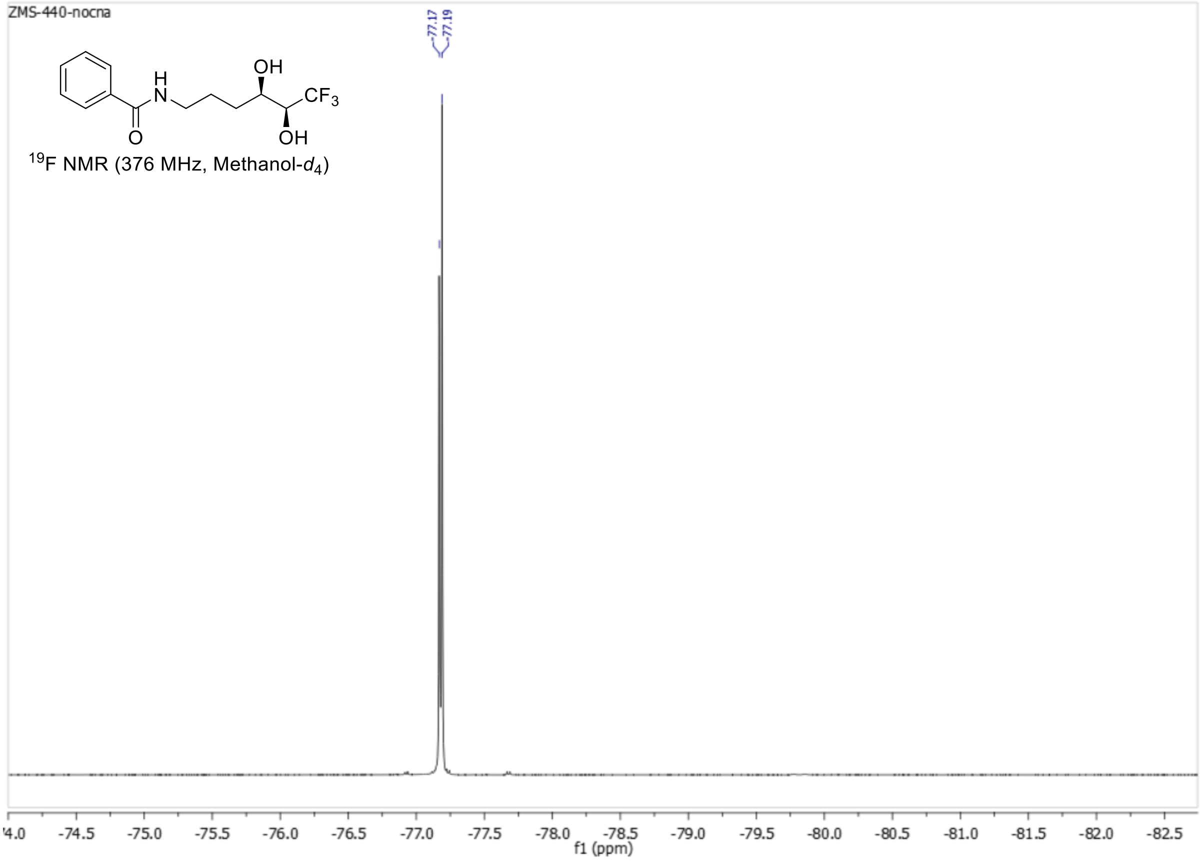

Compound ***syn*-3q**, ^{13}C NMR:

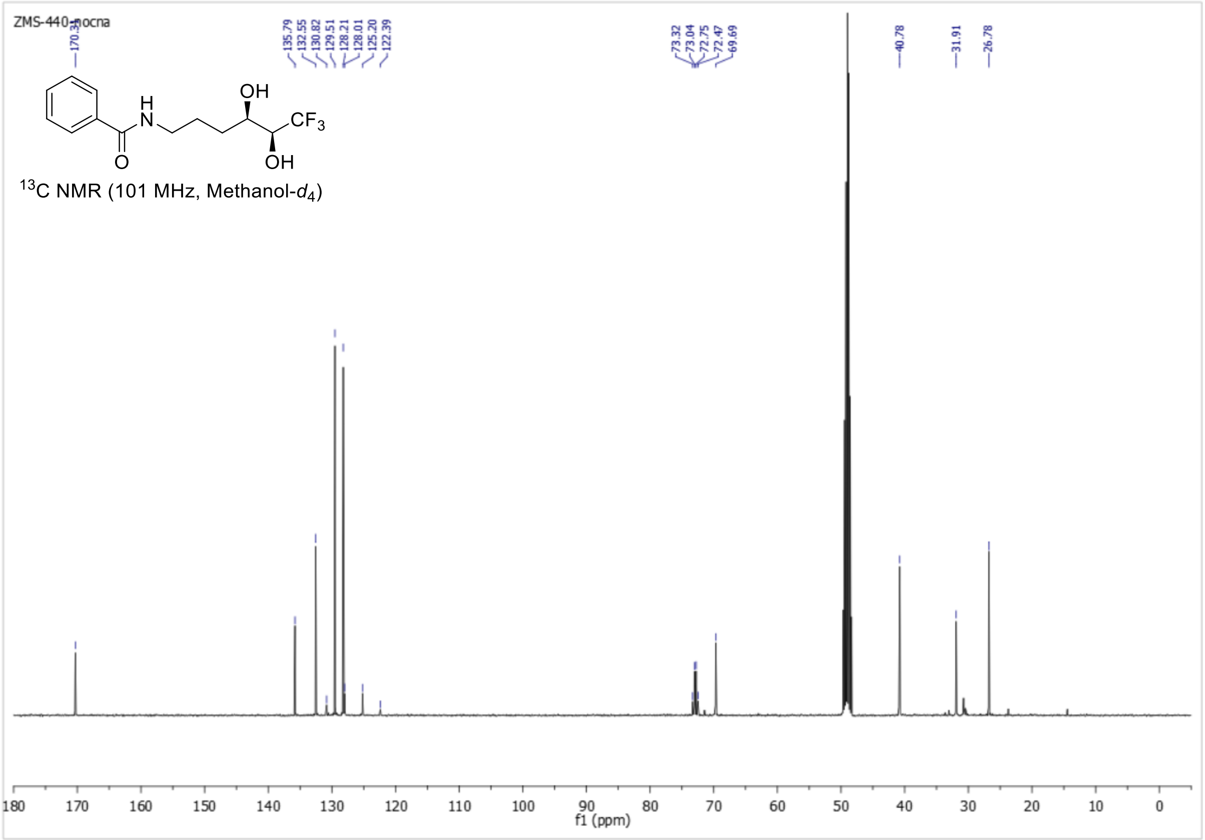

Compound **(±)-*anti*-3q**, ^{1}H NMR:

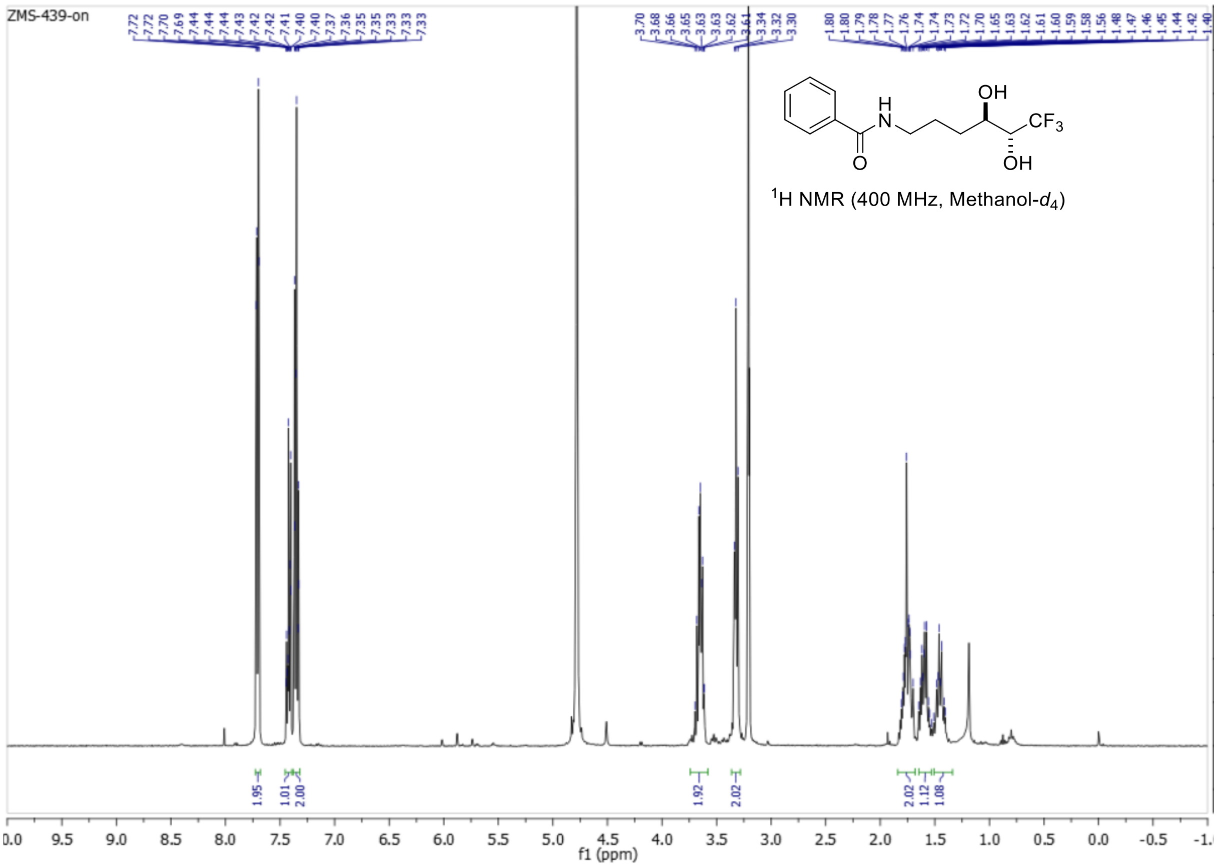

Compound **(±)-*anti*-3q**, ^{19}F NMR:

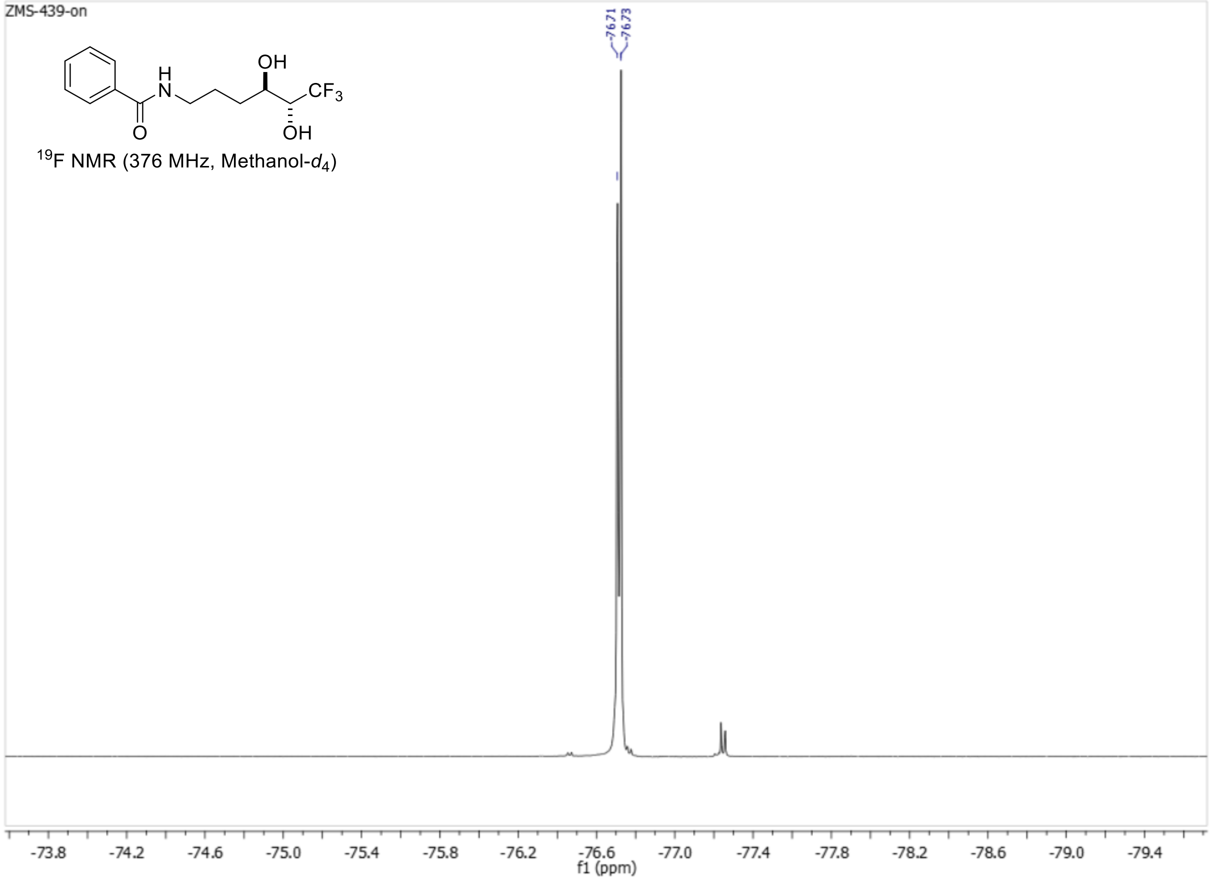

Compound **(±)-*anti*-3q**, ^{13}C NMR:

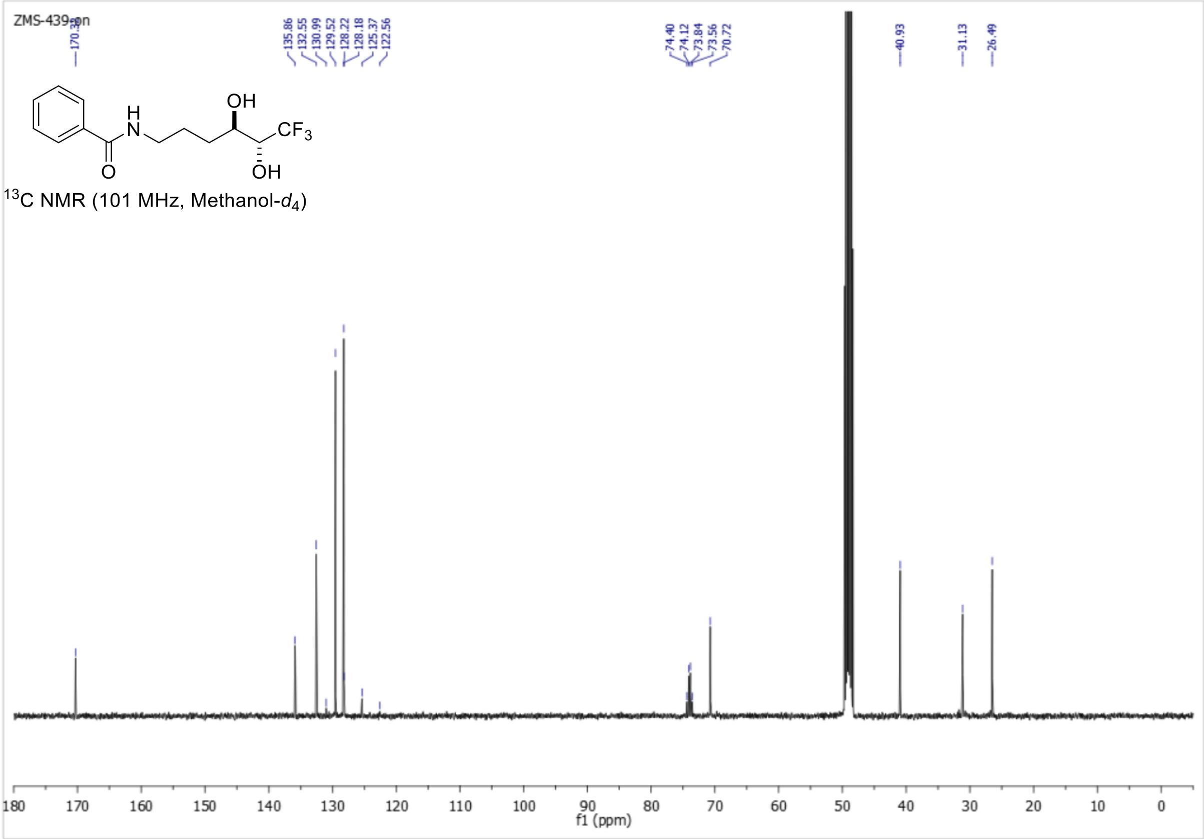

Compound *syn*-**3r**, ^{1}H NMR

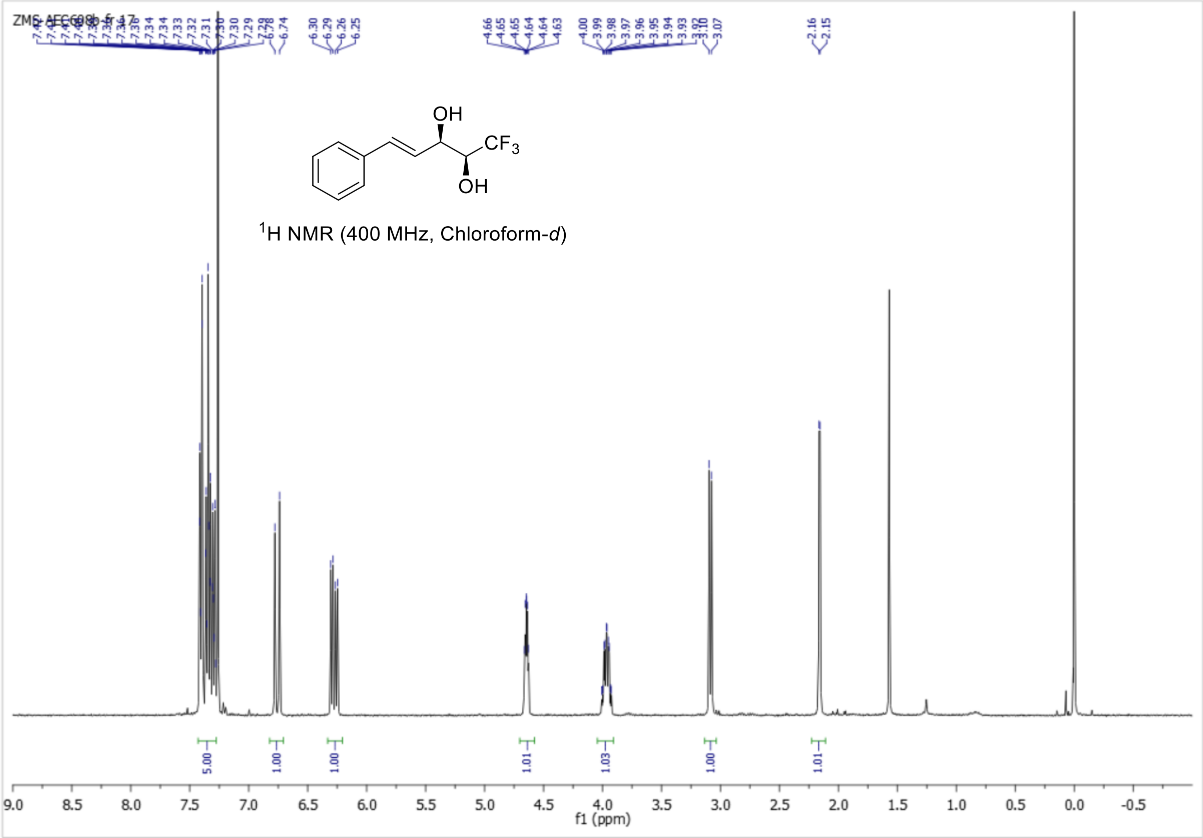

Compound *syn*-**3r**, ^{19}F NMR:

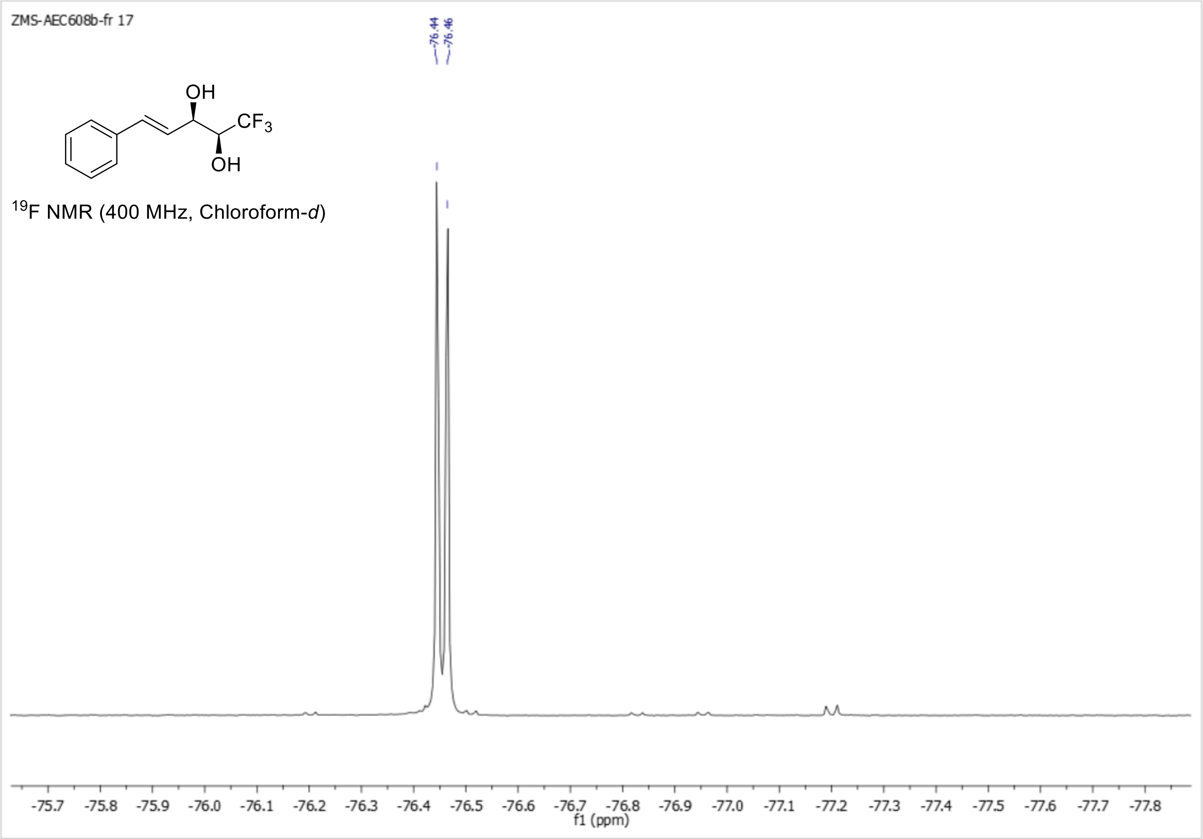

Compound *syn*-**3r**, ^{13}C NMR:

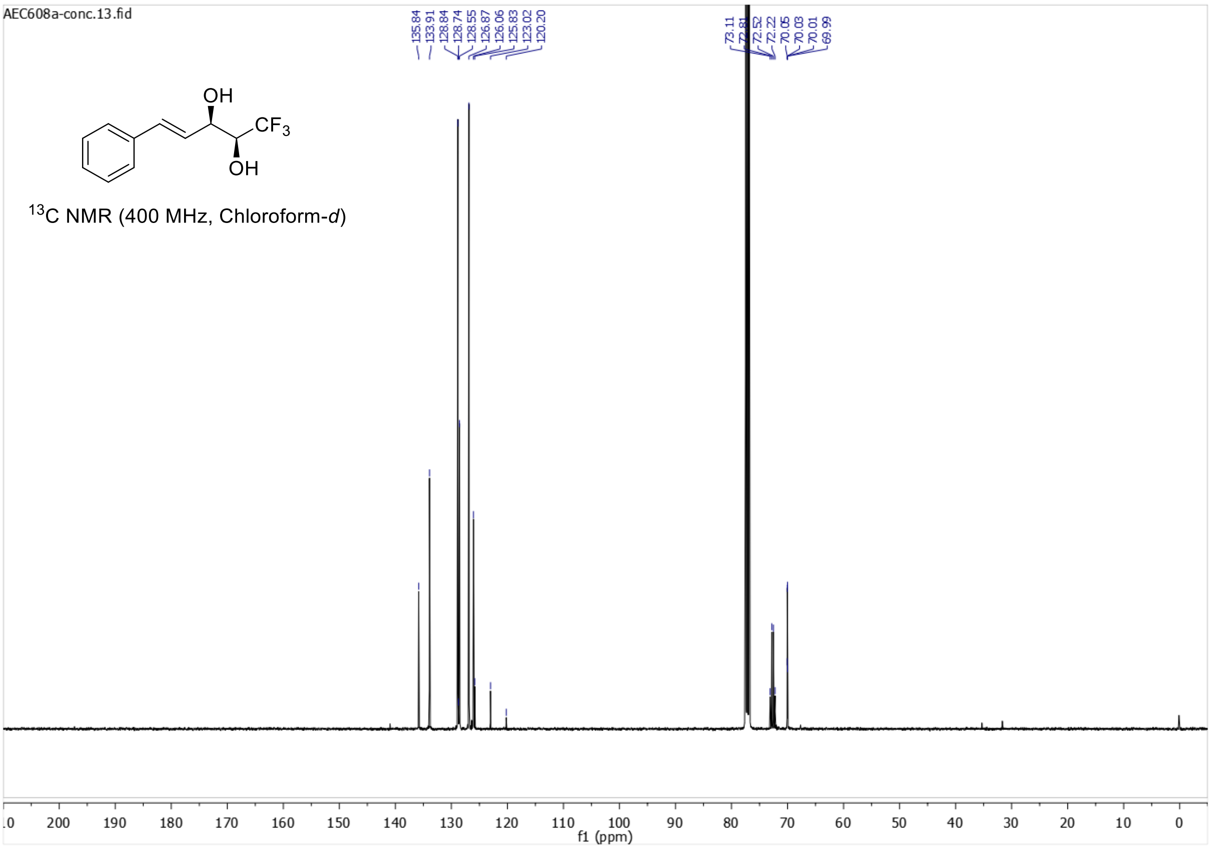

(4*R*,5*S*)-2,2-dimethyl-4-(4-nitrophenyl)-5-(trifluoromethyl)-1,3-dioxolane, ^{1}H NMR:

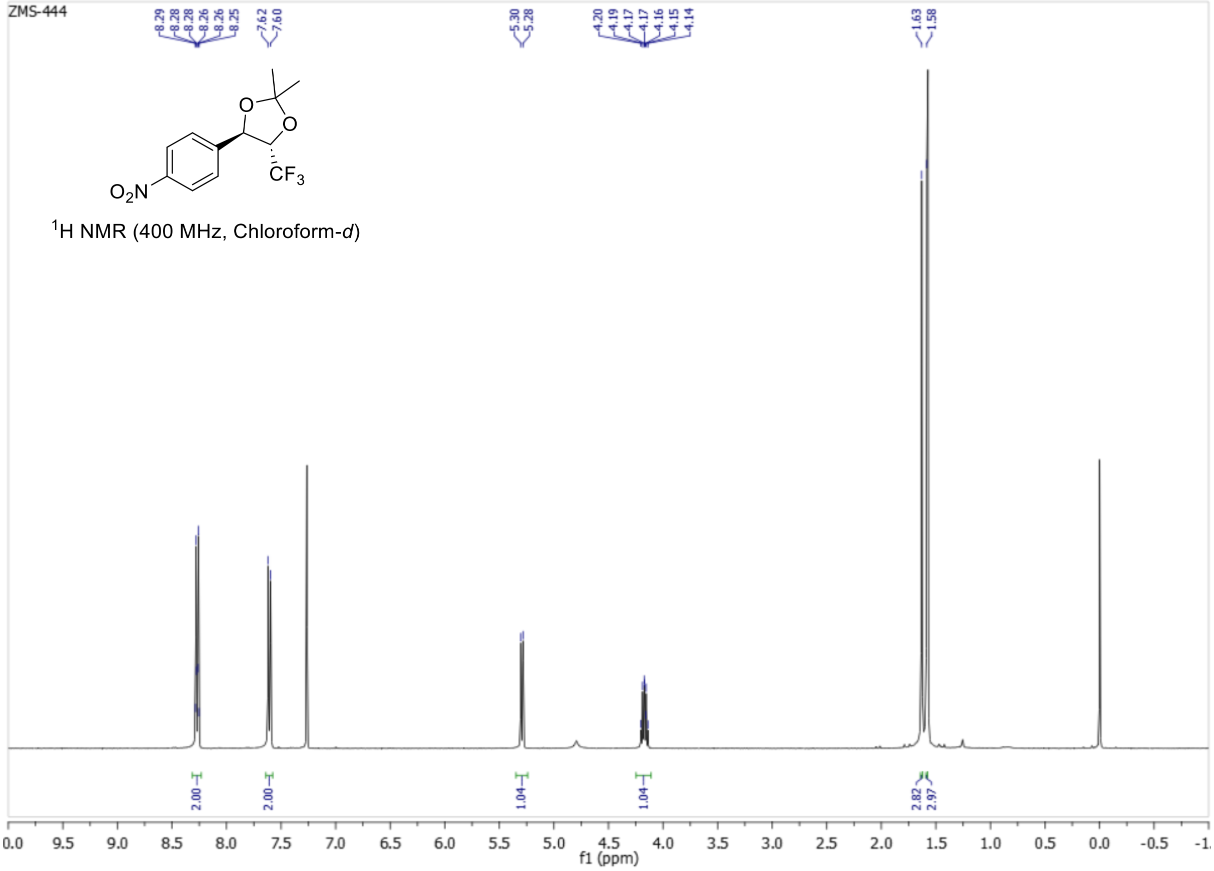

(4*R*,5*S*)-2,2-dimethyl-4-(4-nitrophenyl)-5-(trifluoromethyl)-1,3-dioxolane, ^{19}F NMR:

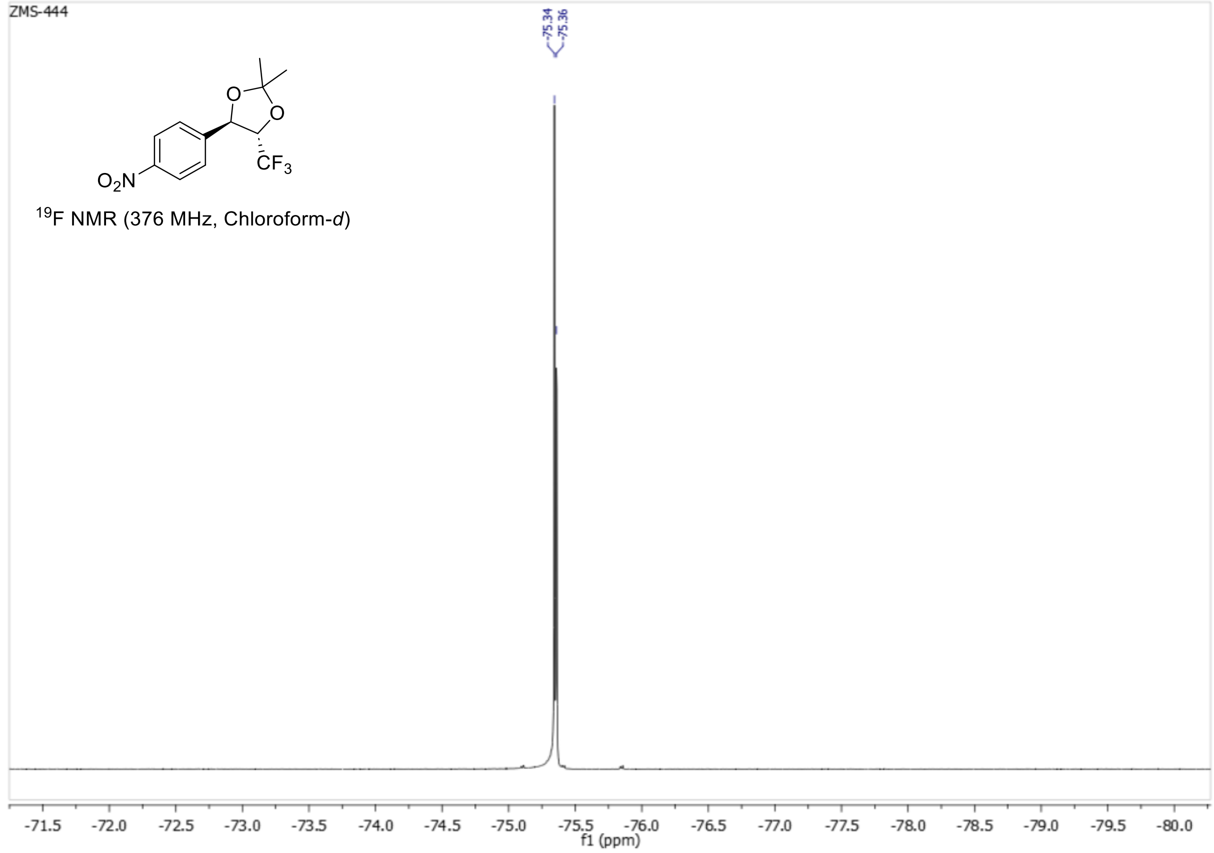

Compound **4**, ^{1}H NMR:

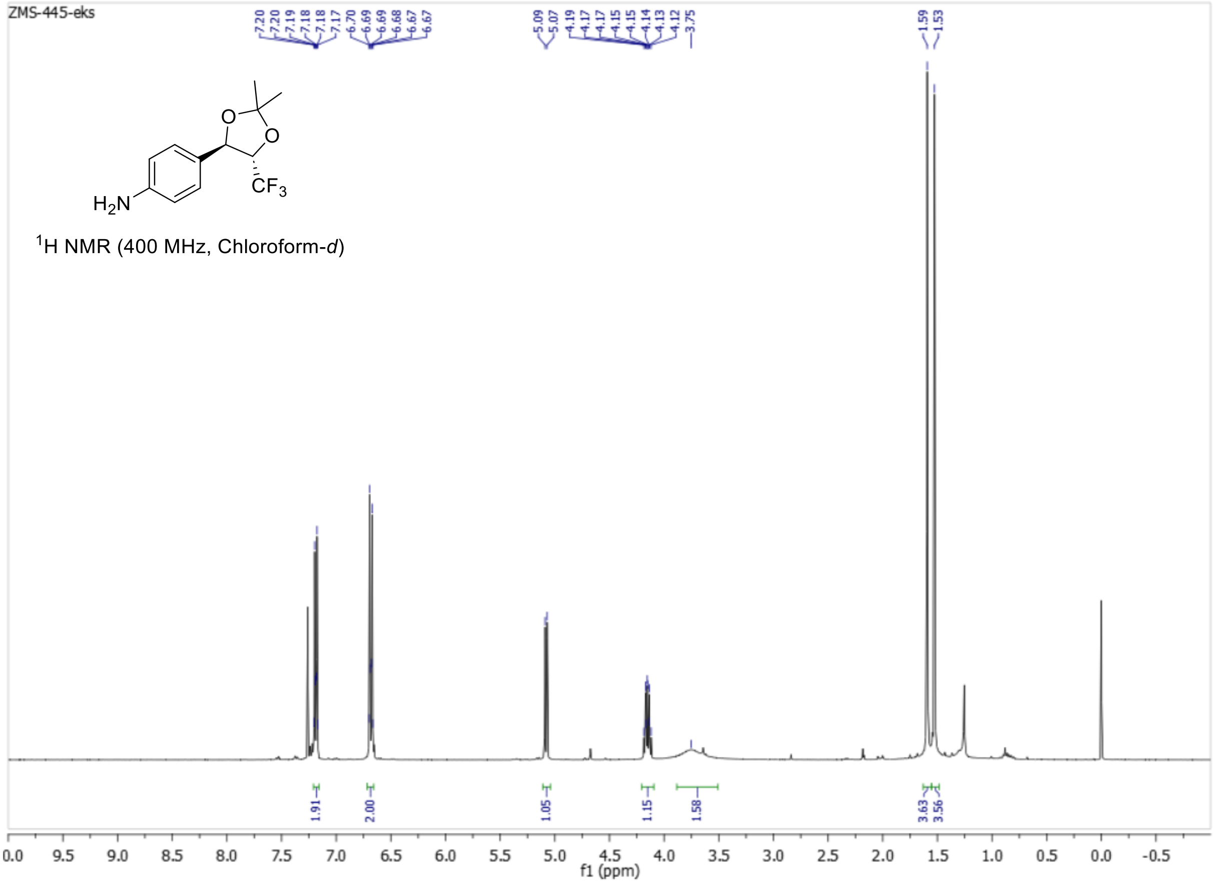

Compound **4**, ^{19}F NMR:

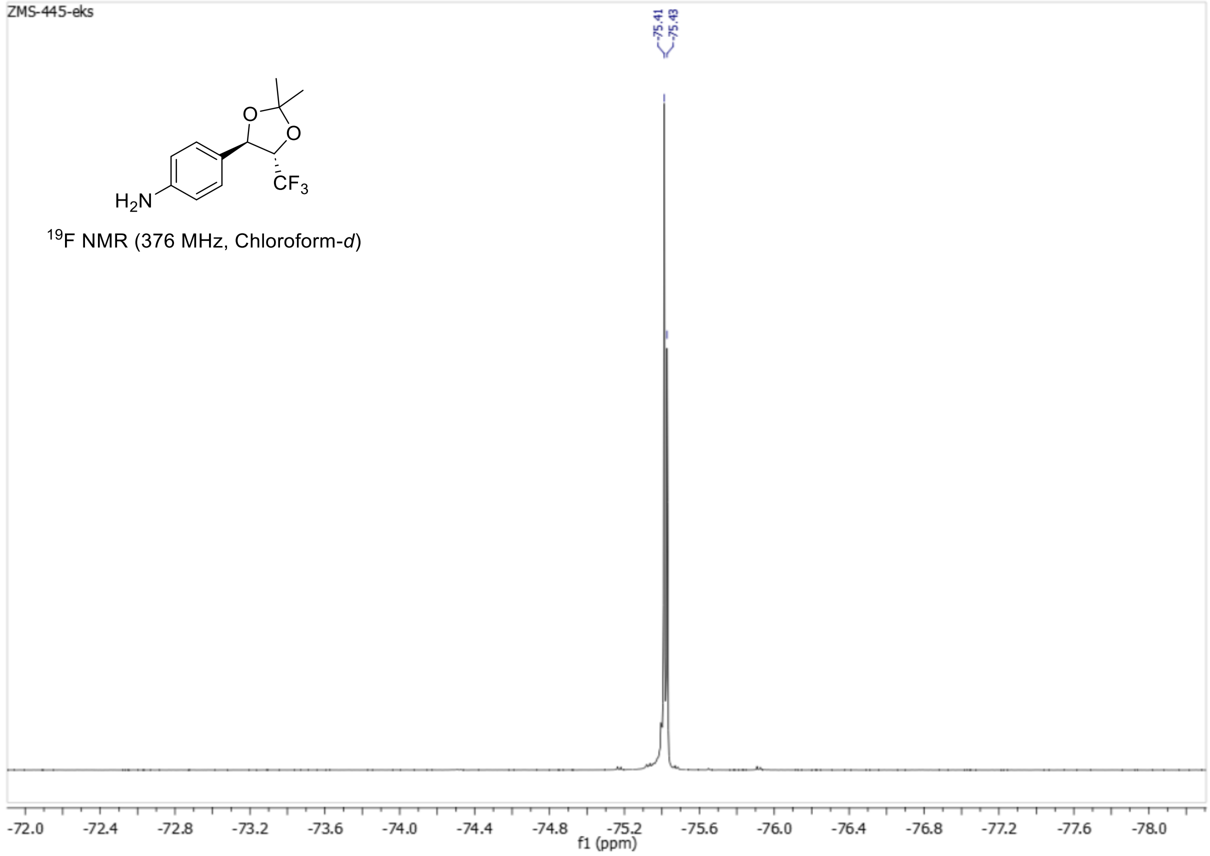

Compound **4**, ^{13}C NMR:

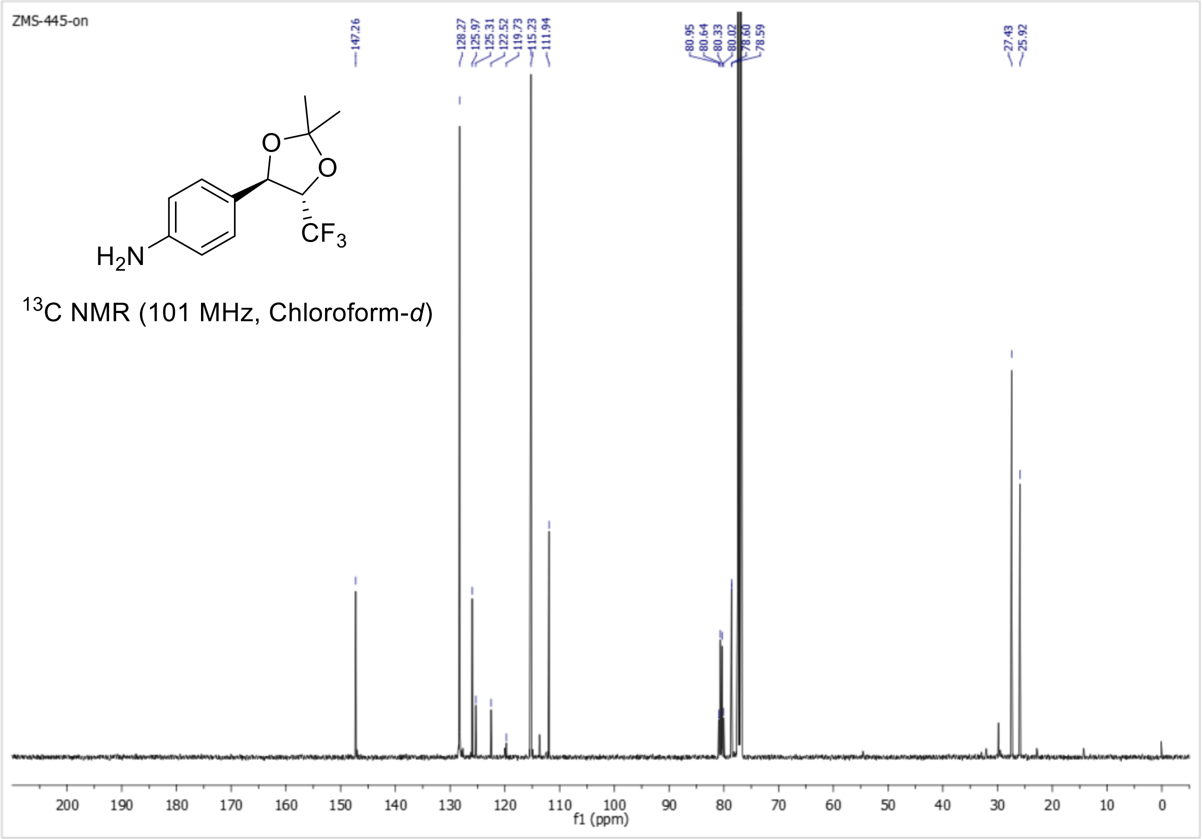

5-chloro-1*H*-indole-2-carbonyl chloride, ^{1}H NMR:

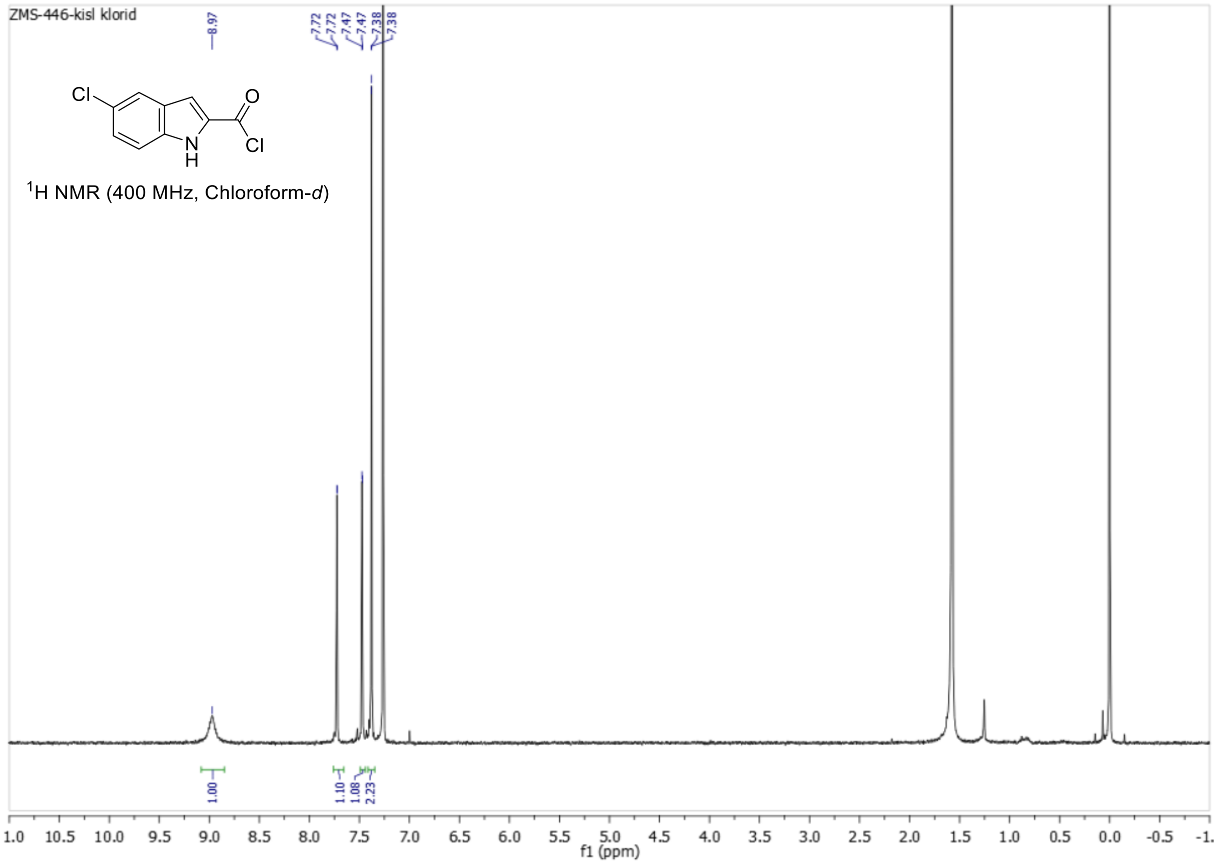

Compound **5-acetonid**, ^{1}H NMR:

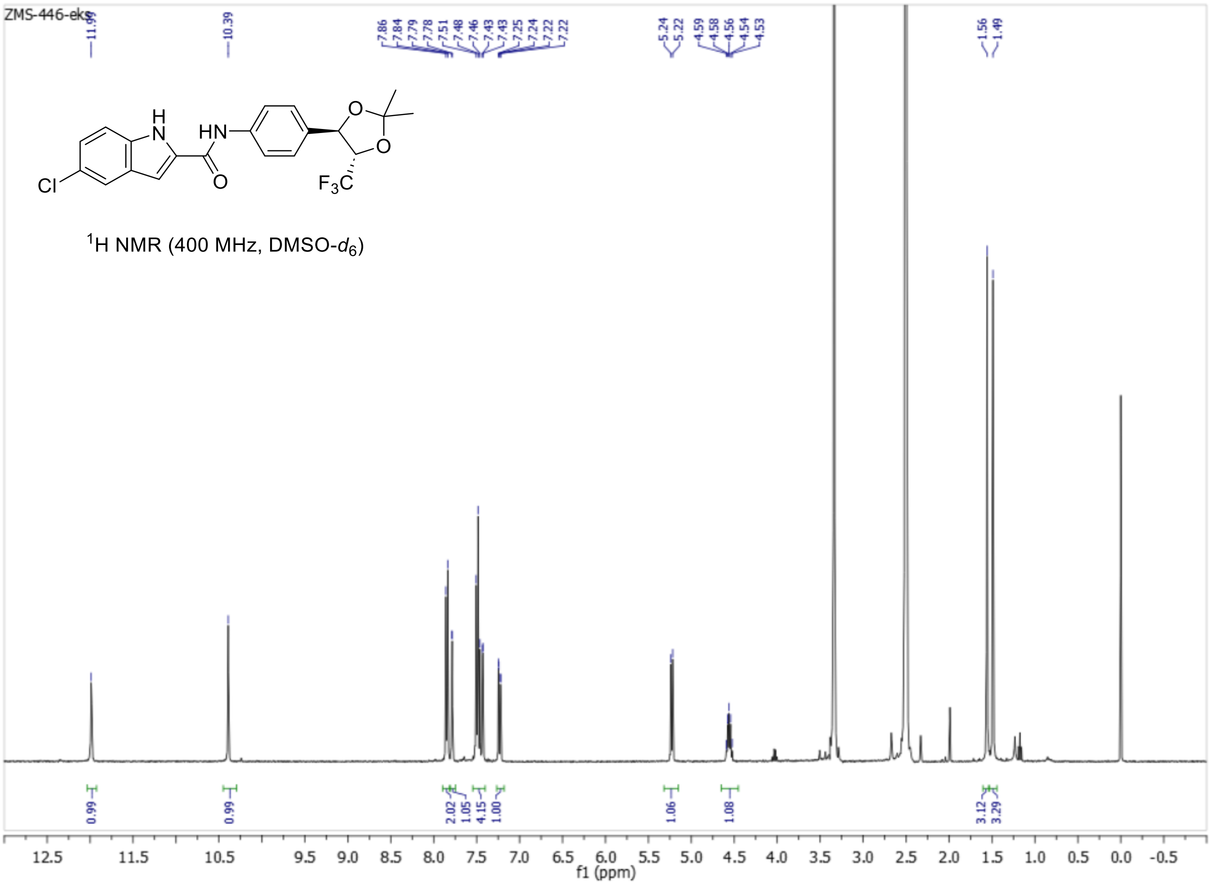

Compound **5-acetonid**, ^{19}F NMR:

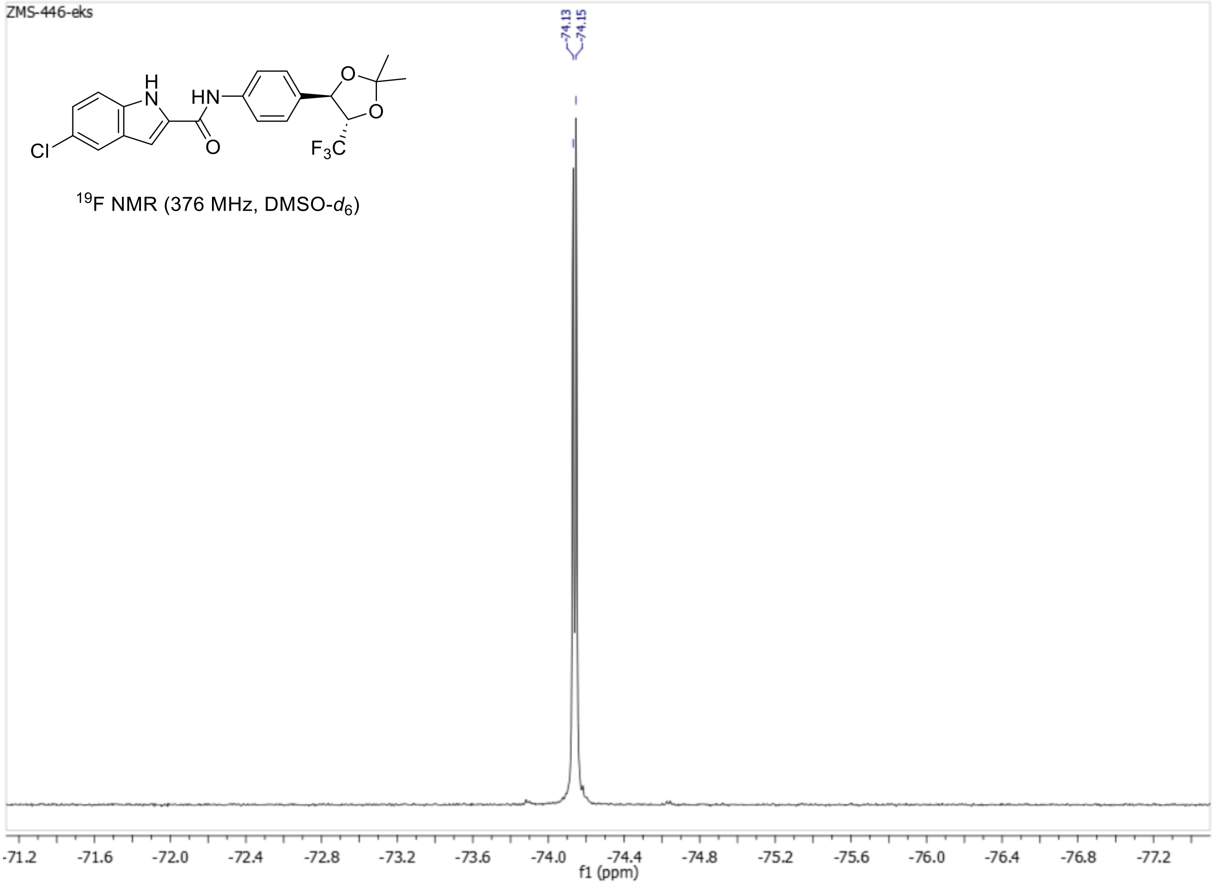

Compound **5-acetonid**, ^{13}C NMR:

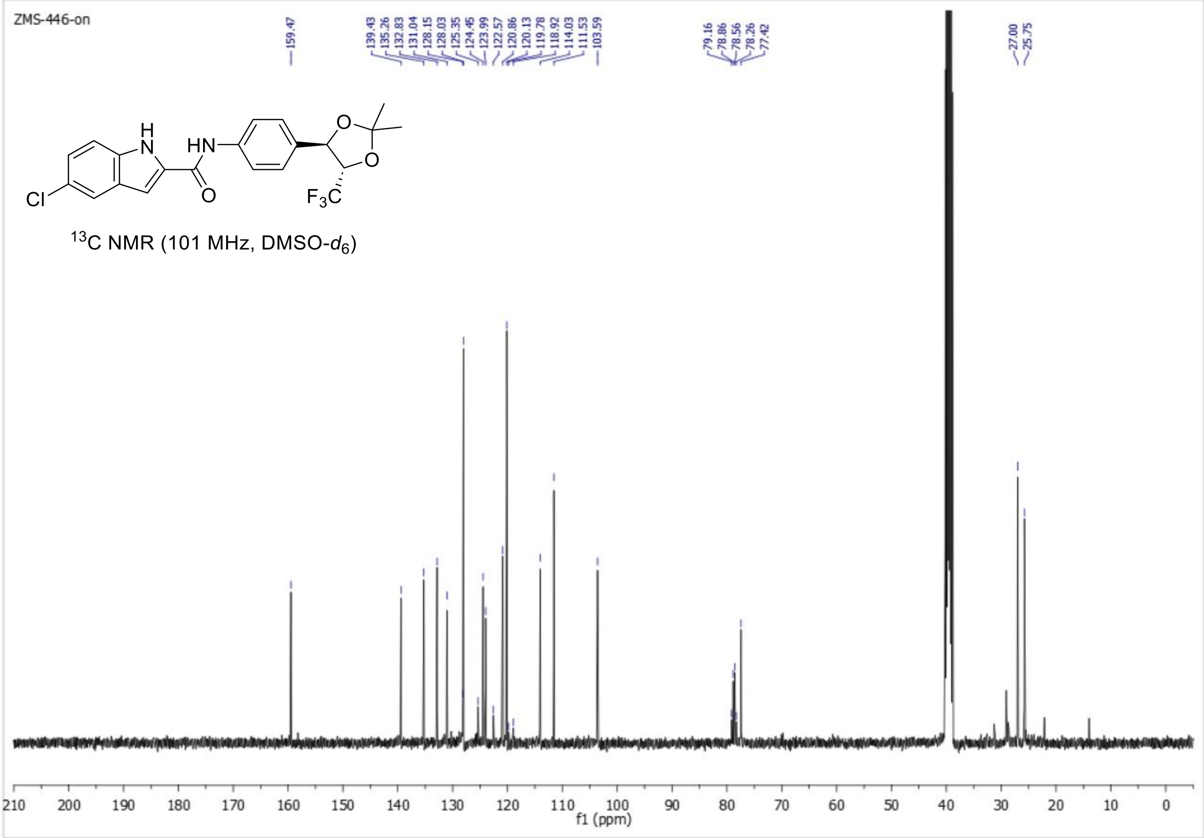

Compound **5**, ^{1}H NMR:

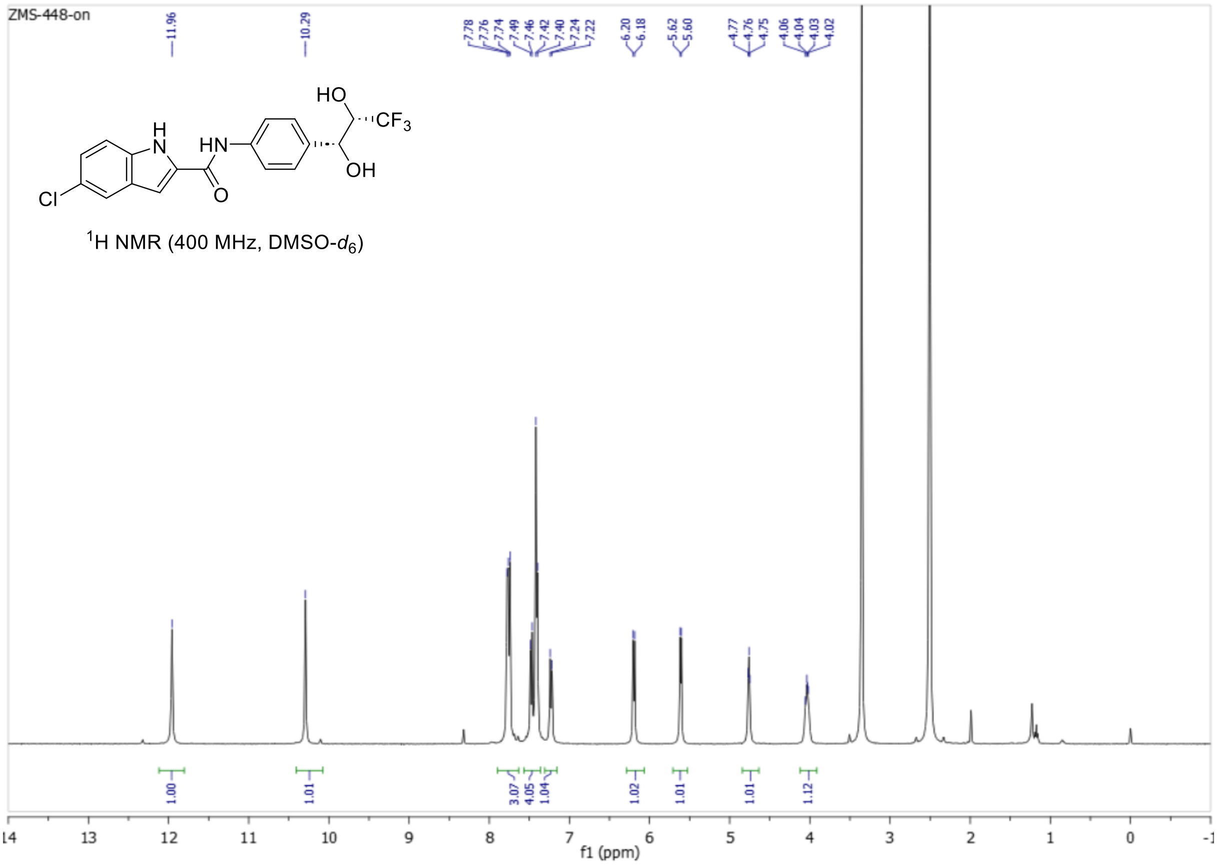

Compound **5**, ^{19}F NMR:

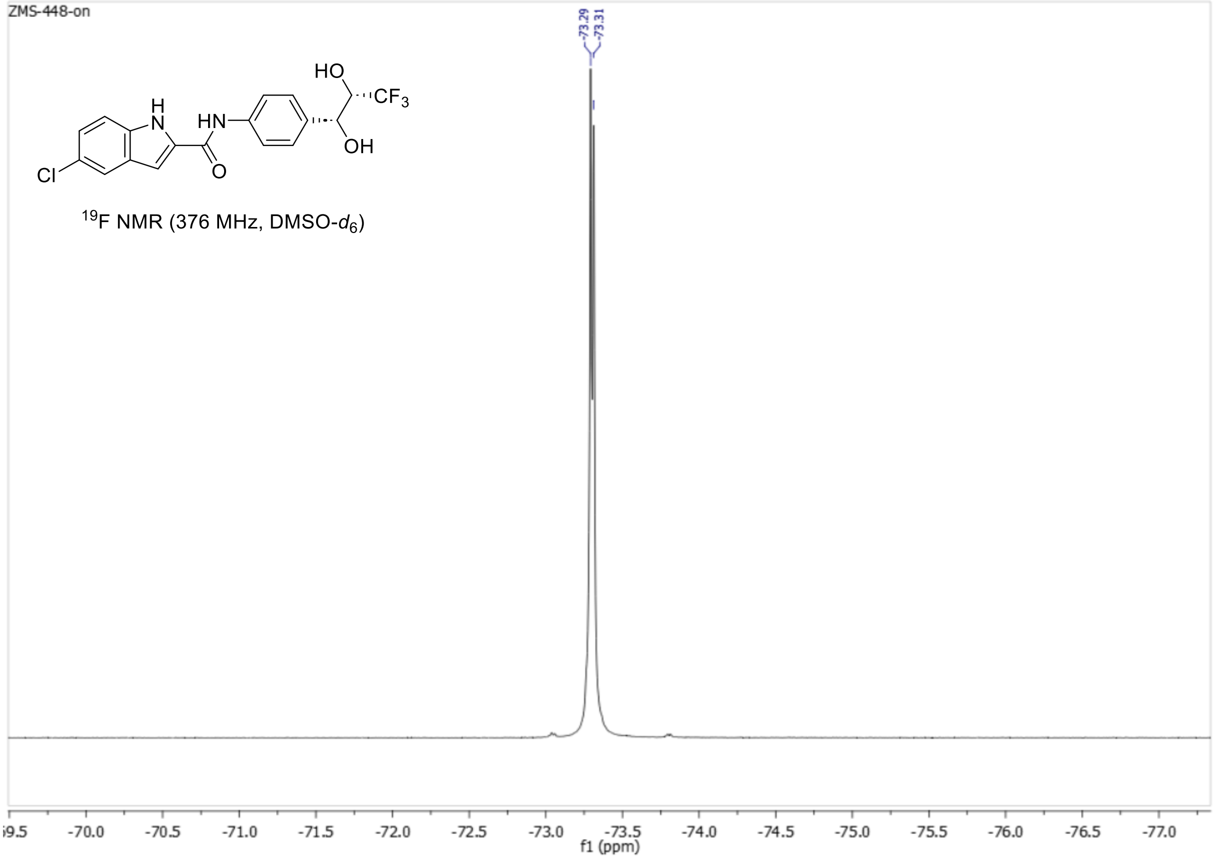

Compound **5**, ^{13}C NMR:

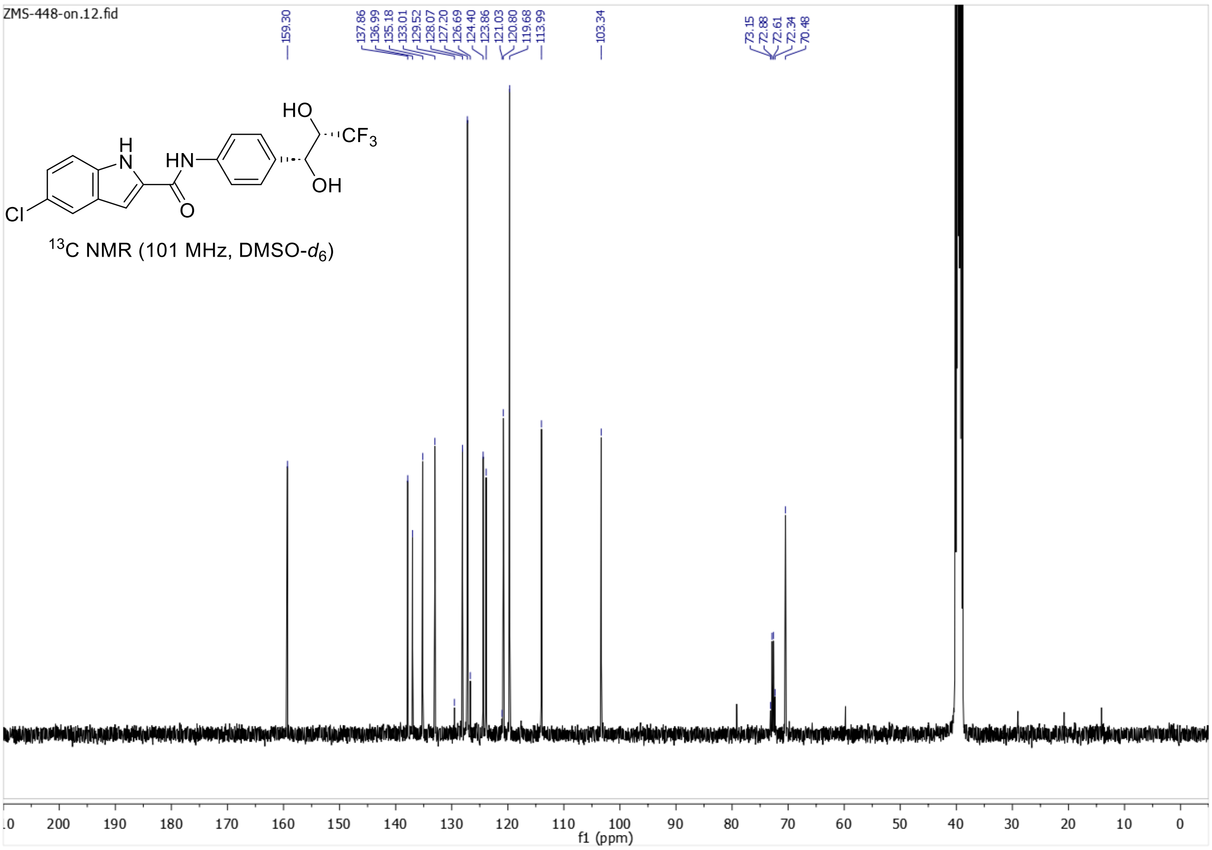

Compound (1*R*,2*S*)-**6′**, ^{1}H NMR:

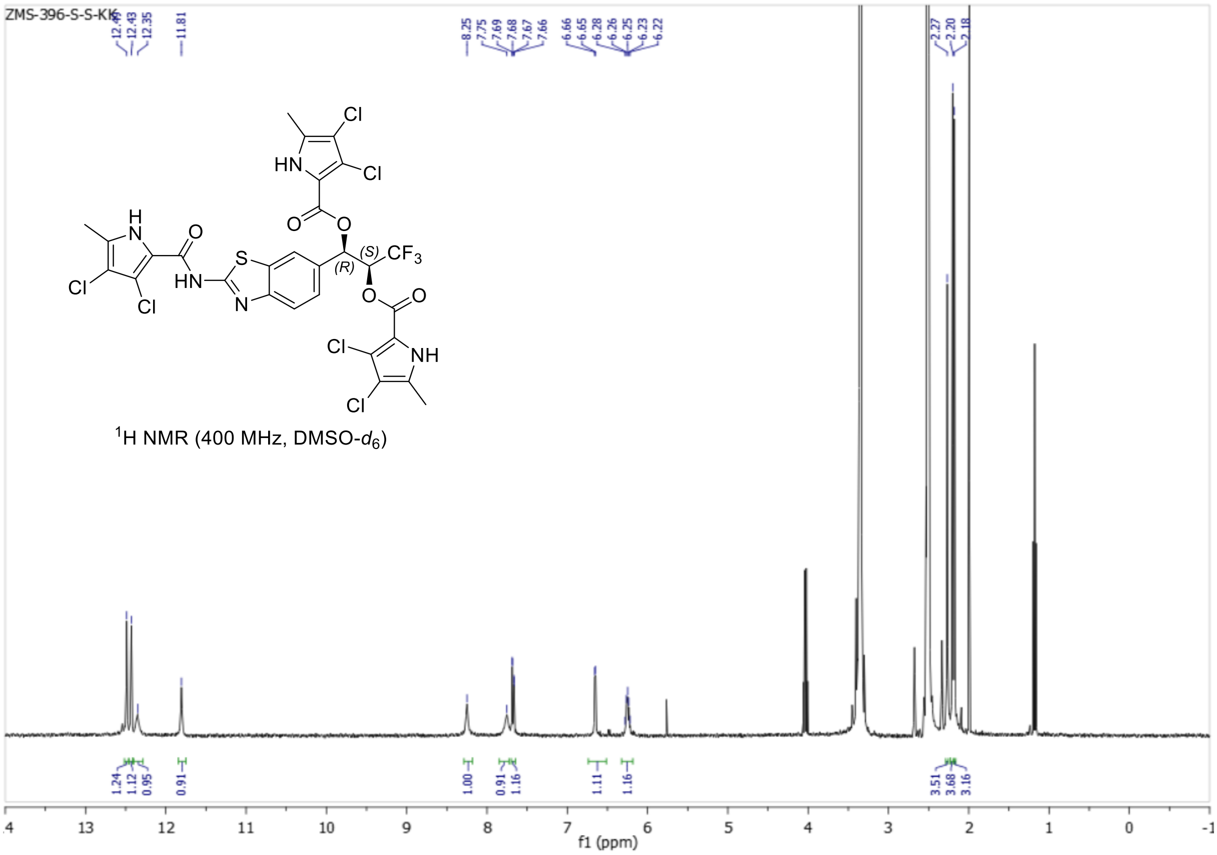

Compound (1*R*,2*S*)-**6′,** ^{19}F NMR:

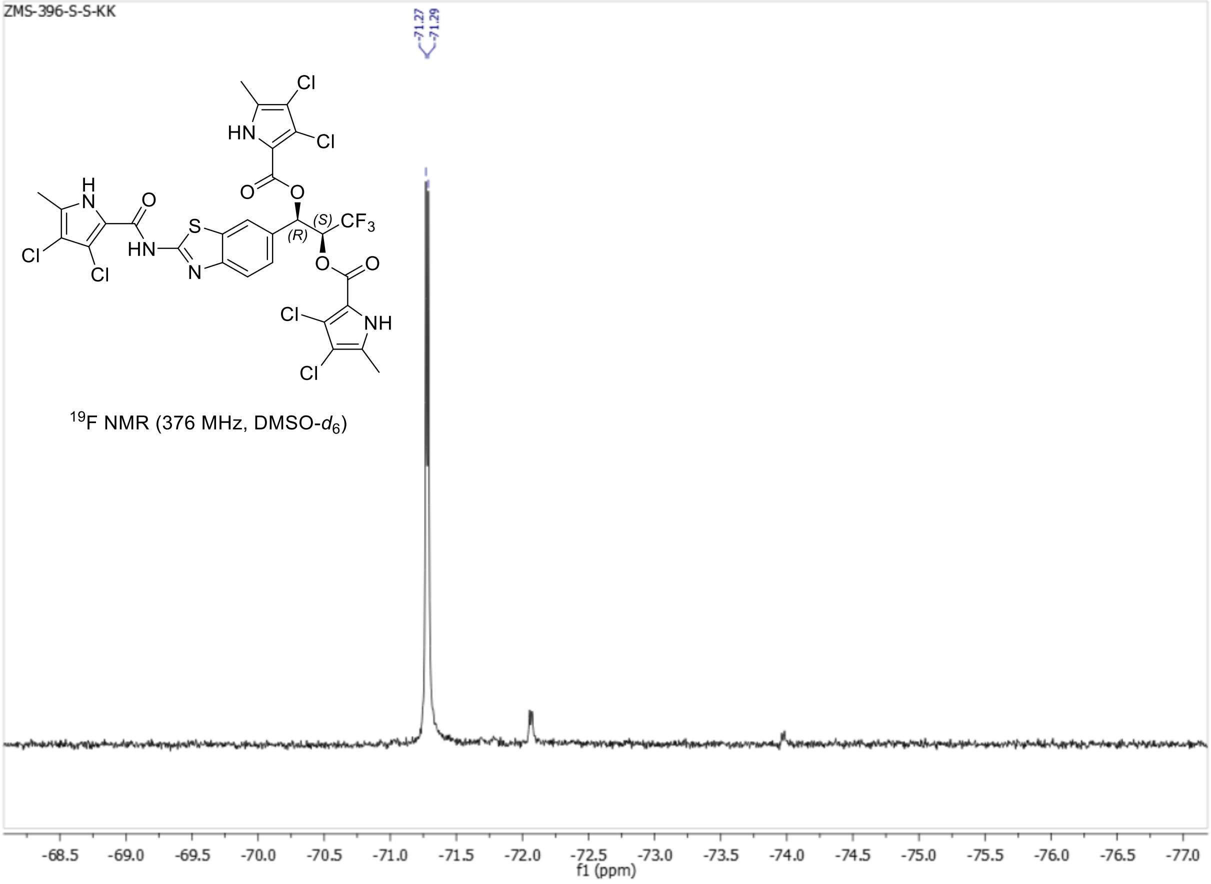

Compound (1*S*,2*R*)-**6'**, ^{1}H NMR:

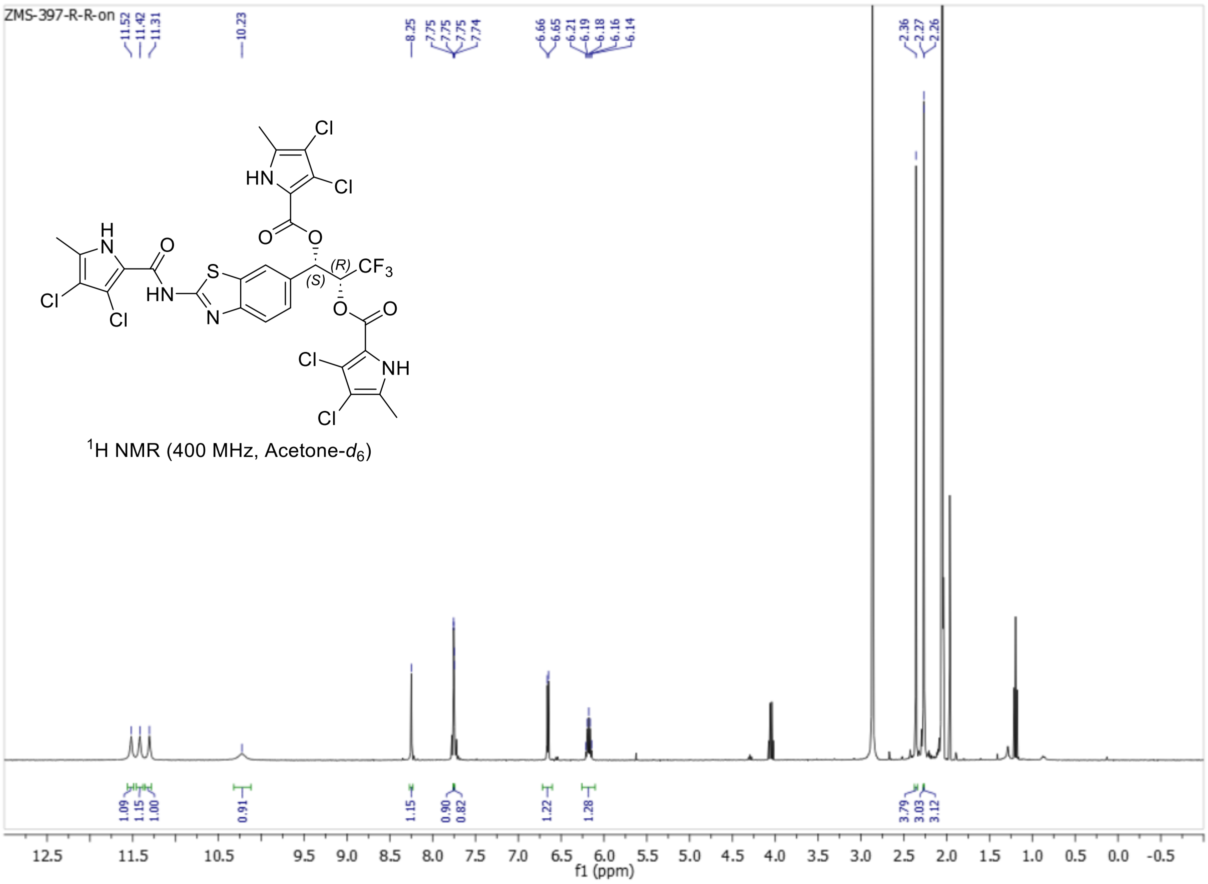

Compound (1*S*,2*R*)-**6'**, ^{19}F NMR:

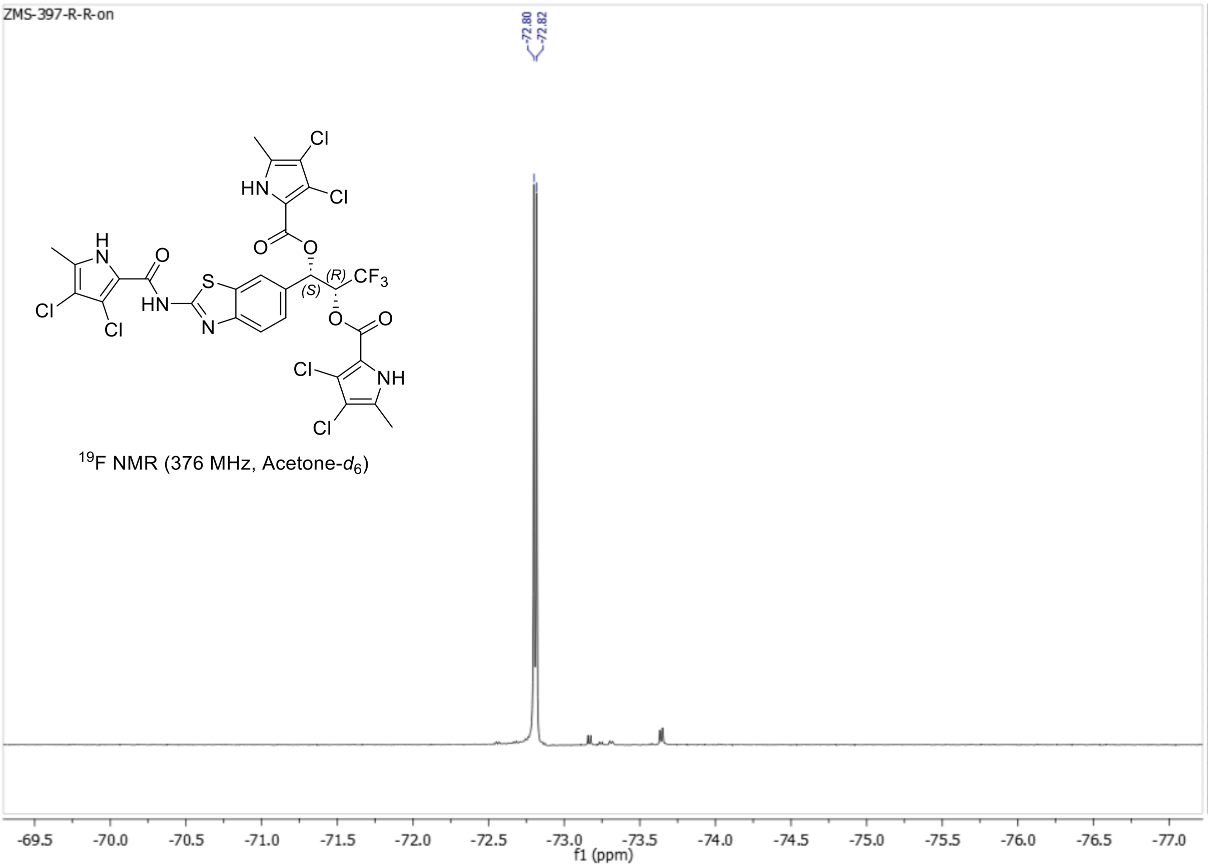

Compound (1*S*,2*R*)-**6'**,^{13}C NMR:

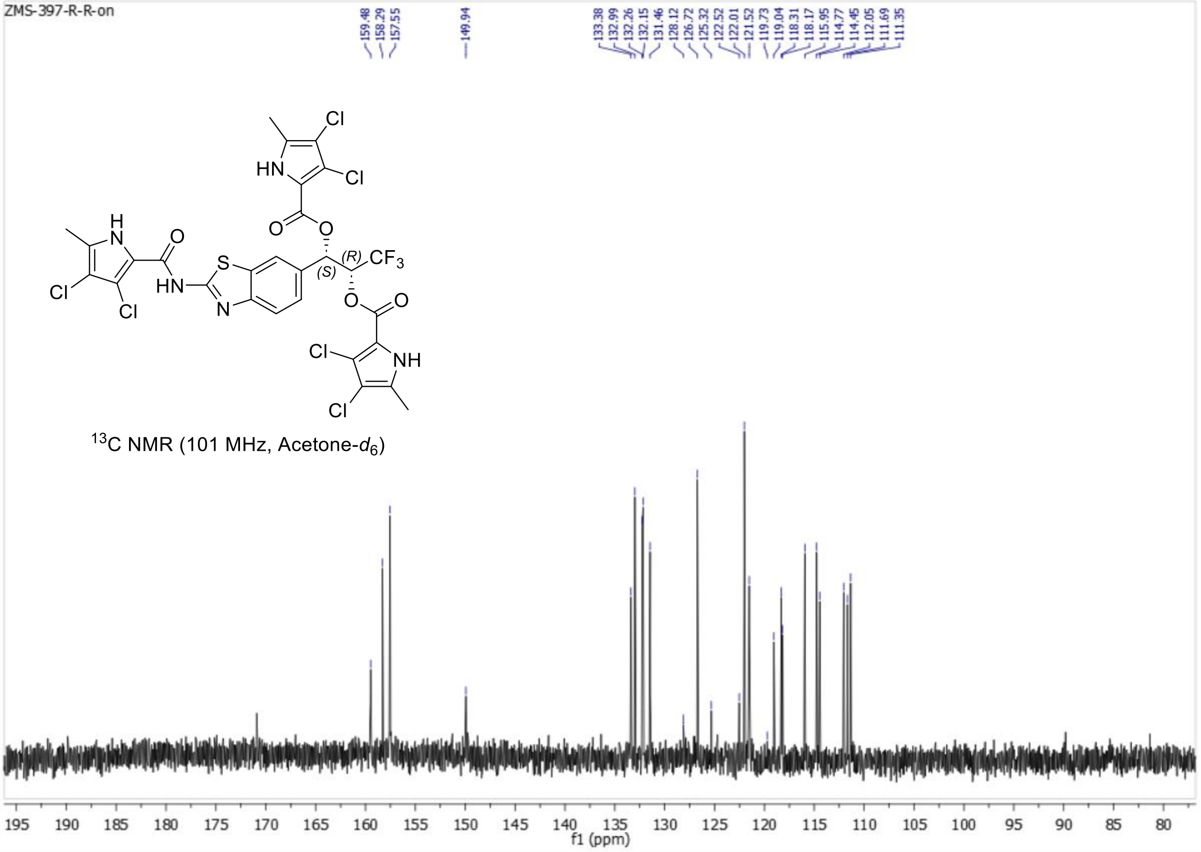

Compound (1*R*,2*S*)-**6,** ^{1}H NMR:

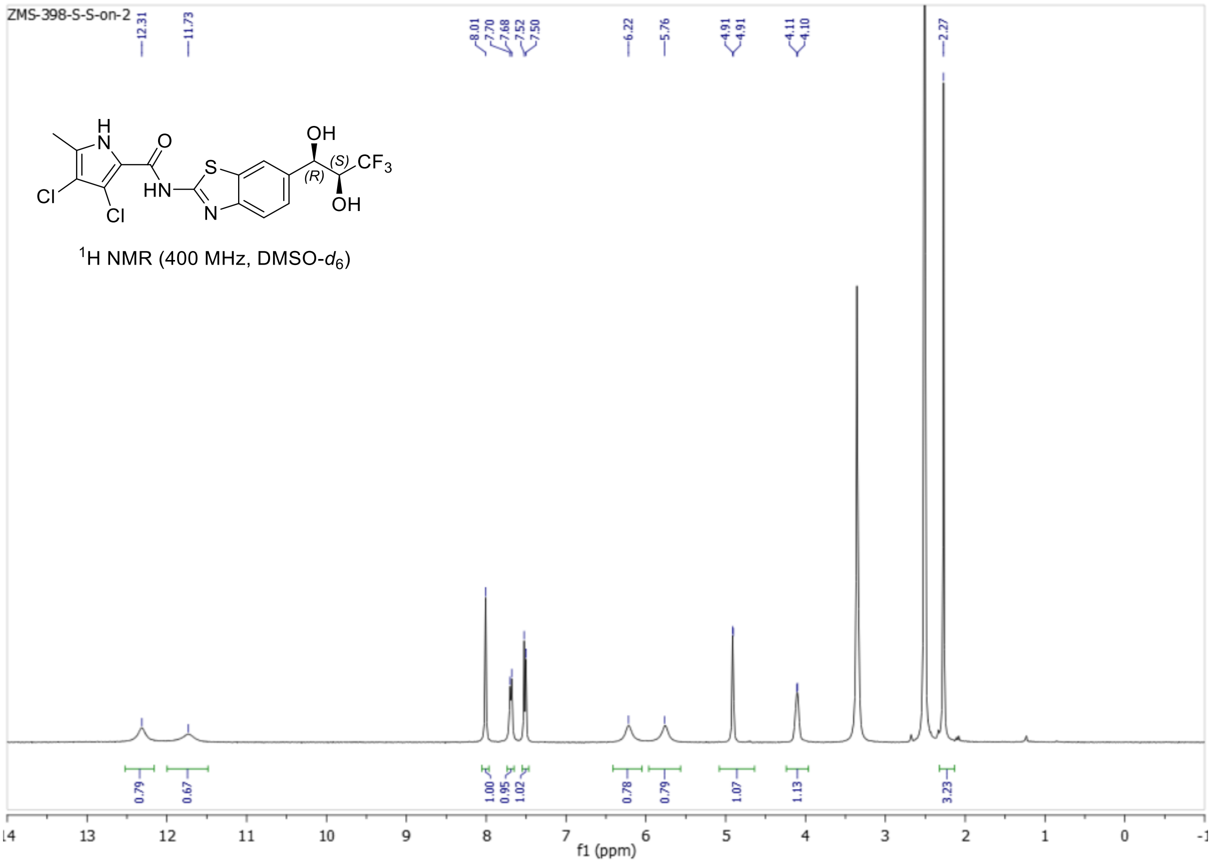

Compound (1*R*,2*S*)-**6,** ^{19}F NMR:

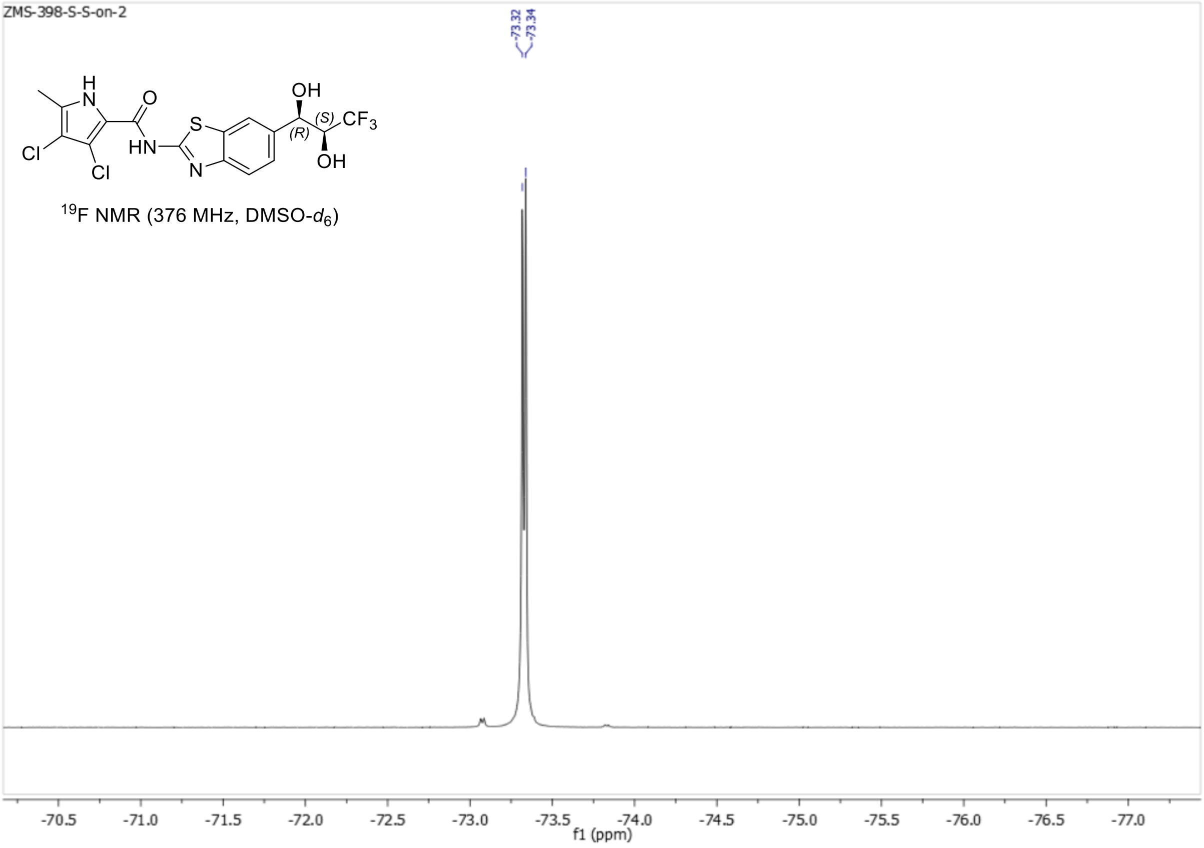

Compound (1*R*, 2*S*)-**6**, ^{13}C NMR:

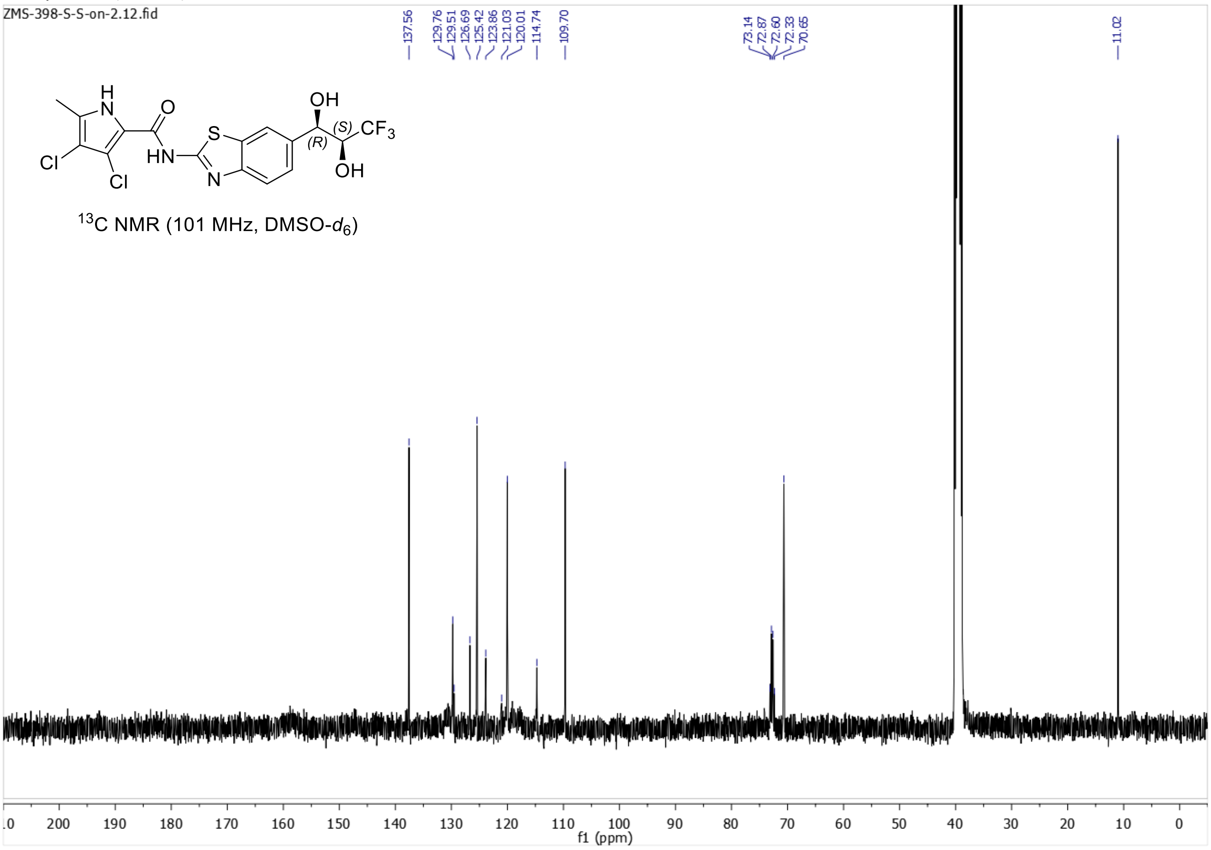

Compound (1*S*,2*R*)-**6**, ^{1}H NMR:

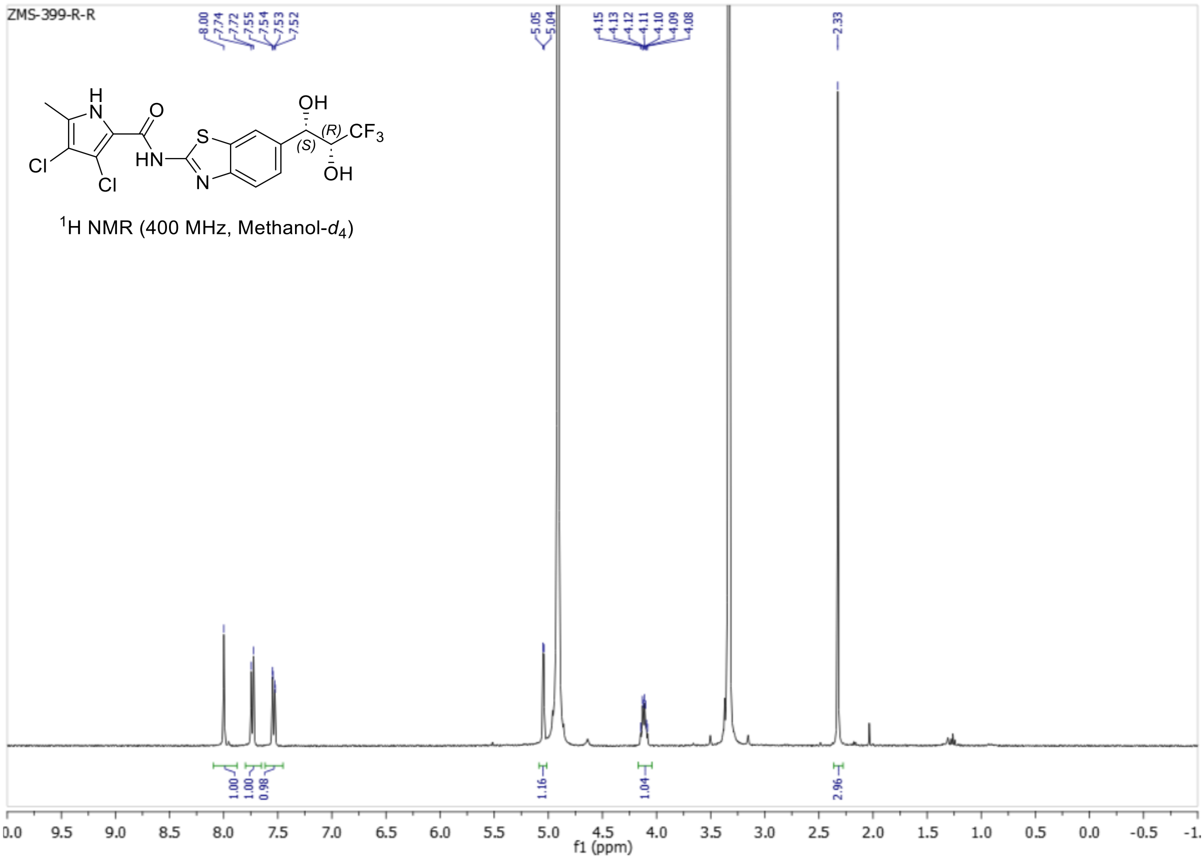

Compound (1*S*,2*R*)-**9**, ^{19}F NMR:

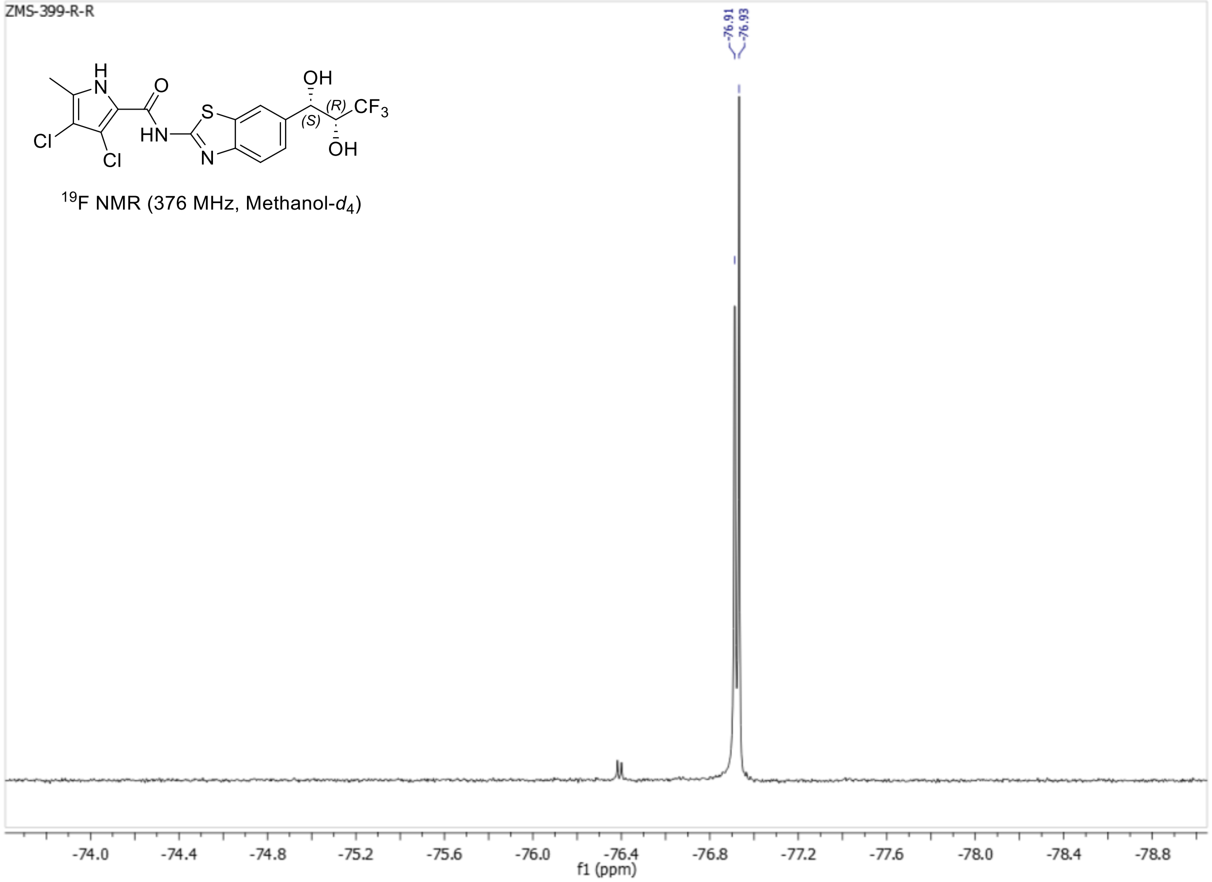

Compound (±)-*anti*-**6**, ^{1}H NMR:

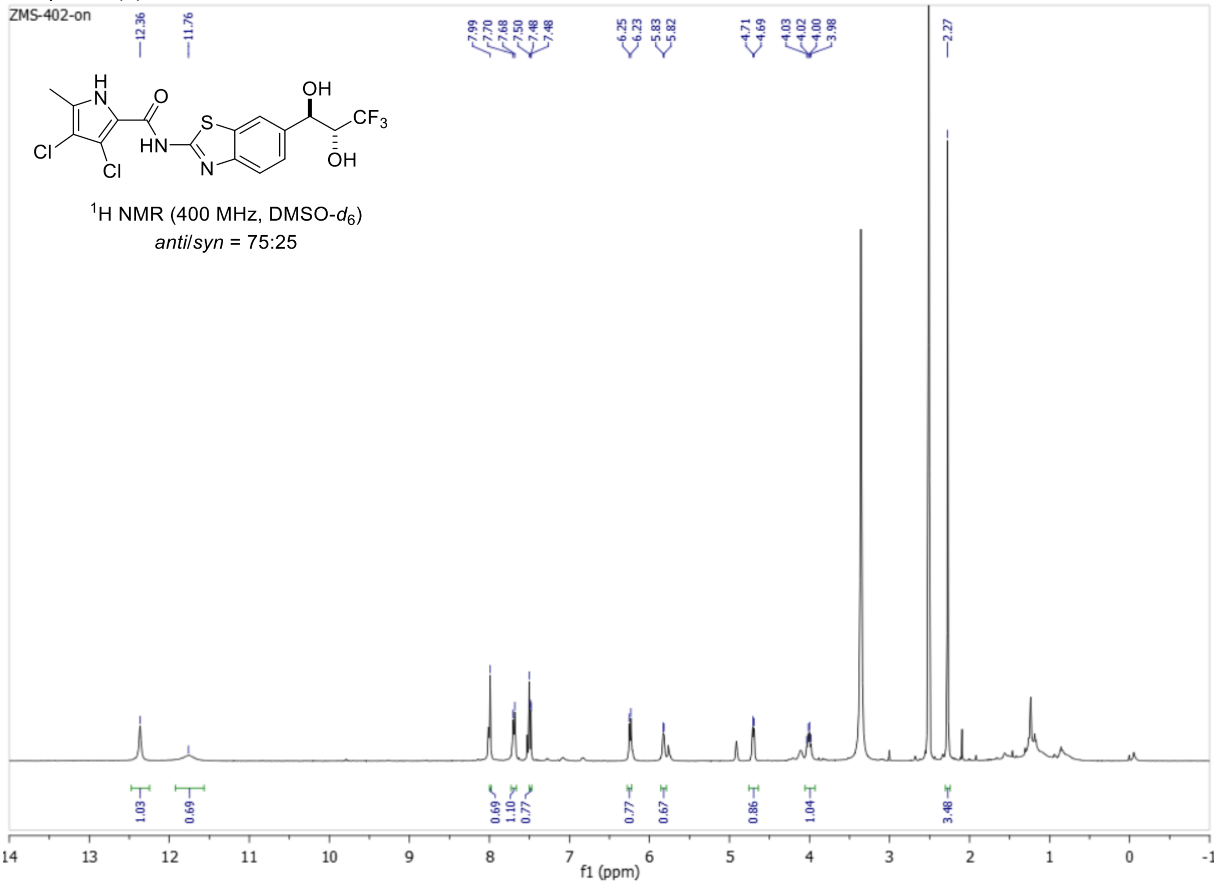

Compound (±)-*anti*-**6**, ^{19}F NMR:

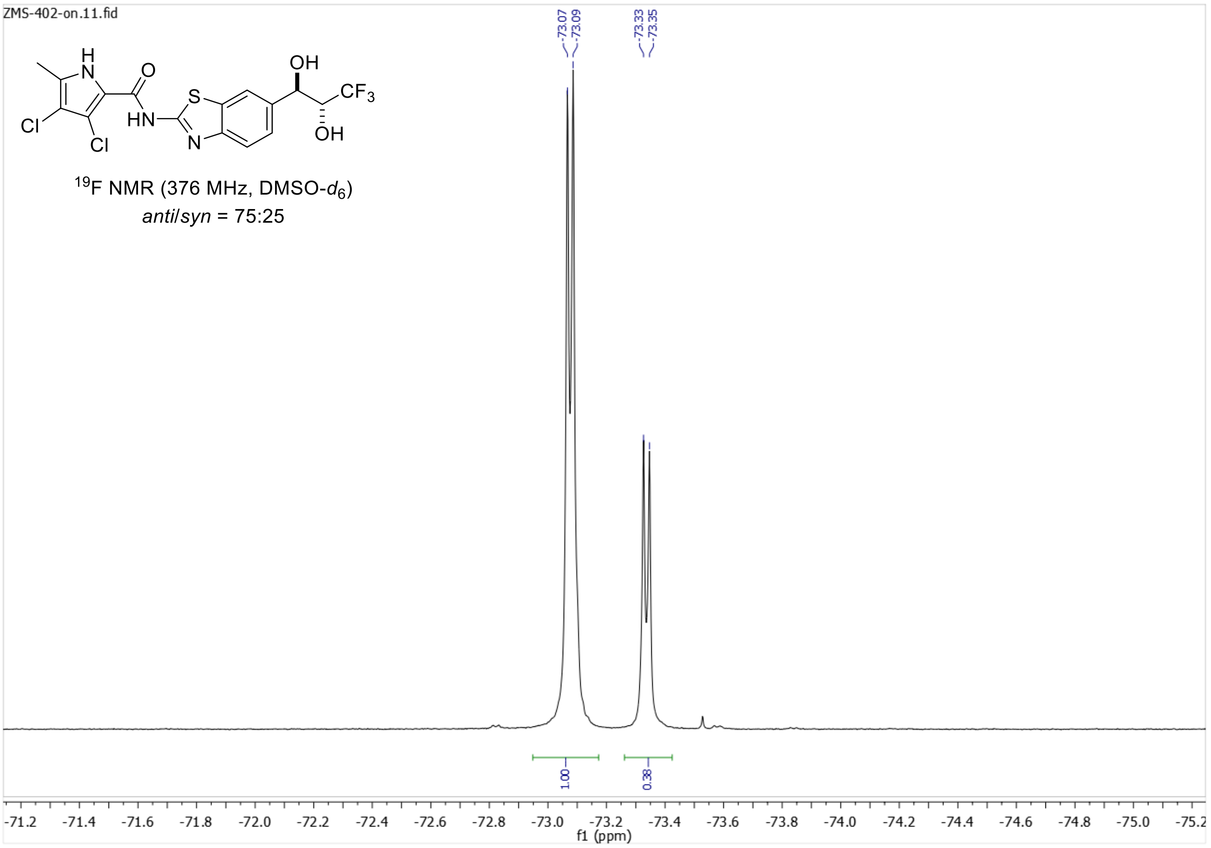

Compound (±)-*anti*-**6**, ^{13}C NMR:

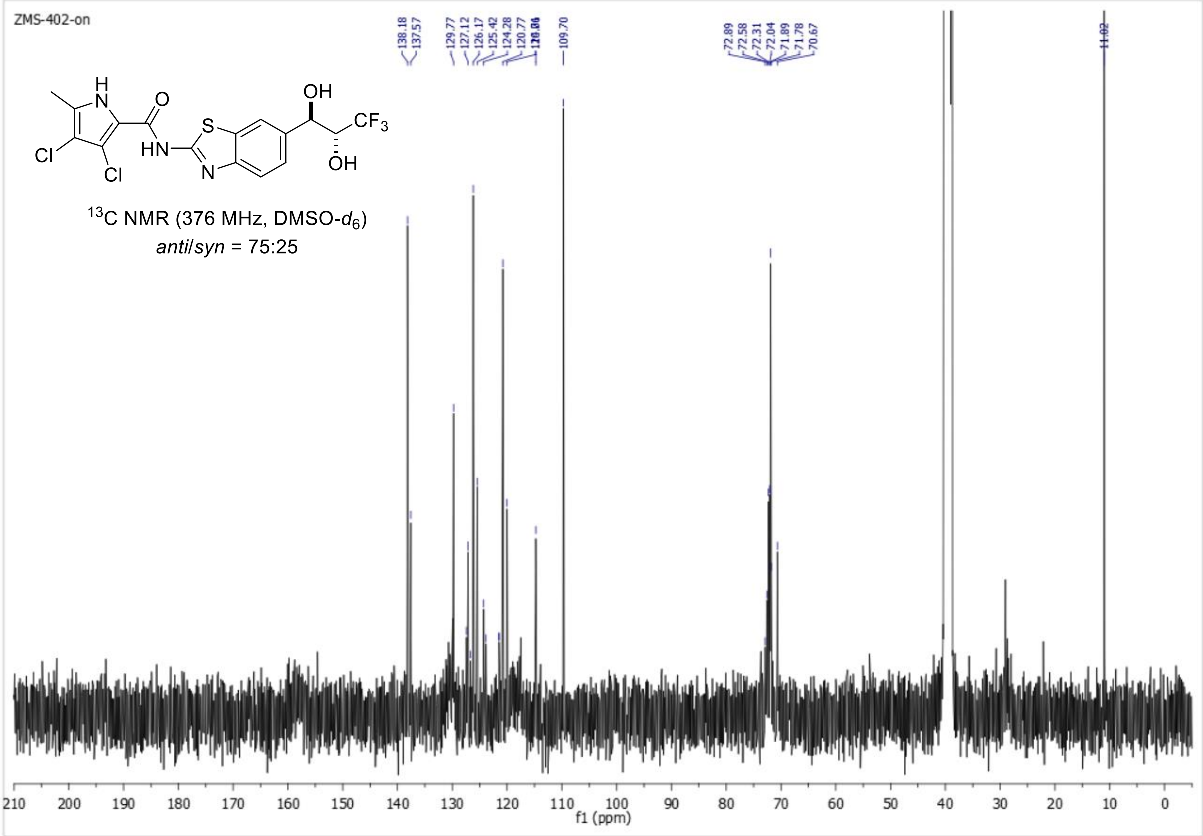

8 Single-crystal X-ray difraction

Crystal structure determination of ***syn*-3e** was performed using a Rigaku OD (Agilent) Gemini single-crystal X-ray diffractometer equipped with fine-focus sealed Cu and Mo Enhance X-ray sources and an Atlas CCD detector. For crystal structure determination of ***syn*-3o**, a Rigaku OD XtaLAB Synergy-S single-crystal X-ray diffractometer equipped with micro-focus sealed Ag and Cu PhotonJet X-ray sources and an Eiger2 R CdTe 1M hybrid pixel detector was used. Both datasets were measured with Cu *Kα* radiation at low temperatures. Data acquisition and processing were performed using *CrysAlis^{Pro}* software.[17] An empirical absorption correction using spherical harmonics implemented in the *SCALE3 ABSPACK* scaling algorithm was applied. An additional analytical numeric absorption correction using a multifaceted crystal model[18] was used for the dataset ***syn*-3e**, whereas numerical absorption correction based on gaussian integration over a multifaceted crystal model was employed for the dataset ***syn*-3o**. Crystal structures were solved using *olex2.solve*[19] and refined with *SHELXL*[20] within the program *Olex2* (v. 1.5).[19] Molecular graphics were created with *Diamond*[21] software. The positions of hydrogen atoms and their isotropic thermal parameters were freely refined.[22]

Table S4: Summary of the crystal data and structure refinements

**Compound**	***syn*-3e**	***syn*-3o**
Formula	$C_9H_8F_3NO_4$	$C_{10}H_{11}F_3O_2$
F_w	251.16	220.19
T [K]	150.0(1)	100.0(1)
Crystal system	Monoclinic	Orthorhombic
Space group	$P2_1$	$P2_12_12_1$
a [Å]	8.63402(9)	4.92394(5)
b [Å]	7.52406(6)	11.45620(11)
c [Å]	15.60788(13)	17.86025(18)
α [°]	90	90
β [°]	96.4100(8)	90
γ [°]	90	90
V [Å^3]	1007.595(15)	1007.491(17)
Z	4	4
ρ_{calc} [g/cm^3]	1.656	1.452
Crystal size [mm]	0.524 × 0.387 × 0.086	0.439 × 0.047 × 0.045
Radiation type	Cu *Kα*	Cu *Kα*
λ [Å]	1.54184	1.54184
μ [mm^{-1}]	1.468	1.194
F(000)	512	456
ϑ_{max} [°]	76.224	76.127
Index ranges	$-10 \le h \le 10$	$-6 \le h \le 6$
	$-9 \le k \le 9$	$-14 \le k \le 14$
	$-19 \le l \le 19$	$-22 \le l \le 22$
Reflections collected	50494	35075
Independent reflections	4216	2115
Reflections with [$I > 2\sigma(I)$]	4191	2068
R_{int}	0.0247	0.0488
R_{sigma}	0.0081	0.0169
Data/restraints/parameters	4216/1/371	2115/0/180
S	1.040	1.053
R_1, wR_2 [$I > 2\sigma(I)$]	0.0268, 0.0730	0.0234, 0.0616
R_1, wR_2 [all data]	0.0269, 0.0731	0.0239, 0.0620
$\Delta\rho_{min}$, $\Delta\rho_{max}$ [eÅ^{-3}]	−0.196, 0.267	−0.193, 0.155
Flack *x* [23]	0.00(2)	−0.02(3)
CCDC deposition number [a]	2236647	2236648

[a] The supplementary crystallographic data for this paper can be obtained free of charge from The Cambridge Crystallographic Data Centre (CCDC) via www.ccdc.cam.ac.uk/structures.

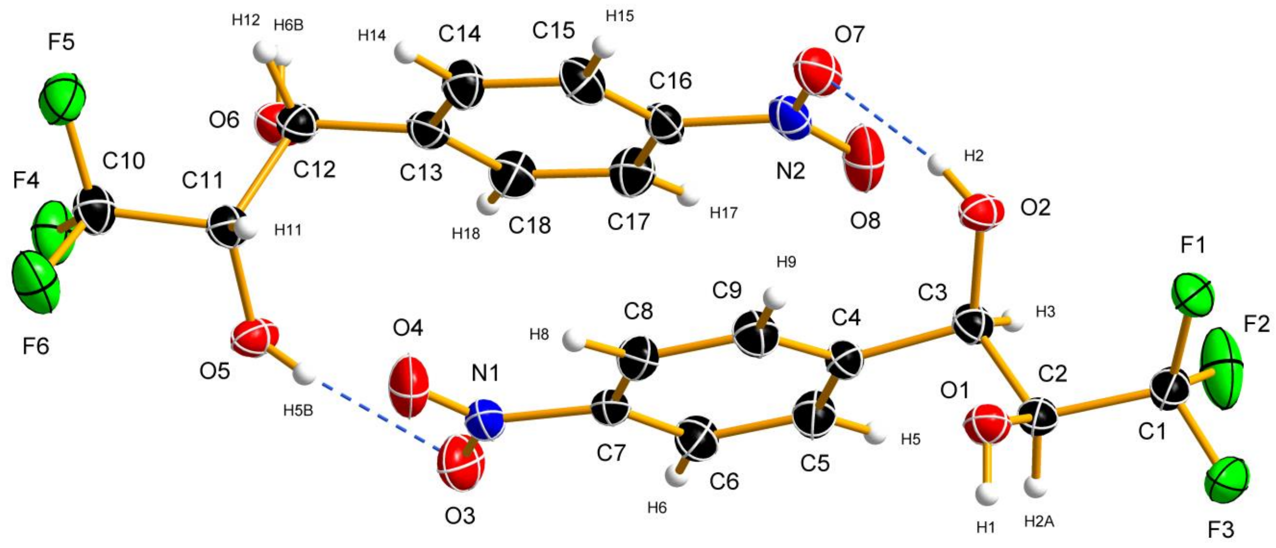

Figure S1. The asymmetric unit (Z' = 2) and atom labelling scheme of the crystal structure ***syn*-3e**. Displacement ellipsoids are depicted at the 50% probability level, hydrogen atoms are drawn as small spheres of arbitrary radius, and hydrogen bonds are shown as blue dashed lines (graph-set[24] motif $R^2{}_2(19)$).

Table S5. Hydrogen-bond geometry (Å, °) in the crystal structure ***syn*-3e**

D–H···*A* [a]	*D*–H	H···*A*	*D*···*A*	*D*–H···*A*
O1–H1···O2^{i}	0.81(4)	2.03(4)	2.822(2)	166(3)
O2–H2···O7	0.80(3)	2.08(3)	2.864(2)	168(3)
O5–H5B···O3	0.75(3)	2.15(3)	2.896(2)	173(3)
O6–H6B···O5^{ii}	0.80(4)	2.04(4)	2.843(2)	174(3)

[a] Symmetry transformations for the generation of equivalent atoms: (i) −*x*, *y* + 1/2, 1 − *z*; (ii) 2 − *x*, *y* − 1/2, 2 − *z*.

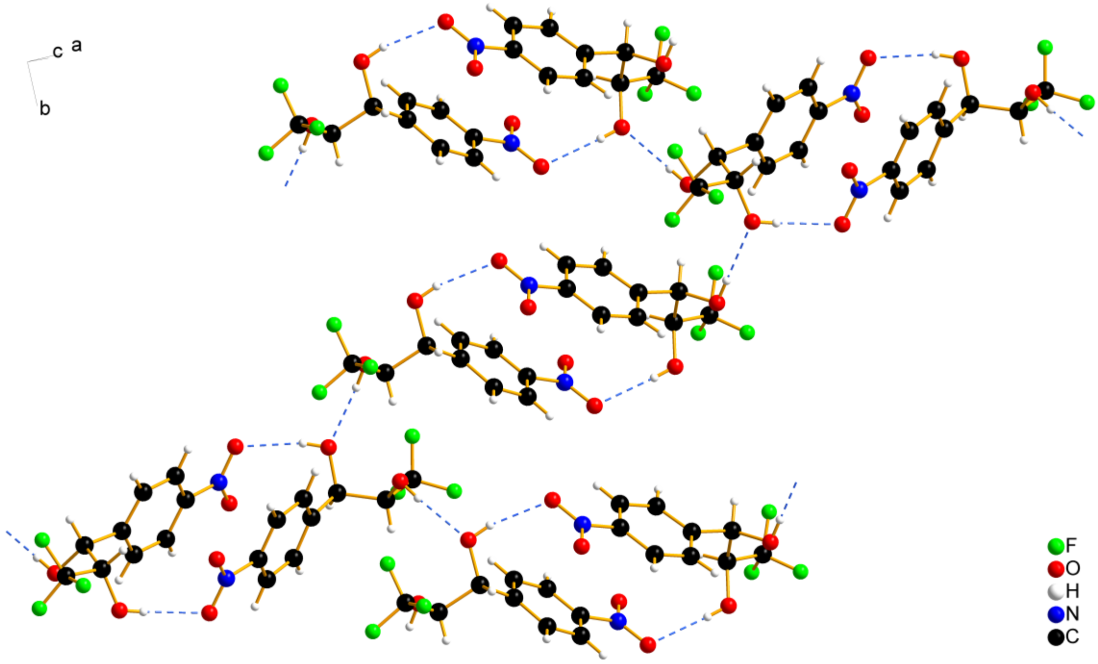

Figure S2. Hydrogen-bonded network (blue dashed line) in the crystal structure of ***syn*-3e**.

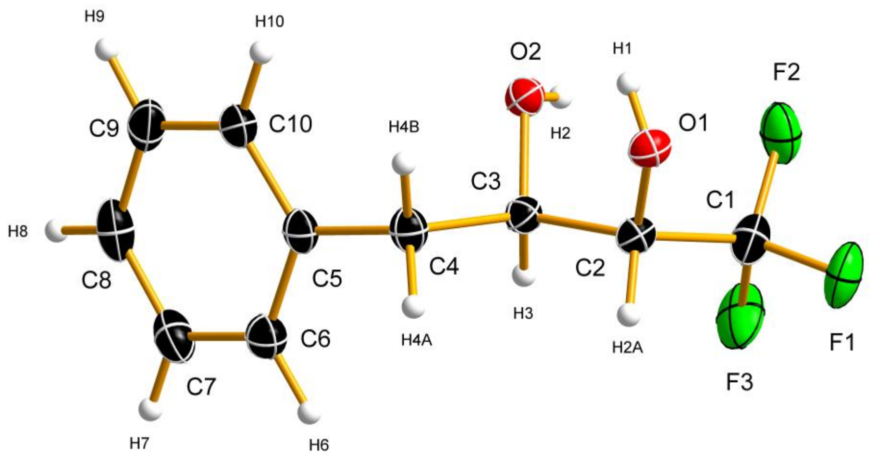

Figure S3. The asymmetric unit and atom labelling scheme of the crystal structure ***syn*-3o**. Displacement ellipsoids are depicted at the 50% probability level and hydrogen atoms are drawn as small spheres of arbitrary radius.

Table S6. Hydrogen-bond geometry (Å, °) in the crystal structure ***syn*-3o**

D–H···*A* [a]	*D*–H	H···*A*	*D*···*A*	*D*–H···*A*
O1–H1···O2^{i}	0.80(2)	2.07(2)	2.7582(14)	144(2)
O2–H2···O1^{ii}	0.85(3)	1.87(3)	2.7160(14)	173(2)

[a] Symmetry transformations for the generation of equivalent atoms: (i) *x* + 1/2, 3/2 − *y*, 1 − *z*; (ii) *x* − 1, *y*, *z*.

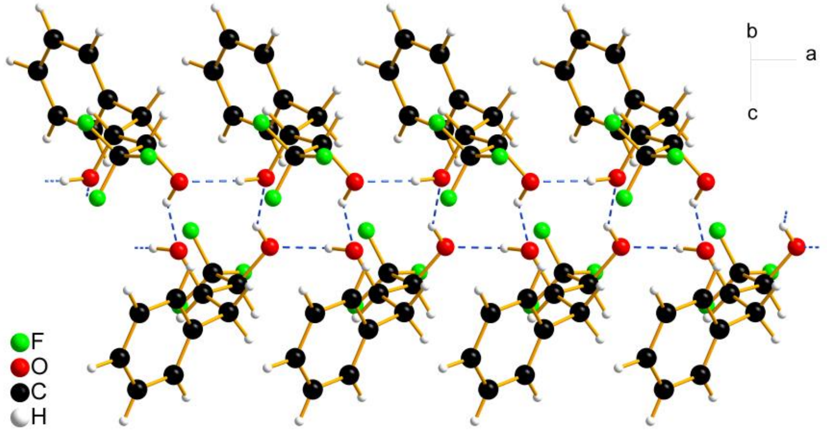

Figure S4. Hydrogen-bonding chain (blue dashed line) parallel to *a*-crystallographic axis in the crystal structure of ***syn*-3o**.

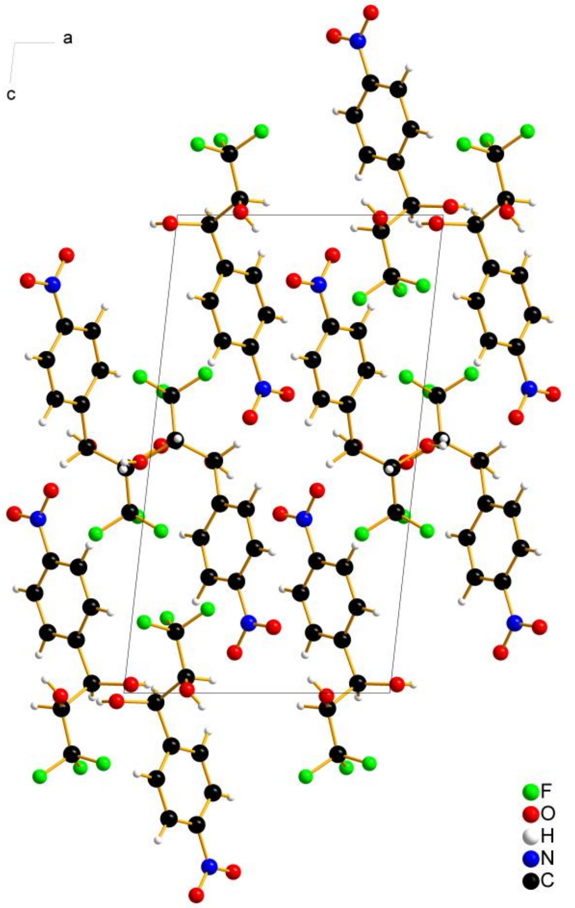

Figure S5. The crystal packing and the unit cell of ***syn*-3e** crystal structure viewed along the *b*-crystallographic axis.

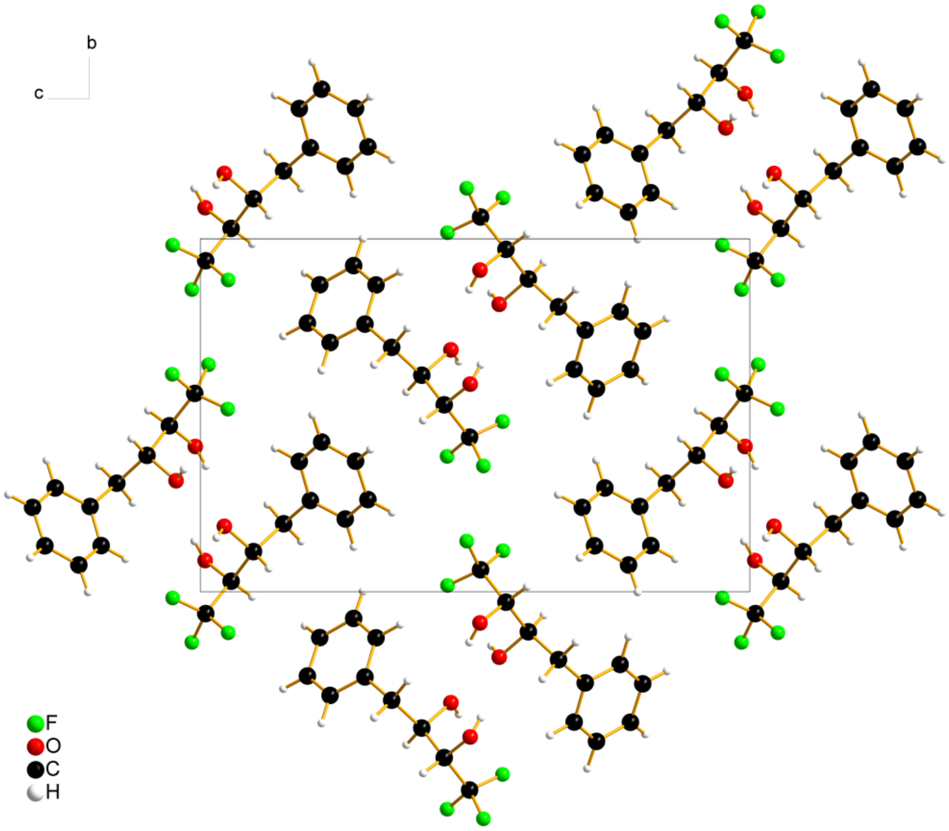

Figure S6. The crystal packing and the unit cell of ***syn*-3o** crystal structure viewed along the *a*-crystallographic axis.

9 Theoretical calculations

Methods

DFT calculations were performed using the Gaussian 16 software.[25] The geometries of reactants, catalyst, transition starts and final states were optimised at the M06-2X/6-31+G(d,p) level Ruthenium was described with an effective core potential (LANL2DZ).[26–28] The Gibbs free energies were computed using the standard frequency analysis approach, accounting for the vibrational (in a harmonic approximation), rotational and translational contributions at 298.15 K and 1.0 atm. All stationary points were confirmed to have zero (stable structures) or exactly one (transition states) imaginary frequencies in the vibrational analysis. Transition states were followed using the internal reaction coordinate (IRC) to show that they connect the desired initial and final states.

Energies

Table S7. Gibbs free energies at 298.15 K and 1 atm for the HCOOH-catalysed dehydration of **1a**. All values in hartrees.

Structure	Gibbs free energy
**1a** geminal diol	-871.983205
**1a'** ketone	-795.575141
H_2O	-76.391659
HCOOH	-189.683318
PrC: (**1a** geminal diol + HCOOH)	-1061.670566
TS (**1a** geminal diol + HCOOH ⟶ **1a'** ketone + H_2O + HCOOH)	-1061.637875
Final adduct: (**1a'** – ketone + H_2O + HCOOH)	-1061.642451

Table S8. Gibbs free energies at 298.15 K and 1 atm for the uncatalysed hydrogenation of (ketone) **1a’** to **2a**. All values in hartrees.

Structure	Gibbs free energy
**1a'** ketone	-795.575141
**2a**	-796.780420
HCOOH	-189.683318
CO_2	-188.525517
PrC: (**1a'** ketone + HCOOH + HCOOH)	-1174.941777
TS (**1a'** ketone + HCOOH + HCOOH ⟶ **2a** + CO_2 + HCOOH)	-1174.878522
Final adduct: (**2a** + CO_2 + HCOOH)	-1174.969367

Table S9. Gibbs free energies at 298.15 K and 1 atm for the uncatalysed intramolecular enolization of **2a**. All values in hartrees.

Structure	Gibbs free energy
**2a** ketone	-796.771493
**TS**	-796.679611
**2a** enol	-796.760698

Table S10. Gibbs free energies at 298.15 K and 1 atm for the HCO_2H/Et_3N-catalysed enolization of **2a**. All values in hartrees.

Structure	Gibbs free energy
**2a** ketone + HCO_2H/Et_3N	-1278.556619
**TS**	-1278.527655
**2a** enol + HCO_2H/Et_3N	-1278.548856

Table S11. Gibbs free energies at 298.15 K and 1 atm for the first half of the catalytic cycle (HCOOH + catalyst oxidised → CO_2 + catalyst reduced). All values in hartrees.

**Structure**	Gibbs free energy
HCOOH	-189.683318
CO_2	-188.525517
Catalyst (oxidised)	-1912.064699
Catalyst (reduced)	-1913.251555
PrC: (HCOOH + catalyst ox)	-2101.744964
TS (HCOOH + catalyst ox ⟶ CO_2 + catalyst red)	-2101.736995
Adduct: (CO_2 + catalyst red)	-2101.773583

Table S12. Gibbs free energies at 298.15 K and 1 atm for the second half of the catalytic cycle (2a + catalyst reduced → 3a + catalyst oxidised). All values in hartrees.

**Type of attack**	***Si-face on (S)-2a***	***Re-face on (S)-2a***	***Si-face on (R)-2a***	***Re-face on (R)-2a***
Substrate **2a**	-796.774090	-796.774090	-796.774090	-796.774090
Catalyst reduced	-1913.250895	-1913.250895	-1913.250895	-1913.250895
PrC	-2710.036182	-2710.033238	-2710.039468	-2710.026442
TS	-2710.015830	-2710.011691	-2710.012368	-2710.005933
Final adduct	-2710.028038	-2710.026668	-2710.029234	-2710.027312
Product **3a**	-797.955539 (*1R,2S*)-**3a**	-797.953248 (*1S,2S*)-**3a**	-797.953248 (*1R,2R*)-**3a**	-797.955539 (*1S,2R*)-**3a**
Catalyst oxidised	-1912.064693	-1912.064693	-1912.064693	-1912.064693

Geometries

1a geminal diol

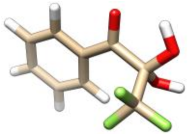

H 2.12011 2.13839 -0.82083
C 2.32021 1.13643 -0.45622
C 3.58682 0.57805 -0.55664
H 4.39448 1.14412 -1.00867
C 3.81693 -0.71196 -0.07432
H 4.80758 -1.14959 -0.15007
C 2.77896 -1.43889 0.50475
H 2.96051 -2.43885 0.88431
C 1.50347 -0.88914 0.60120
H 0.70351 -1.45554 1.05981
C 1.26910 0.40476 0.11742
C -0.04587 1.08704 0.18903
C -1.36918 0.34759 0.56840
O -0.16968 2.27430 -0.04296
O -2.38211 1.26888 0.72033
O -1.15715 -0.37493 1.74257
C -1.82938 -0.61562 -0.54856
F -0.96283 -1.61671 -0.74885
H -2.01637 -0.46917 2.17806
H -2.01024 2.14594 0.51471
F -1.97173 0.03918 -1.70611
F -3.00704 -1.16145 -0.22140

PrC: 1a geminal diol + HCOOH

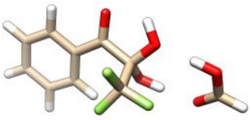

H -3.14142 2.14097 -0.35227
C -3.23370 1.06037 -0.32450

C -4.47749 0.44977 -0.24796
H -5.37730 1.05481 -0.21181
C -4.56576 -0.94364 -0.21786
H -5.53826 -1.42281 -0.16008
C -3.41041 -1.72101 -0.26310
H -3.48174 -2.80337 -0.24690
C -2.15798 -1.11669 -0.33146
H -1.26261 -1.72285 -0.37959
C -2.06671 0.28146 -0.36019
C -0.78482 1.01839 -0.44385
C 0.59755 0.32676 -0.22255
O -0.73540 2.20983 -0.68737
O 1.59411 1.26784 -0.49482
O 0.68458 -0.77858 -1.03502
C 0.80429 -0.05874 1.26054
F -0.00351 -1.04929 1.64778
H 1.62713 -1.05336 -1.10069
H 1.14604 2.10475 -0.72730
F 0.57940 0.99303 2.05869
F 2.06817 -0.46490 1.45019
C 4.31860 -0.69549 -0.82147
O 4.26660 0.55954 -0.42875
H 5.35027 -1.06545 -0.85261
O 3.36613 -1.38596 -1.12104
H 3.33177 0.86933 -0.40823

TS: 1a geminal diol + HCOOH→ 1a ketone + H_2O + HCOOH

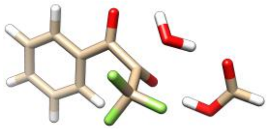

H -3.12520 2.09741 -0.63214
C -3.20870 1.02114 -0.52243
C -4.44662 0.39477 -0.48355
H -5.35496 0.98315 -0.55994
C -4.51939 -0.99352 -0.34611
H -5.48776 -1.48339 -0.31751
C -3.35523 -1.75256 -0.24717
H -3.41456 -2.83116 -0.14838
C -2.10975 -1.13064 -0.27677
H -1.20657 -1.72685 -0.21415
C -2.03555 0.26091 -0.41418
C -0.74576 0.99355 -0.46141
C 0.53240 0.19780 -0.07643
O -0.66583 2.15211 -0.81858
O 1.73968 1.64273 -0.11659
O 0.91429 -0.68670 -0.85276
C 0.78777 -0.00728 1.43455
F 0.07220 -1.06261 1.85752
H 2.39051 -1.37387 -0.85074
H 1.28920 2.30350 -0.66874
F 0.41922 1.04889 2.16012
F 2.07441 -0.27509 1.66283
C 4.16687 -0.72454 -1.12140
O 3.89607 0.46468 -1.09023
H 5.19160 -1.07655 -1.28996
O 3.32642 -1.71295 -0.96977
H 2.54394 1.27032 -0.56983

Final adduct: 1a ketone + H_2O + HCOOH

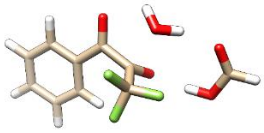

H -3.17205 2.18653 -0.42054
C -3.28834 1.10764 -0.41452
C -4.54282 0.51467 -0.44131
H -5.43437 1.13236 -0.46631
C -4.65528 -0.87821 -0.43579
H -5.63720 -1.34051 -0.45772
C -3.51443 -1.67720 -0.40277
H -3.60482 -2.75811 -0.40356
C -2.25283 -1.08850 -0.36937
H -1.36902 -1.71823 -0.35251
C -2.13946 0.30625 -0.37572
C -0.82263 0.99206 -0.34830
C 0.41066 0.08807 -0.10651
O -0.68930 2.18101 -0.53483
O 1.86794 1.87231 0.26906
O 0.90961 -0.53268 -1.01761
C 0.78462 -0.26895 1.34922
F 0.13442 -1.41121 1.66046
H 2.60039 -1.24013 -1.05042
H 1.40938 2.56307 -0.22485
F 0.39908 0.65870 2.21724
F 2.08576 -0.50547 1.47182

C 4.36851 -0.55384 -1.12238
O 4.07542 0.61023 -0.96455
H 5.40158 -0.89827 -1.25621
O 3.52100 -1.56507 -1.16278
H 2.66740 1.61687 -0.22304

1a ketone

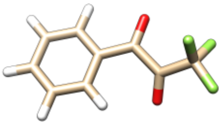

H -1.99953 2.25366 -0.30452
C -2.33088 1.23248 -0.14580
C -3.67752 0.92417 -0.01108
H -4.42260 1.71059 -0.06980
C -4.07000 -0.39932 0.20293
H -5.12306 -0.64000 0.31038
C -3.11605 -1.41202 0.28060
H -3.42367 -2.43845 0.44957
C -1.76389 -1.11145 0.13765
H -1.02849 -1.90549 0.19000
C -1.36931 0.21493 -0.07694
C 0.05103 0.61955 -0.19574
C 1.14603 -0.44767 -0.38099
O 0.43275 1.77035 -0.20416
O 0.99601 -1.48020 -0.97230
C 2.54622 -0.06071 0.16443
F 2.43621 0.45782 1.39318
F 3.14001 0.83203 -0.62899
F 3.32084 -1.14110 0.23422

PrC: 1a ketone + HCOOH + HCOOH

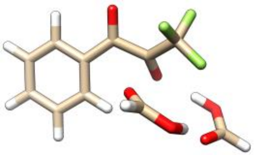

H -3.82099 1.74954 -0.15324
C -3.66760 0.67546 -0.18174
C -4.74029 -0.20636 -0.18046
H -5.75599 0.17413 -0.15312
C -4.50708 -1.58281 -0.21455
H -5.34535 -2.27275 -0.21283
C -3.20342 -2.07571 -0.24762
H -3.02685 -3.14595 -0.26649
C -2.12315 -1.19783 -0.25351
H -1.10933 -1.58009 -0.26114
C -2.35734 0.18170 -0.22288
C -1.26172 1.17533 -0.21692
C 0.16608 0.75402 -0.58213
O -1.41087 2.36487 -0.03144
O 0.42751 -0.06218 -1.43012
C 1.31819 1.61657 -0.00313
F 1.07721 2.10458 1.20674
H 2.40512 -1.46670 1.40972
F 1.55113 2.63623 -0.83828
F 2.43114 0.87185 0.05437
C 0.99353 -0.79209 2.49747
O 0.24131 -0.49814 1.59063
H 0.73912 -0.64542 3.55452
O 2.18928 -1.31767 2.35942
C 3.48443 -1.81272 -1.26866
O 3.04891 -1.99203 -0.15151
H 4.42534 -2.25418 -1.61911
O 2.91294 -1.09264 -2.20924
H 2.07125 -0.70334 -1.88109

TS: 1a ketone + HCOOH + HCOOH → 2a + CO_2 + HCOOH

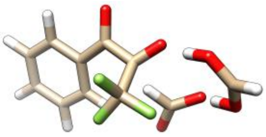

H 3.38956 -2.10677 -1.00710
C 3.46349 -1.10504 -0.59703
C 4.69214 -0.56703 -0.24101
H 5.59619 -1.15433 -0.36320
C 4.76035 0.73090 0.26953
H 5.72100 1.15587 0.54341

C 3.59857 1.48321 0.42338
H 3.64916 2.49691 0.80611
C 2.35810 0.94447 0.08364
H 1.47884 1.56993 0.20140
C 2.28791 -0.35704 -0.42740
C 1.03617 -1.01967 -0.86881
C -0.38818 -0.67009 -0.32992
O 1.02571 -1.96038 -1.62726
O -1.34880 -0.98277 -1.02094
C -0.61998 -0.60856 1.20213
F 0.27019 0.11887 1.86691
H -3.08041 1.28570 -0.56687
F -0.56967 -1.86339 1.66163
F -1.84016 -0.11734 1.45774
C -0.90924 1.93999 -0.57108
O -0.27568 2.91902 -0.26131
H -0.31798 0.97553 -0.44934
O -2.06817 1.74197 -1.05155
C -4.44235 -0.26832 -0.13568
O -4.14017 0.93893 -0.18583
H -5.45770 -0.52741 0.16950
O -3.69567 -1.26427 -0.40457
H -2.71866 -1.06112 -0.64652

Final adduct: 2a + CO_2 + HCOOH

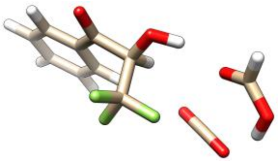

H 4.09057 -1.81144 -0.24505
C 3.93168 -0.73845 -0.27457
C 4.99887 0.15013 -0.24839
H 6.01555 -0.22544 -0.19426
C 4.76159 1.52530 -0.29211
H 5.59473 2.22107 -0.27314
C 3.45593 2.00595 -0.35977
H 3.26925 3.07430 -0.39352
C 2.38335 1.11698 -0.38285
H 1.37543 1.51582 -0.42630
C 2.61600 -0.26236 -0.34091
C 1.52449 -1.28638 -0.38459
C 0.05071 -0.83008 -0.32855
O 1.76406 -2.46744 -0.46611
O -0.75119 -1.82376 -0.87949
C -0.36114 -0.59163 1.12747
F 0.32973 0.41826 1.68500
H -4.81533 0.71386 0.00453
F -0.19172 -1.67240 1.88762
F -1.66978 -0.25361 1.19982
C -2.38558 2.10478 0.01056
O -1.31163 2.13352 -0.42552
H -0.06860 0.12838 -0.84499
O -3.46271 2.12213 0.45771
C -4.20413 -0.74514 -1.01678
O -5.15137 -0.14182 -0.32152
H -4.52938 -1.73439 -1.35862
O -3.11219 -0.27257 -1.25309
H -1.57664 -1.40747 -1.17349

2a ketone

H -2.26108 2.23632 0.18499
C -2.36319 1.16193 0.07089
C -3.61470 0.56395 -0.01613
H -4.51041 1.17388 0.03886
C -3.71800 -0.81837 -0.17815
H -4.69529 -1.28505 -0.24941
C -2.56731 -1.59985 -0.25646
H -2.64469 -2.67204 -0.40353
C -1.31077 -1.00604 -0.15529
H -0.42478 -1.62455 -0.26403
C -1.20349 0.37991 0.01380
C 0.10410 1.10132 0.11041
C 1.33115 0.43264 0.77235
O 0.21637 2.25045 -0.25343
O 1.04050 -0.43637 1.82727
C 2.25349 -0.24026 -0.24698
F 2.51505 0.57261 -1.27593
H 1.91243 1.26224 1.18414
H 0.33193 -1.04466 1.58293
F 1.71135 -1.36891 -0.74895
F 3.41463 -0.58260 0.31919

TS: 2a ketone → 2a enol

H -2.26108 2.23632 0.18499
C -2.36319 1.16193 0.07089
C -3.61470 0.56395 -0.01613
H -4.51041 1.17388 0.03886
C -3.71800 -0.81837 -0.17815
H -4.69529 -1.28505 -0.24941
C -2.56731 -1.59985 -0.25646
H -2.64469 -2.67204 -0.40353
C -1.31077 -1.00604 -0.15529
H -0.42478 -1.62455 -0.26403
C -1.20349 0.37991 0.01380
C 0.10410 1.10132 0.11041
C 1.33115 0.43264 0.77235
O 0.21637 2.25045 -0.25343
O 1.04050 -0.43637 1.82727
C 2.25349 -0.24026 -0.24698
F 2.51505 0.57261 -1.27593
H 1.91243 1.26224 1.18414
H 0.33193 -1.04466 1.58293
F 1.71135 -1.36891 -0.74895
F 3.41463 -0.58260 0.31919

2a enol

H -2.26108 2.23632 0.18499
C -2.36319 1.16193 0.07089
C -3.61470 0.56395 -0.01613
H -4.51041 1.17388 0.03886
C -3.71800 -0.81837 -0.17815
H -4.69529 -1.28505 -0.24941
C -2.56731 -1.59985 -0.25646
H -2.64469 -2.67204 -0.40353
C -1.31077 -1.00604 -0.15529
H -0.42478 -1.62455 -0.26403
C -1.20349 0.37991 0.01380
C 0.10410 1.10132 0.11041
C 1.33115 0.43264 0.77235
O 0.21637 2.25045 -0.25343
O 1.04050 -0.43637 1.82727
C 2.25349 -0.24026 -0.24698
F 2.51505 0.57261 -1.27593
H 1.91243 1.26224 1.18414
H 0.33193 -1.04466 1.58293
F 1.71135 -1.36891 -0.74895
F 3.41463 -0.58260 0.31919

PRC: 2a ketone + HCO_2H + Et_3N

H -0.14974 2.53766 0.67209
C -0.21224 1.80424 1.47006
C 0.36882 2.05213 2.70826
H 0.86876 2.99755 2.89164
C 0.30911 1.08184 3.71059
H 0.75836 1.27672 4.67970
C -0.32252 -0.13774 3.46992
H -0.36934 -0.88986 4.25070
C -0.88353 -0.40372 2.22339
H -1.34695 -1.36258 2.02622
C -0.83765 0.57459 1.22180
C -1.42561 0.36284 -0.12772
C -1.49634 -1.05173 -0.74494
O -1.79316 1.29278 -0.82590
O -0.60807 -1.96730 -0.20924
C -2.90190 -1.62997 -0.59835
F -3.82160 -0.80511 -1.11766
H -1.35964 -0.88858 -1.82567
H 0.33930 -1.61727 -0.32575
F -3.22727 -1.83268 0.69055
F -3.00263 -2.80467 -1.22806
N 1.90653 -1.11625 -0.47855
C 1.89784 0.15029 -1.23028
C 2.30313 -1.01711 0.93415
C 2.43391 -2.27882 -1.20716
H 1.68714 -2.55389 -1.96406
H 2.46463 -3.11157 -0.49490
C 3.78828 -2.12908 -1.90463
H 1.93325 -0.05294 1.30195
C 3.78856 -1.16985 1.26928
H 1.74376 -1.78704 1.48357
H 1.04481 0.74930 -0.88988
H 1.69740 -0.10373 -2.27836
C 3.13372 1.04605 -1.13155
H 4.40928 -0.43369 0.75367
H 3.74226 -1.37493 -2.69549
H 3.92569 -1.03684 2.34679
H 4.05750 -3.08039 -2.37338
H 4.58689 -1.85121 -1.21353
H 4.15375 -2.16896 1.01359
H 3.27465 1.39714 -0.10437
H 2.97261 1.93066 -1.75234
H 4.04797 0.54241 -1.45539
C -0.11799 4.05492 -1.73573

O 0.71845 3.22219 -1.46969
H 0.11840 5.03658 -2.16840
O -1.42012 3.92315 -1.54461
H -1.61326 3.02150 -1.19935

TS: 2a ketone + HCO_2H + Et_3N → 2a enol + HCO_2H + Et_3N

H -0.14974 2.53766 0.67209
C -0.21224 1.80424 1.47006
C 0.36882 2.05213 2.70826
H 0.86876 2.99755 2.89164
C 0.30911 1.08184 3.71059
H 0.75836 1.27672 4.67970
C -0.32252 -0.13774 3.46992
H -0.36934 -0.88986 4.25070
C -0.88353 -0.40372 2.22339
H -1.34695 -1.36258 2.02622
C -0.83765 0.57459 1.22180
C -1.42561 0.36284 -0.12772
C -1.49634 -1.05173 -0.74494
O -1.79316 1.29278 -0.82590
O -0.60807 -1.96730 -0.20924
C -2.90190 -1.62997 -0.59835
F -3.82160 -0.80511 -1.11766
H -1.35964 -0.88858 -1.82567
H 0.33930 -1.61727 -0.32575
F -3.22727 -1.83268 0.69055
F -3.00263 -2.80467 -1.22806
N 1.90653 -1.11625 -0.47855
C 1.89784 0.15029 -1.23028
C 2.30313 -1.01711 0.93415
C 2.43391 -2.27882 -1.20716
H 1.68714 -2.55389 -1.96406
H 2.46463 -3.11157 -0.49490
C 3.78828 -2.12908 -1.90463
H 1.93325 -0.05294 1.30195
C 3.78856 -1.16985 1.26928
H 1.74376 -1.78704 1.48357
H 1.04481 0.74930 -0.88988
H 1.69740 -0.10373 -2.27836
C 3.13372 1.04605 -1.13155
H 4.40928 -0.43369 0.75367
H 3.74226 -1.37493 -2.69549
H 3.92569 -1.03684 2.34679
H 4.05750 -3.08039 -2.37338
H 4.58689 -1.85121 -1.21353
H 4.15375 -2.16896 1.01359
H 3.27465 1.39714 -0.10437
H 2.97261 1.93066 -1.75234
H 4.04797 0.54241 -1.45539
C -0.11799 4.05492 -1.73573
O 0.71845 3.22219 -1.46969
H 0.11840 5.03658 -2.16840
O -1.42012 3.92315 -1.54461
H -1.61326 3.02150 -1.19935

Final adduct: 2a enol + HCO_2H + Et_3N

H -0.14974 2.53766 0.67209
C -0.21224 1.80424 1.47006
C 0.36882 2.05213 2.70826
H 0.86876 2.99755 2.89164
C 0.30911 1.08184 3.71059
H 0.75836 1.27672 4.67970
C -0.32252 -0.13774 3.46992
H -0.36934 -0.88986 4.25070
C -0.88353 -0.40372 2.22339
H -1.34695 -1.36258 2.02622
C -0.83765 0.57459 1.22180
C -1.42561 0.36284 -0.12772
C -1.49634 -1.05173 -0.74494
O -1.79316 1.29278 -0.82590
O -0.60807 -1.96730 -0.20924
C -2.90190 -1.62997 -0.59835
F -3.82160 -0.80511 -1.11766
H -1.35964 -0.88858 -1.82567
H 0.33930 -1.61727 -0.32575
F -3.22727 -1.83268 0.69055
F -3.00263 -2.80467 -1.22806
N 1.90653 -1.11625 -0.47855
C 1.89784 0.15029 -1.23028
C 2.30313 -1.01711 0.93415
C 2.43391 -2.27882 -1.20716
H 1.68714 -2.55389 -1.96406
H 2.46463 -3.11157 -0.49490
C 3.78828 -2.12908 -1.90463
H 1.93325 -0.05294 1.30195
C 3.78856 -1.16985 1.26928
H 1.74376 -1.78704 1.48357
H 1.04481 0.74930 -0.88988
H 1.69740 -0.10373 -2.27836
C 3.13372 1.04605 -1.13155
H 4.40928 -0.43369 0.75367
H 3.74226 -1.37493 -2.69549
H 3.92569 -1.03684 2.34679
H 4.05750 -3.08039 -2.37338
H 4.58689 -1.85121 -1.21353
H 4.15375 -2.16896 1.01359

H 3.27465 1.39714 -0.10437
H 2.97261 1.93066 -1.75234
H 4.04797 0.54241 -1.45539
C -0.11799 4.05492 -1.73573
O 0.71845 3.22219 -1.46969
H 0.11840 5.03658 -2.16840
O -1.42012 3.92315 -1.54461
H -1.61326 3.02150 -1.19935

PrC: HCOOH + catalyst oxidised

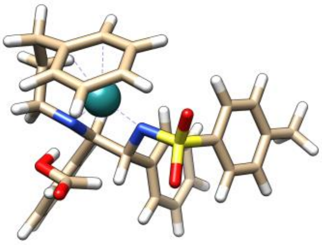

Ru 1.81435 -1.07210 -0.27799
O -1.06253 -1.09833 2.49304
C -6.65686 -1.81500 -1.04954
H -6.76709 -2.53679 -1.86218
H -7.40249 -2.04375 -0.28319
H -6.88909 -0.82074 -1.44778
C -5.26289 -1.83795 -0.47812
C -4.21202 -2.45601 -1.16455
H -4.41243 -2.96155 -2.10556
C -2.91570 -2.42942 -0.65911
H -2.09643 -2.90462 -1.19056
C -2.67214 -1.77511 0.54353
C -3.70137 -1.17648 1.26141
H -3.47841 -0.67973 2.19985
C -4.99188 -1.21271 0.74338
H -5.80045 -0.73350 1.28955
O -0.42343 -3.01146 0.96506
S -0.99510 -1.67346 1.14643
N -0.17120 -0.65837 0.14640
C -0.43619 0.78056 0.34838
H -0.17870 1.06646 1.38129
C 0.52314 1.52160 -0.59717
H 0.15901 1.35644 -1.62742
N 1.84836 0.91737 -0.43063
C 2.88305 1.50193 -1.28752
H 2.65188 2.56005 -1.45556
H 3.84197 1.47958 -0.74752
C 3.06096 0.80832 -2.65327
H 2.07253 0.53373 -3.04351
H 3.49930 1.51712 -3.36448
C 3.97534 -0.43221 -2.60760
H 4.98695 -0.11560 -2.33182
H 4.03950 -0.88092 -3.60455
C 3.50963 -1.47566 -1.62182
C 2.41019 -2.33551 -1.93453
H 1.96321 -2.28194 -2.92214
C 1.87654 -3.23839 -0.96781
H 1.00090 -3.83041 -1.20488
C 2.36899 -3.23149 0.34434
H 1.86505 -3.80441 1.11142
C 3.38568 -2.30334 0.69258
H 3.69131 -2.19847 1.72913
C 4.00011 -1.48503 -0.29501
H 4.74832 -0.76128 0.01131
C -1.86371 1.21863 0.08191
C -2.53577 2.01617 1.00612
H -2.04145 2.28259 1.93714
C -3.83095 2.46577 0.74530
H -4.34352 3.08696 1.47333
C -4.46424 2.11482 -0.44438
H -5.47462 2.45772 -0.64651
C -3.79698 1.31361 -1.37390
H -4.29040 1.02280 -2.29701
C -2.50327 0.87438 -1.11307
H -1.99084 0.22834 -1.82202
C 0.48068 3.01685 -0.32953
C -0.25792 3.85242 -1.16983
H -0.77016 3.42487 -2.02876
C -0.36321 5.21726 -0.90583
H -0.94676 5.85144 -1.56596
C 0.27740 5.76350 0.20434
H 0.19946 6.82601 0.41224
C 1.02104 4.93731 1.04697
H 1.52561 5.35468 1.91282
C 1.11987 3.57289 0.78284
H 1.70632 2.94180 1.44516
C 2.67982 0.16324 2.82985
O 3.29299 -0.30216 3.75646
H 1.59579 -0.01725 2.68407
O 3.24625 0.92124 1.89316
H 2.61568 1.03107 1.11039

TS HCOOH + catalyst oxidised → CO_2 + catalyst reduced

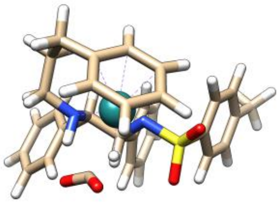

Ru 1.99961 -0.96599 -0.01677
O -1.00317 -1.24653 2.44906
C -6.54078 -2.09398 -1.15522
H -6.74630 -3.06188 -1.62089
H -7.29792 -1.91401 -0.38876
H -6.65729 -1.32498 -1.92731
C -5.15193 -2.05159 -0.57185
C -4.06566 -2.56492 -1.29400
H -4.23964 -3.02883 -2.26193
C -2.77424 -2.49005 -0.78810
H -1.93066 -2.88563 -1.34586
C -2.56362 -1.89246 0.45217
C -3.62327 -1.39892 1.20008
H -3.42754 -0.94621 2.16661
C -4.91411 -1.48164 0.68008
H -5.74697 -1.08512 1.25461
O -0.28709 -3.07302 0.85373
S -0.89453 -1.75616 1.07995
N -0.11739 -0.68775 0.09006
C -0.42951 0.71845 0.40091
H -0.18196 0.94854 1.45088
C 0.48120 1.57681 -0.49414
H 0.24627 1.33475 -1.53831
N 1.87808 1.15500 -0.23294
C 2.87529 1.80615 -1.10921
H 2.61377 2.86645 -1.20182
H 3.83847 1.75277 -0.58815
C 2.99524 1.18291 -2.50193
H 2.00115 0.90688 -2.87393
H 3.38145 1.94366 -3.18748
C 3.93858 -0.03034 -2.56279
H 4.93915 0.27827 -2.24257
H 4.02282 -0.37184 -3.60031
C 3.47838 -1.18549 -1.71179
C 2.29572 -1.89460 -2.06865
H 1.75561 -1.62712 -2.97028
C 1.78123 -2.88534 -1.19804
H 0.81897 -3.34050 -1.40134
C 2.44228 -3.21195 0.00756
H 1.98182 -3.89904 0.70430
C 3.61405 -2.49999 0.35599
H 4.05180 -2.63632 1.33948
C 4.15857 -1.51998 -0.51079
H 5.03995 -0.96347 -0.21204
C -1.87200 1.12154 0.15177
C -2.57946 1.83237 1.11910
H -2.10371 2.05189 2.07165
C -3.88893 2.24809 0.87456
H -4.43128 2.79932 1.63661
C -4.49990 1.94985 -0.34094
H -5.52191 2.26434 -0.52975
C -3.79629 1.23512 -1.31305
H -4.27276 0.98408 -2.25646
C -2.48842 0.82957 -1.06839
H -1.94737 0.24435 -1.80857
C 0.27018 3.05787 -0.25461
C -0.38458 3.83770 -1.20883
H -0.72270 3.37724 -2.13423
C -0.62996 5.18960 -0.97296
H -1.14697 5.78335 -1.72021
C -0.21586 5.77336 0.22244
H -0.40566 6.82555 0.40947
C 0.44191 5.00022 1.17920
H 0.76543 5.44835 2.11336
C 0.68236 3.64788 0.94573
H 1.18926 3.05537 1.70617
C 2.81822 -0.02545 2.43483
O 3.47377 -0.87260 2.98971
H 1.92596 -0.57903 1.67682
O 2.68954 1.19345 2.40860
H 2.10096 1.43513 0.73554

Final adduct: CO_2 + catalyst reduced

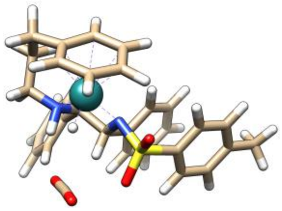

Ru 1.96120 -1.16993 -0.16510
O -1.00550 -1.06111 2.53534
C -6.49891 -1.52324 -1.22291
H -6.67980 -2.35866 -1.90361
H -7.29822 -1.50831 -0.47697
H -6.57148 -0.59526 -1.80192
C -5.14417 -1.62716 -0.57168
C -4.14632 -2.44614 -1.11085
H -4.36775 -3.05229 -1.98568
C -2.87492 -2.48859 -0.54628
H -2.09284 -3.11627 -0.96347
C -2.60161 -1.70925 0.57294
C -3.58598 -0.91509 1.15029
H -3.34760 -0.32783 2.03124
C -4.85097 -0.87662 0.57134
H -5.61979 -0.24235 1.00571
O -0.39485 -3.01377 1.05022
S -0.92326 -1.65503 1.18762
N -0.11184 -0.68952 0.15621
C -0.37345 0.74309 0.34179
H -0.37689 1.00619 1.41149
C 0.78821 1.52292 -0.31581
H 0.75857 1.32001 -1.39370
N 2.05567 0.95088 0.20681
C 3.28926 1.64128 -0.22701
H 3.16825 2.72126 -0.08307
H 4.08584 1.30194 0.44474
C 3.69778 1.36900 -1.67691
H 2.81673 1.41428 -2.32907
H 4.35131 2.18822 -1.99459
C 4.45440 0.04683 -1.89194
H 5.32877 0.02524 -1.23175
H 4.83110 0.01660 -2.92144
C 3.61204 -1.17982 -1.64719
C 2.49702 -1.44544 -2.52124
H 2.31206 -0.79727 -3.37229
C 1.60545 -2.45687 -2.19424
H 0.69670 -2.58643 -2.77326
C 1.82439 -3.28148 -1.04503
H 1.07345 -3.99455 -0.73104
C 3.00967 -3.13829 -0.30266
H 3.18561 -3.77670 0.55487
C 3.93606 -2.09939 -0.61974
H 4.83074 -1.95908 -0.02449
C -1.68702 1.21819 -0.26506
C -2.45060 2.19398 0.37690
H -2.10977 2.59745 1.32788
C -3.64543 2.64326 -0.18374
H -4.23253 3.39882 0.32960
C -4.08929 2.11502 -1.39470
H -5.02527 2.45477 -1.82819
C -3.32888 1.14041 -2.04304
H -3.67744 0.71132 -2.97829
C -2.13251 0.69959 -1.48296
H -1.55193 -0.08736 -1.95845
C 0.66040 3.01416 -0.08450
C 0.36393 3.86873 -1.14605
H 0.23382 3.45426 -2.14304
C 0.20728 5.23844 -0.93427
H -0.03238 5.89025 -1.76850
C 0.35344 5.76609 0.34646
H 0.23261 6.83152 0.51471
C 0.65272 4.91827 1.41399
H 0.76335 5.32223 2.41550
C 0.80122 3.55032 1.20043
H 1.01657 2.89606 2.04324
C 1.30034 -0.43203 3.69764
O 1.36551 -1.46807 4.20985
H 2.14473 -1.25385 1.37535
O 1.30405 0.64342 3.23469
H 2.01093 1.00905 1.22513

(*R*)-2a

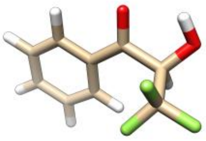

O	0.33577	2.19384	0.10193
C	0.15455	1.04147	-0.23907
C	-1.17983	0.39492	-0.15809
C	1.39360	0.28573	-0.77105
C	-1.45338	-0.85211	-0.73099
C	-2.19873	1.09745	0.49904
O	2.38719	1.18804	-1.12608
H	1.14070	-0.33199	-1.63896
C	-2.73400	-1.39083	-0.64439
C	-3.47351	0.55428	0.58882
H	-1.96678	2.06478	0.93229
C	-3.74185	-0.69042	0.01567
H	-2.94339	-2.35717	-1.09057
H	-4.25919	1.09760	1.10324
H	-4.73876	-1.11448	0.08395
C	1.94465	-0.65450	0.30692

F	3.05090	-1.27022	-0.11831
F	1.05289	-1.60692	0.63675
F	2.24995	0.01874	1.42386
H	-0.67852	-1.41348	-1.24134
H	2.20964	2.00939	-0.63715

(*S*)-2a

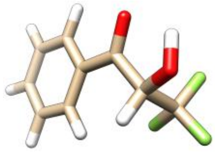

O	-0.33550	2.19413	0.10160
C	-0.15452	1.04166	-0.23929
C	1.17985	0.39493	-0.15822
C	-1.39393	0.28624	-0.77097
C	1.45357	-0.85201	-0.73124
C	2.19860	1.09723	0.49932
O	-2.38757	1.18890	-1.12496
H	-1.14158	-0.33095	-1.63939
C	2.73420	-1.39062	-0.64465
C	3.47336	0.55412	0.58920
H	1.96655	2.06446	0.93277
C	3.74187	-0.69036	0.01576
H	2.94373	-2.35683	-1.09107
H	4.25892	1.09735	1.10391
H	4.73880	-1.11439	0.08408
C	-1.94462	-0.65478	0.30674
F	-3.05139	-1.26985	-0.11816
F	-1.05307	-1.60782	0.63542
F	-2.2905	0.01778	1.42433
H	0.67887	-1.41338	-1.24185
H	-2.20947	2.01003	-0.63584

Catalyst (*S*,*S*)-C4 active form, Cat–H_2

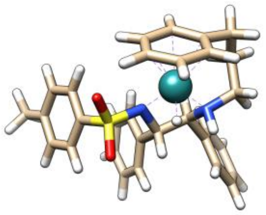

Ru	+2.21085	-0.93319	-0.37494
O	-1.02573	-1.28773	-2.59616
C	-6.14334	-2.12518	1.57976
H	-6.13579	-2.84499	2.40189
H	-6.96331	-2.38408	0.90427
H	-6.36334	-1.13705	2.00033
C	-4.82259	-2.10490	0.85408
C	-3.66900	-2.63590	1.44151
H	-3.73607	-3.10914	2.41795
C	-2.43945	-2.56246	0.79368
H	-1.54036	-2.96706	1.24903
C	-2.36220	-1.95152	-0.45341
C	-3.49866	-1.44507	-1.07297
H	-3.40776	-0.98284	-2.05048
C	-4.72201	-1.52368	-0.41357
H	-5.61151	-1.11275	-0.88501
O	-0.10974	-3.08439	-1.06728
S	-0.76002	-1.78028	-1.23833
N	+0.06931	-0.69240	-0.33606
C	-0.29840	0.70020	-0.61565
H	-0.19564	0.92462	-1.69164
C	+0.69644	1.59250	0.15486
H	+0.59889	1.33693	1.21650
N	+2.07450	1.22345	-0.25349
H	+2.18753	1.50913	-1.22445
C	+3.13169	1.90502	0.53020
H	+2.87389	2.96707	0.62341
H	+4.05431	1.83878	-0.05770
C	+3.36988	1.31154	1.92308
H	+2.41534	1.00699	2.36808
H	+3.77060	2.10542	2.56214
C	+4.35503	0.12952	1.95748
H	+5.31724	0.45317	1.54478
H	+4.53339	-0.15231	3.00097
C	+3.86139	-1.07891	1.19916
C	+2.81398	-1.87016	1.73687
H	+2.41049	-1.64711	2.71864
C	+2.20887	-2.84565	0.91419
H	+1.30769	-3.35201	1.24248
C	+2.72219	-3.15715	-0.36863
H	+2.20852	-3.87444	-0.99369
C	+3.82585	-2.41800	-0.85906

H	+4.20271	-2.61204	-1.85653
C	+4.41552	-1.39733	-0.07496
H	+5.24378	-0.81619	-0.46473
C	-1.70473	1.08721	-0.18379
C	-2.53782	1.80713	-1.03790
H	-2.19021	2.05080	-2.03901
C	-3.81148	2.19843	-0.62280
H	-4.45293	2.75497	-1.29939
C	-4.26145	1.86655	0.65282
H	-5.25595	2.16121	0.97453
C	-3.43269	1.14186	1.51218
H	-3.78490	0.86103	2.50078
C	-2.16094	0.76056	1.09660
H	-1.52458	0.16330	1.74567
C	+0.40705	3.06839	-0.03340
C	-0.07768	3.83465	1.02747
H	-0.23811	3.36298	1.99398
C	-0.37949	5.18445	0.85192
H	-0.76262	5.76478	1.68535
C	-0.19453	5.78458	-0.39171
H	-0.42951	6.83485	-0.53153
C	+0.28899	5.02739	-1.45861
H	+0.42801	5.48546	-2.43288
C	+0.58391	3.67799	-1.27965
H	+0.93709	3.09634	-2.12927
H	+2.07734	-0.65353	-1.90815

PrC: *Si* face on (*S*)

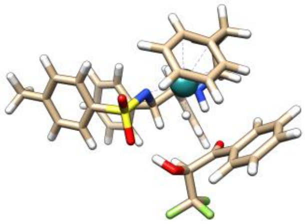

Ru	+1.04170	-1.54554	0.58877
O	-0.75565	0.04236	-2.36904
C	-7.23523	-1.44956	-2.21531
H	-7.60950	-2.47210	-2.12589
H	-7.59487	-1.03286	-3.16004
H	-7.67616	-0.85730	-1.40522
C	-5.73141	-1.40477	-2.13545
C	-5.00408	-2.48433	-1.62308
H	-5.52866	-3.38644	-1.31920
C	-3.62050	-2.41527	-1.49212
H	-3.05152	-3.24708	-1.08776
C	-2.96072	-1.25458	-1.88210
C	-3.65388	-0.17980	-2.42718
H	-3.10958	0.70697	-2.73411
C	-5.03747	-0.26227	-2.54707
H	-5.58976	0.58077	-2.95456
O	-0.65334	-2.45124	-2.01404
S	-1.19963	-1.15445	-1.60674
N	-0.96628	-0.97636	-0.00569
C	-1.25420	0.37757	0.49361
H	-0.70031	1.13679	-0.08573
C	-0.72915	0.44401	1.94318
H	-1.23776	-0.35118	2.50158
N	+0.71745	0.11799	1.92466
H	+1.20177	0.89437	1.46760
C	+1.30651	-0.01556	3.27830
H	+0.97053	0.82959	3.89108
H	+2.39261	0.07747	3.16018
C	+0.97216	-1.33017	3.99413
H	-0.05458	-1.63285	3.75766
H	+0.99920	-1.14365	5.07283
C	+1.92995	-2.49737	3.69100
H	+2.94492	-2.21626	3.99366
H	+1.64030	-3.35972	4.30091
C	+1.93209	-2.89665	2.23549
C	+0.86580	-3.64993	1.69967
H	+0.07358	-4.01283	2.34550
C	+0.77212	-3.79762	0.29051
H	-0.11456	-4.23961	-0.15036
C	+1.79508	-3.34561	-0.57346
H	+1.67722	-3.44265	-1.64440
C	+2.89592	-2.64005	-0.01755
H	+3.66378	-2.22349	-0.66314
C	+2.97477	-2.43005	1.37748
H	+3.80158	-1.86315	1.79107
C	-2.72449	0.76159	0.49040
C	-3.12151	2.03077	0.07153
H	-2.37289	2.73104	-0.29216
C	-4.46505	2.40295	0.11263
H	-4.76135	3.39375	-0.21795
C	-5.42372	1.50179	0.57064
H	-6.47163	1.78561	0.59541
C	-5.03214	0.22918	0.99119
H	-5.77632	-0.48398	1.33441
C	-3.68926	-0.13486	0.95708
H	-3.37965	-1.13430	1.25325
C	-1.02485	1.78085	2.59401
C	-2.02256	1.88161	3.56502

H	-2.56716	0.98916	3.86448
C	-2.34313	3.11494	4.12989
H	-3.12673	3.17925	4.87833
C	-1.66095	4.26220	3.72953
H	-1.90806	5.22433	4.16703
C	-0.65968	4.16893	2.76313
H	-0.12446	5.05819	2.44504
C	-0.34370	2.93617	2.19535
H	+0.43539	2.88474	1.43666
H	+1.44103	-0.43772	-0.42658
O	+2.38323	2.08772	0.31253
C	+2.71805	1.56728	-0.74083
C	+3.97880	0.77321	-0.83664
C	+1.79474	1.77929	-1.95903
C	+4.50417	0.30023	-2.04520
C	+4.65374	0.50868	0.36276
O	+1.80223	0.74536	-2.88480
H	+0.79337	1.94899	-1.54275
C	+5.68799	-0.43774	-2.04180
C	+5.82811	-0.23303	0.36194
H	+4.23637	0.89604	1.28742
C	+6.34594	-0.71168	-0.84468
H	+6.09282	-0.80226	-2.98027
H	+6.34580	-0.43501	1.29484
H	+7.26346	-1.29220	-0.84912
C	+2.18366	3.07222	-2.67666
F	+2.22097	4.11156	-1.82628
F	+1.30808	3.36774	-3.64226
F	+3.39803	2.97707	-3.24475
H	+3.98787	0.49271	-2.97660
H	+0.92790	0.30401	-2.83326

TS: *Si* face on (*S*)

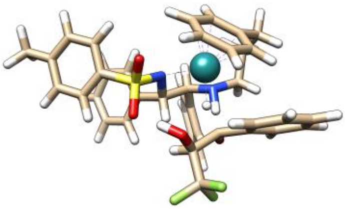

Ru	-1.04385	-0.51455	-1.39013
O	+0.95010	-1.93009	1.61805
C	+7.38949	-2.33807	-0.01472
H	+7.74222	-2.84874	-0.91377
H	+7.81617	-2.84138	0.85704
H	+7.78448	-1.31577	-0.03050
C	+5.88474	-2.31424	0.05063
C	+5.10959	-2.57419	-1.08446
H	+5.59954	-2.84130	-2.01705
C	+3.72162	-2.49086	-1.03484
H	+3.11574	-2.68883	-1.91385
C	+3.10706	-2.14669	0.16412
C	+3.84971	-1.91855	1.31715
H	+3.34008	-1.66746	2.24136
C	+5.23653	-2.00049	1.24991
H	+5.82694	-1.80483	2.14135
O	+0.80657	-3.08028	-0.61172
S	+1.33252	-1.96890	0.18904
N	+0.95869	-0.58227	-0.58501
C	+1.25762	0.65682	0.15661
H	+0.73660	0.67099	1.12860
C	+0.67489	1.80291	-0.69389
H	+1.16161	1.75378	-1.67766
N	-0.76736	1.51377	-0.87555
H	-1.22195	1.54721	0.10028
C	-1.48747	2.51952	-1.68255
H	-1.18695	3.51654	-1.34180
H	-2.55224	2.40286	-1.44651
C	-1.27813	2.40922	-3.19403
H	-0.22916	2.17541	-3.41301
H	-1.47502	3.38749	-3.64387
C	-2.20805	1.38375	-3.86224
H	-3.24814	1.67364	-3.68018
H	-2.05175	1.39853	-4.94689
C	-1.99284	-0.02213	-3.36768
C	-0.74456	-0.67045	-3.62511
H	+0.02226	-0.15848	-4.19622
C	-0.47887	-1.93187	-3.04990
H	+0.51099	-2.36664	-3.12880
C	-1.45838	-2.60433	-2.27740
H	-1.21104	-3.53447	-1.78510
C	-2.68528	-1.96026	-2.03463
H	-3.40685	-2.41371	-1.36565
C	-2.97315	-0.68317	-2.58747
H	-3.90771	-0.18818	-2.33914
C	+2.73201	0.92871	0.39542
C	+3.16989	1.36630	1.64444
H	+2.45267	1.46452	2.45585
C	+4.51374	1.67600	1.85533
H	+4.84224	2.01570	2.83275
C	+5.43072	1.54538	0.81504
H	+6.47853	1.77904	0.97863

C	+4.99761	1.10827	-0.43867
H	+5.70972	0.99109	-1.25070
C	+3.65491	0.80935	-0.64709
H	+3.31635	0.44300	-1.61350
C	+0.94443	3.16085	-0.07691
C	+1.87213	4.01924	-0.66939
H	+2.37550	3.71333	-1.58384
C	+2.17675	5.24859	-0.08643
H	+2.90620	5.90287	-0.55359
C	+1.54716	5.63108	1.09608
H	+1.78123	6.58725	1.55360
C	+0.61516	4.77961	1.68993
H	+0.12107	5.07181	2.61138
C	+0.31433	3.54788	1.11182
H	-0.41126	2.88900	1.58499
H	-1.67572	-0.52054	0.27814
O	-1.94132	1.28914	1.40765
C	-2.33373	0.06746	1.26463
C	-3.77511	-0.11980	0.79171
C	-1.79699	-0.89882	2.35603
C	-4.39999	-1.36920	0.71217
C	-4.48189	1.02232	0.40895
O	-1.79614	-2.24819	1.98815
H	-0.78536	-0.54215	2.57897
C	-5.70155	-1.47042	0.22376
C	-5.78201	0.91946	-0.08511
H	-3.99779	1.98658	0.52742
C	-6.39278	-0.32980	-0.18865
H	-6.18185	-2.44333	0.17350
H	-6.32366	1.81547	-0.37400
H	-7.40688	-0.41383	-0.56734
C	-2.58488	-0.78272	3.66018
F	-2.77393	0.49352	4.02306
F	-1.91998	-1.38933	4.65973
F	-3.79346	-1.36281	3.58665
H	-3.86454	-2.25521	1.04021
H	-0.86655	-2.49633	1.84526

Final adduct: *Si* face on (*S*)

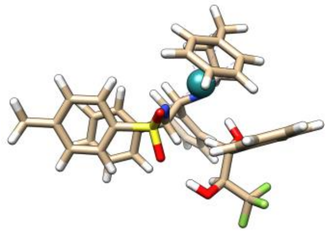

Ru	+0.76270	-1.27447	1.38410
O	-0.63093	-0.44841	-2.36049
C	-7.03720	-2.15004	-2.01769
H	-7.39827	-3.05723	-1.52782
H	-7.33774	-2.18399	-3.06843
H	-7.54383	-1.29384	-1.55835
C	-5.54323	-2.00890	-1.88317
C	-4.82477	-2.78247	-0.96484
H	-5.34726	-3.52317	-0.36534
C	-3.45276	-2.61285	-0.80803
H	-2.89441	-3.20639	-0.08997
C	-2.79618	-1.65957	-1.57871
C	-3.47955	-0.89383	-2.51662
H	-2.93934	-0.15878	-3.10341
C	-4.85111	-1.07556	-2.66141
H	-5.39631	-0.47016	-3.38101
O	-0.42806	-2.71592	-1.27269
S	-1.05359	-1.39094	-1.30323
N	-0.87894	-0.71792	0.16953
C	-1.17376	0.72523	0.24552
H	-0.51356	1.28731	-0.43568
C	-0.82203	1.14390	1.67921
H	-1.58360	0.69386	2.34170
N	+0.49648	0.57653	1.99944
H	+1.89438	1.27198	0.84224
C	+0.94948	0.90231	3.35173
H	+0.65005	1.92788	3.59601
H	+2.04859	0.88652	3.36401
C	+0.41768	-0.04925	4.44319
H	-0.62421	-0.30678	4.21485
H	+0.41210	0.46666	5.40947
C	+1.25803	-1.33133	4.59833
H	+2.25291	-1.05791	4.96584
H	+0.80425	-1.98292	5.35293
C	+1.41543	-2.10183	3.31142
C	+0.32791	-2.86802	2.78038
H	-0.59898	-2.93346	3.34106
C	+0.43173	-3.50056	1.50909
H	-0.43057	-3.99973	1.08363
C	+1.59375	-3.35033	0.73172
H	+1.62086	-3.72222	-0.28436
C	+2.63931	-2.53554	1.21947
H	+3.49565	-2.32517	0.58773
C	+2.57643	-1.95631	2.51624

H	+3.38326	-1.31241	2.85038
C	-2.60639	1.10554	-0.07682
C	-2.87363	2.12770	-0.98537
H	-2.04580	2.62671	-1.48384
C	-4.18859	2.50670	-1.25965
H	-4.38330	3.30499	-1.96914
C	-5.24675	1.85897	-0.62712
H	-6.27088	2.14753	-0.84408
C	-4.98583	0.83186	0.28291
H	-5.80679	0.31194	0.76853
C	-3.67353	0.46278	0.55854
H	-3.46760	-0.35540	1.24466
C	-0.92434	2.65154	1.83642
C	-2.00000	3.19911	2.53847
H	-2.73407	2.53558	2.99016
C	-2.15295	4.58087	2.64707
H	-2.99847	4.98997	3.19137
C	-1.22275	5.43192	2.05455
H	-1.33703	6.50825	2.13700
C	-0.14342	4.89300	1.35405
H	+0.58693	5.54884	0.89016
C	+0.00522	3.51199	1.24224
H	+0.85287	3.10705	0.69556
H	+1.30872	0.26838	-0.91124
O	+2.50417	1.58310	0.13876
C	+2.27796	0.78261	-0.99815
C	+3.33362	-0.28891	-1.17504
C	+2.08746	1.69191	-2.23307
C	+3.04632	-1.42932	-1.93213
C	+4.58464	-0.15882	-0.56988
O	+1.60623	0.98971	-3.33937
H	+1.41229	2.50498	-1.92069
C	+4.00592	-2.42856	-2.08430
C	+5.54364	-1.16111	-0.72088
H	+4.79024	0.72973	0.01895
C	+5.25693	-2.29810	-1.47761
H	+3.77563	-3.31089	-2.67401
H	+6.51654	-1.05292	-0.25076
H	+6.00346	-3.07761	-1.59525
C	+3.36532	2.37504	-2.70663
F	+4.01364	2.96561	-1.68821
F	+3.08309	3.33479	-3.60557
F	+4.21963	1.52544	-3.28895
H	+2.06492	-1.54169	-2.38716
H	+0.78773	0.52523	-3.09297

Product: *Si* face on (*S*)

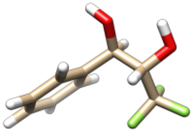

H	-0.33770	2.91524	-0.93816
H	+0.60782	0.86132	-1.61279
O	+0.32446	2.50171	-0.37234
C	+0.30648	1.09304	-0.58220
C	-1.04655	0.48849	-0.29315
C	+1.42631	0.60076	0.34884
C	-1.57438	-0.49099	-1.13524
C	-1.77113	0.89464	0.83100
O	+2.62790	1.25460	0.05301
H	+1.11905	0.76097	1.39380
C	-2.80982	-1.07010	-0.85205
C	-3.00788	0.31959	1.11171
H	-1.36503	1.66852	1.47690
C	-3.52766	-0.66563	0.27191
H	-3.21179	-1.83412	-1.50968
H	-3.56616	0.63858	1.98616
H	-4.49095	-1.11456	0.49216
C	+1.69710	-0.89033	0.19383
F	+0.69359	-1.61906	0.70962
F	+2.81780	-1.24586	0.82782
F	+1.82908	-1.24666	-1.09631
H	-1.00953	-0.81017	-2.00773
H	+2.41628	2.19109	-0.06417

PrC: *Re* face on (*S*)

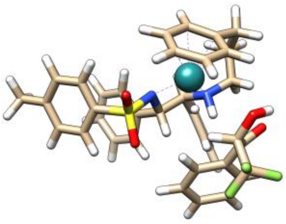

Ru	+0.85901	1.45612	-1.08345
O	-0.77792	0.69299	2.40654
C	-7.31152	1.83702	1.75225
H	-7.71585	2.77196	1.35692
H	-7.67796	1.70909	2.77444
H	-7.71731	1.01460	1.15174
C	-5.80544	1.82547	1.70546
C	-5.10305	2.71168	0.88168
H	-5.65085	3.44978	0.30135
C	-3.71541	2.65663	0.79267
H	-3.16602	3.33808	0.15009
C	-3.02327	1.70716	1.53716
C	-3.69427	0.83774	2.38929
H	-3.12692	0.11491	2.96613
C	-5.08239	0.90108	2.46576
H	-5.61470	0.21127	3.11609
O	-0.75658	2.95710	1.28871
S	-1.25097	1.57781	1.32406
N	-1.01732	0.92047	-0.15366
C	-1.22826	-0.53249	-0.18718
H	-0.66134	-1.03037	0.61752
C	-0.66516	-1.05106	-1.52643
H	-1.20244	-0.53722	-2.33253
N	+0.75819	-0.63528	-1.60718
H	+1.24929	-1.10610	-0.84537
C	+1.44497	-1.03647	-2.85796
H	+1.23074	-2.09393	-3.05315
H	+2.51910	-0.94695	-2.66084
C	+1.06271	-0.20449	-4.08523
H	-0.01147	0.01574	-4.07347
H	+1.23908	-0.81917	-4.97407
C	+1.86242	1.09963	-4.24469
H	+2.92924	0.85761	-4.30276
H	+1.58827	1.57240	-5.19454
C	+1.63330	2.08553	-3.12580
C	+0.38224	2.76036	-3.02517
H	-0.38180	2.60745	-3.77984
C	+0.11259	3.51795	-1.87165
H	-0.88156	3.92204	-1.71145
C	+1.10133	3.72984	-0.87544
H	+0.85066	4.26849	0.02791
C	+2.36912	3.12707	-1.02898
H	+3.12974	3.23580	-0.26417
C	+2.65139	2.32505	-2.16438
H	+3.61568	1.84014	-2.26293
C	-2.68074	-0.96547	-0.06782
C	-3.02928	-2.04537	0.74276
H	-2.25354	-2.55312	1.31277
C	-4.35551	-2.46830	0.82969
H	-4.61500	-3.30849	1.46672
C	-5.34577	-1.80736	0.10579
H	-6.38080	-2.12781	0.17901
C	-5.00292	-0.72530	-0.70737
H	-5.77277	-0.19474	-1.26059
C	-3.67689	-0.31165	-0.79704
H	-3.40547	0.55255	-1.39862
C	-0.84851	-2.54774	-1.66557
C	-1.73370	-3.07732	-2.60395
H	-2.28695	-2.40452	-3.25496
C	-1.93072	-4.45539	-2.69396
H	-2.62793	-4.85455	-3.42386
C	-1.23810	-5.31675	-1.84559
H	-1.39112	-6.38909	-1.91433
C	-0.34979	-4.79380	-0.90492
H	+0.19150	-5.45523	-0.23459
C	-0.15985	-3.41804	-0.81598
H	+0.52078	-3.02308	-0.06460
H	+1.50200	0.88974	0.21606
O	+4.01679	-0.98572	-0.49959
C	+3.55887	-0.71874	0.59743
C	+2.67835	-1.66097	1.33287
C	+3.99242	0.61184	1.23915
C	+1.62284	-1.21204	2.13385
C	+2.92887	-3.03408	1.19348
O	+4.67663	1.38949	0.31012
H	+3.11832	1.15581	1.61587
C	+0.80419	-2.14043	2.77465
C	+2.13794	-3.95191	1.87515
H	+3.75089	-3.35945	0.56329
C	+1.06971	-3.50441	2.65857
H	-0.03898	-1.77687	3.35372
H	+2.34570	-5.01389	1.79025
H	+0.44030	-4.22413	3.17345
C	+4.91560	0.33815	2.43093
F	+4.26614	-0.28045	3.42821
F	+5.41687	1.47660	2.91587
F	+5.94888	-0.44421	2.07220
H	+1.37911	-0.15737	2.22524
H	+4.96510	0.78363	-0.39309

TS: *Re* face on (*S*)

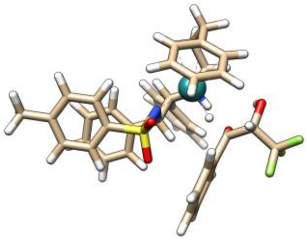

Ru	+0.97067	-0.70456	1.50838	H	+3.43641	-2.44444	1.70796
O	-1.13102	-1.45487	-1.99733	C	+2.58934	-1.02729	3.09004
C	-7.33269	-2.09625	0.24729	H	+3.51000	-0.46096	3.16624
H	-7.73698	-1.07806	0.27187	C	-2.38913	1.14000	-0.66311
H	-7.59594	-2.58333	1.18932	C	-2.65916	1.48199	-1.98617
H	-7.83350	-2.62873	-0.56568	H	-1.84958	1.46401	-2.71165
C	-5.83974	-2.06770	0.04170	C	-3.95600	1.81605	-2.37960
C	-4.96149	-2.23827	1.11853	H	-4.15519	2.07857	-3.41410
H	-5.36333	-2.42724	2.11081	C	-4.99322	1.80392	-1.45010
C	-3.58480	-2.16433	0.93429	H	-6.00368	2.05705	-1.75673
H	-2.90652	-2.27483	1.77550	C	-4.73096	1.45224	-0.12428
C	-3.07768	-1.91713	-0.33706	H	-5.53815	1.41909	0.60198
C	-3.92710	-1.75981	-1.42562	C	-3.43623	1.12518	0.26393
H	-3.50244	-1.55486	-2.40295	H	-3.23954	0.81408	1.28761
C	-5.30335	-1.83825	-1.22819	C	-0.56296	3.23710	0.31556
H	-5.97273	-1.70156	-2.07395	C	-1.52884	4.11519	0.81070
O	-0.72036	-3.00032	-0.03747	H	-2.22786	3.76833	1.56838
S	-1.30669	-1.76485	-0.57706	C	-1.61800	5.41988	0.32772
N	-0.83194	-0.53118	0.38545	H	-2.37824	6.08980	0.71703
C	-0.97245	0.79707	-0.23379	C	-0.73372	5.85863	-0.65552
H	-0.31444	0.88664	-1.11467	H	-0.79930	6.87396	-1.03377
C	-0.50705	1.81036	0.82281	C	+0.23573	4.98741	-1.15212
H	-1.17969	1.71098	1.68267	H	+0.92817	5.32377	-1.91758
N	+0.84872	1.40698	1.27649	C	+0.32149	3.68127	-0.67394
H	+1.50442	1.56346	0.47672	H	+1.08323	3.00920	-1.06475
C	+1.34797	2.22316	2.40401	H	+1.80356	-0.54544	0.00814
H	+1.17130	3.27994	2.17303	O	+2.61461	1.34058	-0.75639
H	+2.43383	2.07527	2.44901	C	+2.63383	0.07633	-0.93628
C	+0.71903	1.88157	3.75829	C	+2.01221	-0.51323	-2.17518
H	-0.34577	1.65544	3.62992	C	+3.95954	-0.57835	-0.45306
H	+0.77014	2.76878	4.39728	C	+1.86862	-1.89322	-2.33307
C	+1.41217	0.72530	4.49803	C	+1.61815	0.34896	-3.19715
H	+2.44868	1.00728	4.71054	O	+4.36943	0.04571	0.72764
H	+0.91756	0.56528	5.46220	H	+3.84359	-1.65208	-0.28364
C	+1.40416	-0.56941	3.72577	C	+1.36142	-2.41066	-3.51718
C	+0.19626	-1.30489	3.57215	C	+1.09570	-0.17098	-4.37985
H	-0.71738	-0.95127	4.03802	H	+1.74490	1.41812	-3.05599
C	+0.17174	-2.43854	2.72212	C	+0.97557	-1.54904	-4.54371
H	-0.76556	-2.93403	2.49810	H	+1.23468	-3.48251	-3.62751
C	+1.33911	-2.89240	2.07050	H	+0.78976	0.50057	-5.17629
H	+1.27615	-3.71513	1.37066	H	+0.56577	-1.95297	-5.46418
C	+2.53924	-2.15923	2.24590	C	+5.08032	-0.42747	-1.48853

F	+4.81779	-1.08940	-2.62415
F	+6.22715	-0.92976	-0.99673
F	+5.30820	0.85449	-1.80189
H	+2.11325	-2.56518	-1.51271
H	+4.08896	0.97307	0.61180

Final adduct: *Re* face on (*S*)

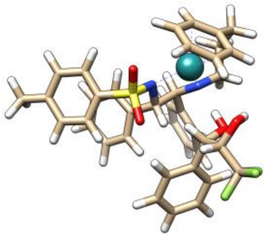

Ru	+0.79428	2.27095	-0.21641
O	-0.58335	-0.08539	2.43272
C	-7.22725	-0.33826	1.98353
H	-7.85748	0.55231	2.03952
H	-7.49079	-0.99943	2.81363
H	-7.46887	-0.86555	1.05348
C	-5.76431	0.01920	2.01305
C	-5.33737	1.33243	1.79314
H	-6.07483	2.11646	1.64286
C	-3.98154	1.64645	1.75794
H	-3.64247	2.66284	1.58349
C	-3.04878	0.63351	1.94912
C	-3.44531	-0.67469	2.20683
H	-2.69304	-1.44113	2.36342
C	-4.80262	-0.97338	2.23289
H	-5.12212	-1.99693	2.41141
O	-1.16067	2.36705	2.38919
S	-1.31547	1.02863	1.80748
N	-0.95284	1.15019	0.21330
C	-0.96804	-0.10278	-0.56794
H	-0.27770	-0.84524	-0.13417
C	-0.41241	0.29109	-1.94773
H	-1.13954	0.99582	-2.39149
N	+0.86273	0.98303	-1.70435
H	+2.15509	-0.09629	-0.88378
C	+1.58044	1.37576	-2.91790
H	+1.39074	0.63684	-3.70410
H	+2.66083	1.33915	-2.71170
C	+1.22450	2.77718	-3.45221
H	+0.14310	2.93353	-3.35035
H	+1.45424	2.82636	-4.52221
C	+1.99681	3.91162	-2.75226
H	+3.06605	3.78854	-2.95582
H	+1.69798	4.87801	-3.17278
C	+1.78396	3.93783	-1.25972
C	+0.52722	4.36166	-0.72018
H	-0.24522	4.71988	-1.39301
C	+0.26266	4.27679	0.67452
H	-0.72421	4.51326	1.05269
C	+1.21949	3.72811	1.54737
H	+0.95802	3.54658	2.58121
C	+2.42453	3.22337	1.01197
H	+3.10804	2.67093	1.64803
C	+2.72998	3.37489	-0.37283
H	+3.65184	2.95994	-0.76766
C	-2.31620	-0.77652	-0.73407
C	-2.40690	-2.16403	-0.61752
H	-1.51770	-2.73695	-0.35935
C	-3.62289	-2.81297	-0.83096
H	-3.68078	-3.89333	-0.73918
C	-4.75886	-2.07569	-1.15894
H	-5.70843	-2.57802	-1.31868
C	-4.67194	-0.68717	-1.27978
H	-5.55644	-0.10540	-1.52391
C	-3.45480	-0.04331	-1.07642
H	-3.38572	1.03957	-1.14681
C	-0.32573	-0.91167	-2.86830
C	-1.26422	-1.07093	-3.88990
H	-2.02071	-0.30474	-4.04336
C	-1.25879	-2.21221	-4.69077
H	-2.00053	-2.32487	-5.47546
C	-0.30831	-3.20872	-4.47738
H	-0.30436	-4.10075	-5.09602
C	+0.63615	-3.05405	-3.46255
H	+1.37801	-3.82639	-3.28140
C	+0.62839	-1.91170	-2.66585
H	+1.36634	-1.80566	-1.87768
H	+1.45232	-0.37411	1.10365
O	+2.85610	-0.59932	-0.40237
C	+2.30667	-1.01543	0.83628
C	+1.82965	-2.45450	0.79810
C	+3.36672	-0.71111	1.92593
C	+0.79359	-2.86326	1.64105
C	+2.41657	-3.37649	-0.07201
O	+3.94540	0.53979	1.66141
H	+2.87815	-0.67431	2.90504

C	+0.35355	-4.18670	1.61671
C	+1.97209	-4.69578	-0.09721
H	+3.21624	-3.04731	-0.72940
C	+0.93977	-5.10504	0.74755
H	-0.45516	-4.49609	2.27224
H	+2.43292	-5.40731	-0.77604
H	+0.59329	-6.13368	0.72554
C	+4.48651	-1.74556	2.04234
F	+4.04126	-2.91650	2.52253
F	+5.44124	-1.31506	2.88190
F	+5.07960	-1.98483	0.85857
H	+0.31856	-2.13628	2.29518
H	+4.03762	0.56012	0.69303

Product: *Re* face on (*S*)

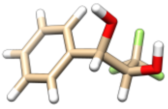

H	-0.17518	2.44761	1.51658
H	-0.23102	2.22714	-0.77704
O	-0.76395	1.76959	1.16642
C	-0.30011	1.35681	-0.10825
C	+1.04903	0.67321	-0.05531
C	-1.44026	0.50522	-0.70168
C	+1.86318	0.66860	-1.18960
C	+1.48391	0.03482	1.10730
O	-2.65192	1.19469	-0.60313
H	-1.23480	0.33264	-1.76350
C	+3.09503	0.01900	-1.16987
C	+2.71961	-0.60835	1.12924
H	+0.84283	0.03126	1.98369
C	+3.52557	-0.62067	-0.00818
H	+3.72091	0.01914	-2.05658
H	+3.05071	-1.10630	2.03506
H	+4.48702	-1.12380	0.01093
C	-1.59055	-0.88774	-0.08254
F	-0.54167	-1.67421	-0.37729
F	-2.68437	-1.49411	-0.56270
F	-1.70869	-0.84854	1.25419
H	+1.53371	1.17852	-2.09260
H	-2.68710	1.58597	0.28263

PrC: *Si* face on (*R*)

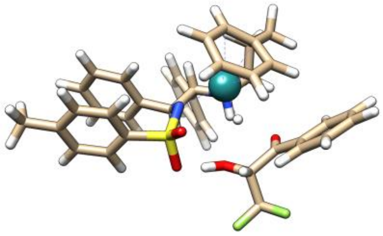

Ru	+0.86330	-0.74043	1.34832
O	-0.81467	-1.13742	-2.24356
C	-7.14263	-2.72089	-0.85336
H	-7.41127	-3.64472	-0.33551
H	-7.60170	-2.73581	-1.84546
H	-7.58206	-1.88358	-0.29900
C	-5.64819	-2.55497	-0.94391
C	-4.79243	-3.26280	-0.09322
H	-5.21026	-3.97882	0.60973
C	-3.41721	-3.05378	-0.12773
H	-2.74746	-3.59014	0.53784
C	-2.89416	-2.13543	-1.03231
C	-3.71778	-1.44738	-1.91658
H	-3.28186	-0.74670	-2.62149
C	-5.09183	-1.65905	-1.86266
H	-5.74462	-1.10907	-2.53568
O	-0.46104	-2.97724	-0.56207
S	-1.15789	-1.74312	-0.92685
N	-1.00813	-0.68051	0.28409
C	-1.50036	0.67302	-0.00866
H	-1.25410	0.97474	-1.03819
C	-0.76810	1.67199	0.92251
H	-1.12046	1.49730	1.94709
N	+0.68129	1.35462	0.88832
H	+0.98154	1.42947	-0.08912
C	+1.53707	2.27669	1.67069
H	+1.25432	3.30926	1.43665
H	+2.55724	2.12985	1.30360
C	+1.50085	2.06980	3.18598
H	+0.46971	1.91788	3.52915
H	+1.83984	3.00123	3.65155
C	+2.40598	0.93140	3.68849

H	+3.42299	1.09243	3.31282
H	+2.45619	0.96815	4.78359
C	+1.93012	-0.43629	3.27107
C	+0.66142	-0.90862	3.76927
H	+0.10542	-0.30702	4.48157
C	+0.11379	-2.06960	3.24496
H	-0.89043	-2.37154	3.52481
C	+0.82976	-2.83639	2.27059
H	+0.36027	-3.68246	1.78557
C	+2.14396	-2.47301	1.92800
H	+2.69774	-3.06665	1.20978
C	+2.72146	-1.27572	2.44971
H	+3.71594	-0.96767	2.14115
C	-3.00546	0.83061	0.17133
C	-3.72488	1.71435	-0.63580
H	-3.20312	2.28436	-1.40195
C	-5.10055	1.86607	-0.47121
H	-5.64752	2.55374	-1.10908
C	-5.77207	1.12946	0.50355
H	-6.84559	1.23794	0.62679
C	-5.05789	0.24852	1.31654
H	-5.57641	-0.33931	2.06875
C	-3.68172	0.10463	1.15366
H	-3.12075	-0.60560	1.75584
C	-1.07217	3.10055	0.51846
C	-1.91492	3.89216	1.29780
H	-2.33948	3.48120	2.21096
C	-2.23804	5.18949	0.89990
H	-2.90260	5.79266	1.51057
C	-1.71091	5.70589	-0.28156
H	-1.95938	6.71539	-0.59378
C	-0.86074	4.92106	-1.06269
H	-0.44474	5.31818	-1.98328
C	-0.54410	3.62361	-0.66828
H	+0.11589	3.01830	-1.28955
H	+1.55002	-0.78705	-0.03117
O	+3.06069	1.80840	-0.94322
C	+3.17962	0.65315	-1.29797
C	+4.26578	-0.21084	-0.74204
C	+2.17632	0.10579	-2.33145
C	+4.29401	-1.59924	-0.90650
C	+5.25181	0.41440	0.03076
O	+0.99095	0.82903	-2.24214
H	+1.99851	-0.96527	-2.17965
C	+5.29360	-2.35262	-0.29464
C	+6.25198	-0.33848	0.63646
H	+5.21381	1.49328	0.14447
C	+6.27069	-1.72565	0.47792
H	+5.30838	-3.43046	-0.42167
H	+7.01685	0.15223	1.22985
H	+7.04802	-2.31651	0.95236
C	+2.76699	0.25294	-3.73387
F	+1.91650	-0.19578	-4.66235
F	+3.90533	-0.46403	-3.84195
F	+3.06369	1.52320	-4.02894
H	+3.53349	-2.10690	-1.49041
H	+0.25495	0.17628	-2.32486

TS: *Si* face on (*R*)

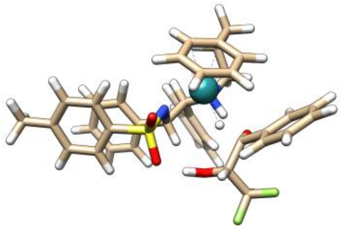

Ru	+0.98183	-1.15511	0.96587
O	-0.96321	-0.69428	-2.47390
C	-7.28915	-2.29735	-1.22529
H	-7.73466	-2.28630	-2.22373
H	-7.71990	-1.46059	-0.66358
H	-7.58382	-3.22407	-0.72743
C	-5.79049	-2.16176	-1.29492
C	-4.95627	-2.95507	-0.50028
H	-5.39384	-3.71174	0.14554
C	-3.57557	-2.78342	-0.52295
H	-2.92639	-3.39479	0.09644
C	-3.02649	-1.81132	-1.35171
C	-3.82903	-1.03266	-2.17836
H	-3.37189	-0.29009	-2.82345
C	-5.20775	-1.21209	-2.14051
H	-5.84379	-0.59320	-2.76823
O	-0.64198	-2.86062	-1.23395
S	-1.26408	-1.53409	-1.29143
N	-0.94471	-0.76885	0.11285
C	-1.29706	0.66479	0.13619
H	-0.82548	1.19960	-0.69420
C	-0.71041	1.25997	1.42606
H	-1.26031	0.82374	2.27001
N	+0.70766	0.84422	1.55364
H	+1.31921	1.34821	0.83295
C	+1.29435	1.24306	2.85222

H	+1.04091	2.29439	3.03063
H	+2.38255	1.19102	2.72901
C	+0.84683	0.39811	4.04725
H	-0.21733	0.15055	3.95943
H	+0.94713	0.99910	4.95654
C	+1.66840	-0.88660	4.23595
H	+2.70906	-0.61747	4.44389
H	+1.29539	-1.43490	5.10800
C	+1.62932	-1.79881	3.03856
C	+0.43219	-2.49165	2.70330
H	-0.45111	-2.37741	3.32244
C	+0.37272	-3.25307	1.50926
H	-0.56944	-3.68052	1.18727
C	+1.50068	-3.38478	0.66946
H	+1.41293	-3.91034	-0.27195
C	+2.68454	-2.69367	1.01354
H	+3.53596	-2.70928	0.34151
C	+2.76451	-1.92378	2.19631
H	+3.67217	-1.37301	2.41769
C	-2.78798	0.95358	0.10746
C	-3.28499	1.96516	-0.71374
H	-2.59817	2.51716	-1.35167
C	-4.64699	2.26720	-0.71866
H	-5.02226	3.05685	-1.36235
C	-5.52237	1.55408	0.09748
H	-6.58409	1.78226	0.08933
C	-5.02981	0.54156	0.92371
H	-5.70902	-0.02736	1.55245
C	-3.66924	0.24954	0.93219
H	-3.28272	-0.55772	1.55041
C	-0.87425	2.76634	1.44538
C	-1.77928	3.36611	2.32185
H	-2.35542	2.74476	3.00421
C	-1.96636	4.74790	2.30980
H	-2.67775	5.20399	2.99127
C	-1.24257	5.53786	1.41896
H	-1.38514	6.61402	1.40649
C	-0.33543	4.94216	0.54157
H	+0.23017	5.55204	-0.15578
C	-0.15025	3.56124	0.54820
H	+0.55763	3.10608	-0.14477
H	+1.70465	-0.30506	-0.46521
O	+2.54723	1.64674	-0.06535
C	+2.59760	0.62959	-0.84683
C	+3.87126	-0.21349	-0.81651
C	+2.01114	0.87595	-2.26714
C	+4.09051	-1.29143	-1.68080
C	+4.84405	0.10883	0.12899
O	+0.72643	1.41056	-2.20682
H	+2.03328	-0.04064	-2.86718
C	+5.26592	-2.03521	-1.59752
C	+6.01354	-0.64561	0.22590
H	+4.66224	0.96489	0.77209
C	+6.22895	-1.71964	-0.63738
H	+5.42783	-2.86463	-2.27923
H	+6.76532	-0.38545	0.96542
H	+7.14322	-2.30101	-0.57054
C	+2.86018	1.90275	-3.01904
F	+2.48078	1.96101	-4.30993
F	+4.16658	1.57606	-3.01135
F	+2.74711	3.13305	-2.51645
H	+3.33779	-1.57671	-2.41059
H	+0.09124	0.69980	-2.42216

Final adduct: *Si* face on (*R*)

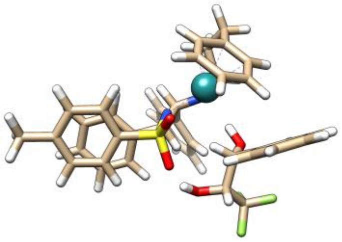

Ru	-0.95213	1.39773	1.21393
O	+0.66775	0.49876	-2.34849
C	+7.10847	2.04982	-1.74537
H	+7.46181	2.99927	-1.33651
H	+7.47158	1.96100	-2.77279
H	+7.56563	1.24119	-1.16384
C	+5.60644	1.94983	-1.68706
C	+4.85783	2.80425	-0.87022
H	+5.36431	3.57902	-0.30075
C	+3.47639	2.67057	-0.77385
H	+2.89323	3.32634	-0.13405
C	+2.84061	1.67313	-1.50536
C	+3.55519	0.82817	-2.34690
H	+3.03226	0.06111	-2.90770
C	+4.93608	0.97304	-2.43022
H	+5.50499	0.30422	-3.07091
O	+0.48952	2.79544	-1.34235
S	+1.08226	1.45494	-1.29518

N	+0.82903	0.84511	0.19268
C	+1.12174	-0.59548	0.34837
H	+0.52453	-1.18578	-0.35944
C	+0.64228	-0.97355	1.75328
H	+1.32813	-0.49786	2.47808
N	-0.71001	-0.41485	1.92312
H	-1.95355	-1.24278	0.66948
C	-1.29830	-0.70865	3.22953
H	-1.01060	-1.72061	3.53582
H	-2.39259	-0.71234	3.12533
C	-0.89884	0.28699	4.33739
H	+0.15676	0.55773	4.20948
H	-0.98606	-0.19693	5.31624
C	-1.77278	1.55599	4.35883
H	-2.79528	1.27428	4.63235
H	-1.41053	2.24298	5.13133
C	-1.81027	2.27810	3.03520
C	-0.68591	3.04623	2.59029
H	+0.17830	3.14750	3.23872
C	-0.66938	3.63222	1.29345
H	+0.22544	4.12926	0.93867
C	-1.74653	3.43843	0.40925
H	-1.67530	3.77930	-0.61546
C	-2.82321	2.62151	0.81647
H	-3.60977	2.37694	0.11034
C	-2.88144	2.08310	2.13188
H	-3.70792	1.43558	2.40542
C	+2.57560	-0.98404	0.16548
C	+2.90446	-2.07122	-0.64380
H	+2.11059	-2.61659	-1.15056
C	+4.23645	-2.45748	-0.80055
H	+4.48112	-3.30523	-1.43314
C	+5.24748	-1.75616	-0.14778
H	+6.28496	-2.05158	-0.27347
C	+4.92274	-0.66950	0.66760
H	+5.70772	-0.11167	1.17056
C	+3.59361	-0.29083	0.82664
H	+3.33754	0.57210	1.43687
C	+0.72276	-2.47559	1.96365
C	+1.57234	-2.99505	2.94185
H	+2.15976	-2.31365	3.55359
C	+1.68550	-4.37240	3.13121
H	+2.35345	-4.76066	3.89398
C	+0.94497	-5.24501	2.33713
H	+1.03063	-6.31788	2.47933
C	+0.09667	-4.73340	1.35437
H	-0.47936	-5.40418	0.72440
C	-0.01459	-3.35791	1.16522
H	-0.67317	-2.98284	0.38581
H	-1.28950	-0.16084	-0.99913
O	-2.51215	-1.54396	-0.07866
C	-2.19477	-0.75277	-1.19545
C	-3.28881	0.24244	-1.53155
C	-1.78880	-1.63696	-2.39663
C	-2.97484	1.34783	-2.33042
C	-4.57876	0.10561	-1.02012
O	-0.46420	-2.08950	-2.29906
H	-1.94689	-1.06656	-3.32248
C	-3.94463	2.30699	-2.61600
C	-5.54992	1.06682	-1.30598
H	-4.80317	-0.75362	-0.39621
C	-5.23606	2.16926	-2.10182
H	-3.69134	3.16478	-3.23181
H	-6.55421	0.95384	-0.90863
H	-5.99222	2.91661	-2.32144
C	-2.64730	-2.89009	-2.52524
F	-2.49376	-3.44602	-3.74202
F	-3.95307	-2.59450	-2.39143
F	-2.34321	-3.83034	-1.62680
H	-1.95757	1.46990	-2.70195
H	+0.10899	-1.31597	-2.44206

Product: *Si* face on (*R*)

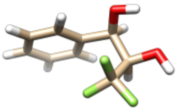

H	+1.59311	2.14229	1.03968
H	+0.24830	2.19683	-0.91710
O	+0.66296	1.88182	1.07893
C	+0.30311	1.37896	-0.18153
C	-1.04758	0.70540	-0.10027
C	+1.39721	0.43871	-0.72356
C	-1.78798	0.50504	-1.26710
C	-1.54765	0.25381	1.12073
O	+2.60103	1.16315	-0.62860
H	+1.15870	0.17473	-1.76327
C	-3.01073	-0.15922	-1.22005
C	-2.77500	-0.40548	1.16773
H	-0.97082	0.42610	2.02295
C	-3.50577	-0.61816	0.00033
H	-3.57971	-0.31088	-2.13184
H	-3.16075	-0.75438	2.12051
H	-4.46052	-1.13284	0.04079
C	+1.54268	-0.89003	0.01904

F	+2.69562	-1.48452	-0.38199
F	+0.54779	-1.73375	-0.27125
F	+1.61412	-0.76496	1.34527
H	-1.41063	0.87648	-2.21792
H	+3.34280	0.61679	-0.91668

PrC: *Re* face on (*R*)

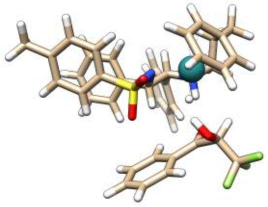

Ru	1.08188	1.60299	0.93931
O	-0.63758	-1.90017	1.19076
C	-7.08060	-0.81311	2.43537
H	-7.44006	-0.10495	3.18580
H	-7.44893	-1.80849	2.69847
H	-7.52919	-0.54018	1.47332
C	-5.57680	-0.79526	2.33799
C	-4.83269	0.26609	2.86503
H	-5.34480	1.06952	3.38835
C	-3.44888	0.30751	2.72352
H	-2.86904	1.13448	3.12257
C	-2.80372	-0.72330	2.04896
C	-3.51554	-1.80373	1.54030
H	-2.98191	-2.59339	1.02110
C	-4.89916	-1.83126	1.68775
H	-5.46473	-2.66389	1.27655
O	-0.46099	-0.22600	3.07640
S	-1.03712	-0.61520	1.78325
N	-0.82005	0.63285	0.73857
C	-1.03291	0.23547	-0.66254
H	-0.41879	-0.64685	-0.91663
C	-0.54462	1.40256	-1.54270
H	-1.10891	2.29310	-1.24051
N	0.88004	1.64658	-1.20856
H	1.41320	0.82837	-1.51439
C	1.46220	2.82117	-1.89883
H	1.19178	2.77255	-2.96024
H	2.55142	2.71713	-1.83310
C	1.03239	4.17638	-1.32645
H	-0.02165	4.13931	-1.02751
H	1.09831	4.91740	-2.12980
C	1.88522	4.68020	-0.14842
H	2.92559	4.78074	-0.47681
H	1.54116	5.68022	0.13685
C	1.82574	3.77637	1.05844
C	0.66612	3.74840	1.86939
H	-0.15831	4.42290	1.66548
C	0.54556	2.74108	2.85770
H	-0.39015	2.61457	3.39138
C	1.61168	1.85926	3.14871
H	1.47928	1.07821	3.88528
C	2.79444	1.93698	2.36990
H	3.60369	1.23483	2.54592
C	2.91549	2.90170	1.34335
H	3.81417	2.94602	0.73765
C	-2.47773	-0.08037	-1.00635
C	-2.79496	-1.22336	-1.73780
H	-1.99852	-1.90749	-2.02219
C	-4.11775	-1.49674	-2.08733
H	-4.35363	-2.39140	-2.65571
C	-5.13444	-0.62656	-1.70038
H	-6.16591	-0.84139	-1.96348
C	-4.82326	0.51790	-0.96276
H	-5.61363	1.19047	-0.64166
C	-3.50180	0.78978	-0.62242
H	-3.25493	1.65952	-0.01798
C	-0.77868	1.14083	-3.01745
C	-1.82119	1.78753	-3.68361
H	-2.44174	2.49683	-3.14109
C	-2.08882	1.51224	-5.02358
H	-2.90805	2.01747	-5.52542
C	-1.30809	0.58741	-5.71414
H	-1.51359	0.37078	-6.75767
C	-0.25988	-0.05733	-5.05804
H	0.35572	-0.77636	-5.58981
C	0.00301	0.21515	-3.71676
H	0.82425	-0.29819	-3.22023
H	1.57128	0.15545	0.64733
O	2.51282	-0.92841	-1.74864
C	2.66052	-1.61720	-0.74900
C	1.86856	-2.84957	-0.52338
C	3.69673	-1.11051	0.28499
C	2.09615	-3.71917	0.55104
C	0.83949	-3.12879	-1.43549
O	3.37921	-1.32938	1.62708
H	3.81568	-0.04306	0.05187
C	1.29571	-4.84639	0.71013
C	0.03909	-4.24833	-1.26613

H	0.67839	-2.45237	-2.26963
C	0.26574	-5.10731	-0.18806
H	1.47101	-5.51393	1.54704
H	-0.76265	-4.45446	-1.96840
H	-0.36471	-5.98053	-0.05015
C	5.06584	-1.74052	0.05609
F	5.98943	-1.15501	0.83136
F	5.44782	-1.58457	-1.22034
F	5.08435	-3.05186	0.32774
H	2.88117	-3.51361	1.26646
H	2.49851	-0.95045	1.79365

TS: *Re* face on (*R*)

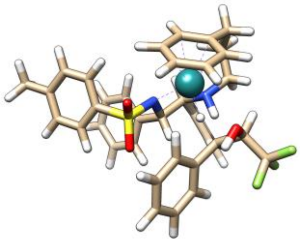

Ru	+0.94346	-0.33617	1.61503
O	-1.07729	-1.83989	-1.65073
C	-7.37263	-1.98938	0.42667
H	-7.84931	-2.66577	-0.28809
H	-7.76443	-0.98335	0.23876
H	-7.67692	-2.28499	1.43358
C	-5.87300	-2.00754	0.27756
C	-5.03669	-2.02676	1.39955
H	-5.47601	-2.06599	2.39309
C	-3.65306	-1.99307	1.25794
H	-3.00671	-1.99524	2.13028
C	-3.09765	-1.93722	-0.01545
C	-3.90540	-1.93938	-1.14686
H	-3.44305	-1.89115	-2.12727
C	-5.28829	-1.97556	-0.99173
H	-5.92504	-1.96128	-1.87291
O	-0.76502	-2.91266	0.62092
S	-1.31770	-1.82879	-0.20549
N	-0.86916	-0.41444	0.48396
C	-1.02236	0.75455	-0.39869
H	-0.39003	0.65227	-1.29600
C	-0.52101	1.96297	0.40776
H	-1.15653	2.04296	1.29812
N	+0.85672	1.65264	0.86939
H	+1.45424	1.58948	0.00872
C	+1.44169	2.70736	1.72389
H	+1.27735	3.67736	1.24127
H	+2.52458	2.53504	1.74516
C	+0.88942	2.74804	3.15154
H	-0.19146	2.56568	3.14056
H	+1.02421	3.76102	3.54379
C	+1.57822	1.77231	4.12001
H	+2.63976	2.03186	4.19215
H	+1.14922	1.89312	5.12090
C	+1.45120	0.32733	3.71054
C	+0.18428	-0.32318	3.76940
H	-0.68452	0.21273	4.13619
C	+0.04959	-1.62846	3.24300
H	-0.93065	-2.08351	3.16286
C	+1.16221	-2.33346	2.72688
H	+1.01541	-3.30050	2.26514
C	+2.41933	-1.69040	2.69659
H	+3.26386	-2.17066	2.21583
C	+2.57729	-0.37962	3.21203
H	+3.54458	0.10894	3.16640
C	-2.45123	1.02005	-0.84137
C	-2.75673	1.16188	-2.19293
H	-1.96664	1.04162	-2.92973
C	-4.06417	1.43692	-2.59767
H	-4.29069	1.54318	-3.65418
C	-5.07601	1.56738	-1.64971
H	-6.09476	1.77411	-1.96382
C	-4.77722	1.42024	-0.29325
H	-5.56451	1.50101	0.45099
C	-3.47235	1.15186	0.10523
H	-3.24405	0.99996	1.15797
C	-0.60968	3.25462	-0.37884
C	-1.53718	4.23076	-0.01047
H	-2.18203	4.05773	0.84830
C	-1.65904	5.40925	-0.74543
H	-2.38953	6.15677	-0.45219
C	-0.84567	5.62149	-1.85648
H	-0.93667	6.53770	-2.43151
C	+0.08471	4.65070	-2.22820
H	+0.72029	4.81026	-3.09372
C	+0.20288	3.46979	-1.49800
H	+0.92815	2.71548	-1.79667
H	+1.79777	-0.56332	0.14611
O	+2.33401	1.03447	-1.26867
C	+2.56746	-0.17547	-0.95880

C	+2.12298	-1.27283	-1.90602
C	+3.95779	-0.40019	-0.29123
C	+1.85811	-2.58115	-1.49165
C	+2.02045	-0.94284	-3.25907
O	+4.19497	-1.69647	0.18622
H	+4.02952	0.28983	0.55460
C	+1.52369	-3.55745	-2.42568
C	+1.67952	-1.91967	-4.19043
H	+2.21439	0.08199	-3.56033
C	+1.44063	-3.22974	-3.77705

H	+1.28602	-4.56164	-2.08980
H	+1.59904	-1.65790	-5.24100
H	+1.16592	-3.98780	-4.50391
C	+5.10114	-0.03861	-1.24741
F	+6.28018	-0.12830	-0.60398
F	+5.01786	1.19443	-1.74712
F	+5.15054	-0.90181	-2.28002
H	+1.82893	-2.81808	-0.43021
H	+4.06824	-2.31990	-0.54534

Final adduct: *Re* face on (*R*)

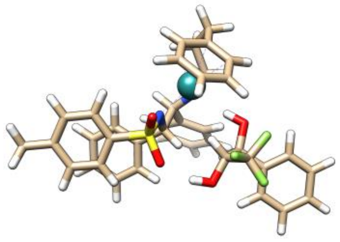

Ru	+0.16401	-1.97687	0.83226
O	-1.01879	0.17074	-2.42812
C	-7.61052	0.53287	-1.79209
H	-8.23425	-0.30040	-1.46039
H	-7.93511	0.82731	-2.79383
H	-7.79760	1.38071	-1.12361
C	-6.14922	0.16534	-1.77958
C	-5.69697	-0.97116	-1.09951
H	-6.41519	-1.61773	-0.60231
C	-4.34186	-1.28236	-1.05138
H	-3.98747	-2.16023	-0.51877
C	-3.43265	-0.44480	-1.68879
C	-3.85420	0.67854	-2.39059
H	-3.11986	1.31201	-2.87633
C	-5.21308	0.97462	-2.43078
H	-5.55074	1.85901	-2.96516
O	-1.55978	-2.24016	-1.91490
S	-1.69262	-0.82336	-1.56769
N	-1.24273	-0.64881	-0.01096
C	-1.02050	0.73736	0.44831
H	-0.17919	1.18848	-0.10517
C	-0.59526	0.62773	1.92574
H	-1.49321	0.34907	2.50730
N	+0.39690	-0.44587	2.02202
H	+2.12253	-0.16214	1.01136
C	+0.90310	-0.68415	3.37461
H	+0.76589	0.21963	3.97761
H	+1.98913	-0.86031	3.32748
C	+0.23359	-1.86611	4.10445
H	-0.84320	-1.84422	3.89391
H	+0.35270	-1.73329	5.18552

C	+0.82008	-3.24194	3.73455
H	+1.86706	-3.27751	4.05449
H	+0.28935	-4.02825	4.28211
C	+0.75368	-3.53466	2.25625
C	-0.49068	-3.88520	1.63810
H	-1.37262	-4.01159	2.25788
C	-0.59506	-4.02879	0.22711
H	-1.56102	-4.20885	-0.22849
C	+0.51606	-3.77092	-0.59651
H	+0.39559	-3.74673	-1.67195
C	+1.72448	-3.34176	-0.00139
H	+2.55200	-3.01678	-0.62183
C	+1.86127	-3.27636	1.41569
H	+2.78776	-2.90298	1.83982
C	-2.21916	1.65485	0.30348
C	-2.09602	2.86766	-0.37055
H	-1.14192	3.12980	-0.82187
C	-3.18419	3.73676	-0.46945
H	-3.07504	4.68055	-0.99477
C	-4.40602	3.39195	0.10261
H	-5.25477	4.06459	0.02288
C	-4.53896	2.17233	0.77154
H	-5.49386	1.88858	1.20474
C	-3.45073	1.31284	0.87234
H	-3.55877	0.35221	1.37032
C	-0.11209	1.97634	2.44354
C	-1.03399	2.87760	2.98551
H	-2.07977	2.59015	3.06381
C	-0.63256	4.14442	3.40406
H	-1.36515	4.83239	3.81460
C	+0.70374	4.52621	3.29542

H	+1.01894	5.51252	3.62108
C	+1.63394	3.62879	2.77442
H	+2.67999	3.90846	2.69528
C	+1.22870	2.36252	2.35448
H	+1.97274	1.66645	1.97865
H	+2.12499	1.66709	-0.21124
O	+2.99135	-0.04309	0.58063
C	+2.82288	0.86305	-0.49097
C	+4.14862	1.49896	-0.84796
C	+2.12021	0.17272	-1.68318
C	+4.16890	2.62587	-1.67564
C	+5.35003	0.96492	-0.38295
O	+1.56463	1.12630	-2.53913
H	+1.34193	-0.48263	-1.26244
C	+5.38061	3.20587	-2.03951
C	+6.56293	1.55011	-0.74633
H	+5.32498	0.09352	0.26206
C	+6.58209	2.66939	-1.57532
H	+5.38755	4.07792	-2.68612
H	+7.49447	1.12850	-0.38086
H	+7.52697	3.12304	-1.85832
C	+3.02760	-0.74576	-2.49990
F	+2.28587	-1.58414	-3.25392
F	+3.79903	-1.52545	-1.70816
F	+3.84279	-0.08686	-3.32251
H	+3.23147	3.03057	-2.04653
H	+0.63758	0.87363	-2.70003

Product: *Re* face on (*R*)

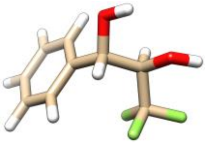

H	-1.10861	2.89370	-0.49679
H	-0.59981	0.91111	-1.63251
O	-0.21647	2.53387	-0.40038
C	-0.29854	1.13954	-0.60023
C	+1.04783	0.52086	-0.31573
C	-1.40179	0.60272	0.32677
C	+1.52771	-0.52136	-1.10877
C	+1.80722	0.97079	0.76663
O	-2.57349	1.29510	-0.03789
H	-1.11838	0.79705	1.37089
C	+2.75024	-1.12237	-0.81489
C	+3.03095	0.37334	1.05771
H	+1.44254	1.80344	1.36078
C	+3.50254	-0.67707	0.27021
H	+3.11632	-1.93343	-1.43629
H	+3.61964	0.72987	1.89713
H	+4.45661	-1.14193	0.49789
C	-1.64341	-0.89850	0.19975
F	-2.81617	-1.21787	0.79070
F	-0.69039	-1.61639	0.80172
F	-1.72387	-1.30201	-1.07619
H	+0.94117	-0.86738	-1.95613
H	-3.29252	1.07452	0.56647

TS-II (Figure 1c)

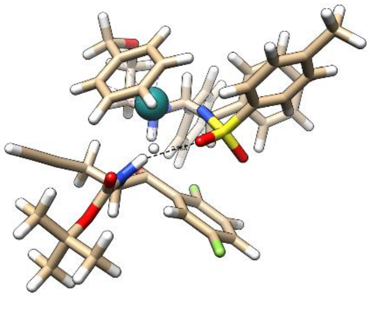

Ru	-0.18033	-0.59623	-1.44312
O	1.15849	1.92001	1.76224
C	4.12275	6.22893	-2.33564
H	5.08775	5.83297	-2.66600
H	3.61210	6.65076	-3.20425
H	4.32546	7.04397	-1.63379
C	3.29324	5.15374	-1.68143
C	1.99240	4.88109	-2.11194
H	1.56588	5.45925	-2.92740
C	1.22552	3.89396	-1.49414
H	0.19668	3.71493	-1.79227
C	1.76959	3.16366	-0.44284

C	3.05358	3.43857	0.02635
H	3.45011	2.89410	0.87845
C	3.80612	4.42459	-0.60112
H	4.80936	4.63728	-0.23963
O	-0.59002	2.15601	-0.06442
S	0.79695	1.88024	0.34439
N	1.24834	0.44880	-0.32036
C	2.10704	-0.39332	0.52997
H	1.67638	-0.51832	1.53153
C	2.17086	-1.78593	-0.12563
H	2.70141	-1.68289	-1.08139
N	0.78415	-2.19853	-0.45192
H	0.19348	-2.27000	0.43799
C	0.67790	-3.54940	-1.04410
H	0.80399	-4.30214	-0.25534
H	-0.34742	-3.65415	-1.41451
C	1.69406	-3.88881	-2.12723
C	0.53108	-2.82445	-3.90549
C	-0.13571	-1.52235	-3.51934
C	0.64548	-0.33852	-3.52155
H	1.70262	-0.39660	-3.75503
C	0.05313	0.88742	-3.12453
H	0.67869	1.76908	-3.03251
C	-1.30821	0.95027	-2.76275
H	-1.74360	1.87177	-2.39296
C	-2.07500	-0.24055	-2.76979
H	-3.10479	-0.21606	-2.42812
C	-1.50801	-1.46915	-3.16652
H	-2.11212	-2.37163	-3.14342
C	3.50907	0.16545	0.67581
C	4.06843	0.35458	1.93846
H	3.47730	0.11624	2.81909
C	5.36002	0.86551	2.06779
H	5.78450	1.01757	3.05532
C	6.10239	1.18553	0.93138
H	7.10714	1.58450	1.03197
C	5.54642	0.99830	-0.33534
H	6.11378	1.26039	-1.22342
C	4.25395	0.49611	-0.45912
H	3.79828	0.39089	-1.44148
C	2.91226	-2.77817	0.75047
C	4.26917	-3.02328	0.52157
H	4.77492	-2.50926	-0.29332
C	4.97936	-3.89997	1.33883
H	6.03436	-4.07611	1.15368
C	4.33393	-4.54601	2.39232
H	4.88381	-5.23101	3.03016
C	2.97984	-4.30783	2.62372
H	2.47111	-4.80474	3.44386

C	2.26961	-3.42642	1.80977
H	1.21634	-3.23743	2.00843
H	-0.97629	-0.58084	0.21471
H	0.75489	-2.79224	-4.97811
H	-0.14970	-3.66830	-3.73180
H	2.70514	-3.90774	-1.71422
H	1.46260	-4.90227	-2.48810
O	1.75178	-2.99384	-3.22572
F	-2.38976	3.02693	3.76692
H	-0.47753	2.33529	5.38060
C	-1.65698	1.90002	3.64014
C	-0.66785	1.63929	4.57210
C	-1.93304	1.05230	2.58494
C	0.06064	0.46914	4.41783
C	-1.20339	-0.13286	2.40260
H	0.85459	0.19874	5.10490
C	-0.20906	-0.38310	3.35600
F	0.57662	-1.47882	3.29203
H	-2.71680	1.34634	1.90186
C	-1.51732	-1.13536	1.28291
O	-1.02679	-2.32046	1.42950
C	-2.99390	-1.07886	0.77412
N	-3.23219	0.15124	0.04705
C	-4.43179	0.76812	-0.14175
O	-5.40198	0.18860	0.58150
O	-4.56458	1.73559	-0.87428
C	-6.76600	0.69712	0.54076
C	-7.50228	-0.25203	1.47966
H	-8.56048	0.01919	1.53011
H	-7.07769	-0.19826	2.48601
H	-7.41732	-1.28089	1.11802
C	-6.80459	2.12626	1.07573
H	-7.84685	2.43961	1.19101
H	-6.32178	2.17190	2.05679
H	-6.29910	2.81466	0.39755
C	-7.33269	0.59378	-0.87265
H	-8.38891	0.88008	-0.85554
H	-7.26059	-0.43927	-1.22428
H	-6.79806	1.24965	-1.56074
H	-2.43157	0.69432	-0.26512
H	-3.65033	-1.10638	1.65045
C	-3.22850	-2.34520	-0.08309
H	-3.39052	-3.19844	0.58080
H	-2.29744	-2.56276	-0.62034
C	-4.30861	-2.24476	-1.06370
C	-5.15129	-2.17595	-1.92741
H	-5.91111	-2.11527	-2.67433

TS-III (Figure 1c)

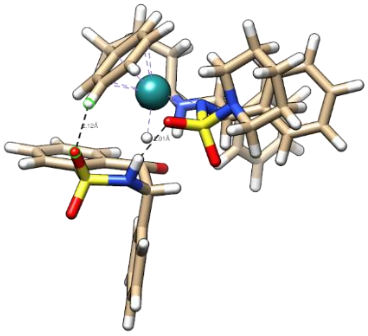

Ru	0.14331	0.78589	-1.43808
O	-1.03649	-2.41783	0.95358
H	-4.63909	-4.65237	-2.51132
C	-5.09735	-3.87574	-1.88305
C	-4.67597	-2.49599	-2.39063
H	-4.92437	-2.37742	-3.45116
C	-3.17537	-2.27965	-2.20513
H	-2.60495	-2.96066	-2.85969
N	-2.81121	-2.50044	-0.80380
C	-3.13264	-3.84972	-0.31607
H	-2.59166	-4.61203	-0.90269
C	-4.63854	-4.06176	-0.43619
H	-4.88823	-5.06461	-0.07432
O	-0.32271	-2.38456	-1.44138
S	-1.28121	-1.94352	-0.41074
N	-1.40690	-0.32957	-0.49689
C	-1.92152	0.26204	0.75229
H	-1.33102	-0.09080	1.61135
C	-1.72224	1.77997	0.65277
H	-2.38752	2.16034	-0.13234
N	-0.32382	2.02144	0.21844
H	0.33366	1.61440	0.96526
C	0.03340	3.45633	0.14937
H	-0.15786	3.90691	1.12916
H	1.11580	3.48908	-0.00487
C	-0.71086	4.22603	-0.95177
H	-1.48930	3.57660	-1.36040
H	-1.24265	5.07540	-0.51189
C	0.17546	4.75606	-2.09311
C	0.88882	2.39626	-2.89971
C	-0.44930	2.04250	-3.27574
H	-1.21726	2.80483	-3.32785
C	-0.78621	0.70565	-3.53088
H	-1.81926	0.44723	-3.73522
C	0.20020	-0.32027	-3.46384
H	-0.07873	-1.35962	-3.58101
C	1.51201	0.01553	-3.11192
H	2.25462	-0.76938	-2.99175
C	1.85328	1.37410	-2.82648
H	2.86257	1.61384	-2.50205
C	-3.38306	-0.05635	1.01154
C	-3.77037	-0.67607	2.19752
H	-3.01012	-0.95509	2.92234
C	-5.11627	-0.94987	2.44607
H	-5.40558	-1.43648	3.37244
C	-6.08545	-0.60000	1.50868
H	-7.13313	-0.80907	1.70189
C	-5.70214	0.01747	0.31607
H	-6.45251	0.28472	-0.42288
C	-4.35829	0.27972	0.06895
H	-4.05417	0.72604	-0.87657
C	-2.06991	2.46144	1.96053
C	-3.21375	3.25464	2.05346
H	-3.84390	3.38820	1.17690
C	-3.56344	3.85612	3.26235
H	-4.46010	4.46479	3.32440
C	-2.76322	3.67009	4.38713
H	-3.03176	4.13663	5.32976
C	-1.61589	2.88022	4.29894
H	-0.98921	2.73166	5.17277
C	-1.26925	2.27400	3.09377
H	-0.37038	1.66203	3.03062
H	1.19011	0.05733	-0.16537
H	3.03185	2.58692	0.92636
C	3.62558	1.95388	0.27330
C	5.16904	0.24396	-1.29788
C	3.24717	0.61424	0.15609
C	4.73912	2.44191	-0.40448
C	5.50690	1.59041	-1.20391
C	4.04322	-0.22977	-0.62307
H	5.02144	3.48549	-0.30044
H	6.37685	1.96805	-1.73099
H	5.77101	-0.44363	-1.88516
C	1.97203	0.14481	0.86257
O	1.47132	0.95132	1.74171
S	3.71013	-1.98488	-0.75031
C	2.00208	-1.35580	1.23016

H	0.98781	-1.62877	1.53934	H	4.80377	-0.27562	4.86684
N	2.28649	-2.18743	0.04980	H	4.29402	-4.42332	3.87458
O	4.80147	-2.65962	-0.07218	H	5.30360	-2.66609	5.31844
O	3.44547	-2.31493	-2.15268	H	-2.80783	-3.91687	0.72232
H	1.50262	-2.29541	-0.60307	H	-5.14244	-3.33267	0.21160
C	2.95926	-1.67446	2.37710	H	-6.18245	-3.99602	-1.96413
C	4.64637	-2.39000	4.49953	H	-5.21268	-1.72302	-1.82870
C	3.24513	-3.01922	2.63736	H	-2.90305	-1.24971	-2.45749
C	3.52475	-0.69138	3.19108	C	1.28577	3.81912	-2.59161
C	4.36726	-1.05118	4.24474	H	-0.46956	5.04356	-2.93220
C	4.07843	-3.37534	3.69106	H	2.10919	3.78049	-1.86956
H	2.82711	-3.78201	1.98798	H	1.71421	4.24937	-3.50572
H	3.29590	0.35455	3.01959	H	0.66617	5.68085	-1.77031

10 References

(1) Kišić, A.; Stephan, M.; Mohar, B. Ansa-Ruthenium(II) Complexes of $R_2NSO_2DPEN-(CH_2)_n(\eta^6$-Aryl) Conjugate Ligands for Asymmetric Transfer Hydrogenation of Aryl Ketones. *Adv. Synth. Catal* **2015**, *357* (11), 2540–2546. https://doi.org/10.1002/adsc.201500288.

(2) Cotman, A. E.; Lozinšek, M.; Wang, B.; Stephan, M.; Mohar, B. *trans* -Diastereoselective Ru(II)-Catalyzed Asymmetric Transfer Hydrogenation of α-Acetamido Benzocyclic Ketones via Dynamic Kinetic Resolution. *Org. Lett.* **2019**, *21* (10), 3644–3648. https://doi.org/10.1021/acs.orglett.9b01069.

(3) Dutta, A. K.; Santra, S.; Harutyunyan, A.; Das, B.; Lisieski, M. J.; Xu, L.; Antonio, T.; Reith, M. E. A.; Perrine, S. A. D-578, an Orally Active Triple Monoamine Reuptake Inhibitor, Displays Antidepressant and Anti-PTSD like Effects in Rats. *Eur. J. Pharmacol.* **2019**, *862*, 172632. https://doi.org/10.1016/j.ejphar.2019.172632.

(4) Shinji, O.; Tomoyuki, I.; Hiroyuki, O.; Seiichi, K.; Takeshi, M. Preparation of (Heterocyclyl)(Propargyloxy)Acetamide Compounds as Agricultural and Horticultural Fungicides. JP2007297310 A, November 15, 2007.

(5) Kamitori, Y.; Hojo, M.; Masuda, R.; Fujitani, T.; Ohara, S.; Yokoyama, T. Electrophilic Substitution at Azomethine Carbon Atoms. Reaction of Aromatic Aldehyde Hydrazones with Trifluoroacetic Anhydride. *J. Org. Chem.* **1988**, *53* (1), 129–135. https://doi.org/10.1021/jo00236a026.

(6) Barrett, I. C.; Langille, J. D.; Kerr, M. A. Facile Preparation of Hydrazones by the Treatment of Azides with Hydrazines Catalyzed by $FeCl_3 \cdot 6H_2O$. *J. Org. Chem.* **2000**, *65* (19), 6268–6269. https://doi.org/10.1021/jo000708w.

(7) Said, S. B.; Skarżewski, J.; Młochowski, J. Conversion of Aldehydes into Nitriles via Oxidation of Their Dimethylhydrazones. *Synthesis* **1989**, *1989* (3), 223–224. https://doi.org/10.1055/s-1989-27210.

(8) Dubrovskiy, A. V.; Larock, R. C. Synthesis of *o*-(Dimethylamino)Aryl Ketones, Acridones, Acridinium Salts, and 1H-Indazoles by the Reaction of Hydrazones and Arynes. *J. Org. Chem.* **2012**, *77* (24), 11232–11256. https://doi.org/10.1021/jo302378w.

(9) Makonen, B.; Van N, N. Preparation of Biphenyls and Biheteroaryls End-Capped with Amino Acid or Peptide Derivates as Hepatitis C Virus Inhibitors. US8329159 B2, December 11, 2012.

(10) Kawase, M.; Kurihara, T. A Convenient Synthesis of α-Trifluoremethylated and α-Perfluoroalkylated Acyloins from α-Hydroxy Acids. *Tetrahedron Lett.* **1994**, *35* (44), 8209–8212. https://doi.org/10.1016/0040-4039(94)88284-3.

(11) Kawase, M.; Saito, S.; Kurihara, T. Convenient Synthesis of α-Trifluoromethylated Acyloins from α-Hydroxy or α-Amino Acids. *Chem. Pharm. Bull.* **2000**, *48* (9), 1338–1343. https://doi.org/10.1248/cpb.48.1338.

(12) Maekawa, H.; Kudo, M.; Nishiyama, Y.; Shimizu, K.; Abe, M. Facile Synthesis of Aromatic Unsymmetrical Fluorine-Containing Acyloins through the Reductive Trifluoroacetylation of Benzaldehyde and Transposition of the Carbonyl Group. *Tetrahedron* **2014**, *70* (12), 2081–2087. https://doi.org/10.1016/j.tet.2014.02.016.

(13) Kawase, M.; Miyamae, H.; Kurihara, T. A General Method for the Preparation of 5-Trifluoromethylated Oxazoles from α-Amino Acids. *Chem. Pharm. Bull.* **1998**, *46* (5), 749–756. https://doi.org/10.1248/cpb.46.749.

(14) Ramanjaneyulu, B. T.; Mahesh, S.; Vijaya Anand, R. *N*-Heterocyclic Carbene Catalyzed Highly Chemoselective Intermolecular Crossed Acyloin Condensation of Aromatic Aldehydes with Trifluoroacetaldehyde Ethyl Hemiacetal. *Org. Lett.* **2015**, *17* (1), 6–9. https://doi.org/10.1021/ol502581b.

(15) Iskra, J.; Bonnet-Delpon, D.; Bégué, J.-P. Ring-Opening of 1-CF_3-Substituted Epoxy Ethers with Carboxylic, Thiocarboxylic, and Phosphinic Acids in Basic Medium and in Hexafluoro-2-Propanol. *Eur. J. Org. Chem.* **2002**, *2002* (20), 3402–3410. https://doi.org/10.1002/1099-0690(200210)2002:20<3402::AID-EJOC3402>3.0.CO;2-M.

(16) Hur, Y.; Lee, H.-J.; Kim, E.-K.; Park, J.-H.; Joo, J.-E.; Kang, H.-W.; Hong, H.-N.; Kim, D.-K.; Hyun, K.-H.; Ahn, K.-K. Imide-Containing Benzothiazole Derivative or Its Salt and Pharmaceutical Composition Comprising the Same. WO2013043002A1, March 28, 2013. https://patents.google.com/patent/WO2013043002A1/en (accessed 2022-03-07).

(17) CrysAlisPro Software System, Rigaku Corporation, Wrocław, Poland, 2022.

(18) Clark, R. C.; Reid, J. S. The Analytical Calculation of Absorption in Multifaceted Crystals. *Acta Crystallogr. A* **1995**, *51* (6), 887–897. https://doi.org/10.1107/S0108767395007367.

(19) Dolomanov, O. V.; Bourhis, L. J.; Gildea, R. J.; Howard, J. A. K.; Puschmann, H. *OLEX2*: A Complete Structure Solution, Refinement and Analysis Program. *J. Appl. Crystallogr.* **2009**, *42* (2), 339–341. https://doi.org/10.1107/S0021889808042726.

(20) Sheldrick, G. M. Crystal Structure Refinement with *SHELXL*. *Acta Crystallogr. C* **2015**, *71* (1), 3–8. https://doi.org/10.1107/S2053229614024218.

(21) Brandenburg, K. Diamond – Crystal and Molecular Structure Visualization, Crystal Impact GbR, Bonn, Germany, 2005.

(22) Cooper, R. I.; Thompson, A. L.; Watkin, D. J. *CRYSTALS* Enhancements: Dealing with Hydrogen Atoms in Refinement. *J. Appl. Crystallogr.* **2010**, *43* (5), 1100–1107. https://doi.org/10.1107/S0021889810025598.

(23) Parsons, S.; Flack, H. D.; Wagner, T. Use of Intensity Quotients and Differences in Absolute Structure Refinement. *Acta Crystallogr. B* **2013**, *69* (3), 249–259. https://doi.org/10.1107/S2052519213010014.

(24) Etter, M. C. Encoding and Decoding Hydrogen-Bond Patterns of Organic Compounds. *Acc. Chem. Res.* **1990**, *23* (4), 120–126. https://doi.org/10.1021/ar00172a005.

(25) Frisch, M. J.; Trucks, G. W.; Schlegel, H. B.; Scuseria, G. E.; Robb, M. A.; Cheeseman, J. R.; Scalmani, G.; Barone, V.; Petersson, G. A.; Nakatsuji, H.; Li, X.; Caricato, M.; Marenich, A. V.; Bloino, J.; Janesko, B. G.; Gomperts, R.; Mennucci, B.; Hratchian, H. P.; Ortiz, J. V.; Izmaylov, A. F.; Sonnenberg, J. L.; Williams-Young, D.; Ding, F.; Lipparini, F.; Egidi, F.; Goings, J.; Peng, B.; Petrone, A.; Henderson, T.; Ranasinghe, D.; Zakrzewski, V. G.; Gao, J.; Rega, N.; Zheng, G.; Liang, W.; Hada, M.; Ehara, M.; Toyota, K.; Fukuda, R.; Hasegawa, J.; Ishida, M.; Nakajima, T.; Honda, Y.; Kitao, O.; Nakai, H.; Vreven, T.; Throssell, K.; Montgomery, J. A., Jr.; Peralta, J. E.; Ogliaro, F.; Bearpark, M. J.; Heyd, J. J.; Brothers, E. N.; Kudin, K. N.; Staroverov, V. N.; Keith, T. A.; Kobayashi, R.; Normand, J.; Raghavachari, K.; Rendell,

A. P.; Burant, J. C.; Iyengar, S. S.; Tomasi, J.; Cossi, M.; Millam, J. M.; Klene, M.; Adamo, C.; Cammi, R.; Ochterski, J. W.; Martin, R. L.; Morokuma, K.; Farkas, O.; Foresman, J. B.; Fox, D. J. Gaussian 16, Revision C.01, 2016.
(26) Zhao, Y.; Truhlar, D. G. Density Functionals with Broad Applicability in Chemistry. *Acc. Chem. Res.* **2008**, *41* (2), 157–167. https://doi.org/10.1021/ar700111a.
(27) Petersson, G. A.; Bennett, A.; Tensfeldt, T. G.; Al-Laham, M. A.; Shirley, W. A.; Mantzaris, J. A Complete Basis Set Model Chemistry. I. The Total Energies of Closed-shell Atoms and Hydrides of the First-row Elements. *J. Chem. Phys.* **1988**, *89* (4), 2193–2218. https://doi.org/10.1063/1.455064.
(28) Petersson, G. A.; Al-Laham, M. A. A Complete Basis Set Model Chemistry. II. Open-shell Systems and the Total Energies of the First-row Atoms. *J. Chem. Phys.* **1991**, *94* (9), 6081–6090. https://doi.org/10.1063/1.460447.
